# Comparative transcriptomic analyses to scrutinize the assumption that genotoxic PAHs exert effects via a common mode of action

**DOI:** 10.1007/s00204-015-1595-5

**Published:** 2015-09-16

**Authors:** S. Labib, A. Williams, C. H. Guo, K. Leingartner, V. M. Arlt, H. H. Schmeiser, C. L. Yauk, P. A. White, S. Halappanavar

**Affiliations:** 1 Environmental Health Science and Research Bureau, Health Canada, Ottawa, ON K1A 0K9 Canada; 2 Analytical and Environmental Sciences Division, MRC-PHE Centre for Environment and Health, King’s College London, London, SE1 9NH UK; 3 Division of Radiopharmaceutical Chemistry, German Cancer Research Center (DKFZ), Im Neuenheimer Feld 280, 69120 Heidelberg, Germany

**Keywords:** Polycyclic aromatic hydrocarbons, Toxicogenomics, Mode of action, Benzo(*a*)pyrene, Complex mixtures

## Abstract

In this study, the accuracy of the assumption that genotoxic, carcinogenic polycyclic aromatic hydrocarbons (PAHs) act via similar mechanisms of action as benzo(*a*)pyrene (BaP), the reference PAH used in the human health risk assessment of PAH-containing complex mixtures, was investigated. Adult male Muta™Mouse were gavaged for 28 days with seven individual, genotoxic PAHs. Global gene expression profiles in forestomach, liver, and lung (target tissues of exposure) were determined at 3 days post-exposure. The results are compared with our previously published results from mice exposed to BaP via the same exposure regimen. Although all PAHs showed enhanced ethoxyresorufin-*O*-deethylase activity, DNA adduct formation, and *lacZ* mutant frequency in the lungs, the unsupervised cluster analysis of differentially expressed genes revealed that the transcriptional changes are both PAH- and tissue-specific, with lung showing the most response. Further bioinformatics-/pathway-based analysis revealed that all PAHs induce expression of genes associated with carcinogenic processes, including DNA damage response, immune/inflammatory response, or cell signaling processes; however, the type of pathways and the magnitude of change varied for each PAH and were not the same as those observed for BaP. Benchmark dose modeling showed transcriptomic data closely reflected the known tumor incidence for the individual PAHs in each tissue. Collectively, the results suggest that the underlying mechanisms of PAH-induced toxicity leading to tumorigenesis are tissue-specific and not the same for all PAHs; based on the tissue type considered, use of BaP as a reference chemical may overestimate or underestimate the carcinogenic potential of PAHs.

**Electronic supplementary material:**

The online version of this article (doi:10.1007/s00204-015-1595-5) contains supplementary material, which is available to authorized users.

## Introduction

Many regulatory agencies recommend the use of a reference chemical approach to assess the hazards posed by chemical mixtures in complex environmental matrices (EFSA 2008; Health Canada 2010; Health Protection Agency 2010; USEPA 2010). For example, the current human health risk assessment approach for evaluating the carcinogenic risks of soils contaminated with mixtures of polycyclic aromatic hydrocarbons (PAHs) involves examination of the cumulative risk posed by a targeted set of PAHs. Benzo(*a*)pyrene (BaP) is the most extensively studied of the carcinogenic PAHs and is used as the point of reference for the carcinogenic potency of the other PAHs.

Human health risk assessment of PAH-contaminated complex environmental mixtures considers the incremental carcinogenic risk of a targeted set of PAHs as toxicological equivalents of BaP (relative potency factor approach). However, to be able to apply this approach, all structurally similar PAHs in a mixture must elicit similar toxicological responses via similar modes/mechanisms of action. The most well-established mode of action of genotoxic, carcinogenic PAHs involves binding and activating the aryl hydrocarbon receptor (AhR), the expression of genes associated with xenobiotic metabolism, and the concurrent metabolism of PAHs. This in turn leads to the production of reactive intermediates (e.g., diol epoxides) that covalently bind DNA and form bulky DNA adducts. When left unrepaired, these DNA adducts can result in mutations in tumor-suppressor genes or oncogenes, which can eventually lead to carcinogenesis, implying a genotoxic mode of action in cancer. Consequently, measurement of DNA adducts and DNA mutations have long been taken as measurable markers of the genotoxic mode of action of PAHs. However, the existing toxicological database (showing evidence for carcinogenicity and the details pertinent to the underlying mechanisms of carcinogenicity) for the individual PAHs is incomplete and thus lacks experimental support for the use of the reference chemical method (Bostrom et al. 2002). In order to strengthen the application of the reference chemical approach, the USEPA (USEPA 2010) has outlined certain considerations that are important for PAH mixture cancer risk assessment, which includes a detailed characterization of toxicological responses elicited by individual PAHs in the mixture.

Recent work from our laboratory examined the levels of DNA damage, mutation, and global gene expression changes in the forestomach (Labib et al. 2013), livers (Malik et al. 2012), and lungs (Labib et al. 2012) of Muta™Mouse males exposed to BaP by oral gavage for 28 consecutive days. The results of these studies revealed that (1) the levels and relationship between bulky DNA adducts and transgene mutant frequencies differ between tissues, and (2) BaP-induced alterations in gene/protein expression associated with a diverse array of cellular signaling pathways/biological functions, including carcinogenesis and the magnitude of change, varied between the tissues. Moreover, compared to the amount of DNA damage and mutations, the observed extent of the transcriptomic response was more closely related to previously reported tumor incidence in each of these tissues (Hakura et al. 1998). These results suggest that the carcinogenic potency and the underlying mode of action (including metabolism, DNA damage, mutation, and pathway perturbations) for BaP differs based on the type of tissue investigated. These results also implied that the current approach of human health risk assessment, which assumes that the toxicological activities of PAHs reflect a single mode of action, may be overgeneralized.

In the present study, we sought to explore the underlying carcinogenic modes of action of several individual PAHs to specifically investigate whether the assumption of similar mode of action is accurate. We exposed Muta™Mouse males to three doses of seven PAH congeners or vehicle for 28 consecutive days via oral gavage. These seven PAHs are known to be genotoxic and constitute the panel of PAHs recommended for routine measurement in contaminated soils for the protection of human and environmental health in the Canadian soil quality guidelines (CCME 2010) and in contaminated food samples (EFSA 2008). Forestomach, livers, and lungs were collected 3 days after the final exposure, and DNA microarrays were employed to generate the gene expression profiles of these tissues. The results were compared to gene expression profiles in forestomach, livers, and lungs of mice previously exposed to BaP (Labib et al. 2012, 2013; Malik et al. 2012). The data were used to (a) identify the cancer-associated biological functions and pathways affected following exposure to different PAHs, (b) evaluate the assumption that all structurally similar PAHs with similar toxicological outcome (i.e., cancer) act via the same mode of action, and (c) determine whether BaP as a reference chemical appropriately represents the toxicity of the other seven PAHs. In addition to the gene expression profiling, DNA adducts and *lac*Z transgene mutant frequencies were measured in lungs to confirm the genotoxic phenotype, and activation of metabolic enzymes in the lungs of mice was measured using the ethoxyresorufin-*O*-deethylase (EROD) assay.

## Methods

### Materials and dose selection

Seven PAHs were individually analyzed in this study: benz(*a*)anthracene (BaA), benzo(*b*)fluoranthene (BbF), benzo(*g*,*h*,*i*)perylene (BghiP), benzo(*k*)fluoranthene (BkF), dibenz(*a*,*h*)anthracene (DBahA), indeno(123-*c*,*d*)pyrene (IP) (Cambridge Isotopes Laboratories, USA), and chrysene (Chr) (Sigma-Aldrich Canada Ltd, Canada). All of these PAHs are classified as possible or probable human carcinogens by IARC and the USEPA (IARC 2010; USEPA 2011), except for BghiP which is classified as a genotoxic PAH. Maximum doses were initially selected based on the maximum tolerated dose of each PAH in a dose-range finding study. This dose was diluted two times, twofold, to obtain two lower doses, except for Chr, which was diluted threefold. The specific doses were as follows: BaA (20, 40, 80 mg/kg-bw/day), BbF (25, 50, 100 mg/kg-bw/day), BghiP (6.25, 12.5, 25 mg/kg-bw/day), BkF (25, 50, 100 mg/kg-bw/day), Chr (17.5, 50, 150 mg/kg-bw/day), DBahA (6.25, 12.5, 25 mg/kg-bw/day), and IP (12.5, 25, 50 mg/kg-bw/day). The results are compared to data derived from experiments using an identical experimental design for BaP exposures (25, 50, 75 mg/kg-bw/day); results from the BaP study were published in Malik et al. (2012) and Labib et al. (2012, 2013). Data from DBahA-exposed livers were also published previously in Malik et al. (2013).

### Muta™Mouse exposures

Animal care, exposures, and tissue collection procedures were described previously (Labib et al. 2012; Lemieux et al. 2011). In brief, adult male Muta™Mouse (transgenic mouse strain 40.6) were exposed by oral gavage for 28 consecutive days to olive oil (vehicle control) or three doses of the seven individual PAHs dissolved in olive oil, according to the established Organization for Economic Co-operation and Development (OECD) guideline for transgenic rodent mutation assays (OECD 2011). Each treatment group contained five animals. Mice were killed by cardiac puncture under isofluorene anesthesia 3 days after the final exposure. No mice showed overt signs of toxicity during the exposure period. Five mice from the BkF group and one from the IP group died as a result of accidental pulmonary puncture during oral gavage. Thus, the final sample size for BkF was 4, 3, 4, and 4 in the control, low-, medium-, and high-dose groups, respectively, at necropsy. The IP control group consisted of only 4 mice at necropsy. The forestomach, left lobe of the liver, and the right lobe of the lung were collected, flash-frozen in liquid nitrogen, and stored at −80 °C until use. Mice were bred, maintained, and treated in accordance with the Canadian Council for Animal Care Guidelines, and all protocols were approved by Health Canada’s Animal Care Committee.

### Tissue RNA extraction and purification

Total RNA was isolated for gene expression analysis using TRIzol reagent (Invitrogen, Canada) and RNeasy Mini Kits following the protocols previously published (Halappanavar et al. 2011; Labib et al. 2012, 2013). For detailed description of methods, please see Online Resource 1A.

### Microarray hybridization and analysis

RNA samples were hybridized to Agilent Sureprint G3 Mouse GE 8 × 60 K microarrays (Agilent Technologies, Canada) following previously published protocols (Labib et al. 2013; Malik et al. 2012) and scanned on the Agilent G2505B scanner. Sample sizes of 5 were used for each treatment and control group, except for the BkF and IP exposures. LOWESS was used to normalize the signal intensities, MAANOVA was used to identify differentially expressed genes, and multiple comparisons were adjusted for using the false discovery rate (FDR) approach, as described in Labib et al. (2013) (Online Resource 1B). All microarray results were deposited in the Gene Expression Omnibus database (http://www.ncbi.nlm.nih.gov/geo/) under the accession number GSE51321. Differentially expressed genes (DEGs) were defined as genes with FDR-adjusted *P* values less than 0.05 (FDR *P* ≤ 0.05) from the pairwise analysis to controls with absolute fold change estimates greater than or equal to 1.5 in either direction (fold change ±1.5). Hierarchical cluster analysis was applied to the filtered data and visualized using a heatmap using R (R Development Core Team 2010) (Online Resource 1C).

### Bioinformatics and pathway analysis

The lists of DEGs (FDR *P* ≤ 0.05, fold change ±1.5) from each PAH were independently analyzed to identify biological functions or processes perturbed in response to the treatment. DAVID (Huang da et al. 2009) Functional Annotation Charts were used to identify gene ontology (GO) terms (biological processes and cellular compartments) associated with the significant genes and to classify the DEGs into biological pathways using KEGG pathways (Kanehisa and Goto 2000). MetaCore (Thomson Reuters, http://www.genego.com/metacore.php) pathway analysis and network process analysis were used for functional classification. The canonical pathway analysis, biological function analysis, and network analysis within Ingenuity Pathway Analysis (IPA, Ingenuity Systems, Redwood City, CA) were used to identify biological pathways and functions associated with the DEGs. Since there was a large redundancy in the annotation of the perturbed biological processes, pathways, and functions, the results were further grouped under parent pathways/processes (Online Resource 2). IPA’s Upstream Regulator analysis was performed to determine the regulatory mechanisms (transcription factors and receptors) operating in response to individual PAHs, and these were compared to BaP. Online Resource 1D describes the criteria used to determine the statistical significance of altered pathways, functions, biological processes, or upstream regulators.

The NextBio meta-analysis function (http://nextbio.com) was used to compare the transcriptomic responses following exposure to individual PAHs and following exposure to BaP. A less stringent non-FDR *P* value cutoff of less than 0.05 and a fold change of ±1.2 was applied. A pairwise comparison was made for each gene between the two datasets (for example, BaP vs BbF). A rank-based enrichment statistic was applied to determine the final correlation score. If the direction of change in the expression of a gene following exposure to a congener PAH was the same as the direction of change in expression of that gene following exposure to BaP, then the correlation was positive.

Since the focus of the study was to compare the carcinogenic potential of PAHs, significantly perturbed pathways and processes were reorganized according to their association with the six hallmarks of cancer (activating invasion and metastasis, enabling replicative immortality, evading growth suppressors, inducing angiogenesis, resisting cell death, sustained proliferative signaling), two emerging hallmarks (deregulating cellular energetics, avoiding immune destruction), and two enabling characteristics (genome instability and mutation, tumor-promoting inflammation), as described in Hanahan and Weinberg (2011). Re-classification was determined by conducting a literature review using search terms related to each significant pathway and each hallmark of cancer. Pathways or processes that could not be classified as cancer-related using these criteria were not used in the subsequent analysis and interpretation of the results.

### DNA extraction from lung tissue

Frozen lung tissues were sliced randomly for isolation of genomic DNA by phenol/chloroform extraction as described previously (Labib et al. 2012; Renault et al. 1997) (Online Resource 1E).

### *Lac*Z mutant frequency in lung tissue

The *lac*Z mutant frequency analysis in lung tissues using the phenyl-β-d-galactopyranoside (P-Gal)-positive selection assay was conducted as described previously (Labib et al. 2012; Lemieux et al. 2011) (Online Resource 1F). Mutant frequency was expressed as the ratio of mutant plaque forming units to total plaque forming units.

### DNA adduct analysis in lung tissue

DNA adduct formation in each sample was determined using the nuclease P1 digestion enrichment version of the ^32^P-post-labeling assay as described previously (Phillips and Arlt 2007, 2014) with minor modifications described in Labib et al. (2012) (Online Resource 1G). The results are expressed as DNA adducts per 10^8^ nucleotides.

### Enzymatic analysis of lung Cyp1a activity

Enzymatic analysis of pulmonary CYP1A activity was assessed via the ethoxyresorufin-*O*-deethylase (EROD) assay. Since the available amount of tissue from each treatment group was small, samples from the same treatment group were pooled (Online Resource 1H).

### Benchmark dose (BMD) calculations

BMD and BMDL (lower confidence limit of BMD) values were modeled for apical endpoint measurements (EROD activity, DNA adduct formation, *lacZ* mutant frequency) in lungs. Further, BMDs were modeled for the total number of DEGs, the number of DEGs related to the hallmarks of cancer, and for the number of hallmarks of cancer perturbed by each PAH in the forestomach, liver, and lung tissues using the USEPA’s Benchmark Dose software BMDS version 2.5.0 (http://www.epa.gov/ncea/bmds/) (Davis et al. 2011). Continuous data were run against five models (Exponential, Hill, Power, Polynomial, and Linear), and proportional (dichotomous) data were run against eight models (Gamma, Logistic, LogLogistic, Probit, LogProbit, Weibull, Multistage, QuantalLinear). The benchmark response (BMR) was set to 10 % extra risk as recommended by the Benchmark Dose Technical Guidance document (USEPA 2012). The best model for each dataset was selected based on the lowest Akaike’s information criterion (AIC) value, excluding models with BMD greater than the highest dose, BMD/BMDL ratios >10, and goodness of fit *P* < 0.1.

## Results

Adult male Muta™Mouse were exposed to three doses of individual PAHs (BaA, BbF, BghiP, BkF, Chr, DBahA, or IP) for 28 consecutive days via oral gavage. The exposure regimen did not cause any overt signs of toxicity, and there was no significant body weight loss in any of the exposed mice compared to vehicle-treated controls. Forestomach, liver, and lung tissues were collected 3 days after the final exposure.

### General overview of the microarray results

The transcriptomic profiles derived from the present study were compared with the profiles of mice exposed to BaP (following the same exposure regimen described for other seven PAHs in this study) to investigate whether BaP, as a reference PAH, accurately represents the toxicity posed by other individual PAHs. Global transcriptomic changes in the forestomach, liver, and lung tissue were characterized.

Table 1 summarizes the total number of DEGs (FDR *P* ≤ 0.05, fold change ±1.5) for each tissue and the different doses tested. Online Resource 3 provides the full lists of DEGs including the gene names, gene accession numbers, *P* values, and fold changes for the forestomach, liver, and lung. The results are presented by tissue type below.

**Table 1:** Total numbers of differentially expressed genes in forestomach, liver, and lung tissues

	BaP*	BaA	BbF	BghiP	BkF	Chr	DBahA	IP
1. **Forestomach**								
*Low*								
Up	9	10	0	0	1	3	0	0
Down	0	0	2	3	0	2	0	2
Total	9	10	2	3	1	5	0	2
*Med*								
Up	119	3	2	4	7	37	1	0
Down	16	2	2	8	128	17	2	1
Total	135	5	4	12	135	54	3	1
*High*								
Up	311	5	15	95	12	166	3	5
Down	97	11	6	14	143	52	2	3
Total	408	16	21	109	155	218	5	8
*Total*	414	27	23	119	213	244	6	9
**2. ** **Liver**								
*Low*								
Up	2	7	16	2	5	2	29	0
Down	3	70	5	3	1	2	27	0
Total	5	77	21	5	6	4	56	0
*Med*								
Up	9	22	122	2	96	3	20	1
Down	1	172	37	2	28	3	37	5
Total	10	194	159	4	124	6	57	6
*High*								
Up	109	51	201	20	111	3	188	6
Down	21	163	83	40	58	1	121	13
Total	130	214	284	60	169	4	309	19
*Total*	139	340	352	69	224	13	334	24
**3. ** **Lung**								
*Low*								
Up	14	22	219	1	7	3	165	6
Down	3	7	37	1	3	3	27	25
Total	17	29	256	2	10	6	192	31
*Med*								
Up	104	152	222	27	61	0	254	41
Down	22	40	131	9	55	1	46	120
Total	126	192	353	36	116	1	300	161
*High*								
Up	240	409	586	51	91	92	415	140
Down	77	214	340	12	108	67	151	132
Total	317	623	926	63	199	159	566	272
*Total*	329	675	1136	84	248	162	624	319

Gene expression profiles for BaP in the forestomach, liver, and lung were previously published* (Labib et al. 2012, 2013)

#### Forestomach gene expression

**Fig. 1:**
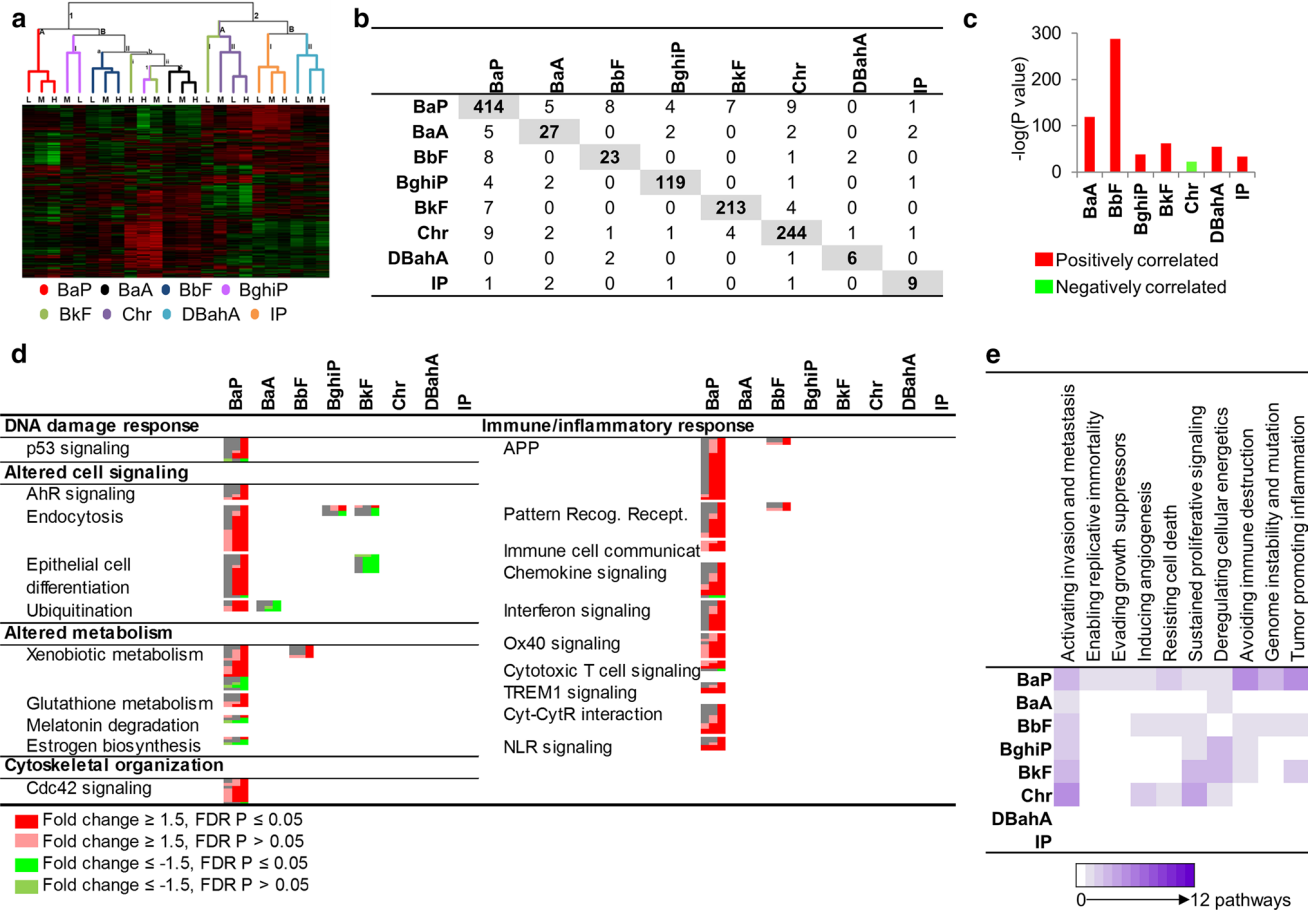
Forestomach **a** hierarchical cluster analysis comparing forestomach gene expression data from adult male Muta™Mouse exposed to three doses (low—L, medium—M, high—H) of eight PAHs. Branches are *color coded* for PAH treatment. Genes were included if they reached significance (FDR *P* ≤ 0.05, fold change ±1.5 in either direction) in at least one treatment group. Groups represent an average of all mice in one group. **b** Table of forestomach DEGs that are common in at least one dose group. Cells highlighted in *gray* represent the total number of DEGs for that PAH. **c** Degree of correlation between BaP DEGs and DEGs for each PAH in the forestomach using the NextBio Meta-analysis tool. The height of each *vertical bar* represents the degree of correlation between the PAH and BaP [−log(*P* value)], which is associated with the directionality of the gene expression changes. *Red color* denotes positive correlation with BaP, and *green color* denotes negative correlation. **d** All pathways significantly enriched for by BaP in the forestomach and the commonalities with other PAHs. Each *column* represents a dose group for the denoted PAH, and each *row* represents a gene. All *colored cells* represent genes with fold change ≥1.5 in either direction. **e** Perturbation of the hallmarks of cancer, emerging hallmarks, and enabling characteristics by each PAH in the forestomach based on transcriptomic data. The *purple cells* represent the relative contribution of pathways associated with each hallmark of cancer (color figure online)

Responses to each individual PAH were analyzed separately, and the results were then compared across all PAHs. Of all the PAHs tested, BaP induced the largest response in forestomach. Hierarchical cluster analysis was conducted using 1008 DEGs that showed differential expression in at least one dose group for at least one PAH (Fig. 1a). The analysis revealed that the samples generally clustered first by PAH (with dose groups together) rather than by dose. BaP clustered along with BbF and BaA (1) on one branch. Chr, DBahA, and IP clustered on a separate branch (2). However, there was no clear clustering observed for BghiP and BkF, which were scattered between the two branches. A VENN analysis of the DEGs from all PAHs revealed that there were no genes in common to all PAHs studied, supporting the results of the cluster analysis. The number of DEGs common between BaP and the other PAHs tested was as follows: 5 for BaA, 8 for BbF, 4 for BghiP, 7 for BkF, 9 for Chr, 0 for DBahA, and 1 for IP (Fig. 1b). Figure 1b also shows the genes in common for each pairwise comparison across all of the PAHs, revealing remarkably little in common.

In addition to comparison of DEGs by VENN analysis, a meta-analysis of all datasets was conducted using NextBio. Since most of the PAHs induced very subtle responses at the low or medium doses, the meta-analysis was conducted on the high-dose groups of each PAH (Fig. 1c). This analysis employed a less stringent statistical model (ANOVA *P* ≤ 0.05) compared to the FDR-adjusted *P* value approach and used a ranking of genes based on their fold changes to explore the correlation of the trends in gene ranking. The results revealed a strong correlation between BaP, BbF, and BaA, whereas BghiP and IP were least correlated with BaP. Chr was negatively correlated with BaP.

##### Similarities in the forestomach response relative to BaP

Figure 1d summarizes the molecular pathways, biological processes, and molecular functions that are altered in forestomach following exposure to BaP as compared to the other seven PAHs. This analysis includes all significantly altered gene ontologies (EASE *P* ≤ 0.05), KEGG pathways (EASE *P* ≤ 0.05), IPA canonical pathways (*P* ≤ 0.05), and MetaCore process networks (*P* ≤ 0.05). Since there was a large redundancy in the annotation of the perturbed biological processes, pathways, and functions, the results were further grouped under parent pathways/processes (Online Resource 1).

Five main functional categories of pathways and processes were perturbed in forestomach following exposure to BaP: DNA damage response, altered cell signaling, immune and inflammatory response, altered metabolism, and cytoskeletal organization (Fig. 1d). Specifically, in the DNA damage response, DEGs were mainly associated with p53 signaling; AhR signaling, endocytosis, epithelial cell differentiation, and ubiquitination were the predominant pathways under altered cell signaling; xenobiotic metabolism, glutathione metabolism, melatonin degradation, and estrogen biosynthesis were related to altered metabolism, and cell division control protein 42 homolog (CDC42) signaling was the most significantly affected under the cytoskeletal organization category. The immune/inflammatory response category was the most enriched, and the DEGs were mainly associated with antigen processing and presentation, pattern recognition receptors, innate to adaptive immune cell communication (immune cell communication), interferon signaling, OX40 signaling, cytotoxic T cell signaling, TREM1 signaling, cytokine–cytokine receptor interaction, and nod-like receptor (NLR) signaling.

To focus on the cancer mode of action, pathway perturbations that functioned in processes related to the published hallmarks of cancer were examined in detail (Hanahan and Weinberg 2011). Specifically, the number and types of pathways associated with the hallmarks of cancer that were perturbed by each PAH were compared to BaP. In the forestomach, BaP exposure perturbed all ten hallmarks of cancer across all doses (Fig. 1e). The number of pathways associated with each hallmark ranged from one to five; the five pathways were associated with the emerging hallmark “avoiding immune destruction” and the enabling characteristic of “tumor-promoting inflammation.”

**Fig. 2:**
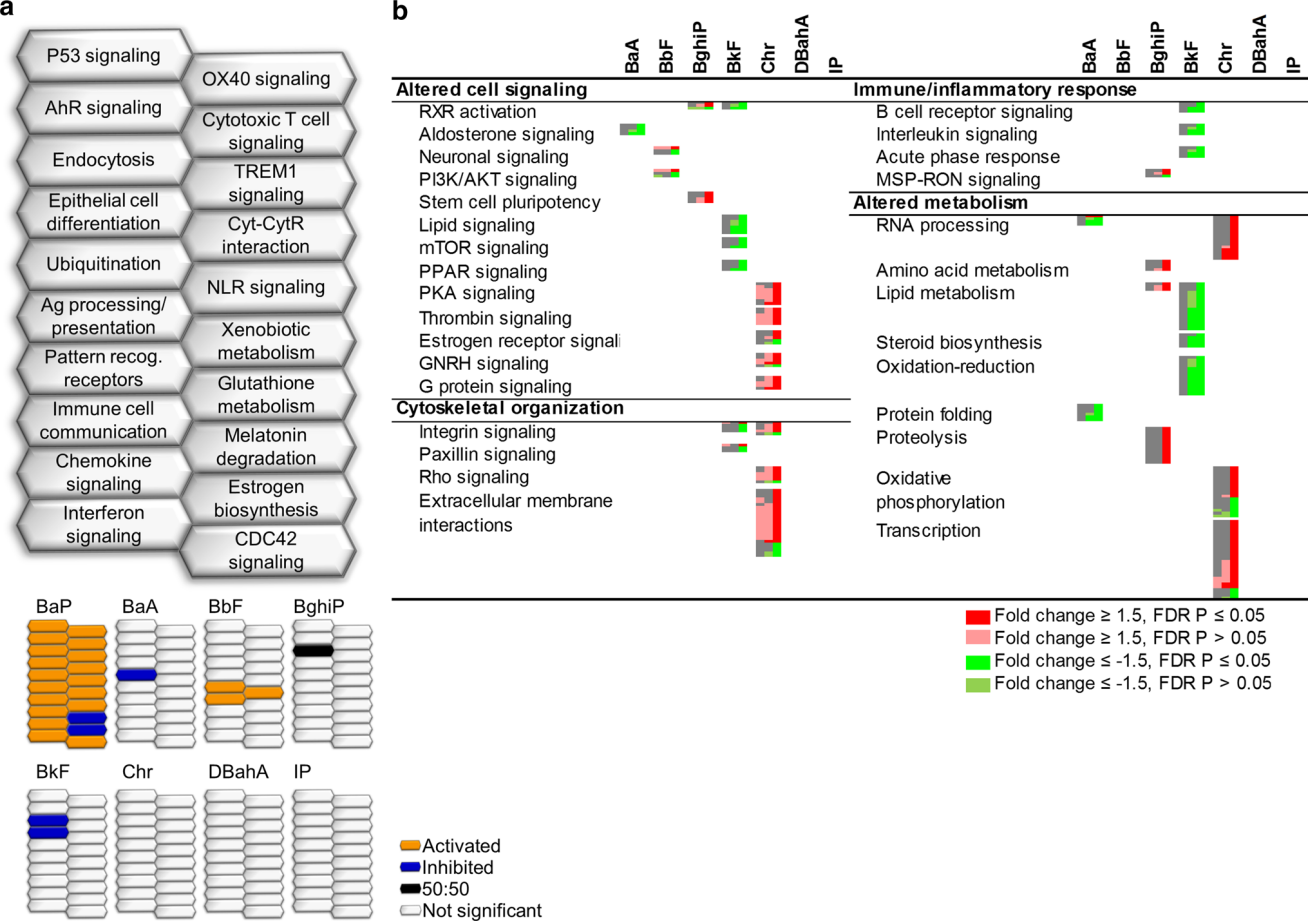
Forestomach **a** honeycomb diagram showing direction of regulation of pathways commonly enriched for by BaP and other PAHs. *Orange honeycombs* represent pathways that are predicted to be activated based on the direction of regulation of the genes, and *blue cells* represent those predicted to be inhibited. *Black cells* represent those in which 50 % of the genes indicate activation and 50 % of the genes indicate inhibition. *White cells* represent pathways not significant. **b** Pathways enriched for by at least one PAH and not by BaP. Each *column* represents a dose group for the denoted PAH and each *row* represents a gene. All *colored cells* represent genes with fold change ≥1.5 in either direction (color figure online)

Of the 20 distinct biological pathways altered in response to BaP, only endocytosis was perturbed by more than one PAH (BghiP and BkF). Other BaP-perturbed pathways such as ubiquitination, antigen processing and presentation (APP), pattern recognition receptors, xenobiotic metabolism, and epithelial cell differentiation were altered following exposure to BaA, BbF, BbF, or BkF, respectively. Chr, DBahA, and IP did not have any pathways in common with BaP. We note that although endocytosis was altered by both BaP and BkF treatment, the direction of change was different; endocytosis appeared to be upregulated following BaP exposure and downregulated following BkF exposure, suggesting that the final toxic outcome of this change may be different for these PAHs (Fig. 2a). Other examples of discrepancies in pathway regulation include epithelial cell differentiation (BaP↑, BkF↓) and protein ubiquitination (BaP↑, BaA↓). It was difficult to determine the directionality of change of endocytosis in BghiP, since 50 % of DEGs in this group suggested upregulation of the pathway and 50 % suggested downregulation. Compared to the BaP-induced response, BaA perturbed only two hallmarks of cancer, with one altered pathway each; BbF perturbed seven hallmarks of cancer with one to two pathways each; Chr perturbed five hallmarks of cancer with one to five pathways each, the strongest response associated with “activating invasion and metastasis”; DBahA and IP did not perturb any hallmarks.

We performed an Upstream Regulator Analysis in IPA to identify upstream biological molecules (transcription factors, membrane receptors, cytokines, etc.) responsible for regulating the expression of DEGs (Online Resource 4) in the forestomach for each of the PAHs. Very few commonalities were observed between BaP and the other PAHs. The results suggest that the transcriptomic changes induced in forestomach by each of the PAHs are mediated by different regulatory molecules.

##### Differences in the forestomach response relative to BaP

Figure 2b shows a range of cellular processes and functions including thrombin signaling, estrogen receptor signaling, transcription, and extracellular membrane interactions that were not altered by BaP, but were altered by exposure to at least one of the other PAHs. Indeed, several pathways (e.g., retinoid X receptor (RXR) activation, lipid metabolism, and integrin signaling) were altered in response to more than one PAH. However, the relevance of these pathways to cancer formation in forestomach is unclear.

#### Hepatic transcriptional response

**Fig. 3:**
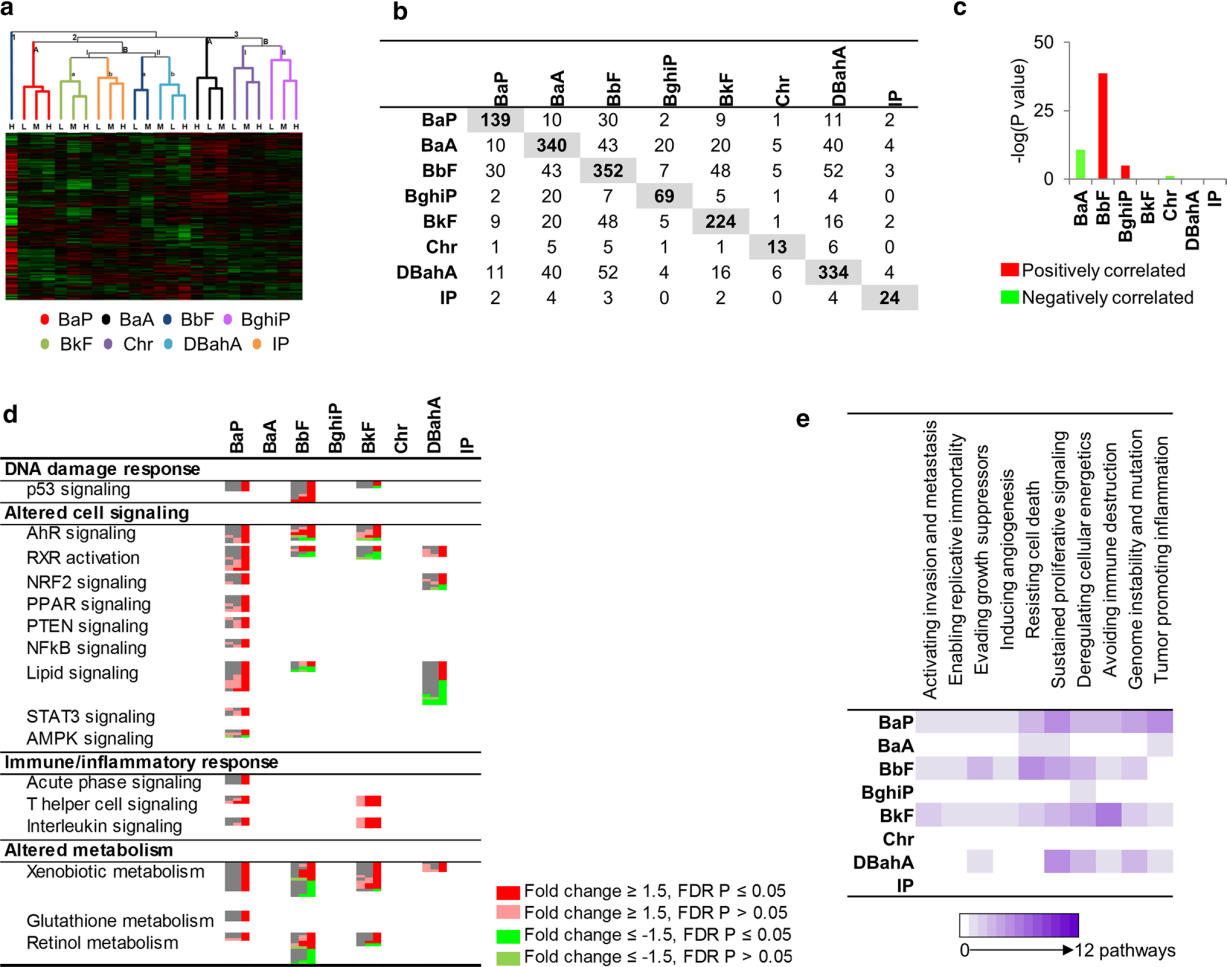
Liver **a** hierarchical cluster analysis comparing hepatic gene expression data from adult male Muta™Mouse exposed to three doses (low—L, medium—M, high—H) of eight PAHs. Branches are *color coded* for PAH treatment. Genes were included if they reached significance (FDR *P* ≤ 0.05, fold change ±1.5 in either direction) in at least one treatment group. Groups represent an average of all mice in one group. **b** Table of hepatic DEGs that are common in at least one dose group. Cells highlighted in *gray* represent the total number of DEGs for that PAH. **c** Degree of correlation between BaP DEGs in the liver and DEGs for each PAH in the liver using the NextBio Meta-analysis tool. The height of each *vertical bar* represents the degree of correlation between the PAH and BaP [−log(*P* value)], which is associated with the directionality of the gene expression changes. *Red color* denotes positive correlation with BaP, and *green color* denotes negative correlation. **d** All pathways significantly enriched for by BaP in the liver and the commonalities with other PAHs. Each *column* represents a dose group for the denoted PAH and each *row* represents a gene. All *colored cells* represent genes with fold change ≥1.5 in either direction. **e** Perturbation of the hallmarks of cancer, emerging hallmarks, and enabling characteristics by each PAH in the liver based on transcriptomic data. The *purple cells* represent the relative contribution of pathways associated with each hallmark of cancer (color figure online)

Of the PAHs tested, BbF, BaA, and DBahA induced the most changes in hepatic gene expression. Hierarchical cluster analysis was conducted using 977 DEGs that were differentially expressed in at least one dose group for at least one PAH. Cluster analysis revealed that each PAH clustered separately, suggesting PAH-specific transcriptomic responses in the liver (Fig. 3a). Except for the high dose of BbF, each PAH clustered along with the other dose groups for that PAH. Three main clusters were observed: cluster 1 consisting of BaP, cluster 2 consisting of BkF, IP, BbF, and DBahA, and cluster 3 consisting of BaA, Chr, and BghiP. Similar to the forestomach results, a VENN analysis of all hepatic DEGs revealed that there were no genes in common to all the PAHs studied. The number of DEGs that were in common between BaP and the other PAHs included: 10 for BaA, 30 for BbF, 2 for BghiP, 9 for BkF, 1 for Chr, 11 for DBahA, and 2 for IP (Fig. 3b). Figure 3b also shows the genes in common for each pairwise comparison across all of the PAHs, revealing little in common.

A meta-analysis of all DEGs from the high-dose group of all the PAHs was conducted in NextBio (Fig. 3c). Transcriptomic profiles of BbF and BaP showed a strong positive correlation, whereas BkF, DBahA, and IP did not correlate with BaP. BaA and Chr were negatively correlated with BaP.

##### Similarities in the hepatic response relative to BaP

BaP-induced hepatic transcriptomic responses were functionally separated into four main categories of pathways and processes: DNA damage response, altered cell signaling, immune and inflammatory response, and altered metabolic signaling. Category 1 (DNA damage response) DEGs were mainly associated with p53 signaling. Category 2 (cell signaling pathways) included DEGs associated with RXR, AHR, nuclear factor-like 2 (NRF2), peroxisome proliferator-activated receptors (PPAR), phosphate and tensin homolog (PTEN), nuclear factor kappa light-activated enhancer of activated B cells (NFkB), lipid, signal transducer and activator of transcription 3 (STAT3), and 5′ adenosine monophosphate-activated protein kinase (AMPK) signaling pathways. Subcategories of cell signaling pathways related to immune/inflammatory response (Category 3) such as acute phase response, T helper cell signaling, and interleukin signaling were also altered. Category 4 (metabolic signaling pathways) included xenobiotic metabolism, glutathione metabolism, and retinol metabolism. In the liver, BaP exposure activated all ten hallmarks of cancer (Fig. 3e). The number of pathways associated with each hallmark ranged from one to five; the five pathways were associated with “sustained proliferative signaling” and the enabling characteristic of “tumor-promoting inflammation”.

**Fig. 4:**
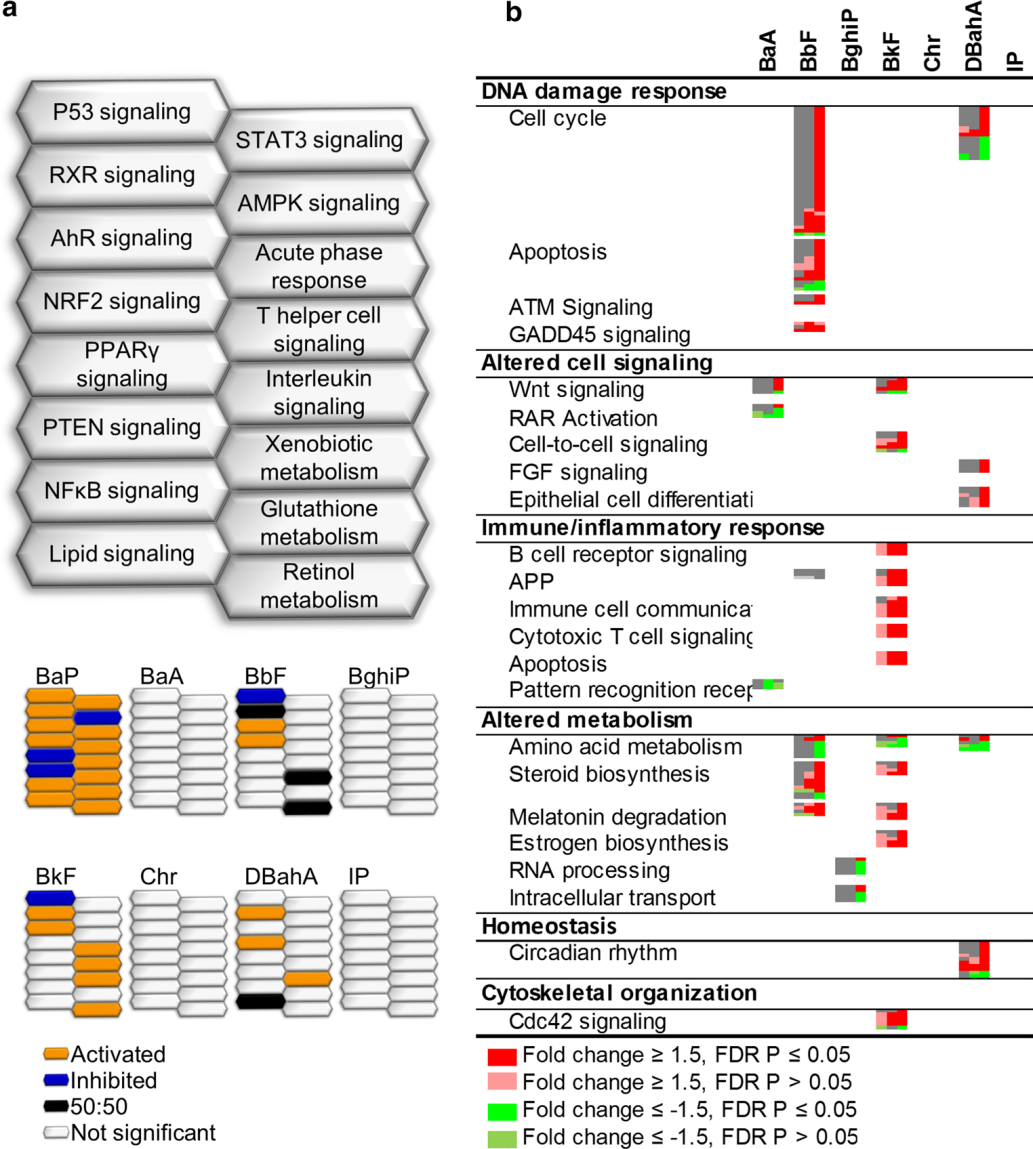
Liver **a** honeycomb diagram showing direction of regulation of pathways commonly enriched for by BaP and other PAHs. *Orange honeycombs* represent pathways that are predicted to be activated based on the direction of regulation of the genes, and *blue cells* represent those predicted to be inhibited. *Black cells* represent those in which 50 % of the genes indicate activation and 50 % of the genes indicate inhibition. *White cells* represent pathways not significant. **b** Pathways enriched for by at least one PAH and not by BaP. Each *column* represents a dose group for the denoted PAH, and each *row* represents a gene. All *colored cells* represent genes with fold change ≥1.5 in either direction (color figure online)

Of the 16 biological pathways altered in response to BaP, RXR signaling and xenobiotic metabolism were commonly perturbed by three other PAHs (BbF, BkF, and DBahA). Pathways common to two other PAHs included p53 signaling, AHR signaling, and retinol metabolism common to BbF and BkF, and lipid signaling common to BbF and DBahA. Although the p53 signaling pathway was altered following treatment with BaP, BbF, and BkF, it was upregulated in response to BaP, whereas it was downregulated in response to BbF and BkF (Fig. 4a). For many other pathways, it was difficult to determine the directionality of change: RXR activation (BaP↑, BbF↕), lipid signaling (BaP↑, DBahA↕), xenobiotic metabolism signaling (BaP↑, BbF↕), and retinol metabolism (BaP↑, BbF↕). Six upstream regulators were identified that were common to three of the PAHs including BaP (Online Resource 4). BbF, BkF, and DBahA each perturbed nine, ten, and six hallmarks of cancer, respectively. The majority of the hallmarks were associated with one pathway; however, “resisting cell death” for BbF was associated with five pathways, “avoiding immune destruction” for BkF associated with six pathways, and “sustained proliferative signaling” for DBahA associated with five pathways. BghiP activated “deregulating cellular energetics” with one pathway, and Chr and IP did not activate any hallmarks of cancer.

##### Differences in the hepatic response relative to BaP

Figure 4b shows a range of cellular processes and functions such as apoptosis, retinoic acid receptor (RAR) signaling, and circadian rhythm that were not perturbed following exposure to BaP, but were for at least one of the other PAHs. A few of these pathways were altered in response to more than one PAH, including cell cycle, wingless integration 1 (WNT) signaling, antigen processing and presentation, amino acid metabolism, steroid biosynthesis, and melatonin degradation. PAH-specific cellular responses were also noted, and they included cell cycle and apoptosis signaling for BbF, immune and inflammatory response (B cell receptor signaling (BCR), antigen processing and presentation, immune cell communication, cytotoxic T cell signaling, immune-mediated apoptosis) for BkF, and circadian rhythm for DBahA.

#### Pulmonary transcriptional response

**Fig. 5:**
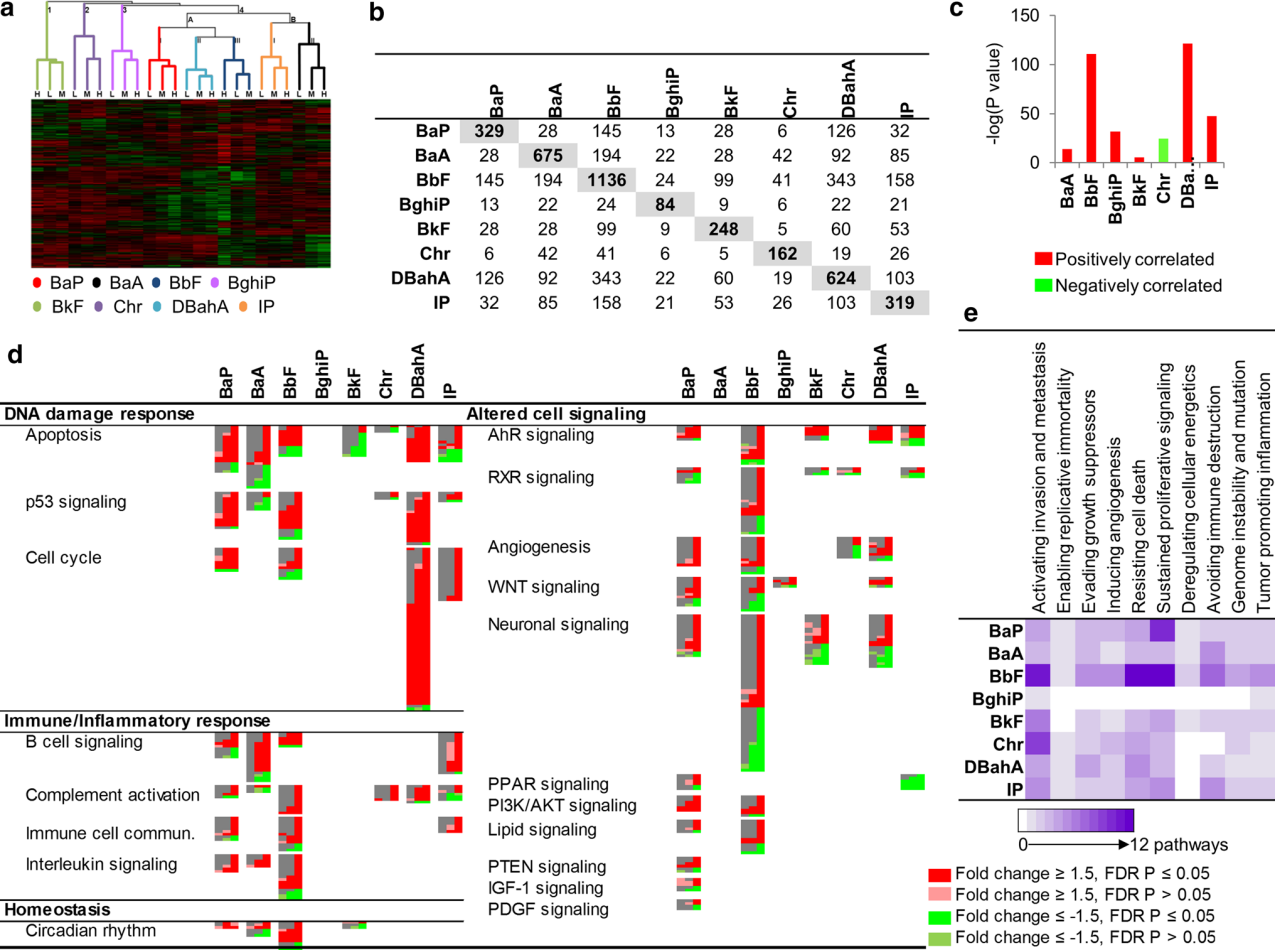
Lung **a** hierarchical cluster analysis comparing pulmonary gene expression data from adult male Muta™Mouse exposed to three doses (low—L, medium—M, high—H) of eight PAHs. Branches are *color coded* for PAH treatment. Genes were included if they reached significance (FDR *P* ≤ 0.05, fold change ±1.5 in either direction) in at least one treatment group. Groups represent an average of all mice in one group. **b** Table of pulmonary DEGs common in at least one dose group. Cells highlighted in *gray* represent the total number of DEGs for that PAH. **c** Degree of correlation between BaP DEGs in the lung and DEGs for each PAH in the lung using the NextBio Meta-analysis tool. The height of each *vertical bar* represents the degree of correlation between the PAH and BaP [−log(*P* value)], which is associated with the directionality of the gene expression changes. *Red color* denotes positive correlation with BaP, and *green color* denotes negative correlation. **d** All pathways significantly enriched for by BaP in the lung and the commonalities with other PAHs. Each *column* represents a dose group for the denoted PAH, and each *row* represents a gene. All *colored cells* represent genes with fold change ≥1.5 in either direction. **e** Perturbation of the hallmarks of cancer, emerging hallmarks, and enabling characteristics by each PAH in the lung based on transcriptomic data. The *purple cells* represent the relative contribution of pathways associated with each hallmark of cancer (color figure online)

A robust transcriptomic response was observed in lungs compared to forestomach and liver for most of the PAHs. Hierarchical cluster analysis was conducted using 2324 DEGs that were differentially expressed in at least one dose group of at least one PAH. Consistent with the observations in the forestomach and liver, transcriptomic patterns were unique in the lungs, exhibiting separate clustering of each PAH along with their dose groups (Fig. 5a). Three main branches were observed: BkF, Chr, and BghiP were clustered on branches 1, 2, and 3, respectively, BaP, DBahA, and BbF on branch 4A, and IP and BaA on 4B. Analysis of all DEGs for commonalities among the PAHs by VENN analysis revealed no genes in common to all PAHs studied. The number of DEGs in common between BaP and the other PAHs was 28 for BaA, 145 for BbF, 13 for BghiP, 28 for BkF, 6 for Chr, 126 for DBahA, and 32 for IP (Fig. 5b). NextBio meta-analysis of all of the DEGs from the high-dose groups of each PAH revealed that DBahA and BbF were the most correlated with the transcriptomic changes induced by BaP, followed by BkF and BaA. Consistent with the observations in forestomach and liver, Chr was negatively correlated with BaP (green bar) (Fig. 5c).

##### Similarities in the pulmonary response relative to BaP

Although BaP induced a robust pulmonary transcriptomic response, the main categories of pathways and processes perturbed in the lungs were the same as those in forestomach and livers, with the exception of alterations in homeostasis (Fig. 5d). However, subcategories of pathways and processes varied. For example, for DNA damage response, BaP-induced DEGs were associated with apoptosis, cell cycle, and p53 signaling. Altered cell signaling was enriched and included several subcategories of pathways and processes: angiogenesis, neuronal signaling, lipid signaling, as well as WNT, PPAR, RXR, PTEN, phosphoinositide-3-kinase protein kinase B (PI3K/AKT), insulin-like growth factor 1 (IGF-1), AHR, and platelet-derived growth factor (PDGF) signaling pathways. The most significantly altered immune and inflammatory pathways were B cell receptor signaling, complement activation, innate-to-adaptive immune cell communication, and interleukin signaling. Circadian rhythm was the only pathway perturbed under the category “homeostasis.” In the lungs, BaP exposure activated pathways associated with all ten hallmarks of cancer (Fig. 5e). The number of pathways associated with each hallmark ranged from one to ten pathways; the ten pathways were associated with “sustained proliferative signaling”.

**Fig. 6:**
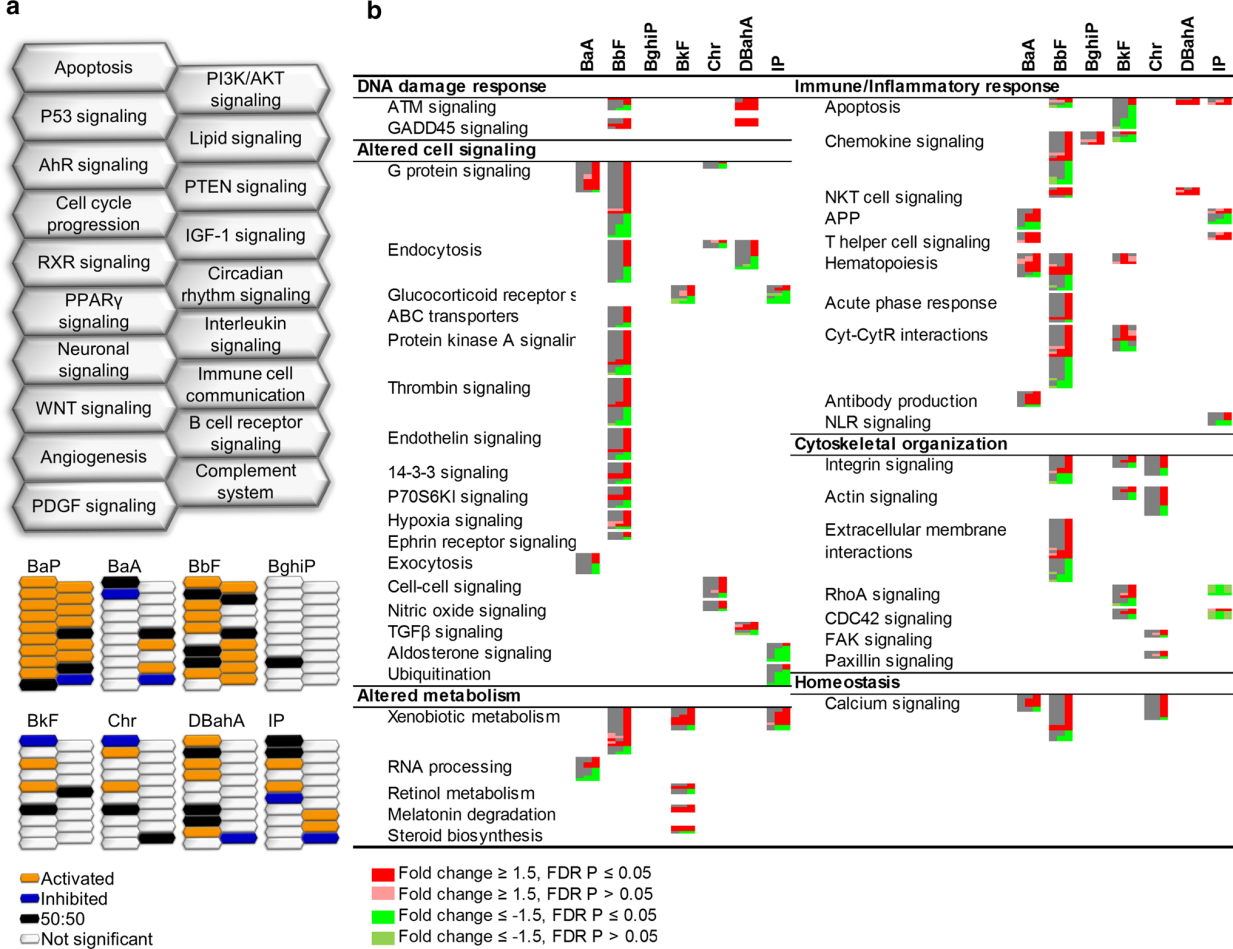
Lung **a** honeycomb diagram showing direction of regulation of pathways commonly enriched for by BaP and other PAHs. *Orange honeycombs* represent pathways that are predicted to be activated based on the direction of regulation of the genes, and *blue cells* represent those predicted to be inhibited. *Black cells* represent those in which 50 % of the genes indicate activation and 50 % of the genes indicate inhibition. *White cells* represent pathways not significant. **b** Pathways enriched for by at least one PAH and not by BaP. Each *column* represents a dose group for the denoted PAH, and each *row* represents a gene. All *colored cells* represent genes with fold change ≥1.5 in either direction (color figure online)

Although 16 pathways or biological processes were perturbed by BaP and at least one other PAH, the collective pathway response induced by the other PAHs varied significantly, which is clearly shown in Fig. 6a. For example, apoptosis, cell cycle, and p53 signaling pathways reflecting DNA damage response were also induced by BbF, DBahA, and IP. Of the 11 distinct pathways in the “altered cell signaling” category perturbed in response to BaP, only seven were significant following BbF exposure, and less than four were altered in response to the other PAHs. Similarly, only BbF, BaA, and IP showed significant changes in at least three of the four pathways associated with immune and inflammatory response. In addition, among the commonly regulated pathways, the directionality of change in pathways reflecting their functionality was different for each PAH. A few examples of this include p53 signaling (BaP↑, BaA↓), apoptosis (BaP↑, BkF↓, Chr↓), and complement system activation (BaP↓, BbF↑). For several others, it was difficult to determine directionality: apoptosis (BaP↑, BaA↕, IP↕), p53 signaling (BaP↑, BbF↕, DBahA↕, IP↕), angiogenesis (BaP↑, Chr↕), Wnt signaling (BaP↑, BbF↕, BghiP↕, DbahA↕), neuronal signaling (BaP↑, BbF↕, BkF↕, DBahA↕), lipid signaling (BaP↑, BbF↕), and complement activation (BaP↑, Chr↕). The other PAHs each affected pathways associated with at least eight hallmarks of cancer, with the exception of BghiP that induced two with one pathway each. BbF perturbed the greatest magnitude (number of pathways) of response with two hallmarks associated with 12 significant pathways, “resisting cell death” and “sustained proliferative signaling” and one hallmark associated with eleven significant pathways, “activating invasion and metastasis.”

Fourteen upstream regulators were associated with the genes altered in response to three of the PAHs and BaP in lungs (Online Resource 4), including endogenous mammalian chemicals, protein complexes and groups, enzymes, growth factors, ligand-dependent nuclear receptors, and transcription regulators. All of these regulators (except β-estradiol) were connected by a single node—p53—a transcription factor commonly implicated in PAH-induced carcinogenesis.

##### Differences in the pulmonary response relative to BaP

Antibody production, circadian rhythm, calcium signaling, xenobiotic metabolism, G protein signaling, and cytoskeletal organization were among the pathways altered following exposure to PAHs that were not perturbed by BaP exposure (Fig. 6b). Although these differences were not found for BaP, pathways such as cytoskeletal organization, immune-mediated apoptosis, circadian rhythm, and calcium signaling were perturbed in response to more than three PAHs, potentially suggesting a non-BaP-like response shared by other PAHs. Some of these pathways were unique to a single PAH, including: exocytosis signaling, RNA processing, and antibody production for BaA; altered cell signaling (ABC transporters, protein kinase A signaling, thrombin signaling, endothelin signaling, 14-3-3 signaling, inhibitor of P70 S6 kinase (P70S6KI) signaling, hypoxia signaling, ephrin receptor signaling), acute phase response, extracellular membrane interactions, and inflammatory response for BbF; altered metabolism (retinol metabolism, melatonin degradation, steroid biosynthesis) for BkF; altered cell signaling (cell-to-cell signaling, nitric oxide signaling) and cytoskeletal reorganization (focal adhesion kinase (FAK) signaling, paxillin signaling) for Chr; transforming growth factor β (TGFβ) signaling for DBahA; and altered cell signaling (aldosterone signaling, ubiquitination) and NLR signaling for IP.

### Apical responses to PAHs in the lungs

Since the lung transcriptome was the most affected of the three tissues studied, in the present study, we evaluated DNA adduct formation, *lacZ* mutant frequency, and EROD activity for lungs. The purpose is to exclusively compare the PAH-specific responses for these apical endpoints within the most affected tissue.

#### Pulmonary DNA adduct formation

**Fig. 7:**
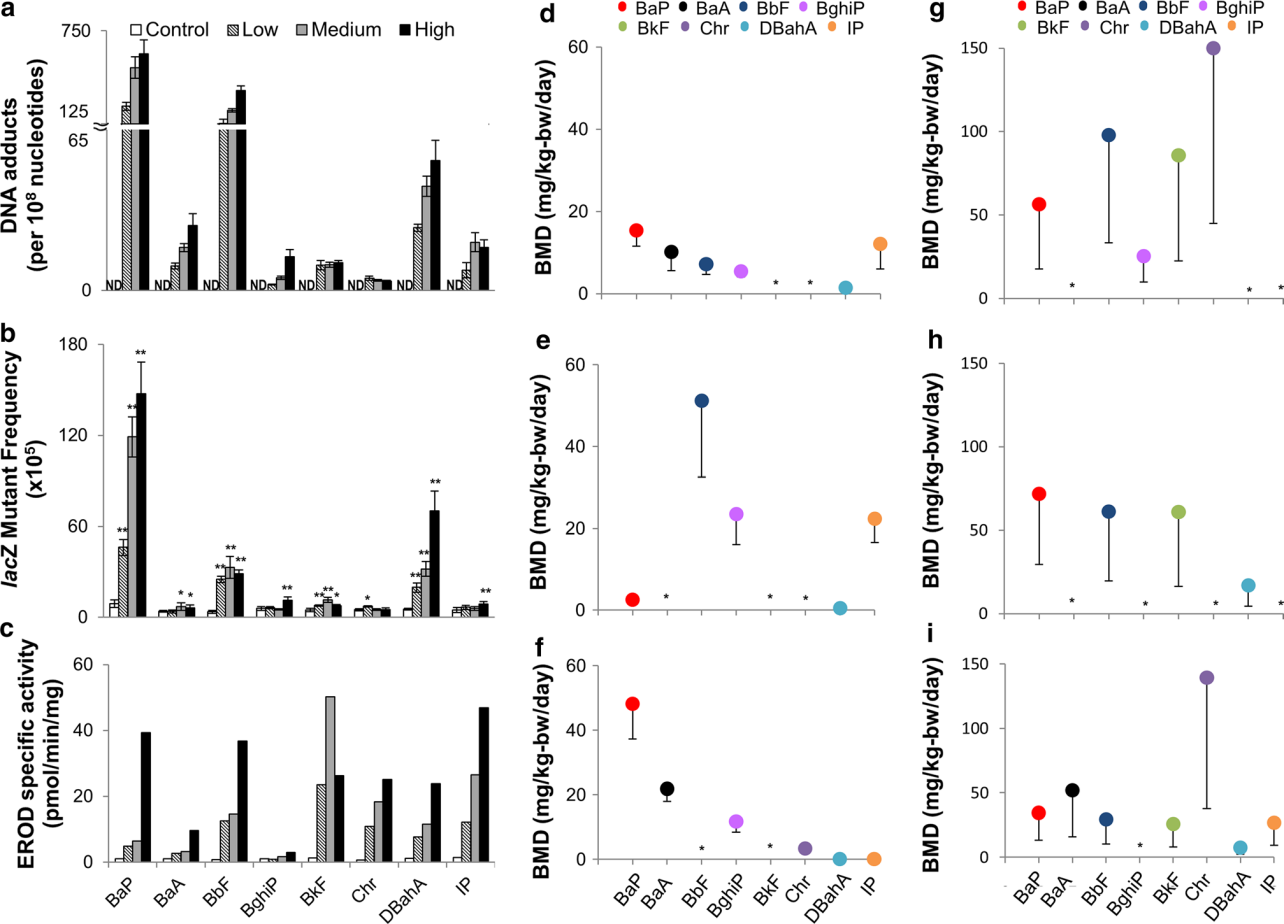
**a** DNA adduct formation, **b** *lac*Z mutant frequency, and **c** EROD activity in the lungs from Muta™Mouse sub-chronically exposed to BaP, BaA, BbF, BghiP, BkF, Chr, DBahA, and IP. Levels of PAH-DNA adducts were determined using the nuclease P1 enrichment version of the ^32^P-post-labeling method. Data are represented as average ± SEM. Average *lac*Z mutant frequency was determined using the P-gal-positive selection assay. Values shown are average frequencies × 10^5^ ± SEM. *P < 0.1, **P < 0.01 compared to vehicle controls. EROD activity was determined by pooling the tissue for all mice in each dose group. No significance can be calculated. DNA adduct and lacZ mutant frequency for BaP were previously reported (Labib et al. 2012). *ND* not detected. **d**–**i** Charts of BMD (−BMDL) values in mg/kg-bw/day for each PAH based on data for **d** DNA adducts, **e** *lac*Z mutant frequency, and **f** EROD activity, and the number of hallmarks of cancer associated with the pathways in the **g** forestomach, **h** liver, and **i** lung. *Asterisk* indicates the data could not be modeled

The presence of bulky DNA adducts indicates the potential mutagenicity of exposure to a chemical. Using the ^32^P-post-labeling technique, DNA adducts were measured in mouse lungs following exposure to each of the individual PAHs (Fig. 7a). No adducts were detected in controls treated with vehicle only. The DNA adduct profiles obtained are shown in Online Resource 5. Although every PAH tested induced adducts in all dose groups, the adduct levels for all of these PAHs were lower than BaP. BaA, BbF, BghiP, and DBahA showed a dose-dependent increase in adduct levels similar to BaP; however, the levels of DNA adducts formed following exposures to BkF, Chr, and IP did not show dose-specific trends. It is important to note that the DNA adduct spots for the BghiP and IP samples were close to the origin on the thin-layer chromatography plate, which may have impacted quantitation. Thus, the observed increases in the adduct levels for these two PAHs are classified as semiquantitative (Online Resource 5).

#### Pulmonary *lacZ* mutant frequency

A dose-dependent increase in pulmonary *lacZ* mutant frequency compared to vehicle controls was observed for BaP (Labib et al. 2012) and DBahA (Fig. 7b). Significant increases in *lacZ* mutant frequencies were observed in at least one dose group of BbF and BkF (ANOVA: *P* ≤ 0.0001), and BghiP and IP (ANOVA: *P* ≤ 0.01). *LacZ* mutant frequency was marginally increased for BaA and Chr despite the presence of DNA adducts (ANOVA: *P* ≤ 0.1).

#### EROD enzymatic activity analysis

We measured enzymatic activity of CYP1A1 via the EROD assay in the lungs of mice exposed to all of the PAHs (Fig. 7c). A dose-dependent increase in the activity of CYP1A1 was observed for BaP, BaA, BbF, BghiP, Chr, DBahA, and IP. BkF also showed an increase in EROD activity, but there was no apparent dose–response. BbF showed the maximum activity at the high dose with a 43.8-fold increase compared to vehicle controls. BghiP was the lowest inducer at all doses and showed maximum activity at the high dose with 2.8-fold increase compared to vehicle controls.

#### BMD analysis

The relative potency of the PAHs was calculated using BMD analysis. BMD and BMDL values for each apical endpoint in the lung tissue (DNA adduct formation, *lacZ* mutant frequency, and EROD activity) and for the number of hallmarks of cancer activated by each PAH in the forestomach, liver, and lung were obtained. BMD values derived from gene expression data were compared to BMDs of the apical endpoints (Fig. 7d–i). In general, the BMD models were able to fit the dose–response relationships exhibited by the apical endpoints and the hallmarks of cancer for the eight PAHs studied. Visual inspection of the curves confirmed this, and BMD/BMDL ratios <4 were obtained for all datasets.

The BMD values derived from the pulmonary DNA adduct data ranged from 1.5 mg/kg-bw/day for DBahA to 15.4 mg/kg-bw/day for BaP. The BkF and Chr data could not be modeled. In order of lowest to highest BMD values: DBahA < BghiP < BbF < BaA < IP < BaP; however, the differences between BMD and BMDL (lower confidence intervals) for each PAH suggest that the potencies are not statistically significant, with the exception of DBahA, which is substantially lower. The BMD values derived from the *lac*Z mutant frequency data ranged from 0.5 mg/kg-bw/day for DBahA to 23.5 mg/kg-bw/day for BghiP, compared to 2.6 mg/kg-bw/day for BaP. The BaA, BbF, BkF, and Chr data could not be modeled. In order of lowest to highest BMD values: DBahA < BaP < IP < BghiP; however, the BghiP and IP lower confidence intervals overlap. The BMD values derived from the pulmonary EROD data ranged from 0.06 mg/kg-bw/day for DBahA to 48.2 mg/kg-bw/day for BaP. The BbF and BkF data could not be modeled. In order of lowest to highest BMD values: DBahA < IP < Chr < BghiP < BaA < BaP. Note that the lower confidence intervals for DBahA and IP overlap.

The BMDL values derived from the hallmarks of cancer in the forestomach tissue ranged from 25.3 mg/kg-bw/day for BghiP to 150.0 mg/kg-bw/day for Chr, compared to 56.5 mg/kg-bw/day for BaP. The forestomach data for BaA, DBahA, and IP could not be modeled because of lack of response at the transcriptional level. In order of lowest to highest, the BMDL values were BghiP < BaP < BkF < BbF < Chr; however, the lower confidence intervals overlap for all PAHs. The BMD values derived from the hallmarks of cancer in the liver ranged from 17.0 mg/kg-bw/day for DBahA to 71.9 mg/kg-bw/day for BaP. The data for BaA and BghiP could not be modeled due to lack of model fit, and the data for Chr and IP could not be modeled due to lack of response. In order of lowest to highest, the BMD values were DBahA < BkF = BbF < BaP; however, the lower confidence intervals for all PAHs except for DBahA overlap. The BMD values derived from the hallmarks of cancer in the lung ranged from 7.53 mg/kg-bw/day for DBahA to 139.2 mg/kg-bw/day for Chr, compared to 34.5 mg/kg-bw/day for BaP. In order of lowest to highest, the BMD values were DBahA < BkF < IP < BbF < BaP < BaA < Chr. Note that the lower confidence intervals for all PAHs overlap except for DBahA. The data for BghiP could not be modeled due to lack of fit.

## Discussion

BaP is commonly accepted as a reference PAH in the estimation of excess lifetime cancer risk posed by genotoxic PAHs or PAH-containing mixtures (CCME 2010; EFSA 2008; Health Canada 2010). This is primarily based on the hypothesis that the genotoxic PAHs, such as those studied herein, act via similar modes/mechanisms of action. However, the results from several recent studies have challenged this paradigm (Jung et al. 2013; Song et al. 2012; Tilton et al. 2015). In the present study, we tested this hypothesis using a toxicogenomic approach that enables investigation of global changes in the expression of genes and associated pathways in a tissue in a single experiment. We specifically characterized the gene expression changes occurring in mouse forestomach, liver, and lung following exposure to seven individual PAHs (CCME 2010) and compared the expression changes in these tissues to those induced by exposure to BaP in previous studies (Labib et al. 2012, 2013; Malik et al. 2013). Systematic bioinformatics analysis of all DEGs and pathways perturbed in response to each individual PAH revealed three major features: (1) all PAHs induce expression changes in genes associated with a wide variety of carcinogenic pathways, processes, and functions, (2) all PAHs induce changes in the expression of genes implicated in DNA damage response conventionally described for genotoxic PAHs; however, the response varies significantly from PAH to PAH in the number of DEGs and the magnitude of the response (level of expression of each gene), and (3) PAHs induce transcriptional responses that are PAH- and tissue-specific. The significant differences noted in the global pathway perturbations across PAHs imply that, in contrast to the assumptions made in mixture risk assessment, the underlying toxicity pathways, some of which have been implicated in carcinogenesis, are not the same for all of the PAHs investigated and vary across tissues.

**Fig. 8:**
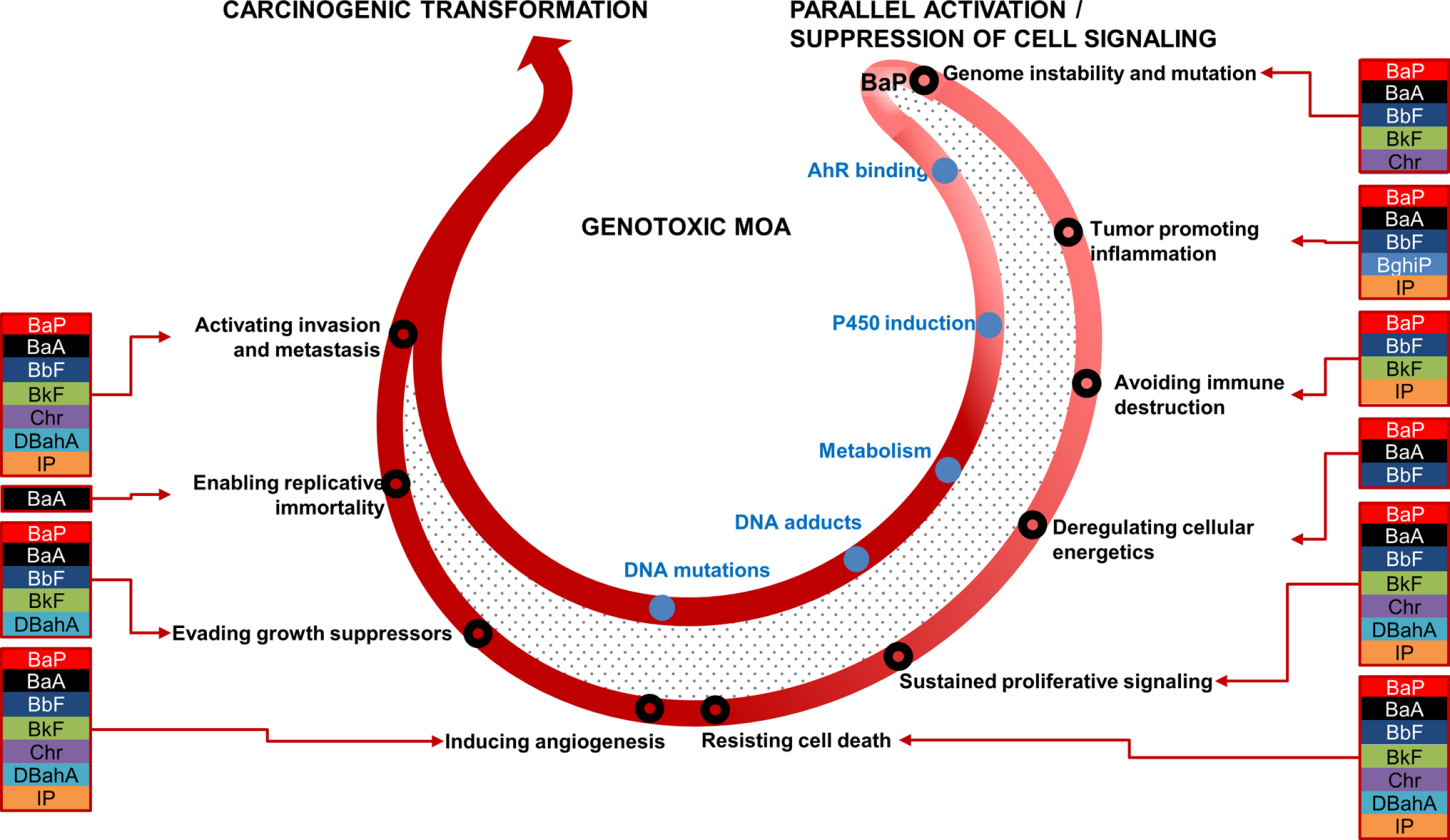
Activation or suppression of cell signaling pathways in support of pulmonary carcinogenic transformation. This schematic describes the contribution of each PAH to the carcinogenic mode of action in the form of the hallmarks of cancer. These signaling events are vital to the progression of cancer and are occurring in parallel to the genotoxic MOA key events. The order of the hallmarks in the figure does not reflect any temporal coordination or cooperation with the existing key events. BaP’s carcinogenic MOA key events are shown in *blue font* (color figure online)

The accepted ways by which carcinogenic PAHs induce DNA damage and mutations involve: (1) induction of the dihydrodiol epoxide pathway catalyzed by CYP enzymes leading to stable pro-mutagenic DNA adducts, (2) the oxidation of PAH dihydrodiols by dihydrogenases to PAH-derived ortho-quinones leading to stable DNA adducts and formation of reactive oxygen species that may further contribute to the DNA damage, and (3) binding and activation of AhR resulting in intensified transcriptional activation of not only CYP1A1 but also sulfotransferases and other conjugation enzymes, which also influence DNA adduct formation. The formation of DNA adducts, an immediate consequence of PAH metabolism, is considered an early key event of PAH-mediated carcinogenesis (Moffat et al. 2015). However, several studies have shown that DNA adducts may not accurately predict the carcinogenic risk of genotoxic PAHs (Pottenger et al. 2009). For example, it has been shown that DNA adducts are present in most of the tissues of rats (Kroese et al. 2001) and mice (Zuo et al. 2014) exposed to BaP, including the tissues that eventually develop cancer, as well as those that do not. Similar observations of lack of tumors in tissues showed that high levels of DNA adducts following exposure to BaP were also noted in mice (Culp et al. 1998) and in rats (Weyand et al. 1995). Furthermore, tissue-specific rates of CYP induction, levels of DNA adducts, and mutation frequencies do not correlate with the extent of tumor formation in the tissues studied (Godschalk et al. 2003). These findings suggest that additional factors may be involved in PAH-induced tissue-specific oncogenesis. While Kroese et al. (2001) suggested that local cellular proliferation may be one such additional factor required for cancer development, here we show that perturbations of several additional biological pathways that are directly favorable to carcinogenesis may be operating in parallel with DNA damage/mutation to influence the probability of developing tissue-specific tumors in mice (Fig. 8).

In the present study, exposure to each of the PAHs caused significant increases in both DNA adducts and transgene mutant frequency in lung compared to matched controls. However, considerable differences in lung responses were observed at the pathway level for each PAH examined. PAH-specific pathway changes were also observed in the liver and forestomach. To determine whether consideration of apical endpoints and perturbations in a number of key pathways that are favorable to cancer formation would help improve cancer predictivity, we compared the BMDs for the lung apical endpoints and genomics endpoints for each of the PAHs examined. The known hallmarks of cancer were used as a directional tool to effectively classify a wide variety of pathways perturbed by the PAHs into functional groups and to highlight the altered tissue microenvironment following exposure. BMD calculation for individual apical endpoints and cancer-specific pathways varied depending on the type of data considered. The DNA adduct-derived BMDs predicted BghiP to be the second most potent, which is contrary to the available literature and its recent classification as a PAH lacking evidence for carcinogenicity by the USEPA and IARC (IARC 2010; USEPA 2011). These results further support the complexities related to the predictive value of DNA adducts for carcinogenic potency of PAHs discussed above. BMD values derived from the results of mutant frequency ranked DBahA and BaP as the most potent with respect to their ability to induce mutation in a non-transcribed transgene. BaA and Chr data could not be modeled due to their poor ability to induce mutation as observed by the lack of response. It was also not possible to model BkF data due to lack of dose response. However, at the low and medium dose, BkF did show increased mutant frequency. Similarly, cancer pathway-based BMDs ranked DBahA as the most potent PAH able to elicit hallmark events and Chr as the least potent, which is in line with IARC’s classification of Chr as a group 3 non-carcinogen (IARC 2010; USEPA 2011). BaP was consistently ranked less potent than DBahA for all endpoints considered, in line with the results of an earlier study that showed DBahA as a more potent carcinogen in rats compared to BaP (Wenzel-Hartung et al. 1990). The importance of the temporal coordination of the hallmarks of cancer should be noted. The activation of angiogenesis or invasion and metastasis are later events in tumor progression; however, identification of perturbations in related signaling pathways at early stages may enhance the tumor predictivity when considered in conjunction with conventional toxicological endpoints. Although, it is not general practice to derive BMDs for DNA adducts, mutations (e.g., *lac*Z mutant frequency), or gene pathways for regulatory purposes, in the absence of rodent cancer bioassay data, BMDs for these toxicological endpoints may serve to set regulatory limits that would protect against effects that are mechanistically or empirically linked to carcinogenesis and prioritize compounds for further testing by long-term cancer bioassays. Recently, in a comprehensive case study, Moffat et al. (2015) compared BMDs derived from traditional cancer bioassays and those derived from pathway-based analyses indicative of carcinogenesis in BaP-exposed tissues. The authors showed a high concordance between the points of departures derived from both apical and gene expression endpoints, as well as cancer bioassays for BaP. However, it is not possible to make direct comparisons between the BMDs derived from cancer bioassays and the results from the present study since not all the PAHs examined have cancer bioassay results available (IARC 2010). Although further studies are needed to conclusively demonstrate the use of such non-cancer bioassay data for regulatory purposes, in the immediate term, the gene expression results obtained from the present and the study by Moffat et al. can be used to support the decision to use non-cancer data in a regulatory setting and to help reduce the uncertainties associated with that approach. Collectively, these results suggest that depending on the endpoint investigated, the potency ranking of each of these PAHs, including BaP, will differ for each tissue; a detailed knowledge of pathway perturbations will reduce the uncertainties involved in making such predictions of future carcinogenesis and will increase the accuracy of these rankings.

With the exception of BghiP, we observed that the key pathways involved in DNA adduct formation and DNA damage in lungs were all activated in response to the individual PAH exposures. These results suggested that six of the PAHs tested induce DNA damage responses similar to those observed in response to BaP. However, activation of several other cellular signaling pathways indicative of hallmarks of cancer, including neuronal signaling (required for activation of invasion and metastasis), activation of angiogenesis (required for blood vessel formation in tumors), activation of PI3 K/AKT signaling pathway (involved in resisting cell death), cell cycle progression (required for cellular proliferation in the presence of damaged DNA), circadian rhythm signaling (associated with the evasion of growth suppressors), inhibition of PPARγ signaling (required for inhibition of neoplastic growth of cancer cells), avoiding immune destruction, and tumor-promoting inflammation, varied between the PAHs. The other pathways uniquely altered only in response to congener PAHs in the present study, such as calcium signaling (BaA, BbF, and Chr), endocytosis (BbF, Chr, and DBahA), chemokine signaling (BbF, BghiP, and BkF), and cytoskeletal reorganization (BbF, BkF, Chr, and IP), have previously been linked to BaP exposure (Ba et al. 2014; Laupeze et al. 2002; Mayati et al. 2012; van Grevenynghe et al. 2003). For example, G protein-coupled receptor signaling mediates alterations in calcium signaling in endothelial HMEC-1 cells and human kidney HEK293 cells following exposure to BaP (Mayati et al. 2012), as demonstrated by the inhibition of BaP-induced signaling in the presence of suramin, a potent inhibitor of G protein. Chemokine signaling induced by BbF, BghiP, and BkF was observed following BaP-induced inflammation in primary human macrophages (Lecureur et al. 2005; Sparfel et al. 2010). Similarly, endocytic function is inhibited in macrophages, and dendritic cells derived from human monocytes exposed to BaP (Laupeze et al. 2002; van Grevenynghe et al. 2003). However, the implications of these pathways in cancer formation in lungs are not clear. Thus, it is not apparent whether a combination of DNA adduct formation, mutation induction, and the perturbation of pathways mentioned above is sufficient to trigger cancer formation following exposure to PAHs. One possibility is that the PAHs that induced low levels of DNA damage or mutations and subtle responses at the pathway levels could be acting as tumor promoters rather than initiators of cancer. For instance, it is known that altered calcium signaling helps sustain cellular proliferation and foster metastatic environment (Prevarskaya et al. 2011; Resende et al. 2013), and defective endocytosis has been linked to increased metastatic function in cancer cells (Mosesson et al. 2008). Cytoskeletal organization has been linked to metastasis and invasion of early tumor cells (Donald et al. 2001), and it is known that BaP exposure promotes cell migration and invasion in both in vivo and in vitro models of human hepatocellular carcinoma (Ba et al. 2014). This may be required for expansion of genetically damaged cells and subsequent cancer formation. Inflammation involving chemokine signaling is also proposed to promote conditions conducive to cancer formation in genotoxic, as well as in non-genotoxic, scenarios (Lu et al. 2006). Thus, in addition to the DNA damage response pathways induced by BaP and the other PAHs examined, activation of distinct signaling pathways induced by the other PAHs suggests greater contribution of alternative carcinogenic mode/mechanisms.

In addition to the induction of cancer-related pathways, the results of the present study demonstrate that the tissue-specific transcriptional responses to PAHs are distinct, suggesting that the carcinogenic potency of each individual PAH will vary based on the type of tissue investigated. Of the forestomach, liver, and lung tissues, the greatest response at the transcriptional level was induced in the lung, which we previously showed for BaP in both acute exposure (B6C3F1 mice orally exposed to BaP for 3 days with sample collection 4 h post-exposure) (Halappanavar et al. 2011) and sub-chronic studies (Labib et al. 2012, 2013). Indeed, the BMD values for transcriptional pathways associated with the hallmarks of cancer for seven of the eight PAHs indicated that the pulmonary response was more sensitive than the liver and forestomach (BaP, BaA, BbF, BkF, Chr, DBahA, and IP). For BghiP, only the forestomach data could be modeled, possibly indicative of lower potential for adverse outcomes in the lung and liver compared to the forestomach for this PAH. Similar observations of enhanced pulmonary response were noted in mice exposed to oral BaP in studies conducted by Halappanavar et al. (2011) and Harrigan et al. (2006). Other recent studies have also shown tissue-specific gene expression changes in a multi-tissue analysis following BaP exposure (Zuo et al. 2014). One of the explanations for the observed tissue-specific discrepancies could be the different rates of metabolism, clearance, and translocation or tissue distribution of these PAHs from the targeted tissues. It is a possibility that some PAHs, such as DBahA and BbF, are quickly distributed to distant organs (e.g., lungs) from the forestomach (site of contact), which may result in reduced impact on the forestomach. This in turn may induce differential responses at the molecular level that are related to the likelihood of eventual cancer formation.

## Conclusions

The present study explored the validity of the assumption that BaP is an acceptable reference PAH for genotoxic, carcinogenic PAHs by examining the global gene expression changes in three separate tissues. Our results showed that all of the PAHs investigated were able to induce genotoxicity in a dose-dependent manner. However, these results were not comparable with the known relative potencies calculated based on BaP as a reference chemical (CCME 2010). The results also revealed a response at the transcriptional level following exposure to the PAHs that varied according to agent, dose, and tissue. Moreover, the gene expression results clearly demonstrated that each PAH induces molecular alterations (signaling pathways) related to carcinogenesis that are distinct from those observed for BaP, thus contradicting the assumption that they all act through similar mechanisms. We show differences in the ability of PAHs to induce major key events, as well as other molecular changes related to carcinogenesis, which suggests that the relative potency approach, which is estimated based on current default assumptions, may not be accurate. These distinct tissue-specific transcriptional responses are indicative of the need to exercise caution when deriving potency equivalence factor (PEF) values; depending on what endpoint/tissue is evaluated in coming up with a PEF value, these differences could lead to overestimation or underestimation of the PEF, which would result in overestimation or underestimation of the total risk estimate.

Thus, the results of the present study show that BaP alone may not accurately reflect the toxicity imparted by other PAH congeners and suggest that consideration of toxicity induced by more than one PAH be used to represent the class of PAHs. Finally, moving forward, an integrated approach that involves multiple endpoints, including genomics, and multiple tissues may help increase the accuracy of the assay-derived relative potencies for each PAH and the predictive potential of the relative potency approach.

## Electronic supplementary material

Below is the link to the electronic supplementary material.

Supplementary material 1 (PDF 250 kb)

Supplementary material 2 (PDF 259 kb)

Supplementary material 3 (PDF 26570 kb)

Supplementary material 4 (PDF 254 kb)

Supplementary material 5 (PDF 274 kb)
