# Supplementary material for: Comparative transcriptomic analyses to scrutinize the assumption that genotoxic PAHs exert effects via a common mode of action
Source: Arch Toxicol. 2015 Sep 16;90(10):2461–80. doi: 10.1007/s00204-015-1595-5 (PMC5043007; doi:10.1007/s00204-015-1595-5)
Supplement: Supplementary file 1 — Supplementary material 1 (PDF 250 kb) [file 204_2015_1595_MOESM1_ESM.pdf]

Article Title: Comparative Transcriptomic Analyses to Scrutinise the Assumption That Genotoxic Priority PAHs Exert Effects via a Common Mode of Action

Journal: Archives in Toxicology

Authors: Labib S, Williams A, Guo CH, Leingartner K, Arlt VM, Schmeiser HH, Yauk CL, White PA, Halappanavar S*

Corresponding Author: *Sabina Halappanavar, Health Canada, Sabina.halappanavar@hc-sc.gc.ca

Supplementary File 1 - Detailed methodology

A - Tissue RNA extraction and purification

Total RNA was isolated for gene expression analysis as described previously (Halappanavar et al. 2011; Labib et al. 2013; Labib et al. 2012). Briefly, total RNA was extracted from a random slice of the forestomach, liver, and lung using TRIzol reagent (Invitrogen, Carlsbad, CA, USA) and purified using RNeasy Mini Kit (Qiagen, Mississauga, ON, Canada) following manufacturer's recommendations. All samples showed high quality RNA: with a A260/A280 ratios between 2.0 and 2.2 as determined by the NanoDrop Spectrophotometer (ThermoFisher Scientific, Mississauga, ON, Canada) and RNA integrity numbers above 7 as determined using an Agilent 2100 Bioanalyzer (Agilent Technologies Inc., Mississauga, ON, Canada).

B - Microarray hybridization and analysis

Detailed protocols of the microarray hybridization and statistical analysis of the gene expression data are described in Labib et al. (2013). A minimum of four individual animals were used from each treatment group from each PAH except for BkF low dose group, which had only 3 mice. Sample sizes of 5 were used for each PAH and treatment group, except for the BkF and IP exposures (Table 1).In brief, 200 ng of total RNA from each individual sample and 200 ng of Universal Mouse Reference RNA (Stratagene, Mississauga, ON, Canada) was used to synthesize cDNA and cyanine-labeled cRNA using the Agilent Linear Amplification Kit (Agilent Technologies Inc., Mississauga, ON, Canada). The labeled cRNA (Cyanine-5 for experimental samples and Cyanine-3 for reference RNA) was purified using RNeasy Mini Kit (Qiagen, Mississauga, ON, Canada). 300 ng of labeled cRNA from each experimental sample was hybridized with the same amount of labeled reference RNA to Agilent Sureprint G3 Mouse GE 8x60K microarrays (Agilent Technologies Inc., Mississauga, ON, Canada) at 65°C for 17 hours in the Agilent SureHyb hybridization chamber. The arrays were washed and scanned on the Agilent G2505B Scanner according to the manufacturer's recommendations. Data were extracted using Feature Extraction 10.7.3.1 (Agilent Technologies Inc., Mississauga, ON, Canada).

Details of the statistical normalization methods of the gene expression data are described in Labib et al. (2013). Briefly, a reference design (Kerr and Churchill 2001; Kerr and Churchill 2007) was used to analyse microarray data. Non-background median signal intensities were normalized using LOWESS (Yang et al. 2002) using the R (R Development Core Team 2010) software. A gene was considered differentially expressed using the Fs statistic (Cui et al. 2005) in the MAANOVA R library (Wu et al. 2003). The P values for all statistical tests for each probe ID were estimated by the permutation method using residual shuffling, followed by adjustment for multiple comparisons using the false discovery rate (FDR) approach (Benjamini and Hochberg 1995). The fold change calculations were based on the least-square means (Goodnight and Harvey 1978; Searle et al. 1980). All microarray results are

deposited in the Gene Expression Omnibus database (http://www.ncbi.nlm.nih.gov/geo/) under the accession number GSE51321.

C - Hierarchical clustering analysis

Biological replicates of each experimental condition were collapsed to an average expression value for each gene and were normalized to the median of the control samples. The expression data relative to controls for all samples treated with the eight PAHs were then merged together using the Agilent probe IDs. This dataset was then filtered using the differentially expressed genes (DEGs) from the eight independent MAANOVA analyses. Hierarchical clustering was then applied to the filtered data using the one minus correlation dissimilarity metric using the spearman correlation with average linkage. Data was visualized using a heatmap.

D - Bioinformatics and pathway analysis

List of DEGs (FDR $P \leq 0.05$, fold change ± 1.5) from each PAH congener were independently analysed to identify biological functions or processes perturbed in response to the treatment. DAVID (Huang da et al. 2009) Functional Annotation Charts were used to identify gene ontology (GO) terms (biological processes and cellular compartments) associated with the significant genes, and to classify the DEGs into biological pathways using KEGG pathways (Kanehisa and Goto 2000). MetaCore (Thomson Reuters, http://www.genego.com/metacore.php) pathway analysis and network process analysis were used for functional classification. Ingenuity Pathway Analysis (IPA, Ingenuity Systems, Redwood City, CA, USA) Canonical Pathway analysis, Biological Function analysis, and Network analysis were used to identify biological pathways and functions associated with the DEGs. Based on the tool used, different criteria were used to determine the significance of pathways, functions or biological processes perturbed. Any pathway or process that was associated with more than 3 DEGs was included in the interpretation of the results. In addition, an EASE score cut-off of $P \leq 0.05$ was applied to DAVID ontologies and KEGG pathways, and $P \leq 0.05$ to IPA canonical pathways and MetaCore process networks. Redundancy in pathways and processes were reduced by collapsing multiple pathways or processes implying perturbation of the same biological function (Supplementary File 2).

IPA's upstream regulator analysis was performed to determine the common regulatory mechanisms (transcription factors and receptors) operating in response to individual PAHs compared to BaP. This analysis constructs a network of all possible relationships between upstream regulators and the genes in the data. The activation Z-score makes predictions about the activation state of the regulators (activated or inhibited) by using information about the direction of gene regulation. All upstream regulators with ≥ 3 genes, $P \leq 0.05$, and activation Z-score ≥ |2.0| were included in the interpretation. Upstream regulators categorized as chemical drugs, chemical toxicants, biologic drugs, chemical kinase inhibitor, chemical protease inhibitor, chemical endogenous (non-mammalian), chemical reagent, and chemical toxicant were not included in the analysis.

The NextBio meta-analysis function was used to compare the transcriptomic responses following exposure to individual PAHs and following exposure to BaP. A less stringent non-FDR p-value cut-off of less than 0.05 and fold change of ± 1.2 was applied. A pairwise comparison was made for each gene between the two datasets (for example, BaP vs BbF). A rank-based enrichment statistic was applied to determine the final correlation score. If the direction of change in the expression of a gene following a PAH is the same as the direction of change in expression of that gene following exposure to BaP, then the correlation is positive.

Since the focus of the study was to compare the carcinogenic potential of PAHs, significantly perturbed pathways and processes were reorganized according to their association with the 6 hallmarks of cancer (activating invasion and metastasis, enabling replicative immortality, evading growth suppressors, inducing angiogenesis, resisting cell death, sustained proliferative signaling), 2 emerging hallmarks (deregulating cellular energetics, avoiding immune destruction), and 2 enabling characteristics (genome instability and mutation, tumor promoting inflammation), as described in Hanahan and Weinberg (2011). The pathways or processes that could not be classified as cancer related were not used in the analysis and interpretation of the results.

E - DNA extraction from lung tissue

The frozen lung tissues were sliced randomly for isolation of genomic DNA as described in Labib et al. (2012). In brief, lung tissue was minced and degassed to remove all traces of air in the alveoli and washed in ice cold Phosphate Buffered Saline (PBS) twice. The minced tissue was lysed in 10 mM Tris, pH 7.6, 10 µM EDTA, 100 µM NaCl, and 1% SDS and digested with proteinase K (1 mg/ml) overnight on a rotating platform at 37°C. Genomic DNA was isolated on the following day using a phenol/chloroform/isoamyl alcohol (25:24:1) and chloroform/isoamyl alcohol (24:1) extraction procedure (Renault et al. 1997). The DNA was precipitated in ethanol and dissolved in TE buffer and stored at 4^{0}C until used.

F - *Lac*Z mutant frequency in lung tissue

The *lac*Z mutant frequency analysis in lung tissues using the phenyl-β-D-galactopyranoside (P-Gal) positive selection assay was conducted as described previously (Labib et al. 2012; Lemieux et al. 2011). Briefly, the λgt10*lac*Z DNA was removed from the mouse genomic DNA and packaged using the Transpack lambda packaging system (Stratagene, La Jolla, CA, USA). The packaged DNA was mixed with bacteria (*Escherichia coli lacZ*$^{-}$, *gal*E^{-}, *rec*A^{-}, pAA19 with *gal*T and *gal*K), plated on minimal medium (0.3% (w/v) P-Gal), and incubated overnight at 37°C. At the same time, total plaque forming units were measured on titers that did not contain P-Gal. Mutant frequency is expressed as a ratio of the number of mutant plaque forming units to total plaque forming units.

G - DNA adduct analysis in lung tissue

DNA adduct formation in each sample was determined using the nuclease P1 digestion enrichment version of ^{32}P-post-labeling assay as described previously (Phillips and Arlt 2014; Phillips and Arlt 2007) with minor modifications as described in Labib et al. (2012). Briefly, 4 µg of total DNA was digested with micrococcal nuclease (288 mUnits, Sigma) and calf spleen phosphodiesterase (1.2 mUnits, MP Biomedicals), enriched, and was radiolabelled (Phillips and Arlt 2014; Phillips and Arlt 2007). The labeled adducted nucleotide biphosphates were separated by thin-layer chromatography (TLC) on polyethyleneimine-cellulose plates (Macherey-Nagel, Düren, Germany) with chromatographic conditions described in (Arlt et al. 2008). Chromatographs were scanned using a Packard Instant Imager (Canberra Packard, Downers Grove, USA) and DNA adduct levels (relative adduct labeling) were calculated from the adduct counts per minute (cpm), the specific activity of [γ-^{32}]PATP, and the amount of DNA (pmol of DNA-P) used. Results are expressed as DNA adducts per 10^{8} nucleotides.

H - Enzymatic analysis of lung Cyp1a activity

For the enzymatic analysis, since the available amount of tissue from each treatment group was small, samples from the same treatment group were pooled (Supplementary File 1). Approximately 30 mg of lung tissue from each individual sample from specific treatment group were pooled and homogenized using a serrated Teflon pestle and glass grinding vessel in 2.5 volumes of ice cold 0.05M Tris-1.15% KCl

buffer pH 7.4. The homogenate was centrifuged at 10,200 g for 20 minutes at 4°C (Sorval Legend Micro 21R) and the supernatant (S9 fraction) was separated for protein and EROD analyses. EROD activity, a measure of CYP1A1 and CYP1A2 enzyme activity, was determined according to the method of Burke et al. (1985), with modifications. Reactions were carried out at 37°C with triplicate technical replicates in 96-well plates. The final incubation consisted of Tris buffer (0.05 M, pH 7.4), 0.5 µM 7-ethoxyresorufin substrate, 15 µM dicumerol, 11 mM $MgCl_2$, 20 µl of lung S9 supernatant, and 280 µM NADPH (Sigma Aldrich Canada Ltd, Oakville, ON, Canada), with a final well volume of 220 µl. The fluorescence of resorufin was measured every minute for 8 minutes with a SpectraMax M2 (Molecular Devices, Sunnyvale, CA, USA), with excitation and emission wavelengths of 530 nm and 585 nm. Fluorescence values were converted to nanomoles using standard resorufin fluorescence. Total protein content of the S9 fraction was measured using the Quick Start™ Bradford Protein Assay (Bio-Rad Laboratories, Hercules, CA, USA), using a standard curve of bovine serum albumin.

References

Arlt VM, Stiborová M, Henderson CJ, et al. (2008) Metabolic activation of benzo[a]pyrene in vitro by hepatic cytochrome P450 contrasts with detoxification in vivo: experiments with hepatic cytochrome P450 reductase null mice. Carcinogenesis 29(3):656-665

Benjamini Y, Hochberg Y (1995) Controlling the false discovery rate: A practical and powerful approach to multiple testing. Journal of the Royal Statistical Society: Series B (Methodological) 57:289-300

Cui X, Hwang JT, Qiu J, Blades NJ, Churchill GA (2005) Improved statistical tests for differential gene expression by shrinking variance components estimates. Biostatistics 6(1):59-75

Goodnight JH, Harvey WR (1978) Least-Square Means in the Fixed-Effects General Linear Models., vol Technical Report R-103. SAS Institute Inc, Cary, N.C.

Halappanavar S, Wu D, Williams A, et al. (2011) Pulmonary gene and microRNA expression changes in mice exposed to benzo(a)pyrene by oral gavage. Toxicology 285:133-141

Hanahan D, Weinberg R (2011) Hallmarks of cancer: the next generation. Cell 144(5):646-674

Huang da W, Sherman BT, Lempicki RA (2009) Systematic and integrative analysis of large gene lists using DAVID bioinformatics resources. Nature Protocols 4(1):44-57

Kanehisa M, Goto S (2000) KEGG: kyoto encyclopedia of genes and genomes. Nucleic Acids Research 28(1):27-30

Kerr MK, Churchill GA (2001) Experimental design for gene expression microarrays. Biostatistics 2(2):183-201

Kerr MK, Churchill GA (2007) Statistical design and the analysis of gene expression microarray data. Genetical Research 89(5-6):509-514

Labib S, Guo C, Williams A, Yauk C, White P, Halappanavar S (2013) Toxicogenomic outcomes predictive of forestomach carcinogenesis following exposure to benzo(a)pyrene: relevance to human cancer risk. Toxicology and Applied Pharmacology 273(1):269-280

Labib S, Yauk C, Williams A, et al. (2012) Sub-chronic oral exposure to benzo(a)pyrene leads to distinct transcriptomic changes in the lungs that are related to carcinogenesis. Toxicological Sciences 129(1):213-224

Lemieux C, Douglas G, Gingerich J, et al. (2011) Simultaneous measurement of benzo[a]pyrene-induced Pig-a and lacZ mutations, micronuclei and DNA adducts in Muta™ Mouse. Environmental and Molecular Mutagenesis 52(9):756-765

Phillips D, Arlt V (2014) 32P-Postlabeling Analysis of DNA Adducts. In: Keohavong P, Grant SG (eds) Molecular Toxicology Protocols. Methods in Molecular Biology, vol 1105. Humana Press, p 127-138

Phillips DH, Arlt VM (2007) The 32P-postlabeling assay for DNA adducts. Nature Protocols 2(11):2772-2781

R Development Core Team (2010) R: A language and environment for statistical computing. R Foundation for Statistical Computing, Vienna, Austria

Renault D, Brault D, Thybaud V (1997) Effect of ethylnitrosourea and methyl methanesulfonate on mutation frequency in MutaTMMouse germ cells seminiferous tubule cells and epididymis spermatozoa. Mutation Research 388:145-153

Searle SR, Speed FM, Milliken GA (1980) Population marginal means in the linear model: An alternative to least squares means. The Journal of American Statistics 34:216-221

Wu H, Kerr MK, Cui X, Churchill GA (2003) MAANOVA: A Software Package for the Analysis of Spotted cDNA Microarray Experiments. In: Parmigiani G, Garrett ES, Irizarry RA, Zeger S (eds) The analysis of gene expression data: methods and software. Springer, Verlag, p 313-431

Yang YH, Dudoit S, Luu P, et al. (2002) Normalization for cDNA microarray data: A robust composite method addressing single and multiple slide systematic variation. Nucleic Acids Research 30(4):e15
