# Supplementary material for: Comparative transcriptomic analyses to scrutinize the assumption that genotoxic PAHs exert effects via a common mode of action
Source: Arch Toxicol. 2015 Sep 16;90(10):2461–80. doi: 10.1007/s00204-015-1595-5 (PMC5043007; doi:10.1007/s00204-015-1595-5)
Supplement: Supplementary file 2 — Supplementary material 2 (PDF 259 kb) [file 204_2015_1595_MOESM2_ESM.pdf]

Article Title: Comparative Transcriptomic Analyses to Scrutinise the Assumption That Genotoxic Priority PAHs Exert Effects via a Common Mode of Action
Journal: Archives in Toxicology
Authors: Labib S, Williams A, Guo CH, Leingartner K, Arlt VM, Schmeiser HH, Yauk CL, White PA, Halappanavar S*
Corresponding Author: *Sabina Halappanavar, Health Canada, sabina.halappanavar@hc-sc.gc.ca

Supplementary File 2 - Collapsed functional groupings for each pathway and process

	Gene Ontologies	KEGG pathways	IPA Canonical Pathways	MetaCore: Process Networks
**DNA damage response**				
Apoptosis	GO:0006915 apoptosis		Apoptosis Signaling	Apoptosis: Apoptotic nucleus
	GO:0006917 induction of apoptosis			Apoptosis: Anti-Apoptosis mediated by external signals via PI3K/AKT
	GO:0043065 positive regulation of apoptosis			Apoptosis: Apoptotic mitochondria
	GO:0008629 induction of apoptosis by intracellular signals			Apoptosis: Endoplasmic reticulum stress pathway
	GO:0001844 protein insertion into mitochondrial membrane during induction of apoptosis			
	GO:0042981 regulation of apoptosis			
	GO:0043066 negative regulation of apoptosis			
	GO:0006916 anti-apoptosis			
ATM signaling			ATM signaling	
Cell cycle	GO:0007049 cell cycle	mmu04110:Cell cycle	Cell Cycle: G1/S Checkpoint Regulation	Cell cycle: Core
	GO:0007050 cell cycle arrest		Cell Cycle: G2/M DNA Damage Checkpoint Regulation	Cell cycle: G1-S
	GO:0000087 M phase of mitotic cell cycle		Cyclins and Cell Cycle Regulation	Cell cycle: G2-M
	GO:0007067 mitosis		Cell Cycle Control of Chromosomal Replication	

	GO:0022402 cell cycle process		Role of CHK Proteins in Cell Cycle Checkpoint Control	Cell cycle: Mitosis
	GO:0051329 interphase of mitotic cell cycle		Role of BRCA1 in DNA Damage Response	Cell cycle: S phase
	GO:0000278 mitotic cell cycle		Mitotic Roles of Polo-Like Kinase	Cell cycle: G0-G1
	GO:0000075 cell cycle checkpoint		Estrogen-mediated S-phase entry	Cell cycle: G1-S Growth factor regulation
	GO:0000082 G1/S transition of mitotic cell cycle			Cell cycle: G1-S Interleukin regulation
	GO:0000910 cytokinesis			DNA damage: Checkpoint
	GO:0007093 mitotic cell cycle checkpoint			
	GO:0007346 regulation of mitotic cell cycle			
	GO:0051325 interphase			
	GO:0051726 regulation of cell cycle			
GADD45 signaling			GADD45 signaling	
p53 signaling pathway		mmu04115:p53 signaling pathway	p53 Signaling	
**Altered cell signaling**				
14-3-3 signaling			14-3-3-mediated Signaling	
ABC transporter signaling		mmu02010:ABC transporters		
AhR signaling			Aryl Hydrocarbon Receptor Signaling	
Aldosterone signaling			Aldosterone Signaling in Epithelial Cells	
AMPK signaling			AMPK Signaling	
Angiogenesis	GO:0001525 angiogenesis			Development: Regulation of angiogenesis
	GO:0045765 regulation of angiogenesis			Cardiac development: Wnt_beta-catenin, Notch, VEGF, IP3 and integrin signaling
	GO:0045766 positive regulation of angiogenesis			

	GO:0016525 negative regulation of angiogenesis	
Cell-cell signaling		Epithelial Adherens Junction Signaling<br>Gap Junction Signaling<br>Tight Junction Signaling
Endocytosis	GO:0006897~endocytosis	Virus Entry via Endocytic Pathways<br>Caveolar-mediated Endocytosis Signaling<br>Fcγ Receptor-mediated Phagocytosis in Macrophages and Monocytes<br>Macropinocytosis Signaling
Endothelin signaling		Endothelin-1 Signaling
Ephrin receptor signaling	GO:0048013~ephrin receptor signaling pathway	
Estrogen receptor signaling		Estrogen receptor signaling
Exocytosis	GO:0006887~exocytosis	
FGF signaling		FGF Signaling
G protein signaling		G-Protein Coupled Receptor Signaling<br>cAMP-mediated signaling<br>Dopamine-DARPP32 Feedback in cAMP Signaling<br>Gαi Signaling<br>Gαq Signaling<br>Cholecystokinin/Gastrin-mediated Signaling
Glucocorticoid receptor signaling		Glucocorticoid Receptor Signaling
GNRH signaling		GNRH Signaling
Hypoxia signaling		HIF1α Signaling
IGF-1 signaling		IGF-1 Signaling

Lipid signaling		Sphingosine-1-phosphate signaling<br>Cholecystokinin/Gastrin-mediated signaling<br>Docosahexaenoic Acid (DHA) Signaling<br>Eicosanoid Signaling
NF-κB Signaling		NF-κB Signaling
Nitric oxide signaling		nNOS Signaling in Skeletal Muscle Cells<br>Nitric Oxide Signaling in the Cardiovascular System
MTOR signaling		mTOR Signaling
Neuronal signaling	mmu04360:Axon guidance<br><br>mmu04080:Neuroactive ligand-receptor interaction	Axonal Guidance Signaling<br><br>Neuropathic Pain Signaling In Dorsal Horn Neurons<br><br>Synaptic Long Term Potentiation<br>CDK5 Signaling<br>Netrin Signaling<br>GABA Receptor Signaling
NRF2 signaling		NRF2-mediated Oxidative Stress Response
p70S6K Signaling		p70S6K Signaling
PDGF signaling		PDGF Signaling
PI3K/Akt signaling		PI3K/AKT Signaling
PPAR signaling	mmu03320:PPAR signaling pathway	PPAR Signaling
PKA signaling		Protein Kinase A Signaling
PTEN Signaling		PTEN Signaling
RAR signaling		RAR Activation
STAT3 signaling		STAT3 Pathway
Stem cell pluripotency		Human Embryonic Stem Cell Pluripotency
RXR signaling		LXR/RXR Activation<br>VDR/RXR Activation<br>PPARα/RXRα Activation

			PXR/RXR Activation<br>FXR/RXR Activation	
TGF-β Signaling			TGF-β Signaling	
TREM1 signaling			TREM1 Signaling	
Ubiquitination			Protein Ubiquitination Pathway	
Wnt signaling	GO:0016055 Wnt receptor signaling pathway<br>GO:0030111 regulation of Wnt receptor signaling pathway<br>GO:0030178~negative regulation of Wnt receptor signaling pathway	mmu04340:Hedgehog signaling pathway	Wnt/β-catenin Signaling<br><br>Sonic Hedgehog Signaling	
**Immune/inflammatory response**				
Antibody production		mmu04672:Intestinal immune network for IgA production		
Antigen processing and presentation	GO:0002474 antigen processing and presentation of peptide antigen via MHC class I<br>GO:0002475 antigen processing and presentation via MHC class Ib<br><br>GO:0002478 antigen processing and presentation of exogenous peptide antigen<br>GO:0002495 antigen processing and presentation of peptide antigen via MHC class II<br>GO:0002504 antigen processing and presentation of peptide or polysaccharide antigen via MHC class II	mmu04612:Antigen processing and presentation	Antigen Presentation Pathway	Immune response: Phagosome in antigen presentation<br><br>Immune response: Antigen presentation

	GO:0019882 antigen processing and presentation<br><br>GO:0019884 antigen processing and presentation of exogenous antigen<br><br>GO:0019886 antigen processing and presentation of exogenous peptide antigen via MHC class II<br><br>GO:0048002 antigen processing and presentation of peptide antigen			
B cell receptor signaling	GO:0019724 B cell mediated immunity<br>GO:0030183 B cell differentiation<br>GO:0030888 regulation of B cell proliferation<br>GO:0030890 positive regulation of B cell proliferation<br>GO:0042113 B cell activation<br>GO:0050853 B cell receptor signaling pathway<br><br>GO:0050864 regulation of B cell activation<br>GO:0050871 positive regulation of B cell activation	mmu04662:B cell receptor signaling pathway	B Cell Development<br><br>B Cell Activating Factor Signaling<br>B cell receptor signaling	Immune response: BCR pathway
Chemokine signaling	GO:0006935 chemotaxis	mmu04062:Chemokine signaling pathway	Chemokine Signaling	Chemotaxis<br><br>Cell adhesion: Leucocyte chemotaxis

Complement system signaling	GO:0006956 complement activation<br>GO:0006958 complement activation, classical pathway	mmu04610:Complement and coagulation cascades	Complement System	Inflammation: Complement system
Cytokine-cytokine receptor signaling	GO:0006954 inflammatory response<br><br>GO:0034097 response to cytokine stimulus<br>GO:0019221 cytokine-mediated signaling pathway	mmu04060:Cytokine-cytokine receptor interaction		
Cytotoxic T cell signaling			CTLA4 Signaling in Cytotoxic T Lymphocytes<br>Cytotoxic T Lymphocyte-mediated Apoptosis of Target Cells	
Hematopoiesis		mmu04640:Hematopoietic cell lineage		
Immune cell communication			Communication between innate and adaptive immune cells<br>Crosstalk between Dendritic Cells and Natural Killer Cells<br><br>Dendritic Cell Maturation	
Immune-mediated apoptosis		mmu04650:Natural killer cell mediated cytotoxicity	Granzyme A Signaling<br><br>Calcium-induced T Lymphocyte Apoptosis<br>Nur77 Signaling in T Lymphocytes	
Interferon signaling			Interferon Signaling	Inflammation: Interferon signaling<br>Inflammation: IFN-gamma signaling
Interleukin signaling			IL-4 Signaling	Inflammation: IL-10 anti-inflammatory response

		Role of IL-17A in Arthritis<br><br>IL-8 Signaling<br><br>IL-17A Signaling in Gastric Cells<br>IL-6 Signaling	Inflammation: IL-6 signaling<br><br>Inflammation: IL-4 signaling<br><br>Inflammation: IL-13 signaling pathway
MSP-RON signaling		MSP-RON Signaling Pathway	
Natural killer T cell signaling		Natural Killer Cell Signaling<br><br>Tumoricidal Function of Hepatic Natural Killer Cells<br><br>Crosstalk between Dendritic Cells and Natural Killer Cells	Inflammation: NK cell cytotoxicity
Nod-like receptor signaling	mmu04621:NOD-like receptor signaling pathway		
OX40 signaling		OX40 Signaling Pathway	
Pattern recognition receptors		Role of Pattern Recognition Receptors in Recognition of Bacteria and Viruses<br><br>Activation of IRF by Cytosolic Pattern Recognition Receptors	
T helper cell signaling		T Helper Cell Differentiation<br><br>CD28 Signaling in T Helper Cells<br>iCOS-iCOSL Signaling in T Helper Cells	
**Cytoskeletal organization**			
Actin signaling		Actin Cytoskeleton Signaling	
CDC42 signaling		Cdc42 Signaling	
Extracellular membrane interactions	mmu04510:Focal adhesion		

		mmu04512:ECM-receptor interaction		
FAK signaling			FAK Signaling	
Integrin signaling			Integrin Signaling	Cell adhesion: Integrin-mediated cell-matrix adhesion
Paxillin signaling			Paxillin Signaling	
RHO signaling			RhoA Signaling<br>Signaling by Rho Family GTPases<br>Regulation of Actin-based Motility by Rho<br>RhoGDI Signaling	
Thrombin signaling			Extrinsinc pro-thrombin activation pathway<br>Thrombin signaling	
**Altered metabolism**				
Amino acid metabolism	GO:0009063~cellular amino acid catabolic process<br><br>GO:0009066~aspartate family amino acid metabolic process	mmu00270:Cysteine and methionine metabolism<br><br>mmu00260:Glycine, serine and threonine metabolism<br><br>mmu00280:Valine, leucine and isoleucine degradation		
Estrogen biosynthesis			Estrogen biosynthesis	
Glutathione metabolism		mmu00480:Glutathione metabolism	Glutathione-mediated Detoxification	
Intracellular transport	GO:0046907 intracellular transport<br>GO:0006890 retrograde vesicle-mediated transport, Golgi to ER<br>GO:0090004 positive regulation of establishment of protein localization to plasma membrane			

	GO:0090003 regulation of establishment of protein localization to plasma membrane<br>GO:0016192~vesicle-mediated transport		
Lipid metabolism	GO:0006633 fatty acid biosynthetic process<br>GO:0006631~fatty acid metabolic process<br>GO:0006643 membrane lipid metabolic process<br>GO:0006644 phospholipid metabolic process<br>GO:0006665 sphingolipid metabolic process<br>GO:0006695~cholesterol biosynthetic process<br>GO:0006869 lipid transport<br><br>GO:0008610 lipid biosynthetic process<br>GO:0010876 lipid localization<br>GO:0016042 lipid catabolic process<br>GO:0030149 sphingolipid catabolic process<br>GO:0044242 cellular lipid catabolic process<br>GO:0046466 membrane lipid catabolic process<br>GO:0046486 glycerolipid metabolic process	mmu00600:Sphingolipid metabolism<br>mmu00561:Glycerolipid metabolism<br>mmu00590:Arachidonic acid metabolism<br>mmu00591:Linoleic acid metabolism	Lipid Metabolism
Melatonin degradation			Melatonin Degradation I<br>Superpathway of Melatonin Degradation
Oxidative Phosphorylation		mmu00190:Oxidative phosphorylation	

Protein folding	GO:0051085 chaperone mediated protein folding requiring cofactor<br>GO:0006458 'de novo' protein folding<br>GO:0051084 'de novo' posttranslational protein folding<br>GO:0006457 protein folding			Protein folding: Response to unfolded proteins<br><br>Protein folding: Folding in normal condition<br>Protein folding: Protein folding nucleus<br><br>Protein folding: ER and cytoplasm
Proteolysis	GO:0006508 proteolysis			Proteolysis: ECM remodeling<br>Proteolysis: Proteolysis in cell cycle and apoptosis<br>Proteolysis: Connective tissue degradation
Retinol metabolism		mmu00830:Retinol metabolism	Retinol Biosynthesis<br><br>Retinoate Biosynthesis I	
RNA processing	GO:0008380~RNA splicing<br><br>GO:0016071 mRNA metabolic process<br>GO:0006397 mRNA processing<br>GO:0006396 RNA processing	mmu03040:Spliceosome		
Steroid biosynthesis	GO:0006694~steroid biosynthetic process<br>GO:0008202~steroid metabolic process<br>GO:0016125~sterol metabolic process<br>GO:0016126~sterol biosynthetic process<br>GO:0008203~cholesterol metabolic process	mmu00140:Steroid hormone biosynthesis		
Transcription	GO:0006350 transcription			Transcription: mRNA processing

	GO:0006355 regulation of transcription, DNA-dependent<br>GO:0006357 regulation of transcription from RNA polymerase II promoter<br>GO:0010553 negative regulation of specific transcription from RNA polymerase II promoter<br>GO:0016481 negative regulation of transcription<br>GO:0045892 negative regulation of transcription, DNA-dependent			Transcription: Transcription by RNA polymerase II
Xenobiotic metabolism	GO:0006805 xenobiotic metabolic process	mmu00980:Metabolism of xenobiotics by cytochrome P450	Xenobiotic Metabolism Signaling	
	GO:0009410 response to xenobiotic stimulus	mmu00982:Drug metabolism	Nicotine Degradation II	
	GO:0009404~toxin metabolic process	mmu00983:Drug metabolism	Nicotine Degradation III	
	GO:0018894~dibenzo-p-dioxin metabolic process		Bupropion Degradation	
	GO:0042537~benzene and derivative metabolic process		Acetone Degradation I (to Methylglyoxal)	
**Homeostasis**				
Calcium sigaling	GO:0006874 cellular calcium ion homeostasis	mmu04020:Calcium signaling pathway	Calcium Signaling	
	GO:0055074 calcium ion homeostasis		D-myo-inositol (1,4,5)-Trisphosphate Biosynthesis	
	GO:0051480~cytosolic calcium ion homeostasis		Regulation of Cellular Mechanics by Calpain Protease	
Circadian rhythm	GO:0007623 circadian rhythm	mmu04710:Circadian rhythm	Circadian Rhythm Signaling	
