# Supplementary material for: Comparative transcriptomic analyses to scrutinize the assumption that genotoxic PAHs exert effects via a common mode of action
Source: Arch Toxicol. 2015 Sep 16;90(10):2461–80. doi: 10.1007/s00204-015-1595-5 (PMC5043007; doi:10.1007/s00204-015-1595-5)
Supplement: Supplementary file 3 — Supplementary material 3 (PDF 26570 kb) [file 204_2015_1595_MOESM3_ESM.pdf]

Article Title: Comparative Transcriptomic Analyses to Scrutinise the Assumption That Genotoxic Priority PAHs Exert Effects via a Common Mode of Action

Journal: Archives in Toxicology

Authors: Labib S, Williams A, Guo CH, Leingartner K, Arlt VM, Schmeiser HH, Yauk CL, White PA, Halappanavar S*

Corresponding Author: *Sabina Halappanavar, Health Canada, Sabina.halappanavar@hc-sc.gc.ca

Supplementary File 3 – Gene lists for PAHs in forestomach, liver, and lung tissues.

Contents

Supplementary File 3 – Gene lists for PAHs in forestomach, liver, and lung tissues 1
3.1 – FORESTOMACH 2
3.1.1 BaA Forestomach 2
3.1.2 BbF Forestomach 3
3.1.3 BghiP Forestomach 3
3.1.4 BkF Forestomach 7
3.1.5 Chr Forestomach 12
3.1.6 DBahA Forestomach 18
3.1.7 IP Forestomach 18
3.2 LIVER 19
3.2.1 BaA Liver 19
3.2.2 BbF Liver 29
3.2.3 BghiP Liver 38
3.2.4 BkF Liver 40
3.2.5 Chr Liver 46
3.2.6 DBahA Liver 46
3.2.7 IP Liver 55
3.3 LUNG 56
3.3.1 BaA Lung 56
3.3.2 BbF Lung 74
3.3.3 BghiP Lung 105
3.3.4 BkF Lung 107
3.3.5 Chr Lung 113
3.3.6 DBahA Lung 118
3.3.7 IP Lung 134

3.1 – FORESTOMACH

3.1.1 BaA Forestomach. Significant probe list. List of all significantly differentially expressed probes in at least 1 treatment group (FDR P ≤ 0.05, fold change ± 1.5) in response to sub-chronic oral exposure to 20, 40, and 80 mg/kg-bw/day benz(a)anthracene in the forestomach. The list is sorted from highest to lowest fold change in the 80 mg/kg-bw/day treatment group.

			20 mg/kg-bw/day		40 mg/kg-bw/day		80 mg/kg-bw/day	
Agilent Probe	Accession Number	Gene Symbol	*FDR P value*	*Fold change*	*FDR P value*	*Fold change*	*FDR P value*	*Fold change*
A_51_P113906	NM_025467	Gkn2	0.00	16.3	0.48	6.2	0.47	5.2
A_51_P216679	NM_011922	Anxa10	0.00	33.2	0.76	5.7	0.82	3.3
A_55_P2011097	NM_029727	Gsdma2	0.00	8.9	0.69	3.5	0.67	2.8
A_51_P333923	NM_133681	Tspan1	0.00	19.7	0.82	3.8	0.84	2.7
A_55_P2112005	NM_009362	Tff1	0.00	4.7	0.44	2.8	0.45	2.6
A_55_P2140042	NM_010659	Krt31	0.96	1.0	0.97	1.0	0.00	2.6
A_66_P133397	NM_145126	Chi3l4	0.00	17.0	0.75	4.1	0.84	2.4
A_55_P2025687	NM_080457	Muc4	0.00	6.8	0.80	2.5	0.81	2.0
A_66_P115236	AK007416	1810010D01Rik	0.00	11.7	0.87	2.6	0.89	1.9
A_55_P2157872	NM_001079695	Sfrs5	0.29	1.3	0.00	1.7	0.00	1.9
A_55_P2114187	XM_884904	LOC620515	0.00	20.9	0.88	2.9	0.91	1.8
A_51_P169476	NM_008570	Mcpt1	0.00	5.4	0.76	2.3	0.84	1.7
A_55_P2140195	XM_900959	Gm15517	0.88	-1.1	0.91	-1.1	0.00	1.7
A_55_P2215640	AK007112	1700102J08Rik	1.00	1.0	0.91	-1.1	0.00	1.6
A_55_P2116833	NM_029886	9430038I01Rik	0.27	1.3	0.00	1.5	0.00	1.5
A_30_P01028944			0.14	1.3	0.00	1.5	0.04	1.4
A_30_P01017548			0.51	1.2	0.00	1.5	0.06	1.4
A_55_P2111399	NM_001129803	Pcp2	0.86	1.1	0.00	1.8	0.60	1.1
A_30_P01021428			0.91	-1.1	0.00	-4.2	0.90	1.1
A_55_P2108903	AK038731		0.00	-1.4	0.20	-1.4	0.04	-1.5
A_30_P01026599			0.00	-1.4	0.15	-1.4	0.00	-1.5
A_55_P2015734	NM_028769	Syvn1	0.89	-1.1	0.17	-1.5	0.00	-1.5
A_30_P01024138			0.89	-1.1	0.88	-1.1	0.00	-1.5
A_51_P441970	NM_175162	Stox2	0.62	-1.2	0.61	-1.2	0.00	-1.5
A_66_P100937	XM_001487796	Zfp33b	0.78	-1.2	0.15	-1.5	0.00	-1.7
A_55_P2286493	AK016664	4933405E24Rik	0.76	-1.2	0.80	-1.2	0.00	-1.9
A_52_P12877	NM_031165	Hspa8	0.61	-1.3	0.22	-1.6	0.00	-1.9
A_55_P1968304			0.42	-1.4	0.07	-1.7	0.00	-1.9
A_52_P671812	XM_918536	LOC641192	0.19	-1.4	0.00	-1.7	0.00	-1.9
A_55_P2080603	NM_031165	Hspa8	0.60	-1.3	0.07	-1.6	0.00	-1.9
A_55_P2069721	NM_031165	Hspa8	0.60	-1.3	0.12	-1.6	0.00	-1.9
A_55_P2035320	NM_017373	Nfil3	0.76	-1.3	0.25	-1.7	0.04	-2.1
A_55_P2176963	NM_013559	Hsph1	0.59	-1.6	0.31	-1.9	0.04	-2.3
A_55_P2135967	XM_979793	Gm7816	0.76	-1.4	0.32	-1.8	0.00	-2.3
A_55_P1993404			0.67	-1.4	0.17	-1.9	0.00	-2.4
A_55_P2068459	NM_010479	Hspa1a	0.31	-1.8	0.00	-2.6	0.00	-2.4
A_55_P2003513	NM_013559	Hsph1	0.48	-1.6	0.30	-1.9	0.00	-2.5
A_55_P2087984	NM_001164671	Dnaja1	0.64	-1.5	0.15	-2.0	0.00	-2.6

3.1.2 BbF Forestomach. Significant probe list. List of all significantly differentially expressed probes in at least 1 treatment group (FDR P ≤ 0.05, fold change ± 1.5) in response to sub-chronic oral exposure to 25, 50, and 100 mg/kg-bw/day benzo(b)fluoranthene in the forestomach. The list is sorted from highest to lowest fold change in the 100 mg/kg-bw/day treatment group.

			25 mg/kg-bw/day		50 mg/kg-bw/day		100 mg/kg-bw/day	
Agilent Probe	Accession Number	Gene Symbol	FDR P value	Fold change	FDR P value	Fold change	FDR P value	Fold change
A_55_P2032079	NM_016974	Dbp	0.22	2.3	0.00	3.3	0.00	6.6
A_55_P2032081	NM_016974	Dbp	0.33	2.1	0.00	3.1	0.00	5.8
A_51_P464703	NM_021443	Ccl8	0.84	2.3	0.39	3.2	0.02	5.3
A_55_P2035018	NM_020001	Clec4n	0.18	1.9	0.32	1.7	0.00	2.6
A_51_P284608	NM_001042605	Cd74	0.39	2.0	0.41	1.9	0.02	2.4
A_52_P51078	NM_007801	Ctsh	0.31	1.7	0.09	2.0	0.00	2.4
A_52_P303891	NM_011584	Nr1d2	0.15	1.6	0.15	1.7	0.00	2.4
A_55_P1954393	NM_144796	Susd4	0.29	1.7	0.06	2.0	0.00	2.4
A_55_P2015994	NM_013518	Fgf9	0.06	1.8	0.00	1.9	0.00	2.3
A_52_P462472	NM_001077354	C77370	0.78	1.5	0.26	1.8	0.01	2.2
A_51_P461779	NM_172994	Ppp2r2c	0.34	1.5	0.21	1.6	0.00	2.2
A_55_P1958165	NM_001025610	Ms4a7	0.55	1.6	0.40	1.6	0.02	2.1
A_55_P2148534	BC096461	Nr1d2	0.60	1.5	0.49	1.5	0.02	2.1
A_55_P2024888	NM_021281	Ctss	0.70	1.5	0.43	1.6	0.04	2.0
A_51_P278868	NM_010387	H2-DMb1	0.21	1.8	0.21	1.8	0.02	2.0
A_51_P223776	NM_145434	Nr1d1	0.75	1.5	0.43	1.6	0.02	2.0
A_55_P2181738	NM_013590	Lyz1	0.29	1.9	0.39	1.7	0.04	1.9
A_30_P01032527			0.00	-2.0	0.90	1.1	0.89	-1.1
A_30_P01025874			0.00	-2.1	0.86	1.2	0.86	-1.1
A_55_P1993858	NR_001460	Rmrp	0.00	-1.5	0.90	-1.1	0.38	-1.2
A_30_P01031385			0.00	-2.1	0.99	-1.0	0.58	-1.4
A_55_P1988384	NM_007515	Slc7a3	0.00	-1.5	0.38	-1.3	0.02	-1.4
A_55_P2010912	NM_010589	Jak3	0.39	-1.5	0.58	-1.3	0.04	-1.6
A_30_P01023209			0.46	-1.5	0.35	-1.5	0.03	-1.7
A_51_P110471	NM_026993	Ddah1	0.30	-1.6	0.43	-1.5	0.01	-1.9
A_51_P422194	NM_153390	Pxt1	0.36	-1.8	0.28	-1.9	0.04	-2.0
A_51_P338443	NM_020581	Angptl4	0.76	-1.6	0.47	-1.7	0.03	-2.3
A_52_P493620	NM_026218	Fgfr1op2	0.30	-1.7	0.00	-2.1	0.00	-2.3
A_55_P1978895	NM_177578	Skint3	0.09	-12.0	0.00	-21.2	0.02	-9.1

3.1.3 BghiP Forestomach. Significant probe list. List of all significantly differentially expressed probes in at least 1 treatment group (FDR P ≤ 0.05, fold change ± 1.5) in response to sub-chronic oral exposure to 6.25, 12.5, and 25 mg/kg-bw/day benzo(ghi)perylene in the forestomach. The list is sorted from highest to lowest fold change in the 25 mg/kg-bw/day treatment group.

			6.25 mg/kg-bw/day		12.5 mg/kg-bw/day		25 mg/kg-bw/day	
Agilent Probe	Accession Number	Gene Symbol	FDR P value	Fold change	FDR P value	Fold change	FDR P value	Fold change

A_55_P2114187	XM_884904	LOC620515	0.88	3.5	0.12	24.0	0.02	43.9
A_55_P2101290	NM_011646	Try4	1.00	1.1	0.32	7.3	0.04	23.2
A_55_P2095513	NM_001003405	Try5	0.97	1.5	0.31	6.2	0.02	21.8
A_55_P2042860	NM_001003664	Gm5409	0.97	1.3	0.28	4.7	0.02	12.3
A_55_P2013555	NM_053243	Prss1	0.96	1.4	0.37	3.6	0.00	11.0
A_55_P1983528	NM_025458	Tmed6	0.97	1.4	0.43	3.7	0.04	10.9
A_51_P136781	NM_018874	Pnliprp1	0.97	1.3	0.37	3.5	0.02	10.0
A_55_P2006499	NM_011935	Esrrg	0.97	1.2	0.37	3.1	0.04	8.2
A_51_P220806	NM_008110	Gdf9	0.96	1.4	0.31	3.2	0.04	7.6
A_51_P307741	NM_009827	Cckar	0.97	1.3	0.46	2.8	0.04	7.5
A_52_P340669	NM_010800	Bhlha15	0.99	1.1	0.39	3.0	0.02	7.3
A_51_P418901	NM_009430	Prss2	0.99	1.1	0.48	2.7	0.04	6.6
A_51_P185939	NM_029771	Gper	0.98	1.2	0.53	2.3	0.02	6.2
A_52_P557293	NM_015775	Tmprss2	0.96	1.3	0.55	2.3	0.04	6.0
A_55_P2003216	NM_028078	Igsf5	0.96	1.4	0.37	2.7	0.02	5.7
A_55_P2050354	NM_001024705	Prpmp5	0.97	1.2	0.27	3.0	0.00	5.4
A_30_P01021703			0.96	1.3	0.28	3.0	0.04	5.1
A_55_P2097964	XM_001481064	9030622O22Rik	0.98	1.2	0.50	2.2	0.02	5.0
A_65_P19089	NM_011935	Esrrg	1.00	1.1	0.57	2.2	0.04	5.0
A_55_P2131642	NM_178240	Trim50	0.99	1.1	0.71	1.8	0.04	4.9
A_55_P2002933	NM_008456	Klk1b5	0.94	1.5	0.57	2.0	0.04	4.8
A_30_P01024672			0.99	1.1	0.37	2.7	0.04	4.7
A_55_P2054261	NM_001081314	C2cd4b	0.99	1.1	0.68	1.8	0.04	4.7
A_55_P2019890	NM_001017427	Rasef	0.96	1.3	0.67	1.8	0.04	4.5
A_55_P1999953	NM_207683	Pik3c2g	0.96	1.3	0.48	2.2	0.04	4.4
A_55_P2097962	XM_001481064	9030622O22Rik	1.00	1.1	0.58	2.0	0.02	4.3
A_55_P2108708	NM_020574	Kcne3	0.95	1.4	0.72	1.6	0.04	4.2
A_52_P517762	NM_023557	Slc44a4	0.97	1.2	0.57	2.0	0.04	4.2
A_55_P1985544	NM_029911	Kcnk10	0.99	1.1	0.62	1.9	0.04	4.1
A_55_P2236268	NM_172804	Syt16	0.97	1.2	0.63	1.9	0.04	4.1
A_55_P2031939	NM_009700	Aqp4	0.96	1.3	0.50	1.8	0.00	3.8
A_55_P2394490	AK052609	D630004K10Rik	0.99	1.1	0.35	2.2	0.04	3.8
A_52_P564413	NM_207683	Pik3c2g	0.99	1.1	0.53	1.9	0.04	3.8
A_51_P314669	NM_011128	Pnliprp2	0.97	1.2	0.70	1.6	0.04	3.8
A_52_P662711	NM_027211	Anxa13	0.96	1.2	0.76	1.5	0.04	3.7
A_55_P2080021	NM_011864	Papss2	0.97	1.2	0.32	2.3	0.04	3.7
A_55_P1973588	NM_177084	Slc9a4	0.96	1.3	0.67	1.7	0.04	3.7
A_30_P01025658			0.96	-1.2	0.55	1.9	0.04	3.6
A_55_P2176565	NM_001122993	B3galt5	1.00	1.0	0.80	1.4	0.04	3.6
A_51_P480073	NM_007689	Chad	0.96	1.3	0.57	1.8	0.04	3.6
A_52_P625315	NR_003617	1700018B24Rik	0.96	1.2	0.68	1.7	0.04	3.5
A_30_P01023405			0.98	1.1	0.63	1.6	0.00	3.3
A_55_P2085150	XM_001480594	Gm4461	0.95	1.4	0.27	2.3	0.04	3.3
A_51_P115738	NM_053248	Slc5a5	0.97	1.1	0.53	1.8	0.04	3.3
A_55_P1953919	NM_181407	Me3	0.96	1.2	0.42	1.8	0.02	3.2
A_55_P2042923	NM_013731	Sgk2	0.96	1.3	0.20	2.4	0.02	3.2
A_30_P01030522			0.96	1.2	0.73	1.4	0.04	3.1
A_52_P308669	NM_030703	Cpn1	0.98	1.1	0.41	1.9	0.04	3.1
A_55_P2064701			0.97	1.2	0.66	1.5	0.04	2.9

A_55_P2070185			0.97	1.1	0.67	1.5	0.02	2.9
A_51_P245414	NM_010639	Klk1	0.94	1.3	0.63	1.5	0.00	2.9
A_55_P1953920	NM_181407	Me3	0.97	1.1	0.38	1.8	0.00	2.9
A_51_P393305	NM_198034	Sidt1	0.99	1.0	0.58	1.6	0.00	2.9
A_55_P2071266	BC057333		0.96	1.2	0.31	2.0	0.04	2.8
A_30_P01033545			0.96	1.2	0.47	1.6	0.02	2.8
A_51_P104392	NM_133982	Rpp25	0.96	1.1	0.82	1.2	0.00	2.8
A_30_P01032867			0.95	1.3	0.47	1.7	0.02	2.7
A_55_P2151388	NM_025312	Sostdc1	0.92	1.4	0.25	2.0	0.04	2.7
A_55_P1953783			0.98	-1.1	0.71	1.4	0.02	2.6
A_55_P2147896	NM_001163143	C2cd4a	1.00	-1.0	0.84	1.3	0.02	2.6
A_30_P01027140			1.00	1.0	0.50	1.6	0.02	2.5
A_55_P2147897	NM_001163143	C2cd4a	0.97	1.1	0.73	1.3	0.04	2.5
A_51_P155755	NM_183139	Pld6	0.96	1.2	0.42	1.6	0.04	2.5
A_30_P01026399			0.96	1.2	0.70	1.4	0.04	2.4
A_51_P308048	NM_027294	Cmtm8	0.99	1.1	0.58	1.5	0.04	2.4
A_55_P2020338	NM_172938	Scml4	0.96	1.2	0.54	1.6	0.04	2.4
A_55_P2072666	BC059104		1.00	1.0	0.70	1.3	0.04	2.3
A_51_P435068	NM_025826	Acadsb	0.95	1.2	0.39	1.6	0.04	2.3
A_51_P249313	NM_028705	Herc3	0.91	1.3	0.22	1.7	0.02	2.3
A_55_P2010622	NM_033354	Sec16b	1.00	1.0	0.70	1.3	0.04	2.2
A_51_P404193	NM_022435	Sp5	0.99	1.0	0.71	1.3	0.04	2.2
A_51_P471791	NM_145838	St8sia6	0.92	1.3	0.53	1.4	0.04	2.2
A_51_P456208	NM_011575	Tff3	0.97	1.1	0.65	1.4	0.04	2.2
A_55_P2005549	NM_177839	Tnn	0.96	1.1	0.23	1.8	0.04	2.2
A_51_P211998	NM_028943	Sgms2	0.98	1.1	0.68	1.3	0.04	2.1
A_55_P2060922	NM_153131	Unc5a	0.96	1.1	0.00	2.1	0.00	2.1
A_55_P2096602			0.98	1.1	0.48	1.5	0.04	2.0
A_51_P341336	NM_025778	Bcl2l14	0.98	1.1	0.71	1.3	0.04	2.0
A_52_P238846	NM_011794	Bpnt1	0.96	1.1	0.52	1.3	0.02	2.0
A_51_P193336	NM_016773	Nucb2	0.81	1.4	0.19	1.7	0.04	2.0
A_51_P229613	NM_008792	Pcsk2	0.95	1.2	0.36	1.4	0.00	2.0
A_55_P2127357			0.95	1.2	0.40	1.5	0.04	1.9
A_30_P01025051			0.75	1.3	0.04	1.9	0.00	1.9
A_55_P1971991	NM_001083916	1810019J16Rik	0.66	1.4	0.19	1.6	0.00	1.9
A_55_P2101231	NM_001127338	Aldh7a1	0.96	-1.1	0.48	1.4	0.04	1.9
A_51_P188126	NM_145523	Gca	0.97	1.1	0.52	1.3	0.02	1.9
A_55_P2064862	NM_010492	Ica1	0.97	1.1	0.78	1.2	0.04	1.9
A_52_P523445	NM_028069	Mupcdh	1.00	-1.0	0.72	1.2	0.04	1.9
A_51_P279038	NM_008904	Ppargc1a	0.97	1.1	0.67	1.3	0.04	1.9
A_51_P268559	NM_029573	Idh3a	0.88	1.2	0.18	1.4	0.00	1.8
A_55_P2135153	NM_153558	Lcn13	0.97	1.1	0.70	1.3	0.04	1.8
A_30_P01028353			0.30	1.6	0.14	1.6	0.04	1.7
A_30_P01025230			0.74	1.4	0.30	1.5	0.04	1.7
A_51_P466148	NM_007533	Bckdha	1.00	1.0	0.45	1.4	0.04	1.7
A_55_P1997651	NM_207687	Espn	0.74	1.3	0.00	1.8	0.02	1.7
A_55_P2048259	NM_146133	Golph3l	0.96	1.1	0.35	1.4	0.04	1.7
A_51_P403564	NM_008499	Lhx5	0.99	1.0	0.85	1.1	0.04	1.7
A_55_P2054362	XM_001472585	LOC100048875	0.90	1.2	0.00	1.8	0.07	1.7

A_55_P2183854	NM_001012322	Sctr	0.94	1.2	0.33	1.4	0.02	1.7
A_55_P2157627			0.97	1.1	0.16	1.5	0.04	1.6
A_55_P1983368	NM_001162533	1700029G01Rik	0.95	1.1	0.45	1.3	0.04	1.6
A_55_P2026982	NM_173744	2610019F03Rik	0.72	1.3	0.22	1.5	0.04	1.6
A_52_P542794	NM_001002004	2610507B11Rik	0.97	1.1	0.26	1.4	0.02	1.6
A_55_P1959833	NM_172923	AI118078	0.96	1.1	0.81	1.1	0.04	1.6
A_55_P1952385	NM_033571	Fkbp6	1.00	1.0	0.85	1.1	0.04	1.6
A_55_P2067342	XM_001478579	Gm9782	0.96	1.1	0.33	1.4	0.04	1.6
A_55_P2081630	NM_021610	Gpa33	0.91	1.2	0.33	1.3	0.04	1.6
A_51_P465232	NM_139295	Mcfd2	0.75	1.2	0.14	1.3	0.00	1.6
A_66_P108770	NM_024188	Oxct1	0.97	1.1	0.43	1.3	0.04	1.6
A_55_P1985337	NM_031395	Sytl3	0.91	1.2	0.58	1.2	0.04	1.6
A_51_P284244	NM_025360	Tmed3	0.98	1.0	0.76	1.2	0.04	1.6
A_55_P2103115	NM_172843	Tor1aip2	0.89	1.2	0.18	1.4	0.00	1.6
A_55_P2175145	AK154426		1.00	1.0	0.89	1.1	0.04	1.5
A_30_P01028140			0.99	1.0	0.94	-1.0	0.00	1.5
A_55_P2062538	XM_984645	1700027A15Rik	0.67	1.2	0.16	1.3	0.00	1.5
A_55_P2045876	XM_001477165	2900008C10Rik	0.96	1.1	0.85	1.1	0.04	1.5
A_51_P517787	NM_146047	Clptm1l	0.90	1.1	0.38	1.2	0.00	1.5
A_52_P508317	NM_025745	Erlec1	0.58	1.3	0.23	1.4	0.04	1.5
A_55_P2026340	NM_001161765	Fmo5	0.91	1.2	0.56	1.2	0.04	1.5
A_52_P763938	XM_001478475	LOC100047597	1.00	1.0	0.67	1.2	0.04	1.5
A_55_P1962736	NM_019787	Sec23b	0.95	-1.1	0.51	1.2	0.04	1.5
A_55_P2020203	NM_018780	Sfrp5	0.79	1.2	0.04	1.5	0.04	1.5
A_51_P419971	NM_027992	Tmem106b	0.83	1.2	0.18	1.3	0.02	1.5
A_55_P1963483	NM_030559	Vps16	1.00	1.0	0.00	-5.5	0.81	1.3
A_55_P2087984	NM_001164671	Dnaja1	0.00	-1.7	0.54	-1.3	0.98	-1.0
A_55_P1999287	XM_355880	Ovol3	0.53	-1.3	0.04	-1.5	0.40	-1.2
A_55_P2004370	NM_009802	Car6	0.20	-1.3	0.00	-1.5	0.02	-1.3
A_55_P2417569	NM_009116	Prrx2	0.30	-1.4	0.04	-1.5	0.13	-1.3
A_55_P2063283	XM_001001076		0.00	-1.5	0.00	-1.4	0.02	-1.4
A_51_P416509	NM_030609	Hist1h1a	0.50	-1.3	0.00	-1.7	0.09	-1.4
A_51_P284577	NM_010489	Hyal2	0.88	-1.2	0.00	-1.5	0.09	-1.4
A_55_P2000454	XM_001478824	Tspan18	0.96	-1.1	0.00	-1.5	0.06	-1.4
A_55_P1972948	NM_176954	Brunol5	0.96	-1.1	0.65	-1.2	0.00	-1.5
A_55_P2131766	NM_023665	D4Wsu53e	0.00	-2.4	0.14	-1.9	0.29	-1.5
A_55_P2057537	NM_001109657	Gas7	1.00	-1.0	0.30	-1.2	0.00	-1.5
A_51_P286488	NM_133662	Ier3	0.96	-1.1	0.31	-1.3	0.04	-1.5
A_55_P2117033	NM_028860	Mtmr3	0.98	1.0	0.49	-1.2	0.04	-1.5
A_51_P355943	NM_138656	Mvd	0.96	1.1	0.81	-1.1	0.04	-1.5
A_55_P2074796	NM_007671	Cdkn2c	0.96	-1.1	0.27	-1.4	0.04	-1.6
A_52_P432580	XM_001473421	LOC100044968	0.60	-1.2	0.00	-1.6	0.00	-1.6
A_51_P441974	NM_008847	Pip5k1a	0.96	1.1	0.64	-1.2	0.02	-1.6
A_55_P1952877	NM_027246	Snrpf	1.00	-1.0	0.27	-1.4	0.04	-1.6
A_30_P01029299			0.99	1.0	0.66	-1.3	0.04	-1.7
A_51_P381260	NM_008761	Fxyd5	0.64	-1.3	0.18	-1.4	0.04	-1.7
A_51_P127681	NM_013885	Clic4	0.00	-2.0	0.19	-1.6	0.04	-1.8
A_51_P246854	NM_009606	Acta1	0.96	-1.1	0.91	-1.1	0.04	-1.9
A_52_P90363	NM_029803	Ifi27l2a	0.81	-1.6	0.21	-2.1	0.02	-2.8

A_51_P431329	NM_007606	Car3	0.37	-3.7	0.31	-2.9	0.00	-6.2

3.1.4 BkF Forestomach. Significant probe list. List of all significantly differentially expressed probes in at least 1 treatment group (FDR P ≤ 0.05, fold change ± 1.5) in response to sub-chronic oral exposure to 25, 50, and 100 mg/kg-bw/day benzo(k)fluoranthene in the forestomach. The list is sorted from highest to lowest fold change in the 100 mg/kg-bw/day treatment group.

			25 mg/kg-bw/day		50 mg/kg-bw/day		100 mg/kg-bw/day	
Agilent Probe	Accession Number	Gene Symbol	*FDR P value*	*Fold change*	*FDR P value*	*Fold change*	*FDR P value*	*Fold change*
A_55_P2124461	XM_001472850	Gm2251	1.00	-1.4	0.08	2.6	0.03	2.3
A_55_P2046877	NM_008239	Foxq1	1.00	1.1	0.04	5.5	0.43	2.2
A_55_P1977628	NM_021362	Pappa	0.00	2.4	0.92	1.2	0.06	2.0
A_51_P234692	NR_003513	Neat1	1.00	1.3	0.93	1.2	0.03	2.0
A_52_P222230	XM_001476722		1.00	-1.8	0.03	5.4	0.51	2.0
A_55_P2090505	XM_001480011	Gm4382	1.00	-1.3	0.03	2.1	0.06	1.8
A_55_P2293351	AK082896	C430010C01	0.84	1.6	0.98	1.1	0.03	1.7
A_51_P378789	NM_018866	Cxcl13	1.00	1.2	0.82	1.2	0.03	1.7
A_30_P01018771			0.95	1.4	0.99	1.0	0.04	1.6
A_51_P184300	NM_010087	Dtna	0.87	1.4	0.71	1.2	0.03	1.6
A_51_P423880	NM_025891	Smarcd3	1.00	-1.1	0.47	1.3	0.04	1.6
A_55_P2097913	NM_001081053	Itga10	0.67	1.5	0.49	1.3	0.03	1.5
A_55_P2040815	NM_153545	Lrrc45	0.97	1.4	1.00	-1.0	0.04	1.5
A_55_P1976574	NM_016712	Tmod4	0.82	1.4	0.62	1.2	0.03	1.5
A_55_P2356840	BC029726	1600020E01Rik	0.74	1.4	0.43	1.3	0.00	1.5
A_55_P1969032	NM_011268	Rgs9	0.89	1.3	0.86	1.1	0.03	1.5
A_55_P1994047	XM_619437	Gm5813	1.00	1.2	0.51	1.3	0.03	1.5
A_52_P624149	NM_011631	Hsp90b1	1.00	1.1	0.04	2.0	0.30	1.5
A_55_P2044045	NM_011732	Ybx1	1.00	1.2	0.00	1.9	0.18	1.4
A_55_P2028159	NM_001081343	3110043O21Rik	1.00	1.2	0.02	1.9	0.82	1.2
A_55_P2115151	NM_153420	Acpl2	1.00	1.1	0.04	1.8	0.63	1.2
A_55_P2035951	NM_001163042	Haus8	1.00	1.3	0.02	1.6	0.97	1.0
A_30_P01020072			1.00	1.1	0.00	1.6	0.96	1.0
A_66_P119376	NM_177715	Kctd12	1.00	1.2	0.02	-1.8	0.41	-1.3
A_51_P371119	NM_012001	Cops4	1.00	-1.1	0.04	-1.5	0.24	-1.3
A_55_P2115257	NM_001037997	Fert2	1.00	-1.1	0.03	-1.5	0.22	-1.3
A_51_P301736	NM_028032	Ppp2r2a	1.00	-1.1	0.02	-1.5	0.03	-1.3
A_55_P2103596	NM_011774	Slc30a4	1.00	-1.1	0.02	-1.5	0.13	-1.3
A_55_P2046443	NM_145395	Duoxa1	1.00	-1.1	0.02	-2.9	0.82	-1.3
A_55_P2062777	NM_015776	Mfap5	1.00	1.1	0.04	-1.6	0.13	-1.4
A_51_P320401	NM_009000	Rab24	1.00	1.0	0.00	-2.1	0.35	-1.4
A_30_P01033587			1.00	-1.0	0.04	-1.8	0.16	-1.4
A_51_P439403	NM_011059	Padi1	1.00	-1.0	0.00	-1.8	0.15	-1.4
A_52_P674309	NM_013826	Mocs2	1.00	-1.1	0.00	-1.7	0.13	-1.4
A_55_P2044710	NM_080444	Asb10	1.00	-1.2	0.05	-1.5	0.03	-1.4
A_55_P2076533	NM_026784	Pmvk	0.83	-1.4	0.04	-1.6	0.04	-1.4
A_51_P455866	NM_010125	Elf5	1.00	1.3	0.04	-2.7	0.59	-1.5
A_30_P01026224			1.00	1.1	0.04	-2.6	0.54	-1.5

A_55_P2072801	NM_029806	Lypd5	1.00	1.0	0.00	-2.1	0.24	-1.5
A_55_P2062549	NR_028307	Gm6524	1.00	1.0	0.22	-1.4	0.04	-1.5
A_51_P345593	NM_009269	Sptlc1	1.00	1.0	0.12	-1.4	0.00	-1.5
A_51_P438952	NM_026121	Bag4	1.00	-1.0	0.20	-1.4	0.04	-1.5
A_51_P510849	NM_030018	Tmem50b	1.00	-1.0	0.43	-1.3	0.03	-1.5
A_51_P147373	NM_133803	Dpp3	1.00	-1.1	0.05	-1.6	0.02	-1.5
A_51_P253547	NM_009984	Ctsl	1.00	-1.1	0.00	-2.1	0.12	-1.5
A_30_P01030433			1.00	-1.1	0.24	-1.3	0.03	-1.5
A_51_P117604	NM_025887	Rab5a	1.00	-1.1	0.03	-1.4	0.00	-1.5
A_55_P2082235			1.00	-1.1	0.33	-1.3	0.00	-1.5
A_51_P519189	NM_018799	Eif3i	1.00	-1.2	0.36	-1.3	0.02	-1.5
A_55_P2045258	NM_010119	Ehd1	0.75	-1.4	0.16	-1.4	0.00	-1.5
A_66_P102232	NM_001166537	Hmga1	0.68	-1.5	0.07	-1.5	0.03	-1.5
A_30_P01024134			1.00	1.1	0.00	-2.7	0.32	-1.6
A_51_P189272	NM_172051	Tmcc3	1.00	1.0	0.02	-2.0	0.10	-1.6
A_55_P2116924	NM_001037999	Dbi	1.00	1.0	0.10	-1.3	0.00	-1.6
A_55_P1963737	NM_010241	Aktip	1.00	-1.0	0.00	-1.5	0.00	-1.6
A_52_P489778	NM_178688	Ablim1	1.00	-1.0	0.15	-1.6	0.03	-1.6
A_52_P77204	NM_153542	Lrrc20	1.00	-1.1	0.02	-1.7	0.00	-1.6
A_55_P1999334	NM_008385	Inpp5b	1.00	-1.1	0.00	-1.9	0.03	-1.6
A_52_P480301	NM_029012	Sppl3	1.00	-1.1	0.04	-2.0	0.19	-1.6
A_55_P1956627	NM_001134385	Gpr160	1.00	-1.1	0.04	-2.0	0.13	-1.6
A_55_P2170514	NM_018754	Sfn	1.00	-1.1	0.04	-2.3	0.24	-1.6
A_51_P128575	NM_011681	Scgb1a1	1.00	-1.1	0.03	-3.6	0.52	-1.6
A_51_P222590	NM_007481	Arf6	1.00	-1.1	0.39	-1.4	0.04	-1.6
A_52_P194805	NM_026521	Zfp706	1.00	-1.1	0.29	-1.4	0.04	-1.6
A_55_P2149363	NM_172286	6430548M08Rik	1.00	-1.2	0.00	-1.8	0.02	-1.6
A_55_P2148912	NM_026416	S100a16	1.00	-1.2	0.03	-2.0	0.06	-1.6
A_55_P1980119	NM_024472	Gltpd1	1.00	-1.2	0.12	-1.6	0.03	-1.6
A_66_P132062	XM_001474815	Gm5608	1.00	-1.3	0.00	-1.8	0.02	-1.6
A_52_P676063	NM_026734	Tmem126b	1.00	-1.3	0.89	-1.1	0.03	-1.6
A_55_P2148957	NM_001085542	Gm13124	0.95	-1.4	0.10	-1.6	0.04	-1.6
A_51_P489153	NM_023733	Crot	1.00	1.1	0.45	-1.4	0.04	-1.7
A_52_P42834	NM_013918	Usp25	1.00	-1.1	0.00	-1.7	0.00	-1.7
A_55_P2070441	NM_031183	Sp6	1.00	-1.1	0.02	-2.1	0.08	-1.7
A_55_P2169415	NM_001037711	Cgn	1.00	-1.1	0.37	-1.5	0.04	-1.7
A_30_P01027526			1.00	-1.1	0.09	-1.5	0.00	-1.7
A_55_P1966620	NM_025887	Rab5a	1.00	-1.2	0.02	-1.9	0.03	-1.7
A_66_P124420			1.00	-1.2	0.52	-1.4	0.04	-1.7
A_66_P126313	NM_175092	Rhof	1.00	-1.2	0.99	-1.0	0.03	-1.7
A_55_P2054743	NM_172507	Sh3bgrl2	1.00	-1.2	0.13	-1.6	0.02	-1.7
A_30_P01026871			1.00	-1.2	0.13	-1.6	0.02	-1.7
A_55_P1979330	NM_011932	Dapp1	1.00	-1.3	0.21	-1.5	0.03	-1.7
A_30_P01026014			1.00	-1.3	0.09	-1.7	0.03	-1.7
A_66_P112886	NM_011908	Ubl3	1.00	-1.3	0.48	-1.3	0.00	-1.7
A_55_P1993518	NM_001113362	Tbc1d14	1.00	-1.1	0.07	-1.8	0.00	-1.8
A_55_P1956593	NM_148927	Plekha4	1.00	-1.1	0.16	-1.6	0.00	-1.8
A_51_P187082	NM_008062	G6pdx	1.00	-1.1	0.24	-1.5	0.00	-1.8
A_55_P2007966	NM_177175	Tmem215	1.00	-1.2	0.03	-1.9	0.02	-1.8

A_55_P2086329	NM_016879	Krt85	1.00	-1.2	0.15	-1.8	0.04	-1.8
A_51_P329370	NM_030093	Snrnp25	1.00	-1.2	0.45	-1.5	0.03	-1.8
A_52_P203948	NM_177845	Pla2g4e	1.00	1.2	0.04	-3.4	0.30	-1.9
A_51_P140321	NM_026779	Mocos	1.00	-1.0	0.04	-2.5	0.11	-1.9
A_55_P2140057	NM_001159374	Krt32	1.00	-1.0	0.02	-4.1	0.31	-1.9
A_55_P2054331	XM_982830	LOC236260	1.00	-1.0	0.08	-1.9	0.03	-1.9
A_55_P2162160	NM_009672	Anp32a	1.00	-1.1	0.02	-2.7	0.13	-1.9
A_51_P423127	NM_001077411	Gba	1.00	-1.1	0.08	-1.9	0.04	-1.9
A_55_P2007001	NM_023465	Ctnnbip1	1.00	-1.1	0.07	-2.0	0.03	-1.9
A_52_P229709	NM_025356	Ube2d3	1.00	-1.2	0.16	-1.7	0.04	-1.9
A_52_P266530	NM_031156	Ide	1.00	-1.3	0.00	-2.7	0.06	-1.9
A_52_P434974	NM_152234	Ubqln1	1.00	-1.3	0.22	-1.8	0.04	-1.9
A_51_P113773	NM_029420	Giyd2	1.00	-1.4	0.10	-1.9	0.04	-1.9
A_55_P2004208	NM_007847	Defa-rs2	0.27	-2.2	0.03	-2.1	0.02	-1.9
A_51_P479914	NM_029094	Pik3cb	1.00	1.0	0.15	-1.9	0.03	-2.0
A_66_P115996	NM_172404	Ccbl1	1.00	-1.0	0.04	-2.5	0.09	-2.0
A_52_P12623	NM_134253	Bnipl	1.00	-1.0	0.03	-3.0	0.10	-2.0
A_55_P2120777	XM_355890	Vsig10l	1.00	-1.0	0.02	-3.0	0.05	-2.0
A_52_P436643	NM_018830	Asah2	1.00	-1.1	0.02	-2.3	0.00	-2.0
A_51_P491504	NM_172532	Aldh5a1	1.00	-1.1	0.02	-2.5	0.04	-2.0
A_51_P317941	NM_022032	Perp	1.00	-1.1	0.02	-2.9	0.14	-2.0
A_52_P63680	NM_175731	Acer1	1.00	-1.1	0.11	-1.9	0.04	-2.0
A_51_P130475	NM_009523	Wnt4	1.00	-1.2	0.02	-2.6	0.03	-2.0
A_30_P01023156			1.00	-1.2	0.12	-1.9	0.04	-2.0
A_55_P2046408			1.00	-1.2	0.13	-1.7	0.00	-2.0
A_55_P2076876	NM_001085503	Aadacl3	1.00	-1.3	0.07	-2.2	0.03	-2.0
A_52_P95910	NM_011673	Ugcg	1.00	-1.4	0.07	-1.8	0.00	-2.0
A_55_P2076994	NM_007845	Defa-rs10	0.59	-1.7	0.03	-2.0	0.00	-2.0
A_55_P2064351	NM_011703	Vipr1	1.00	1.4	0.04	-3.8	0.28	-2.1
A_51_P400366	NM_021375	Rhbg	1.00	1.1	0.04	-3.4	0.24	-2.1
A_55_P2124976	NM_145890	Grhl1	1.00	1.1	0.02	-3.4	0.11	-2.1
A_55_P2185821	NM_007819	Cyp3a13	1.00	1.0	0.04	-2.1	0.00	-2.1
A_55_P1967820	NM_145489	AI661453	1.00	1.0	0.03	-2.1	0.00	-2.1
A_55_P2171378	NM_001164557	Pdzk1ip1	1.00	-1.0	0.03	-3.4	0.13	-2.1
A_51_P410949	NM_001081176	Polr3g	1.00	-1.1	0.04	-2.6	0.07	-2.1
A_55_P2002757	NM_008528	Blnk	1.00	-1.1	0.00	-2.6	0.02	-2.1
A_55_P2079713	NM_153528	Gramd1c	1.00	-1.1	0.02	-2.8	0.09	-2.1
A_51_P314285	NM_026436	Tmem86a	1.00	-1.1	0.38	-1.6	0.00	-2.1
A_55_P1997936	NM_010476	Hsd17b7	1.00	-1.2	0.02	-2.4	0.04	-2.1
A_52_P355084	NM_144797	Metrnl	1.00	-1.2	0.00	-2.5	0.02	-2.1
A_55_P2078073	NM_145384	Pqlc2	1.00	-1.3	0.14	-2.0	0.03	-2.1
A_51_P447258	NM_009659	Alox12b	1.00	-1.3	0.07	-2.2	0.02	-2.1
A_55_P1959550	NM_001033320	Rltpr	1.00	-1.4	0.07	-2.3	0.04	-2.1
A_66_P101703	NM_001037822	Krtap5-5	1.00	-1.4	0.08	-2.0	0.03	-2.1
A_51_P257938	NM_020047	Tacstd2	1.00	1.2	0.04	-3.6	0.18	-2.2
A_52_P220783	NM_178745	Tmem229b	1.00	-1.0	0.04	-2.8	0.09	-2.2
A_55_P2124736	NM_181277	Col14a1	1.00	-1.0	0.02	-3.7	0.13	-2.2
A_30_P01020149			1.00	-1.1	0.00	-2.2	0.00	-2.2
A_55_P2015074	NM_029415	Slc10a6	1.00	-1.2	0.00	-2.9	0.04	-2.2

A_52_P465886	NM_026240	Gramd3	1.00	-1.2	0.32	-1.7	0.03	-2.2
A_55_P2081437	AK031012		1.00	-1.3	0.02	-2.5	0.00	-2.2
A_51_P395921	NM_033607	Uchl4	1.00	-1.3	0.24	-1.8	0.02	-2.2
A_51_P375146	NM_007643	Cd36	1.00	-1.4	0.64	-1.5	0.03	-2.2
A_51_P402160	NM_178763	Zfp750	1.00	1.3	0.04	-3.1	0.13	-2.3
A_55_P2171236	XM_355890	Vsig10l	1.00	-1.0	0.04	-2.7	0.05	-2.3
A_52_P255849	AK160183	Fam57a	1.00	-1.0	0.00	-3.3	0.03	-2.3
A_55_P2184606	AK154115		1.00	-1.1	0.02	-2.6	0.00	-2.3
A_55_P2022861	NM_001039176	Elovl1	1.00	-1.1	0.14	-2.0	0.03	-2.3
A_55_P2135551	NM_025718	Dnase1l2	1.00	-1.2	0.02	-4.3	0.13	-2.3
A_51_P117109	NM_173751	Ilvbl	1.00	-1.2	0.06	-2.0	0.02	-2.3
A_55_P1985304	NM_023860	Krtap5-3	1.00	-1.3	0.07	-2.7	0.04	-2.3
A_51_P210395	NM_053108	Glrx	1.00	-1.4	0.00	-2.8	0.00	-2.3
A_51_P336833	NM_024406	Fabp4	1.00	1.0	0.20	-2.0	0.02	-2.4
A_52_P421713	NM_001004155	9930012K11Rik	1.00	-1.1	0.00	-3.2	0.02	-2.4
A_55_P2022724	NR_030716	5430417L22Rik	1.00	-1.1	0.03	-4.0	0.15	-2.4
A_55_P2081805	NM_026740	Slc46a1	1.00	-1.1	0.07	-2.6	0.03	-2.4
A_30_P01025103			1.00	-1.2	0.03	-4.2	0.28	-2.4
A_51_P212390	NM_133712	Klk10	1.00	-1.2	0.06	-2.7	0.04	-2.4
A_52_P148514	NM_152803	Hpse	1.00	-1.3	0.04	-2.4	0.00	-2.4
A_52_P482251	NM_001010937	Gjb6	1.00	1.3	0.04	-4.9	0.16	-2.5
A_55_P2084965	NM_001039042	Klk13	1.00	1.3	0.02	-5.5	0.26	-2.5
A_55_P1953583	NM_001130513	Ace2	1.00	1.1	0.04	-4.6	0.19	-2.5
A_51_P275435	NM_172411	2310007B03Rik	1.00	1.1	0.06	-2.9	0.04	-2.5
A_55_P2084970	NM_027097	Klk12	1.00	1.1	0.15	-2.2	0.02	-2.5
A_30_P01018307			1.00	-1.0	0.02	-3.0	0.02	-2.5
A_55_P2066559	NM_001168693	Pp11r	1.00	-1.0	0.05	-3.1	0.03	-2.5
A_51_P427516	NM_019576	Thsd1	1.00	-1.1	0.02	-3.4	0.05	-2.5
A_30_P01024948			1.00	-1.2	0.04	-2.7	0.03	-2.5
A_51_P440047	NM_173752	1110067D22Rik	1.00	-1.4	0.00	-2.7	0.00	-2.5
A_52_P605812	NM_178595	Ptrh1	1.00	-1.4	0.05	-2.8	0.02	-2.5
A_51_P487219	NM_012018	Cep110	1.00	-1.5	0.02	-4.2	0.00	-2.5
A_55_P2174143	NM_009265	Sprr1b	1.00	-1.5	0.63	-1.6	0.04	-2.5
A_55_P2023391	NM_001013756	Grhl3	1.00	1.1	0.04	-3.6	0.08	-2.6
A_52_P402677	NM_023903	Lipm	1.00	1.1	0.00	-5.5	0.07	-2.6
A_30_P01029477			1.00	1.0	0.11	-2.4	0.00	-2.6
A_51_P495560	NM_009374	Tgm3	1.00	-1.0	0.03	-3.4	0.03	-2.6
A_51_P441914	NM_008290	Hsd17b2	1.00	-1.0	0.20	-1.7	0.00	-2.6
A_52_P195246	NM_177775	Esyt3	1.00	-1.1	0.02	-3.7	0.06	-2.6
A_51_P344878	NM_022653	Thop1	1.00	-1.2	0.00	-3.0	0.00	-2.6
A_51_P463440	NM_130450	Elovl6	1.00	-1.2	0.04	-3.9	0.10	-2.6
A_65_P10195	NM_022879	Myl7	1.00	1.1	0.04	-3.5	0.07	-2.7
A_55_P1985015	NM_033620	Pard3	1.00	1.1	0.07	-2.7	0.02	-2.7
A_52_P338956	NM_001081169	Aspg	1.00	1.0	0.02	-3.7	0.06	-2.7
A_51_P472726	NM_145978	Pdlim2	1.00	-1.1	0.06	-3.4	0.04	-2.7
A_52_P35064	NM_028454	Tm7sf2	1.00	-1.2	0.04	-3.0	0.02	-2.7
A_55_P2171406	NM_028454	Tm7sf2	1.00	-1.2	0.00	-3.0	0.00	-2.7
A_55_P2015941			1.00	-1.3	0.00	-2.9	0.00	-2.7
A_51_P131888	NM_027112	Capns2	1.00	1.2	0.04	-3.8	0.06	-2.8

A_55_P1987146	NM_030067	Gpr115	1.00	1.1	0.00	-3.5	0.00	-2.8
A_51_P480904	NM_178645	Blmh	1.00	-1.1	0.04	-3.1	0.04	-2.8
A_55_P2063216	NM_019819	Dusp14	1.00	-1.1	0.03	-3.3	0.03	-2.8
A_51_P263965	NM_010442	Hmox1	1.00	-1.3	0.03	-3.6	0.02	-2.8
A_30_P01020140			1.00	-1.3	0.00	-3.9	0.00	-2.8
A_55_P2146655	XM_001472967	Gm2262	1.00	-1.3	0.02	-4.1	0.02	-2.8
A_55_P2029498	NM_207530	Osbpl1a	1.00	-1.3	0.07	-3.2	0.00	-2.8
A_66_P106783	NM_001081664	4833423E24Rik	1.00	1.1	0.00	-4.6	0.04	-2.9
A_30_P01033229			1.00	1.0	0.02	-3.5	0.00	-2.9
A_30_P01018420			1.00	1.0	0.00	-3.7	0.00	-2.9
A_55_P2109057	NM_177354	Vash1	1.00	-1.2	0.02	-3.3	0.00	-2.9
A_51_P173678	NM_029415	Slc10a6	1.00	1.2	0.00	-4.4	0.04	-3.0
A_55_P1997595	NM_028878	Slc6a19	1.00	1.0	0.05	-3.9	0.03	-3.0
A_30_P01022929			1.00	-1.0	0.03	-4.9	0.06	-3.0
A_52_P574759	NM_009036	Rbpjl	1.00	-1.6	0.07	-2.6	0.00	-3.0
A_55_P2130478	AK076465		1.00	1.0	0.02	-5.1	0.06	-3.1
A_55_P2165234	NM_001081961	2300005B03Rik	1.00	-1.0	0.10	-2.9	0.02	-3.1
A_55_P2023046	NM_178730	Tmprss11f	1.00	-1.1	0.04	-4.3	0.06	-3.1
A_55_P2120016	NM_213728	Krt72	1.00	-1.3	0.08	-3.4	0.04	-3.1
A_51_P351923	NR_027827	A030009H04Rik	1.00	1.1	0.06	-4.0	0.04	-3.2
A_55_P2055742	NM_001145875	9530008L14Rik	1.00	-1.0	0.00	-4.0	0.03	-3.2
A_51_P159612	NM_019487	Hebp2	1.00	-1.0	0.03	-4.3	0.02	-3.2
A_55_P2398995	NM_027042	Lelp1	1.00	-1.3	0.00	-3.2	0.00	-3.2
A_51_P429209	NM_011421	Smpd1	1.00	-1.3	0.02	-3.7	0.00	-3.2
A_51_P288876	NM_019631	Tmem45a	1.00	-1.3	0.03	-4.4	0.04	-3.2
A_52_P590625	NM_175532	Nlrp10	1.00	1.3	0.04	-3.9	0.04	-3.3
A_55_P2034475	NM_027548	Serpinb7	1.00	-1.1	0.00	-4.5	0.02	-3.3
A_30_P01031718			1.00	-1.2	0.02	-5.1	0.04	-3.3
A_55_P1953545	NM_010238	Brd2	1.00	-1.3	0.02	-4.2	0.03	-3.3
A_55_P2085340	XM_001475466	Abhd12b	1.00	-1.3	0.00	-4.8	0.00	-3.4
A_51_P438853	NM_025867	Serpinb11	1.00	1.0	0.02	-6.0	0.04	-3.5
A_55_P2057430	NM_027340	Lipn	1.00	-1.0	0.04	-4.6	0.03	-3.5
A_66_P103198	AK131883		1.00	-1.2	0.02	-4.2	0.03	-3.5
A_55_P2118525	XM_001476279	H60c	1.00	-1.3	0.10	-3.6	0.00	-3.6
A_52_P533270	NM_183281	2310005G13Rik	1.00	1.1	0.00	-5.6	0.03	-3.7
A_55_P2157735	NM_013504	Dsc1	1.00	-1.1	0.04	-4.8	0.04	-3.7
A_55_P2062986	NM_001033233	Tmprss11a	1.00	-1.1	0.00	-6.4	0.03	-3.7
A_55_P1983858	NM_022886	Scel	1.00	-1.1	0.00	-6.5	0.04	-3.7
A_51_P272066	NM_025929	2010109I03Rik	1.00	-1.1	0.06	-4.7	0.02	-3.7
A_51_P391805	NM_015789	Dkkl1	1.00	-1.4	0.04	-4.4	0.02	-3.7
A_55_P2004746	NM_183319	Xkrx	1.00	1.1	0.03	-5.4	0.03	-3.9
A_55_P2134938	NM_001135991	Krtap10-4	1.00	-1.8	0.00	-3.7	0.00	-3.9
A_55_P2112882	NM_026945	Adh6a	1.00	1.1	0.07	-4.9	0.03	-4.0
A_55_P1954945	NM_027111	Lyg1	1.00	-1.3	0.03	-4.6	0.03	-4.0
A_55_P1972381	NM_008127	Gjb4	1.00	-1.3	0.02	-4.8	0.02	-4.1
A_55_P2027461	NM_027163	Il1f8	1.00	1.1	0.00	-5.1	0.00	-4.2
A_55_P2021953	NM_146600	Olfr700	1.00	1.0	0.17	-2.8	0.03	-4.2
A_66_P138319	NM_053115	Acox2	1.00	-1.4	0.02	-3.7	0.00	-4.3
A_55_P2071226	NM_001146087	Il1f5	1.00	1.1	0.12	-4.3	0.03	-4.4

A_55_P2038752	NM_146563	Olfr18	1.00	1.0	0.00	-5.1	0.00	-4.4
A_52_P172910	NM_027971	Serpinb12	1.00	-1.1	0.04	-5.2	0.02	-4.6
A_55_P1982533	NM_148941	Elovl4	1.00	-1.1	0.03	-5.2	0.02	-4.7
A_51_P243667	NM_023631	Aox4	1.00	1.0	0.00	-7.4	0.00	-5.0
A_52_P562267	NM_001033819	9130409I23Rik	1.00	-1.2	0.00	-6.7	0.02	-5.0
A_55_P2003746	NM_009126	Serpinb3a	1.00	1.7	0.00	-7.6	0.02	-5.1
A_55_P2085005	NM_011475	Sprr2i	1.00	1.2	0.00	-6.3	0.02	-5.1
A_51_P100997	NM_201363	Serpinb3c	1.00	1.6	0.00	-7.1	0.00	-5.7
A_51_P319180	NM_008475	Krt4	1.00	1.0	0.03	-6.0	0.02	-5.7
A_55_P2133997	NM_175210	Abca12	1.00	-1.0	0.00	-7.0	0.00	-5.7
A_55_P2125451	NR_003185	Sprr2j-ps	1.00	-1.1	0.00	-9.3	0.00	-5.7
A_55_P2050944	NM_019515	Nmu	1.00	-1.1	0.00	-7.9	0.00	-6.4

3.1.5 Chr Forestomach. Significant probe list. List of all significantly differentially expressed probes in at least 1 treatment group (FDR P ≤ 0.05, fold change ± 1.5) in response to sub-chronic oral exposure to 17.5, 50, and 150 mg/kg-bw/day chrysene in the forestomach. The list is sorted from highest to lowest fold change in the 150 mg/kg-bw/day treatment group.

			**17.5 mg/kg-bw/day**		**50 mg/kg-bw/day**		**150 mg/kg-bw/day**	
Agilent Probe	Accession Number	Gene Symbol	*FDR P value*	*Fold change*	*FDR P value*	*Fold change*	*FDR P value*	*Fold change*
A_55_P1958906	NM_007732	Col17a1	0.30	2.3	0.07	3.1	0.01	3.9
A_55_P2388428	AK014513	4631405K08Rik	0.75	1.6	0.15	2.4	0.00	3.3
A_55_P1952915	NM_015825	Sh3bgr	0.47	2.1	0.19	2.5	0.03	3.1
A_55_P2052016	NM_030209	Crispld2	0.15	2.8	0.06	3.1	0.01	3.1
A_30_P01024815			0.26	1.9	0.11	2.0	0.00	2.8
A_55_P2077048	NM_172471	Itih5	0.59	1.7	0.41	1.7	0.04	2.7
A_55_P2023523	NM_011786	Aloxe3	0.17	2.4	0.13	2.2	0.02	2.7
A_30_P01025000			0.26	2.0	0.13	2.0	0.00	2.7
A_55_P1958480	BC089308	LOC545005	0.53	1.5	0.23	1.7	0.00	2.7
A_55_P1974567	NM_175556	Plch2	0.34	2.0	0.07	2.6	0.02	2.6
A_52_P173442	NM_177292	Wscd2	0.00	2.6	0.27	2.0	0.06	2.5
A_55_P1982727	NM_019874	Dnajb5	0.00	2.3	0.30	1.6	0.00	2.5
A_52_P920129	NM_010305	Gnai1	0.35	2.0	0.20	2.0	0.04	2.5
A_30_P01020135			0.73	1.4	0.00	2.5	0.00	2.5
A_30_P01025798			0.72	1.4	0.00	2.4	0.00	2.5
A_51_P234692	NR_003513	Neat1	0.85	1.3	0.00	2.5	0.00	2.5
A_52_P64356	NM_010097	Sparcl1	0.39	1.9	0.24	1.9	0.04	2.4
A_51_P455997	NR_027652	Meg3	0.62	1.5	0.11	2.1	0.01	2.4
A_52_P516409	NM_053185	Col4a6	0.51	1.6	0.32	1.7	0.01	2.4
A_55_P2167803	NM_001014423	Abi3bp	0.76	1.4	0.23	1.8	0.04	2.3
A_55_P2261772	AK082735	C230098O21Rik	0.27	1.9	0.28	1.7	0.04	2.3
A_55_P2131766	NM_023665	D4Wsu53e	0.95	-1.2	0.63	1.4	0.03	2.3
A_55_P1969615	NM_012040	Pnck	0.86	1.3	0.37	1.6	0.02	2.3
A_55_P2011290	NM_011856	Odz2	0.35	1.8	0.14	2.0	0.02	2.3
A_55_P1958532	NM_021877	Hr	0.10	2.1	0.05	2.2	0.01	2.3
A_55_P2023314	NM_001159344	Casz1	0.23	1.8	0.11	1.9	0.00	2.3
A_51_P365516	NM_009258	Spink3	0.89	1.2	0.76	1.2	0.00	2.3

A_55_P1997105	NM_172670	Gyltl1b	0.21	1.7	0.02	2.0	0.00	2.3
A_30_P01018472			0.40	1.7	0.38	1.6	0.04	2.2
A_55_P2075080	NM_001029988	Fat2	0.70	1.5	0.61	1.4	0.04	2.2
A_51_P206405	NM_001081306	Ptprz1	0.45	1.7	0.26	1.7	0.04	2.2
A_51_P342567	NM_031185	Akap12	0.23	1.9	0.50	1.4	0.02	2.2
A_55_P2181251	NM_001001982	A430105I19Rik	0.26	1.7	0.15	1.7	0.00	2.2
A_55_P1956659	AK045799		0.53	1.5	0.19	1.7	0.00	2.2
A_30_P01032357			0.42	1.6	0.18	1.7	0.00	2.2
A_55_P2202524	AK034200	9330162012Rik	0.55	1.6	0.22	1.7	0.04	2.1
A_52_P686785	NM_053247	Lyve1	0.30	1.8	0.43	1.6	0.04	2.1
A_51_P368210	NM_145981	Phyhip	0.30	1.8	0.37	1.6	0.04	2.1
A_66_P105032	NM_001145034	Gm13889	0.28	1.7	0.64	1.3	0.03	2.1
A_55_P2059765	AK087349		0.38	1.6	0.21	1.7	0.02	2.1
A_55_P2030282	NM_001029930	ENSMUSG0000006879<br>0	0.72	1.4	0.46	1.5	0.01	2.1
A_55_P2031871	NM_146030	Plekhh3	0.29	1.5	0.12	1.6	0.00	2.1
A_51_P237752	NM_008986	Ptrf	0.44	1.6	0.25	1.6	0.00	2.1
A_30_P01021631			0.95	1.1	0.02	2.1	0.00	2.1
A_30_P01024344			0.76	1.4	0.33	1.6	0.04	2.0
A_30_P01024669			0.84	1.3	0.25	1.7	0.04	2.0
A_55_P1989296	NM_031176	Tnxb	0.64	1.5	0.25	1.6	0.04	2.0
A_55_P2064321	NM_001081171	Lama5	0.31	1.7	0.12	2.0	0.04	2.0
A_55_P2005470	NM_029568	Mfap4	0.12	2.0	0.18	1.7	0.03	2.0
A_55_P2053374	NM_178362	Sorbs1	0.26	1.8	0.46	1.4	0.03	2.0
A_55_P2067712	BC034076		0.29	1.6	0.15	1.6	0.02	2.0
A_52_P52849	NM_018867	Cpxm2	0.72	1.5	0.49	1.5	0.02	2.0
A_30_P01023251			0.89	1.2	0.02	2.1	0.01	2.0
A_52_P367520	NM_199465	Nexn	0.51	1.5	0.42	1.4	0.01	2.0
A_55_P1997106	NM_172670	Gyltl1b	0.57	1.4	0.11	1.8	0.01	2.0
A_55_P1956502	NM_021510	Hnrnph1	0.28	1.5	0.04	1.8	0.00	2.0
A_51_P296036	NM_144847	Nrbp2	0.52	1.5	0.16	1.7	0.03	1.9
A_55_P2150343	NM_001037298	Fam38a	0.26	1.7	0.21	1.6	0.03	1.9
A_55_P2222870	NM_139300	Mylk	0.52	1.5	0.32	1.5	0.03	1.9
A_55_P1979728	NM_009716	Atf4	0.72	1.4	0.06	2.0	0.03	1.9
A_51_P500344	NM_145569	Mat2a	0.66	1.3	0.00	1.9	0.00	1.9
A_55_P2000409	NM_001002786	Rab44	0.26	1.5	0.12	1.6	0.00	1.9
A_55_P2157872	NM_001079695	Sfrs5	0.71	1.3	0.10	1.6	0.00	1.9
A_51_P114826	NM_019707	Cdh13	0.65	1.4	0.37	1.5	0.04	1.8
A_55_P2008061	NM_019923	Itpr2	0.83	1.3	0.27	1.5	0.04	1.8
A_55_P2022364	NM_008750	Nxn	0.41	1.5	0.42	1.4	0.04	1.8
A_55_P2004797	NM_001004468	Tacc2	0.30	1.6	0.20	1.6	0.03	1.8
A_30_P01020357			0.78	1.3	0.37	1.5	0.03	1.8
A_55_P2083694	NM_033615	Adam33	0.89	1.2	0.79	1.2	0.03	1.8
A_52_P19016	NM_016680	Sfrs16	0.73	1.3	0.20	1.5	0.02	1.8
A_66_P126425	NM_011508	Eif1	0.62	1.3	0.02	1.9	0.02	1.8
A_52_P445387	NM_007714	Clk4	0.96	1.1	0.10	1.7	0.02	1.8
A_55_P2319035	BC051535	AW011956	0.32	1.5	0.14	1.6	0.02	1.8
A_55_P1983036	NM_172605	Tdrd3	0.48	1.4	0.16	1.5	0.01	1.8
A_55_P1970636	NM_133952	Unc45a	0.27	1.6	0.38	1.4	0.01	1.8

A_55_P1985788	NM_198092	Usp2	0.85	1.3	0.73	1.2	0.01	1.8
A_55_P1957219	NM_008972	Ptma	0.28	1.5	0.04	1.8	0.01	1.8
A_66_P127567	NM_016813	Nxf1	0.61	1.3	0.00	1.7	0.00	1.8
A_55_P1966664	NM_172587	Cdc14b	0.38	1.3	0.15	1.4	0.00	1.8
A_51_P520966	NM_015790	Icosl	0.69	1.3	0.00	1.9	0.00	1.8
A_55_P1986306	NM_181470	Ltv1	0.00	1.5	0.00	1.5	0.00	1.7
A_52_P307739	NM_011443	Sox2	0.72	1.3	0.31	1.4	0.04	1.7
A_52_P494380	NM_199011	Dgkq	0.59	1.4	0.38	1.4	0.04	1.7
A_51_P305547	NM_011415	Snai2	0.41	1.4	0.75	1.2	0.03	1.7
A_55_P2171008	NM_007714	Clk4	0.84	1.2	0.04	1.6	0.03	1.7
A_55_P1988872	AK165756		0.51	1.4	0.26	1.4	0.03	1.7
A_51_P486217	NM_175638	Wnk4	0.27	1.5	0.37	1.4	0.03	1.7
A_52_P348250	NM_001164493	Klhl29	0.46	1.4	0.76	1.2	0.03	1.7
A_55_P2183750	NM_008679	Ncoa3	0.55	1.4	0.25	1.5	0.03	1.7
A_55_P1996578	NM_008010	Fgfr3	0.89	1.2	0.68	1.2	0.03	1.7
A_55_P1958275	NM_016707	Bcl11a	0.10	1.7	0.00	1.8	0.03	1.7
A_55_P2115442	NM_053109	Clec2d	0.98	1.0	0.11	1.6	0.02	1.7
A_55_P2040815	NM_153545	Lrrc45	0.86	1.2	0.15	1.5	0.02	1.7
A_30_P01033124			0.12	1.8	0.07	1.8	0.02	1.7
A_51_P242414	NM_177461	Micall1	0.48	1.4	0.58	1.2	0.02	1.7
A_55_P1968928	NM_027185	Def6	0.32	1.5	0.56	1.3	0.01	1.7
A_55_P2081123	NM_018873	Srcin1	0.52	1.3	0.11	1.5	0.01	1.7
A_51_P295420	NM_145931	Zc3h7a	0.88	1.2	0.11	1.5	0.01	1.7
A_51_P455807	NM_133838	Ehd4	0.62	1.3	0.17	1.5	0.01	1.7
A_30_P01025839			0.15	1.5	0.00	1.6	0.00	1.7
A_55_P1968143	NM_027349	Rbm25	0.70	1.2	0.05	1.5	0.00	1.7
A_55_P1979893	NM_017376	Tef	0.83	1.2	0.07	1.6	0.00	1.7
A_51_P386304	NM_207678	Ccnl2	0.95	1.1	0.13	1.6	0.00	1.7
A_55_P1962906	NM_001146060	Als2cl	0.61	1.2	0.08	1.5	0.00	1.7
A_51_P342906	NM_009164	Sh3bp1	0.26	1.5	0.37	1.3	0.00	1.7
A_55_P2114218	NM_138590	Zcchc7	0.89	1.1	0.00	1.6	0.00	1.7
A_51_P231687	NM_011879	Ik	0.26	1.4	0.02	1.6	0.00	1.7
A_55_P1953459	NM_009534	Yap1	0.65	1.3	0.45	1.3	0.04	1.6
A_66_P118759	NM_001029979	Safb2	0.75	1.2	0.19	1.4	0.04	1.6
A_55_P2009783	NM_026377	6330577E15Rik	0.33	1.4	0.20	1.4	0.04	1.6
A_55_P2373852	AK009987	2310058N22Rik	0.91	1.1	0.46	1.3	0.04	1.6
A_55_P2027969	NM_010860	Myl6	0.26	1.5	0.15	1.5	0.03	1.6
A_55_P2056774	NM_025330	Hsd17b14	0.26	1.5	0.56	1.2	0.03	1.6
A_55_P1969166	NM_011814	Fxr2	0.56	1.3	0.22	1.4	0.03	1.6
A_55_P2045946	NM_183137	2410002I01Rik	0.75	1.2	0.30	1.4	0.03	1.6
A_55_P1973402	NM_172562	Tada2a	0.61	1.3	0.19	1.4	0.03	1.6
A_51_P240986	NM_198604	Plekhg6	0.24	1.5	0.18	1.5	0.03	1.6
A_51_P133792	NM_175097	Prickle3	0.63	1.3	0.20	1.5	0.02	1.6
A_51_P467410	NM_008722	Npm1	0.29	1.4	0.12	1.5	0.02	1.6
A_55_P2124228	NM_028283	Uaca	0.29	1.4	0.12	1.5	0.02	1.6
A_52_P665202			0.17	1.6	0.12	1.5	0.02	1.6
A_55_P1990573	NM_009224	Snrnp70	0.87	1.2	0.20	1.4	0.02	1.6
A_51_P279437	NM_029662	Mfsd2a	0.40	1.3	0.10	1.5	0.02	1.6
A_51_P130427	NM_009439	Psmd3	0.79	1.2	0.19	1.4	0.02	1.6

A_51_P296100	NM_145999	Rhot2	0.37	1.4	0.11	1.5	0.02	1.6
A_55_P2071097	XM_001472762	Gm9774	0.24	1.5	0.11	1.5	0.02	1.6
A_55_P2046378	NM_145941	Eif4g1	0.29	1.4	0.11	1.5	0.01	1.6
A_55_P1994204	NM_177680	Ythdc1	0.48	1.3	0.00	1.7	0.01	1.6
A_51_P183995	NM_019458	Paf1	0.57	1.3	0.17	1.4	0.01	1.6
A_55_P2001203	NM_001039520	Dnm2	0.15	1.5	0.04	1.7	0.01	1.6
A_52_P360515	NM_017392	Celsr2	0.35	1.4	0.02	1.6	0.01	1.6
A_51_P498023	NM_023912	Scyl1	0.35	1.3	0.15	1.4	0.00	1.6
A_51_P123047	NM_019710	Smc1a	0.29	1.3	0.00	1.5	0.00	1.6
A_51_P104933	NM_145520	Trub2	0.34	1.2	0.00	1.5	0.00	1.6
A_30_P01017507			0.33	1.2	0.07	1.4	0.00	1.6
A_55_P2145227	NM_032008	Slmap	0.22	1.5	0.15	1.4	0.00	1.6
A_65_P03022	NM_172587	Cdc14b	0.44	1.3	0.48	1.2	0.00	1.6
A_55_P1980651	NM_001081359	Ubr5	0.34	1.3	0.07	1.5	0.00	1.6
A_30_P01021814			0.26	1.3	0.06	1.4	0.00	1.6
A_55_P1980180	NM_145556	Tardbp	0.21	1.4	0.17	1.4	0.00	1.6
A_51_P169920	NM_021303	Noc2l	0.62	1.2	0.32	1.3	0.00	1.6
A_51_P246317	NM_008630	Mt2	0.41	1.5	0.02	2.2	0.17	1.6
A_55_P2118674	NM_021327	Tnip1	0.72	1.3	0.00	2.1	0.06	1.6
A_55_P2101585	NM_010016	Cd55	0.44	1.3	0.42	1.3	0.04	1.5
A_55_P2034003	NM_207239	Gtf3c1	0.62	1.2	0.23	1.3	0.04	1.5
A_55_P2131875	NM_178627	Poldip3	0.39	1.4	0.10	1.5	0.04	1.5
A_55_P1993522	BC094673		0.70	1.2	0.48	1.2	0.04	1.5
A_55_P2062418	NM_016920	Atp6v0a1	0.19	1.4	0.15	1.4	0.04	1.5
A_55_P2356840	BC029726	1600020E01Rik	0.90	1.1	0.53	1.2	0.04	1.5
A_30_P01017746			0.67	1.2	0.34	1.3	0.04	1.5
A_55_P2054352	NM_029169	Rbm6	0.88	1.1	0.12	1.4	0.04	1.5
A_55_P2079619	NM_172448	Rnf43	0.30	1.4	0.04	1.7	0.04	1.5
A_51_P148355	NM_175433	Zfp710	0.33	1.4	0.04	1.6	0.04	1.5
A_55_P1995652	NM_207234	Rexo4	0.52	1.3	0.18	1.4	0.03	1.5
A_52_P313080	NM_010448	Hnrnpab	0.26	1.3	0.15	1.4	0.03	1.5
A_55_P2179834	NM_145596	Gatad2a	0.90	1.1	0.80	1.1	0.03	1.5
A_66_P103301	NM_148948	Dicer1	0.86	1.1	0.17	1.4	0.03	1.5
A_51_P370286	NM_011869	Med24	0.31	1.4	0.14	1.4	0.03	1.5
A_55_P2016346	NM_018749	Eif3d	0.53	1.3	0.02	1.5	0.03	1.5
A_55_P2169714	NM_172719	Gcn1l1	0.35	1.4	0.13	1.5	0.03	1.5
A_55_P2062622	NM_023144	Nono	0.37	1.3	0.15	1.4	0.03	1.5
A_55_P2386592	NM_001081122	Cep63	0.48	1.3	0.10	1.5	0.03	1.5
A_55_P1965391	NM_177771	Klhl18	0.12	1.5	0.50	1.2	0.03	1.5
A_55_P2034372	NM_172703	Eif4g3	0.84	1.2	0.13	1.4	0.03	1.5
A_55_P1997415	NM_177077	Exoc6b	0.24	1.4	0.29	1.3	0.02	1.5
A_55_P2064578	NM_008857	Prkci	0.26	1.4	0.17	1.4	0.02	1.5
A_55_P2086258	NM_028291	Pan3	0.75	1.2	0.23	1.3	0.02	1.5
A_55_P2041045	NM_134002	Csnk1g2	0.41	1.3	0.02	1.6	0.02	1.5
A_51_P258078	NM_138747	Nop2	0.34	1.3	0.18	1.3	0.02	1.5
A_55_P1981296	NM_001002929	Nup85	0.38	1.3	0.27	1.3	0.02	1.5
A_52_P88695	NM_016811	Dgka	0.12	1.5	0.21	1.3	0.02	1.5
A_55_P2015994	NM_013518	Fgf9	0.35	1.3	0.29	1.3	0.02	1.5
A_30_P01028695			0.30	1.3	0.40	1.2	0.02	1.5

A_55_P2078940	NM_198326	Nsfl1c	0.38	1.3	0.16	1.4	0.02	1.5
A_51_P202430	NM_001033313	Pdap1	0.29	1.3	0.13	1.4	0.02	1.5
A_55_P2141088			0.44	1.3	0.29	1.3	0.01	1.5
A_55_P1991802	NM_181417	Csrp2bp	0.79	1.2	0.10	1.4	0.01	1.5
A_55_P2069856	NM_177296	Tnpo3	0.41	1.3	0.15	1.3	0.01	1.5
A_51_P379443	NM_001025392	Bclaf1	0.77	1.2	0.07	1.4	0.01	1.5
A_55_P2000823	NM_133242	Rbm39	0.95	1.1	0.23	1.3	0.01	1.5
A_55_P2014635	NM_025534	Ccdc82	0.97	1.0	0.53	1.2	0.01	1.5
A_52_P611798	NM_007840	Ddx5	0.83	1.2	0.14	1.4	0.01	1.5
A_52_P357402	NM_019988	Mlst8	0.37	1.2	0.08	1.4	0.01	1.5
A_55_P2019814	NM_001163263	Rnf20	0.53	1.3	0.06	1.5	0.01	1.5
A_55_P2092909	NM_019713	Rassf1	0.68	1.1	0.00	1.4	0.00	1.5
A_55_P2146002	NM_172990	Pank4	0.78	1.2	0.35	1.2	0.00	1.5
A_55_P1956842	XM_001478894	Gm6506	0.16	1.5	0.15	1.4	0.00	1.5
A_66_P101600	NM_008482	Lamb1-1	0.72	1.2	0.15	1.3	0.00	1.5
A_55_P1990663	NM_007840	Ddx5	0.85	1.1	0.14	1.3	0.00	1.5
A_52_P278497	NM_201256	Eif4ebp3	0.51	1.2	0.11	1.4	0.00	1.5
A_55_P2315457	NM_207670	Gripap1	0.34	1.3	0.08	1.4	0.00	1.5
A_55_P2120615	NM_198113	Ssh3	0.52	1.2	0.04	1.4	0.00	1.5
A_55_P1990127	NM_020329	Dolpp1	0.64	1.2	0.10	1.4	0.00	1.5
A_55_P2005190	NM_026101	Herc4	0.79	1.2	0.16	1.3	0.00	1.5
A_51_P342271	NM_001035123	Setd6	0.34	1.2	0.00	1.5	0.00	1.5
A_52_P311104	NM_012047	Brd7	0.43	1.2	0.06	1.3	0.00	1.5
A_55_P2145804	NM_026531	Aen	0.21	1.4	0.17	1.4	0.00	1.5
A_66_P111660	NM_013602	Mt1	0.76	1.3	0.00	3.1	0.30	1.5
A_55_P2044045	NM_011732	Ybx1	0.37	1.4	0.00	1.9	0.06	1.5
A_55_P2031167	NM_010107	Efna1	0.78	1.2	0.04	1.7	0.06	1.5
A_55_P1992257	NM_007457	Ap1s1	0.57	1.2	0.04	1.5	0.04	1.4
A_30_P01019965			0.49	1.2	0.02	1.5	0.05	1.4
A_51_P167374	NM_026181	Gpatch1	0.51	1.2	0.02	1.5	0.05	1.4
A_55_P2028821	NM_001166430	Hnrnpf	0.26	1.3	0.00	1.5	0.01	1.4
A_30_P01023424			0.12	1.3	0.00	1.5	0.00	1.4
A_55_P2097478	NM_010266	Gda	0.88	1.2	0.04	1.7	0.46	1.3
A_55_P2048011	NM_172585	Larp4b	0.68	1.2	0.02	1.6	0.17	1.3
A_52_P35377	NM_018807	Plagl2	0.70	1.2	0.00	1.5	0.09	1.3
A_55_P2111399	NM_001129803	Pcp2	0.88	1.1	0.04	1.5	0.91	1.1
A_55_P2121846	XM_994545	Gm9000	0.88	-1.1	0.00	-1.5	0.71	-1.1
A_55_P1991718	NM_013552	Hmmr	0.99	1.0	0.00	-1.5	0.60	-1.1
A_55_P2176240	NM_172134	Pdxk	0.72	-1.2	0.04	-1.6	0.52	-1.2
A_55_P2092296	NM_176848	Fbxo2	0.93	-1.1	0.02	-1.7	0.68	-1.2
A_30_P01021428			0.99	1.0	0.00	-2.5	0.65	-1.2
A_51_P132718	XM_896611	2310010J17Rik	0.00	-1.7	0.69	-1.2	0.24	-1.3
A_52_P302041	XM_001472266	LOC100044517	0.63	-1.2	0.00	-1.5	0.05	-1.3
A_30_P01025354			0.23	-1.3	0.00	-1.5	0.07	-1.3
A_66_P104390	NM_019718	Arl3	0.34	-1.3	0.00	-1.6	0.07	-1.3
A_51_P483557	NM_009458	Ube2b	0.26	-1.3	0.05	-1.5	0.04	-1.4
A_55_P1954400			0.52	-1.3	0.04	-1.6	0.08	-1.4
A_55_P2021109	NM_010500	Ier5	0.94	-1.1	0.04	-1.6	0.17	-1.4
A_55_P2009206	NM_133354	Sumo2	0.65	-1.3	0.02	-1.7	0.08	-1.4

A_55_P2073482	XM_001479274	Gm13430	0.85	-1.2	0.00	-1.6	0.04	-1.5
A_55_P1980721	XM_001473516	Gm16399	0.21	-1.4	0.13	-1.4	0.04	-1.5
A_52_P119117	NM_001163431	1810006K21Rik	0.34	-1.3	0.13	-1.4	0.04	-1.5
A_55_P2141311	NM_001012400	AU022252	0.74	-1.2	0.22	-1.4	0.04	-1.5
A_55_P2144391			0.24	-1.4	0.75	-1.1	0.04	-1.5
A_55_P2045114	NM_027457	5730437N04Rik	0.26	-1.5	0.12	-1.5	0.04	-1.5
A_55_P2182483	NM_010581	Cd47	0.71	-1.2	0.25	-1.4	0.04	-1.5
A_55_P2051962	NM_024171	Sec61b	0.39	-1.3	0.30	-1.3	0.04	-1.5
A_55_P2176753	XM_917662	LOC640614	0.70	-1.2	0.10	-1.5	0.04	-1.5
A_52_P575771	NM_026182	Mtfr1	0.76	-1.2	0.48	-1.2	0.03	-1.5
A_55_P1972128	NM_010761	Ccndbp1	0.86	-1.2	0.54	-1.2	0.03	-1.5
A_55_P2056348	XM_885168	Gm6181	0.26	-1.4	0.29	-1.3	0.03	-1.5
A_55_P2154536	NM_001159626	Hagh	0.51	-1.3	0.15	-1.4	0.03	-1.5
A_51_P105408	NM_024178	Alg14	0.94	-1.1	0.60	-1.2	0.03	-1.5
A_52_P572284	NM_008640	Laptm4a	0.79	-1.2	0.12	-1.5	0.03	-1.5
A_55_P2090406	NM_010376	H13	0.85	-1.2	0.51	-1.2	0.03	-1.5
A_51_P210956	NM_011693	Vcam1	0.35	-1.3	0.21	-1.3	0.02	-1.5
A_51_P357422	NM_025556	2410022L05Rik	0.64	-1.2	0.31	-1.3	0.02	-1.5
A_51_P428505	NM_025327	Krtcap2	0.56	-1.3	0.15	-1.4	0.02	-1.5
A_55_P1960281	NM_025426	Med7	0.70	-1.2	0.17	-1.3	0.02	-1.5
A_52_P543430			0.84	-1.2	0.35	-1.3	0.02	-1.5
A_51_P252199	NM_146234	Mmgt1	0.89	-1.1	0.27	-1.3	0.01	-1.5
A_55_P2168781			0.81	-1.2	0.17	-1.4	0.01	-1.5
A_51_P496845	NM_007604	Capza2	0.79	-1.2	0.12	-1.5	0.01	-1.5
A_51_P252410	NM_021538	Cope	0.24	-1.3	0.13	-1.3	0.01	-1.5
A_51_P401987	NM_019432	Tmem37	0.26	-1.4	0.13	-1.4	0.00	-1.5
A_51_P314763	NM_011231	Rabggtb	0.68	-1.2	0.13	-1.4	0.00	-1.5
A_55_P2055232	NM_011341	Sdf4	0.33	-1.3	0.15	-1.3	0.00	-1.5
A_55_P2127683	NM_027500	4933434E20Rik	0.88	-1.1	0.02	-1.4	0.00	-1.5
A_52_P229728	NM_009861	Cdc42	0.43	-1.3	0.10	-1.5	0.00	-1.5
A_55_P2091486	NM_026827	Tmem219	0.30	-1.3	0.07	-1.4	0.00	-1.5
A_65_P01783	NM_025895	Med28	0.76	-1.2	0.00	-1.7	0.08	-1.5
A_55_P1995622	NM_025624	Pomp	0.43	-1.4	0.00	-1.9	0.06	-1.5
A_51_P396708	NM_025315	Med21	0.76	-1.2	0.17	-1.5	0.04	-1.6
A_51_P504588	NM_027194	Tm2d2	0.66	-1.3	0.51	-1.3	0.04	-1.6
A_55_P2067682	NM_013795	Atp5l	0.23	-1.6	0.15	-1.6	0.04	-1.6
A_51_P483311	NM_183170	Mpv17l2	0.56	-1.3	0.57	-1.2	0.04	-1.6
A_52_P89683	XM_001474667	Gm14470	0.85	-1.2	0.08	-1.6	0.03	-1.6
A_55_P2127425	NM_025575	Sys1	0.43	-1.3	0.16	-1.4	0.02	-1.6
A_66_P129484	NM_026211	Tmed9	0.98	-1.0	0.14	-1.5	0.02	-1.6
A_55_P2108334	NM_138596	Med10	0.44	-1.3	0.24	-1.4	0.02	-1.6
A_30_P01033314			0.70	-1.2	0.07	-1.5	0.01	-1.6
A_30_P01028065			0.38	-1.4	0.10	-1.5	0.00	-1.6
A_55_P2145557			0.23	-1.4	0.05	-1.6	0.00	-1.6
A_51_P221014	NM_010581	Cd47	0.10	-1.4	0.02	-1.5	0.00	-1.6
A_30_P01025197			0.34	-1.4	0.02	-1.7	0.06	-1.6
A_55_P1969522	XM_001472002	Smt3h2-ps2	0.87	-1.2	0.02	-1.8	0.06	-1.6
A_55_P1969523	NM_133354	Sumo2	0.71	-1.3	0.00	-1.9	0.09	-1.6
A_52_P542970			0.36	-1.4	0.02	-2.0	0.06	-1.6

A_51_P336874	NM_025272	Atp6v0e	0.81	-1.3	0.40	-1.4	0.04	-1.7
A_55_P2175942			0.30	-1.5	0.29	-1.4	0.04	-1.7
A_51_P323610	NM_024439	H47	0.75	-1.3	0.40	-1.4	0.04	-1.7
A_55_P2029630	NM_011900	Mpdu1	0.89	-1.2	0.19	-1.5	0.02	-1.7
A_55_P2106175	NM_008102	Gch1	0.38	-1.4	0.67	-1.2	0.02	-1.7
A_55_P2175245	NM_030254	Tusc3	0.39	-1.4	0.12	-1.5	0.01	-1.7
A_55_P2155593			0.66	-1.3	0.07	-1.8	0.01	-1.7
A_55_P2016681	NM_025538	Alkbh7	0.51	-1.4	0.16	-1.5	0.01	-1.7
A_30_P01022383			0.78	-1.2	0.20	-1.4	0.01	-1.7
A_52_P210338	NM_001033305	Ndufb6	0.26	-1.4	0.12	-1.5	0.00	-1.7
A_55_P1999842	XM_001477263	Gm3386	0.16	-1.6	0.10	-1.6	0.00	-1.7
A_55_P1974467	NM_001033305	Ndufb6	0.29	-1.4	0.15	-1.4	0.00	-1.7
A_55_P2043723	NM_010261	Rabac1	0.26	-1.4	0.05	-1.5	0.00	-1.7
A_52_P375873	NM_012058	Srp9	0.43	-1.4	0.16	-1.6	0.02	-1.8
A_55_P2115235	NM_199195	Bckdhb	0.49	-1.4	0.63	-1.2	0.02	-1.8
A_55_P1980931			0.33	-1.5	0.02	-1.9	0.01	-1.8
A_52_P191975	NM_001003934	Rtn3	0.77	-1.3	0.02	-1.9	0.00	-1.8
A_51_P304170	NM_023386	Rtp4	0.00	-1.9	0.02	-1.8	0.00	-1.9
A_55_P2098802	NM_025352	Uqcrq	0.42	-1.5	0.39	-1.4	0.03	-1.9
A_51_P314264	NM_025448	Ssr2	0.73	-1.4	0.28	-1.6	0.03	-1.9
A_55_P2099750			0.33	-1.5	0.30	-1.4	0.01	-1.9
A_52_P401311	XM_484885	Gm5529	0.50	-1.5	0.42	-1.5	0.02	-2.0

3.1.6 DBahA Forestomach. Significant probe list. List of all significantly differentially expressed probes in at least 1 treatment group (FDR P ≤ 0.05, fold change ± 1.5) in response to sub-chronic oral exposure to 6.25, 12.5, and 25 mg/kg-bw/day dibenz(ah)anthracene in the forestomach. The list is sorted from highest to lowest fold change in the 25 mg/kg-bw/day treatment group.

			6.25 mg/kg-bw/day		12.5 mg/kg-bw/day		25 mg/kg-bw/day	
Agilent Probe	Accession Number	Gene Symbol	*FDR P value*	*Fold change*	*FDR P value*	*Fold change*	*FDR P value*	*Fold change*
A_55_P2032079	NM_016974	Dbp	0.78	2.3	0.17	3.7	0.00	5.3
A_55_P2032081	NM_016974	Dbp	0.78	2.2	0.17	3.2	0.00	4.8
A_55_P2148534	BC096461	Nr1d2	0.76	1.7	0.00	2.6	0.00	2.9
A_55_P1979893	NM_017376	Tef	1.00	1.4	0.52	1.8	0.00	2.4
A_52_P303891	NM_011584	Nr1d2	1.00	1.3	0.32	1.8	0.00	2.0
A_30_P01025159			0.67	1.3	0.72	1.3	0.00	1.7
A_55_P2167530	BU610818	Scube3	0.48	-1.2	0.00	-1.5	0.00	-1.4
A_30_P01030803			1.00	-1.1	0.00	-1.4	0.00	-1.5
A_55_P2057283	AY170502		1.00	-1.1	0.08	-1.5	0.00	-1.5
A_51_P383032	NM_010819	Clec4d	1.00	-1.2	0.00	-1.6	0.05	-1.5
A_55_P1969392	NM_026594	Rpl39l	1.00	-1.2	0.50	-1.3	0.00	-1.6

3.1.7 IP Forestomach. Significant probe list. List of all significantly differentially expressed probes in at least 1 treatment group (FDR P ≤ 0.05, fold change ± 1.5) in response to sub-chronic oral exposure to 12.5, 25, and 50 mg/kg-bw/day indeno(123,cd)pyrene in the forestomach. The list is sorted from highest to lowest fold change in the 50 mg/kg-bw/day treatment group.

			12.5 mg/kg-bw/day	25 mg/kg-bw/day	50 mg/kg-bw/day

Agilent Probe	Accession Number	Gene Symbol	*FDR P value*	*Fold change*	*FDR P value*	*Fold change*	*FDR P value*	*Fold change*
A_52_P533280	NM_013637	Prm1	0.78	2.1	0.44	4.3	0.00	9.0
A_51_P138705	NM_008933	Prm2	0.73	2.1	0.44	3.6	0.00	7.6
A_51_P242403	NM_013580	Ldhc	0.80	1.7	0.50	2.9	0.00	6.1
A_30_P0102746 4			0.87	1.2	0.73	1.4	0.00	1.9
A_55_P2157872	NM_00107969 5	Sfrs5	0.97	1.0	0.71	1.3	0.00	1.7
A_66_P106760	NM_153397	Adam32	0.99	1.0	0.77	1.2	0.00	1.5
A_55_P2096204	NM_172262	Kdm1b	0.00	-1.5	0.00	-1.5	0.00	-1.5
A_55_P2058957	NM_009306	Syt1	0.00	-1.8	0.20	-1.7	0.34	-1.5
A_55_P2001494	NM_013598	Kitl	0.93	-1.1	0.44	-1.6	0.00	-2.0
A_55_P2087984	NM_00116467 1	Dnaja1	0.48	-1.6	0.32	-1.9	0.00	-2.3

3.2 LIVER

3.2.1 BaA Liver. Significant probe list. List of all significantly differentially expressed probes in at least 1 treatment group (FDR P ≤ 0.05, fold change ± 1.5) in response to sub-chronic oral exposure to 20, 40, and 80 mg/kg-bw/day benz(a)anthracene in the liver. The list is sorted from highest to lowest fold change in the 80 mg/kg-bw/day treatment group.

			**20 mg/kg-bw/day**		**40 mg/kg-bw/day**		**80 mg/kg-bw/day**	
Agilent Probe	Accession Number	Gene Symbol	*FDR P value*	*Fold change*	*FDR P value*	*Fold change*	*FDR P value*	*Fold change*
A_55_P1985788	NM_198092	Usp2	1.00	-1.0	0.75	1.4	0.02	3.9
A_66_P128293	NM_177391	Fam109b	1.00	-1.0	0.78	-1.2	0.00	3.2
A_55_P2170454	NM_008182	Gsta2	0.98	1.1	0.29	1.8	0.01	3.2
A_55_P2032081	NM_016974	Dbp	0.86	1.3	0.14	2.0	0.01	2.7
A_55_P1987439	NM_133779	Pigt	0.92	1.1	0.28	1.3	0.00	2.7
A_66_P112495	NM_001013390	Scn4b	0.95	-1.1	0.90	-1.1	0.00	2.7
A_51_P219483	NM_001024619	Tsku	0.96	-1.1	0.88	1.1	0.00	2.7
A_55_P2029106	NM_138313	Bmf	0.69	1.3	0.16	1.5	0.00	2.5
A_66_P136186	NM_009516	Wee1	0.97	1.1	0.39	1.5	0.01	2.5
A_55_P2162160	NM_009672	Anp32a	0.68	1.4	0.03	2.1	0.03	2.2
A_55_P2133632	NM_001081337	Sipa1l2	0.90	1.1	0.02	1.5	0.00	2.1
A_30_P01026009			0.98	1.0	0.99	1.0	0.00	2.0
A_55_P1983754	NM_025557	Pcp4l1	0.80	1.2	0.23	1.4	0.00	2.0
A_52_P318673	NM_009117	Saa1	0.00	3.4	0.87	1.2	0.25	2.0
A_51_P155747	NM_028776	Scyl3	0.78	1.1	0.29	1.2	0.00	2.0
A_55_P2002319	NM_027129	2310035K24Rik	0.72	1.3	0.25	1.5	0.04	1.9
A_55_P2077613	NM_153560	Fam102a	0.99	-1.0	0.80	1.1	0.02	1.8
A_55_P2144526	NM_001080381	Fam65b	0.38	1.3	0.01	1.6	0.00	1.8
A_55_P2018417	NM_001163645	Osbpl3	0.89	1.1	0.01	1.4	0.00	1.8
A_51_P240986	NM_198604	Plekhg6	0.51	1.3	0.61	1.2	0.00	1.8
A_55_P1994807	NM_011314	Saa2	0.00	3.4	0.30	1.7	0.29	1.8
A_30_P01024922			0.06	1.5	0.01	1.6	0.00	1.7
A_51_P386503	NM_026185	Abhd15	0.17	1.3	0.02	1.4	0.00	1.7

A_52_P373893	AK154578	Acvr1b	0.89	1.1	0.09	1.3	0.00	1.7
A_51_P308347	NM_172826	Dact2	0.37	1.2	0.07	1.3	0.00	1.7
A_55_P1967538	NM_015755	Hunk	0.31	1.4	0.99	1.0	0.03	1.7
A_51_P196844	NM_027881	Osbpl3	0.98	1.0	0.00	1.5	0.00	1.7
A_55_P1976351	NM_001042672	Prei4	0.60	1.4	0.02	2.0	0.17	1.7
A_55_P1987290	NR_004413	Rnu1b6	0.01	2.3	0.00	2.8	0.09	1.7
A_51_P502150	NM_012030	Slc9a3r1	0.27	1.3	0.11	1.4	0.00	1.7
A_51_P250807	NM_030176	Spata2L	0.97	1.0	0.32	1.3	0.01	1.7
A_30_P01019195			0.43	1.3	0.25	1.3	0.04	1.6
A_30_P01019132			0.36	1.3	0.64	1.2	0.04	1.6
A_30_P01031642			0.96	1.0	0.80	1.1	0.01	1.6
A_55_P2179726	NM_027279	2810422O20Rik	0.94	1.1	0.22	1.3	0.03	1.6
A_55_P2322709	AK014609	4633401B06Rik	0.85	1.1	0.15	1.4	0.04	1.6
A_55_P2029366	NM_025341	Abhd6	0.82	1.1	0.36	1.2	0.00	1.6
A_51_P164296	NM_021475	Adamdec1	0.01	1.7	0.11	1.4	0.02	1.6
A_55_P1960216	NM_009842	Cd151	0.79	1.1	0.75	1.1	0.00	1.6
A_51_P200667	NM_053155	Clmn	0.95	1.1	0.99	1.0	0.03	1.6
A_52_P217710	NM_008056	Fzd6	0.09	1.3	0.11	1.3	0.00	1.6
A_55_P1967539	NM_015755	Hunk	0.74	1.2	0.93	1.1	0.04	1.6
A_65_P10491	XM_001479477	Raph1	0.07	1.5	0.07	1.5	0.04	1.6
A_51_P191893	AK163489		0.97	1.0	0.27	1.3	0.04	1.5
A_30_P01018838			0.97	1.0	0.99	-1.0	0.03	1.5
A_30_P01017753			0.80	1.2	0.09	1.4	0.03	1.5
A_30_P01021665			0.87	1.1	0.18	1.3	0.03	1.5
A_55_P2135023			0.82	1.1	0.44	1.1	0.00	1.5
A_51_P494125	NM_007431	Alpl	0.69	-1.1	0.92	-1.0	0.00	1.5
A_52_P179178	NM_199028	Bend3	0.18	1.3	0.00	1.6	0.01	1.5
A_52_P748958	NM_007687	Cfl1	0.66	1.1	0.80	1.1	0.00	1.5
A_55_P2016540	NM_009963	Cry2	0.85	1.1	0.01	1.4	0.00	1.5
A_51_P161946	NM_175332	E130012A19Rik	0.84	1.1	0.37	1.2	0.04	1.5
A_51_P361220	NM_008055	Fzd4	0.80	1.1	0.11	1.4	0.04	1.5
A_55_P2095035	XM_129965	Gm1833	0.81	1.1	0.28	1.3	0.02	1.5
A_55_P1993708	NM_172814	Lrp12	0.90	1.1	0.05	1.3	0.00	1.5
A_52_P16563	NM_177727	Lsm14b	0.45	1.2	0.02	1.3	0.00	1.5
A_51_P369862	NM_178772	Nceh1	0.47	1.3	0.02	1.5	0.05	1.5
A_51_P430973	NM_027995	Paqr7	0.85	1.1	0.38	1.2	0.01	1.5
A_55_P2003096	NM_016915	Pla2g6	0.62	1.1	0.56	1.1	0.00	1.5
A_55_P2095342	NM_009024	Rara	0.02	1.4	0.00	1.4	0.00	1.5
A_55_P2141479	NR_004414	Rnu2	0.00	1.9	0.00	1.9	0.05	1.5
A_51_P300572	NM_009579	Slc30a1	0.31	1.3	0.16	1.3	0.03	1.5
A_52_P451834	NM_183262	Stk35	0.73	1.2	0.62	1.2	0.04	1.5
A_55_P1981090	NM_026909	Thap7	0.34	1.2	0.00	1.4	0.00	1.5
A_51_P273508	NM_025985	Ube2g1	0.85	1.1	0.60	1.1	0.01	1.5
A_51_P148355	NM_175433	Zfp710	0.76	1.1	0.54	1.1	0.00	1.5
A_55_P2286650	AK032850	6720462K09Rik	0.15	1.3	0.00	1.5	0.00	1.4
A_55_P1985623	NM_029600	Abcc3	0.15	1.4	0.03	1.5	0.17	1.4
A_52_P661044	NM_009914	Ccr3	0.04	1.5	0.92	-1.1	0.12	1.4
A_51_P314679	NM_001039521	Rrn3	0.84	1.1	0.03	1.5	0.13	1.4
A_55_P2157872	NM_001079695	Sfrs5	0.04	-1.5	0.59	-1.2	0.17	1.4

A_66_P110798	AK145507		0.27	1.3	0.02	1.5	0.19	1.3
A_55_P2042813	NM_001039647	EG634650	0.47	-1.8	0.00	-3.4	0.80	1.3
A_30_P01025874			0.43	1.3	0.02	1.6	0.62	1.2
A_30_P01032527			0.18	1.4	0.01	1.6	0.70	1.2
A_30_P01032549			0.95	1.1	0.01	-1.9	0.56	1.2
A_55_P2210213	AK028644	4732423E21Rik	0.15	1.2	0.00	1.5	0.13	1.2
A_51_P299149	NM_145451	Gpx6	0.88	1.1	0.02	1.5	0.65	1.2
A_55_P1974695	AK083375		0.01	1.6	0.20	1.3	0.90	1.1
A_30_P01026106			0.47	1.3	0.02	1.5	0.75	1.1
A_55_P2067221	AK147351		0.62	1.2	0.02	1.5	0.79	1.1
A_51_P234544	NM_018745	Azin1	0.77	1.1	0.00	1.5	0.69	1.1
A_55_P2270412	AK049070	C230096K16Rik	0.85	1.2	0.02	1.6	0.75	1.1
A_55_P2035932	NM_008008	Fgf7	0.97	1.0	0.04	1.5	0.79	1.1
A_55_P2071601	NM_011144	Ppara	0.42	1.3	0.01	1.5	0.80	1.1
A_55_P1969002	NM_011535	Tbx3	0.06	1.6	0.01	1.9	0.82	1.1
A_55_P2131379	M60419	Ybx1	0.99	-1.0	0.03	1.9	0.93	1.1
A_52_P213483	AF045501		0.99	-1.0	0.00	1.6	0.94	1.0
A_30_P01021428			0.95	-1.1	0.00	-2.2	1.00	1.0
A_55_P2075230	XM_001474042	BC051226	0.22	-1.4	0.02	-1.6	0.99	1.0
A_55_P2223851	AK036131		0.14	1.6	0.03	1.7	0.98	-1.0
A_55_P2224431	AK018162	6330412A17Rik	0.00	1.6	0.83	1.1	0.99	-1.0
A_55_P2278775	AK050221	9130016M20Rik	0.58	-1.2	0.01	-1.5	0.99	-1.0
A_55_P2129373	NM_001013767	Capn11	0.86	1.1	0.01	1.6	0.94	-1.0
A_55_P2046877	NM_008239	Foxq1	0.88	-1.2	0.03	-2.2	0.97	-1.0
A_55_P2043509	NM_080462	Hnmt	0.89	-1.1	0.04	-1.7	0.95	-1.0
A_30_P01022977			0.00	-1.5	0.08	-1.3	0.59	-1.1
A_30_P01025896			0.15	-1.3	0.00	-1.5	0.57	-1.1
A_55_P2016064			0.21	-1.6	0.03	-1.8	0.83	-1.1
A_55_P1984881	NM_001162980	1700024P16Rik	0.59	-1.2	0.00	-1.6	0.68	-1.1
A_55_P2116689	NM_001162980	1700024P16Rik	0.66	-1.2	0.00	-1.6	0.79	-1.1
A_55_P2345116	AK051328	1700109K24Rik	0.40	1.3	0.03	1.5	0.91	-1.1
A_51_P120066	NR_033222	9330151L19Rik	0.01	-1.6	0.01	-1.5	0.86	-1.1
A_55_P2043367	NM_173786	Apol9a	0.66	-1.2	0.00	-1.6	0.76	-1.1
A_66_P110633	NM_001168660	Apol9b	0.73	-1.2	0.01	-1.5	0.85	-1.1
A_51_P212038	NM_133764	Atp6v0e2	0.83	-1.2	0.04	-1.9	0.84	-1.1
A_65_P16680	NM_019835	B4galt5	0.43	-1.3	0.04	-1.5	0.77	-1.1
A_51_P118779	NM_172725	C330006K01Rik	0.05	-1.5	0.01	-1.6	0.69	-1.1
A_55_P1990663	NM_007840	Ddx5	0.02	-1.5	0.03	-1.5	0.91	-1.1
A_66_P128931	XM_001479188	LOC100047937	0.66	-1.3	0.01	-1.7	0.87	-1.1
A_51_P310949	NM_008911	Ppox	0.34	-1.3	0.00	-1.6	0.75	-1.1
A_51_P216496	NM_009364	Tfpi2	0.77	-1.2	0.01	-1.7	0.70	-1.1
A_52_P68477	NM_172763	Zfp809	0.41	-1.4	0.03	-1.7	0.80	-1.1
A_30_P01027752			0.02	-1.5	0.00	-1.7	0.30	-1.2
A_30_P01028020			0.90	1.1	0.00	1.7	0.65	-1.2
A_55_P2041823	AK076101		0.38	-1.2	0.00	-1.5	0.18	-1.2
A_30_P01028097			0.97	-1.0	0.01	-1.7	0.58	-1.2
A_55_P2020306	NM_173765	Aasdh	0.28	-1.3	0.01	-1.5	0.48	-1.2
A_55_P2100290	NM_007416	Adra1b	0.30	-1.3	0.00	-1.6	0.40	-1.2
A_55_P1957922	NM_027560	Arrdc2	0.00	-1.5	0.00	-1.5	0.42	-1.2

A_55_P2088440	NM_027560	Arrdc2	0.04	-1.4	0.00	-1.6	0.50	-1.2
A_55_P1959953	NM_183162	BC006779	0.27	-1.3	0.00	-1.5	0.28	-1.2
A_51_P137336	NM_009864	Cdh1	0.69	-1.3	0.03	-1.7	0.69	-1.2
A_55_P2115442	NM_053109	Clec2d	0.00	-2.2	0.03	-1.6	0.52	-1.2
A_51_P382928	NM_145529	Cstf3	0.30	-1.2	0.00	-1.5	0.17	-1.2
A_52_P27122	NM_153177	Eif2c4	0.45	-1.3	0.01	-1.6	0.35	-1.2
A_51_P152155	NM_029626	Glt8d1	0.02	-1.4	0.01	-1.5	0.22	-1.2
A_55_P2139087	NM_001013820	Gm5631	0.03	-1.7	0.25	-1.4	0.66	-1.2
A_55_P2327958	NM_001162945	Mtx3	0.04	-1.5	0.02	-1.4	0.55	-1.2
A_55_P1990573	NM_009224	Snrnp70	0.04	-1.3	0.00	-1.5	0.30	-1.2
A_55_P2071286	NM_181820	Tmc4	0.00	-1.5	0.00	-1.6	0.31	-1.2
A_55_P1963344	NM_026573	Upf3b	0.15	-1.4	0.01	-1.5	0.43	-1.2
A_51_P487073	NM_138684	Wfdc12	0.57	-1.3	0.04	-1.6	0.68	-1.2
A_55_P2069525	NM_177462	Zmym6	0.00	-1.6	0.01	-1.5	0.50	-1.2
A_30_P01026904			0.03	-1.5	0.01	-1.5	0.18	-1.3
A_30_P01027782			0.00	-1.5	0.00	-1.5	0.09	-1.3
A_30_P01033133			0.00	-1.5	0.01	-1.4	0.03	-1.3
A_30_P01018953			0.02	-1.6	0.00	-1.7	0.22	-1.3
A_30_P01021218			0.56	-1.2	0.01	-1.5	0.13	-1.3
A_55_P2075258	AK015504		0.23	-1.4	0.01	-1.6	0.26	-1.3
A_30_P01022571			0.98	-1.0	0.02	-1.8	0.42	-1.3
A_30_P01033600			0.60	-1.5	0.01	-2.5	0.67	-1.3
A_52_P176619	NM_001142744	2610110G12Rik	0.05	-1.4	0.01	-1.5	0.06	-1.3
A_51_P225832	NM_028314	2700097O09Rik	0.00	-1.4	0.00	-1.5	0.09	-1.3
A_55_P2361647	NR_030700	4831440E17Rik	0.00	-1.6	0.01	-1.4	0.07	-1.3
A_52_P337427	NM_027829	9030607L17Rik	0.06	-1.4	0.01	-1.5	0.20	-1.3
A_66_P117058	NM_173765	Aasdh	0.07	-1.4	0.01	-1.5	0.15	-1.3
A_55_P1962906	NM_001146060	Als2cl	0.12	-1.3	0.00	-1.7	0.06	-1.3
A_55_P2049582	NM_146085	Apbb3	0.28	-1.3	0.02	-1.5	0.25	-1.3
A_52_P251450	NM_016675	Cldn2	0.88	-1.1	0.04	-1.5	0.31	-1.3
A_52_P679152	NM_001042634	Clk1	0.01	-1.4	0.00	-1.5	0.05	-1.3
A_55_P2107731	NM_028518	Col20a1	0.14	-1.4	0.01	-1.5	0.12	-1.3
A_55_P2039044	NM_001105160	Cyp3a59	0.79	-1.3	0.03	-2.0	0.64	-1.3
A_51_P359570	NM_010501	Ifit3	0.54	-1.2	0.00	-1.6	0.12	-1.3
A_52_P609334	NM_030075	Klhdc8b	0.04	-1.4	0.00	-1.6	0.06	-1.3
A_55_P2040815	NM_153545	Lrrc45	0.00	-1.5	0.00	-1.4	0.06	-1.3
A_66_P127567	NM_016813	Nxf1	0.00	-1.7	0.00	-1.7	0.07	-1.3
A_51_P261107	NM_139144	Ogt	0.00	-1.7	0.00	-1.6	0.21	-1.3
A_66_P125831	NM_008891	Pnn	0.00	-1.6	0.00	-1.5	0.06	-1.3
A_52_P670026	NM_021384	Rsad2	0.89	-1.1	0.01	-1.6	0.16	-1.3
A_52_P171791	NM_001031814	Smg1	0.00	-1.5	0.00	-1.5	0.02	-1.3
A_52_P550147	AJ584850	Sned1	0.02	-1.5	0.04	-1.4	0.26	-1.3
A_51_P267933	NM_175692	Snhg11	0.09	-1.7	0.01	-1.9	0.50	-1.3
A_55_P2059606	NM_019963	Stat2	0.25	-1.3	0.00	-1.7	0.20	-1.3
A_51_P164939	NM_144916	Tmem150a	0.20	-1.3	0.01	-1.5	0.07	-1.3
A_52_P207635	NM_001033261	Zfc3h1	0.02	-1.6	0.02	-1.7	0.27	-1.3
A_55_P1999022	NM_001008501	Zfp760	0.00	-1.6	0.02	-1.4	0.22	-1.3
A_52_P118706	AK052880		0.02	-1.5	0.04	-1.4	0.13	-1.4
A_30_P01033161			0.00	-1.5	0.00	-1.5	0.01	-1.4

A_30_P01030677			0.00	-1.5	0.00	-1.7	0.03	-1.4
A_55_P1959061	AF328926		0.00	-1.5	0.00	-1.5	0.06	-1.4
A_30_P01028826			0.00	-1.5	0.01	-1.4	0.02	-1.4
A_30_P01033086			0.02	-1.7	0.08	-1.5	0.19	-1.4
A_30_P01032624			0.86	-1.1	0.04	-1.5	0.13	-1.4
A_30_P01018881			0.11	-1.4	0.02	-1.5	0.05	-1.4
A_30_P01018104			0.10	-1.4	0.02	-1.5	0.05	-1.4
A_30_P01029457			0.05	-1.4	0.00	-1.6	0.05	-1.4
A_30_P01028635			0.99	-1.0	0.03	-1.8	0.37	-1.4
A_30_P01024852			0.90	1.2	0.03	-1.9	0.53	-1.4
A_30_P01022259			0.30	-1.4	0.00	-2.1	0.22	-1.4
A_30_P01027314			0.95	1.1	0.03	-2.3	0.61	-1.4
A_55_P2390776	NR_027981	1810064F22Rik	0.44	-1.3	0.00	-1.6	0.13	-1.4
A_51_P163233	NM_028814	2810403A07Rik	0.00	-1.4	0.00	-1.6	0.04	-1.4
A_55_P2251974	AK044736	A930038B10Rik	0.09	-1.5	0.01	-1.7	0.15	-1.4
A_55_P1985428	NM_001111111	Atg16l2	0.61	-1.3	0.03	-1.5	0.18	-1.4
A_66_P115996	NM_172404	Ccbl1	0.31	-1.3	0.02	-1.5	0.05	-1.4
A_51_P470715	NM_009895	Cish	0.93	-1.2	0.01	-3.4	0.73	-1.4
A_52_P480088	NM_025685	Col27a1	0.01	-1.9	0.00	-1.9	0.37	-1.4
A_51_P325501	NM_053250	Crip3	0.02	-1.6	0.00	-1.7	0.14	-1.4
A_55_P2047621	NM_030021	D730039F16Rik	0.56	-1.3	0.03	-1.5	0.11	-1.4
A_52_P223809	NM_030150	Dhx58	0.78	-1.2	0.00	-1.7	0.11	-1.4
A_51_P406165	NM_030069	Fam55b	0.92	-1.1	0.01	-1.8	0.29	-1.4
A_55_P1952638	NM_053072	Fgd6	0.79	-1.2	0.00	-1.7	0.14	-1.4
A_55_P2040090	NM_001038699	Fn3k	0.01	-1.5	0.01	-1.5	0.10	-1.4
A_55_P2067533	NM_010446	Foxa2	0.99	-1.0	0.04	-1.7	0.28	-1.4
A_55_P1956502	NM_021510	Hnrnph1	0.00	-1.4	0.00	-1.5	0.00	-1.4
A_51_P424221	NM_001164598	Irf2bp2	0.68	-1.2	0.02	-1.5	0.09	-1.4
A_55_P2103698	NM_015783	Isg15	0.80	-1.2	0.02	-1.6	0.16	-1.4
A_55_P2149500	NM_010630	Kifc2	0.03	-1.6	0.02	-1.5	0.18	-1.4
A_55_P2102857	NM_027221	Krtcap3	0.00	-1.4	0.00	-1.5	0.03	-1.4
A_55_P2064771	NM_010741	Ly6c1	0.77	-1.2	0.03	-1.7	0.17	-1.4
A_52_P128964	NM_029342	Nhej1	0.04	-1.4	0.00	-1.5	0.04	-1.4
A_55_P2016034	NM_001033207	Nlrc5	0.40	-1.3	0.00	-2.0	0.14	-1.4
A_52_P356204	NM_181547	Nostrin	0.87	-1.1	0.01	-1.5	0.06	-1.4
A_55_P2128606	NM_198658	Nr1h5	0.08	-1.5	0.02	-1.6	0.14	-1.4
A_55_P2053958	NM_030566	Rabep2	0.00	-1.5	0.06	-1.4	0.03	-1.4
A_66_P121787	NM_010156	Samd9l	0.15	-1.4	0.00	-1.6	0.03	-1.4
A_55_P2141306	NM_025292	Synj2bp	0.00	-1.5	0.00	-1.6	0.04	-1.4
A_55_P2083629	NM_019725	Tle2	0.03	-1.4	0.00	-1.5	0.07	-1.4
A_51_P348183	NM_001040130	Tmem141	0.23	-1.2	0.00	-1.5	0.00	-1.4
A_52_P158431	NM_080455	Tshz2	0.94	1.1	0.04	-1.6	0.21	-1.4
A_55_P2114953	NM_011909	Usp18	0.59	-1.3	0.02	-1.6	0.15	-1.4
A_55_P2079103	NM_133992	Usp52	0.06	-1.4	0.00	-1.5	0.02	-1.4
A_55_P2083879	NM_020518	Vsig2	0.21	-1.3	0.01	-1.5	0.06	-1.4
A_51_P108020	NM_001029929	Zmynd15	0.18	-1.3	0.00	-1.5	0.03	-1.4
A_30_P01031034			0.02	-1.5	0.00	-1.7	0.02	-1.5
A_30_P01020357			0.02	-1.5	0.00	-1.6	0.01	-1.5
A_30_P01029682			0.00	-1.5	0.00	-1.4	0.01	-1.5

A_30_P01029746			0.04	-1.6	0.11	-1.4	0.08	-1.5
A_30_P01032180			0.00	-1.6	0.00	-1.5	0.02	-1.5
A_55_P2184449	AK134871		0.00	-1.6	0.00	-2.0	0.03	-1.5
A_30_P01026923			0.12	-1.4	0.38	-1.2	0.04	-1.5
A_55_P1995593			0.71	-1.2	0.38	-1.2	0.04	-1.5
A_55_P2073329			0.99	1.0	0.70	-1.1	0.04	-1.5
A_30_P01026230			0.78	-1.1	0.77	-1.1	0.04	-1.5
A_55_P2170405			0.99	-1.0	0.07	-1.5	0.04	-1.5
A_52_P311031	AK016943		0.85	-1.1	0.78	-1.1	0.03	-1.5
A_30_P01024297			0.83	-1.1	0.67	-1.1	0.01	-1.5
A_30_P01019898			1.00	1.0	0.86	1.1	0.01	-1.5
A_30_P01028808			0.96	1.0	0.99	1.0	0.01	-1.5
A_30_P01027714			0.99	-1.0	0.14	-1.3	0.01	-1.5
A_55_P1959800	AK080717		0.66	-1.2	0.09	-1.3	0.01	-1.5
A_51_P483483	AK014549		0.01	-1.4	0.00	-1.4	0.00	-1.5
A_30_P01027463			0.90	1.1	0.58	1.1	0.00	-1.5
A_52_P392509	AK018404		0.03	-1.4	0.00	-1.5	0.00	-1.5
A_30_P01031307			0.20	-1.3	0.01	-1.4	0.00	-1.5
A_30_P01026204			0.50	-1.2	0.10	-1.2	0.00	-1.5
A_55_P2072925			0.95	-1.1	0.01	-1.9	0.29	-1.5
A_30_P01024256			0.25	-1.7	0.00	-2.8	0.38	-1.5
A_66_P107745	NR_027930	1110020A21Rik	0.00	-1.5	0.00	-1.6	0.01	-1.5
A_55_P2029746	XM_001472371	1200016E24Rik	0.88	1.1	0.83	-1.1	0.04	-1.5
A_55_P2201454	AK142062	2310079F09Rik	0.86	-1.1	0.03	-1.4	0.03	-1.5
A_55_P2095118	NR_015531	2700023E23Rik	0.04	-1.4	0.02	-1.4	0.01	-1.5
A_55_P2286493	AK016664	4933405E24Rik	0.95	-1.0	0.42	-1.2	0.00	-1.5
A_55_P2218334	AK034586	9430011C21Rik	0.92	1.1	0.76	-1.1	0.02	-1.5
A_55_P2116650	NR_002860	A130040M12Rik	0.56	-1.2	0.22	-1.3	0.01	-1.5
A_55_P1954724	NR_002860	A130040M12Rik	0.75	1.1	0.90	1.1	0.00	-1.5
A_55_P2367415	AK035805	A630026N12Rik	0.27	-1.4	0.00	-1.6	0.04	-1.5
A_55_P2052166	NM_029332	Akap13	0.86	-1.1	0.13	-1.3	0.01	-1.5
A_55_P2149931	NM_178407	Arap2	0.88	-1.1	0.42	-1.2	0.00	-1.5
A_55_P2055419	NM_001081195	Arid4a	0.92	-1.1	0.77	-1.1	0.01	-1.5
A_51_P155763	NM_001146089	Ascc3	0.60	-1.1	0.47	-1.1	0.00	-1.5
A_55_P2225460	AK157022	AW555355	0.94	-1.0	0.01	-1.4	0.00	-1.5
A_52_P43150	NM_007519	Baat	0.51	-1.2	0.03	-1.4	0.01	-1.5
A_55_P2340101	AK144577	BC062258	0.15	-1.4	0.00	-1.6	0.05	-1.5
A_55_P2303310	AK050309	C730036E19Rik	0.22	-1.6	0.02	-1.8	0.19	-1.5
A_55_P2068723	NM_011336	Ccl27a	0.14	-1.3	0.11	-1.3	0.00	-1.5
A_52_P657240	NM_027879	Cdc40	0.99	-1.0	0.67	-1.1	0.02	-1.5
A_52_P350750	NM_015730	Chrna4	0.01	-1.5	0.05	-1.4	0.02	-1.5
A_66_P115531	NM_145151	Crebzf	0.00	-1.7	0.00	-1.8	0.00	-1.5
A_51_P116813	NM_007809	Cyp17a1	1.00	1.0	0.92	1.0	0.01	-1.5
A_52_P640922	NM_177577	Dcdc2a	0.40	-1.2	0.17	-1.2	0.00	-1.5
A_52_P226127	NM_011805	Dido1	0.00	-2.0	0.00	-2.0	0.04	-1.5
A_55_P2430472	NM_011805	Dido1	0.93	-1.1	0.97	1.0	0.00	-1.5
A_51_P315904	NM_011817	Gadd45g	0.77	-1.5	0.01	-2.9	0.58	-1.5
A_55_P2124461	XM_001472850	Gm2251	0.80	-1.1	0.64	-1.1	0.01	-1.5
A_55_P2083894	XM_001478221	Gm3697	0.00	-1.6	0.00	-1.7	0.00	-1.5

A_55_P2037787	XM_001478340	Gm3798	0.00	-1.5	0.00	-1.6	0.00	-1.5
A_55_P1957213	XM_001479389	Gm4148	0.71	1.2	0.89	1.1	0.01	-1.5
A_55_P2120141	XM_001480410	Gm4522	0.66	-1.2	0.47	-1.2	0.02	-1.5
A_55_P2120919	XM_001481156	Gm4703	0.00	-1.6	0.00	-1.8	0.01	-1.5
A_55_P2041693	XM_619677	Gm5834	0.93	1.0	0.61	-1.1	0.00	-1.5
A_55_P2203051	NR_027008	Gt(ROSA)26Sor	0.02	-1.3	0.00	-1.5	0.00	-1.5
A_55_P1988202	NM_001045481	Ifi203	0.07	-1.4	0.01	-1.5	0.01	-1.5
A_52_P90363	NM_029803	Ifi27l2a	0.63	-1.4	0.02	-2.1	0.39	-1.5
A_51_P487690	NM_133871	Ifi44	0.29	-1.3	0.01	-1.5	0.02	-1.5
A_66_P103271	NM_001164598	Irf2bp2	0.81	-1.1	0.10	-1.3	0.02	-1.5
A_55_P2431118	NM_001110275	Itsn1	0.69	-1.2	0.32	-1.2	0.00	-1.5
A_52_P617636	NM_010636	Klf12	0.98	1.0	0.24	-1.3	0.03	-1.5
A_66_P130035	NM_010643	Klk1b24	0.98	-1.0	0.07	-1.4	0.04	-1.5
A_55_P2002933	NM_008456	Klk1b5	0.61	-1.2	0.04	-1.5	0.05	-1.5
A_55_P2102419	NM_023684	Lime1	0.01	-1.5	0.00	-1.6	0.02	-1.5
A_51_P459661	NM_021460	Lipa	0.97	-1.0	0.82	-1.1	0.04	-1.5
A_55_P2072041	XM_001472087	LOC100038980	0.69	-1.2	0.52	-1.1	0.01	-1.5
A_55_P2078831	XM_001473861	LOC100045156	0.82	-1.1	0.67	-1.1	0.01	-1.5
A_55_P2033615	XM_001473874	LOC100045214	0.66	-1.2	0.01	-1.4	0.01	-1.5
A_55_P1961241	XM_001476526	LOC100046627	0.17	-1.5	0.01	-1.7	0.12	-1.5
A_66_P112862	NR_033146	LOC100316870	0.99	-1.0	0.91	-1.1	0.03	-1.5
A_55_P2052385	NM_194336	Mpa2l	0.90	-1.1	0.04	-1.8	0.21	-1.5
A_55_P2082604	NM_001082476	Ndor1	0.73	-1.1	0.71	-1.1	0.00	-1.5
A_55_P2116978	NM_010889	Neb	0.27	-1.6	0.01	-2.2	0.33	-1.5
A_51_P296036	NM_144847	Nrbp2	0.09	-1.3	0.00	-1.4	0.00	-1.5
A_55_P2019719	NM_145227	Oas2	0.05	-1.4	0.00	-1.7	0.03	-1.5
A_55_P1998374	NM_001170433	Ppfibp1	0.92	-1.1	0.59	-1.2	0.01	-1.5
A_51_P504314	NM_025845	Prpf38b	0.58	-1.2	0.07	-1.3	0.00	-1.5
A_55_P2408330	NM_028234	Prr8	0.00	-1.6	0.00	-1.9	0.02	-1.5
A_55_P2151601	NM_010156	Samd9l	0.46	-1.3	0.00	-1.8	0.07	-1.5
A_51_P267278	NM_021301	Slc15a2	0.11	-1.6	0.00	-1.9	0.14	-1.5
A_55_P2022123	NM_001045531	Spata22	0.82	-1.1	0.09	-1.4	0.03	-1.5
A_55_P2139913	NM_009278	Ssb	0.88	-1.1	0.66	-1.1	0.02	-1.5
A_55_P1960298	NM_027797	Tmem80	0.04	-1.5	0.00	-1.7	0.01	-1.5
A_55_P2032818	NM_030706	Trim2	0.92	-1.1	0.58	-1.2	0.04	-1.5
A_55_P2052485	NM_181418	Ushbp1	0.01	-1.6	0.01	-1.8	0.08	-1.5
A_51_P476509	NM_175537	Zbtb38	0.62	-1.2	0.27	-1.3	0.03	-1.5
A_52_P217604	NM_182996	Zfp692	0.00	-1.7	0.00	-1.6	0.00	-1.5
A_30_P01021604			0.04	-1.5	0.01	-1.5	0.02	-1.6
A_30_P01031524			0.03	-1.5	0.03	-1.5	0.01	-1.6
A_30_P01030640			0.00	-1.5	0.00	-1.4	0.00	-1.6
A_30_P01022593			0.02	-1.6	0.20	-1.4	0.04	-1.6
A_30_P01030419			0.01	-1.6	0.01	-1.6	0.02	-1.6
A_55_P2129222	AK013167		0.95	1.1	0.36	-1.3	0.04	-1.6
A_30_P01019750			0.97	1.0	0.79	-1.1	0.03	-1.6
A_30_P01026357			0.91	-1.1	0.29	-1.3	0.01	-1.6
A_55_P2003199	AK141316		0.97	-1.0	0.60	-1.2	0.01	-1.6
A_55_P2131190			0.86	-1.1	0.01	-1.6	0.01	-1.6
A_55_P2003393	AK141001		0.06	-1.4	0.08	-1.4	0.01	-1.6

A_30_P01026192			0.89	-1.1	0.27	-1.2	0.00	-1.6
A_30_P01025480			0.18	-1.3	0.00	-1.6	0.00	-1.6
A_55_P2019577	NR_027818	1500011B03Rik	0.30	-1.4	0.00	-2.1	0.05	-1.6
A_52_P546660	NM_183112	1700029I15Rik	0.00	-1.8	0.01	-1.7	0.05	-1.6
A_55_P2387665	AK033690	9130221J18Rik	0.90	-1.2	0.02	-2.4	0.45	-1.6
A_55_P2276224	AK034303	9330175E14Rik	0.00	-1.6	0.00	-1.7	0.00	-1.6
A_55_P2105321	NM_023190	Acin1	0.78	-1.1	0.60	-1.1	0.01	-1.6
A_66_P127175	NM_177268	Ankrd16	0.00	-1.7	0.00	-1.7	0.01	-1.6
A_55_P1990879	NM_016812	Banp	0.17	-1.2	0.00	-1.4	0.00	-1.6
A_55_P2098598	NM_007569	Btg1	1.00	-1.0	0.28	-1.3	0.03	-1.6
A_55_P2298319	AK083183	C730029A08Rik	0.95	-1.1	0.01	-1.7	0.06	-1.6
A_55_P1987904	AK138814	Chpf2	0.00	-1.8	0.00	-1.9	0.00	-1.6
A_55_P2078494	NM_001080812	Cib3	0.18	-2.0	0.03	-2.3	0.36	-1.6
A_51_P180452	NM_133656	Crk	0.41	-1.3	0.35	-1.2	0.00	-1.6
A_51_P341465	NM_009970	Csf2ra	0.23	-1.4	0.00	-1.9	0.02	-1.6
A_55_P2303972	AK077973	D830044D21Rik	0.15	-1.6	0.01	-1.8	0.12	-1.6
A_55_P1992164	XM_001472210	E130102H24Rik	0.00	-1.9	0.00	-2.0	0.07	-1.6
A_55_P2430221	NM_029389	Fam35a	0.93	-1.1	0.88	-1.1	0.04	-1.6
A_51_P267544	NM_013522	Frg1	0.98	-1.0	0.94	-1.0	0.00	-1.6
A_66_P105032	NM_001145034	Gm13889	0.87	-1.1	0.30	-1.3	0.02	-1.6
A_55_P2146655	XM_001472967	Gm2262	0.76	-1.2	0.20	-1.3	0.02	-1.6
A_55_P2140212	XM_001474216	Gm2627	0.98	1.0	0.70	-1.1	0.02	-1.6
A_55_P2088720	XM_001477698	Gm3651	0.95	1.1	0.92	-1.1	0.04	-1.6
A_52_P119350	XM_001478300	Gm3866	0.76	-1.2	0.13	-1.4	0.03	-1.6
A_55_P2079713	NM_153528	Gramd1c	0.03	-1.6	0.00	-1.9	0.04	-1.6
A_55_P1982451	NM_016710	Hmgn5	0.81	-1.1	0.11	-1.2	0.00	-1.6
A_51_P347452	NM_028242	Htatsf1	0.26	-1.2	0.00	-1.3	0.00	-1.6
A_55_P2063257	NM_010706	Lgals4	0.30	-1.5	0.03	-1.8	0.12	-1.6
A_55_P1974452	NM_023684	Lime1	0.00	-1.8	0.00	-2.0	0.00	-1.6
A_55_P2162404	XM_001477908	LOC100047327	0.94	-1.1	0.27	-1.3	0.02	-1.6
A_55_P2001238	NM_026313	Luc7l3	0.35	-1.2	0.19	-1.2	0.00	-1.6
A_55_P2059019	NM_001163447	Mapk8ip3	0.00	-1.6	0.00	-2.0	0.00	-1.6
A_51_P514085	NM_013606	Mx2	0.18	-1.4	0.00	-2.3	0.02	-1.6
A_55_P2061645	AK140300	ND6	0.92	-1.1	0.88	-1.1	0.00	-1.6
A_52_P506250	NM_146655	Olfr441	0.89	-1.1	0.07	-1.3	0.00	-1.6
A_55_P1977653	NM_001081390	Palld	0.77	-1.2	0.06	-1.5	0.04	-1.6
A_51_P279038	NM_008904	Ppargc1a	0.72	-1.2	0.18	-1.4	0.03	-1.6
A_52_P134023	NM_153100	Rtp3	0.41	-1.4	0.01	-1.7	0.04	-1.6
A_55_P2149821	NM_026989	Sfrs11	0.00	-1.3	0.10	-1.3	0.00	-1.6
A_51_P390967	NM_021398	Slc43a3	0.69	-1.1	0.57	-1.1	0.00	-1.6
A_51_P314186	NM_001079686	Syne1	0.94	-1.1	0.28	-1.3	0.02	-1.6
A_55_P1955078	NM_145580	Tmem149	0.00	-1.7	0.00	-1.8	0.01	-1.6
A_55_P2126572	NM_027865	Tmem25	0.02	-1.8	0.00	-1.9	0.09	-1.6
A_55_P2130219	NM_133780	Tpr	0.99	-1.0	0.90	-1.0	0.00	-1.6
A_51_P235801	NM_007564	Zfp36l1	0.69	-1.2	0.00	-1.5	0.00	-1.6
A_55_P2153555	NM_001145778	Zkscan3	0.07	-1.5	0.11	-1.4	0.01	-1.6
A_30_P01031627			0.00	-1.5	0.00	-1.7	0.00	-1.7
A_30_P01022284			0.00	-1.6	0.00	-1.8	0.01	-1.7
A_30_P01032980			0.01	-1.7	0.02	-1.7	0.02	-1.7

A_55_P2146500			0.00	-1.7	0.00	-1.7	0.00	-1.7
A_30_P01024984			0.00	-1.9	0.00	-2.2	0.02	-1.7
A_30_P01025102			0.00	-2.1	0.00	-1.9	0.00	-1.7
A_30_P01022085			0.83	-1.2	0.03	-1.6	0.02	-1.7
A_30_P01030159			0.82	-1.2	0.06	-1.5	0.02	-1.7
A_30_P01027919			0.99	-1.0	0.01	-1.6	0.01	-1.7
A_55_P2076805	AK129022		0.48	-1.3	0.82	-1.1	0.00	-1.7
A_30_P01031134			0.50	-1.2	0.01	-1.5	0.00	-1.7
A_66_P116451	XM_001476411	2210039B01Rik	0.02	-1.7	0.00	-2.0	0.03	-1.7
A_55_P2011121	NM_029197	4930528F23Rik	0.83	-1.2	0.02	-1.6	0.01	-1.7
A_55_P2079928	NM_201362	Ccdc68	0.55	-1.3	0.00	-1.9	0.03	-1.7
A_52_P329367	NM_001025566	Chka	0.96	-1.1	0.21	-1.4	0.02	-1.7
A_66_P122511	NM_016675	Cldn2	0.97	1.0	0.17	-1.4	0.01	-1.7
A_55_P2176535	NM_010007	Cyp2j5	0.96	1.0	0.43	-1.2	0.01	-1.7
A_51_P330044	NM_028979	Cyp2j9	0.04	-1.7	0.00	-2.1	0.07	-1.7
A_55_P2142451	NM_026728	Echdc2	0.00	-1.6	0.00	-1.6	0.00	-1.7
A_55_P2142439	NM_026728	Echdc2	0.07	-1.4	0.05	-1.4	0.00	-1.7
A_51_P407879	NM_145382	Fam193b	0.00	-1.8	0.00	-2.1	0.00	-1.7
A_51_P421876	NM_016850	Irf7	0.72	-1.3	0.09	-1.6	0.04	-1.7
A_55_P2063256	NM_010706	Lgals4	0.18	-1.6	0.01	-1.9	0.06	-1.7
A_51_P497100	NM_010706	Lgals4	0.58	-1.4	0.00	-1.9	0.08	-1.7
A_55_P2026959	XM_001476908	LOC100046852	0.67	-1.2	0.09	-1.4	0.00	-1.7
A_66_P139460	NM_177389	Mia3	0.98	1.0	0.69	-1.1	0.00	-1.7
A_55_P2347976	NR_029382	Mirhg1	0.00	-1.7	0.00	-1.8	0.00	-1.7
A_55_P2034067	NM_001085509	Myom3	0.60	-1.5	0.01	-2.1	0.18	-1.7
A_55_P1985239	NM_178617	Necab1	0.56	-1.2	0.10	-1.3	0.00	-1.7
A_55_P1979699	NM_025287	Spop	0.64	-1.2	0.32	-1.2	0.00	-1.7
A_55_P1968355	NM_011599	Tle1	0.88	-1.1	0.60	-1.2	0.03	-1.7
A_55_P1996613	NM_029704	Ttc19	0.90	-1.1	0.94	1.0	0.00	-1.7
A_55_P2052490	NM_181418	Ushbp1	0.41	-1.3	0.00	-1.7	0.00	-1.7
A_66_P100937	XM_001487796	Zfp33b	0.99	-1.0	0.35	-1.3	0.00	-1.7
A_30_P01031014			0.00	-1.7	0.00	-1.6	0.00	-1.8
A_30_P01031112			0.00	-2.0	0.00	-2.3	0.00	-1.8
A_30_P01026776			0.98	-1.0	0.03	-1.8	0.03	-1.8
A_30_P01021387			0.72	-1.3	0.11	-1.5	0.03	-1.8
A_30_P01023533			0.69	-1.3	0.00	-2.0	0.02	-1.8
A_30_P01024055			0.09	-1.7	0.02	-1.9	0.02	-1.8
A_30_P01019709			0.07	-1.5	0.00	-1.7	0.01	-1.8
A_55_P2189893	AK042233	A630073K07Rik	0.89	-1.1	0.07	-1.5	0.00	-1.8
A_55_P2293351	AK082896	C430010C01	0.00	-1.8	0.00	-2.1	0.00	-1.8
A_55_P2080956	NM_013490	Chka	0.90	-1.1	0.60	-1.3	0.04	-1.8
A_52_P48681	NM_016674	Cldn1	0.78	-1.2	0.02	-1.9	0.04	-1.8
A_55_P2059010	NM_011254	Rbp1	0.20	-1.5	0.23	-1.4	0.00	-1.8
A_55_P2022499	NM_025669	Sfrs18	0.00	-1.9	0.00	-2.2	0.00	-1.8
A_55_P1976574	NM_016712	Tmod4	0.00	-2.0	0.00	-2.2	0.01	-1.8
A_55_P1966833	NM_001037713	Xaf1	0.74	-1.2	0.00	-1.8	0.00	-1.8
A_30_P01031056			0.00	-1.7	0.00	-1.7	0.00	-1.9
A_55_P2112225	AK191463		0.00	-2.0	0.00	-2.2	0.00	-1.9
A_30_P01018976			0.50	-1.4	0.18	-1.5	0.01	-1.9

A_55_P2153191			0.96	1.1	0.99	-1.0	0.01	-1.9
A_66_P115161	AK039734		0.74	-1.2	0.65	-1.2	0.00	-1.9
A_55_P2258261	AK050412	1810008I18Rik	0.89	1.2	0.86	-1.1	0.02	-1.9
A_51_P158073	NM_175687	A230050P20Rik	0.00	-1.8	0.00	-2.1	0.00	-1.9
A_51_P356265	NM_016845	Acrbp	0.00	-2.2	0.00	-2.6	0.01	-1.9
A_55_P1992849	NM_013462	Adrb3	0.02	-2.2	0.00	-3.1	0.06	-1.9
A_55_P2008538	NM_029362	Chmp4b	0.48	-1.4	0.03	-1.8	0.02	-1.9
A_55_P2140107	NM_009995	Cyp21a1	0.01	-2.2	0.00	-3.0	0.07	-1.9
A_51_P381321	NM_199080	Ddx17	0.00	-2.2	0.00	-2.5	0.00	-1.9
A_51_P383270	NM_175473	Fras1	0.25	-1.4	0.06	-1.4	0.00	-1.9
A_55_P1990633	NM_001146275	Iigp1	0.61	-1.3	0.00	-1.9	0.00	-1.9
A_66_P134394	BC027050	Pde6c	0.06	-1.9	0.27	-1.5	0.03	-1.9
A_51_P229602	NM_008882	Plxna2	0.82	-1.2	0.12	-1.6	0.04	-1.9
A_55_P2097518	NM_001080943	Zdhhc22	0.77	-1.3	0.33	-1.5	0.04	-1.9
A_30_P01022612			0.00	-2.2	0.00	-2.6	0.01	-2.0
A_55_P2340593	AK007545	1810019D21Rik	0.00	-2.4	0.00	-3.0	0.00	-2.0
A_55_P2348582	AK019523	4833439F03Rik	0.15	-1.7	0.01	-2.0	0.00	-2.0
A_55_P2105685	NM_013462	Adrb3	0.01	-1.9	0.00	-2.8	0.01	-2.0
A_51_P315042	NM_016847	Avpr1a	0.91	-1.1	0.23	-1.5	0.02	-2.0
A_55_P1956223	NM_013490	Chka	0.70	-1.3	0.25	-1.5	0.01	-2.0
A_51_P153486	NM_018808	Dnajb1	0.19	-1.3	0.00	-1.7	0.00	-2.0
A_55_P1986341	NR_002858	Gm4956	0.00	-2.6	0.00	-2.4	0.01	-2.0
A_52_P322826	XM_001473345	Gm7461	0.67	-1.3	0.19	-1.5	0.01	-2.0
A_52_P86693	NM_026790	Ifi27l1	0.69	-1.3	0.00	-2.2	0.00	-2.0
A_66_P139387	NM_011169	Prlr	0.95	1.1	0.74	-1.2	0.03	-2.0
A_30_P01021853			0.00	-2.5	0.00	-3.9	0.02	-2.1
A_30_P01021970			0.70	-1.2	0.71	-1.1	0.00	-2.1
A_52_P223508	NR_026976	6720401G13Rik	0.00	-2.2	0.00	-1.9	0.00	-2.1
A_51_P142923	NM_013490	Chka	0.64	-1.4	0.31	-1.5	0.03	-2.1
A_55_P2027213	NM_001163615	Krtap20-2	0.56	-1.4	0.01	-1.8	0.00	-2.1
A_66_P122158	NR_003518	Pisd-ps3	0.00	-2.1	0.00	-2.2	0.00	-2.1
A_55_P1963483	NM_030559	Vps16	0.89	-1.2	0.81	-1.2	0.04	-2.1
A_30_P01031894			0.00	-2.5	0.00	-3.1	0.00	-2.2
A_30_P01018771			0.00	-2.6	0.00	-2.5	0.00	-2.2
A_52_P379277	NM_134005	Enpp3	0.69	1.4	0.83	1.2	0.01	-2.2
A_55_P2048499	XM_001474426	Gm10061	0.37	-1.4	0.00	-1.8	0.00	-2.2
A_55_P2065929	XM_914410	LOC638393	0.28	-1.6	0.03	-1.8	0.00	-2.2
A_30_P01030953			0.00	-2.3	0.00	-2.5	0.00	-2.3
A_30_P01020303			0.00	-3.0	0.00	-5.3	0.02	-2.3
A_30_P01022030			0.00	-3.3	0.00	-5.3	0.01	-2.3
A_55_P2279807	AK160312	6720427I07Rik	0.00	-2.4	0.00	-2.3	0.00	-2.3
A_55_P2274378	AK035112	AW549542	0.01	-2.5	0.00	-3.2	0.03	-2.3
A_30_P01028297			0.00	-2.5	0.00	-2.7	0.00	-2.4
A_30_P01028215			0.63	-1.2	0.84	-1.1	0.00	-2.4
A_30_P01020677			0.56	-1.5	0.00	-2.2	0.00	-2.4
A_51_P386304	NM_207678	Ccnl2	0.00	-2.5	0.00	-2.8	0.00	-2.4
A_55_P2068459	NM_010479	Hspa1a	0.02	-1.7	0.00	-2.4	0.00	-2.4
A_51_P257885	NM_175217	Mmd2	0.58	-1.6	0.06	-2.2	0.03	-2.4
A_30_P01031754			0.56	-1.2	0.45	-1.2	0.00	-2.5

A_55_P2332194	AK140090	Gm15998	0.82	-1.3	0.00	-2.6	0.01	-2.5
A_55_P2127179	XM_142052	Gm379	0.64	1.5	0.02	-2.4	0.01	-2.5
A_55_P2061104	NM_001081285	Mup6	0.77	-1.4	0.85	-1.2	0.01	-2.5
A_52_P409578	NM_178045	Rassf4	0.15	-2.0	0.22	-1.7	0.02	-2.5
A_51_P455997	NR_027652	Meg3	0.02	-2.1	0.00	-2.6	0.01	-2.6
A_30_P01033314			0.75	-1.3	0.06	-1.9	0.00	-2.7
A_51_P416059	NM_153550	Dirc2	0.46	-1.4	0.03	-1.8	0.00	-2.7
A_55_P2024155	NM_001033324	Zbtb16	0.59	-1.5	0.00	-3.6	0.01	-2.8
A_51_P241995	NM_016919	Col5a3	0.00	-3.1	0.00	-3.7	0.00	-3.1
A_51_P162671	NM_008035	Folr2	0.95	1.1	0.91	-1.2	0.00	-3.1
A_55_P2042778	NM_153422	Pde5a	0.38	-1.6	0.00	-2.2	0.00	-3.4
A_55_P2165091	NM_145368	Acnat2	0.50	-1.7	0.00	-5.6	0.00	-3.7
A_51_P456208	NM_011575	Tff3	0.39	-1.8	0.16	-1.9	0.00	-3.9
A_52_P267391	NM_023835	Trim12	0.97	-1.1	0.97	-1.1	0.02	-3.9
A_30_P01023251			0.00	-3.5	0.00	-4.7	0.00	-4.0
A_55_P2075263	NM_145368	Acnat2	0.22	-2.1	0.00	-5.7	0.00	-4.1
A_30_P01020135			0.00	-3.7	0.00	-5.1	0.00	-4.2
A_30_P01025798			0.00	-3.9	0.00	-5.3	0.00	-4.5
A_55_P2055423	XM_485799	Nat8b	0.70	-1.8	0.09	-2.9	0.02	-4.5
A_30_P01021631			0.00	-4.5	0.00	-5.6	0.00	-4.6
A_51_P234692	NR_003513	Neat1	0.00	-4.6	0.00	-5.8	0.00	-4.6
A_30_P01029720			0.00	-4.2	0.00	-5.5	0.00	-4.8
A_66_P101646	AK165865	COX1	0.33	-1.8	0.00	-3.1	0.00	-5.7

3.2.2 BbF Liver. Significant probe list. List of all significantly differentially expressed probes in at least 1 treatment group (FDR P ≤ 0.05, fold change ± 1.5) in response to sub-chronic oral exposure to 25, 50, and 100 mg/kg-bw/day benzo(b)fluoranthene in the liver. The list is sorted from highest to lowest fold change in the 100 mg/kg-bw/day treatment group.

			**25 mg/kg-bw/day**		**50 mg/kg-bw/day**		**100 mg/kg-bw/day**	
Agilent Probe	Accession Number	Gene Symbol	*FDR P value*	*Fold change*	*FDR P value*	*Fold change*	*FDR P value*	*Fold change*
A_51_P279693	NM_009992	Cyp1a1	0.83	-1.5	0.72	-1.5	0.00	6.7
A_55_P2032081	NM_016974	Dbp	0.00	4.0	0.00	5.1	0.00	5.8
A_55_P2032079	NM_016974	Dbp	0.00	3.5	0.00	4.1	0.00	5.0
A_55_P2135203	XM_001477565	Gm9454	0.11	2.3	0.00	3.1	0.00	5.0
A_55_P1953387	NM_010634	Fabp5	0.00	2.6	0.00	3.0	0.00	4.4
A_55_P2150976	XM_886827	Fabp5l2	0.00	2.6	0.00	3.0	0.00	4.2
A_55_P1998471	NM_009114	S100a9	0.18	1.7	0.00	2.6	0.00	3.9
A_55_P1983773	NM_001012273	Birc5	0.74	1.4	0.14	1.8	0.00	3.5
A_52_P366803	NM_177380	Cyp3a44	0.92	1.2	0.39	1.6	0.00	3.5
A_55_P1965154	NM_025565	Spc25	0.76	1.3	0.29	1.5	0.00	3.4
A_51_P269404	NM_008030	Fmo3	0.72	1.7	0.10	2.8	0.02	3.4
A_55_P1996946	NM_023223	Cdc20	0.87	1.2	0.36	1.5	0.01	3.2
A_55_P2122841			0.08	2.0	0.00	2.3	0.00	3.0
A_51_P414396	NM_153127	Mmrn2	0.00	1.5	0.00	2.2	0.00	2.8
A_55_P2085779	NM_145449	Ifi27l2b	0.99	1.0	0.19	1.8	0.00	2.7
A_66_P111562	NM_007631	Ccnd1	0.20	1.5	0.00	1.9	0.00	2.6

A_51_P421140	NM_026473	Tubb6	0.88	1.2	0.29	1.5	0.00	2.5
A_55_P2068663	NM_019641	Stmn1	0.63	1.4	0.16	1.6	0.00	2.5
A_51_P287198	NM_033373	Krt23	0.63	1.4	0.20	1.6	0.01	2.5
A_51_P360492	NM_008567	Mcm6	0.98	1.1	0.24	1.6	0.00	2.5
A_51_P451151	NM_026785	Ube2c	0.81	1.3	0.29	1.5	0.00	2.5
A_52_P140005	NM_001081205	Nipal1	0.88	1.2	0.00	2.9	0.00	2.4
A_51_P367866	NM_007913	Egr1	0.00	2.6	0.00	3.5	0.01	2.4
A_55_P1974487	NM_177195	4930417M19Rik	0.38	1.5	0.01	1.9	0.00	2.4
A_51_P365516	NM_009258	Spink3	0.99	-1.0	0.84	1.2	0.03	2.4
A_52_P686785	NM_053247	Lyve1	0.24	1.6	0.00	2.1	0.00	2.4
A_55_P2044653	NM_009999	Cyp2b10	0.29	1.9	0.00	2.7	0.02	2.3
A_51_P363947	NM_007669	Cdkn1a	0.05	1.6	0.00	1.9	0.00	2.3
A_52_P628067	NM_013538	Cdca3	0.94	1.1	0.40	1.4	0.00	2.3
A_51_P253803	NM_001081117	Mki67	0.85	1.2	0.18	1.6	0.00	2.3
A_52_P294510	BC029734	Fgl1	0.15	1.6	0.13	1.6	0.00	2.3
A_55_P2052563	NM_010495	Id1	0.64	1.3	0.22	1.5	0.00	2.2
A_51_P164014	NM_173762	Cenpe	0.92	1.2	0.49	1.4	0.01	2.2
A_55_P1973560			0.99	1.0	0.68	1.3	0.00	2.2
A_55_P1979893	NM_017376	Tef	0.30	1.5	0.00	2.0	0.00	2.2
A_55_P2044242	NM_001004148	Slc13a5	0.35	1.5	0.00	2.0	0.00	2.2
A_55_P2274378	AK035112	AW549542	0.51	1.4	0.00	2.2	0.00	2.2
A_55_P2011436	XM_001474074	Gm11223	0.78	1.3	0.22	1.5	0.00	2.2
A_52_P289091	NM_007813	Cyp2b13	0.72	1.4	0.55	1.4	0.03	2.2
A_52_P267391	NM_023835	Trim12	0.82	1.4	0.02	2.6	0.09	2.2
A_55_P2004447	NM_145594	Fgl1	0.13	1.7	0.33	1.4	0.01	2.1
A_52_P308465	NM_172775	Plxnb1	0.43	1.4	0.00	2.0	0.00	2.1
A_55_P2103706	XM_485921	Gm5593	0.88	1.2	0.40	1.5	0.02	2.1
A_51_P470715	NM_009895	Cish	0.05	4.1	0.00	3.9	0.16	2.1
A_52_P136709	NM_008796	Pctp	1.00	1.0	0.89	1.1	0.02	2.1
A_51_P284608	NM_001042605	Cd74	0.40	1.5	0.20	1.5	0.01	2.1
A_55_P2027392	NM_030258	Gpr146	0.31	1.4	0.00	1.6	0.00	2.1
A_55_P2022074	NM_013692	Klf10	0.50	1.5	0.09	1.7	0.01	2.1
A_51_P375146	NM_007643	Cd36	0.74	1.3	0.04	1.6	0.00	2.1
A_51_P329928	NM_013750	Phlda3	0.35	1.3	0.00	1.5	0.00	2.1
A_55_P2178578	NM_145403	Tmprss4	0.76	1.3	0.02	2.1	0.02	2.1
A_52_P140881	NM_177615	Slc26a10	0.00	1.6	0.00	2.1	0.00	2.1
A_51_P481920	NM_009828	Ccna2	0.85	1.2	0.30	1.5	0.01	2.1
A_66_P111660	NM_013602	Mt1	0.98	-1.0	0.57	1.3	0.00	2.1
A_55_P2046877	NM_008239	Foxq1	0.00	3.6	0.00	4.1	0.07	2.1
A_52_P566840	NM_133776	Gpr110	0.00	1.8	0.00	2.2	0.00	2.0
A_55_P2065671	NM_172301	Ccnb1	1.00	-1.0	0.74	1.2	0.01	2.0
A_55_P1992849	NM_013462	Adrb3	0.35	1.4	0.00	2.0	0.00	2.0
A_55_P2067777	NM_001162906	2410089E03Rik	0.22	1.5	0.00	1.8	0.00	2.0
A_55_P2115330	AK080089		0.97	1.1	0.33	1.5	0.03	2.0
A_55_P2074499	NM_001029985	Kcp	0.55	1.5	0.01	2.0	0.02	2.0
A_51_P117581	NM_022021	Cables1	0.09	1.8	0.01	1.9	0.00	2.0
A_51_P404193	NM_022435	Sp5	0.34	1.4	0.09	1.5	0.00	2.0
A_55_P1996941	NM_026785	Ube2c	0.87	1.2	0.59	1.3	0.00	2.0
A_55_P1984830	NM_145594	Fgl1	0.50	1.5	0.60	1.3	0.00	2.0

A_52_P197402	NM_029057	Tbc1d30	0.32	1.5	0.00	2.1	0.01	2.0
A_55_P1985788	NM_198092	Usp2	0.36	1.5	0.13	1.5	0.00	2.0
A_55_P2024046	NM_001080934	Slc16a5	0.86	1.2	0.04	1.8	0.01	2.0
A_51_P457528	NM_007630	Ccnb2	0.81	1.2	0.51	1.3	0.00	2.0
A_55_P2071059	NM_008668	Nab2	0.00	1.4	0.00	1.8	0.00	1.9
A_55_P2007601	NM_009160	Sftpd	0.96	1.1	0.25	1.5	0.02	1.9
A_55_P2063146			0.94	1.1	0.76	1.2	0.02	1.9
A_51_P152990	NM_011825	Grem2	0.09	1.7	0.00	1.9	0.00	1.9
A_51_P455647	NM_009801	Car2	0.35	1.5	0.12	1.5	0.00	1.9
A_55_P2021119	NM_194342	Unc84b	0.77	1.2	0.02	1.7	0.00	1.9
A_55_P2404484	NR_027980	1700001L05Rik	0.25	1.5	0.00	1.8	0.00	1.9
A_52_P354373	XM_001481164	1190002F15Rik	0.94	1.1	0.46	1.3	0.02	1.9
A_55_P2154132	NM_011654	Tuba1b	0.78	1.2	0.27	1.4	0.01	1.9
A_55_P2013586	NM_133351	Prss8	0.32	1.5	0.18	1.4	0.00	1.9
A_55_P1980636	NM_011497	Aurka	0.86	1.2	0.55	1.3	0.02	1.9
A_52_P240542	NM_010496	Id2	0.11	1.4	0.00	1.7	0.00	1.9
A_55_P2073377	NM_001081117	Mki67	0.90	1.2	0.41	1.4	0.01	1.9
A_51_P177762	NM_153133	Rdh9	0.70	1.3	0.12	1.5	0.00	1.9
A_51_P172054	NM_019521	Gas6	0.68	1.2	0.00	1.8	0.00	1.9
A_55_P2146560	XM_918601	LOC641240	0.72	1.3	0.36	1.4	0.01	1.9
A_55_P2071191	NM_133351	Prss8	0.13	1.5	0.00	1.7	0.00	1.9
A_52_P590154	NM_013786	Hsd17b6	0.09	1.5	0.00	1.7	0.00	1.9
A_55_P2018417	NM_001163645	Osbpl3	0.81	1.2	0.19	1.5	0.01	1.9
A_51_P153063	NM_029310	Fabp12	0.36	1.4	0.27	1.4	0.01	1.9
A_51_P133137	NM_009004	Kif20a	0.95	1.1	0.70	1.2	0.03	1.9
A_55_P2173982	NM_009104	Rrm2	0.82	1.2	0.24	1.5	0.00	1.9
A_55_P1976127	NM_007900	Ect2	0.93	1.1	0.52	1.3	0.01	1.9
A_52_P162099	NM_001004140	Ckap2	0.98	1.0	0.73	1.2	0.01	1.9
A_55_P2063257	NM_010706	Lgals4	0.69	1.2	0.06	1.5	0.01	1.9
A_55_P2133255	NM_019499	Mad2l1	0.82	1.2	0.47	1.3	0.01	1.8
A_52_P603038	NM_016968	Olig1	0.79	1.2	0.44	1.4	0.03	1.8
A_30_P01026091			0.93	1.1	0.71	1.1	0.00	1.8
A_51_P243755	NM_011388	Slc10a2	0.70	1.2	0.01	1.4	0.00	1.8
A_55_P2141860	NM_026531	Aen	0.77	1.2	0.23	1.3	0.00	1.8
A_51_P316951	NM_001167860	Wipf3	0.33	1.5	0.04	1.6	0.01	1.8
A_51_P196844	NM_027881	Osbpl3	0.87	1.2	0.29	1.4	0.02	1.8
A_55_P1982499	NM_177350	Gldn	0.55	1.4	0.11	1.6	0.01	1.8
A_55_P1988083	NM_145150	Prc1	0.97	1.1	0.77	1.2	0.02	1.8
A_52_P303891	NM_011584	Nr1d2	0.24	1.5	0.10	1.6	0.02	1.8
A_55_P2007964	NM_009987	Cx3cr1	0.58	1.3	0.00	1.8	0.02	1.8
A_55_P2178800	NM_201641	Ugt1a10	0.45	1.4	0.04	1.6	0.01	1.8
A_55_P2052485	NM_181418	Ushbp1	0.18	1.4	0.00	1.9	0.00	1.8
A_52_P64707	NM_008260	Foxa3	0.08	1.5	0.00	1.8	0.00	1.8
A_51_P402160	NM_178763	Zfp750	0.74	1.2	0.20	1.4	0.01	1.8
A_51_P255682	NM_008594	Mfge8	0.97	1.0	0.19	1.4	0.00	1.8
A_55_P2061338	NM_153081	Slc16a11	0.49	1.3	0.06	1.5	0.00	1.8
A_51_P375201	NM_013807	Plk3	0.15	1.7	0.02	1.8	0.02	1.8
A_52_P361081	NM_001112744	Arhgef16	0.75	1.3	0.27	1.4	0.02	1.8
A_51_P400366	NM_021375	Rhbg	0.27	1.3	0.28	1.3	0.00	1.8

A_55_P2042813	NM_001039647	EG634650	0.08	2.3	0.00	3.3	0.19	1.8
A_55_P1995074	NM_176902	Fam100b	0.89	1.1	0.08	1.4	0.00	1.8
A_55_P2059010	NM_011254	Rbp1	0.60	1.4	0.18	1.5	0.02	1.8
A_51_P121915	NM_145424	BC089597	0.74	1.2	0.52	1.3	0.01	1.8
A_55_P2127702	NM_012025	Racgap1	0.97	1.1	0.67	1.2	0.02	1.8
A_55_P2066559	NM_001168693	Pp11r	0.83	1.1	0.01	1.6	0.00	1.8
A_51_P189361	NM_027950	Osgin1	0.35	1.4	0.00	1.7	0.01	1.8
A_66_P128293	NM_177391	Fam109b	0.94	1.1	0.94	1.1	0.01	1.8
A_55_P2140107	NM_009995	Cyp21a1	0.42	1.3	0.05	1.5	0.00	1.8
A_55_P2063256	NM_010706	Lgals4	0.68	1.2	0.07	1.5	0.00	1.8
A_55_P2145804	NM_026531	Aen	0.58	1.3	0.06	1.5	0.01	1.8
A_55_P1967539	NM_015755	Hunk	0.70	1.3	0.30	1.4	0.03	1.7
A_55_P2124736	NM_181277	Col14a1	0.52	1.3	0.06	1.5	0.01	1.7
A_55_P2035018	NM_020001	Clec4n	0.76	1.2	0.12	1.4	0.00	1.7
A_55_P2156731	NM_010382	H2-Eb1	0.57	1.3	0.38	1.3	0.01	1.7
A_55_P1981836	NM_145536	BC020535	0.05	1.6	0.00	1.6	0.01	1.7
A_52_P574668	NM_011851	Nt5e	0.79	1.2	0.03	1.7	0.02	1.7
A_55_P2004801	NM_001040435	Tacc3	0.96	1.1	0.54	1.3	0.04	1.7
A_51_P451428	NM_001038602	Marveld2	0.15	1.5	0.00	1.8	0.00	1.7
A_66_P136788	NM_176902	Fam100b	0.93	1.1	0.14	1.3	0.01	1.7
A_30_P01021866			0.89	1.1	0.56	1.2	0.00	1.7
A_51_P240453	NM_133851	Nusap1	0.99	1.0	0.58	1.3	0.03	1.7
A_55_P2158018	NM_001099632	Rnf39	0.68	1.3	0.00	1.8	0.02	1.7
A_52_P111031	NM_001013753	Pcdh17	0.40	1.4	0.01	1.6	0.02	1.7
A_55_P2085955	NM_026400	Dnajb11	0.38	1.5	0.00	1.9	0.03	1.7
A_51_P300506	NM_183405	Cox6b2	0.99	1.0	0.65	1.2	0.02	1.7
A_55_P2105685	NM_013462	Adrb3	0.78	1.2	0.00	1.8	0.01	1.7
A_55_P2185900	NM_032002	Nrg4	0.55	1.4	0.02	1.8	0.05	1.7
A_55_P2015292	NM_008521	Ltc4s	0.26	1.3	0.00	1.8	0.00	1.7
A_55_P2180944	NM_029857	Tmco4	0.96	1.1	0.02	1.8	0.06	1.7
A_51_P430259	NM_001081275	1700009P17Rik	0.24	1.6	0.11	1.5	0.04	1.7
A_55_P2109505	XM_890094	Gm6594	0.47	1.3	0.22	1.3	0.00	1.7
A_55_P2153292	NM_146116	Tubb2c	0.98	-1.0	0.91	-1.1	0.04	1.7
A_55_P2126572	NM_027865	Tmem25	0.46	1.3	0.00	1.6	0.00	1.7
A_52_P211185	NM_008140	Gnat1	0.25	1.6	0.01	1.9	0.04	1.7
A_51_P497100	NM_010706	Lgals4	0.82	1.2	0.12	1.5	0.02	1.7
A_55_P2187034			0.91	1.1	0.63	1.2	0.02	1.7
A_55_P1987499	NM_013917	Pttg1	0.98	1.0	0.55	1.2	0.00	1.7
A_51_P279712	NM_145923	Rell1	0.49	1.3	0.01	1.5	0.00	1.7
A_55_P1982171	NM_016750	H2afz	0.98	-1.0	0.91	1.1	0.00	1.7
A_55_P2387665	AK033690	9130221J18Rik	0.39	1.4	0.05	1.5	0.01	1.7
A_51_P369252	NM_001080995	4632434I11Rik	0.31	1.4	0.04	1.5	0.00	1.7
A_55_P2073219	NM_138646	Hps4	0.45	1.3	0.01	1.6	0.00	1.6
A_55_P2073218	NM_138646	Hps4	0.57	1.2	0.00	1.5	0.01	1.6
A_51_P267933	NM_175692	Snhg11	0.89	1.1	0.20	1.4	0.04	1.6
A_55_P2112225	AK191463		0.80	1.1	0.04	1.4	0.02	1.6
A_51_P237585	NM_172793	Btnl9	0.15	1.5	0.03	1.5	0.01	1.6
A_52_P222073	NM_021609	Ccbp2	0.00	1.6	0.01	1.6	0.00	1.6
A_51_P483118	NM_016660	Hmga1	0.73	1.1	0.02	1.4	0.01	1.6

A_51_P265806	NM_030601	Clca2	0.39	1.5	0.03	1.7	0.07	1.6
A_52_P14456	NM_026886	Srrm4	0.81	1.2	0.08	1.5	0.04	1.6
A_51_P272553	NM_011498	Bhlhe40	0.22	1.5	0.13	1.5	0.04	1.6
A_55_P1974645	NM_009849	Entpd2	0.93	1.1	0.24	1.3	0.02	1.6
A_51_P288916	NM_177368	Tmtc2	0.64	1.3	0.08	1.5	0.04	1.6
A_55_P2123566	AK132916		0.90	1.1	0.15	1.5	0.03	1.6
A_51_P164296	NM_021475	Adamdec1	0.24	1.5	0.02	1.6	0.02	1.6
A_55_P2162404	XM_001477908	LOC100047327	0.77	1.2	0.37	1.3	0.04	1.6
A_51_P371750	NM_010766	Marco	0.13	1.7	0.00	1.9	0.06	1.6
A_51_P250807	NM_030176	Spata2L	0.75	1.2	0.15	1.5	0.03	1.6
A_55_P2182705	NM_010655	Kpna2	0.95	1.1	0.87	1.1	0.01	1.6
A_51_P482711	NM_053272	Dhcr24	0.91	-1.1	0.68	1.2	0.02	1.6
A_55_P1996578	NM_008010	Fgfr3	0.61	1.2	0.02	1.4	0.00	1.6
A_55_P2123471	NM_010655	Kpna2	0.97	1.0	0.98	1.0	0.02	1.6
A_51_P344566	NM_011121	Plk1	0.99	-1.0	0.92	1.1	0.04	1.6
A_55_P2028054	NM_016692	Incenp	0.96	1.1	0.69	1.2	0.03	1.6
A_55_P2094019	NM_177016	Slc17a4	0.29	1.4	0.29	1.3	0.01	1.6
A_55_P1994032	NM_013842	Xbp1	0.30	1.3	0.00	1.6	0.00	1.6
A_66_P128434	NM_001122733	Kit	0.05	1.6	0.01	1.6	0.01	1.6
A_55_P2037712	NM_133786	Smc4	0.98	1.0	0.54	1.2	0.02	1.6
A_55_P2012799	NM_133641	Rtkn	0.70	1.2	0.01	1.5	0.00	1.6
A_55_P1975475	NM_010786	Mdm2	0.88	1.1	0.32	1.2	0.00	1.6
A_51_P444437	NM_025968	Ptgr1	0.51	1.3	0.22	1.4	0.03	1.6
A_66_P136186	NM_009516	Wee1	0.76	1.2	0.00	1.8	0.09	1.6
A_30_P01026611			0.85	1.1	0.49	1.2	0.01	1.6
A_55_P2152225	NM_010544	Ihh	0.40	1.4	0.13	1.5	0.04	1.6
A_55_P2011678	NM_001164557	Pdzk1ip1	0.84	1.2	0.14	1.4	0.02	1.6
A_55_P2084706	BC023946	Acaca	0.59	1.2	0.16	1.3	0.01	1.6
A_51_P495269	NM_008508	Lor	0.73	1.2	0.29	1.3	0.02	1.6
A_55_P1978201	NM_016692	Incenp	0.97	1.0	0.65	1.2	0.03	1.5
A_55_P2084631	NM_178184	Hist1h2an	0.89	1.1	0.37	1.2	0.01	1.5
A_51_P177171	NM_011587	Tie1	0.07	1.4	0.02	1.5	0.01	1.5
A_65_P16680	NM_019835	B4galt5	0.54	1.3	0.53	1.2	0.04	1.5
A_52_P547612	NM_178715	Tmem30b	0.00	1.6	0.02	1.4	0.01	1.5
A_55_P2303972	AK077973	D830044D21Rik	0.82	1.2	0.03	1.6	0.07	1.5
A_52_P608322	NM_010755	Maff	0.48	1.3	0.04	1.6	0.03	1.5
A_51_P446825	NM_176952	6430573F11Rik	0.81	1.2	0.38	1.3	0.01	1.5
A_51_P315904	NM_011817	Gadd45g	0.11	2.2	0.00	3.1	0.45	1.5
A_30_P01031894			0.85	1.1	0.13	1.4	0.04	1.5
A_51_P155503	NM_008457	Klk1b8	0.35	1.4	0.21	1.4	0.03	1.5
A_55_P2152074	NM_018737	Ctps2	0.95	1.1	0.16	1.3	0.00	1.5
A_51_P108020	NM_001029929	Zmynd15	0.41	1.2	0.00	1.5	0.01	1.5
A_55_P2078459	NM_010655	Kpna2	0.88	1.1	0.93	1.0	0.01	1.5
A_52_P194851	NM_001162906	2410089E03Rik	0.95	1.1	0.25	1.3	0.03	1.5
A_55_P2028600	NM_022410	Myh9	0.98	-1.0	0.46	1.1	0.00	1.5
A_66_P105843	NM_011181	Cyth2	0.82	1.1	0.20	1.2	0.00	1.5
A_52_P238019	NM_001038230	Anapc11	0.55	1.2	0.46	1.1	0.00	1.5
A_30_P01025742			0.00	1.4	0.00	1.5	0.00	1.5
A_30_P01033174			0.82	1.1	0.12	1.4	0.03	1.5

A_55_P2005426	NM_019445	Fmn2	0.43	1.3	0.39	1.2	0.02	1.5
A_51_P341465	NM_009970	Csf2ra	0.93	1.1	0.28	1.3	0.00	1.5
A_66_P116451	XM_001476411	2210039B01Rik	0.76	1.2	0.11	1.4	0.04	1.5
A_51_P103397	NM_011708	Vwf	0.51	1.3	0.12	1.4	0.02	1.5
A_55_P2136732	NM_025584	Cd99	0.92	1.1	0.13	1.4	0.03	1.5
A_55_P2177658	NM_019945	Mast1	0.27	1.4	0.00	1.6	0.05	1.5
A_55_P2243431	BC052902	Gdap10	0.58	1.4	0.03	1.7	0.14	1.5
A_52_P480402	NM_030235	Avl9	0.86	1.1	0.39	1.2	0.02	1.5
A_52_P503730	NM_199012	Fchsd2	0.07	1.5	0.16	1.4	0.04	1.5
A_51_P448391	NM_023526	Nkiras1	0.46	1.3	0.57	1.2	0.02	1.5
A_55_P2027836	NM_020275	Tnfrsf10b	0.95	1.1	0.46	1.2	0.02	1.5
A_55_P2061737	NM_021278	Tmsb4x	0.93	1.1	0.28	1.3	0.04	1.5
A_52_P117352	NM_008120	Gja4	0.70	1.2	0.31	1.3	0.03	1.5
A_51_P500344	NM_145569	Mat2a	0.82	1.1	0.47	1.2	0.03	1.5
A_30_P01024578			0.79	1.1	0.13	1.3	0.02	1.5
A_55_P2023542	NM_007719	Ccr7	0.96	1.0	0.11	1.3	0.02	1.5
A_55_P2082688	NM_021515	Ak1	0.98	-1.0	0.49	1.2	0.01	1.5
A_55_P2111355	NM_178444	Egfl7	0.76	1.1	0.00	1.4	0.00	1.5
A_55_P2120254	NM_027106	Avpi1	0.95	1.1	0.62	1.2	0.02	1.5
A_30_P01022030			0.05	2.2	0.00	2.4	0.29	1.5
A_51_P450573	NM_009371	Tgfbr2	0.65	1.2	0.02	1.4	0.00	1.5
A_55_P1954092	XM_001473985	Gm2563	0.79	1.1	0.28	1.3	0.00	1.5
A_55_P2072666	BC059104		0.25	1.4	0.12	1.4	0.02	1.5
A_52_P604629	NM_153287	Csrnp1	0.00	1.6	0.00	1.7	0.03	1.5
A_51_P401343	NM_019500	Cldn14	0.44	1.3	0.01	1.5	0.02	1.5
A_51_P143893	NM_054098	Steap4	0.54	1.2	0.32	1.2	0.01	1.5
A_30_P01023033			0.15	1.3	0.04	1.4	0.00	1.5
A_51_P229911	NM_080435	Adcy4	0.00	1.4	0.18	1.3	0.00	1.5
A_55_P2127179	XM_142052	Gm379	0.77	1.2	0.84	1.1	0.03	1.5
A_55_P2054362	XM_001472585	LOC100048875	0.87	1.1	0.25	1.4	0.04	1.5
A_52_P612803	NM_009831	Ccng1	0.20	1.5	0.02	1.6	0.06	1.5
A_55_P2178084	NM_007872	Dnmt3a	0.38	1.3	0.00	1.5	0.01	1.5
A_51_P360918	NM_020578	Ehd3	0.89	1.1	0.66	1.2	0.03	1.5
A_51_P466829	NM_175474	Fam109a	0.56	1.2	0.08	1.3	0.00	1.5
A_51_P436878	NM_018820	Sertad1	0.42	1.3	0.02	1.5	0.02	1.5
A_51_P238722	NM_010740	Cd93	0.57	1.2	0.01	1.5	0.03	1.4
A_55_P2058957	NM_009306	Syt1	0.63	1.2	0.03	1.5	0.05	1.4
A_55_P1984307	NM_001081178	Gpr116	0.36	1.3	0.00	1.5	0.06	1.4
A_55_P2125811	NM_027102	Esam	0.49	1.3	0.02	1.5	0.09	1.4
A_51_P124535	NM_008590	Mest	0.48	1.3	0.04	1.5	0.12	1.4
A_55_P2019690	NM_001081349	Slc43a1	0.30	1.3	0.00	1.5	0.08	1.4
A_55_P2133632	NM_001081337	Sipa1l2	0.53	1.2	0.00	1.5	0.03	1.4
A_30_P01022612			0.52	1.3	0.03	1.5	0.04	1.4
A_30_P01027115			0.57	1.2	0.00	1.5	0.04	1.4
A_55_P2121456	NM_177632	Fam43a	0.64	1.2	0.00	1.5	0.02	1.4
A_52_P99888	NM_023158	Cxcl16	0.09	1.4	0.00	1.5	0.03	1.4
A_55_P2165560	NM_001004156	Plekhg5	0.42	1.2	0.00	1.5	0.00	1.4
A_30_P01022537			0.59	1.3	0.04	1.5	0.16	1.4
A_51_P502054	NM_145546	Gtf2b	0.30	1.3	0.00	1.5	0.03	1.4

A_55_P2104975	NM_001168294	Serpina3f	0.25	1.5	0.03	1.6	0.12	1.4
A_51_P472274	NM_009236	Sox18	0.22	1.3	0.01	1.5	0.03	1.4
A_66_P105689	NM_030684	Trim34	0.79	1.3	0.03	1.8	0.34	1.4
A_51_P346668	NM_012057	Irf5	0.81	1.2	0.01	1.7	0.24	1.4
A_55_P2105638	NM_001033348	Ralgapa2	0.85	1.1	0.04	1.5	0.09	1.4
A_51_P195958	NM_009344	Phlda1	0.07	2.4	0.00	2.9	0.63	1.4
A_51_P137452	NM_013809	Cyp2g1	0.18	1.7	0.01	2.0	0.41	1.4
A_30_P01020303			0.00	2.5	0.00	2.5	0.45	1.4
A_52_P298002	NM_008102	Gch1	0.33	1.4	0.02	1.5	0.12	1.4
A_52_P326354	NM_010155	Erf	0.79	1.1	0.00	1.5	0.03	1.4
A_52_P485850	NM_001171003	Mgam	0.23	1.3	0.02	1.5	0.08	1.4
A_51_P212420	NM_010681	Lama4	0.09	1.4	0.00	1.5	0.02	1.4
A_55_P2046509	NM_153319	Amot	0.88	1.1	0.01	1.5	0.05	1.4
A_55_P2114318	XM_001478955	Gm4080	0.72	1.2	0.01	1.6	0.20	1.3
A_55_P2043182	NM_153175	Gimap6	0.11	1.5	0.01	1.5	0.14	1.3
A_51_P455997	NR_027652	Meg3	0.60	1.3	0.03	1.6	0.44	1.3
A_30_P01024256			0.05	2.0	0.00	2.4	0.59	1.3
A_51_P329949	NM_153574	Fam13a	0.13	1.7	0.00	1.8	0.51	1.3
A_51_P378298	NM_026976	Faim3	0.68	1.1	0.01	1.5	0.04	1.3
A_55_P2011387	NM_145133	Tifa	0.57	1.2	0.03	1.5	0.26	1.3
A_55_P1983769	NM_001012273	Birc5	0.95	1.1	0.02	1.5	0.12	1.3
A_55_P1976978	NM_001081065	Zfp707	0.80	1.1	0.01	1.5	0.08	1.3
A_55_P2140151	NM_146018	Flcn	0.36	1.3	0.00	1.6	0.15	1.3
A_55_P2072925			0.24	1.6	0.00	1.9	0.75	1.2
A_55_P2046842	NM_001033805	G630016D24Rik	0.95	1.1	0.01	1.5	0.61	1.2
A_30_P01021853			0.41	1.5	0.04	1.8	0.76	1.2
A_51_P474459	NM_007707	Socs3	0.51	1.3	0.01	1.7	0.47	1.2
A_55_P2126192	NM_010195	Lgr5	0.00	1.7	0.38	1.3	0.61	1.2
A_66_P134394	BC027050	Pde6c	0.99	-1.0	0.02	1.5	0.56	1.2
A_30_P01033600			0.24	1.8	0.04	1.9	0.91	1.1
A_30_P01027314			0.50	1.7	0.02	2.6	0.98	1.0
A_55_P2335718	AK086235	Wdr45l	0.41	1.4	0.04	1.6	0.97	-1.0
A_51_P279437	NM_029662	Mfsd2a	0.50	-1.6	0.01	-2.1	0.89	-1.1
A_55_P2100241	NR_003278	Rn18s	0.00	-1.8	0.57	-1.2	0.84	-1.1
A_55_P2069579	XM_001475771	LOC100046200	0.57	-1.2	0.00	-1.5	0.14	-1.2
A_30_P01021428			0.91	-1.1	0.00	-2.4	0.71	-1.2
A_55_P2181928	NM_145977	Slc45a3	0.15	-1.4	0.00	-1.6	0.30	-1.2
A_52_P28651	NM_021424	Pvrl1	0.55	-1.2	0.01	-1.5	0.23	-1.3
A_55_P2104259	NM_001077348	Plin5	0.85	-1.1	0.04	-1.5	0.17	-1.3
A_51_P333111	NM_009676	Aox1	0.47	-1.4	0.04	-1.6	0.46	-1.3
A_51_P349727	NM_134154	Slc25a45	0.83	-1.1	0.00	-1.5	0.16	-1.3
A_55_P2027213	NM_001163615	Krtap20-2	0.09	-1.4	0.00	-1.5	0.01	-1.4
A_30_P01032549			0.97	-1.1	0.02	-1.8	0.30	-1.4
A_51_P421876	NM_016850	Irf7	0.13	-1.5	0.00	-1.6	0.08	-1.4
A_30_P01018959			0.45	-1.3	0.00	-1.6	0.09	-1.4
A_30_P01018039			0.00	-1.6	0.16	-1.3	0.04	-1.4
A_55_P2083649	NM_020559	Alas1	0.50	-1.6	0.00	-2.4	0.53	-1.4
A_55_P2305420	AK038263	D9Wsu90e	0.07	-1.9	0.00	-2.1	0.29	-1.4
A_55_P1961210	NM_178111	Trp53inp2	0.89	-1.1	0.01	-1.5	0.07	-1.4

A_51_P344376	NM_013533	Gpr162	0.70	-1.2	0.04	-1.5	0.06	-1.4
A_30_P01021970			0.60	-1.2	0.51	-1.2	0.01	-1.5
A_66_P109802	NM_001159415	Gm4738	0.96	1.0	0.79	-1.1	0.03	-1.5
A_55_P1967133	NM_177741	Ppp1r3b	0.38	-1.3	0.08	-1.4	0.02	-1.5
A_55_P2051486	NM_001012323	Mup20	0.85	-1.1	0.18	-1.3	0.02	-1.5
A_51_P350453	NM_013743	Pdk4	0.51	-1.5	0.03	-1.9	0.18	-1.5
A_66_P126877	NM_134214	V1rh5	0.57	-1.3	0.09	-1.4	0.02	-1.5
A_30_P01022987			0.00	-1.5	0.04	-1.4	0.03	-1.5
A_51_P239737	NM_011082	Pigr	0.75	-1.2	0.83	-1.1	0.01	-1.5
A_51_P389539	NM_054053	Gpr98	0.55	-1.4	0.00	-1.9	0.15	-1.5
A_55_P2010097	NM_001045550	Mup2	0.95	1.1	0.46	-1.2	0.00	-1.5
A_55_P2238059	AK043390	A730091E23Rik	0.99	1.0	0.39	-1.3	0.01	-1.5
A_55_P2091350	AK140187	ND4L	0.99	-1.0	0.32	-1.2	0.00	-1.5
A_52_P493620	NM_026218	Fgfr1op2	0.56	-1.3	0.02	-1.6	0.05	-1.5
A_55_P1987811	XM_001474233	Gm2724	0.55	-1.3	0.01	-1.6	0.03	-1.5
A_52_P405206	NM_026281	Tm7sf3	0.49	-1.3	0.01	-1.6	0.07	-1.5
A_52_P467930	XM_001000802	Prdx6-rs2	0.63	-1.2	0.27	-1.3	0.01	-1.5
A_55_P2060343	NM_153102	Zfp352	0.07	-1.5	0.02	-1.5	0.03	-1.5
A_51_P503625	NM_001077353	Gsta3	0.31	-1.3	0.11	-1.4	0.03	-1.5
A_55_P2058467	XM_001479915	Gm4415	0.54	-1.2	0.19	-1.2	0.00	-1.5
A_55_P2031100	NM_016661	Ahcy	0.80	-1.1	0.17	-1.3	0.01	-1.5
A_66_P103271	NM_001164598	Irf2bp2	0.00	-1.5	0.06	-1.4	0.03	-1.5
A_52_P176160	NM_016661	Ahcy	0.14	-1.4	0.22	-1.3	0.01	-1.5
A_51_P480982	NM_025374	Glo1	0.84	-1.1	0.31	-1.2	0.00	-1.5
A_30_P01020013			0.88	-1.1	0.57	-1.2	0.01	-1.5
A_52_P30451	NM_016854	Ppp1r3c	0.75	-1.2	0.00	-1.6	0.04	-1.5
A_55_P2103963	NM_016668	Bhmt	0.73	-1.1	0.24	-1.2	0.00	-1.5
A_51_P516728	NM_010404	Hap1	0.08	-1.4	0.01	-1.4	0.01	-1.5
A_30_P01018128			0.42	-1.4	0.04	-1.6	0.07	-1.5
A_51_P117618	NM_023154	Ethe1	1.00	-1.0	0.93	1.0	0.02	-1.5
A_55_P1960291	NM_013769	Tjp3	0.48	-1.3	0.01	-1.5	0.02	-1.5
A_55_P1993419	NM_206537	Cyp2c54	0.54	-1.3	0.60	-1.2	0.03	-1.5
A_51_P431737	NM_145953	Cth	0.88	1.1	0.70	-1.1	0.02	-1.5
A_66_P112862	NR_033146	LOC100316870	0.13	-1.3	0.01	-1.3	0.00	-1.5
A_55_P2171206	NM_013592	Matn4	0.47	-1.3	0.06	-1.4	0.02	-1.5
A_55_P1977653	NM_001081390	Palld	0.84	-1.1	0.47	-1.2	0.04	-1.5
A_55_P2050192	AK142726	ND5	0.97	-1.0	0.13	-1.2	0.00	-1.5
A_52_P286360	NM_008769	Otc	0.98	-1.0	0.79	-1.1	0.01	-1.5
A_55_P2029061	NM_011612	Tnfrsf9	0.88	-1.1	0.49	-1.2	0.04	-1.5
A_30_P01022386			0.83	-1.2	0.58	-1.2	0.03	-1.5
A_51_P189442	NM_011996	Adh4	0.55	-1.3	0.40	-1.2	0.03	-1.5
A_55_P2125947	NM_026212	Agpat2	0.57	-1.2	0.00	-1.4	0.01	-1.5
A_51_P236324	NM_028812	Gtf2e1	0.66	-1.2	0.06	-1.3	0.00	-1.5
A_55_P2047188	NM_010197	Fgf1	0.86	-1.1	0.13	-1.3	0.00	-1.5
A_55_P2183208	NM_001045532	Prl2c1	0.15	-1.3	0.00	-1.4	0.00	-1.5
A_52_P413947	NM_010840	Mthfr	0.74	-1.2	0.03	-1.6	0.11	-1.5
A_55_P2189893	AK042233	A630073K07Rik	0.70	-1.2	0.27	-1.3	0.04	-1.5
A_51_P483544	NM_013930	Aass	0.92	-1.1	0.76	-1.1	0.00	-1.6
A_52_P614777	NM_032400	Sucnr1	0.98	-1.0	0.79	-1.1	0.03	-1.6

A_51_P257885	NM_175217	Mmd2	0.88	-1.1	0.76	-1.2	0.04	-1.6
A_55_P1959973	NM_175539	Dcaf12l2	0.72	-1.1	0.19	-1.2	0.00	-1.6
A_55_P1977776	NR_003280	LOC790956	0.12	-1.5	0.36	-1.3	0.03	-1.6
A_51_P228574	NM_146214	Tat	0.97	-1.0	0.57	-1.2	0.02	-1.6
A_55_P1993049	XM_001478943	Gm4076	0.94	-1.1	0.00	-1.3	0.00	-1.6
A_51_P174645	NM_133768	Asl	0.77	-1.2	0.73	-1.1	0.00	-1.6
A_30_P01021789			0.80	1.2	0.62	-1.2	0.04	-1.6
A_30_P01030307			0.05	-1.6	0.00	-1.7	0.00	-1.6
A_51_P326685	NM_176920	Lrtm1	0.84	-1.2	0.20	-1.4	0.04	-1.6
A_55_P2039429	NM_007860	Dio1	0.66	-1.4	0.02	-2.0	0.13	-1.6
A_55_P2010672	NM_010623	Kif17	0.76	-1.2	0.14	-1.4	0.02	-1.6
A_55_P2169259	NM_019792	Cyp3a25	0.91	-1.1	0.35	-1.3	0.03	-1.6
A_30_P01032670			0.12	-1.4	0.02	-1.4	0.00	-1.6
A_55_P2142222	NM_001034870	Serpina3h	1.00	1.0	0.02	-1.7	0.06	-1.6
A_30_P01027977			0.91	-1.1	0.39	-1.3	0.03	-1.6
A_55_P2017998			0.22	-1.5	0.00	-2.0	0.05	-1.6
A_66_P106611	NM_008137	Gna14	0.91	-1.1	0.13	-1.4	0.01	-1.7
A_55_P2077783	NR_003964	Tubb2a-ps2	0.27	-1.4	0.00	-1.7	0.02	-1.7
A_55_P2000938	BC086781	Ahcy	0.88	-1.1	0.57	-1.2	0.01	-1.7
A_55_P2160686	NM_009366	Tsc22d1	0.84	-1.2	0.08	-1.6	0.03	-1.7
A_51_P341918	NM_009366	Tsc22d1	0.80	-1.2	0.00	-1.7	0.03	-1.7
A_66_P124715	NM_011145	Ppard	0.57	-1.4	0.07	-1.6	0.04	-1.7
A_51_P440743	NM_009886	Celsr1	0.48	-1.4	0.01	-1.7	0.02	-1.7
A_55_P2035320	NM_017373	Nfil3	0.32	-1.5	0.03	-1.7	0.03	-1.7
A_55_P1989061	NM_001077364	Tsc22d3	0.18	-1.6	0.03	-1.6	0.01	-1.7
A_55_P2146655	XM_001472967	Gm2262	0.05	-1.6	0.00	-1.7	0.00	-1.7
A_55_P1978770	XM_973294	E030018B13Rik	0.65	-1.3	0.03	-1.7	0.03	-1.7
A_55_P1971674	NM_001141983	Golga7b	0.88	-1.1	0.77	-1.2	0.00	-1.7
A_55_P2142724	XM_001472454	Gm16483	0.82	-1.2	0.27	-1.3	0.02	-1.7
A_51_P117881	NM_153069	Leap2	0.63	-1.3	0.16	-1.5	0.02	-1.7
A_55_P2003483	NM_138595	Gldc	0.76	-1.2	0.60	-1.2	0.00	-1.7
A_55_P2086433	NM_145209	Oasl1	0.00	-1.5	0.00	-1.8	0.00	-1.7
A_30_P01019010			0.55	-1.3	0.03	-1.5	0.00	-1.7
A_52_P412506	NM_008649	Mup5	0.99	-1.0	0.95	-1.0	0.00	-1.7
A_51_P327451	NM_009653	Alas2	0.96	-1.1	0.78	-1.1	0.00	-1.8
A_30_P01021387			0.00	-1.6	0.01	-1.8	0.00	-1.8
A_30_P01018444			0.44	1.4	0.79	-1.1	0.01	-1.8
A_51_P490023	NM_009450	Tubb2a	0.26	-1.7	0.04	-1.9	0.07	-1.8
A_51_P316935	NM_145980	8430408G22Rik	0.32	-1.5	0.02	-1.8	0.02	-1.9
A_55_P2057430	NM_027340	Lipn	0.99	1.0	0.50	-1.3	0.02	-1.9
A_55_P2076196	NM_001134644	CU041261.1	0.98	1.0	0.74	-1.2	0.01	-1.9
A_51_P334942	NM_013467	Aldh1a1	0.23	-1.4	0.00	-1.6	0.00	-1.9
A_55_P2162160	NM_009672	Anp32a	0.13	-1.7	0.36	-1.4	0.03	-1.9
A_55_P2033321	AW012817		0.97	-1.1	0.85	-1.1	0.00	-2.0
A_30_P01017959			0.99	1.0	0.53	-1.4	0.01	-2.1
A_51_P268529	NM_144942	Csad	0.14	-1.6	0.00	-1.9	0.00	-2.1
A_30_P01024647			0.67	1.4	0.52	-1.4	0.01	-2.1
A_30_P01030756			0.41	-1.7	0.16	-1.8	0.04	-2.1
A_55_P2043083	NM_177406	Cyp4a12a	0.97	-1.1	0.98	-1.0	0.01	-2.1

A_52_P402127	NM_001126319	Mup9	0.74	1.4	0.98	-1.0	0.03	-2.1
A_55_P2116674			0.81	1.2	0.89	-1.1	0.00	-2.2
A_55_P2113587			0.92	1.1	0.73	-1.2	0.00	-2.3
A_52_P327156	NR_027970	0610008F07Rik	0.40	-1.5	0.10	-1.6	0.01	-2.3
A_55_P2076832	NM_146150	Nrd1	0.15	-2.1	0.14	-1.9	0.02	-2.3
A_55_P2111980	NM_008295	Hsd3b5	0.95	-1.1	0.98	-1.0	0.00	-2.3
A_51_P403477	NM_007860	Dio1	0.87	-1.2	0.02	-2.2	0.01	-2.4
A_55_P2065449	NM_152895	Kdm5b	0.22	-2.3	0.16	-2.1	0.04	-2.5
A_55_P2103703	XM_001475400	LOC635091	0.58	1.5	0.93	-1.1	0.01	-2.6
A_51_P501844	NM_175475	Cyp26b1	0.00	-3.5	0.07	-2.0	0.01	-2.6
A_55_P2046671	XM_001477211	CU104690.1	0.72	1.3	0.57	-1.3	0.00	-2.6
A_55_P2075127	NM_011037	Pax2	0.96	1.1	0.93	-1.1	0.00	-2.8
A_55_P2055423	XM_485799	Nat8b	0.85	-1.3	0.90	1.2	0.02	-2.8
A_55_P2061104	NM_001081285	Mup6	0.99	1.0	0.25	-1.5	0.00	-4.6
A_51_P493987	NM_021509	Moxd1	0.88	-1.4	0.00	-6.9	0.00	-7.1

3.2.3 BghiP Liver. Significant probe list. List of all significantly differentially expressed probes in at least 1 treatment group (FDR P ≤ 0.05, fold change ± 1.5) in response to sub-chronic oral exposure to 6.25, 12.5, and 25 mg/kg-bw/day benzo(ghi)perylene in the liver. The list is sorted from highest to lowest fold change in the 25 mg/kg-bw/day treatment group.

			**6.25 mg/kg-bw/day**		**12.5 mg/kg-bw/day**		**25 mg/kg-bw/day**	
Agilent Probe	Accession Number	Gene Symbol	*FDR P value*	*Fold change*	*FDR P value*	*Fold change*	*FDR P value*	*Fold change*
A_51_P137452	NM_013809	Cyp2g1	1.00	1.3	0.00	3.2	0.16	2.2
A_55_P1992849	NM_013462	Adrb3	0.57	1.6	0.95	1.3	0.01	2.0
A_51_P267933	NM_175692	Snhg11	1.00	1.4	0.46	1.7	0.04	1.9
A_51_P478098	NM_001113416	Epb4.1l5	1.00	1.2	0.97	1.2	0.03	1.8
A_51_P137604	NM_007995	Fcna	1.00	1.2	0.96	1.3	0.04	1.8
A_51_P478952	NM_133898	N4bp2l1	1.00	1.3	0.93	1.4	0.03	1.8
A_55_P2157872	NM_001079695	Sfrs5	1.00	1.2	0.97	1.3	0.02	1.8
A_55_P2105685	NM_013462	Adrb3	0.38	1.6	0.97	1.3	0.01	1.7
A_55_P2116978	NM_010889	Neb	0.98	1.3	0.71	1.4	0.00	1.7
A_52_P18068	NM_178935	4932441K18Rik	1.00	1.2	0.88	1.2	0.01	1.6
A_51_P108581	NM_177078	Adrbk2	1.00	1.1	0.91	1.2	0.01	1.6
A_55_P2109479	NM_001001180	BC066028	1.00	1.2	0.54	1.4	0.04	1.6
A_51_P241995	NM_016919	Col5a3	0.48	1.5	0.69	1.4	0.03	1.6
A_55_P1967168	NM_172668	Lrp4	1.00	1.2	0.77	1.3	0.02	1.6
A_52_P140881	NM_177615	Slc26a10	0.87	1.3	0.48	1.4	0.01	1.6
A_55_P1972527	NM_178935	4932441K18Rik	1.00	1.2	0.82	1.2	0.00	1.5
A_52_P373893	AK154578	Acvr1b	0.66	1.2	0.39	1.3	0.00	1.5
A_55_P1982291	NM_009899	Clca1	1.00	1.2	0.88	1.3	0.01	1.5
A_52_P331212	NM_026493	Cspp1	1.00	1.1	0.48	1.3	0.00	1.5
A_52_P217710	NM_008056	Fzd6	1.00	1.2	0.97	1.2	0.01	1.5
A_51_P176387	NM_207659	Hook3	1.00	1.1	0.99	1.1	0.03	1.5
A_52_P550147	AJ584850	Sned1	0.43	1.3	0.23	1.3	0.00	1.5
A_51_P264634	NM_009261	Strbp	1.00	1.1	0.94	1.2	0.00	1.5
A_55_P1962747	NM_207105	H2-Ab1	1.00	1.1	0.00	1.8	0.31	1.4

A_55_P2024953	NM_001081278	Tbc1d4	0.00	1.5	1.00	1.0	0.23	1.3
A_51_P470715	NM_009895	Cish	0.00	-5.1	1.00	1.1	0.95	1.2
A_55_P1979973	NM_028778	Nuak2	0.00	1.5	0.26	1.3	0.37	1.2
A_52_P381430	NM_001081278	Tbc1d4	0.00	1.7	1.00	1.0	0.55	1.2
A_52_P455404	NM_025922	Itpa	1.00	1.4	0.00	-2.9	0.94	1.1
A_55_P1963483	NM_030559	Vps16	1.00	1.3	0.00	-3.4	0.91	1.1
A_52_P528600	NM_008245	Hhex	0.00	-2.3	0.97	-1.4	0.78	-1.2
A_30_P01018959			1.00	-1.0	0.00	-1.6	0.11	-1.4
A_52_P432580	XM_001473421	LOC100044968	0.00	-2.2	0.97	-1.3	0.49	-1.4
A_30_P01028215			1.00	-1.1	0.77	-1.3	0.04	-1.5
A_52_P272534			1.00	-1.1	0.89	-1.2	0.00	-1.5
A_30_P01025659			1.00	-1.1	0.81	-1.3	0.04	-1.5
A_30_P01028735			1.00	-1.1	0.77	-1.3	0.00	-1.5
A_30_P01030969			1.00	-1.2	0.96	-1.2	0.02	-1.5
A_30_P01021097			1.00	-1.2	1.00	-1.0	0.01	-1.5
A_30_P01031014			1.00	-1.0	0.54	-1.3	0.01	-1.5
A_30_P01027388			1.00	-1.1	0.88	-1.3	0.03	-1.5
A_30_P01019829			1.00	-1.1	0.90	-1.2	0.02	-1.5
A_30_P01021078			1.00	-1.1	0.73	-1.3	0.01	-1.5
A_55_P2345116	AK051328	1700109K24Rik	1.00	-1.2	0.82	-1.3	0.02	-1.5
A_55_P2402134	AK078921	9130020K20Rik	1.00	-1.2	0.69	-1.2	0.00	-1.5
A_55_P2225470	AK039069	A230092J17Rik	1.00	-1.2	0.82	-1.3	0.03	-1.5
A_55_P2375121	AK045926	AI225934	1.00	-1.1	0.64	-1.3	0.00	-1.5
A_51_P400453	NM_177096	B430203M17Rik	1.00	-1.1	0.79	-1.2	0.01	-1.5
A_55_P2076797	NM_007840	Ddx5	1.00	-1.0	0.96	-1.2	0.00	-1.5
A_55_P2076984	NM_134082	Farp1	1.00	1.0	0.98	-1.1	0.01	-1.5
A_55_P2026340	NM_001161765	Fmo5	1.00	-1.1	0.79	-1.3	0.04	-1.5
A_55_P1955715	NM_001164689	Gm6710	1.00	-1.2	0.97	-1.2	0.02	-1.5
A_51_P239750	NM_008380	Inhba	0.74	-1.3	0.53	-1.3	0.01	-1.5
A_55_P1957593	NM_023547	Ino80b	1.00	-1.1	0.89	-1.2	0.01	-1.5
A_55_P2129826	NM_178641	Inpp5f	1.00	-1.0	0.80	-1.3	0.00	-1.5
A_55_P2408415	NR_001461	Kcnq1ot1	1.00	1.0	0.97	-1.1	0.00	-1.5
A_55_P2001238	NM_026313	Luc7l3	1.00	-1.1	0.71	-1.3	0.00	-1.5
A_55_P2081388	NM_008575	Mdm4	1.00	-1.2	0.63	-1.4	0.04	-1.5
A_51_P150521	NM_146035	Mgat2	1.00	-1.1	0.97	-1.1	0.00	-1.5
A_55_P2027906	NM_021552	Nsa2	1.00	-1.0	0.97	-1.2	0.00	-1.5
A_51_P503192	NM_175402	Rbm15b	1.00	-1.1	0.97	-1.2	0.02	-1.5
A_52_P393306	NM_009222	Snap23	1.00	-1.1	0.92	-1.2	0.01	-1.5
A_51_P328968	NM_172586	Zfp322a	1.00	-1.2	0.84	-1.2	0.00	-1.5
A_30_P01018686			1.00	-1.1	0.69	-1.3	0.02	-1.6
A_30_P01018620			1.00	-1.2	0.55	-1.4	0.00	-1.6
A_30_P01019815			1.00	-1.1	0.74	-1.3	0.01	-1.6
A_30_P01026106			1.00	-1.1	0.69	-1.4	0.02	-1.6
A_30_P01029653			1.00	-1.2	0.80	-1.4	0.03	-1.6
A_30_P01018010			1.00	-1.1	0.82	-1.3	0.01	-1.6
A_30_P01030038			1.00	-1.0	0.97	-1.2	0.01	-1.6
A_30_P01031627			1.00	-1.2	0.81	-1.3	0.00	-1.6
A_30_P01027833			1.00	-1.2	0.63	-1.4	0.03	-1.6
A_55_P2169124	AK040781		1.00	-1.0	0.97	-1.2	0.00	-1.6

A_30_P01021775			1.00	-1.1	0.57	-1.4	0.03	-1.6
A_30_P01024081			1.00	-1.1	0.86	-1.3	0.04	-1.6
A_30_P01030943			1.00	-1.1	0.77	-1.4	0.02	-1.6
A_55_P2244677	AK032738	6720422M22Rik	1.00	-1.2	0.89	-1.3	0.03	-1.6
A_55_P2189893	AK042233	A630073K07Rik	1.00	-1.2	0.97	-1.3	0.03	-1.6
A_55_P2430472	NM_011805	Dido1	1.00	1.0	0.77	-1.3	0.00	-1.6
A_55_P2076418	NM_001110327	Dmtf1	1.00	-1.1	0.98	-1.2	0.00	-1.6
A_66_P140533	XM_001478088	Gm3813	1.00	-1.2	0.87	-1.3	0.00	-1.6
A_52_P86693	NM_026790	Ifi27l1	0.97	-1.3	0.50	-1.4	0.00	-1.6
A_55_P2104798	XM_001480269	LOC100044040	1.00	1.0	0.96	-1.2	0.00	-1.6
A_55_P2077866	NM_010918	Nktr	1.00	-1.0	0.97	-1.2	0.01	-1.6
A_52_P134023	NM_153100	Rtp3	1.00	-1.0	0.26	-1.6	0.01	-1.6
A_55_P2462940	NM_001081008	Taf1	1.00	-1.1	0.97	-1.1	0.00	-1.6
A_55_P2123716	NR_002321	Tug1	1.00	-1.1	0.99	-1.1	0.00	-1.6
A_55_P1986743			1.00	-1.2	0.46	-1.4	0.00	-1.7
A_30_P01028562			1.00	-1.2	0.63	-1.4	0.00	-1.7
A_55_P1977875	NM_176849	Arglu1	1.00	-1.1	0.61	-1.3	0.00	-1.7
A_55_P2148400	XM_001472970	Gm2264	1.00	1.0	0.97	-1.3	0.04	-1.7
A_55_P1962602	XM_001474429	Gm2690	1.00	-1.0	0.92	-1.3	0.02	-1.7
A_55_P1987811	XM_001474233	Gm2724	1.00	1.1	0.59	-1.5	0.03	-1.7
A_55_P2149821	NM_026989	Sfrs11	1.00	1.0	0.96	-1.2	0.00	-1.7
A_55_P2006662	BC124559		1.00	-1.2	0.97	-1.2	0.00	-1.8
A_30_P01021604			1.00	-1.2	0.60	-1.4	0.02	-1.8
A_55_P1962603	NM_009946	Cplx2	1.00	-1.1	0.91	-1.3	0.00	-1.8
A_55_P2430221	NM_029389	Fam35a	1.00	1.0	0.94	-1.3	0.00	-1.8
A_55_P2201454	AK142062	2310079F09Rik	1.00	-1.2	0.43	-1.5	0.00	-1.9
A_30_P01022120			1.00	-1.3	0.99	-1.1	0.01	-2.0
A_51_P255295	NM_013485	C9	1.00	-1.1	0.48	-1.7	0.02	-2.0
A_30_P01033600			0.00	-3.5	0.38	-2.8	0.31	-2.1

3.2.4 BkF Liver. Significant probe list. List of all significantly differentially expressed probes in at least 1 treatment group (FDR P ≤ 0.05, fold change ± 1.5) in response to sub-chronic oral exposure to 25, 50, and 100 mg/kg-bw/day benzo(k)fluoranthene in the liver. The list is sorted from highest to lowest fold change in the 100 mg/kg-bw/day treatment group.

			**25 mg/kg-bw/day**		**50 mg/kg-bw/day**		**100 mg/kg-bw/day**	
Agilent Probe	Accession Number	Gene Symbol	*FDR P value*	*Fold change*	*FDR P value*	*Fold change*	*FDR P value*	*Fold change*
A_51_P269404	NM_008030	Fmo3	1.00	1.4	0.10	10.6	0.00	46.0
A_51_P279693	NM_009992	Cyp1a1	1.00	5.0	0.00	38.8	0.00	43.7
A_66_P119518	NM_017379	Tuba8	1.00	2.8	0.00	6.7	0.00	9.9
A_55_P1998471	NM_009114	S100a9	1.00	1.6	0.05	3.6	0.00	8.0
A_51_P255456	NM_009994	Cyp1b1	1.00	1.8	0.13	3.8	0.02	7.3
A_55_P1988703	NM_146896	Olfr1203	1.00	1.0	1.00	1.0	0.00	6.2
A_51_P279437	NM_029662	Mfsd2a	1.00	1.7	0.25	2.7	0.03	5.8
A_51_P375146	NM_007643	Cd36	1.00	3.0	0.10	3.1	0.02	5.7
A_52_P366803	NM_177380	Cyp3a44	1.00	-1.3	1.00	-1.0	0.03	5.6
A_51_P229893	NM_021445	Cts6	1.00	1.0	0.99	1.0	0.04	5.2

A_51_P153063	NM_029310	Fabp12	1.00	1.5	0.17	2.6	0.03	4.8
A_51_P365516	NM_009258	Spink3	1.00	-1.2	0.95	-1.2	0.00	4.3
A_51_P209183	NM_019568	Cxcl14	0.69	2.3	0.00	3.2	0.00	4.0
A_52_P343306	NM_010378	H2-Aa	1.00	2.3	0.00	3.9	0.01	3.9
A_51_P164296	NM_021475	Adamdec1	0.15	2.7	0.00	5.0	0.00	3.8
A_51_P284608	NM_001042605	Cd74	0.95	2.2	0.00	3.6	0.00	3.8
A_55_P2185840	NM_010924	Nnmt	1.00	1.2	0.03	2.5	0.02	3.7
A_55_P2032714	NM_010210	Fhit	1.00	1.5	0.27	2.0	0.03	3.6
A_55_P1952517	NM_001111296	Sult2a1	1.00	1.1	0.99	-1.0	0.03	3.5
A_66_P114333	NM_205823	Tlr12	0.52	1.7	0.00	3.2	0.00	3.3
A_55_P1983754	NM_025557	Pcp4l1	0.95	2.3	0.00	2.9	0.03	2.9
A_55_P1985788	NM_198092	Usp2	1.00	1.0	0.01	2.7	0.01	2.9
A_51_P335569	NM_030687	Slco1a4	1.00	-1.2	0.83	1.2	0.01	2.9
A_52_P375312	NM_001005421	Amica1	1.00	1.4	0.00	2.2	0.00	2.9
A_55_P2085779	NM_145449	Ifi27l2b	1.00	-1.3	0.75	1.4	0.03	2.8
A_51_P254425	NM_009644	Ahrr	1.00	1.4	0.15	2.2	0.03	2.8
A_55_P2136847	NM_001077514	Slc1a2	1.00	1.5	0.01	2.6	0.01	2.8
A_55_P2035018	NM_020001	Clec4n	1.00	1.5	0.00	2.7	0.00	2.8
A_51_P461665	NM_008599	Cxcl9	1.00	1.1	0.17	2.0	0.04	2.6
A_55_P1974645	NM_009849	Entpd2	1.00	1.5	0.00	2.0	0.00	2.6
A_55_P2097219	NM_010259	Gbp1	1.00	1.4	0.00	2.3	0.00	2.6
A_51_P464918	NM_019453	Mefv	0.84	1.7	0.00	2.4	0.00	2.6
A_55_P2131238	NM_153392	Ttc39a	0.39	1.8	0.00	2.5	0.00	2.6
A_51_P278868	NM_010387	H2-DMb1	1.00	1.5	0.02	2.4	0.03	2.4
A_51_P164835	NM_008909	Ppl	1.00	1.0	0.21	1.7	0.02	2.4
A_55_P2044653	NM_009999	Cyp2b10	1.00	1.5	0.91	1.2	0.02	2.4
A_51_P455647	NM_009801	Car2	1.00	1.5	0.00	2.6	0.01	2.4
A_55_P2007964	NM_009987	Cx3cr1	0.69	2.1	0.00	3.3	0.00	2.4
A_51_P144531	NM_172776	D630002G06Rik	1.00	-1.1	0.92	1.1	0.00	2.4
A_51_P146753	NM_007781	Csf2rb2	1.00	1.6	0.00	2.0	0.00	2.4
A_55_P2018417	NM_001163645	Osbpl3	0.00	2.2	0.00	2.8	0.00	2.3
A_55_P1963508	NM_001004148	Slc13a5	1.00	1.3	0.09	1.9	0.04	2.3
A_52_P515036	NM_016865	Htatip2	1.00	1.5	0.35	1.5	0.03	2.3
A_55_P1953387	NM_010634	Fabp5	1.00	1.9	0.00	2.8	0.08	2.3
A_55_P2150976	XM_886827	Fabp5l2	0.95	2.1	0.01	2.9	0.06	2.3
A_51_P255682	NM_008594	Mfge8	1.00	1.4	0.46	1.5	0.04	2.2
A_51_P269792	NM_009014	Rad51l1	1.00	1.2	0.59	1.4	0.03	2.2
A_66_P111562	NM_007631	Ccnd1	1.00	1.1	0.87	1.2	0.00	2.2
A_55_P2064771	NM_010741	Ly6c1	0.00	1.7	0.00	2.1	0.00	2.1
A_51_P151732	NM_019645	Pkp1	1.00	1.2	0.36	1.5	0.03	2.1
A_55_P2127179	XM_142052	Gm379	1.00	1.6	0.07	1.8	0.03	2.1
A_55_P2146560	XM_918601	LOC641240	0.75	2.0	0.00	2.3	0.03	2.1
A_55_P2140107	NM_009995	Cyp21a1	1.00	1.6	0.46	1.4	0.02	2.1
A_51_P105124	NM_183168	P2ry6	1.00	1.3	0.00	2.0	0.02	2.1
A_51_P124535	NM_008590	Mest	1.00	1.5	0.10	1.5	0.00	2.1
A_51_P265495	NM_010738	Ly6a	1.00	1.8	0.00	2.3	0.07	2.1
A_55_P1953169	NM_011315	Saa3	1.00	1.4	0.01	3.4	0.25	2.1
A_52_P537545	NM_021491	Smpd3	1.00	1.4	0.24	1.5	0.03	2.0
A_51_P304109	NM_010003	Cyp2c39	1.00	1.0	0.79	1.2	0.01	2.0

A_51_P172054	NM_019521	Gas6	1.00	1.7	0.00	2.0	0.01	2.0
A_51_P377452	NM_008677	Ncf4	1.00	1.3	0.03	1.9	0.01	2.0
A_55_P1983773	NM_001012273	Birc5	1.00	-1.3	0.41	1.3	0.00	2.0
A_51_P196844	NM_027881	Osbpl3	0.45	1.8	0.00	2.6	0.00	2.0
A_55_P2054420	NM_013685	Tcf4	0.00	1.8	0.00	2.0	0.00	1.9
A_51_P427516	NM_019576	Thsd1	1.00	1.3	0.17	1.7	0.04	1.9
A_51_P151126	NM_013706	Cd52	1.00	1.4	0.01	1.9	0.04	1.9
A_55_P1967168	NM_172668	Lrp4	1.00	1.4	0.04	1.7	0.04	1.9
A_51_P515605	NM_009930	Col3a1	1.00	1.2	0.00	2.1	0.04	1.9
A_55_P2142326	XM_923315		1.00	-1.0	0.71	1.3	0.03	1.9
A_55_P2004099	XM_001477458	Gm9933	1.00	1.2	0.00	1.8	0.03	1.9
A_51_P421140	NM_026473	Tubb6	1.00	1.5	0.14	1.6	0.03	1.9
A_51_P283004	NM_012033	Tinag	1.00	1.1	0.29	1.4	0.02	1.9
A_55_P2110512	NM_201529	Lmo7	1.00	1.2	0.14	1.5	0.01	1.9
A_55_P2033041	XM_001471956	LOC100038947	1.00	1.1	0.00	1.7	0.00	1.9
A_51_P291501	NM_001081062	Ccno	1.00	1.3	0.02	1.7	0.00	1.9
A_55_P1972720	NM_013872	Pmm1	1.00	1.3	0.01	1.7	0.00	1.9
A_55_P2156731	NM_010382	H2-Eb1	0.79	1.9	0.00	1.9	0.00	1.9
A_55_P2001250	NM_153107	Cpz	1.00	1.3	0.04	1.9	0.08	1.9
A_55_P2004732	NM_153392	Ttc39a	1.00	1.4	0.02	1.9	0.07	1.9
A_55_P2005470	NM_029568	Mfap4	1.00	1.3	0.00	3.0	0.15	1.9
A_52_P222230	XM_001476722		0.95	1.6	0.14	1.5	0.03	1.8
A_52_P228236	NM_011638	Tfrc	1.00	1.2	0.66	1.2	0.03	1.8
A_55_P1996946	NM_023223	Cdc20	1.00	-1.2	0.77	1.2	0.03	1.8
A_55_P2110513	NM_201529	Lmo7	1.00	1.2	0.22	1.4	0.03	1.8
A_51_P454196	NM_028182	Sh2d4a	1.00	1.1	0.23	1.4	0.02	1.8
A_51_P114634	NM_173405	Amz1	0.88	1.5	0.00	2.1	0.01	1.8
A_55_P1962747	NM_207105	H2-Ab1	0.65	1.8	0.03	1.9	0.07	1.8
A_55_P2161170	NM_146032	Srp68	0.95	1.9	0.02	2.2	0.10	1.8
A_55_P2122841			1.00	1.7	0.00	2.2	0.10	1.8
A_51_P411271	NM_010902	Nfe2l2	1.00	1.0	0.09	1.4	0.01	1.7
A_55_P2109505	XM_890094	Gm6594	1.00	1.2	0.02	1.4	0.00	1.7
A_55_P2119985	NM_130862	Baiap2	1.00	1.2	0.13	1.4	0.00	1.7
A_51_P463765	NM_011595	Timp3	1.00	1.4	0.00	1.5	0.00	1.7
A_51_P246653	NM_020008	Clec7a	1.00	1.1	0.00	1.7	0.06	1.7
A_51_P247694	NM_173036	Gpr97	1.00	1.7	0.04	1.8	0.11	1.7
A_52_P21486	NM_183257	Hamp2	1.00	1.5	0.03	1.8	0.12	1.7
A_55_P2124736	NM_181277	Col14a1	1.00	1.2	0.00	2.0	0.14	1.7
A_51_P359603	NM_013566	Itgb7	1.00	1.4	0.01	2.1	0.16	1.7
A_55_P2023637	NM_021400	Prg4	1.00	-1.0	0.00	2.1	0.14	1.7
A_55_P1957413	NM_019391	Lsp1	1.00	1.4	0.00	2.2	0.16	1.7
A_52_P642488	NM_008430	Kcnk1	0.85	2.0	0.00	2.9	0.23	1.7
A_55_P1960556	NM_015729	Acox1	0.00	2.2	0.00	2.2	0.07	1.6
A_51_P261517	NM_011662	Tyrobp	1.00	1.0	0.11	1.5	0.04	1.6
A_51_P436727	NM_177231	Arrb1	1.00	1.1	0.46	1.2	0.04	1.6
A_51_P498631	NM_018769	Dfna5	1.00	1.4	0.27	1.4	0.04	1.6
A_55_P2116111	NM_172911	D8Ertd82e	1.00	1.3	0.04	1.4	0.03	1.6
A_55_P1965154	NM_025565	Spc25	1.00	-1.0	0.61	1.2	0.03	1.6
A_51_P187750	NM_153408	Neurl3	0.98	1.4	0.00	1.6	0.03	1.6

A_55_P2081530	NM_080559	Sh3bgrl3	1.00	1.0	0.02	1.5	0.03	1.6
A_55_P2148062	NM_175391	Apol7c	1.00	1.2	0.00	1.7	0.03	1.6
A_55_P2129348	NM_153510	Pilra	1.00	1.1	0.00	1.5	0.02	1.6
A_55_P2048348	NM_025446	Aig1	1.00	-1.1	0.73	1.1	0.01	1.6
A_66_P125722	NM_025797	Cyb5	1.00	1.3	0.01	1.5	0.01	1.6
A_55_P2004452	NM_025703	Tceal8	1.00	1.3	0.00	1.5	0.01	1.6
A_51_P184484	NM_008607	Mmp13	1.00	1.3	0.02	1.5	0.00	1.6
A_55_P1955412	NM_011393	Slc1a2	1.00	1.2	0.00	1.5	0.00	1.6
A_55_P2157023	NM_011441	Sox17	1.00	1.0	0.00	1.6	0.00	1.6
A_51_P146970	NM_145831	Dmrt2	1.00	1.4	0.00	1.8	0.14	1.6
A_51_P121031	NM_175188	41334.0	1.00	1.0	0.02	1.5	0.05	1.5
A_55_P2130885	NM_008552	Mas1	1.00	1.1	0.40	1.3	0.04	1.5
A_52_P417825	NM_145950	Osgin2	1.00	1.2	0.26	1.3	0.04	1.5
A_52_P392674	NM_019736	Acot9	1.00	1.0	0.68	1.2	0.04	1.5
A_55_P2042823	NM_001145859	Sh3bp2	1.00	1.2	0.01	1.5	0.04	1.5
A_55_P2080042	NR_003629	BB287469	1.00	1.2	0.94	1.1	0.04	1.5
A_52_P465129	XM_001473647	LOC100045212	1.00	-1.0	0.01	1.5	0.04	1.5
A_55_P2066559	NM_001168693	Pp11r	0.23	1.5	0.00	1.6	0.03	1.5
A_51_P224575	NM_010322	Gnpat	1.00	1.3	0.65	1.2	0.03	1.5
A_51_P215438	NM_011172	Prodh	0.94	1.4	0.01	1.5	0.03	1.5
A_55_P2028496	XM_001472085	Hmgn2l6	1.00	1.2	0.14	1.3	0.03	1.5
A_51_P320022	NM_009728	Atp10a	1.00	1.2	0.19	1.3	0.03	1.5
A_55_P2074631	NM_007755	Cpeb1	1.00	1.1	0.02	1.5	0.02	1.5
A_30_P01029371			1.00	-1.1	0.87	1.1	0.02	1.5
A_55_P2128821	NM_019979	Selk	1.00	1.3	0.11	1.4	0.02	1.5
A_55_P2011380	NM_009612	Acvrl1	1.00	1.2	0.00	1.6	0.01	1.5
A_51_P400543	NM_019467	Aif1	1.00	1.1	0.06	1.4	0.00	1.5
A_51_P355753	NM_010430	Hic1	0.78	1.3	0.00	1.5	0.00	1.5
A_55_P1987261	NM_172691	B230312A22Rik	1.00	1.1	0.00	1.4	0.00	1.5
A_55_P1968193	NM_010210	Fhit	1.00	1.4	0.00	1.4	0.00	1.5
A_52_P393314	NM_011027	P2rx7	1.00	1.1	0.03	1.5	0.12	1.5
A_55_P1983044	NM_001081687	Gm5150	1.00	1.0	0.03	1.6	0.12	1.5
A_52_P318361	NM_145603	Ces2	1.00	1.6	0.00	1.6	0.12	1.5
A_55_P2109479	NM_001001180	BC066028	1.00	1.4	0.03	1.7	0.09	1.5
A_55_P2158946	NM_008481	Lama2	1.00	1.2	0.00	1.7	0.08	1.5
A_55_P2144526	NM_001080381	Fam65b	1.00	1.5	0.03	2.0	0.37	1.5
A_55_P1963960	XM_918548	LOC641201	1.00	1.1	0.03	1.5	0.25	1.4
A_52_P10041	NM_009658	Akr1b3	1.00	1.2	0.02	1.5	0.14	1.4
A_52_P52618	NM_007780	Csf2rb	1.00	1.2	0.02	1.5	0.14	1.4
A_55_P2129658	NM_008542	Smad6	1.00	1.2	0.01	1.5	0.03	1.4
A_51_P256246	NM_025359	Tspan13	1.00	1.3	0.02	1.7	0.33	1.4
A_51_P299805	NM_027872	Slc46a3	0.69	1.4	0.00	1.7	0.02	1.4
A_55_P2116978	NM_010889	Neb	1.00	1.3	0.03	1.8	0.52	1.4
A_55_P2029106	NM_138313	Bmf	1.00	1.4	0.04	1.9	0.51	1.4
A_51_P404193	NM_022435	Sp5	1.00	1.2	0.02	2.3	0.68	1.4
A_55_P2181419	NM_013685	Tcf4	0.85	1.4	0.03	1.5	0.25	1.3
A_55_P2130388	NM_138315	Mical1	1.00	1.2	0.03	1.5	0.29	1.3
A_55_P2034663	NM_007781	Csf2rb2	1.00	1.2	0.00	1.5	0.14	1.3
A_55_P2051322	NM_025994	Efhd2	1.00	1.0	0.00	1.6	0.40	1.3

A_55_P2137941	NM_052823	Fxyd2	1.00	1.2	0.03	1.5	0.57	1.2
A_52_P213696	NM_027402	Fndc5	1.00	1.1	0.04	1.6	0.78	1.1
A_55_P2069935	NM_052823	Fxyd2	1.00	1.2	0.02	1.7	0.71	1.1
A_55_P2099742	NM_011888	Ccl19	1.00	1.5	0.00	2.0	0.94	1.1
A_52_P650453	NM_011463	Spink4	1.00	1.0	0.04	3.4	0.95	1.1
A_55_P2086089	XM_111973	Smek3	0.00	1.5	0.56	1.2	0.89	1.0
A_66_P113892	NM_001162998	1110017F19Rik	1.00	1.2	0.00	1.6	0.95	-1.0
A_51_P156955	NM_013459	Cfd	1.00	1.2	0.00	7.1	1.00	-1.0
A_55_P1999838	NM_007749	Cox7c	1.00	-1.0	0.00	-1.5	0.67	-1.2
A_55_P2115220	NM_001037932	Gm11437	1.00	-1.2	0.00	-1.6	0.71	-1.3
A_55_P2003483	NM_138595	Gldc	1.00	-1.2	0.00	-1.6	0.32	-1.3
A_51_P434101	NM_022331	Herpud1	0.95	-1.5	0.00	-1.5	0.32	-1.3
A_66_P120472	NM_021319	Pglyrp2	0.69	-1.5	0.00	-1.8	0.10	-1.4
A_55_P2279035	AK018519	9030419F21Rik	1.00	-1.4	0.02	-1.7	0.16	-1.4
A_55_P2293013	NM_133960	Ces6	1.00	-1.2	0.00	-1.5	0.02	-1.4
A_30_P01027623			1.00	-1.3	0.00	-1.5	0.11	-1.4
A_55_P1972018	NM_178208	Hist1h4c	1.00	-1.2	0.08	-1.4	0.04	-1.5
A_66_P136632	NM_019769	1500003O03Rik	1.00	-1.3	0.27	-1.2	0.03	-1.5
A_30_P01021865			1.00	-1.0	0.42	-1.2	0.03	-1.5
A_55_P2105436	AK144920		1.00	-1.0	0.30	-1.2	0.03	-1.5
A_52_P188425	NM_001039077	Prosc	1.00	-1.1	0.13	-1.3	0.03	-1.5
A_30_P01030882			1.00	-1.1	0.24	-1.3	0.03	-1.5
A_55_P2129826	NM_178641	Inpp5f	1.00	-1.0	0.51	-1.2	0.03	-1.5
A_55_P2005859	NM_022014	Fn3k	1.00	-1.1	0.74	-1.1	0.02	-1.5
A_30_P01020465			0.69	-1.4	0.35	-1.2	0.02	-1.5
A_51_P462385	NM_008061	G6pc	1.00	1.1	0.24	-1.3	0.02	-1.5
A_55_P1966537	XM_897326	Gm6358	1.00	-1.1	0.00	-1.4	0.02	-1.5
A_30_P01030745			1.00	-1.0	0.23	-1.2	0.02	-1.5
A_30_P01032777			1.00	-1.1	0.14	-1.3	0.02	-1.5
A_55_P2170514	NM_018754	Sfn	1.00	-1.1	0.62	-1.2	0.01	-1.5
A_55_P2377527	NM_178930	Gbf1	1.00	-1.1	0.30	-1.2	0.00	-1.5
A_55_P2141068	NM_001033259	Ccdc109a	1.00	-1.1	0.00	-1.3	0.00	-1.5
A_30_P01019049			1.00	-1.1	0.00	-1.5	0.00	-1.5
A_30_P01018959			1.00	-1.0	0.00	-1.6	0.00	-1.5
A_51_P145785	NM_007443	Ambp	1.00	-1.1	0.02	-1.4	0.00	-1.5
A_52_P636427	NM_021319	Pglyrp2	1.00	-1.5	0.00	-2.1	0.26	-1.5
A_55_P2053459	NM_001161355	Timd2	1.00	-1.2	0.00	-1.9	0.43	-1.5
A_30_P01021387			1.00	1.1	0.02	-1.7	0.16	-1.5
A_55_P1957198	NM_009693	Apob	1.00	-1.2	0.03	-1.5	0.16	-1.5
A_51_P494825	NM_001081279	Mfhas1	1.00	-1.2	0.76	-1.2	0.04	-1.6
A_55_P2459006	NM_177351	Agphd1	1.00	-1.2	0.26	-1.3	0.04	-1.6
A_55_P2153517	NM_027147	Enho	1.00	-1.4	0.06	-1.5	0.04	-1.6
A_55_P2087647	NM_080289	Grhpr	1.00	-1.1	0.23	-1.4	0.03	-1.6
A_30_P01022310			1.00	1.1	0.34	-1.3	0.03	-1.6
A_30_P01018142			1.00	-1.1	0.03	-1.4	0.01	-1.6
A_55_P2061520			1.00	-1.1	0.23	-1.3	0.01	-1.6
A_30_P01026609			1.00	-1.2	0.48	-1.2	0.00	-1.6
A_30_P01032527			1.00	-1.2	0.51	-1.2	0.00	-1.6
A_51_P368591	NM_053254	Tle6	1.00	-1.2	0.00	-1.4	0.00	-1.6

A_55_P2061645	AK140300	ND6	1.00	-1.1	0.00	-1.6	0.00	-1.6
A_30_P01021237			0.96	-1.4	0.14	-1.4	0.00	-1.6
A_55_P1968362	NM_011599	Tle1	1.00	-1.2	0.00	-1.5	0.00	-1.6
A_51_P153486	NM_018808	Dnajb1	0.61	-2.0	0.00	-2.2	0.20	-1.6
A_55_P2038358	NM_012006	Acot1	1.00	-1.6	0.01	-2.1	0.29	-1.6
A_30_P01029662			1.00	-1.5	0.00	-1.8	0.09	-1.6
A_55_P1985015	NM_033620	Pard3	1.00	-1.5	0.88	-1.1	0.04	-1.7
A_55_P2140118	NM_027455	Qpct	0.85	-1.5	0.17	-1.4	0.03	-1.7
A_51_P469951	NM_080448	Srgap3	1.00	-1.4	0.02	-1.7	0.03	-1.7
A_55_P1968355	NM_011599	Tle1	1.00	-1.2	0.03	-1.5	0.03	-1.7
A_55_P2107528			1.00	-1.2	0.02	-1.5	0.02	-1.7
A_30_P01028952			1.00	-1.2	0.15	-1.4	0.01	-1.7
A_55_P2125633	NM_001102438	Acbd5	1.00	-1.2	0.00	-1.5	0.00	-1.7
A_30_P01031082			1.00	-1.3	0.08	-1.3	0.00	-1.7
A_51_P319070	NM_026159	Retsat	1.00	-1.4	0.00	-1.8	0.00	-1.7
A_51_P175424	NM_011797	Car14	1.00	-1.3	0.01	-2.1	0.19	-1.7
A_55_P2096917	NM_001005423	Mreg	1.00	-1.2	0.49	-1.3	0.04	-1.8
A_51_P196862	NM_027908	Amdhd1	1.00	-1.2	0.16	-1.4	0.01	-1.8
A_55_P2177410	XM_001473808	Gm2814	0.23	-1.8	0.00	-1.6	0.00	-1.8
A_55_P1992582	NM_008256	Hmgcs2	1.00	-1.2	0.00	-1.7	0.00	-1.8
A_55_P2002757	NM_008528	Blnk	1.00	-1.0	0.87	-1.1	0.04	-1.9
A_51_P496432	NM_007981	Acsl1	1.00	-1.2	0.36	-1.4	0.03	-1.9
A_51_P463452	NM_007981	Acsl1	1.00	-1.4	0.16	-1.5	0.03	-1.9
A_55_P2068459	NM_010479	Hspa1a	0.69	-4.0	0.02	-3.2	0.46	-1.9
A_55_P1992720	XM_001476656	LOC100046701	1.00	-1.3	0.09	-1.7	0.04	-2.0
A_55_P2207186	DV055649		1.00	-1.3	0.92	-1.1	0.04	-2.0
A_30_P01031652			1.00	-1.3	0.46	-1.4	0.03	-2.0
A_55_P2141479	NR_004414	Rnu2	1.00	-1.3	0.23	-1.6	0.02	-2.1
A_55_P2076196	NM_001134644	CU041261.1	1.00	-1.1	0.56	-1.3	0.00	-2.1
A_30_P01030951			1.00	-1.5	0.00	-1.9	0.00	-2.2
A_55_P2031668	NM_013541	Gstp1	1.00	-1.5	0.15	-1.8	0.03	-2.3
A_52_P205255	NM_001110778	Adam11	0.69	-2.1	0.14	-1.8	0.03	-2.3
A_51_P344376	NM_013533	Gpr162	1.00	-1.6	0.01	-1.8	0.00	-2.3
A_55_P2043627	NM_001081120	Fam89a	1.00	-1.6	0.56	-1.5	0.04	-2.4
A_55_P2083307	NM_130890	Capn8	1.00	-1.5	0.30	-1.6	0.01	-2.4
A_55_P2151956	XM_001472653	Gm4480	0.59	-1.8	0.00	-2.2	0.00	-2.4
A_55_P1960735	NM_011819	Gdf15	1.00	-1.4	0.32	-1.6	0.04	-2.5
A_51_P266618	NM_010012	Cyp8b1	1.00	-1.3	0.46	-1.4	0.01	-2.5
A_55_P2056729	NM_008342	Igfbp2	1.00	-1.7	0.10	-2.2	0.03	-2.6
A_55_P2360266	BC052524	4833411C07Rik	1.00	-1.4	0.00	-2.0	0.00	-2.6
A_55_P1977776	NR_003280	LOC790956	1.00	-1.6	0.11	-2.0	0.02	-2.7
A_51_P268529	NM_144942	Csad	0.52	-2.5	0.06	-2.4	0.02	-2.7
A_55_P1954393	NM_144796	Susd4	1.00	-1.2	0.38	-1.6	0.00	-2.7
A_30_P01017959			1.00	1.0	0.69	-1.4	0.00	-2.7
A_55_P2000148	NM_175138	Dnaic1	0.59	-2.5	0.03	-2.4	0.01	-2.8
A_55_P2061104	NM_001081285	Mup6	1.00	-1.3	0.80	-1.3	0.00	-2.8
A_55_P2051476	NM_146148	C8a	1.00	-1.6	0.40	-1.6	0.00	-2.8
A_51_P204247	NM_146148	C8a	1.00	-1.6	0.15	-2.1	0.04	-3.0
A_55_P2351193	AF357397	Snora47	1.00	-1.5	0.21	-1.8	0.00	-3.0

A_55_P2081116	NM_001081120	Fam89a	1.00	-1.4	0.66	-1.5	0.01	-3.1
A_55_P2139087	NM_001013820	Gm5631	1.00	-1.3	0.63	-1.6	0.03	-3.3
A_55_P1994733	AK017734		1.00	-1.5	0.83	-1.3	0.00	-3.3
A_51_P257885	NM_175217	Mmd2	0.46	-2.5	0.00	-5.3	0.00	-3.9
A_55_P2075127	NM_011037	Pax2	1.00	-1.1	0.85	-1.4	0.03	-5.1
A_55_P2046671	XM_001477211	CU104690.1	1.00	-1.3	0.83	-1.4	0.00	-5.3
A_52_P402127	NM_001126319	Mup9	1.00	-1.1	0.93	-1.3	0.03	-6.7
A_51_P189733	NM_175250	2810007J24Rik	1.00	-1.1	0.92	-1.4	0.04	-6.8
A_51_P456208	NM_011575	Tff3	0.00	-5.3	0.00	-3.6	0.00	-6.9
A_55_P2143923	NM_022411	Slc13a2	0.15	-2.6	0.00	-4.5	0.00	-8.4
A_51_P493987	NM_021509	Moxd1	0.42	-5.0	0.00	-7.6	0.00	-23.9

3.2.5 Chr Liver. Significant probe list. List of all significantly differentially expressed probes in at least 1 treatment group (FDR P ≤ 0.05, fold change ± 1.5) in response to sub-chronic oral exposure to 17.5, 50, and 150 mg/kg-bw/day chrysene in the liver. The list is sorted from highest to lowest fold change in the 150 mg/kg-bw/day treatment group.

			**17.5 mg/kg-bw/day**		**50 mg/kg-bw/day**		**150 mg/kg-bw/day**	
Agilent Probe	Accession Number	Gene Symbol	*FDR P value*	*Fold change*	*FDR P value*	*Fold change*	*FDR P value*	*Fold change*
A_55_P2142226	NM_001034870	Serpina3h	0.00	2.9	0.96	1.2	0.07	3.3
A_55_P2032079	NM_016974	Dbp	0.97	1.5	0.00	5.6	0.00	3.1
A_55_P2032081	NM_016974	Dbp	0.98	1.5	0.00	5.8	0.07	3.1
A_66_P110251	NM_177102	Tmem91	0.12	1.7	0.72	1.4	0.00	2.0
A_51_P164296	NM_021475	Adamdec1	0.09	1.6	0.86	1.2	0.00	1.8
A_55_P1979893	NM_017376	Tef	1.00	1.2	0.00	2.2	0.42	1.6
A_30_P01024113			0.00	1.7	0.80	1.3	0.32	1.5
A_30_P01031799			0.00	1.7	0.90	1.1	0.12	1.5
A_30_P01031204			0.00	1.6	0.57	1.4	0.00	1.5
A_30_P01033354			0.00	1.5	0.40	1.4	0.15	1.4
A_55_P2339601	CO043442	AW060742	0.00	1.5	0.81	1.2	0.18	1.4
A_30_P01032773			0.00	1.5	0.72	1.2	0.25	1.3
A_55_P2095181	AK135025		0.00	1.5	0.87	1.2	0.44	1.3
A_55_P2185330	AK038241		0.00	1.5	0.84	1.2	0.30	1.3
A_55_P1967231	NM_175096	Stbd1	0.98	1.2	0.00	1.8	0.62	1.3
A_51_P271865	NM_016751	Clec4f	1.00	1.0	0.00	-1.8	0.99	1.0
A_55_P1985070	NM_019413	Robo1	0.00	-1.9	0.99	-1.0	0.97	-1.1
A_55_P1956223	NM_013490	Chka	1.00	-1.1	0.00	-1.6	0.44	-1.3
A_55_P2408588	NM_007489	Arntl	1.00	-1.1	0.00	-2.7	0.42	-1.7
A_51_P470715	NM_009895	Cish	0.00	-5.9	0.86	-1.8	0.84	-1.7
A_55_P2165091	NM_145368	Acnat2	0.74	-1.5	0.23	-2.2	0.00	-2.1

3.2.6 DBahA Liver. Significant probe list. List of all significantly differentially expressed probes in at least 1 treatment group (FDR P ≤ 0.05, fold change ± 1.5) in response to sub-chronic oral exposure to 6.25, 12.5, and 25 mg/kg-bw/day dibenz(ah)anthracene in the liver. The list is sorted from highest to lowest fold change in the 25 mg/kg-bw/day treatment group.

			**6.25 mg/kg-bw/day**		**12.5 mg/kg-bw/day**		**25 mg/kg-bw/day**	

Agilent Probe	Accession Number	Gene Symbol	FDR P value	Fold change	FDR P value	Fold change	FDR P value	Fold change
A_55_P2135203	XM_001477565	Gm9454	0.00	3.8	0.00	7.0	0.00	20.0
A_55_P1985788	NM_198092	Usp2	0.00	4.4	0.00	6.5	0.00	13.5
A_55_P2032081	NM_016974	Dbp	0.00	6.4	0.00	11.4	0.00	12.8
A_55_P2032079	NM_016974	Dbp	0.00	5.9	0.00	10.0	0.00	11.6
A_55_P2042813	NM_001039647	Gbp11	0.12	3.2	0.09	4.4	0.00	7.1
A_55_P2067777	NM_001162906	2410089E03Rik	0.15	2.0	0.00	4.9	0.00	6.2
A_51_P470715	NM_009895	Cish	0.61	2.2	0.36	3.5	0.02	6.1
A_30_P01018928	0	NA	0.75	1.4	0.30	2.0	0.00	3.9
A_55_P2024046	NM_001080934	Slc16a5	0.05	2.0	0.24	1.9	0.00	3.8
A_66_P120125	NM_053078	D0H4S114	0.71	1.5	0.42	2.1	0.01	3.8
A_55_P2051159	NM_029692	Upp2	0.11	2.3	0.22	2.2	0.00	3.8
A_55_P1979893	NM_017376	Tef	0.00	2.6	0.00	2.9	0.00	3.6
A_66_P136186	NM_009516	Wee1	0.20	1.9	0.16	2.1	0.00	3.5
A_52_P259817	NM_029692	Upp2	0.18	2.1	0.27	2.1	0.00	3.4
A_55_P2029574	NM_008898	Por	0.18	2.0	0.18	2.2	0.00	3.4
A_55_P1965456	NM_008262	Onecut1	0.98	1.0	0.70	1.6	0.00	3.4
A_51_P372456	NM_001081441	Wdr86	0.59	1.8	0.80	1.5	0.02	3.3
A_55_P2116165	NM_133232	Pfkfb3	0.58	1.6	0.10	2.7	0.00	3.3
A_52_P194851	NM_001162906	2410089E03Rik	0.68	1.4	0.08	2.3	0.00	3.1
A_51_P282760	NM_011066	Per2	0.24	1.9	0.79	1.3	0.00	3.1
A_52_P495869	NM_010658	Mafb	0.13	1.9	0.00	2.6	0.00	3.0
A_55_P2089488	NM_001039710	Coq10b	0.15	1.8	0.03	2.2	0.00	3.0
A_30_P01030435	0	NA	0.37	2.1	0.36	2.4	0.04	3.0
A_55_P1974487	NM_177195	Atp8b5	0.24	1.8	0.21	2.0	0.00	3.0
A_30_P01030934	0	NA	0.33	2.2	0.54	1.9	0.03	2.9
A_30_P01024344	0	NA	0.60	1.4	0.04	2.4	0.00	2.8
A_30_P01025511	0	NA	0.43	1.5	0.06	2.2	0.00	2.8
A_55_P2185900	NM_032002	Nrg4	0.34	1.9	0.64	1.6	0.01	2.7
A_55_P2019690	NM_001081349	Slc43a1	0.26	1.7	0.37	1.6	0.00	2.7
A_55_P1963134	XM_895691	Gm6135	0.22	2.0	0.71	1.5	0.00	2.7
A_52_P293682	XM_001477336	Sult2a7	0.45	1.6	0.45	1.7	0.01	2.6
A_51_P301998	NM_018881	Fmo2	0.32	1.5	0.72	1.3	0.00	2.6
A_52_P663526	NM_145497	BC016495	0.63	1.4	0.62	1.5	0.00	2.6
A_52_P547662	NM_008772	P2ry1	0.11	2.0	0.74	1.3	0.00	2.6
A_55_P2185905	NM_032002	Nrg4	0.40	1.9	0.90	1.2	0.04	2.5
A_51_P258493	NM_011067	Per3	0.09	2.0	0.32	1.7	0.01	2.5
A_52_P627816	NM_019984	Tgm1	0.79	1.3	0.78	1.3	0.01	2.5
A_52_P197402	NM_029057	Tbc1d30	0.77	1.3	0.76	1.3	0.01	2.5
A_55_P2081761	XM_001475152	LOC100045680	0.72	1.3	0.55	1.6	0.00	2.5
A_52_P366525	NM_001039710	Coq10b	0.70	1.3	0.25	1.9	0.00	2.5
A_55_P2167269	NM_008793	Pcsk4	0.34	1.5	0.16	1.8	0.00	2.5
A_55_P2142326	XM_923315	Zfp167	0.16	1.7	0.75	1.3	0.00	2.5
A_55_P1969660	NM_028730	Pex26	0.22	1.7	0.24	1.8	0.00	2.5
A_55_P1966029	AK084964	A930033H14Rik	0.49	1.4	0.25	1.7	0.00	2.4

A_55_P2404484	NR_027980	1700001L05Rik	0.74	1.3	0.17	1.8	0.00	2.4
A_55_P2119917	NM_011772	Ikzf4	0.33	1.7	0.61	1.5	0.00	2.4
A_51_P219483	NM_001024619	Tsku	0.73	1.3	0.83	1.2	0.00	2.4
A_52_P303891	NM_011584	Nr1d2	0.00	1.8	0.00	2.0	0.00	2.3
A_55_P2148534	BC096461	Nr1d2	0.00	2.1	0.00	2.1	0.00	2.3
A_55_P2114318	XM_001478955	Gm4080	0.04	2.2	0.24	1.9	0.00	2.3
A_55_P1983754	NM_025557	Pcp4l1	0.00	2.4	0.30	1.6	0.00	2.3
A_52_P211185	NM_008140	Gnat1	0.54	1.5	0.35	1.9	0.03	2.3
A_55_P2387665	AK033690	9130221J18Rik	0.21	2.0	0.93	1.1	0.03	2.3
A_51_P272553	NM_011498	Bhlhe40	0.84	1.2	0.39	1.6	0.01	2.3
A_30_P01029443	0	NA	0.79	1.2	0.59	1.5	0.01	2.3
A_51_P269404	NM_008030	Fmo3	0.59	1.4	0.81	1.2	0.01	2.3
A_55_P1979674	NM_011772	Ikzf4	0.61	1.5	0.51	1.6	0.01	2.3
A_51_P185292	NR_029475	4930581F22Rik	0.29	1.6	0.33	1.7	0.00	2.3
A_55_P2416494	AK020235	8430426J06Rik	0.64	1.4	0.36	1.7	0.00	2.3
A_55_P2082658	XM_001472995	Gm2268	0.15	1.7	0.69	1.3	0.00	2.3
A_55_P1970033	NM_011065	Per1	0.48	1.5	0.09	2.1	0.02	2.2
A_55_P2027102	NM_134257	Rgs3	0.72	1.3	0.28	1.9	0.02	2.2
A_51_P176042	NM_013631	Pklr	0.74	1.3	0.93	1.1	0.01	2.2
A_55_P2007495	XM_001472204	Gm2034	0.07	2.0	0.33	1.7	0.00	2.2
A_51_P243755	NM_011388	Slc10a2	0.13	2.0	0.63	1.5	0.03	2.1
A_51_P152990	NM_011825	Grem2	0.84	1.2	0.39	1.8	0.03	2.1
A_55_P2072925	0	NA	0.58	1.4	0.72	1.3	0.02	2.1
A_51_P473953	NM_001081295	4631416L12Rik	0.47	1.4	0.33	1.6	0.01	2.1
A_55_P2256586	AK016293	4930573O21Rik	0.06	1.7	0.14	1.6	0.00	2.1
A_51_P387235	NM_021524	Nampt	0.67	1.3	0.50	1.5	0.00	2.1
A_51_P158814	AK081212	Marveld1	0.29	1.5	0.29	1.6	0.00	2.1
A_55_P2047614	NM_172763	Zfp809	0.23	1.5	0.41	1.5	0.00	2.1
A_52_P376135	NM_021462	Mknk2	0.03	1.9	0.07	1.9	0.00	2.0
A_55_P2145804	NM_026531	Aen	0.53	1.2	0.00	1.7	0.00	2.0
A_55_P2303310	AK050309	C730036E19Rik	0.43	1.6	0.84	1.2	0.04	2.0
A_51_P288916	NM_177368	Tmtc2	0.43	1.5	0.60	1.5	0.03	2.0
A_51_P164296	NM_021475	Adamdec1	0.13	1.9	0.18	2.0	0.03	2.0
A_55_P2153783	NM_010231	Fmo1	0.12	1.8	0.85	-1.2	0.03	2.0
A_55_P2064851	NM_001164682	Mpp4	0.39	1.5	0.63	1.4	0.02	2.0
A_51_P200667	NM_053155	Clmn	0.40	1.4	0.40	1.5	0.01	2.0
A_55_P2243431	BC052902	Gdap10	0.09	1.9	0.28	1.7	0.01	2.0
A_55_P2006662	BC124559	Gm14431	0.20	1.6	0.56	1.4	0.01	2.0
A_55_P2394490	AK052609	D630004K10Rik	0.13	1.8	0.85	1.2	0.01	2.0
A_55_P2067533	NM_010446	Foxa2	0.16	1.6	0.83	1.2	0.01	2.0
A_55_P1954643	XM_001474512	2310081J21Rik	0.03	1.9	0.90	-1.1	0.01	1.9
A_52_P468068	NM_001163098	Tchh	0.03	2.2	0.53	1.5	0.05	1.9
A_55_P2051596	XM_001479558	Gm4499	0.70	1.2	0.04	1.8	0.00	1.9
A_52_P612803	NM_009831	Ccng1	0.06	1.5	0.00	1.7	0.00	1.9
A_55_P2048478	NM_172907	Olfml1	0.81	1.2	0.64	1.4	0.04	1.9
A_55_P2088028	NM_144800	Mtss1	0.54	1.3	0.46	1.5	0.02	1.9
A_55_P1956624	XM_001004267	Gm14431	0.60	1.3	0.84	1.2	0.02	1.9
A_55_P1998811	XM_001476688	Gm3430	0.72	1.2	0.09	1.7	0.01	1.9
A_55_P2141860	NM_026531	Aen	0.53	1.2	0.06	1.6	0.00	1.9

A_55_P2132512	NM_001111314	Ngef	0.12	1.7	0.47	1.4	0.00	1.9
A_52_P508991	NM_010231	Fmo1	0.57	1.3	0.91	1.1	0.00	1.9
A_55_P2022082	NM_021366	Klf13	0.22	1.4	0.12	1.6	0.00	1.9
A_55_P2091473	NM_009760	Bnip3	0.17	1.5	0.17	1.5	0.00	1.9
A_51_P352303	NM_011983	Homer2	0.35	1.5	0.85	1.2	0.00	1.9
A_55_P2016540	NM_009963	Cry2	0.00	1.6	0.09	1.5	0.00	1.8
A_55_P2035424	NM_008278	Hpgd	0.00	1.9	0.84	1.2	0.00	1.8
A_55_P2159934	NM_025786	Rnf186	0.00	3.5	0.08	2.6	0.27	1.8
A_55_P2000182	NM_177870	Slc5a6	0.36	1.3	0.04	1.6	0.00	1.8
A_55_P2083894	XM_001478221	Gm3697	0.88	1.1	0.87	1.2	0.04	1.8
A_51_P483473	NM_011375	St3gal5	0.57	1.4	0.40	1.5	0.04	1.8
A_30_P01018879	0	NA	0.91	1.1	0.61	1.4	0.04	1.8
A_55_P2390776	NR_027981	1810064F22Rik	0.14	1.8	0.35	1.6	0.04	1.8
A_51_P309854	NM_080465	Kcnn2	0.19	1.7	0.95	1.1	0.04	1.8
A_51_P369252	NM_001080995	4632434I11Rik	0.83	1.2	0.24	1.7	0.04	1.8
A_51_P488730	NM_175480	Zfp612	0.50	1.4	0.90	1.1	0.03	1.8
A_66_P123155	NM_027442	Ddo	0.67	1.3	1.00	1.0	0.03	1.8
A_55_P2255449	AK033778	NA	0.12	1.6	0.53	1.4	0.03	1.8
A_55_P2170881	NM_175164	Arhgap26	0.70	1.2	0.94	1.1	0.03	1.8
A_55_P2090633	NM_011200	Ptp4a1	0.28	1.5	0.66	1.3	0.01	1.8
A_30_P01032002	0	NA	0.90	1.1	0.28	1.6	0.01	1.8
A_55_P2325698	AK008051	2010002M09Rik	0.99	1.0	0.91	1.1	0.01	1.8
A_51_P316951	NM_001167860	Wipf3	0.17	1.5	0.41	1.4	0.00	1.8
A_55_P2052535	NM_001085546	LOC664987	0.08	1.5	0.42	1.4	0.00	1.8
A_51_P494125	NM_007431	Alpl	0.10	1.6	0.98	-1.0	0.00	1.8
A_51_P493037	NM_018831	Dclre1a	0.33	1.4	0.72	1.2	0.00	1.8
A_52_P40504	AK162948	NA	0.20	1.4	0.58	1.3	0.00	1.8
A_55_P2201395	AK082845	2410017I17Rik	0.24	1.5	0.90	1.1	0.00	1.8
A_55_P2084910	NM_178723	Zfp385b	0.07	1.6	0.82	1.2	0.00	1.8
A_66_P122086	NM_001039720	9030619P08Rik	0.75	1.2	0.46	1.4	0.00	1.8
A_55_P1994032	NM_013842	Xbp1	0.74	1.2	0.33	1.5	0.00	1.8
A_51_P285916	NM_019688	Rapgef4	0.57	1.3	0.80	1.2	0.00	1.8
A_51_P152845	NM_145076	Trim24	0.39	1.3	0.60	1.3	0.00	1.8
A_55_P2091472	NM_009760	Bnip3	0.13	1.5	0.11	1.5	0.00	1.8
A_51_P101460	NM_023842	Dsp	0.04	1.5	0.67	1.2	0.00	1.7
A_66_P118430	AK040717	NA	0.00	1.8	0.77	1.2	0.02	1.7
A_55_P2027392	NM_030258	Gpr146	0.61	1.2	0.03	1.5	0.00	1.7
A_55_P1979377	NM_011391	Slc16a7	0.42	1.4	0.85	1.2	0.04	1.7
A_52_P669005	NM_023624	Lrat	0.86	1.1	0.77	-1.2	0.04	1.7
A_55_P2031288	NM_031884	Abcg5	0.34	1.5	0.55	1.4	0.04	1.7
A_51_P458778	NM_008278	Hpgd	0.06	1.8	0.99	1.0	0.03	1.7
A_51_P314679	NM_001039521	Rrn3	0.75	1.2	0.46	1.5	0.03	1.7
A_51_P296608	NM_007836	Gadd45a	0.12	1.7	0.18	1.7	0.03	1.7
A_55_P1972297	NM_183152	Plk5	0.54	1.3	0.97	-1.0	0.03	1.7
A_52_P281659	AK002926	Klf13	0.31	1.4	0.61	1.3	0.02	1.7
A_51_P300572	NM_009579	Slc30a1	0.15	1.6	0.44	1.4	0.02	1.7
A_55_P2033997	NM_027442	Ddo	0.54	1.3	0.99	1.0	0.02	1.7
A_55_P2134236	NM_010446	Foxa2	0.35	1.4	0.80	1.2	0.02	1.7
A_30_P01018133	0	NA	0.78	1.2	0.81	1.2	0.02	1.7

A_55_P2007713	NM_053082	Tspan4	0.62	1.3	0.42	1.5	0.02	1.7
A_55_P2108248	NM_026639	Art4	0.68	1.3	0.84	1.2	0.01	1.7
A_55_P2052563	NM_010495	Id1	0.94	1.1	0.27	1.5	0.01	1.7
A_55_P2174601	XM_001477810	Plekha7	0.53	1.3	1.00	1.0	0.01	1.7
A_30_P01024578	0	NA	0.78	1.2	0.55	1.3	0.01	1.7
A_66_P125701	XM_001481105	Gm4673	0.55	1.3	0.72	1.2	0.01	1.7
A_55_P2097964	XM_001481064	9030622O22Rik	0.44	1.3	0.80	1.2	0.01	1.7
A_52_P516034	NM_011200	Ptp4a1	0.36	1.4	0.56	1.3	0.01	1.7
A_52_P451834	NM_183262	Stk35	0.11	1.6	0.27	1.5	0.01	1.7
A_55_P2032388	NM_145076	Trim24	0.55	1.3	0.73	1.2	0.01	1.7
A_52_P168575	NM_001013811	Fam169b	0.12	1.6	0.81	1.2	0.01	1.7
A_55_P1997911	0	NA	0.33	1.4	0.61	1.3	0.01	1.7
A_55_P2244142	BC030682	Klf13	0.37	1.4	0.25	1.5	0.00	1.7
A_51_P335981	NM_026272	Narf	0.07	1.4	0.33	1.3	0.00	1.7
A_55_P1981836	NM_145536	BC020535	0.00	1.6	0.80	1.1	0.00	1.6
A_55_P1961241	XM_001476526	LOC100046627	0.00	1.7	0.89	1.1	0.02	1.6
A_51_P109258	NM_138686	Cys1	0.00	2.0	0.96	1.1	0.07	1.6
A_52_P232580	NM_145508	Dyrk3	0.45	1.3	0.00	1.7	0.01	1.6
A_55_P2013760	NM_001135151	Slc39a14	0.31	1.3	0.00	1.6	0.00	1.6
A_51_P201338	NM_144800	Mtss1	0.70	1.2	0.47	1.4	0.04	1.6
A_52_P336585	NM_028717	Als2	0.32	1.4	0.91	1.1	0.04	1.6
A_55_P2083806	NM_177082	Sp8	0.78	1.2	0.78	1.2	0.04	1.6
A_55_P2073024	XM_001471547	Gm4841	0.74	1.2	0.90	-1.1	0.04	1.6
A_65_P06147	NM_008010	Fgfr3	0.67	1.2	0.87	1.1	0.04	1.6
A_55_P2009752	NM_172563	Hlf	0.15	1.5	0.69	1.2	0.03	1.6
A_52_P262676	0	NA	0.31	1.4	0.60	1.3	0.03	1.6
A_66_P120736	NM_009760	Bnip3	0.33	1.4	0.30	1.4	0.03	1.6
A_51_P446583	NM_173364	Zfp445	0.99	-1.0	0.67	1.3	0.03	1.6
A_51_P352738	NM_033564	Mpv17l	0.41	1.3	0.82	1.1	0.03	1.6
A_55_P2012815	NM_007397	Acvr2b	0.49	1.3	0.31	1.5	0.03	1.6
A_30_P01022288	0	NA	0.60	1.3	0.97	1.0	0.03	1.6
A_55_P1979973	NM_028778	Nuak2	0.49	1.3	0.72	1.2	0.02	1.6
A_51_P369862	NM_178772	Nceh1	0.84	1.1	0.40	1.4	0.02	1.6
A_55_P2322709	AK014609	4633401B06Rik	0.33	1.4	0.74	1.2	0.02	1.6
A_51_P386503	NM_026185	Abhd15	0.15	1.5	0.88	1.1	0.02	1.6
A_51_P240986	NM_198604	Plekhg6	0.73	1.2	0.91	1.1	0.02	1.6
A_51_P465292	NM_080462	Hnmt	0.22	1.4	0.68	1.2	0.01	1.6
A_55_P1959305	NM_178446	Rbm47	0.44	1.3	0.52	1.3	0.01	1.6
A_51_P256093	NM_011943	Map2k6	0.56	1.3	0.67	1.2	0.01	1.6
A_55_P2082045	NM_173028	Vps13a	0.63	1.2	0.78	1.2	0.01	1.6
A_55_P2065621	NM_133255	Hook2	0.42	1.3	0.11	1.5	0.01	1.6
A_51_P155755	NM_183139	Pld6	0.33	1.3	0.79	1.2	0.01	1.6
A_52_P607128	NM_031195	Msr1	0.13	1.4	0.41	1.3	0.01	1.6
A_55_P2040295	NM_011200	Ptp4a1	0.27	1.4	0.70	1.2	0.01	1.6
A_55_P1996087	XM_902322	Gm14326	0.27	1.4	0.49	1.3	0.01	1.6
A_55_P2009861	XM_001472138	Gm2015	0.13	1.5	0.28	1.4	0.01	1.6
A_55_P2156068	XM_001478105	Gm3823	0.07	1.5	0.07	1.5	0.01	1.6
A_51_P511236	NM_001077495	Pik3r1	0.70	1.2	0.62	1.3	0.01	1.6
A_55_P2031999	NM_145448	9030617O03Rik	0.44	1.3	0.46	1.3	0.01	1.6

A_51_P164835	NM_008909	Ppl	0.17	1.4	0.68	1.2	0.00	1.6
A_55_P1971889	NM_010171	F3	0.57	1.2	0.19	1.5	0.00	1.6
A_51_P191893	AK163489	2410022M11Rik	0.12	1.3	0.28	1.3	0.00	1.6
A_55_P2130393	NM_001034885	Pnpla1	0.37	1.3	0.83	1.1	0.00	1.6
A_55_P1957088	NM_212473	Fam53b	0.65	1.2	0.27	1.4	0.00	1.6
A_30_P01020716	0	NA	0.23	1.3	0.28	1.4	0.00	1.6
A_55_P2002854	NM_001039194	Aifm2	0.22	1.3	0.41	1.3	0.00	1.6
A_55_P2064771	NM_010741	Ly6c1	0.53	1.2	0.13	1.5	0.00	1.6
A_55_P2104119	0	NA	0.83	1.1	0.60	1.2	0.00	1.6
A_55_P2097048	0	NA	0.31	1.3	0.33	1.4	0.00	1.6
A_51_P108020	NM_001029929	Zmynd15	0.04	1.5	0.47	1.3	0.03	1.5
A_52_P659258	NM_172146	Ppat	0.00	1.5	0.08	1.4	0.00	1.5
A_52_P78373	NM_011366	Sorbs3	0.00	1.5	0.03	1.4	0.00	1.5
A_55_P2102769	NM_153145	Abca8a	0.03	1.9	0.93	1.1	0.19	1.5
A_52_P608322	NM_010755	Maff	0.41	1.3	0.00	1.7	0.06	1.5
A_55_P2089238	NM_145553	Fam76a	0.16	1.3	0.00	1.5	0.00	1.5
A_55_P1990780	NM_011200	Ptp4a1	0.33	1.4	0.67	1.2	0.04	1.5
A_52_P538673	NM_010197	Fgf1	0.86	1.1	0.88	-1.1	0.04	1.5
A_55_P1972527	NM_178935	Txlng	0.47	1.3	0.80	1.1	0.04	1.5
A_55_P2007802	NM_026003	Smarca2	0.40	1.3	0.72	1.2	0.04	1.5
A_52_P609109	NM_145823	Pitpnc1	0.17	1.4	0.83	1.1	0.04	1.5
A_55_P1995774	0	NA	0.30	1.3	0.77	1.2	0.04	1.5
A_51_P212420	NM_010681	Lama4	0.18	1.4	0.57	1.3	0.04	1.5
A_55_P2160761	NM_001122676	Zcchc2	0.09	1.4	0.77	1.1	0.03	1.5
A_55_P2040860	NM_177576	Sun3	0.25	1.4	0.50	1.3	0.03	1.5
A_55_P2002578	NM_010145	Ephx1	0.58	1.2	0.53	1.3	0.03	1.5
A_55_P2360501	DV051837	NA	0.18	1.4	0.74	1.2	0.03	1.5
A_55_P2023484	NM_024289	Osbpl5	0.75	1.2	0.54	1.3	0.03	1.5
A_51_P478098	NM_001113416	Epb4.1l5	0.44	1.3	0.96	-1.0	0.03	1.5
A_51_P160907	NM_001045529	Morc3	0.20	1.4	0.78	1.1	0.02	1.5
A_52_P225584	NM_026185	Abhd15	0.20	1.4	1.00	1.0	0.02	1.5
A_30_P01032861	0	NA	0.14	1.4	0.79	1.1	0.02	1.5
A_55_P1969477	NM_011076	Abcb1a	0.35	1.3	0.71	1.2	0.02	1.5
A_55_P2081303	NM_001040400	Tet2	0.57	1.2	0.95	1.0	0.02	1.5
A_30_P01023346	0	NA	0.65	1.2	0.68	1.2	0.02	1.5
A_51_P490348	NM_025423	1110059E24Rik	0.95	1.0	0.64	1.2	0.02	1.5
A_30_P01021491	0	NA	0.42	1.3	0.79	1.1	0.01	1.5
A_55_P1954306	NM_007953	Esrra	0.59	1.2	0.81	1.1	0.01	1.5
A_51_P332141	NM_009900	Clcn2	0.30	1.3	0.38	1.3	0.01	1.5
A_55_P1966189	NM_001008700	Il4ra	0.80	1.1	0.63	1.2	0.01	1.5
A_55_P2024463	NM_198100	Tbkbp1	0.75	1.1	0.99	-1.0	0.01	1.5
A_51_P483483	AK014549	NA	0.16	1.3	0.45	1.2	0.00	1.5
A_51_P109449	NM_008519	Ltb4r1	0.16	1.4	0.63	1.2	0.00	1.5
A_55_P1956807	NM_001099308	Gm14391	0.04	1.4	0.42	1.3	0.00	1.5
A_52_P573552	NM_144549	Trib1	0.65	1.2	0.16	1.4	0.00	1.5
A_55_P2099742	NM_011888	Ccl19	0.49	1.2	0.88	1.1	0.00	1.5
A_30_P01028387	0	NA	0.49	1.2	0.58	1.2	0.00	1.5
A_55_P2019463	NM_001081260	Tnks1bp1	0.70	1.1	0.37	1.3	0.00	1.5
A_55_P2320085	AK014704	4833415N18Rik	0.93	1.0	0.55	1.2	0.00	1.5

A_55_P2055123	NM_001113478	Frrs1	0.00	1.4	0.47	1.2	0.00	1.5
A_55_P2367415	AK035805	A630026N12Rik	0.03	1.5	0.55	1.2	0.01	1.4
A_55_P2124385	NM_009647	Ak4	0.03	1.6	0.74	1.2	0.06	1.4
A_55_P2073248	NM_001013780	Slc25a34	0.03	1.7	0.95	1.1	0.18	1.4
A_30_P01027568	0	NA	0.00	1.5	0.85	1.1	0.08	1.3
A_55_P2340593	AK007545	1810019D21Rik	0.00	1.5	0.47	1.2	0.07	1.3
A_51_P509679	XM_001474025	Igh-VJ558	0.04	3.0	0.14	2.5	0.79	1.3
A_51_P117226	AK046533	NA	0.33	1.2	0.04	1.5	0.14	1.3
A_30_P01022593	0	NA	0.00	1.5	0.68	1.2	0.21	1.2
A_55_P2129000	NM_144853	Cyyr1	0.00	1.5	0.90	-1.1	0.79	1.1
A_55_P2272311	AK015661	4930500G05Rik	0.89	1.1	0.04	1.7	0.82	1.1
A_55_P2048493	NR_027955	4931440P22Rik	0.39	1.2	0.00	1.5	0.70	1.1
A_51_P436469	NM_144821	AI317395	0.91	1.1	0.04	-2.0	0.97	1.0
A_55_P1976744	XM_001478262	Gm4324	0.84	1.1	0.04	-1.7	0.83	-1.1
A_55_P2121225	NM_007818	Cyp3a11	0.97	1.0	0.00	-2.3	0.85	-1.1
A_52_P613653	NM_153525	Tmem41b	0.31	-1.3	0.04	-1.5	0.54	-1.2
A_30_P01019938	0	NA	0.67	1.2	0.00	-1.7	0.59	-1.2
A_30_P01022479	0	NA	0.92	-1.1	0.00	-1.8	0.64	-1.2
A_66_P106611	NM_008137	Gna14	0.79	-1.2	0.03	-2.1	0.74	-1.2
A_51_P137336	NM_009864	Cdh1	0.42	-1.4	0.00	-2.2	0.64	-1.2
A_30_P01033222	0	NA	0.00	-1.6	0.53	-1.3	0.15	-1.3
A_30_P01017460	0	NA	0.03	-1.5	0.57	-1.2	0.23	-1.3
A_30_P01026097	0	NA	0.00	-1.5	0.87	-1.1	0.18	-1.3
A_30_P01021625	0	NA	0.86	1.1	0.03	-1.7	0.33	-1.3
A_30_P01018251	0	NA	0.00	-2.1	0.56	-1.4	0.25	-1.4
A_52_P388164	NM_146756	Olfr608	0.00	-1.7	0.48	-1.3	0.12	-1.4
A_30_P01033615	0	NA	0.03	-1.6	0.21	-1.4	0.10	-1.4
A_55_P2107647	0	NA	0.04	-1.5	0.53	-1.2	0.04	-1.4
A_52_P550843	NM_024282	Pppde1	0.62	-1.2	0.04	-1.6	0.03	-1.4
A_52_P662098	NM_019671	Net1	0.65	-1.2	0.00	-1.6	0.03	-1.4
A_55_P2177248	AK137750	NA	0.55	-1.2	0.00	-1.6	0.07	-1.4
A_52_P269158	NM_001003948	Pid1	0.54	-1.3	0.03	-1.8	0.24	-1.4
A_66_P134704	NM_001013762	Gm4952	0.89	-1.1	0.03	-2.0	0.34	-1.4
A_55_P2179974	NM_029035	Spsb1	0.00	-2.0	0.51	-1.3	0.01	-1.5
A_30_P01020059	0	NA	0.04	-1.9	0.42	-1.5	0.25	-1.5
A_55_P2182392	NM_001100606	Adat3	0.00	-1.8	0.45	-1.3	0.03	-1.5
A_55_P2249897	AK081173	B930096F20Rik	0.00	-1.6	0.27	-1.3	0.00	-1.5
A_55_P2008874	NM_001003961	Dnmt3b	0.00	-1.6	0.03	-1.7	0.01	-1.5
A_55_P2391185	DV044254	NA	0.05	-1.5	0.42	-1.3	0.02	-1.5
A_55_P2065671	NM_172301	Ccnb1	0.00	-1.5	0.99	-1.0	0.00	-1.5
A_66_P135403	AK135748	AI481877	0.00	-1.5	0.39	-1.3	0.01	-1.5
A_55_P2119764	NM_010672	Krtap6-1	0.67	-1.2	0.04	-1.8	0.16	-1.5
A_30_P01021637	0	NA	0.35	-1.3	0.04	-1.8	0.05	-1.5
A_66_P105736	NM_019671	Net1	0.83	-1.1	0.00	-1.9	0.01	-1.5
A_55_P2048499	XM_001474426	NA	0.64	-1.3	0.04	-2.0	0.17	-1.5
A_30_P01018914	0	NA	0.43	-1.5	0.04	-2.2	0.34	-1.5
A_52_P175242	NM_010570	Irs1	0.83	1.1	0.24	-1.4	0.04	-1.5
A_51_P111164	NM_172612	Rnd1	0.97	-1.0	0.92	-1.1	0.04	-1.5
A_55_P2020188	XM_993808	LOC676890	0.21	-1.4	0.12	-1.5	0.04	-1.5

A_55_P2106394	NM_026412	D2Ertd750e	0.41	-1.3	0.93	-1.1	0.03	-1.5
A_55_P2070194	NM_016739	Caprin1	0.64	-1.2	0.37	-1.4	0.03	-1.5
A_51_P227004	NM_016904	Cks1b	0.27	-1.4	0.52	-1.3	0.03	-1.5
A_55_P1999648	NM_007408	Plin2	0.52	-1.3	0.46	-1.3	0.03	-1.5
A_51_P344376	NM_013533	Gpr162	0.22	-1.4	0.40	-1.3	0.03	-1.5
A_30_P01019283	0	NA	0.16	-1.5	0.65	-1.2	0.02	-1.5
A_51_P104891	NM_027652	Ept1	0.71	-1.2	0.28	-1.4	0.02	-1.5
A_51_P114028	NM_009746	Bcl7c	0.18	-1.4	0.29	-1.4	0.02	-1.5
A_52_P568805	NM_146093	Ubxn1	0.53	-1.3	0.73	-1.2	0.02	-1.5
A_51_P479052	NM_033074	Tars	0.14	-1.4	0.08	-1.6	0.01	-1.5
A_55_P2087280	XM_001003060	LOC677388	0.76	-1.1	0.45	-1.3	0.01	-1.5
A_52_P226929	NM_207653	Cflar	0.32	-1.3	0.21	-1.3	0.01	-1.5
A_55_P2013043	NM_011454	Serpinb6b	0.75	-1.2	0.38	-1.3	0.01	-1.5
A_52_P504478	NM_007414	Adprh	0.12	-1.3	0.33	-1.2	0.00	-1.5
A_55_P2178803	NM_001163284	Zbtb5	0.20	-1.3	0.25	-1.3	0.00	-1.5
A_55_P2044143	NM_001164311	Loxl4	0.04	-1.3	0.19	-1.2	0.00	-1.5
A_66_P129763	XM_897153	Gm6329	0.00	-1.9	0.12	-1.6	0.05	-1.6
A_51_P392429	NM_175534	Mrgpre	0.00	-1.8	0.30	-1.4	0.01	-1.6
A_55_P1964613	AK168315	Dnttip1	0.00	-1.8	0.33	-1.4	0.01	-1.6
A_55_P2055168	NM_001122662	Gm2016	0.03	-1.6	0.06	-1.6	0.01	-1.6
A_55_P2160726	AK147898	Tcfcp2	0.00	-1.6	0.78	-1.1	0.00	-1.6
A_55_P2012779	NM_027445	Rnf167	0.00	-1.6	0.21	-1.3	0.00	-1.6
A_51_P488510	NM_145221	Appl1	0.03	-1.5	0.00	-1.4	0.01	-1.6
A_51_P379976	NM_001039515	Arl4a	0.32	-1.3	0.00	-1.6	0.00	-1.6
A_55_P2097665	NM_001039515	Arl4a	0.55	-1.2	0.00	-1.8	0.01	-1.6
A_55_P2027213	NM_001163615	Krtap20-2	0.47	-1.4	0.03	-2.0	0.05	-1.6
A_52_P883557	NM_001033286	Slc30a10	0.50	-1.5	0.04	-2.4	0.26	-1.6
A_51_P241995	NM_016919	Col5a3	0.76	-1.2	0.71	-1.2	0.04	-1.6
A_55_P1970910	NM_001163493	Stard13	0.94	-1.1	0.29	-1.4	0.04	-1.6
A_55_P1966863	NM_027985	Mad2l2	0.44	-1.3	0.69	-1.3	0.04	-1.6
A_52_P214630	NM_011448	Sox9	0.12	-1.5	0.24	-1.5	0.03	-1.6
A_55_P2368710	AK082316	C230037E05Rik	0.30	-1.4	0.35	-1.5	0.03	-1.6
A_51_P218953	NM_172385	Zfp536	0.12	-1.5	0.92	-1.1	0.03	-1.6
A_55_P1968178	NM_174992	Smagp	0.30	-1.4	0.31	-1.4	0.02	-1.6
A_55_P2279035	AK018519	9030419F21Rik	0.22	-1.4	0.29	-1.4	0.02	-1.6
A_55_P2143436	0	NA	0.10	-1.5	0.09	-1.5	0.02	-1.6
A_55_P1957038	NM_181796	Gstp2	0.62	-1.2	0.77	-1.2	0.01	-1.6
A_55_P1956718	NM_028238	Rab38	0.93	-1.1	0.37	-1.4	0.01	-1.6
A_51_P374464	NM_013541	Gstp1	0.50	-1.3	0.88	-1.1	0.01	-1.6
A_55_P2032818	NM_030706	Trim2	0.43	-1.3	0.11	-1.6	0.01	-1.6
A_52_P334670	NM_007715	Clock	0.71	-1.2	0.14	-1.5	0.01	-1.6
A_52_P91618	NM_030257	Lysmd3	0.15	-1.4	0.21	-1.5	0.01	-1.6
A_51_P264825	NM_008479	Lag3	0.34	-1.3	0.21	-1.5	0.01	-1.6
A_55_P1980836	XM_001472686	NA	0.32	-1.3	0.90	-1.1	0.00	-1.6
A_52_P547187	NM_138667	Tab2	0.51	-1.3	0.39	-1.4	0.00	-1.6
A_66_P107231	NM_001164311	Loxl4	0.07	-1.4	0.06	-1.4	0.00	-1.6
A_30_P01027122	0	NA	0.47	-1.3	0.83	-1.1	0.00	-1.6
A_52_P625683	NM_021534	Pxmp4	0.16	-1.4	0.11	-1.4	0.00	-1.6
A_55_P2247096	BC028777	BC028777	0.13	-1.5	0.85	-1.1	0.00	-1.6

A_55_P2221236	AK085976	NA	0.00	-2.3	0.79	-1.2	0.03	-1.7
A_55_P2076749	NM_023525	Cad	0.05	-1.8	0.03	-2.0	0.01	-1.7
A_55_P2173288	NM_053126	Pcdhb1	0.03	-1.7	0.42	-1.4	0.01	-1.7
A_55_P2099947	NM_001163282	Gdf1	0.03	-1.7	0.00	-1.9	0.01	-1.7
A_51_P349495	NM_153546	Mboat1	0.05	-1.6	0.38	-1.3	0.00	-1.7
A_51_P418560	NM_080795	Lnx2	0.52	-1.3	0.03	-1.6	0.00	-1.7
A_55_P2146655	XM_001472967	Gm2262	0.13	-1.7	0.00	-2.1	0.06	-1.7
A_55_P2069755	NM_138302	Tymp	0.52	-1.3	0.47	-1.4	0.04	-1.7
A_51_P352594	NM_001001326	St5	0.35	-1.4	0.17	-1.7	0.03	-1.7
A_55_P1972490	XM_001474107	Gm4055	0.62	-1.3	0.42	-1.4	0.03	-1.7
A_55_P1987290	NR_004413	Rnu1b6	0.45	-1.3	0.35	-1.5	0.03	-1.7
A_55_P2008704	NM_181796	Gstp2	0.63	-1.2	0.86	-1.1	0.01	-1.7
A_51_P487298	NM_139307	Vasn	0.21	-1.5	0.32	-1.5	0.01	-1.7
A_55_P2173457	XM_001479022	1700097N02Rik	0.54	-1.3	0.42	-1.5	0.01	-1.7
A_51_P117618	NM_023154	Ethe1	0.17	-1.5	0.30	-1.5	0.01	-1.7
A_55_P2115955	NM_198193	Raet1e	0.83	-1.1	0.49	-1.4	0.01	-1.7
A_55_P2106241	NM_029045	4930432K21Rik	0.27	-1.4	0.79	-1.2	0.01	-1.7
A_52_P376360	NM_019971	Pdgfc	0.15	-1.4	0.22	-1.5	0.00	-1.7
A_51_P456208	NM_011575	Tff3	0.03	-2.2	0.24	-1.7	0.05	-1.8
A_30_P01021387	0	NA	0.10	-1.6	0.00	-2.4	0.01	-1.8
A_30_P01032489	0	NA	0.13	-1.7	0.06	-1.9	0.04	-1.8
A_51_P469951	NM_080448	Srgap3	0.80	-1.2	0.62	-1.4	0.03	-1.8
A_51_P228295	NM_001001880	Mpzl1	0.42	-1.4	0.39	-1.5	0.02	-1.8
A_55_P1999958	XM_001475982	Gm3185	0.27	-1.5	0.28	-1.5	0.01	-1.8
A_51_P275496	XM_001480912	BC026762	0.24	-1.5	0.27	-1.5	0.01	-1.8
A_66_P124052	NM_016778	Bok	0.56	-1.3	0.61	-1.4	0.01	-1.8
A_51_P415519	NM_026407	Tmem39a	0.27	-1.5	0.27	-1.5	0.01	-1.8
A_55_P2000148	NM_175138	Dnaic1	0.16	-1.5	0.32	-1.5	0.00	-1.8
A_51_P342871	NM_009112	S100a10	0.21	-1.5	0.28	-1.5	0.00	-1.8
A_55_P2031668	NM_013541	Gstp1	0.78	-1.2	0.84	-1.2	0.00	-1.8
A_66_P124715	NM_011145	Ppard	0.00	-1.8	0.00	-1.7	0.00	-1.9
A_30_P01017875	0	NA	0.39	-1.5	0.43	-1.6	0.03	-1.9
A_55_P2083307	NM_130890	Capn8	0.09	-1.9	0.78	-1.3	0.03	-1.9
A_52_P533146	NM_007837	Ddit3	0.24	-1.5	0.21	-1.6	0.01	-1.9
A_55_P2024530	NM_009174	Siah2	0.27	-1.6	0.22	-1.8	0.01	-1.9
A_55_P2182273	XM_001000082	NA	0.06	-1.7	0.22	-1.6	0.00	-1.9
A_55_P2043122	NM_028710	Arsg	0.16	-1.5	0.36	-1.4	0.00	-1.9
A_55_P2048259	NM_146133	Golph3l	0.38	-1.4	0.40	-1.5	0.00	-1.9
A_55_P1954436	XM_991070	Gm7967	0.15	-1.5	0.09	-1.5	0.00	-1.9
A_51_P489153	NM_023733	Crot	0.44	-1.4	0.43	-1.4	0.00	-1.9
A_55_P2055112	AB047758	Prss44	0.03	-2.2	0.65	-1.4	0.02	-2.0
A_55_P2102936	NM_001033225	Pnrc1	0.04	-1.6	0.03	-1.7	0.00	-2.0
A_55_P2332194	AK140090	Gm15998	0.76	-1.3	0.54	-1.5	0.04	-2.0
A_55_P1959748	NM_012055	Asns	0.89	-1.2	0.98	-1.0	0.04	-2.0
A_51_P341918	NM_009366	Tsc22d1	0.70	-1.3	0.20	-1.7	0.01	-2.0
A_51_P144349	NM_172442	Dtx4	0.48	-1.4	0.24	-1.7	0.01	-2.0
A_55_P2105858	NM_030693	Atf5	0.41	-1.4	0.17	-1.7	0.00	-2.0
A_51_P257675	NM_030203	Tspyl4	0.55	-1.3	0.56	-1.3	0.00	-2.0
A_51_P405606	NM_008681	Ndrg1	0.56	-1.4	0.28	-1.7	0.00	-2.0

A_55_P2105416	NR_003624	Gm10319	0.11	-2.6	0.04	-3.2	0.19	-2.1
A_51_P315042	NM_016847	Avpr1a	0.36	-1.5	0.00	-2.7	0.00	-2.2
A_52_P161495	NM_009744	Bcl6	0.71	1.4	0.69	-1.5	0.04	-2.2
A_55_P2035320	NM_017373	Nfil3	0.14	-1.6	0.00	-2.1	0.00	-2.3
A_51_P351860	NM_009777	C1qb	0.92	-1.2	0.97	-1.1	0.04	-2.3
A_55_P2062589	NM_008639	Mtnr1a	0.03	-2.3	0.00	-2.7	0.00	-2.4
A_55_P2143923	NM_022411	Slc13a2	0.55	-1.4	0.00	-2.9	0.00	-2.4
A_51_P257885	NM_175217	Mmd2	0.65	-1.5	0.30	-2.1	0.04	-2.4
A_51_P267354	NM_175478	Lrfn3	0.31	-1.7	0.06	-2.3	0.01	-2.4
A_55_P2157008	NM_053112	Ear10	0.37	-1.7	0.45	-1.8	0.00	-2.4
A_51_P386539	NM_026301	Rnf125	0.17	-1.6	0.11	-1.9	0.00	-2.4
A_51_P304683	NM_011802	Clpx	0.18	-1.7	0.09	-2.1	0.00	-2.5
A_55_P1957918	NM_001135192	Asap2	0.11	-2.0	0.04	-2.6	0.00	-2.6
A_55_P2077783	NR_003964	Tubb2a-ps2	0.11	-2.1	0.00	-2.8	0.01	-2.6
A_51_P259296	NM_008509	Lpl	0.61	-1.4	0.61	-1.5	0.01	-2.6
A_55_P1956223	NM_013490	Chka	0.08	-1.9	0.03	-2.3	0.00	-2.7
A_52_P413947	NM_010840	Mthfr	0.14	-1.8	0.00	-2.3	0.00	-2.7
A_55_P2080956	NM_013490	Chka	0.06	-2.0	0.04	-2.4	0.00	-2.7
A_51_P440047	NM_173752	1110067D22Rik	0.25	-1.7	0.00	-2.4	0.00	-2.7
A_51_P350453	NM_013743	Pdk4	0.53	-1.6	0.00	-2.8	0.00	-2.7
A_55_P1982499	NM_177350	Gldn	0.04	-3.1	0.09	-3.2	0.05	-2.8
A_52_P329367	NM_001025566	Chka	0.00	-2.1	0.00	-3.0	0.00	-2.8
A_30_P01018128	0	NA	0.53	-1.4	0.00	-2.3	0.00	-2.8
A_52_P30451	NM_016854	Ppp1r3c	0.26	-2.1	0.00	-4.0	0.02	-2.8
A_51_P455338	NM_053113	Ear11	0.21	-2.4	0.39	-2.2	0.03	-3.1
A_52_P381484	NM_133903	Spon2	0.03	-2.2	0.10	-2.1	0.00	-3.2
A_51_P439452	NM_133748	Insig2	0.14	-1.9	0.09	-2.1	0.00	-3.2
A_51_P142923	NM_013490	Chka	0.03	-2.2	0.00	-3.0	0.00	-3.3
A_52_P2710	NM_023493	Cml5	0.77	-1.4	0.03	-3.4	0.00	-3.4
A_51_P490023	NM_009450	Tubb2a	0.20	-2.3	0.00	-3.8	0.01	-3.5
A_55_P1957624	NM_178082	Insig2	0.14	-2.0	0.12	-2.2	0.00	-4.2
A_51_P447545	NM_008341	Igfbp1	0.22	-2.8	0.50	-2.3	0.00	-4.6
A_55_P2408588	NM_007489	Arntl	0.16	-2.4	0.00	-4.2	0.00	-5.1

3.2.7 IP Liver. Significant probe list. List of all significantly differentially expressed probes in at least 1 treatment group (FDR P ≤ 0.05, fold change ± 1.5) in response to sub-chronic oral exposure to 12.5, 25, and 50 mg/kg-bw/day indeno(123,cd)pyrene in the liver. The list is sorted from highest to lowest fold change in the 50 mg/kg-bw/day treatment group.

			12.5 mg/kg-bw/day		25 mg/kg-bw/day		50 mg/kg-bw/day	
Agilent Probe	Accession Number	Gene Symbol	FDR P value	Fold change	FDR P value	Fold change	FDR P value	Fold change
A_51_P141535	NM_016978	Oat	1.00	1.4	0.73	1.5	0.00	2.1
A_51_P360492	NM_008567	Mcm6	1.00	-1.2	0.95	1.3	0.00	2.0
A_55_P1987439	NM_133779	Pigt	1.00	1.0	0.99	1.0	0.04	2.0
A_51_P464918	NM_019453	Mefv	1.00	1.1	0.66	1.4	0.04	1.7
A_51_P215489	NM_153062	Slc37a1	1.00	1.3	0.95	1.2	0.04	1.7
A_55_P2027392	NM_030258	Gpr146	1.00	1.1	0.92	1.2	0.00	1.5

A_55_P1985623	NM_029600	Abcc3	1.00	1.2	0.00	1.5	0.11	1.4
A_55_P2139009			1.00	-1.2	0.00	-1.5	0.44	-1.2
A_55_P1974542	NM_198612	Gxylt2	1.00	-1.2	0.00	-1.5	0.59	-1.2
A_55_P2108143	XM_001480454	LOC100048588	1.00	-1.2	0.00	-1.6	0.18	-1.4
A_55_P2106394	NM_026412	D2Ertd750e	1.00	-1.3	0.00	-1.5	0.11	-1.5
A_30_P01027755			1.00	-1.3	0.95	-1.2	0.00	-1.5
A_55_P2048259	NM_146133	Golph3l	1.00	-1.2	0.92	-1.2	0.00	-1.5
A_30_P01032612			1.00	-1.3	0.73	-1.3	0.00	-1.5
A_55_P2127174	NM_170759	Zfp628	1.00	-1.2	0.51	-1.3	0.00	-1.5
A_30_P01030745			1.00	-1.2	0.96	-1.0	0.00	-1.5
A_51_P453475	NM_009201	Slc1a5	0.00	-1.3	0.05	-1.4	0.00	-1.5
A_55_P1988260	XM_001479382	LOC100048847	1.00	-1.2	0.45	-1.3	0.00	-1.5
A_55_P2078123	NM_013646	Rora	1.00	-1.1	0.95	-1.1	0.04	-1.5
A_55_P2158522	NM_183355	Pbx1	1.00	-1.3	0.41	-1.4	0.00	-1.6
A_30_P01022751			1.00	-1.3	0.31	-1.4	0.00	-1.6
A_55_P2183672	NM_018858	Pebp1	1.00	-1.2	0.32	-1.3	0.00	-1.6
A_30_P01029815			1.00	-1.3	0.86	-1.3	0.04	-1.6
A_30_P01020476			1.00	-1.3	0.40	-1.4	0.00	-1.7
A_30_P01026230			1.00	-1.2	0.42	-1.4	0.00	-1.7
A_55_P2014978	NM_007889	Dvl3	1.00	-1.3	0.73	-1.4	0.00	-1.8
A_30_P01032942			1.00	-1.3	0.31	-1.5	0.00	-1.8
A_55_P2230506	AK016424	4931402H11Rik	1.00	-1.5	0.95	-1.3	0.00	-1.8
A_55_P2031898	XM_001475136	LOC100045902	1.00	-1.2	0.39	-1.5	0.00	-1.8
A_30_P01024297			1.00	-1.3	0.30	-1.5	0.04	-1.8
A_30_P01022987			1.00	-1.4	0.95	-1.4	0.00	-1.9
A_52_P311031	AK016943		1.00	-1.4	0.19	-1.6	0.00	-1.9
A_55_P2086433	NM_145209	Oasl1	1.00	-1.5	0.00	-2.6	0.09	-2.1
A_66_P138976	NM_001164885	Lpin2	1.00	-1.2	0.95	-1.3	0.04	-2.1
A_55_P2042778	NM_153422	Pde5a	1.00	-1.3	0.73	-2.1	0.00	-3.5
A_51_P456208	NM_011575	Tff3	1.00	-2.7	0.05	-5.5	0.04	-5.4

3.3 LUNG

3.3.1 BaA Lung. Significant probe list. List of all significantly differentially expressed probes in at least 1 treatment group (FDR P ≤ 0.05, fold change ± 1.5) in response to sub-chronic oral exposure to 20, 40, and 80 mg/kg-bw/day benz(a)anthracene in the lung. The list is sorted from highest to lowest fold change in the 80 mg/kg-bw/day treatment group.

			**20 mg/kg-bw/day**		**40 mg/kg-bw/day**		**80 mg/kg-bw/day**	
Agilent Probe	Accession Number	Gene Symbol	*FDR P value*	*Fold change*	*FDR P value*	*Fold change*	*FDR P value*	*Fold change*
A_55_P2142226	NM_001034870	Serpina3h	0.08	8.0	0.51	2.5	0.00	8.0
A_55_P2032081	NM_016974	Dbp	0.54	1.6	0.00	2.8	0.00	3.5
A_55_P2032079	NM_016974	Dbp	0.52	1.6	0.00	2.7	0.00	3.5
A_51_P326191	NM_009251	Serpina3g	0.06	2.9	0.11	2.1	0.00	3.5
A_30_P01021631			0.90	-1.2	0.00	2.3	0.00	3.3
A_51_P234692	NR_003513	Neat1	0.89	-1.2	0.00	2.3	0.00	3.1
A_55_P1966774	XM_893705	Gm6930	0.08	3.5	0.61	1.5	0.01	3.1
A_30_P01025798			0.84	-1.2	0.01	2.0	0.00	2.9

A_55_P2157872	NM_001079695	Sfrs5	0.14	1.4	0.00	2.3	0.00	2.8
A_30_P01023251			0.84	-1.2	0.00	2.0	0.00	2.7
A_30_P01020135			0.81	-1.2	0.01	1.9	0.00	2.6
A_30_P01031894			0.98	-1.0	0.00	1.9	0.00	2.6
A_66_P124254	AK046653		0.80	1.2	0.00	2.0	0.00	2.5
A_30_P01029720			0.75	-1.3	0.03	1.9	0.00	2.5
A_55_P1964648	NM_001037719	Btla	0.28	1.6	0.00	2.4	0.00	2.5
A_51_P487818	NM_017399	Fabp1	0.63	1.4	0.04	1.8	0.00	2.5
A_51_P267933	NM_175692	Snhg11	0.95	1.1	0.34	1.6	0.00	2.5
A_30_P01022612			0.97	-1.0	0.00	1.8	0.00	2.4
A_51_P272106	NM_007705	Cirbp	0.58	1.3	0.00	1.8	0.00	2.4
A_51_P170959	AK005011	Proz	0.78	1.3	0.33	1.5	0.00	2.4
A_30_P01018771			0.99	-1.0	0.00	1.9	0.00	2.3
A_55_P2036495			0.58	1.2	0.00	1.8	0.00	2.3
A_55_P2115442	NM_053109	Clec2d	0.98	-1.0	0.00	2.0	0.00	2.3
A_30_P01030953			1.00	-1.0	0.00	2.0	0.00	2.2
A_55_P2018191	NM_145536	BC020535	0.11	1.5	0.00	1.8	0.00	2.2
A_52_P151240	XM_973835	Fam150a	0.74	1.3	0.11	1.7	0.00	2.2
A_55_P2116993	NM_016690	Hnrpdl	0.94	1.0	0.00	1.9	0.00	2.2
A_55_P1974452	NM_023684	Lime1	0.78	1.2	0.00	1.9	0.00	2.2
A_55_P2131964	NM_001081070	Pdia2	0.94	1.1	0.00	1.6	0.00	2.2
A_55_P2279807	AK160312	6720427I07Rik	0.93	-1.1	0.00	1.9	0.00	2.2
A_52_P38964	NM_001081962	Sap25	0.96	-1.0	0.00	1.8	0.00	2.2
A_52_P469789	NM_007588	Calcr	0.53	1.3	0.57	1.2	0.00	2.1
A_55_P2102419	NM_023684	Lime1	0.78	1.1	0.00	1.7	0.00	2.1
A_51_P360492	NM_008567	Mcm6	0.80	1.2	0.02	1.9	0.00	2.1
A_55_P2054098	XM_001475988	Gm9495	0.63	1.2	0.00	1.7	0.00	2.1
A_55_P2107155	NM_029182	Rasd2	0.56	1.4	0.00	1.9	0.00	2.1
A_55_P2032232	NM_011268	Rgs9	0.47	1.3	0.00	1.8	0.00	2.1
A_55_P1955078	NM_145580	Tmem149	0.33	1.3	0.00	1.9	0.00	2.1
A_51_P247542	NM_172799	Ttll6	0.99	1.0	0.03	1.6	0.00	2.1
A_30_P01022284			0.98	1.0	0.04	1.5	0.00	2.0
A_30_P01023697			0.86	1.1	0.06	1.6	0.00	2.0
A_55_P1990663	NM_007840	Ddx5	0.86	1.1	0.00	1.6	0.00	2.0
A_55_P2274378	AK035112	AW549542	0.93	1.1	0.01	1.7	0.00	2.0
A_55_P2071354	NM_177087	Fam179a	0.99	1.0	0.09	1.5	0.00	2.0
A_51_P362176	NM_133862	Fgg	0.80	1.3	0.55	1.3	0.00	2.0
A_51_P249313	NM_028705	Herc3	0.75	1.2	0.00	1.7	0.00	2.0
A_55_P2009752	NM_172563	Hlf	0.91	1.1	0.23	1.6	0.01	2.0
A_55_P2082929	NM_010389	H2-Ob	0.56	1.3	0.00	2.0	0.00	2.0
A_55_P2293351	AK082896	C430010C01	0.96	1.0	0.00	1.7	0.00	2.0
A_55_P2059019	NM_001163447	Mapk8ip3	0.99	1.0	0.00	1.7	0.00	2.0
A_55_P2129348	NM_153510	Pilra	0.04	1.8	0.00	1.7	0.00	2.0
A_55_P2408330	NM_028234	Prr8	0.99	1.0	0.00	1.6	0.00	2.0
A_55_P2095271	NM_153805	Pkn3	0.36	1.2	0.00	1.7	0.00	2.0
A_55_P2000409	NM_001002786	Rab44	0.28	1.4	0.00	1.7	0.00	2.0
A_66_P139946	XM_976924	E030019B13Rik	0.83	1.1	0.01	1.6	0.00	2.0
A_55_P2104975	NM_001168294	Serpina3f	0.52	1.7	0.10	1.9	0.03	2.0
A_30_P01027042			0.96	-1.0	0.01	1.7	0.00	1.9

A_52_P1016836	AV256778		0.99	1.0	0.00	1.7	0.00	1.9
A_30_P01025102			0.93	-1.1	0.00	1.7	0.00	1.9
A_55_P2181634	AV253706		0.99	-1.0	0.01	1.6	0.00	1.9
A_30_P01022593			0.96	1.0	0.00	1.6	0.00	1.9
A_55_P2177463	AK045070		0.95	-1.0	0.00	1.6	0.00	1.9
A_30_P01024984			0.91	-1.1	0.03	1.5	0.00	1.9
A_52_P390944	NM_016803	Chst3	0.64	1.2	0.00	1.8	0.00	1.9
A_55_P2079079	NM_009844	Cd19	0.73	1.2	0.00	2.2	0.00	1.9
A_55_P2154982	NM_009858	Cd8b1	0.55	1.6	0.34	1.5	0.03	1.9
A_55_P1969356	NM_145536	BC020535	0.36	1.4	0.00	1.7	0.00	1.9
A_55_P1981836	NM_145536	BC020535	0.67	1.2	0.02	1.6	0.00	1.9
A_52_P340408	NM_001039495	Ccdc108	0.96	-1.0	0.26	1.3	0.00	1.9
A_55_P2025675	NM_013811	Dnahc8	0.28	1.3	0.00	1.6	0.00	1.9
A_51_P155323	NM_010406	Hc	0.94	1.1	0.03	1.6	0.00	1.9
A_51_P449325	NM_008206	H2-Oa	0.11	1.4	0.00	1.9	0.00	1.9
A_55_P1958480	BC089308	LOC545005	0.86	1.1	0.00	1.5	0.00	1.9
A_51_P479352	NM_001033039	Klhdc9	0.85	1.1	0.05	1.4	0.00	1.9
A_52_P282500	NM_001039472	Kif21b	0.09	1.4	0.00	1.8	0.00	1.9
A_55_P2040815	NM_153545	Lrrc45	0.95	1.0	0.00	1.6	0.00	1.9
A_55_P1954231	NM_172492	Lrtm2	0.87	1.2	0.20	1.5	0.00	1.9
A_51_P258493	NM_011067	Per3	0.91	1.1	0.11	1.5	0.00	1.9
A_55_P2000354	NM_001081069	Rgs11	0.75	-1.2	0.05	1.5	0.00	1.9
A_55_P1969032	NM_011268	Rgs9	0.30	1.4	0.00	1.8	0.00	1.9
A_55_P1979893	NM_017376	Tef	0.96	1.1	0.10	1.6	0.00	1.9
A_51_P483576	AF138742		0.23	1.9	0.01	2.4	0.03	1.8
A_55_P2057283	AY170502		0.18	2.1	0.00	2.4	0.07	1.8
A_52_P308875	AK049998		0.96	1.1	0.00	2.3	0.02	1.8
A_30_P01021037			1.00	1.0	0.00	1.7	0.00	1.8
A_66_P115580	AK076360		0.11	1.4	0.00	1.6	0.00	1.8
A_30_P01025159			0.89	1.1	0.00	1.5	0.00	1.8
A_30_P01031112			0.97	-1.0	0.00	1.5	0.00	1.8
A_30_P01025535			0.93	-1.1	0.99	-1.0	0.02	1.8
A_30_P01018104			0.95	1.1	0.09	1.4	0.00	1.8
A_30_P01033086			0.94	1.0	0.01	1.4	0.00	1.8
A_55_P1988689	AK131832		0.80	1.2	0.23	1.4	0.00	1.8
A_55_P1985001			1.00	-1.0	0.16	1.4	0.00	1.8
A_52_P625277	NM_183220	Accs	0.20	1.2	0.00	1.5	0.00	1.8
A_55_P2049717	NM_007427	Agrp	0.96	1.0	0.05	1.5	0.00	1.8
A_52_P467449	NM_007440	Alox12	0.04	2.0	0.12	1.6	0.01	1.8
A_55_P2081164	NM_001169153	Cd300lf	0.28	1.5	0.31	1.4	0.01	1.8
A_52_P445387	NM_007714	Clk4	0.97	1.0	0.00	1.7	0.00	1.8
A_55_P2171008	NM_007714	Clk4	0.97	1.0	0.00	1.6	0.00	1.8
A_52_P679152	NM_001042634	Clk1	0.98	1.0	0.00	1.5	0.00	1.8
A_52_P70796	NM_007551	Cxcr5	0.19	1.5	0.00	1.8	0.00	1.8
A_66_P115531	NM_145151	Crebzf	0.99	-1.0	0.02	1.5	0.00	1.8
A_51_P284426	NM_030137	Cstad	0.06	1.5	0.00	1.7	0.00	1.8
A_51_P386304	NM_207678	Ccnl2	1.00	1.0	0.01	1.5	0.00	1.8
A_52_P51429	NM_153551	Dennd1c	0.48	1.3	0.00	1.5	0.00	1.8
A_51_P435339	NM_010149	Epor	0.57	1.2	0.00	1.7	0.00	1.8

A_51_P407879	NM_145382	Fam193b	1.00	-1.0	0.01	1.6	0.00	1.8
A_55_P2066230	NM_010407	Hck	0.50	1.5	0.51	1.3	0.01	1.8
A_51_P520966	NM_015790	Icosl	0.73	1.1	0.00	1.6	0.00	1.8
A_55_P2077053	NM_018746	Itih4	0.48	1.5	0.75	1.2	0.01	1.8
A_55_P2149500	NM_010630	Kifc2	0.99	1.0	0.00	1.6	0.00	1.8
A_55_P2063256	NM_010706	Lgals4	0.99	-1.0	0.07	1.5	0.00	1.8
A_51_P497100	NM_010706	Lgals4	0.94	-1.1	0.10	1.4	0.00	1.8
A_51_P279437	NM_029662	Mfsd2a	0.92	1.1	0.56	1.3	0.02	1.8
A_51_P341725	NM_008588	Mesp1	0.82	1.2	0.10	1.4	0.00	1.8
A_51_P163953	NM_008741	Nsg2	0.83	1.2	0.53	1.3	0.02	1.8
A_51_P261107	NM_139144	Ogt	0.94	-1.0	0.00	1.5	0.00	1.8
A_51_P282760	NM_011066	Per2	0.96	1.1	0.52	1.3	0.01	1.8
A_52_P63343	NM_001033302	Gm129	0.93	1.1	0.21	1.4	0.00	1.8
A_55_P2030282	NM_001029930	ENSMUSG00000068790	0.94	1.1	0.04	1.4	0.00	1.8
A_51_P215438	NM_011172	Prodh	0.90	1.1	0.01	1.5	0.00	1.8
A_55_P2133057	NM_001081070	Pdia2	0.96	1.0	0.04	1.5	0.00	1.8
A_55_P2039225	NM_001081155	Rap1gap	0.19	1.3	0.00	1.7	0.00	1.8
A_52_P574759	NM_009036	Rbpjl	0.97	1.0	0.42	1.3	0.01	1.8
A_55_P2002319	NM_027129	2310035K24Rik	0.79	1.3	0.83	1.1	0.03	1.8
A_52_P613498	NM_026127	4833420G17Rik	0.99	-1.0	0.00	1.7	0.00	1.8
A_55_P2022499	NM_025669	Sfrs18	1.00	-1.0	0.02	1.6	0.00	1.8
A_55_P2141306	NM_025292	Synj2bp	0.78	1.2	0.04	1.4	0.00	1.8
A_51_P295420	NM_145931	Zc3h7a	0.98	1.0	0.00	1.5	0.00	1.8
A_55_P2145224	AF290571		0.28	1.8	0.00	2.3	0.05	1.7
A_30_P01020858			0.58	1.2	0.00	1.7	0.00	1.7
A_30_P01032392			0.89	1.1	0.00	1.6	0.00	1.7
A_30_P01028387			0.98	-1.0	0.00	1.6	0.00	1.7
A_30_P01027568			0.98	-1.0	0.74	1.2	0.01	1.7
A_30_P01030240			0.78	-1.2	0.88	1.1	0.01	1.7
A_30_P01028753			0.85	1.2	0.54	1.3	0.01	1.7
A_30_P01018881			0.99	1.0	0.22	1.3	0.00	1.7
A_30_P01024344			1.00	-1.0	0.46	1.2	0.00	1.7
A_30_P01033468			0.69	1.2	0.03	1.4	0.00	1.7
A_66_P106716	NM_019774	Akap8	0.83	1.1	0.00	1.6	0.00	1.7
A_51_P108581	NM_177078	Adrbk2	0.83	1.1	0.02	1.4	0.00	1.7
A_52_P645862	NM_177322	Agtr1a	0.81	1.1	0.08	1.4	0.00	1.7
A_51_P365903	NM_026187	Ankzf1	0.65	1.1	0.00	1.4	0.00	1.7
A_66_P127175	NM_177268	Ankrd16	0.90	-1.1	0.07	1.4	0.00	1.7
A_55_P2089091	NM_178278	Caps2	0.70	1.2	0.01	1.5	0.00	1.7
A_51_P342652	NM_008339	Cd79b	0.41	1.3	0.00	2.1	0.00	1.7
A_65_P03022	NM_172587	Cdc14b	0.72	1.1	0.00	1.4	0.00	1.7
A_55_P2132403	NM_198170	BC059842	0.67	1.2	0.01	1.5	0.00	1.7
A_52_P329054	NM_023873	Cep70	1.00	-1.0	0.15	1.4	0.00	1.7
A_55_P1987904	AK138814	Chpf2	0.99	1.0	0.02	1.5	0.00	1.7
A_55_P2092904	NM_028119	Ddb2	0.98	1.0	0.02	1.4	0.00	1.7
A_52_P611798	NM_007840	Ddx5	0.70	1.1	0.00	1.6	0.00	1.7
A_52_P226127	NM_011805	Dido1	0.91	-1.1	0.00	1.5	0.00	1.7
A_55_P2106180	NM_001081330	Dnahc2	0.97	-1.0	0.28	1.4	0.00	1.7

A_55_P2079158	NM_177894	Fam154b	0.98	1.0	0.05	1.5	0.00	1.7
A_55_P2059337	NM_023229	Fastk	0.48	1.4	0.00	1.9	0.00	1.7
A_55_P1964664	NM_139149	Fus	0.52	1.2	0.00	1.5	0.00	1.7
A_55_P2156653	AK156069	Gpr174	0.73	1.1	0.00	1.5	0.00	1.7
A_52_P16232	NM_019439	Gabbr1	0.90	-1.1	0.13	1.4	0.00	1.7
A_51_P322972	NM_145419	Hkdc1	0.49	1.4	0.18	1.4	0.00	1.7
A_55_P2009187	NM_001039669	Iffo1	0.87	1.1	0.01	1.5	0.00	1.7
A_66_P128434	NM_001122733	Kit	0.56	1.4	0.18	1.5	0.02	1.7
A_51_P501453	NM_022430	Ms4a8a	0.34	1.4	0.22	1.3	0.01	1.7
A_55_P2347976	NR_029382	Mirhg1	0.96	-1.0	0.01	1.5	0.00	1.7
A_52_P333097	AK033008	Mcoln3	0.48	1.5	0.88	1.1	0.03	1.7
A_55_P1955015	NM_178440	Myo1g	0.23	1.4	0.02	1.5	0.00	1.7
A_52_P357484	NM_172766	Nfrkb	0.83	1.1	0.00	1.4	0.00	1.7
A_51_P383194	NM_008804	Pde9a	0.54	1.3	0.00	1.6	0.00	1.7
A_66_P129710	NM_175437	Pion	0.78	1.2	0.18	1.4	0.00	1.7
A_51_P356265	NM_016845	Acrbp	0.95	-1.0	0.03	1.5	0.00	1.7
A_55_P2105638	NM_001033348	Ralgapa2	0.77	1.1	0.00	1.5	0.00	1.7
A_55_P2068096	NM_001081155	Rap1gap	0.23	1.3	0.00	1.6	0.00	1.7
A_51_P339822	NM_138630	Arhgap4	0.38	1.3	0.00	1.5	0.00	1.7
A_51_P258473	NM_001081121	4931429I11Rik	0.93	1.1	0.03	1.6	0.00	1.7
A_55_P2283310	AK039829	5830428M24Rik	0.95	1.0	0.28	1.3	0.00	1.7
A_55_P2372228	DT902180	A430104N18Rik	0.81	1.1	0.01	1.6	0.00	1.7
A_55_P2367415	AK035805	A630026N12Rik	0.99	-1.0	0.07	1.4	0.00	1.7
A_55_P2340448	AK045415	B230114P17Rik	0.75	1.2	0.01	1.6	0.00	1.7
A_51_P267278	NM_021301	Slc15a2	0.99	1.0	0.00	1.6	0.00	1.7
A_52_P19016	NM_016680	Sfrs16	0.94	1.0	0.01	1.5	0.00	1.7
A_55_P2083098	NM_001163421	Tatdn3	0.93	1.0	0.05	1.3	0.00	1.7
A_55_P2007713	NM_053082	Tspan4	0.59	1.4	0.33	1.4	0.01	1.7
A_66_P124146	NM_177384	Ttc16	0.94	-1.1	0.09	1.5	0.00	1.7
A_51_P316951	NM_001167860	Wipf3	0.41	1.3	0.05	1.4	0.00	1.7
A_55_P2044252	NM_001164597	Znf512b	0.79	1.1	0.00	1.5	0.00	1.7
A_55_P2072233	NM_028325	Zcchc12	0.98	-1.0	0.33	1.5	0.04	1.7
A_55_P2088145			0.00	2.0	0.06	1.6	0.01	1.6
A_55_P2111322	AY170578		0.10	1.9	0.00	2.1	0.04	1.6
A_30_P01031844			0.34	1.3	0.00	1.9	0.00	1.6
A_30_P01024593			0.97	1.0	0.05	1.6	0.01	1.6
A_30_P01027066			0.86	1.1	0.05	1.5	0.01	1.6
A_30_P01029746			0.99	-1.0	0.04	1.5	0.01	1.6
A_30_P01019380			0.86	1.1	0.02	1.5	0.00	1.6
A_30_P01027782			0.98	1.0	0.01	1.5	0.00	1.6
A_55_P2085940	AK165743		0.71	1.1	0.00	1.5	0.00	1.6
A_30_P01031674			0.80	-1.2	0.97	-1.0	0.02	1.6
A_30_P01030888			0.83	-1.2	0.88	-1.1	0.02	1.6
A_30_P01030677			0.99	1.0	0.21	1.4	0.01	1.6
A_30_P01032960			0.85	1.1	0.00	1.4	0.00	1.6
A_55_P2144476			0.83	1.1	0.04	1.3	0.00	1.6
A_30_P01032788			0.92	-1.1	0.07	1.4	0.00	1.6
A_51_P378381	AK017076		0.92	-1.1	0.23	1.3	0.00	1.6
A_66_P138567			0.80	1.1	0.05	1.4	0.00	1.6

A_30_P01019990			0.90	1.1	0.11	1.3	0.00	1.6
A_30_P01029835			0.78	1.1	0.03	1.4	0.00	1.6
A_30_P01028297			0.99	1.0	0.05	1.4	0.00	1.6
A_55_P2016316	NM_011917	Xrn2	0.50	1.2	0.01	1.3	0.00	1.6
A_55_P2065926	NM_017476	Akap8l	0.98	-1.0	0.08	1.3	0.00	1.6
A_51_P179907	NM_145220	Appl2	0.93	1.1	0.17	1.3	0.00	1.6
A_55_P2101776	NM_001008533	Adora1	0.74	1.2	0.00	1.5	0.00	1.6
A_52_P586141	NM_001037724	Adcy7	0.75	1.2	0.20	1.3	0.00	1.6
A_55_P1992849	NM_013462	Adrb3	0.90	1.1	0.06	1.6	0.02	1.6
A_55_P1988010	NM_001164727	LOC100038847	0.94	1.0	0.04	1.2	0.00	1.6
A_55_P2002849	NM_175178	Aifm3	0.69	1.2	0.08	1.3	0.00	1.6
A_51_P247249	NM_009662	Alox5	0.23	1.5	0.39	1.3	0.00	1.6
A_52_P330359	NM_178755	Agbl2	0.86	1.1	0.00	1.4	0.00	1.6
A_52_P277104	NM_001033350	Bank1	0.55	1.3	0.00	1.8	0.01	1.6
A_55_P2162324	NM_009759	Bmx	0.97	-1.0	0.29	1.3	0.00	1.6
A_55_P2036472	NM_001161832	T2	0.72	-1.2	0.20	1.2	0.00	1.6
A_51_P316042	NM_175362	Card11	0.68	1.2	0.07	1.3	0.00	1.6
A_52_P422494	NM_145634	Cd300lf	0.40	1.5	0.37	1.4	0.01	1.6
A_55_P2060072	NM_007712	Clk2	0.90	1.1	0.17	1.3	0.00	1.6
A_51_P331752	NM_011330	Ccl11	0.90	1.1	0.34	1.3	0.01	1.6
A_55_P2023542	NM_007719	Ccr7	0.72	1.2	0.02	1.6	0.01	1.6
A_52_P35304	NM_007692	Chkb	0.96	-1.0	0.00	1.5	0.00	1.6
A_55_P2169415	NM_001037711	Cgn	0.91	1.1	0.62	1.2	0.00	1.6
A_55_P2086860	NM_207280	Ccdc121	0.99	1.0	0.29	1.4	0.02	1.6
A_55_P2004711	NM_177088	Ccdc45	0.93	1.0	0.00	1.5	0.00	1.6
A_55_P2079922	NM_001165929	Ccdc78	0.85	-1.1	0.13	1.3	0.00	1.6
A_55_P1971724	NM_007758	Cr2	0.30	1.3	0.00	1.7	0.00	1.6
A_52_P624434	NM_175526	Clec1a	0.87	1.1	0.34	1.3	0.01	1.6
A_55_P2099980	NM_001081335	Cul9	0.39	1.2	0.00	1.4	0.00	1.6
A_51_P109258	NM_138686	Cys1	1.00	1.0	0.59	1.2	0.02	1.6
A_51_P255456	NM_009994	Cyp1b1	0.04	2.5	0.49	1.4	0.16	1.6
A_55_P1957957	NM_027883	Dhx34	0.71	1.1	0.01	1.3	0.00	1.6
A_55_P2184023	XM_355197	Dnahc7a	0.89	1.1	0.83	-1.1	0.00	1.6
A_55_P2111355	NM_178444	Egfl7	0.92	1.1	0.00	1.5	0.00	1.6
A_51_P455807	NM_133838	Ehd4	0.91	1.1	0.05	1.4	0.00	1.6
A_51_P115005	NM_010104	Edn1	0.60	1.3	0.15	1.4	0.01	1.6
A_51_P214269	NM_023580	Epha1	1.00	1.0	0.00	1.4	0.00	1.6
A_55_P2037454	NM_023794	Etv5	0.70	1.2	0.01	1.5	0.00	1.6
A_55_P2011877	NM_012011	Eif2s3y	0.72	1.2	0.63	1.2	0.01	1.6
A_55_P2150741	NM_177788	Exoc3l	0.30	1.3	0.07	1.4	0.00	1.6
A_55_P2255449	AK033778	AI663975	0.55	1.2	0.00	1.6	0.00	1.6
A_51_P140641	NM_145570	Fam176a	0.67	1.2	0.04	1.5	0.00	1.6
A_51_P153063	NM_029310	Fabp12	0.75	1.2	0.27	1.3	0.01	1.6
A_51_P154485	NM_145141	Fcrla	0.71	1.3	0.00	1.8	0.04	1.6
A_55_P2145506	U04807	Flt3l	0.90	1.1	0.08	1.3	0.00	1.6
A_55_P2128646	NM_020567	Gmnn	0.80	1.1	0.02	1.4	0.00	1.6
A_55_P2073084	NM_027901	Gtf3c2	0.66	1.1	0.00	1.4	0.00	1.6
A_52_P351669	NM_027375	Gcc2	0.56	1.2	0.00	1.5	0.00	1.6
A_51_P124089	NM_019581	Gtpbp2	0.95	1.0	0.01	1.4	0.00	1.6

A_55_P2159600	NM_145617	Herc1	0.91	1.1	0.00	1.4	0.00	1.6
A_52_P283910	NM_016690	Hnrpdl	0.94	1.0	0.00	1.4	0.00	1.6
A_55_P2135064	NM_008204	H2-M2	0.63	1.4	0.46	1.3	0.04	1.6
A_55_P1982762	NM_133943	Hsd3b7	0.10	1.4	0.00	1.5	0.00	1.6
A_55_P2054708	XM_001473132	LOC100044824	0.93	-1.1	0.03	1.5	0.00	1.6
A_55_P2087622	NM_010215	Il4i1	0.12	1.5	0.05	1.4	0.00	1.6
A_55_P2004084	NM_028833	Iqce	0.66	1.2	0.01	1.4	0.00	1.6
A_55_P2113498	NM_010654	Klrd1	0.84	1.2	0.04	1.7	0.03	1.6
A_55_P1980986	NM_001001492	Lca5l	0.44	1.3	0.01	1.4	0.00	1.6
A_55_P2063257	NM_010706	Lgals4	0.99	-1.0	0.26	1.3	0.01	1.6
A_51_P130079	NM_008511	Lrmp	0.63	1.2	0.00	1.6	0.00	1.6
A_66_P132222	NM_053015	Mlph	0.56	1.2	0.00	1.5	0.00	1.6
A_52_P167278	NM_172308	Mthfd1l	0.68	1.2	0.42	1.2	0.00	1.6
A_52_P204689	NM_009006	Map4k2	0.83	1.1	0.04	1.4	0.00	1.6
A_55_P2076866	NM_001162977	Megf6	0.70	1.2	0.11	1.3	0.00	1.6
A_51_P246387	NM_010918	Nktr	0.91	-1.1	0.01	1.4	0.00	1.6
A_55_P2021565	NM_001164034	Ntf3	0.57	1.2	0.00	1.5	0.00	1.6
A_55_P2050268	NM_172142	Nfkbid	0.35	1.3	0.02	1.5	0.00	1.6
A_55_P2148534	BC096461	Nr1d2	0.72	1.2	0.05	1.5	0.02	1.6
A_51_P521374	NM_026341	Nudt13	0.95	1.0	0.01	1.4	0.00	1.6
A_55_P2146002	NM_172990	Pank4	0.91	1.1	0.03	1.4	0.00	1.6
A_55_P1987439	NM_133779	Pigt	0.96	-1.0	0.98	1.0	0.03	1.6
A_66_P122158	NR_003518	Pisd-ps3	0.84	-1.1	0.53	1.2	0.01	1.6
A_55_P2014755	NM_001110796	Pclo	0.89	1.1	0.03	1.4	0.00	1.6
A_55_P2069592	NM_001024927	Pitpnm3	0.94	1.1	0.11	1.4	0.00	1.6
A_55_P2108708	NM_020574	Kcne3	0.48	1.4	0.18	1.4	0.02	1.6
A_55_P1986341	NR_002858	Gm4956	0.82	-1.2	0.73	-1.2	0.04	1.6
A_55_P2128456	XM_001477292	Gm9571	0.97	1.0	0.03	1.4	0.00	1.6
A_52_P107234	NM_177806	Prpf39	0.88	1.1	0.00	1.4	0.00	1.6
A_51_P250358	NM_177806	Prpf39	1.00	-1.0	0.07	1.3	0.00	1.6
A_55_P2053958	NM_030566	Rabep2	0.77	1.1	0.00	1.4	0.00	1.6
A_52_P378968	NM_009061	Rgs2	0.83	1.2	0.06	1.5	0.01	1.6
A_55_P2137431	NM_001001882	Rtel1	0.99	1.0	0.09	1.3	0.00	1.6
A_55_P2340593	AK007545	1810019D21Rik	0.75	-1.2	0.50	1.2	0.01	1.6
A_55_P2180481	XM_001478842	1810020O05Rik	1.00	1.0	0.06	1.4	0.00	1.6
A_55_P2126448	NR_027821	1810032O08Rik	0.88	-1.1	0.03	1.5	0.00	1.6
A_55_P2092364	NM_001039552	2210404J11Rik	0.44	1.1	0.00	1.4	0.00	1.6
A_55_P2373852	AK009987	2310058N22Rik	0.82	1.3	0.04	1.9	0.08	1.6
A_51_P157270	XM_126991	3830431G21Rik	0.83	1.1	0.10	1.3	0.00	1.6
A_55_P2144597	NM_001123370	9030025P20Rik	0.95	1.0	0.09	1.3	0.00	1.6
A_52_P337427	NM_027829	9030607L17Rik	1.00	-1.0	0.27	1.3	0.00	1.6
A_55_P2361652	AK088035	C230085N15Rik	0.65	1.4	0.44	1.3	0.04	1.6
A_55_P2029892	XM_916666	E030019B06Rik	0.97	1.0	0.43	1.3	0.01	1.6
A_55_P2010197	NM_144834	Serpina10	0.23	1.5	0.05	1.6	0.01	1.6
A_51_P424641	NM_001167691	Sirt4	0.97	1.0	0.02	1.3	0.00	1.6
A_51_P349727	NM_134154	Slc25a45	0.28	1.2	0.00	1.5	0.00	1.6
A_51_P113403	NM_178743	Slc26a11	0.57	1.2	0.01	1.4	0.00	1.6
A_55_P2094706	NM_011441	Sox17	0.73	1.3	0.24	1.5	0.04	1.6
A_52_P550147	AJ584850	Sned1	0.92	-1.1	0.17	1.3	0.00	1.6

A_52_P681488	NM_026343	Stx17	0.61	1.2	0.00	1.4	0.00	1.6
A_51_P267314	NM_178650	Tbc1d10c	0.91	1.1	0.07	1.5	0.00	1.6
A_55_P2168168	NM_031384	Tex11	0.28	1.4	0.01	1.5	0.00	1.6
A_51_P336721	NM_025372	Tipin	0.30	1.3	0.00	1.7	0.00	1.6
A_51_P368591	NM_053254	Tle6	0.99	-1.0	0.08	1.3	0.00	1.6
A_55_P2079103	NM_133992	Usp52	0.98	-1.0	0.00	1.5	0.00	1.6
A_66_P139647	NM_177697	Vwa3a	0.74	-1.2	0.34	1.3	0.00	1.6
A_51_P394847	NR_024599	Gm11346	0.09	1.4	0.01	1.5	0.00	1.6
A_55_P2034250	NM_001013786	Zfp187	0.79	1.1	0.01	1.5	0.00	1.6
A_52_P207635	NM_001033261	Zfc3h1	1.00	-1.0	0.02	1.4	0.00	1.6
A_55_P2122688	NM_019831	Zmym3	0.86	1.1	0.00	1.4	0.00	1.6
A_30_P01028905			0.52	1.3	0.00	1.7	0.00	1.5
A_30_P01030419			0.91	1.1	0.01	1.5	0.00	1.5
A_30_P01027299			0.95	1.0	0.00	1.5	0.00	1.5
A_55_P2040873	AK144847		0.99	-1.0	0.48	1.3	0.04	1.5
A_30_P01021129			0.95	-1.0	0.26	1.3	0.02	1.5
A_30_P01023567			1.00	-1.0	0.24	1.3	0.02	1.5
A_30_P01031056			0.87	-1.1	0.06	1.4	0.01	1.5
A_51_P485472	AK029938		0.99	-1.0	0.47	1.2	0.01	1.5
A_30_P01028374			1.00	-1.0	0.17	1.3	0.01	1.5
A_30_P01029770			0.98	-1.0	0.03	1.4	0.00	1.5
A_30_P01026904			0.86	-1.1	0.06	1.3	0.00	1.5
A_30_P01021437			1.00	1.0	0.04	1.3	0.00	1.5
A_55_P1995133			0.88	1.1	0.16	1.3	0.00	1.5
A_30_P01024871			0.99	-1.0	0.05	1.4	0.00	1.5
A_55_P1988009			0.93	1.1	0.19	1.3	0.00	1.5
A_55_P2184449	AK134871		0.98	-1.0	0.06	1.3	0.00	1.5
A_55_P1964658			0.29	1.2	0.00	1.4	0.00	1.5
A_30_P01032525			0.98	-1.0	0.17	1.3	0.00	1.5
A_30_P01024315			0.93	1.0	0.00	1.4	0.00	1.5
A_52_P336768			0.38	1.2	0.01	1.3	0.00	1.5
A_66_P109220	AK077292		0.74	1.1	0.06	1.3	0.00	1.5
A_55_P2115260	AK148532		0.95	1.0	0.01	1.4	0.00	1.5
A_51_P184849	NM_001162415	Pfkfb2	0.97	-1.0	0.11	1.3	0.00	1.5
A_55_P2028474	NM_001098225	Adam22	0.87	1.1	0.22	1.3	0.00	1.5
A_52_P203691	NM_207231	Arl5c	0.34	1.5	0.14	1.5	0.03	1.5
A_55_P2108248	NM_026639	Art4	0.72	1.2	0.15	1.3	0.00	1.5
A_55_P1962906	NM_001146060	Als2cl	0.92	1.0	0.00	1.4	0.00	1.5
A_51_P260658	NM_172935	Amdhd2	0.14	1.2	0.00	1.3	0.00	1.5
A_66_P117058	NM_173765	Aasdh	0.85	1.1	0.03	1.3	0.00	1.5
A_55_P2020306	NM_173765	Aasdh	0.99	-1.0	0.02	1.3	0.00	1.5
A_55_P2049582	NM_146085	Apbb3	0.92	-1.1	0.00	1.4	0.00	1.5
A_51_P201982	NM_007426	Angpt2	0.71	1.2	0.00	1.6	0.00	1.5
A_55_P2042156	NM_007377	Aatk	0.52	1.2	0.00	1.4	0.00	1.5
A_65_P09090	NM_011790	Arih2	0.54	1.1	0.00	1.4	0.00	1.5
A_55_P1957922	NM_027560	Arrdc2	0.94	1.0	0.04	1.3	0.00	1.5
A_55_P2088440	NM_027560	Arrdc2	0.95	1.0	0.05	1.4	0.00	1.5
A_51_P254425	NM_009644	Ahrr	0.06	1.5	0.02	1.4	0.00	1.5
A_55_P1955691	NM_001081264	Alg6	0.98	1.0	0.00	1.4	0.00	1.5

A_55_P1971074	NM_015804	Atp11a	0.91	1.1	0.10	1.3	0.00	1.5
A_55_P1985428	NM_001111111	Atg16l2	0.98	-1.0	0.00	1.4	0.00	1.5
A_55_P2142430	NM_001033350	Bank1	0.70	1.2	0.02	1.6	0.01	1.5
A_51_P379443	NM_001025392	Bclaf1	0.82	1.1	0.00	1.3	0.00	1.5
A_55_P2157093	NM_025778	Bcl2l14	0.87	1.1	0.59	1.2	0.03	1.5
A_55_P2079076	NM_001044308	Cacna1i	0.82	1.1	0.00	1.5	0.00	1.5
A_55_P2020341	NM_178597	Camk2g	0.73	1.1	0.00	1.3	0.00	1.5
A_55_P2142028	NM_030179	Clip4	0.71	-1.2	0.96	1.0	0.01	1.5
A_51_P277006	NM_175140	Chst8	0.94	1.1	0.71	1.1	0.03	1.5
A_55_P1960216	NM_009842	Cd151	0.95	1.0	0.83	1.1	0.00	1.5
A_55_P1978216	NM_134158	AF251705	0.11	1.6	0.62	1.2	0.04	1.5
A_55_P2029846	NM_001113283	BC031353	0.94	1.0	0.00	1.5	0.00	1.5
A_55_P1962354	NM_183182	BC055111	0.95	-1.0	0.64	-1.1	0.00	1.5
A_52_P167535	NM_001110506	BC060267	0.99	-1.0	0.10	1.4	0.00	1.5
A_55_P1999666	NM_012018	Cep110	0.93	1.0	0.00	1.4	0.00	1.5
A_65_P15875	NM_133913	Chpf2	0.14	1.2	0.00	1.3	0.00	1.5
A_66_P102733	NM_025455	Ccdc28b	0.93	-1.1	0.17	1.3	0.00	1.5
A_66_P106421	NM_029286	Ccdc30	0.96	-1.0	0.11	1.4	0.01	1.5
A_51_P156135	NM_026222	Ccdc39	0.95	1.0	0.25	1.3	0.01	1.5
A_55_P2079928	NM_201362	Ccdc68	0.77	1.2	0.06	1.4	0.01	1.5
A_52_P456448	NM_001162979	Ccdc81	0.99	-1.0	0.72	1.1	0.01	1.5
A_55_P2014635	NM_025534	Ccdc82	0.93	1.1	0.13	1.3	0.00	1.5
A_55_P2038106	NM_009978	Cst8	0.72	1.2	0.34	1.3	0.01	1.5
A_51_P337662	NM_172779	Ddx26b	0.97	-1.0	0.08	1.4	0.00	1.5
A_55_P2029612	NM_145742	Dhx35	0.81	1.1	0.00	1.4	0.00	1.5
A_55_P1985793	NM_177030	Dock6	0.91	1.1	0.04	1.3	0.00	1.5
A_52_P494380	NM_199011	Dgkq	0.87	-1.1	0.00	1.4	0.00	1.5
A_52_P425651	XM_129357	D19Ertd652e	0.95	-1.1	0.62	1.2	0.01	1.5
A_52_P578790	NM_013739	Dok3	0.51	1.3	0.03	1.5	0.01	1.5
A_52_P399934	NM_010090	Dusp2	0.48	1.3	0.16	1.4	0.03	1.5
A_55_P1962149	NM_019536	Dnahc10	0.86	-1.1	0.56	1.2	0.02	1.5
A_55_P1952449	NM_026314	Dyx1c1	0.90	1.1	0.06	1.3	0.00	1.5
A_51_P204252	NM_172497	Efhb	1.00	-1.0	0.24	1.3	0.02	1.5
A_51_P286357	NM_139138	Emr4	0.10	1.6	0.14	1.4	0.02	1.5
A_55_P2022861	NM_001039176	Elovl1	0.62	1.3	0.33	1.3	0.01	1.5
A_51_P482043	NM_175266	Epm2aip1	0.82	1.1	0.01	1.4	0.00	1.5
A_52_P37757	NM_026146	Eps8l1	0.97	1.0	0.17	1.2	0.00	1.5
A_55_P1998396	NM_013719	Eif2ak4	0.97	1.0	0.01	1.3	0.00	1.5
A_55_P2010332	NM_007968	Ewsr1	0.79	1.1	0.01	1.3	0.00	1.5
A_55_P2074771	NM_001081188	Exosc7	0.69	1.1	0.00	1.5	0.00	1.5
A_55_P1997282	NM_026604	Fam135a	0.90	-1.1	0.20	1.2	0.00	1.5
A_51_P104125	NM_176836	Fam76b	0.81	1.1	0.02	1.3	0.00	1.5
A_51_P512783	NM_025476	Fam82b	0.60	1.2	0.03	1.4	0.00	1.5
A_51_P378298	NM_026976	Faim3	0.07	1.5	0.00	1.6	0.00	1.5
A_55_P2034670	NM_013911	Fbxl12	0.98	1.0	0.01	1.5	0.00	1.5
A_55_P2078695	NM_177076	Fbxl13	0.94	1.0	0.46	1.2	0.00	1.5
A_51_P149373	NM_028149	Fbxl20	0.42	1.2	0.00	1.3	0.00	1.5
A_55_P2117345	NM_013517	Fcer2a	0.15	1.5	0.01	1.6	0.01	1.5
A_52_P644690	NM_013520	Flt3l	0.83	1.1	0.00	1.4	0.00	1.5

A_51_P509808	NM_018828	Fnbp4	0.86	1.1	0.10	1.4	0.00	1.5
A_55_P2114776	NM_015764	Greb1	0.90	1.1	0.52	1.2	0.03	1.5
A_55_P2045896	NM_010275	Gdnf	0.72	1.2	0.19	1.4	0.01	1.5
A_55_P2011943	NM_027898	Gramd1a	0.91	1.1	0.03	1.3	0.00	1.5
A_55_P2178378	NR_002840	Gas5	0.92	-1.1	0.04	1.4	0.00	1.5
A_52_P263658	NM_008236	Hes2	0.74	1.2	0.04	1.5	0.02	1.5
A_51_P214306	NM_145462	Haus4	0.65	1.1	0.03	1.3	0.00	1.5
A_55_P1973178	NM_027999	Haus5	0.97	1.0	0.03	1.3	0.00	1.5
A_55_P2017082	NM_182650	Hnrnpa2b1	0.92	1.1	0.04	1.4	0.00	1.5
A_55_P1993789	NM_016957	Hmgn2	0.44	1.3	0.37	1.3	0.01	1.5
A_55_P2002103	NM_001142701	Hmha1	0.55	1.2	0.05	1.4	0.00	1.5
A_55_P2004761	NM_001033978	H2-Eb2	0.32	1.4	0.17	1.3	0.02	1.5
A_66_P130916	NM_010389	H2-Ob	0.40	1.3	0.00	1.7	0.01	1.5
A_55_P2036813	NM_030082	Hist3h2ba	0.76	1.2	0.21	1.3	0.00	1.5
A_55_P1971804	NM_199198	Hdac10	0.87	1.0	0.00	1.4	0.00	1.5
A_51_P292073	NM_026897	Haghl	0.85	1.1	0.05	1.3	0.00	1.5
A_55_P2178972	XM_001474677	LOC100045652	0.95	1.0	0.07	1.3	0.00	1.5
A_55_P2097178	NM_008325	Idua	0.77	1.2	0.11	1.4	0.01	1.5
A_51_P505696	NM_008916	Inpp5k	0.80	1.1	0.03	1.4	0.00	1.5
A_51_P355360	NM_010589	Jak3	0.87	1.1	0.41	1.3	0.03	1.5
A_52_P609334	NM_030075	Klhdc8b	0.99	1.0	0.19	1.3	0.00	1.5
A_55_P2067895	NM_133922	Krba1	0.96	-1.0	0.00	1.3	0.00	1.5
A_51_P354706	NM_010094	Lefty1	0.00	1.5	0.05	1.4	0.00	1.5
A_55_P2028365	NM_001170788	Lrrc36	0.88	-1.1	0.52	1.2	0.02	1.5
A_55_P2102175	NM_025808	Lztr1	0.78	1.1	0.00	1.3	0.00	1.5
A_55_P2023783	NM_172736	Leng8	0.99	-1.0	0.12	1.3	0.00	1.5
A_55_P2005946	NM_026915	Lyzl4	0.97	-1.0	0.38	1.2	0.00	1.5
A_55_P2068560	NM_026915	Lyzl4	0.99	-1.0	0.09	1.4	0.00	1.5
A_51_P481644	NM_145442	Mbip	0.90	1.1	0.26	1.2	0.00	1.5
A_51_P455997	NR_027652	Meg3	0.91	1.1	0.51	1.3	0.03	1.5
A_51_P497692	NM_023788	Mageh1	0.79	1.1	0.31	1.2	0.01	1.5
A_55_P2130388	NM_138315	Mical1	0.32	1.2	0.00	1.4	0.00	1.5
A_52_P364130	NM_016896	Map3k14	0.76	1.1	0.01	1.4	0.00	1.5
A_55_P2077068	NM_172833	Malt1	0.65	1.2	0.00	1.4	0.00	1.5
A_55_P2111907	NM_027988	Noxo1	0.95	1.1	0.53	1.2	0.01	1.5
A_55_P1953683	NM_010945	Nsmaf	0.82	1.1	0.00	1.4	0.00	1.5
A_51_P519251	NM_019738	Nupr1	1.00	-1.0	0.06	1.3	0.00	1.5
A_52_P303891	NM_011584	Nr1d2	0.71	1.2	0.18	1.3	0.01	1.5
A_66_P127567	NM_016813	Nxf1	0.98	1.0	0.01	1.4	0.00	1.5
A_55_P2029191	NM_183408	Pde4a	0.92	1.1	0.13	1.3	0.00	1.5
A_52_P269158	NM_001003948	Pid1	0.21	1.3	0.00	1.5	0.00	1.5
A_55_P2052048	NM_028300	Pih1d2	0.96	-1.0	0.37	1.3	0.02	1.5
A_66_P104473	NM_015810	Polg2	0.73	1.1	0.00	1.4	0.00	1.5
A_55_P2122255	AJ251804	Polm	0.89	1.1	0.00	1.4	0.00	1.5
A_55_P2017055	NM_175429	Kctd12b	0.69	1.2	0.07	1.4	0.01	1.5
A_55_P2146413	NM_001085536	Gm13178	0.86	1.1	0.06	1.3	0.00	1.5
A_66_P116860	XM_001473103	Gm2370	1.00	1.0	0.42	1.2	0.00	1.5
A_55_P2115189	XM_001477969	Gm3796	0.98	-1.0	0.21	1.3	0.01	1.5
A_55_P2006677	XM_001473218	Gm7969	0.89	1.1	0.24	1.2	0.00	1.5

A_55_P2072631	NM_001083810	Prr5l	0.51	1.3	0.18	1.3	0.01	1.5
A_55_P2167269	NM_008793	Pcsk4	0.85	-1.1	0.04	1.3	0.00	1.5
A_52_P487362	NM_028980	Ppp4r4	0.96	-1.0	0.11	1.3	0.01	1.5
A_52_P114722	NM_001077705	Ptpn6	0.04	1.4	0.01	1.4	0.00	1.5
A_55_P1963533	NM_001003672	Pcdhac2	0.98	1.0	0.55	1.2	0.04	1.5
A_51_P310949	NM_008911	Ppox	0.98	-1.0	0.87	1.1	0.01	1.5
A_51_P257065	NM_018786	Prpf40b	0.88	1.1	0.02	1.3	0.00	1.5
A_55_P2000603	NM_009057	Rag1ap1	0.78	1.1	0.00	1.4	0.00	1.5
A_55_P2078365	NM_173402	Rgs12	0.68	1.2	0.00	1.5	0.00	1.5
A_55_P2027117	NM_019492	Rgs3	0.63	1.2	0.03	1.3	0.00	1.5
A_51_P278653	NM_023396	Rprm	0.74	1.2	0.40	1.3	0.04	1.5
A_55_P2175206	NM_175023	Rbbp6	0.83	1.1	0.18	1.2	0.00	1.5
A_66_P138289	NM_134006	Rdh5	0.96	1.0	0.03	1.3	0.00	1.5
A_51_P474454	NM_026001	Rnaseh2b	0.15	1.3	0.04	1.3	0.00	1.5
A_55_P2041090	NM_001037742	1110034B05Rik	0.66	1.1	0.00	1.3	0.00	1.5
A_52_P478745	NM_026110	1810007M14Rik	1.00	1.0	0.05	1.3	0.00	1.5
A_55_P2197338	AK008218	2010012P19Rik	0.92	1.1	0.27	1.2	0.00	1.5
A_52_P638513	NR_027965	2310061J03Rik	0.60	1.2	0.01	1.4	0.00	1.5
A_51_P144770	BC016099	2410002F23Rik	0.89	1.1	0.01	1.4	0.00	1.5
A_55_P2415930	AK013749	2900064F13Rik	0.76	1.1	0.46	1.2	0.00	1.5
A_66_P105874	NM_030192	4930562C15Rik	0.87	1.1	0.34	1.3	0.03	1.5
A_55_P2349148	AK017909	5830408B19Rik	0.90	1.1	0.93	1.0	0.02	1.5
A_51_P301636	NM_001109685	9030409G11Rik	0.84	1.1	0.88	1.1	0.02	1.5
A_51_P241213	XM_896385	A030005L19Rik	0.70	1.1	0.13	1.2	0.00	1.5
A_51_P158073	NM_175687	A230050P20Rik	0.86	1.1	0.00	1.4	0.00	1.5
A_51_P118779	NM_172725	C330006K01Rik	0.69	1.2	0.02	1.4	0.00	1.5
A_55_P2047621	NM_030021	D730039F16Rik	0.80	1.1	0.23	1.3	0.01	1.5
A_55_P2331546	AK081964	D830026I12Rik	0.80	1.1	0.22	1.3	0.01	1.5
A_52_P876147	XM_001474855	E030010N08Rik	0.89	1.1	0.06	1.3	0.00	1.5
A_55_P2000823	NM_133242	Rbm39	0.94	-1.0	0.02	1.3	0.00	1.5
A_55_P2031471	NM_001034060	Rufy4	0.61	1.3	0.71	1.2	0.03	1.5
A_52_P257686	NM_028456	Rwdd3	0.60	1.3	0.26	1.3	0.02	1.5
A_51_P155747	NM_028776	Scyl3	0.91	1.1	0.34	1.3	0.04	1.5
A_55_P2052425	NM_145482	Setd4	0.95	1.0	0.05	1.4	0.00	1.5
A_51_P388847	NM_145934	Stap2	0.81	1.1	0.04	1.3	0.00	1.5
A_52_P171791	NM_001031814	Smg1	0.86	1.1	0.00	1.4	0.00	1.5
A_55_P2030155	NM_175316	Slco2b1	0.93	1.0	0.00	1.4	0.00	1.5
A_55_P2079009	NM_175316	Slco2b1	0.90	1.1	0.18	1.3	0.00	1.5
A_55_P1953301	NM_146126	Sord	0.77	1.2	0.10	1.4	0.00	1.5
A_51_P374549	NM_027617	Spata1	0.90	1.1	0.16	1.3	0.00	1.5
A_55_P1958986	NM_178914	Spata7	0.89	1.1	0.00	1.4	0.00	1.5
A_55_P2113703	NM_019866	Spib	0.31	1.4	0.01	1.7	0.01	1.5
A_52_P543824	NM_026499	Sfrs6	0.96	1.0	0.15	1.2	0.00	1.5
A_55_P2059110	NM_001033186	Skap1	0.71	1.2	0.57	1.2	0.03	1.5
A_51_P236267	NM_009183	St8sia4	0.14	1.4	0.00	1.7	0.00	1.5
A_55_P2183597	NM_198664	Tbc1d2	0.75	1.2	0.44	1.2	0.00	1.5
A_51_P317695	NM_019510	Trpc3	0.85	1.1	0.00	1.4	0.00	1.5
A_55_P1966644	NM_013838	Trpc6	0.49	1.3	0.01	1.6	0.01	1.5
A_55_P2037962	NM_153417	Trpm6	0.92	1.1	0.17	1.3	0.00	1.5

A_51_P164939	NM_144916	Tmem150a	0.67	1.2	0.10	1.3	0.00	1.5
A_51_P489736	NM_001142647	Tmem194b	0.78	1.1	0.00	1.4	0.00	1.5
A_65_P10673	NM_172614	Tmem44	0.40	1.3	0.00	1.6	0.00	1.5
A_52_P112110	NM_145987	Tmem82	0.59	1.3	0.18	1.3	0.01	1.5
A_55_P1962359	NM_053168	Trim11	0.92	1.1	0.03	1.3	0.00	1.5
A_51_P104933	NM_145520	Trub2	0.68	1.1	0.00	1.4	0.00	1.5
A_52_P144297	NM_198617	Tspyl3	0.20	1.4	0.02	1.4	0.00	1.5
A_51_P201773	NM_198031	Tubgcp3	0.86	1.1	0.00	1.4	0.00	1.5
A_55_P2117614	NM_028075	Tnfrsf13c	0.49	1.3	0.00	1.9	0.01	1.5
A_55_P1963344	NM_026573	Upf3b	0.69	-1.2	0.07	1.3	0.00	1.5
A_52_P301495	NM_009497	Vamp2	0.94	1.0	0.04	1.4	0.00	1.5
A_51_P316801	NM_146039	Wdr60	0.78	1.2	0.01	1.6	0.03	1.5
A_55_P2004746	NM_183319	Xkrx	0.91	1.1	0.15	1.4	0.03	1.5
A_55_P1970017	NM_178694	Zer1	0.76	1.1	0.03	1.3	0.00	1.5
A_52_P139819	NM_178404	Zc3h6	0.28	1.2	0.00	1.4	0.00	1.5
A_51_P312997	NM_012017	Zfp346	0.73	1.1	0.00	1.4	0.00	1.5
A_51_P156263	NM_027201	Zfp511	0.71	1.1	0.00	1.4	0.00	1.5
A_52_P217604	NM_182996	Zfp692	0.87	-1.1	0.44	1.2	0.02	1.5
A_55_P2139889	NR_027963	Zfp783	0.70	1.2	0.06	1.3	0.00	1.5
A_51_P396917	NM_026752	Zfyve21	0.76	1.2	0.36	1.2	0.00	1.5
A_51_P392967	NM_053253	Zmynd10	0.95	-1.1	0.37	1.3	0.00	1.5
A_30_P01031072			0.91	-1.1	0.01	1.5	0.00	1.4
A_30_P01018252			0.46	1.3	0.01	1.5	0.02	1.4
A_30_P01020325			0.69	-1.1	0.00	1.5	0.00	1.4
A_51_P298802	NM_001002896	Bfsp2	0.66	1.2	0.00	1.6	0.01	1.4
A_66_P114768	NM_001109661	Bach2	0.75	1.1	0.00	1.5	0.00	1.4
A_51_P181286	NM_001033122	Cd69	0.41	1.3	0.01	1.5	0.00	1.4
A_51_P124345	NM_153166	Cpne5	0.61	1.2	0.00	1.6	0.00	1.4
A_55_P1999301	NM_021282	Cyp2e1	0.97	1.1	0.03	2.1	0.34	1.4
A_55_P1966690	NM_021282	Cyp2e1	1.00	-1.0	0.04	1.9	0.34	1.4
A_51_P185247	NM_145741	Gdf10	0.97	1.0	0.01	1.7	0.04	1.4
A_52_P1391	NM_010388	H2-DMb2	0.35	1.3	0.02	1.5	0.03	1.4
A_51_P196695	NM_008372	Il7r	0.00	1.6	0.49	1.2	0.02	1.4
A_51_P121031	NM_175188	40969.0	0.00	1.5	0.11	1.3	0.03	1.4
A_51_P232281	NM_011109	Pla2g2d	0.81	1.1	0.00	1.5	0.00	1.4
A_55_P2089233	NM_011136	Pou2af1	0.61	1.3	0.03	1.5	0.05	1.4
A_55_P1976278	NM_008855	Prkcb	0.09	1.6	0.04	1.5	0.08	1.4
A_55_P2095899	XM_001476219	2310057B04Rik	0.59	1.2	0.00	1.5	0.00	1.4
A_66_P114333	NM_205823	Tlr12	0.08	1.7	0.01	1.8	0.08	1.4
A_55_P2125491	NM_011659	Tnfrsf4	0.85	1.1	0.03	1.5	0.02	1.4
A_51_P399853	NM_133218	Zfp704	0.75	1.1	0.00	1.5	0.00	1.4
A_30_P01020777			0.53	1.2	0.00	1.6	0.04	1.3
A_30_P01020208			0.28	1.4	0.03	1.5	0.04	1.3
A_55_P1981964	AK004668		0.33	1.3	0.01	1.5	0.04	1.3
A_55_P2108943	NM_009835	Ccr6	0.35	1.3	0.00	1.7	0.04	1.3
A_51_P146753	NM_007781	Csf2rb2	0.00	1.7	0.97	-1.0	0.11	1.3
A_51_P258372	NM_030691	Igsf6	0.00	2.1	0.72	1.2	0.24	1.3
A_51_P231320	NM_008611	Mmp8	0.00	2.5	0.70	1.2	0.48	1.3
A_52_P483336	NM_007641	Ms4a1	0.58	1.3	0.00	1.8	0.26	1.3

A_55_P2185900	NM_032002	Nrg4	0.94	1.1	0.03	1.6	0.22	1.3
A_55_P1957249	NM_001146268	Pdgfrb	0.84	1.1	0.00	1.5	0.04	1.3
A_55_P2142232	XM_893705	Gm6930	0.00	1.5	0.99	1.0	0.05	1.3
A_65_P20249	NM_008855	Prkcb	0.00	1.6	0.09	1.4	0.17	1.3
A_51_P114456	NM_025617	2210012G02Rik	0.64	1.3	0.04	1.6	0.33	1.3
A_51_P408649	NM_145463	Shisa2	0.89	1.1	0.04	1.6	0.23	1.3
A_51_P305052	NM_172900	Siglecg	0.52	1.2	0.00	1.5	0.02	1.3
A_55_P2091193	XM_001480396	LOC100048513	0.00	1.5	0.11	1.3	0.04	1.3
A_55_P1963960	XM_918548	LOC641201	0.00	2.1	0.69	1.2	0.41	1.3
A_51_P495581	NM_030682	Tlr1	0.00	1.5	0.00	1.4	0.01	1.3
A_55_P2011387	NM_145133	Tifa	0.00	1.6	0.59	1.2	0.15	1.3
A_66_P120987	NM_007655	Cd79a	0.77	1.2	0.03	1.5	0.41	1.2
A_55_P2054445	NM_013489	Cd84	0.04	1.8	0.80	1.1	0.47	1.2
A_55_P2037812	NM_023245	Palmd	0.80	1.1	0.02	1.5	0.17	1.2
A_51_P366672	NM_153170	Slc36a2	0.71	1.2	0.00	1.7	0.23	1.2
A_52_P392644	NM_173769	Zfp641	0.87	1.1	0.02	1.5	0.37	1.2
A_55_P2066778	NM_206536	AB124611	0.00	1.5	0.49	1.2	0.80	1.1
A_55_P2137049	NM_001004174	AA467197	0.04	2.8	0.98	1.0	0.98	1.0
A_66_P114784	NM_013737	Pla2g7	0.04	1.9	0.84	-1.1	0.92	1.0
A_55_P2040485	XM_889011	Ms4a4a	0.00	2.2	0.80	1.1	0.88	-1.1
A_51_P284823	NM_009086	Polr1b	0.33	-1.5	0.01	-1.7	0.76	-1.1
A_55_P2143765	NM_145079	Ugt1a6a	0.74	-1.1	0.00	-1.5	0.26	-1.2
A_51_P423709	NM_029007	Fam84a	1.00	1.0	0.04	-1.5	0.11	-1.3
A_52_P525317	NM_008121	Gja5	0.61	-1.3	0.03	-1.5	0.17	-1.3
A_51_P464387	NM_030704	Hspb8	0.29	-1.3	0.00	-1.5	0.00	-1.3
A_52_P318673	NM_009117	Saa1	0.04	6.5	0.92	-1.2	0.84	-1.3
A_55_P1953169	NM_011315	Saa3	0.00	12.2	0.88	-1.4	0.86	-1.3
A_55_P2165790	NM_178706	Siglech	1.00	1.0	0.00	-1.5	0.01	-1.3
A_30_P01017790			0.38	-1.3	0.02	-1.5	0.01	-1.4
A_66_P100365			0.04	-1.3	0.00	-1.5	0.00	-1.4
A_55_P2019719	NM_145227	Oas2	0.77	-1.3	0.03	-2.0	0.34	-1.4
A_66_P124858	NM_001024474	Diras2	0.16	-1.5	0.03	-1.5	0.04	-1.4
A_55_P2085546	NM_021422	Dnaja4	0.00	-1.5	0.01	-1.5	0.00	-1.4
A_51_P507290	NM_009769	Klf5	0.06	-1.4	0.00	-1.5	0.01	-1.4
A_55_P2022569	NM_008778	Pak3	0.88	-1.1	0.00	-1.5	0.00	-1.4
A_30_P01025874			0.77	-1.2	0.01	-1.6	0.01	-1.5
A_30_P01024706			0.97	1.0	0.26	-1.4	0.03	-1.5
A_30_P01029545			0.89	1.1	0.33	-1.3	0.03	-1.5
A_30_P01032527			0.93	-1.1	0.06	-1.5	0.03	-1.5
A_55_P2069530			0.95	-1.0	0.53	-1.2	0.02	-1.5
A_30_P01024724			0.66	-1.2	0.13	-1.4	0.02	-1.5
A_30_P01031754			0.83	-1.1	0.38	-1.3	0.02	-1.5
A_30_P01019049			0.73	-1.2	0.14	-1.4	0.01	-1.5
A_30_P01020907			0.66	-1.2	0.33	-1.2	0.01	-1.5
A_30_P01020742			0.91	-1.1	0.20	-1.3	0.01	-1.5
A_30_P01028808			0.88	-1.1	0.38	-1.2	0.01	-1.5
A_30_P01021970			0.85	-1.1	0.20	-1.3	0.00	-1.5
A_30_P01032942			0.71	-1.2	0.34	-1.2	0.00	-1.5
A_30_P01025051			0.85	-1.1	0.08	-1.3	0.00	-1.5

A_30_P01021932			0.54	-1.2	0.03	-1.4	0.00	-1.5
A_30_P01029711			0.16	-1.2	0.25	-1.1	0.00	-1.5
A_30_P01024297			0.77	-1.1	0.29	-1.2	0.00	-1.5
A_55_P1955733	XM_001477303		0.83	-1.1	0.27	-1.2	0.00	-1.5
A_30_P01031683			0.85	-1.1	0.21	-1.2	0.00	-1.5
A_55_P1981002			0.63	-1.1	0.01	-1.3	0.00	-1.5
A_55_P2021923	AB241120		0.76	-1.1	0.06	-1.3	0.00	-1.5
A_30_P01022343			0.83	-1.1	0.17	-1.3	0.00	-1.5
A_30_P01026230			0.78	-1.1	0.47	-1.2	0.00	-1.5
A_30_P01023400			0.40	-1.3	0.00	-1.4	0.00	-1.5
A_30_P01029815			0.75	-1.2	0.15	-1.3	0.00	-1.5
A_30_P01028277			0.92	-1.1	0.35	-1.2	0.00	-1.5
A_30_P01028705			0.76	-1.1	0.50	-1.2	0.00	-1.5
A_30_P01028258			0.84	-1.1	0.10	-1.4	0.00	-1.5
A_30_P01027919			0.93	-1.1	0.08	-1.4	0.00	-1.5
A_30_P01033110			0.74	-1.2	0.19	-1.3	0.00	-1.5
A_30_P01024606			0.54	-1.2	0.76	-1.1	0.00	-1.5
A_55_P1962707			0.84	-1.1	0.20	-1.3	0.00	-1.5
A_30_P01032304			0.64	-1.2	0.00	-1.4	0.00	-1.5
A_30_P01022751			0.77	-1.1	0.19	-1.2	0.00	-1.5
A_30_P01032647			0.53	-1.2	0.02	-1.4	0.00	-1.5
A_30_P01025204			0.66	-1.1	0.05	-1.3	0.00	-1.5
A_51_P265571	NM_009627	Adm	0.82	-1.2	0.50	-1.2	0.03	-1.5
A_51_P234544	NM_018745	Azin1	0.64	-1.3	0.19	-1.4	0.03	-1.5
A_55_P2043367	NM_173786	Apol9a	0.86	-1.1	0.03	-1.6	0.02	-1.5
A_55_P2000369	NM_009700	Aqp4	0.38	-1.4	0.30	-1.3	0.03	-1.5
A_55_P2003824	NM_009714	Asgr1	0.15	-1.7	0.18	-1.5	0.04	-1.5
A_55_P2003823	NM_009714	Asgr1	0.25	-1.4	0.10	-1.4	0.01	-1.5
A_52_P168097	NM_009722	Atp2a2	0.59	-1.2	0.38	-1.2	0.01	-1.5
A_51_P371174	NM_013863	Bag3	0.00	-1.3	0.00	-1.5	0.00	-1.5
A_52_P278354	NM_007557	Bmp7	0.80	-1.1	0.49	-1.2	0.01	-1.5
A_55_P1990879	NM_016812	Banp	0.15	-1.3	0.00	-1.4	0.00	-1.5
A_51_P175424	NM_011797	Car14	0.43	-1.4	0.17	-1.4	0.02	-1.5
A_51_P517120	NM_001013379	D10627	0.76	-1.1	0.23	-1.2	0.00	-1.5
A_52_P329367	NM_001025566	Chka	0.55	-1.2	0.05	-1.3	0.00	-1.5
A_55_P2175469	NM_001081345	Chd2	0.83	-1.2	0.78	-1.1	0.04	-1.5
A_51_P446477	NM_171826	Cldnd1	0.61	-1.2	0.38	-1.2	0.00	-1.5
A_52_P590474	NM_019937	Ccnl1	0.63	-1.2	0.04	-1.3	0.00	-1.5
A_51_P208603	NM_010050	Dio2	0.86	-1.1	0.58	-1.2	0.02	-1.5
A_55_P2112967	NM_007858	Diap1	0.38	-1.3	0.04	-1.4	0.00	-1.5
A_55_P2014978	NM_007889	Dvl3	0.82	-1.1	0.22	-1.2	0.00	-1.5
A_52_P52263	NM_001044719	D17Wsu92e	0.89	-1.1	0.13	-1.4	0.01	-1.5
A_55_P2334484	BE650233	D9Ertd115e	0.17	-1.3	0.00	-1.5	0.00	-1.5
A_51_P439857	NM_030046	Dnajc21	0.14	-1.3	0.05	-1.2	0.00	-1.5
A_52_P445253	NM_133833	Dst	0.80	-1.1	0.19	-1.3	0.01	-1.5
A_52_P654965	NM_144545	Eif3j	0.73	-1.2	0.45	-1.2	0.00	-1.5
A_52_P265544	NM_177077	Exoc6b	0.40	-1.2	0.05	-1.2	0.00	-1.5
A_55_P2231747	BB325257	AW742560	0.94	-1.1	0.30	-1.3	0.03	-1.5
A_51_P371485	NM_198017	Fam175b	0.56	-1.2	0.07	-1.3	0.00	-1.5

A_55_P2076984	NM_134082	Farp1	0.74	-1.2	0.34	-1.2	0.00	-1.5
A_55_P2039196	NM_020014	Gfra4	0.72	-1.2	0.10	-1.3	0.00	-1.5
A_55_P2059532	NM_027389	Gon4l	0.72	-1.2	0.34	-1.2	0.00	-1.5
A_55_P1955457	NM_010345	Grb10	0.94	-1.1	0.28	-1.3	0.01	-1.5
A_65_P15809	NR_003967	Gm4759	0.98	-1.0	0.30	-1.2	0.00	-1.5
A_55_P1955587	NM_010477	Hspd1	0.44	-1.3	0.55	-1.2	0.01	-1.5
A_52_P218537	NM_016884	Hnrnpc	0.89	-1.1	0.07	-1.3	0.00	-1.5
A_55_P2021089	XM_001472541	Ighg	0.40	-1.4	0.04	-1.6	0.03	-1.5
A_52_P212686	NM_146191	Lrrk1	0.84	-1.1	0.09	-1.3	0.00	-1.5
A_55_P2010097	NM_001045550	Mup2	0.61	-1.3	0.13	-1.5	0.03	-1.5
A_51_P150521	NM_146035	Mgat2	0.87	-1.1	0.01	-1.4	0.00	-1.5
A_55_P2039250	NM_026037	Mboat2	0.80	-1.1	0.46	-1.2	0.01	-1.5
A_51_P418168	NM_029103	Manf	0.97	-1.0	0.19	-1.3	0.01	-1.5
A_55_P2095047	NM_145569	Mat2a	0.38	-1.3	0.00	-1.5	0.00	-1.5
A_55_P2078710	AK160137	Morc4	0.42	-1.3	0.07	-1.3	0.00	-1.5
A_52_P58257	NM_080456	Mrps6	0.61	-1.2	0.13	-1.3	0.00	-1.5
A_55_P2069926	NM_009268	Mucl1	0.04	-1.8	0.03	-1.6	0.04	-1.5
A_55_P2077866	NM_010918	Nktr	0.50	-1.3	0.51	-1.2	0.01	-1.5
A_55_P2104917	NM_016902	Nphp1	0.44	-1.4	0.71	-1.2	0.03	-1.5
A_55_P2068772	NM_026554	Ncbp2	0.55	-1.2	0.03	-1.3	0.00	-1.5
A_51_P151433	NM_026554	Ncbp2	0.93	-1.1	0.28	-1.3	0.00	-1.5
A_52_P565940	NM_008739	Nsd1	0.73	-1.1	0.22	-1.2	0.00	-1.5
A_52_P572197	NM_145962	Pank3	0.84	-1.1	0.11	-1.3	0.00	-1.5
A_55_P2056236	NM_019809	Pdlim5	0.54	-1.3	0.22	-1.4	0.03	-1.5
A_65_P20174	NM_172303	Phf17	0.55	-1.2	0.18	-1.2	0.00	-1.5
A_55_P2126627	NM_001081409	Phf20l1	0.60	-1.2	0.26	-1.3	0.01	-1.5
A_55_P2133624	XM_001475193	Gm2891	0.87	-1.1	0.25	-1.2	0.00	-1.5
A_55_P1960738	XM_001472780	Gm4470	0.69	-1.2	0.50	-1.2	0.01	-1.5
A_55_P2055763	NM_001034902	Gm5878	0.88	-1.1	0.16	-1.3	0.00	-1.5
A_55_P1963645	XM_897102	Gm6323	0.81	-1.2	0.42	-1.2	0.01	-1.5
A_55_P1967002	XM_001473953	Gm9441	0.65	-1.2	0.29	-1.3	0.00	-1.5
A_55_P2031989	NM_028724	Rin2	0.84	-1.1	0.76	-1.1	0.00	-1.5
A_52_P565575	NM_175535	Arhgap20	0.72	-1.2	0.33	-1.3	0.01	-1.5
A_55_P2363902	AK011494	2610020P09Rik	0.91	-1.1	0.05	-1.4	0.00	-1.5
A_55_P2022724	NR_030716	5430417L22Rik	0.65	-1.2	0.03	-1.3	0.00	-1.5
A_55_P2278965	AK017326	5430420F09Rik	0.97	-1.0	0.15	-1.2	0.00	-1.5
A_55_P2193064	AK142114	6720473M08Rik	0.88	-1.1	0.36	-1.3	0.02	-1.5
A_52_P393589	NM_133733	9030425E11Rik	0.96	1.0	0.44	-1.2	0.02	-1.5
A_55_P2067608	NM_133733	9030425E11Rik	1.00	-1.0	0.37	-1.2	0.00	-1.5
A_55_P2189893	AK042233	A630073K07Rik	0.91	-1.1	0.10	-1.3	0.00	-1.5
A_55_P2037618	NM_001163502	C130039O16Rik	0.85	-1.1	0.61	-1.1	0.00	-1.5
A_55_P2329313	AK049648	C530014P21Rik	0.98	-1.0	0.32	-1.3	0.03	-1.5
A_55_P2446231	AK042002	D730040F13Rik	0.91	-1.1	0.55	-1.2	0.03	-1.5
A_55_P2233373	AK032962	D930023I05Rik	0.77	-1.2	0.61	-1.2	0.01	-1.5
A_51_P503192	NM_175402	Rbm15b	0.69	-1.2	0.03	-1.4	0.00	-1.5
A_52_P280360	NM_016809	Rbm3	0.76	-1.2	0.08	-1.4	0.00	-1.5
A_55_P1993858	NR_001460	Rmrp	0.92	-1.1	0.23	-1.3	0.01	-1.5
A_51_P161354	NM_144907	Sesn2	0.85	-1.1	0.14	-1.3	0.00	-1.5
A_55_P2021011	NM_030207	Sfi1	0.34	-1.3	0.03	-1.3	0.00	-1.5

A_52_P273169	NM_001082414	Sh3d19	0.65	-1.2	0.28	-1.3	0.01	-1.5
A_51_P466886	NM_019708	Scoc	0.53	-1.2	0.24	-1.2	0.00	-1.5
A_55_P2183498	NM_025668	Spcs2	0.91	-1.1	0.20	-1.2	0.00	-1.5
A_55_P2151082	XM_919699	LOC632154	0.82	-1.2	0.59	-1.2	0.01	-1.5
A_66_P131645	XM_001477593	LOC100047183	0.92	-1.1	0.05	-1.5	0.01	-1.5
A_55_P2048660	XM_908118	LOC633654	0.85	-1.1	0.20	-1.3	0.00	-1.5
A_55_P2124619	NM_025481	Smurf2	0.32	-1.2	0.08	-1.3	0.00	-1.5
A_55_P2351193	AF357397	Snora47	0.77	-1.1	0.01	-1.4	0.00	-1.5
A_55_P2079388	NR_002905	Snora74a	0.99	-1.0	0.05	-1.3	0.00	-1.5
A_55_P2058117	NM_177732	Slc35d1	0.55	-1.3	0.48	-1.2	0.03	-1.5
A_52_P532227	NM_007901	S1pr1	0.85	-1.2	0.16	-1.4	0.04	-1.5
A_66_P134690	NM_146043	Spin1	0.23	-1.2	0.00	-1.3	0.00	-1.5
A_52_P291971	NM_025303	Stau2	0.64	-1.1	0.04	-1.3	0.00	-1.5
A_51_P142744	NM_172294	Sulf1	0.87	1.1	0.26	-1.4	0.03	-1.5
A_52_P393306	NM_009222	Snap23	0.83	-1.1	0.03	-1.4	0.00	-1.5
A_55_P2462940	NM_001081008	Taf1	0.70	-1.2	0.70	-1.1	0.00	-1.5
A_55_P2081388	NM_008575	Mdm4	0.75	-1.2	0.93	-1.0	0.01	-1.5
A_51_P490023	NM_009450	Tubb2a	0.85	-1.1	0.01	-1.3	0.00	-1.5
A_52_P434974	NM_152234	Ubqln1	0.62	-1.1	0.00	-1.4	0.00	-1.5
A_51_P180452	NM_133656	Crk	0.94	-1.1	0.26	-1.3	0.01	-1.5
A_66_P100937	XM_001487796	Zfp33b	0.95	-1.1	0.72	-1.1	0.01	-1.5
A_55_P2173373	NM_001024846	Zfp62	0.71	-1.2	0.65	-1.1	0.00	-1.5
A_55_P2039038	NM_001081750	Zfp664	0.45	-1.2	0.25	-1.2	0.00	-1.5
A_30_P01031627			0.00	-1.5	0.00	-1.5	0.00	-1.6
A_30_P01019583			0.64	-1.3	0.41	-1.3	0.01	-1.6
A_55_P2109564	AK039146		0.90	1.1	0.77	-1.1	0.00	-1.6
A_30_P01030704			0.54	-1.3	0.13	-1.3	0.00	-1.6
A_30_P01025354			0.68	-1.2	0.20	-1.3	0.00	-1.6
A_30_P01018220			0.86	-1.1	0.13	-1.4	0.00	-1.6
A_55_P1991239	AK051413		0.69	-1.1	0.01	-1.3	0.00	-1.6
A_55_P1998721	AK137722		0.60	-1.2	0.11	-1.3	0.00	-1.6
A_52_P245766			0.51	-1.3	0.26	-1.3	0.00	-1.6
A_30_P01029345			0.82	-1.1	0.04	-1.4	0.00	-1.6
A_55_P2072315	AK133410		0.78	-1.1	0.14	-1.3	0.00	-1.6
A_30_P01017707			0.84	-1.1	0.05	-1.4	0.00	-1.6
A_30_P01027949			0.55	-1.2	0.04	-1.3	0.00	-1.6
A_30_P01026028			0.52	-1.3	0.27	-1.3	0.00	-1.6
A_55_P2090205	U04541		0.23	-1.3	0.01	-1.4	0.00	-1.6
A_55_P2114994			0.94	1.0	0.11	-1.2	0.00	-1.6
A_30_P01032670			0.45	-1.3	0.06	-1.4	0.00	-1.6
A_30_P01025230			0.76	-1.2	0.06	-1.4	0.00	-1.6
A_30_P01026318			1.00	-1.0	0.08	-1.4	0.00	-1.6
A_51_P155763	NM_001146089	Ascc3	0.70	-1.1	0.00	-1.5	0.00	-1.6
A_55_P1977875	NM_176849	Arglu1	0.14	-1.3	0.10	-1.3	0.00	-1.6
A_52_P97670	NM_001099628	Atad2b	0.72	-1.2	0.25	-1.2	0.00	-1.6
A_66_P101519	NM_021041	Abcc9	0.12	-1.6	0.56	-1.2	0.01	-1.6
A_55_P1985259	NM_001017985	C2cd3	0.69	-1.2	0.17	-1.3	0.01	-1.6
A_52_P334670	NM_007715	Clock	0.48	-1.3	0.17	-1.3	0.00	-1.6
A_55_P2143837	NM_007735	Col4a4	0.14	-1.4	0.08	-1.3	0.00	-1.6

A_51_P302167	NM_172496	Cobl	0.07	-1.5	0.01	-1.5	0.00	-1.6
A_51_P363947	NM_007669	Cdkn1a	0.66	-1.3	0.01	-1.6	0.00	-1.6
A_55_P1959973	NM_175539	Dcaf12l2	0.69	-1.2	0.17	-1.3	0.00	-1.6
A_55_P2017845	NM_153078	Ehbp1	0.80	-1.1	0.20	-1.3	0.00	-1.6
A_66_P105564	NM_175452	Gjc2	0.49	-1.2	0.00	-1.4	0.00	-1.6
A_52_P554703	NM_183183	Gprin3	0.66	-1.3	0.01	-1.6	0.01	-1.6
A_52_P281702	NM_010518	Igfbp5	0.81	-1.2	0.11	-1.4	0.01	-1.6
A_52_P351816	NM_010578	Itgb1	0.87	-1.1	0.21	-1.3	0.01	-1.6
A_55_P2408415	NR_001461	Kcnq1ot1	0.32	-1.3	0.07	-1.4	0.00	-1.6
A_55_P2151209	NM_130873	Krtap16-4	0.69	-1.1	0.12	-1.2	0.00	-1.6
A_52_P52128	NM_001039522	Leo1	1.00	-1.0	0.44	-1.3	0.01	-1.6
A_55_P2062642	NM_025952	Magt1	0.73	-1.1	0.00	-1.4	0.00	-1.6
A_55_P1988970	NM_021527	Mkks	0.32	-1.3	0.01	-1.4	0.00	-1.6
A_55_P2111172	NM_021442	Mecom	0.81	-1.1	0.07	-1.4	0.00	-1.6
A_55_P2336173	AK077315	Mirg	1.00	1.0	0.22	-1.5	0.03	-1.6
A_51_P108978	NM_016969	Myadm	0.86	-1.1	0.17	-1.4	0.01	-1.6
A_55_P1987409	NM_173444	Nbeal1	0.58	-1.3	0.07	-1.4	0.01	-1.6
A_52_P634822	NM_008672	Nap1l4	0.73	-1.2	0.26	-1.2	0.00	-1.6
A_55_P1993054	NM_133948	Psip1	0.48	-1.2	0.08	-1.3	0.00	-1.6
A_55_P2052623	NM_145583	Pgap2	0.91	-1.1	0.69	-1.2	0.04	-1.6
A_55_P1999818	XM_001472026	Gm2006	0.77	-1.1	0.13	-1.3	0.00	-1.6
A_52_P239023	NM_001142957	Gm4455	0.66	-1.2	0.14	-1.2	0.00	-1.6
A_55_P2067741	NM_020261	Psg23	0.85	-1.1	0.88	-1.1	0.00	-1.6
A_55_P2413598	NM_001080813	Rab11fip1	0.53	-1.2	0.19	-1.3	0.00	-1.6
A_51_P477364	NM_007483	Rhob	0.71	-1.2	0.21	-1.3	0.00	-1.6
A_52_P527874	NM_199447	Rrp12	0.58	-1.2	0.00	-1.3	0.00	-1.6
A_55_P2281624	AK142273	2610034E01Rik	0.61	-1.2	0.15	-1.3	0.00	-1.6
A_52_P40954	NR_028300	5330426P16Rik	0.91	-1.1	0.04	-1.4	0.00	-1.6
A_51_P474169	NM_144883	5430407P10Rik	0.00	-1.4	0.00	-1.4	0.00	-1.6
A_55_P1974522	NM_175688	A530099J19Rik	0.06	-1.4	0.00	-1.4	0.00	-1.6
A_55_P2292046	AK084291	D230018H15Rik	0.26	-1.4	0.00	-1.7	0.00	-1.6
A_52_P181468	NM_030241	Setd8	0.31	-1.3	0.12	-1.3	0.00	-1.6
A_55_P2104798	XM_001480269	LOC100044040	0.52	-1.3	0.18	-1.3	0.00	-1.6
A_55_P2018307	XM_001005025	LOC677576	0.76	-1.1	0.20	-1.2	0.00	-1.6
A_55_P2072980	NM_001076554	Spna2	0.75	-1.2	0.43	-1.3	0.01	-1.6
A_51_P485458	NM_001005506	Txlna	0.38	-1.3	0.12	-1.3	0.00	-1.6
A_55_P1989865	NM_146153	Thrap3	0.83	-1.2	0.81	-1.1	0.01	-1.6
A_55_P2433438	NM_011544	Tcf12	0.86	-1.1	0.11	-1.3	0.00	-1.6
A_52_P45738	NM_029979	Trim35	0.80	-1.2	0.64	-1.2	0.04	-1.6
A_55_P2108754	NM_138589	Ubfd1	0.80	-1.1	0.13	-1.3	0.00	-1.6
A_55_P2006035	NM_030166	Galntl2	0.55	-1.4	0.67	-1.2	0.01	-1.6
A_52_P543040	NM_028276	Utp14a	0.63	-1.2	0.18	-1.2	0.00	-1.6
A_55_P2212027	NM_011682	Utrn	0.65	-1.2	0.22	-1.3	0.00	-1.6
A_55_P1964559	NR_002888	Vmn2r-ps14	0.78	-1.1	0.39	-1.2	0.00	-1.6
A_55_P1966833	NM_001037713	Xaf1	0.69	-1.3	0.11	-1.5	0.01	-1.6
A_30_P01028666			0.87	-1.1	0.02	-1.5	0.00	-1.7
A_30_P01025818			0.44	-1.4	0.02	-1.6	0.00	-1.7
A_30_P01029702			0.60	-1.2	0.00	-1.6	0.00	-1.7
A_30_P01030969			0.97	1.0	0.41	-1.3	0.01	-1.7

A_30_P01028353			0.34	-1.2	0.01	-1.4	0.00	-1.7
A_30_P01025536			0.65	-1.2	0.03	-1.3	0.00	-1.7
A_55_P2108903	AK038731		0.87	-1.1	0.09	-1.4	0.00	-1.7
A_66_P115161	AK039734		0.85	-1.1	0.04	-1.4	0.00	-1.7
A_30_P01029006			0.30	-1.5	0.06	-1.5	0.00	-1.7
A_30_P01021097			0.89	-1.1	0.20	-1.4	0.00	-1.7
A_55_P2105321	NM_023190	Acin1	0.72	-1.2	0.64	-1.2	0.00	-1.7
A_51_P300618	NM_001163566	Crb2	0.91	1.1	0.45	-1.4	0.02	-1.7
A_55_P2430472	NM_011805	Dido1	0.68	-1.2	0.62	-1.2	0.00	-1.7
A_55_P1992149	NM_001164671	Dnaja1	0.33	-1.3	0.00	-1.7	0.00	-1.7
A_51_P383270	NM_175473	Fras1	0.96	1.1	0.27	-1.4	0.00	-1.7
A_55_P2060284	NM_001033380	Itpripl2	0.66	-1.2	0.12	-1.3	0.00	-1.7
A_52_P176737	NM_178142	Lcorl	0.76	-1.1	0.03	-1.3	0.00	-1.7
A_55_P2454099	NM_023799	Mgea5	0.85	-1.1	0.41	-1.2	0.00	-1.7
A_55_P2175752	NM_001081975	Mfap1b	0.88	-1.1	0.66	-1.2	0.00	-1.7
A_55_P2115127	NM_026483	Mphosph10	0.72	-1.1	0.18	-1.2	0.00	-1.7
A_55_P2169356	XM_001000891	Gm1966	0.93	-1.1	0.86	-1.1	0.04	-1.7
A_52_P332788	XM_001473420	Gm2397	0.90	1.1	0.91	-1.1	0.00	-1.7
A_55_P2126557	XM_619973	Gm5858	0.72	-1.2	0.13	-1.3	0.00	-1.7
A_52_P30451	NM_016854	Ppp1r3c	0.45	-1.4	0.39	-1.3	0.00	-1.7
A_52_P667913	NM_033592	Pcdhga9	0.79	-1.2	0.72	-1.2	0.04	-1.7
A_51_P227392	NM_133955	Rhou	0.94	1.1	0.35	-1.4	0.03	-1.7
A_51_P112762	NM_017391	Slc5a3	0.07	-1.4	0.00	-1.5	0.00	-1.7
A_52_P283055	NM_017391	Slc5a3	0.46	-1.3	0.01	-1.4	0.00	-1.7
A_55_P2170514	NM_018754	Sfn	0.36	-1.7	0.11	-1.7	0.04	-1.7
A_51_P210143	NM_001005510	Syne2	0.88	-1.1	0.31	-1.3	0.00	-1.7
A_55_P2413458	NM_023755	Tcfcp2l1	0.90	-1.1	0.60	-1.2	0.01	-1.7
A_30_P01031080			0.61	-1.2	0.02	-1.5	0.00	-1.8
A_30_P01022661			0.76	-1.2	0.01	-1.5	0.00	-1.8
A_30_P01019037			0.44	-1.4	0.04	-1.6	0.00	-1.8
A_30_P01031450			0.94	-1.0	0.00	-1.4	0.00	-1.8
A_52_P625171	NM_133723	Asph	0.32	-1.4	0.17	-1.4	0.00	-1.8
A_51_P153486	NM_018808	Dnajb1	0.00	-1.5	0.00	-1.9	0.00	-1.8
A_55_P2240823	NM_001128606	Epb4.1	0.75	-1.2	0.37	-1.3	0.00	-1.8
A_55_P2018176	AK039146	AI504432	0.91	-1.1	0.25	-1.3	0.00	-1.8
A_51_P267544	NM_013522	Frg1	0.77	-1.1	0.12	-1.3	0.00	-1.8
A_51_P157083	NM_008086	Gas1	0.97	-1.0	0.29	-1.4	0.01	-1.8
A_51_P327751	NM_008331	Ifit1	0.72	-1.3	0.04	-1.8	0.02	-1.8
A_66_P139460	NM_177389	Mia3	0.75	-1.2	0.22	-1.3	0.00	-1.8
A_55_P2061645	AK140300	ND6	0.76	-1.1	0.02	-1.4	0.00	-1.8
A_55_P1981455	XM_001472240	LOC100044430	1.00	-1.0	0.09	-1.6	0.00	-1.8
A_52_P355169	NM_011607	Tnc	0.82	-1.2	0.23	-1.4	0.00	-1.8
A_55_P2130219	NM_133780	Tpr	0.88	-1.1	0.31	-1.2	0.00	-1.8
A_55_P1968304			0.15	-1.3	0.00	-1.8	0.00	-1.9
A_30_P01032544			0.51	-1.7	0.27	-1.6	0.04	-1.9
A_55_P2160416	NM_028765	Acoxl	0.94	-1.1	0.97	-1.0	0.04	-1.9
A_55_P2073935	NM_013790	Abcc5	0.78	-1.2	0.03	-1.4	0.00	-1.9
A_51_P129803	NM_009786	Cacybp	0.31	-1.3	0.00	-1.7	0.00	-1.9
A_52_P650387	NM_001045530	Ccnjl	0.72	-1.2	0.06	-1.4	0.00	-1.9

A_52_P493620	NM_026218	Fgfr1op2	0.91	-1.1	0.54	-1.3	0.01	-1.9
A_55_P2080603	NM_031165	Hspa8	0.08	-1.4	0.00	-1.7	0.00	-1.9
A_51_P421876	NM_016850	Irf7	0.97	-1.1	0.10	-1.9	0.03	-1.9
A_55_P2076196	NM_001134644	CU041261.1	0.46	-1.7	0.17	-1.8	0.04	-1.9
A_51_P284665	U85712	Plcb1	0.92	1.1	0.66	-1.2	0.01	-1.9
A_51_P114693	NM_145562	Parm1	0.59	-1.4	0.26	-1.4	0.00	-1.9
A_52_P671812	XM_918536	LOC641192	0.12	-1.3	0.00	-1.8	0.00	-1.9
A_30_P01031385			0.75	-1.3	0.03	-1.9	0.00	-2.0
A_51_P305843	NM_025844	Chordc1	0.00	-1.6	0.00	-1.8	0.00	-2.0
A_55_P2069721	NM_031165	Hspa8	0.14	-1.4	0.00	-1.8	0.00	-2.0
A_55_P2001494	NM_013598	Kitl	0.58	-1.3	0.15	-1.3	0.00	-2.0
A_55_P2035320	NM_017373	Nfil3	0.89	-1.1	0.00	-1.9	0.00	-2.0
A_55_P2408588	NM_007489	Arntl	0.79	-1.3	0.16	-1.7	0.01	-2.1
A_51_P497985	NM_013484	C2	0.93	1.1	0.22	-1.6	0.00	-2.1
A_52_P12877	NM_031165	Hspa8	0.29	-1.4	0.00	-2.1	0.00	-2.1
A_55_P1974080	NM_001135127	LOC100189605	0.57	-1.7	0.05	-2.2	0.03	-2.1
A_55_P1979904	NM_001126319	Mup9	0.52	-1.8	0.17	-1.9	0.03	-2.1
A_55_P2141479	NR_004414	Rnu2	0.81	-1.2	0.03	-1.7	0.00	-2.1
A_55_P1962918	NM_001033450	Mnda	0.92	1.2	0.30	-1.7	0.03	-2.2
A_51_P520849	NM_009144	Sfrp2	0.77	1.4	0.28	-1.7	0.02	-2.2
A_52_P381484	NM_133903	Spon2	0.91	-1.2	0.75	-1.2	0.01	-2.2
A_55_P1953377			0.38	-2.1	0.07	-2.4	0.04	-2.3
A_55_P2153620	NM_001039959	Ahnak	0.67	-1.3	0.31	-1.3	0.00	-2.3
A_55_P2017636	NM_011580	Thbs1	0.95	-1.1	0.02	-1.7	0.00	-2.3
A_30_P01019464			0.99	-1.0	0.72	-1.3	0.03	-2.4
A_30_P01026324			0.70	-1.3	0.99	-1.0	0.00	-2.4
A_51_P146560	NM_018857	Msln	0.91	1.2	0.22	-2.0	0.03	-2.4
A_55_P2176963	NM_013559	Hsph1	0.22	-1.8	0.02	-2.0	0.00	-2.5
A_55_P2024155	NM_001033324	Zbtb16	0.90	-1.2	0.07	-2.5	0.02	-2.5
A_55_P1987290	NR_004413	Rnu1b6	0.86	-1.2	0.00	-2.3	0.00	-2.6
A_55_P2087985			0.04	-1.6	0.00	-2.4	0.00	-2.8
A_55_P2003513	NM_013559	Hsph1	0.15	-1.9	0.01	-2.1	0.00	-2.9
A_55_P1993404			0.00	-1.7	0.00	-2.6	0.00	-3.0
A_55_P2135967	XM_979793	Gm7816	0.00	-1.7	0.00	-2.7	0.00	-3.1
A_55_P1953143	NM_144783	Wt1	0.68	1.8	0.25	-2.1	0.01	-3.1
A_52_P460836	NM_177303	Lrrn4	1.00	1.0	0.08	-2.3	0.00	-3.3
A_52_P114889	NM_175309	Upk3b	0.92	1.2	0.28	-2.1	0.01	-3.3
A_55_P2087984	NM_001164671	Dnaja1	0.04	-1.8	0.00	-2.9	0.00	-3.4
A_55_P2009217	NR_004415	Rnu3b1	0.14	-4.1	0.00	-9.0	0.00	-7.2
A_55_P2068459	NM_010479	Hspa1a	0.00	-2.9	0.00	-6.8	0.00	-7.9

3.3.2 BbF Lung. Significant probe list. List of all significantly differentially expressed probes in at least 1 treatment group (FDR P ≤ 0.05, fold change ± 1.5) in response to sub-chronic oral exposure to 25, 50, and 100 mg/kg-bw/day benzo(b)fluoranthene in the lung. The list is sorted from highest to lowest fold change in the 100 mg/kg-bw/day treatment group.

			**25 mg/kg-bw/day**		**50 mg/kg-bw/day**		**100 mg/kg-bw/day**	

Agilent Probe	Accession Number	Gene Symbol	*FDR P value*	*Fold change*	*FDR P value*	*Fold change*	*FDR P value*	*Fold change*
A_51_P279693	NM_009992	Cyp1a1	0.55	-2.1	0.99	1.0	0.00	19.1
A_51_P255456	NM_009994	Cyp1b1	0.69	1.5	0.35	1.8	0.00	17.5
A_55_P1960735	NM_011819	Gdf15	0.00	4.8	0.00	4.2	0.00	8.0
A_51_P254425	NM_009644	Ahrr	0.07	2.0	0.00	2.4	0.00	7.8
A_55_P2032081	NM_016974	Dbp	0.00	4.4	0.00	6.0	0.00	7.7
A_55_P2032079	NM_016974	Dbp	0.00	4.2	0.00	5.7	0.00	7.3
A_55_P1986282	NM_001111099	Cdkn1a	0.00	3.1	0.00	4.1	0.00	7.2
A_51_P414396	NM_153127	Mmrn2	0.00	2.2	0.00	3.0	0.00	6.2
A_55_P1959500	NM_172759	Ces5	0.00	4.4	0.00	4.6	0.00	5.9
A_51_P204740	NM_133654	Cd34	0.01	1.6	0.00	2.1	0.00	5.8
A_52_P675395	NM_007722	Cxcr7	0.02	2.0	0.00	2.3	0.00	5.8
A_51_P363947	NM_007669	Cdkn1a	0.00	2.6	0.00	2.7	0.00	5.6
A_55_P2143025	NM_013657	Sema3c	0.09	1.4	0.00	2.4	0.00	5.0
A_52_P24308	NM_011670	Uchl1	0.68	1.2	0.17	1.4	0.00	5.0
A_66_P118600	NM_008480	Lama1	0.02	2.2	0.00	2.5	0.00	4.8
A_55_P1954835	XM_001475752	LOC100046186	0.15	1.5	0.00	2.2	0.00	4.5
A_55_P2090429	NM_010063	Dync1i1	0.93	-1.1	0.58	1.2	0.00	4.4
A_55_P2101340	NM_019511	Ramp3	0.24	1.3	0.00	2.1	0.00	4.3
A_51_P329928	NM_013750	Phlda3	0.00	2.1	0.00	2.4	0.00	4.1
A_51_P516133	NM_015786	Hist1h1c	0.61	1.2	0.00	1.7	0.00	3.9
A_51_P231320	NM_008611	Mmp8	0.32	1.5	0.06	1.8	0.00	3.9
A_55_P2143572	AK020725		0.18	1.3	0.00	2.1	0.00	3.8
A_55_P2095271	NM_153805	Pkn3	0.00	2.0	0.00	2.2	0.00	3.8
A_55_P1953169	NM_011315	Saa3	0.85	-1.2	0.46	-1.6	0.00	3.6
A_55_P2107528			0.01	1.9	0.01	1.8	0.00	3.4
A_51_P405397	NM_007899	Ecm1	0.01	1.6	0.01	1.6	0.00	3.4
A_55_P2119257	NM_008871	Serpine1	0.04	1.5	0.00	1.8	0.00	3.4
A_55_P1972948	NM_176954	Brunol5	0.00	1.7	0.00	2.2	0.00	3.3
A_55_P1972034	XM_001476091	Muc16	0.85	1.3	0.99	1.0	0.01	3.3
A_52_P539310	NM_001160326	Serp2	0.00	1.7	0.00	1.9	0.00	3.3
A_55_P2005213	NM_145603	Ces2	0.00	2.7	0.00	2.4	0.00	3.2
A_55_P1988048	NM_001163522	Emcn	0.03	1.5	0.06	1.4	0.00	3.2
A_55_P2015292	NM_008521	Ltc4s	0.01	1.6	0.00	1.8	0.00	3.2
A_55_P2037812	NM_023245	Palmd	0.00	2.0	0.00	2.4	0.00	3.2
A_55_P2011146	NM_178608	Reep1	0.02	1.5	0.00	1.8	0.00	3.2
A_51_P487073	NM_138684	Wfdc12	0.01	2.7	0.10	1.7	0.00	3.2
A_52_P627068	NM_170593	Disp2	0.02	1.8	0.00	1.9	0.00	3.1
A_55_P2004099	XM_001477458	Gm9933	0.74	-1.3	0.62	-1.4	0.00	3.1
A_55_P2145804	NM_026531	Aen	0.00	2.0	0.00	2.1	0.00	2.9
A_55_P2023637	NM_021400	Prg4	0.85	1.2	0.89	-1.1	0.00	2.9
A_55_P2405784	AK141429	BC023202	0.01	1.4	0.00	1.6	0.00	2.8
A_55_P2009225	NM_133654	Cd34	0.30	1.2	0.01	1.5	0.00	2.8
A_30_P01024344			0.00	1.9	0.00	1.8	0.00	2.7
A_55_P2141860	NM_026531	Aen	0.00	1.9	0.00	1.9	0.00	2.7

A_52_P612803	NM_009831	Ccng1	0.00	1.8	0.00	2.0	0.00	2.7
A_51_P307168	NM_026993	Ddah1	0.01	1.6	0.00	2.8	0.00	2.7
A_51_P480328	NM_133222	Eltd1	0.25	1.5	0.03	1.7	0.00	2.7
A_55_P2027836	NM_020275	Tnfrsf10b	0.00	1.6	0.00	1.6	0.00	2.7
A_52_P151393	NM_198860	AI646023	0.00	1.6	0.00	1.8	0.00	2.6
A_55_P2137406	NM_007527	Bax	0.00	2.0	0.00	2.2	0.00	2.6
A_55_P2142251	NM_054045	Hist2h3c2	0.86	1.1	0.09	1.4	0.00	2.6
A_52_P380263	NM_013723	Podxl	0.75	1.1	0.05	1.4	0.00	2.6
A_52_P87839	NM_013657	Sema3c	0.80	1.1	0.03	1.5	0.00	2.6
A_66_P138053	AK044848		0.06	1.6	0.01	1.9	0.00	2.5
A_52_P29953	NM_175398	6530418L21Rik	0.00	1.7	0.00	1.7	0.00	2.5
A_55_P2040245	NM_001039485	Fam38b	0.15	1.3	0.09	1.3	0.00	2.5
A_51_P111962	NM_001141922	mCG_21548	0.58	1.2	0.51	1.2	0.00	2.5
A_51_P124535	NM_008590	Mest	0.28	1.3	0.13	1.4	0.00	2.5
A_55_P1963017	NM_001082543	Stfa1	0.44	1.5	0.42	1.4	0.00	2.5
A_51_P323620	NM_144543	Thyn1	0.00	1.9	0.00	2.0	0.00	2.5
A_55_P2031999	NM_145448	9030617O03Rik	0.01	1.6	0.00	1.8	0.00	2.4
A_55_P2162160	NM_009672	Anp32a	0.31	1.7	0.06	2.0	0.00	2.4
A_55_P1964648	NM_001037719	Btla	0.00	1.9	0.00	2.4	0.00	2.4
A_66_P111562	NM_007631	Ccnd1	0.14	1.4	0.00	1.6	0.00	2.4
A_52_P311853	NM_030143	Ddit4l	0.20	1.3	0.02	1.5	0.00	2.4
A_51_P449824	XM_001471750	Exoc3l2	0.77	1.2	0.73	1.2	0.00	2.4
A_51_P350403	NM_009148	Exoc4	0.02	1.4	0.00	1.8	0.00	2.4
A_52_P686785	NM_053247	Lyve1	0.00	1.9	0.00	2.0	0.00	2.4
A_51_P464918	NM_019453	Mefv	0.00	2.1	0.01	1.6	0.00	2.4
A_51_P146560	NM_018857	Msln	0.81	1.3	0.94	1.1	0.03	2.4
A_51_P139651	NM_008713	Nos3	0.10	1.4	0.05	1.4	0.00	2.4
A_55_P2006008	NM_025429	Serpinb1a	0.01	1.4	0.00	1.8	0.00	2.4
A_51_P267933	NM_175692	Snhg11	0.06	2.0	0.04	1.9	0.00	2.4
A_51_P415220	NM_009517	Zmat3	0.08	1.3	0.00	1.6	0.00	2.4
A_30_P01027010			0.00	1.6	0.00	1.9	0.00	2.3
A_30_P01020960			0.00	1.6	0.00	1.9	0.00	2.3
A_66_P115580	AK076360		0.27	1.4	0.05	1.5	0.00	2.3
A_51_P112308	NM_026931	1810011O10Rik	0.92	-1.0	0.79	1.1	0.00	2.3
A_51_P128987	NM_008012	Akr1b8	0.95	1.0	0.89	1.1	0.00	2.3
A_52_P487686	NM_001082546	BC100530	0.02	3.1	0.60	1.4	0.03	2.3
A_51_P181286	NM_001033122	Cd69	0.09	1.4	0.00	1.6	0.00	2.3
A_51_P401907	NM_001082547	Gm5483	0.72	1.2	0.29	1.4	0.00	2.3
A_51_P187750	NM_153408	Neurl3	0.98	-1.0	0.74	1.2	0.00	2.3
A_51_P256827	NM_013650	S100a8	1.00	-1.0	0.19	1.6	0.00	2.3
A_55_P2094925	NM_011157	Srgn	0.02	1.4	0.00	1.9	0.00	2.3
A_52_P398925	NM_173869	Stfa2l1	0.61	1.3	0.14	1.5	0.00	2.3
A_51_P175580	NM_021897	Trp53inp1	0.00	1.7	0.00	2.1	0.00	2.3
A_55_P2007713	NM_053082	Tspan4	0.00	1.6	0.00	1.8	0.00	2.3
A_30_P01025511			0.00	1.7	0.01	1.5	0.00	2.2
A_55_P2023542	NM_007719	Ccr7	0.41	1.3	0.00	1.8	0.00	2.2
A_52_P70796	NM_007551	Cxcr5	0.10	1.5	0.06	1.5	0.00	2.2
A_52_P325527	NM_010103	Edil3	0.93	1.0	0.60	1.2	0.00	2.2
A_55_P2002578	NM_010145	Ephx1	0.00	1.6	0.00	1.7	0.00	2.2

A_55_P2063426	XR_004815	Gm5649	0.91	1.1	0.83	1.1	0.00	2.2
A_55_P2109122	NM_023422	Hist1h2bc	0.24	1.2	0.00	1.5	0.00	2.2
A_51_P371750	NM_010766	Marco	0.39	1.5	0.09	1.8	0.00	2.2
A_55_P2090025	NM_008590	Mest	0.65	1.2	0.57	1.2	0.00	2.2
A_51_P270184	NM_025811	Nhlrc2	0.73	1.1	0.07	1.3	0.00	2.2
A_51_P282760	NM_011066	Per2	0.08	1.5	0.04	1.5	0.00	2.2
A_52_P236705	NM_133229	Ripply3	0.40	1.3	0.00	1.8	0.00	2.2
A_55_P2008936	NM_001102414	Slc2a9	0.05	1.4	0.01	1.5	0.00	2.2
A_55_P2039320	NM_178679	Zfp365	0.19	1.3	0.00	1.6	0.00	2.2
A_30_P01026536			0.04	1.4	0.00	1.6	0.00	2.1
A_30_P01026613			0.81	1.1	0.18	1.4	0.00	2.1
A_55_P2136121	NM_145448	9030617O03Rik	0.00	1.5	0.00	1.8	0.00	2.1
A_51_P424959	NM_007528	Bcl6b	0.00	1.8	0.01	1.7	0.00	2.1
A_55_P2124791	NM_001109991	Col18a1	1.00	1.0	0.98	1.0	0.00	2.1
A_51_P498631	NM_018769	Dfna5	0.81	1.1	0.27	1.3	0.00	2.1
A_55_P2056729	NM_008342	Igfbp2	0.00	1.7	0.00	1.8	0.00	2.1
A_51_P212782	NM_008361	Il1b	0.53	1.4	0.30	1.4	0.00	2.1
A_55_P2064333	NM_010680	Lama3	0.07	1.7	0.29	1.4	0.00	2.1
A_55_P2034027	NM_008713	Nos3	0.19	1.3	0.38	1.2	0.00	2.1
A_51_P424338	NM_008706	Nqo1	0.97	-1.0	0.82	-1.1	0.00	2.1
A_52_P587738	NM_008773	P2ry2	0.30	1.3	0.40	1.2	0.00	2.1
A_51_P383194	NM_008804	Pde9a	0.00	1.5	0.00	1.5	0.00	2.1
A_55_P2173313	NM_019677	Plcb1	0.03	1.6	0.00	2.0	0.00	2.1
A_55_P2107155	NM_029182	Rasd2	0.53	1.3	0.02	1.8	0.00	2.1
A_52_P425839	NM_181596	Retnlg	0.93	1.1	0.53	1.3	0.00	2.1
A_51_P329332	NM_054087	Slc19a2	0.01	1.6	0.01	1.6	0.00	2.1
A_55_P2068673	NM_025285	Stmn2	0.30	1.2	0.00	1.6	0.00	2.1
A_55_P1979893	NM_017376	Tef	0.00	1.7	0.00	1.9	0.00	2.1
A_55_P2088145			0.05	1.5	0.03	1.6	0.00	2.0
A_30_P01025790			0.03	1.4	0.00	1.6	0.00	2.0
A_30_P01022788			0.89	1.1	0.09	1.3	0.00	2.0
A_51_P367780	NM_029981	Adamtsl2	0.07	1.5	0.30	1.3	0.00	2.0
A_55_P2169227	NM_177716	AI836003	0.59	1.2	0.22	1.3	0.00	2.0
A_55_P2002849	NM_175178	Aifm3	0.38	1.2	0.28	1.2	0.00	2.0
A_55_P2059352	NM_001109991	Col18a1	1.00	1.0	0.98	-1.0	0.00	2.0
A_51_P461429	NM_007825	Cyp7b1	0.94	-1.0	0.47	1.1	0.00	2.0
A_55_P1959763	NM_007826	Dach1	0.27	1.7	0.06	2.0	0.02	2.0
A_51_P110471	NM_026993	Ddah1	0.00	1.8	0.00	1.9	0.00	2.0
A_55_P1964183	NM_001077694	Dysf	0.84	1.1	0.76	1.1	0.00	2.0
A_66_P104815	NM_007899	Ecm1	0.97	-1.0	0.60	1.2	0.00	2.0
A_55_P2002577	NM_010145	Ephx1	0.00	1.5	0.00	1.7	0.00	2.0
A_52_P63343	NM_001033302	Gm129	0.23	1.3	0.03	1.4	0.00	2.0
A_51_P247694	NM_173036	Gpr97	0.09	1.4	0.21	1.3	0.00	2.0
A_55_P2009752	NM_172563	Hlf	0.16	1.5	0.16	1.4	0.00	2.0
A_55_P2085295	NM_198411	Inf2	0.25	1.2	0.08	1.3	0.00	2.0
A_66_P106388	NM_029499	Ms4a4c	0.26	1.4	0.05	1.6	0.00	2.0
A_55_P1953728	NM_016701	Nes	0.05	1.3	0.01	1.4	0.00	2.0
A_55_P2008634	NM_053135	Pcdhb10	0.60	1.2	0.15	1.4	0.00	2.0
A_55_P2000158	NM_181585	Pik3r3	0.49	1.2	0.07	1.4	0.00	2.0

A_51_P229602	NM_008882	Plxna2	0.34	1.3	0.09	1.4	0.00	2.0
A_51_P382152	NM_011171	Procr	0.83	1.1	0.87	1.1	0.00	2.0
A_52_P318673	NM_009117	Saa1	0.92	-1.1	0.67	-1.2	0.00	2.0
A_55_P2003813	NM_153522	Scn3b	0.00	1.7	0.00	1.7	0.00	2.0
A_55_P1965154	NM_025565	Spc25	0.00	5.1	0.00	4.3	0.00	2.0
A_55_P2035286	NM_010931	Uhrf1	0.00	2.4	0.00	2.5	0.00	2.0
A_30_P01027827			0.03	1.6	0.00	1.7	0.00	1.9
A_30_P01032002			0.02	1.6	0.06	1.4	0.00	1.9
A_30_P01032951			0.02	1.3	0.00	1.7	0.00	1.9
A_55_P1953533	AK134774		0.68	1.3	0.60	1.3	0.01	1.9
A_30_P01032234			0.79	1.2	0.31	1.4	0.01	1.9
A_30_P01023701			0.22	1.5	0.12	1.5	0.00	1.9
A_30_P01021048			0.35	1.2	0.02	1.4	0.00	1.9
A_30_P01031037			0.71	1.1	0.37	1.2	0.00	1.9
A_55_P2055819	NM_001037722	Adam15	0.32	1.3	0.04	1.4	0.00	1.9
A_55_P2143070	NM_007494	Ass1	0.14	1.3	0.02	1.4	0.00	1.9
A_55_P1969356	NM_145536	BC020535	0.01	1.5	0.00	1.7	0.00	1.9
A_52_P469789	NM_007588	Calcr	0.88	1.1	0.70	1.2	0.00	1.9
A_55_P2079079	NM_009844	Cd19	0.01	1.7	0.00	1.8	0.00	1.9
A_55_P2394490	AK052609	D630004K10Rik	0.39	1.3	0.56	1.2	0.00	1.9
A_51_P153063	NM_029310	Fabp12	0.40	1.3	0.19	1.4	0.00	1.9
A_51_P142153	NM_030163	Filip1l	0.02	1.4	0.00	1.4	0.00	1.9
A_55_P2010567	XM_975768	Gm7564	0.32	1.3	0.32	1.3	0.00	1.9
A_51_P180140	NM_175663	Hist1h2ba	0.54	1.2	0.06	1.3	0.00	1.9
A_55_P2177154	NM_178200	Hist1h2bm	0.37	1.2	0.03	1.3	0.00	1.9
A_52_P615375	NM_178218	Hist3h2a	0.30	1.3	0.09	1.3	0.00	1.9
A_55_P1989956	NM_001081298	Lphn2	0.77	1.1	0.28	1.2	0.00	1.9
A_55_P2148534	BC096461	Nr1d2	0.03	1.7	0.00	1.8	0.00	1.9
A_52_P303891	NM_011584	Nr1d2	0.01	1.8	0.00	1.9	0.00	1.9
A_55_P2178678	NM_033321	P2rx5	0.96	1.0	0.91	1.1	0.00	1.9
A_55_P2090359	NM_148937	Plcd4	0.83	1.1	0.61	1.1	0.00	1.9
A_52_P679105	NM_029614	Prss23	0.42	1.3	0.10	1.5	0.00	1.9
A_55_P2168628	NM_133678	Sac3d1	0.00	1.4	0.00	1.6	0.00	1.9
A_55_P1960238	NM_172659	Slc2a6	0.75	1.2	0.30	1.3	0.00	1.9
A_52_P193925	NM_028072	Sulf2	0.80	1.1	0.55	1.2	0.00	1.9
A_51_P448664	NM_011539	Tbxas1	0.49	1.4	0.26	1.5	0.00	1.9
A_55_P2081616	NM_001164081	Timeless	0.00	1.6	0.00	1.7	0.00	1.9
A_51_P269203	NM_022017	Trpv4	0.49	1.4	0.47	1.4	0.02	1.9
A_55_P1973770	NM_029770	Unc5b	0.97	1.0	0.52	1.2	0.00	1.9
A_55_P2071952	NM_178909	Wdr92	0.99	1.0	0.70	-1.2	0.02	1.9
A_55_P1976204	U09507		0.02	1.5	0.05	1.4	0.00	1.8
A_52_P431859	AK167772		0.00	1.7	0.01	1.4	0.00	1.8
A_30_P01027042			0.00	1.8	0.01	1.5	0.00	1.8
A_30_P01021631			0.00	2.2	0.02	1.5	0.00	1.8
A_30_P01029956			0.03	1.3	0.00	1.4	0.00	1.8
A_30_P01031506			0.36	1.3	0.19	1.4	0.00	1.8
A_30_P01021359			0.22	1.2	0.00	1.4	0.00	1.8
A_55_P2019577	NR_027818	1500011B03Rik	0.38	1.4	0.13	1.6	0.01	1.8
A_55_P2262593	AK142388	4930429F24Rik	0.11	1.3	0.06	1.3	0.00	1.8

A_51_P113178	NM_175398	6530418L21Rik	0.10	1.4	0.01	1.5	0.00	1.8
A_51_P245368	NM_011075	Abcb1b	0.53	1.2	0.16	1.2	0.00	1.8
A_55_P2013158	NM_178688	Ablim1	0.63	1.1	0.00	1.4	0.00	1.8
A_55_P2320313	BC058714	AI852064	0.70	1.1	0.15	1.3	0.00	1.8
A_51_P392005	NM_007592	Car8	0.38	-1.4	0.31	1.4	0.01	1.8
A_55_P2079927	NM_201362	Ccdc68	0.11	1.4	0.01	1.6	0.00	1.8
A_55_P2052062	NM_010818	Cd200	0.15	1.4	0.01	1.6	0.00	1.8
A_51_P491350	NM_009932	Col4a2	0.40	1.2	0.21	1.2	0.00	1.8
A_51_P300506	NM_183405	Cox6b2	0.07	1.3	0.18	1.2	0.00	1.8
A_51_P384629	NM_009983	Ctsd	0.81	1.1	0.29	1.2	0.00	1.8
A_52_P559975	NM_009909	Cxcr2	0.54	1.3	0.51	1.2	0.00	1.8
A_55_P1980262	NM_001018063	Cxx1b	0.80	1.1	0.16	1.3	0.00	1.8
A_55_P2111355	NM_178444	Egfl7	0.00	1.5	0.00	1.5	0.00	1.8
A_51_P140641	NM_145570	Fam176a	0.39	1.2	0.12	1.3	0.00	1.8
A_51_P351896	NM_133187	Fam198b	0.46	1.3	0.15	1.4	0.00	1.8
A_51_P468464	NM_201352	Gdpd5	0.79	1.1	0.61	1.2	0.00	1.8
A_55_P2075919	NM_001145015	Gm14203	0.28	1.3	0.36	1.3	0.00	1.8
A_55_P1960936	XM_001473982	Gm2562	0.98	-1.0	1.00	-1.0	0.01	1.8
A_55_P2114779	NM_015764	Greb1	0.79	-1.2	0.75	1.2	0.01	1.8
A_55_P2114776	NM_015764	Greb1	0.58	1.2	0.52	1.2	0.00	1.8
A_51_P172231	NM_026960	Gsdmd	0.12	1.3	0.11	1.3	0.00	1.8
A_55_P2037343	NM_010391	H2-Q10	0.09	1.5	0.45	1.2	0.00	1.8
A_66_P135391	NM_008342	Igfbp2	0.03	1.5	0.00	1.6	0.00	1.8
A_51_P451338	NM_010588	Jag2	0.75	1.1	0.66	1.1	0.00	1.8
A_55_P2086203	NM_008434	Kcnq1	0.22	1.2	0.02	1.3	0.00	1.8
A_55_P2122020	NM_010637	Klf4	0.03	1.4	0.14	1.3	0.00	1.8
A_51_P354706	NM_010094	Lefty1	0.01	1.5	0.00	1.5	0.00	1.8
A_52_P137765	NM_019390	Lmna	0.04	1.3	0.19	1.2	0.00	1.8
A_55_P2095311	XM_001475753	Ly6g	0.20	1.3	0.19	1.3	0.00	1.8
A_55_P2071858	NM_008598	Mgmt	0.16	1.3	0.03	1.4	0.00	1.8
A_55_P2118694	NM_027280	Nkd1	0.59	1.2	0.12	1.4	0.00	1.8
A_55_P1967736	NM_027280	Nkd1	0.40	1.2	0.05	1.4	0.00	1.8
A_55_P2103452	NM_010934	Npy1r	0.95	-1.0	0.29	1.2	0.00	1.8
A_52_P393314	NM_011027	P2rx7	0.28	1.2	0.12	1.3	0.00	1.8
A_51_P337771	NM_053142	Pcdhb17	0.75	1.1	0.09	1.3	0.00	1.8
A_51_P258493	NM_011067	Per3	0.03	1.5	0.00	1.6	0.00	1.8
A_52_P302433	NM_008873	Plau	0.36	1.8	0.04	2.4	0.14	1.8
A_55_P1960496	NM_025800	Ppp1r2	0.57	1.2	0.15	1.3	0.00	1.8
A_51_P332652	NM_172574	Pqlc3	0.24	1.3	0.05	1.4	0.00	1.8
A_51_P170959	AK005011	Proz	0.76	-1.2	0.99	1.0	0.01	1.8
A_55_P2039225	NM_001081155	Rap1gap	0.67	1.1	0.04	1.4	0.00	1.8
A_51_P282508	NM_007484	Rhoc	0.22	1.3	0.08	1.3	0.00	1.8
A_55_P2304864	NM_011282	Ros1	0.85	1.1	0.39	1.3	0.00	1.8
A_51_P246903	NM_026467	Rps27l	0.00	1.5	0.00	1.6	0.00	1.8
A_52_P73620	NM_198028	Serpinb10	0.02	1.9	0.16	1.5	0.01	1.8
A_55_P2121608	AK153771	Sox4	0.32	1.3	0.04	1.5	0.00	1.8
A_52_P571371	NM_027495	Tmem144	0.31	1.3	0.01	1.5	0.00	1.8
A_65_P10673	NM_172614	Tmem44	0.02	1.5	0.00	1.7	0.00	1.8
A_52_P112110	NM_145987	Tmem82	0.24	1.3	0.03	1.4	0.00	1.8

A_52_P260555	NM_009509	Vil1	0.88	1.1	0.50	1.2	0.00	1.8
A_30_P01023785			0.17	1.5	0.16	1.5	0.01	1.7
A_30_P01018535			0.94	1.1	0.47	1.3	0.01	1.7
A_52_P425092			0.83	1.1	0.61	1.2	0.00	1.7
A_55_P2021149	AK146816		0.46	1.2	0.37	1.2	0.00	1.7
A_52_P670263	BC036146		0.92	1.0	0.65	1.1	0.00	1.7
A_30_P01026167			0.34	1.2	0.04	1.4	0.00	1.7
A_30_P01019638			0.03	1.3	0.00	1.3	0.00	1.7
A_30_P01028922			0.76	1.1	0.81	1.1	0.00	1.7
A_51_P301636	NM_001109685	9030409G11Rik	0.10	1.3	0.25	1.2	0.00	1.7
A_52_P665675	NM_013454	Abca1	0.88	1.1	0.05	1.4	0.00	1.7
A_55_P2082688	NM_021515	Ak1	1.00	1.0	0.95	-1.0	0.00	1.7
A_55_P2142430	NM_001033350	Bank1	0.17	1.4	0.03	1.6	0.00	1.7
A_55_P2029846	NM_001113283	BC031353	0.00	1.4	0.00	1.5	0.00	1.7
A_51_P110301	NM_009778	C3	0.86	-1.1	0.64	-1.2	0.02	1.7
A_51_P343252	NM_009842	Cd151	0.72	1.1	0.11	1.2	0.00	1.7
A_55_P2181904	NM_027152	Cd164l2	0.99	1.0	0.77	1.1	0.00	1.7
A_66_P101835	NM_007646	Cd38	0.78	1.1	0.10	1.4	0.00	1.7
A_51_P372550	NM_026770	Cgref1	0.01	1.7	0.05	1.5	0.00	1.7
A_55_P2148906	NM_028870	Cltb	0.72	1.1	0.37	1.1	0.00	1.7
A_55_P1971724	NM_007758	Cr2	0.00	1.6	0.00	1.8	0.00	1.7
A_55_P2016462	NM_021274	Cxcl10	0.94	-1.1	0.67	-1.2	0.04	1.7
A_55_P2344608	AK081893	D7Wsu130e	0.67	1.2	0.56	1.2	0.00	1.7
A_55_P2182026	NM_183160	E030010A14Rik	0.77	1.2	0.48	1.3	0.04	1.7
A_55_P1954221	NM_010128	Emp1	0.00	1.3	0.12	1.2	0.00	1.7
A_52_P518997	NM_010139	Epha2	0.20	1.3	0.07	1.3	0.00	1.7
A_52_P365011	NM_010194	Fes	0.01	1.4	0.00	1.3	0.00	1.7
A_55_P2026547	NM_199366	Gal3st2	0.04	1.5	0.08	1.4	0.00	1.7
A_52_P351669	NM_027375	Gcc2	0.03	1.3	0.00	1.4	0.00	1.7
A_51_P394847	NR_024599	Gm11346	0.62	1.1	0.05	1.4	0.00	1.7
A_55_P2105843	XM_001472740	Gm2371	0.95	-1.1	0.96	-1.0	0.01	1.7
A_55_P1954092	XM_001473985	Gm2563	0.87	-1.1	0.92	-1.1	0.01	1.7
A_52_P69558	XM_001475933	Gm8221	0.98	1.0	0.87	1.1	0.00	1.7
A_55_P2084691	NM_178189	Hist1h2ac	0.74	1.1	0.32	1.2	0.00	1.7
A_55_P2036813	NM_030082	Hist3h2ba	0.56	1.2	0.49	1.2	0.00	1.7
A_51_P179701	NM_008250	Hlx	0.61	1.1	0.14	1.2	0.00	1.7
A_55_P2052563	NM_010495	Id1	0.06	1.8	0.44	1.3	0.02	1.7
A_51_P470079	NM_010555	Il1r2	0.25	1.5	0.82	1.1	0.02	1.7
A_55_P1998299	NM_001005608	Itgb4	0.48	1.2	0.84	1.1	0.00	1.7
A_52_P350554	NM_008420	Kcnb1	0.81	1.1	0.31	1.3	0.00	1.7
A_55_P2145617	NM_028770	Krt80	0.66	1.1	0.21	1.2	0.00	1.7
A_55_P2094641	XM_001474594	LOC100044122	0.57	1.2	0.18	1.2	0.00	1.7
A_52_P480351	NM_033325	Loxl2	0.80	1.1	0.64	1.1	0.00	1.7
A_51_P234728	AK032901	Lpar6	0.58	1.2	0.16	1.3	0.00	1.7
A_51_P295237	NM_172784	Lrp11	0.89	1.0	0.61	1.1	0.00	1.7
A_55_P2039250	NM_026037	Mboat2	0.21	1.4	0.31	1.3	0.00	1.7
A_51_P360492	NM_008567	Mcm6	0.06	1.9	0.00	2.2	0.02	1.7
A_51_P426270	NM_008597	Mgp	0.60	1.2	0.32	1.2	0.00	1.7
A_52_P167278	NM_172308	Mthfd1l	0.54	1.2	0.01	1.4	0.00	1.7

A_55_P2042086	NM_008738	Nrtn	0.67	1.1	0.33	1.2	0.00	1.7
A_55_P2142172	NM_001011761	Olfr1229	0.00	1.4	0.00	1.5	0.00	1.7
A_52_P111031	NM_001013753	Pcdh17	0.58	1.2	0.12	1.3	0.00	1.7
A_55_P2081488	NM_009402	Pglyrp1	0.18	1.3	0.61	1.1	0.00	1.7
A_55_P2170813	NM_001164053	Pkig	0.46	1.2	0.02	1.4	0.00	1.7
A_66_P119034	NM_013737	Pla2g7	0.47	1.2	0.62	1.2	0.00	1.7
A_51_P290576	NM_152804	Plk2	0.33	1.3	0.02	1.5	0.00	1.7
A_55_P2000533	NM_012048	Polk	0.77	1.1	0.23	1.2	0.00	1.7
A_55_P2089233	NM_011136	Pou2af1	0.35	1.3	0.00	1.6	0.00	1.7
A_55_P1992084	NM_008981	Ptprg	0.41	1.2	0.02	1.4	0.00	1.7
A_55_P2105638	NM_001033348	Ralgapa2	0.53	1.2	0.05	1.3	0.00	1.7
A_55_P2068096	NM_001081155	Rap1gap	0.58	1.2	0.05	1.4	0.00	1.7
A_55_P2010197	NM_144834	Serpina10	0.68	-1.2	0.61	1.2	0.00	1.7
A_51_P161354	NM_144907	Sesn2	0.06	1.4	0.17	1.3	0.00	1.7
A_51_P366672	NM_153170	Slc36a2	0.31	1.2	0.32	1.2	0.00	1.7
A_55_P2187076	NM_011430	Sncg	0.08	1.3	0.00	1.5	0.00	1.7
A_51_P236267	NM_009183	St8sia4	0.75	1.1	0.01	1.5	0.00	1.7
A_52_P62444	NM_013681	Syn2	0.36	1.4	0.09	1.5	0.01	1.7
A_55_P2168168	NM_031384	Tex11	0.03	1.5	0.00	1.8	0.00	1.7
A_51_P291417	NM_009378	Thbd	0.85	1.1	0.02	1.7	0.00	1.7
A_55_P2102295	NM_145599	Tmem184c	0.31	1.3	0.24	1.2	0.00	1.7
A_51_P243808	NM_153137	Traf3ip3	0.09	1.4	0.00	1.6	0.00	1.7
A_55_P2067453	NM_028841	Tspan17	0.31	1.2	0.02	1.4	0.00	1.7
A_66_P136186	NM_009516	Wee1	0.41	1.3	0.20	1.3	0.00	1.7
A_51_P220343	NM_018865	Wisp1	0.26	1.3	0.05	1.4	0.00	1.7
A_30_P01018771			0.00	2.0	0.00	1.7	0.00	1.6
A_30_P01030953			0.00	2.2	0.00	1.7	0.00	1.6
A_30_P01022528			0.22	1.3	0.00	1.5	0.00	1.6
A_55_P2159555	AK028364		0.85	1.1	0.83	1.1	0.04	1.6
A_30_P01033517			0.22	1.4	0.49	1.2	0.01	1.6
A_30_P01022942			0.64	1.1	0.71	1.1	0.00	1.6
A_52_P336768			0.53	1.1	0.00	1.4	0.00	1.6
A_30_P01027743			0.30	1.3	0.06	1.4	0.00	1.6
A_30_P01030148			0.85	1.1	0.38	1.2	0.00	1.6
A_30_P01020003			0.50	1.2	0.10	1.3	0.00	1.6
A_30_P01019169			0.42	1.2	0.00	1.4	0.00	1.6
A_30_P01019146			0.31	1.3	0.11	1.4	0.00	1.6
A_30_P01019476			0.71	1.1	0.57	1.1	0.00	1.6
A_30_P01025143			0.40	1.2	0.06	1.3	0.00	1.6
A_30_P01026520			0.17	1.3	0.14	1.3	0.00	1.6
A_30_P01024670			0.27	1.1	0.01	1.3	0.00	1.6
A_55_P2184449	AK134871		0.01	1.4	0.26	1.2	0.00	1.6
A_30_P01033274			0.20	1.3	0.01	1.4	0.00	1.6
A_30_P01026752			0.46	1.2	0.13	1.3	0.00	1.6
A_55_P2129316			0.81	1.1	0.32	1.2	0.00	1.6
A_51_P252677	NM_026738	1110007C09Rik	0.03	1.2	0.08	1.2	0.00	1.6
A_52_P267824	NM_197999	2210023G05Rik	0.09	1.4	0.32	1.2	0.00	1.6
A_55_P2095899	XM_001476219	2310057B04Rik	0.22	1.2	0.13	1.3	0.00	1.6
A_51_P214470	NM_173744	2610019F03Rik	0.62	1.1	0.08	1.3	0.00	1.6

A_55_P2026982	NM_173744	2610019F03Rik	0.99	1.0	0.47	1.2	0.00	1.6
A_51_P358243	NM_029840	2610029I01Rik	0.26	1.2	0.01	1.4	0.00	1.6
A_51_P369252	NM_001080995	4632434I11Rik	0.01	1.5	0.00	1.6	0.00	1.6
A_55_P2279807	AK160312	6720427I07Rik	0.00	2.1	0.00	1.6	0.00	1.6
A_66_P130310	NM_001170954	A4galt	0.50	1.1	0.04	1.3	0.00	1.6
A_55_P2172396	NM_178796	A530064D06Rik	0.65	1.1	0.43	1.2	0.00	1.6
A_51_P374869	NM_013850	Abca7	0.07	1.3	0.10	1.3	0.00	1.6
A_55_P1953846	NM_013851	Abca8b	0.39	1.2	0.30	1.2	0.00	1.6
A_55_P1969477	NM_011076	Abcb1a	0.39	1.3	0.27	1.3	0.00	1.6
A_52_P489778	NM_178688	Ablim1	0.47	1.1	0.00	1.5	0.00	1.6
A_55_P2047305	NM_001012765	Adcy5	0.55	1.3	0.93	1.0	0.02	1.6
A_51_P259603	NM_007407	Adcyap1r1	0.63	1.2	0.57	1.2	0.03	1.6
A_55_P1988623	NM_001164099	Add3	0.40	1.2	0.00	1.3	0.00	1.6
A_55_P2004248	NM_133237	Apcdd1	0.99	-1.0	0.02	2.6	0.29	1.6
A_55_P2123491	NM_001159407	B3gnt5	0.18	1.4	0.10	1.4	0.01	1.6
A_52_P277104	NM_001033350	Bank1	0.27	1.5	0.03	1.8	0.02	1.6
A_51_P165182	NM_028967	Batf2	0.92	1.1	0.60	1.2	0.00	1.6
A_55_P2100739	NM_145601	BC016201	0.18	1.2	0.02	1.4	0.00	1.6
A_55_P1981836	NM_145536	BC020535	0.01	1.5	0.01	1.5	0.00	1.6
A_55_P2018191	NM_145536	BC020535	0.06	1.5	0.02	1.5	0.00	1.6
A_55_P2073308	NM_009668	Bin1	0.21	1.2	0.04	1.3	0.00	1.6
A_55_P2115401	NM_009763	Bst1	0.78	1.2	0.47	1.3	0.03	1.6
A_55_P2042612	NM_144938	C1s	0.77	1.2	0.67	1.2	0.01	1.6
A_55_P2038525	NM_009778	C3	0.97	-1.0	0.69	-1.2	0.04	1.6
A_55_P2078633	NM_009780	C4b	0.82	-1.2	0.76	-1.2	0.04	1.6
A_66_P126640	NM_007592	Car8	0.67	-1.2	0.31	1.4	0.04	1.6
A_55_P2079928	NM_201362	Ccdc68	0.02	1.4	0.07	1.3	0.00	1.6
A_52_P53906	NM_009829	Ccnd2	0.45	1.2	0.06	1.4	0.00	1.6
A_55_P2041075	NM_001081636	Ccnd3	0.41	1.2	0.04	1.3	0.00	1.6
A_52_P422494	NM_145634	Cd300lf	0.58	1.2	0.19	1.4	0.00	1.6
A_55_P2081164	NM_001169153	Cd300lf	0.21	1.3	0.13	1.3	0.00	1.6
A_52_P248604	NM_009868	Cdh5	0.84	1.1	0.59	1.1	0.00	1.6
A_51_P383032	NM_010819	Clec4d	0.93	1.1	0.67	1.2	0.03	1.6
A_51_P357341	NM_033444	Clic1	0.02	1.3	0.00	1.3	0.00	1.6
A_51_P413785	NM_147778	Commd3	0.01	1.4	0.00	1.4	0.00	1.6
A_66_P115531	NM_145151	Crebzf	0.00	1.7	0.05	1.3	0.00	1.6
A_51_P423976	NM_013498	Crem	0.77	1.1	0.03	1.3	0.00	1.6
A_55_P2038106	NM_009978	Cst8	0.96	1.0	0.05	1.5	0.01	1.6
A_55_P1985591	NM_024170	Cxx1a	0.73	1.1	0.33	1.2	0.00	1.6
A_55_P2133266	NM_028375	Cxx1c	0.74	1.1	0.24	1.2	0.00	1.6
A_55_P2124941	NM_019967	Dbc1	0.43	1.2	0.22	1.2	0.00	1.6
A_55_P1968928	NM_027185	Def6	0.02	1.3	0.02	1.3	0.00	1.6
A_52_P578790	NM_013739	Dok3	0.12	1.3	0.00	1.5	0.00	1.6
A_55_P2150496	NM_010063	Dync1i1	0.98	-1.0	0.87	1.1	0.00	1.6
A_52_P583458	NM_010093	E2f3	0.13	1.2	0.02	1.3	0.00	1.6
A_55_P2097022	NM_025613	Eid1	0.89	1.0	0.11	1.3	0.00	1.6
A_55_P2022861	NM_001039176	Elovl1	0.75	1.1	0.18	1.3	0.00	1.6
A_55_P1954277	NM_080595	Emid1	0.90	-1.1	0.75	1.1	0.01	1.6
A_51_P507801	NM_028784	F13a1	0.54	1.3	0.46	1.3	0.02	1.6

A_52_P5891	NM_029658	Fam101b	0.78	1.1	0.27	1.3	0.00	1.6
A_55_P2139077	NM_001039485	Fam38b	0.82	-1.1	0.96	-1.0	0.00	1.6
A_55_P2076448	NM_172930	Fam70a	0.90	1.0	0.89	1.0	0.00	1.6
A_51_P154485	NM_145141	Fcrla	0.29	1.4	0.05	1.6	0.03	1.6
A_55_P2064572	NM_172731	Fgd5	0.30	1.3	0.26	1.3	0.00	1.6
A_55_P2100884	NM_010218	Fjx1	0.04	1.4	0.25	1.2	0.00	1.6
A_55_P1981259	NM_153573	Fkbp14	0.71	1.1	0.05	1.3	0.00	1.6
A_55_P2097478	NM_010266	Gda	0.95	-1.0	0.40	1.2	0.00	1.6
A_55_P2115189	XM_001477969	Gm3796	0.11	1.3	0.13	1.2	0.00	1.6
A_51_P171200	NM_027307	Golm1	0.29	1.3	0.50	1.2	0.00	1.6
A_52_P68221	NM_016886	Gria3	0.01	1.4	0.17	1.2	0.00	1.6
A_55_P1970090	NM_010350	Grin2c	0.18	1.6	0.16	1.5	0.04	1.6
A_55_P2099540	NM_177688	H2afj	0.03	1.3	0.02	1.3	0.00	1.6
A_55_P2109128	NM_178194	Hist1h2be	0.97	-1.0	0.46	1.1	0.00	1.6
A_55_P2131438	NM_178212	Hist2h2aa2	0.00	1.4	0.00	1.4	0.00	1.6
A_55_P1982762	NM_133943	Hsd3b7	0.08	1.3	0.00	1.4	0.00	1.6
A_55_P2021114	NM_010500	Ier5	0.19	1.3	0.02	1.5	0.00	1.6
A_51_P125467	NM_010515	Igf2r	0.14	1.2	0.02	1.3	0.00	1.6
A_55_P1964368	NM_001012434	Kctd14	0.36	1.3	0.24	1.3	0.00	1.6
A_51_P361830	NM_144945	Lgi2	0.34	1.3	0.07	1.4	0.00	1.6
A_55_P1978866	XM_001472829	LOC100039402	0.83	-1.1	0.96	-1.0	0.01	1.6
A_55_P2130501	XM_001472813	LOC100044727	0.89	-1.1	0.72	-1.1	0.00	1.6
A_55_P2042016	XM_001479435	LOC100048058	0.98	1.0	0.16	1.2	0.00	1.6
A_55_P1967069	NM_175116	Lpar6	0.73	1.1	0.31	1.2	0.00	1.6
A_51_P487813	NM_016753	Lxn	0.02	1.4	0.01	1.4	0.00	1.6
A_55_P2064771	NM_010741	Ly6c1	0.78	1.1	0.53	1.1	0.00	1.6
A_55_P1961499	NM_010741	Ly6c1	0.88	1.1	0.54	1.1	0.00	1.6
A_55_P2106039	NM_016693	Map3k6	0.70	1.1	0.86	1.1	0.00	1.6
A_55_P1987914	NM_008696	Map4k4	0.01	1.5	0.00	1.5	0.00	1.6
A_52_P168449	NM_011161	Mapk11	0.04	1.3	0.01	1.4	0.00	1.6
A_55_P2078433	NM_026656	Mcoln2	0.23	1.3	0.02	1.5	0.00	1.6
A_55_P1971897	NM_010784	Mdk	0.30	1.3	0.58	1.1	0.00	1.6
A_55_P1975475	NM_010786	Mdm2	0.22	1.2	0.12	1.2	0.00	1.6
A_55_P2151868	NM_008590	Mest	0.42	1.2	0.89	1.1	0.00	1.6
A_55_P1967291	NM_144818	Ncaph	0.00	2.4	0.00	2.2	0.01	1.6
A_51_P405606	NM_008681	Ndrg1	0.11	1.4	0.02	1.5	0.00	1.6
A_51_P223776	NM_145434	Nr1d1	0.02	1.7	0.01	1.7	0.00	1.6
A_55_P2105963	NM_172435	P2ry10	0.54	1.2	0.32	1.3	0.01	1.6
A_55_P1980321	NM_021568	Pcbp3	0.61	1.1	0.19	1.2	0.00	1.6
A_55_P2035159	NM_029357	Pcdh1	0.73	1.1	0.24	1.2	0.00	1.6
A_55_P2087087	NM_001032378	Pecam1	0.41	1.3	0.13	1.3	0.00	1.6
A_51_P363801	NM_023217	Pgpep1	0.40	1.2	0.04	1.3	0.00	1.6
A_52_P513347	NM_199446	Phkb	0.42	1.2	0.10	1.2	0.00	1.6
A_55_P2166773	NM_008850	Pitpna	0.15	1.2	0.03	1.3	0.00	1.6
A_51_P239766	NM_019676	Plcd1	0.12	1.3	0.41	1.2	0.00	1.6
A_55_P2433218	NM_019788	Pldn	0.46	1.2	0.16	1.3	0.00	1.6
A_51_P375201	NM_013807	Plk3	0.02	1.4	0.07	1.3	0.00	1.6
A_51_P302458	NM_023564	Plscr3	0.28	1.1	0.04	1.2	0.00	1.6
A_55_P1954086	NM_015784	Postn	0.04	1.4	0.03	1.4	0.00	1.6

A_55_P2236291	NM_008903	Ppap2a	0.95	-1.0	0.94	-1.0	0.00	1.6
A_55_P2109263	NM_008889	Ppp1r14b	0.16	1.2	0.02	1.3	0.00	1.6
A_51_P279100	NM_008969	Ptgs1	0.92	1.0	0.61	1.1	0.00	1.6
A_55_P2116465	NM_008981	Ptprg	0.25	1.3	0.26	1.2	0.00	1.6
A_55_P2035509	NM_175026	Pyhin1	0.15	1.4	0.16	1.4	0.01	1.6
A_55_P1956567	NM_023852	Rab3c	0.81	-1.1	0.88	1.1	0.00	1.6
A_52_P272119	NM_001081651	Rab42	0.19	1.3	0.12	1.3	0.00	1.6
A_52_P198435	NM_207246	Rasgrp3	0.33	1.2	0.01	1.4	0.00	1.6
A_52_P125467	NM_144865	Reep2	0.32	1.2	0.04	1.3	0.00	1.6
A_55_P2078365	NM_173402	Rgs12	0.73	1.1	0.26	1.2	0.00	1.6
A_52_P89567	NM_007483	Rhob	0.28	1.3	0.15	1.3	0.00	1.6
A_55_P1979684	NM_007484	Rhoc	0.31	1.2	0.17	1.2	0.00	1.6
A_51_P345663	NM_024281	Rrbp1	0.06	1.3	0.00	1.5	0.00	1.6
A_55_P2173982	NM_009104	Rrm2	0.00	3.2	0.00	2.8	0.00	1.6
A_52_P353478	NM_009135	Scn7a	0.87	1.1	0.97	1.0	0.00	1.6
A_51_P436878	NM_018820	Sertad1	0.50	1.2	0.14	1.3	0.00	1.6
A_51_P113403	NM_178743	Slc26a11	0.06	1.3	0.00	1.4	0.00	1.6
A_55_P1953301	NM_146126	Sord	0.53	1.2	0.16	1.3	0.00	1.6
A_55_P2157023	NM_011441	Sox17	1.00	1.0	0.97	1.0	0.04	1.6
A_55_P2094706	NM_011441	Sox17	0.74	1.1	0.90	1.1	0.00	1.6
A_55_P2046812	NM_009242	Sparc	0.94	1.0	1.00	-1.0	0.00	1.6
A_55_P2046807	NM_009242	Sparc	0.95	-1.0	0.71	1.1	0.00	1.6
A_66_P139618	NM_001082545	Stfa2	0.95	1.0	0.48	1.2	0.00	1.6
A_55_P2077188	NM_001113569	Stxbp1	0.56	1.2	0.19	1.2	0.00	1.6
A_55_P2097616	NM_001113569	Stxbp1	0.48	1.2	0.25	1.2	0.00	1.6
A_55_P1985890	NM_178892	Tiparp	0.91	-1.1	0.86	-1.1	0.01	1.6
A_52_P8324	NM_026516	Tmem178	0.61	1.2	0.72	1.1	0.00	1.6
A_55_P1976584	NM_001038710	Tmod2	0.36	1.2	0.06	1.4	0.00	1.6
A_51_P288916	NM_177368	Tmtc2	0.69	1.2	0.75	1.1	0.00	1.6
A_55_P1989296	NM_031176	Tnxb	0.84	1.1	0.94	1.0	0.00	1.6
A_52_P220810	NM_144551	Trib2	0.98	-1.0	0.77	1.1	0.00	1.6
A_51_P341918	NM_009366	Tsc22d1	0.85	1.1	0.23	1.2	0.00	1.6
A_55_P1996573	NM_145607	Ttc13	0.11	1.3	0.16	1.2	0.00	1.6
A_55_P2034864	NM_023716	Tubb2b	0.51	1.2	0.17	1.3	0.00	1.6
A_55_P2124228	NM_028283	Uaca	0.23	1.2	0.01	1.3	0.00	1.6
A_55_P1965827	NM_011664	Ubb	0.11	1.2	0.01	1.3	0.00	1.6
A_51_P433870	NM_009506	Vegfc	0.08	1.5	0.04	1.5	0.01	1.6
A_55_P2012146	BC075649	Veph1	0.22	1.4	0.08	1.4	0.00	1.6
A_55_P2102335	NM_033327	Zfp423	0.54	1.2	0.62	1.1	0.00	1.6
A_30_P01032788			0.03	1.5	0.26	1.2	0.00	1.5
A_51_P483576	AF138742		0.20	1.6	0.00	2.1	0.08	1.5
A_30_P01021307			0.95	1.0	0.96	1.0	0.04	1.5
A_51_P505521	BC019757		0.61	-1.2	0.88	-1.1	0.03	1.5
A_55_P2067362	AK089244		0.74	1.2	0.57	1.2	0.02	1.5
A_30_P01026716			0.58	1.2	0.24	1.3	0.01	1.5
A_30_P01026973			0.61	1.2	0.82	1.1	0.01	1.5
A_66_P136813	AK020048		0.09	1.4	0.78	1.1	0.01	1.5
A_30_P01032267			0.79	1.1	0.86	1.1	0.01	1.5
A_30_P01021251			0.92	1.0	0.66	1.1	0.00	1.5

A_30_P01024850			0.44	1.2	0.06	1.3	0.00	1.5
A_30_P01022991			0.03	1.3	0.00	1.4	0.00	1.5
A_55_P2108693			0.75	1.1	0.25	1.2	0.00	1.5
A_30_P01022914			0.39	1.1	0.44	1.1	0.00	1.5
A_52_P148678	AK169695		0.23	1.2	0.01	1.3	0.00	1.5
A_55_P2161465	AK142593		0.19	1.2	0.06	1.3	0.00	1.5
A_30_P01021453			0.76	1.1	0.52	1.2	0.00	1.5
A_30_P01027037			0.55	1.2	0.25	1.2	0.00	1.5
A_30_P01032293			0.45	1.1	0.18	1.2	0.00	1.5
A_30_P01020914			0.02	1.3	0.00	1.3	0.00	1.5
A_30_P01026510			0.45	1.1	0.39	1.1	0.00	1.5
A_30_P01018970			0.70	1.1	0.31	1.2	0.00	1.5
A_30_P01025417			0.07	1.4	0.08	1.4	0.00	1.5
A_30_P01021640			0.40	1.2	0.11	1.2	0.00	1.5
A_55_P2059765	AK087349		0.21	1.3	0.01	1.4	0.00	1.5
A_30_P01023499			0.02	1.4	0.04	1.4	0.00	1.5
A_30_P01028912			0.78	1.1	0.05	1.3	0.00	1.5
A_30_P01027810			0.91	1.0	0.03	1.3	0.00	1.5
A_55_P2112085	NM_027906	1300010F03Rik	0.79	1.1	0.19	1.2	0.00	1.5
A_55_P2146590	NM_026931	1810011O10Rik	0.94	-1.0	0.85	-1.1	0.00	1.5
A_55_P2383283	AK036632	2310001H17Rik	0.37	1.2	0.00	1.4	0.00	1.5
A_51_P183213	NM_025506	2310007A19Rik	0.09	1.5	0.30	1.3	0.01	1.5
A_51_P511707	NM_025636	2310079N02Rik	0.82	1.1	0.22	1.3	0.00	1.5
A_65_P09285	NM_181075	2610524H06Rik	0.82	1.1	0.08	1.2	0.00	1.5
A_55_P1960049	NM_027419	2810408A11Rik	0.01	1.2	0.07	1.2	0.00	1.5
A_55_P1981664	NM_033144	41160.0	0.02	1.2	0.01	1.3	0.00	1.5
A_51_P276479	NM_178098	4930486L24Rik	0.24	1.3	0.04	1.3	0.00	1.5
A_55_P2098210	NM_172741	4931406P16Rik	0.59	1.1	0.00	1.4	0.00	1.5
A_55_P2349148	AK017909	5830408B19Rik	0.30	1.3	0.26	1.3	0.01	1.5
A_55_P1956457	NM_029998	6030458C11Rik	0.34	1.2	0.11	1.2	0.00	1.5
A_55_P2470474	AK087205	9530082P21Rik	0.63	1.2	0.49	1.2	0.01	1.5
A_55_P2042156	NM_007377	Aatk	0.24	1.3	0.18	1.3	0.00	1.5
A_51_P249118	NM_008830	Abcb4	0.60	1.2	0.15	1.3	0.00	1.5
A_51_P185906	NM_025659	Abi3	0.30	1.3	0.39	1.2	0.01	1.5
A_55_P2077009	NM_207223	Acap3	0.05	1.3	0.15	1.2	0.00	1.5
A_51_P382789	NM_027025	Adora3	0.47	1.2	0.07	1.3	0.00	1.5
A_51_P247249	NM_009662	Alox5	0.84	1.1	0.63	1.2	0.04	1.5
A_55_P1962039	NM_009674	Anxa7	0.24	1.2	0.00	1.3	0.00	1.5
A_51_P399889	NM_026193	Ap4b1	0.02	1.3	0.00	1.4	0.00	1.5
A_55_P2122709	NM_027871	Arhgef3	0.94	1.0	0.21	1.2	0.00	1.5
A_51_P259975	NM_023113	Aspa	0.83	-1.1	0.97	1.0	0.01	1.5
A_55_P2004781	NM_007494	Ass1	0.62	1.1	0.26	1.2	0.00	1.5
A_55_P1987261	NM_172691	B230312A22Rik	0.16	1.2	0.09	1.2	0.00	1.5
A_55_P2110758	NM_177083	B430306N03Rik	0.88	1.1	0.81	1.1	0.02	1.5
A_55_P2170732	NM_178699	B930041F14Rik	0.98	1.0	0.06	1.3	0.00	1.5
A_55_P2157033	NM_019517	Bace2	0.72	1.1	0.36	1.2	0.01	1.5
A_55_P2015984	NM_145511	BC003331	0.02	1.3	0.00	1.4	0.00	1.5
A_55_P2132403	NM_198170	BC059842	0.29	1.2	0.07	1.3	0.00	1.5
A_66_P105175	NM_009738	Bche	0.78	1.1	0.53	1.2	0.03	1.5

A_51_P237668	NM_009749	Bex2	0.87	1.1	0.24	1.3	0.00	1.5
A_55_P2003903	NM_009751	Bfsp1	0.31	1.2	0.19	1.3	0.00	1.5
A_51_P272553	NM_011498	Bhlhe40	0.60	1.2	0.06	1.4	0.00	1.5
A_55_P1983773	NM_001012273	Birc5	0.00	4.0	0.00	3.0	0.02	1.5
A_55_P2355330	NM_145613	C1qtnf5	0.17	1.2	0.32	1.2	0.00	1.5
A_51_P384318	NM_023143	C1ra	0.93	1.1	0.99	1.0	0.01	1.5
A_51_P208931	NM_145974	C330016O10Rik	0.99	-1.0	0.44	1.1	0.00	1.5
A_55_P2020341	NM_178597	Camk2g	0.14	1.2	0.02	1.3	0.00	1.5
A_52_P683441	NM_007602	Capn5	0.87	1.1	0.24	1.3	0.01	1.5
A_55_P2080163	NM_025821	Carhsp1	0.01	1.3	0.00	1.3	0.00	1.5
A_51_P255304	NM_007617	Cav3	0.10	1.4	0.87	1.1	0.01	1.5
A_51_P238448	NM_007632	Ccnd3	0.33	1.2	0.02	1.3	0.00	1.5
A_51_P291501	NM_001081062	Ccno	0.42	1.2	0.37	1.2	0.01	1.5
A_51_P285206	NM_013487	Cd3d	0.73	1.1	0.05	1.4	0.00	1.5
A_51_P342652	NM_008339	Cd79b	0.44	1.3	0.03	1.7	0.08	1.5
A_65_P05252	NM_007658	Cdc25a	0.11	1.3	0.01	1.4	0.00	1.5
A_51_P513224	NM_001081373	Cep164	0.91	1.0	0.54	1.1	0.00	1.5
A_55_P2175190	NM_001129999	Cep250	0.16	1.2	0.01	1.3	0.00	1.5
A_51_P179919	NM_172759	Ces5	0.10	1.3	0.04	1.3	0.00	1.5
A_51_P408363	NM_008823	Cfp	0.50	1.2	0.59	1.1	0.00	1.5
A_65_P15875	NM_133913	Chpf2	0.79	1.1	0.83	1.1	0.00	1.5
A_52_P390944	NM_016803	Chst3	0.04	1.4	0.00	1.6	0.00	1.5
A_51_P515605	NM_009930	Col3a1	0.20	1.4	1.00	-1.0	0.01	1.5
A_51_P124254	NM_009931	Col4a1	0.56	1.2	0.69	1.1	0.00	1.5
A_51_P353592	NM_025417	Commd4	0.07	1.3	0.00	1.4	0.00	1.5
A_52_P366525	NM_001039710	Coq10b	0.92	1.0	0.52	1.2	0.00	1.5
A_55_P2087607	NM_001042611	Cp	0.59	1.1	0.37	1.2	0.00	1.5
A_55_P2021285	NM_146067	Cpped1	0.40	1.1	0.01	1.3	0.00	1.5
A_55_P1971729	NM_007758	Cr2	0.38	1.4	0.03	1.8	0.07	1.5
A_55_P1963150	NM_011804	Creg1	0.76	1.1	0.15	1.4	0.03	1.5
A_52_P460957	NM_013498	Crem	0.68	1.1	0.16	1.2	0.00	1.5
A_55_P2018847	NM_001164735	Crlf2	0.80	1.1	0.82	1.1	0.00	1.5
A_55_P1977473	NM_023118	Dab2	0.88	1.1	0.59	1.1	0.00	1.5
A_51_P422208	NM_007875	Dpagt1	0.13	1.2	0.00	1.3	0.00	1.5
A_55_P1998001	NM_001025384	DXBay18	0.70	1.1	0.05	1.3	0.00	1.5
A_55_P2142439	NM_026728	Echdc2	0.28	1.2	0.16	1.2	0.00	1.5
A_51_P160544	NM_021474	Efemp2	0.48	1.1	0.27	1.1	0.00	1.5
A_51_P360918	NM_020578	Ehd3	0.81	1.1	0.34	1.2	0.00	1.5
A_51_P455807	NM_133838	Ehd4	0.48	1.2	0.01	1.5	0.00	1.5
A_55_P1956488	NM_013514	Epb4.9	0.83	1.1	0.63	1.1	0.00	1.5
A_51_P103594	NM_029894	Fam162b	0.81	1.1	0.62	1.2	0.01	1.5
A_55_P2007389	NM_001014995	Fam189b	0.65	1.1	0.03	1.3	0.00	1.5
A_55_P2150343	NM_001037298	Fam38a	0.18	1.2	0.14	1.2	0.00	1.5
A_55_P2102901	NM_172988	Fbxl4	0.79	1.1	0.05	1.3	0.00	1.5
A_52_P503730	NM_199012	Fchsd2	0.77	1.1	0.49	1.1	0.00	1.5
A_55_P1966209	NM_199448	Fez2	0.69	1.1	0.06	1.2	0.00	1.5
A_65_P06147	NM_008010	Fgfr3	0.23	1.3	0.81	1.1	0.00	1.5
A_52_P222350	NM_134080	Flnb	0.21	1.2	0.18	1.2	0.00	1.5
A_51_P381260	NM_008761	Fxyd5	1.00	-1.0	0.70	1.1	0.00	1.5

A_55_P2024431	NM_008064	Gaa	0.61	1.3	0.26	1.4	0.04	1.5
A_55_P2096867	NM_008083	Gap43	0.72	1.1	0.59	1.2	0.01	1.5
A_55_P2022158	NM_008096	Gc	0.81	-1.1	0.98	-1.0	0.02	1.5
A_52_P116006	NM_010266	Gda	0.60	1.1	0.58	1.1	0.00	1.5
A_55_P2009861	XM_001472138	Gm2015	0.83	-1.1	0.85	-1.1	0.03	1.5
A_55_P2035326	XM_001473557	Gm2433	0.90	1.0	0.14	1.2	0.00	1.5
A_55_P2114318	XM_001478955	Gm4080	0.82	-1.1	0.93	-1.0	0.02	1.5
A_55_P1976744	XM_001478262	Gm4324	0.01	1.6	0.00	1.6	0.01	1.5
A_55_P2133370	XM_001479361	Gm4610	0.32	1.2	0.29	1.2	0.00	1.5
A_55_P2054098	XM_001475988	Gm9495	0.11	1.3	0.18	1.2	0.00	1.5
A_52_P920129	NM_010305	Gnai1	0.94	-1.0	0.85	1.1	0.02	1.5
A_55_P2027392	NM_030258	Gpr146	0.77	1.1	0.32	1.2	0.01	1.5
A_55_P2004761	NM_001033978	H2-Eb2	0.23	1.3	0.01	1.6	0.01	1.5
A_55_P2082929	NM_010389	H2-Ob	0.18	1.5	0.02	1.7	0.01	1.5
A_51_P450021	NM_025933	Higd2a	0.85	1.1	0.19	1.2	0.00	1.5
A_51_P322972	NM_145419	Hkdc1	0.94	-1.1	0.60	1.2	0.03	1.5
A_55_P2112295	NM_010440	Hmg20b	0.06	1.2	0.02	1.3	0.00	1.5
A_55_P2116993	NM_016690	Hnrpdl	0.00	1.5	0.01	1.3	0.00	1.5
A_51_P466613	NM_011984	Homer3	0.04	1.3	0.02	1.3	0.00	1.5
A_52_P624149	NM_011631	Hsp90b1	0.59	1.2	0.04	1.4	0.00	1.5
A_66_P120995	NM_172812	Htr2a	0.51	1.2	0.38	1.2	0.00	1.5
A_55_P2045136	XM_001477102	I830127L07Rik	0.37	1.2	0.47	1.1	0.00	1.5
A_51_P196695	NM_008372	Il7r	0.81	1.1	0.23	1.3	0.00	1.5
A_52_P281145	NM_172872	Kank4	0.00	1.7	0.00	2.0	0.00	1.5
A_55_P2055189	NM_001081134	Kcng1	0.72	1.1	0.97	1.0	0.00	1.5
A_52_P268713	NM_010600	Kcnh1	0.63	1.2	0.21	1.3	0.00	1.5
A_55_P2074499	NM_001029985	Kcp	0.76	1.1	0.36	1.2	0.01	1.5
A_55_P1981949	NM_175174	Klhl5	0.47	1.2	0.04	1.4	0.00	1.5
A_55_P2084965	NM_001039042	Klk13	0.05	1.6	0.53	1.2	0.03	1.5
A_55_P2051834	NM_001033534	Layn	0.45	1.1	0.06	1.2	0.00	1.5
A_52_P569549	NM_176953	Lig4	0.36	1.2	0.03	1.3	0.00	1.5
A_55_P2102419	NM_023684	Lime1	0.00	1.5	0.04	1.3	0.00	1.5
A_55_P1974452	NM_023684	Lime1	0.00	1.6	0.00	1.4	0.00	1.5
A_51_P324651	NM_181039	Lphn1	0.47	1.2	0.35	1.2	0.00	1.5
A_51_P116487	NM_015816	Lsm4	0.92	1.0	0.39	1.2	0.00	1.5
A_55_P1970105	NM_013589	Ltbp2	0.51	1.2	0.13	1.3	0.00	1.5
A_55_P1986306	NM_181470	Ltv1	0.36	1.2	0.00	1.4	0.00	1.5
A_51_P265495	NM_010738	Ly6a	0.47	1.2	0.21	1.2	0.00	1.5
A_51_P521052	NM_029627	Ly6k	0.51	1.3	0.08	1.6	0.04	1.5
A_55_P2102175	NM_025808	Lztr1	0.16	1.2	0.01	1.3	0.00	1.5
A_51_P451458	NM_174857	Mamdc2	0.47	1.2	0.47	1.2	0.01	1.5
A_55_P2142580	AK146784	Mark4	0.88	1.1	0.89	1.0	0.00	1.5
A_66_P107379	NM_010784	Mdk	0.44	1.2	0.82	1.1	0.00	1.5
A_55_P2044439	NM_008595	Mfng	0.47	1.2	0.07	1.3	0.00	1.5
A_51_P253803	NM_001081117	Mki67	0.00	4.1	0.00	3.0	0.01	1.5
A_55_P2073377	NM_001081117	Mki67	0.00	4.1	0.00	3.0	0.01	1.5
A_55_P2046802	NM_029005	Mlkl	0.81	1.1	0.84	1.1	0.00	1.5
A_52_P322421	NM_007962	Mpzl2	0.71	1.2	0.91	1.1	0.02	1.5
A_52_P607128	NM_031195	Msr1	0.79	1.1	0.98	1.0	0.02	1.5

A_55_P2157966	NM_032393	Mtap1a	1.00	1.0	0.93	1.0	0.00	1.5
A_51_P479230	NM_023455	Nat8	0.77	1.1	0.25	1.3	0.01	1.5
A_55_P2165334	NM_023317	Nde1	0.00	1.4	0.00	1.6	0.00	1.5
A_51_P234692	NR_003513	Neat1	0.00	2.0	0.29	1.3	0.01	1.5
A_55_P2157770	NM_001160165	Neu2	0.06	1.4	0.12	1.3	0.00	1.5
A_51_P515965	NM_008685	Nfe2	0.29	1.3	0.27	1.3	0.00	1.5
A_66_P129564	NM_022414	Ngb	0.87	1.1	0.90	1.1	0.01	1.5
A_51_P405227	NM_026012	Nradd	0.30	1.2	0.02	1.3	0.00	1.5
A_51_P504354	NM_025980	Nrarp	0.01	1.6	0.02	1.5	0.01	1.5
A_52_P467726	NM_010942	Nsg1	0.65	1.2	0.04	1.5	0.00	1.5
A_51_P163953	NM_008741	Nsg2	0.28	1.3	0.10	1.3	0.00	1.5
A_51_P191782	NM_133859	Olfml3	0.42	1.2	0.28	1.2	0.00	1.5
A_51_P506733	NM_001038845	P2rx7	0.75	1.1	0.50	1.2	0.00	1.5
A_51_P374900	NM_028808	P2ry13	0.84	1.1	0.62	1.2	0.02	1.5
A_55_P2049752	NM_173749	Pamr1	0.52	1.3	0.62	1.2	0.03	1.5
A_55_P1958517	NM_029922	Parp6	0.00	1.3	0.01	1.3	0.00	1.5
A_52_P278295	NM_028460	Pear1	0.45	1.2	0.11	1.3	0.00	1.5
A_55_P2054409	NM_011089	Pira2	0.82	1.1	0.65	1.2	0.04	1.5
A_51_P232281	NM_011109	Pla2g2d	0.03	1.3	0.00	1.4	0.00	1.5
A_52_P681310	NM_011113	Plaur	0.98	1.0	0.99	1.0	0.00	1.5
A_52_P327588	NM_148937	Plcd4	0.83	1.1	0.20	1.3	0.01	1.5
A_55_P2139027	NM_001163540	Plec1	0.90	1.0	0.93	1.0	0.00	1.5
A_55_P1985219	NM_001122818	Pnpla6	0.22	1.2	0.16	1.2	0.00	1.5
A_51_P169516	NM_001085501	Ppp1r3d	0.29	1.2	0.15	1.2	0.00	1.5
A_52_P487362	NM_028980	Ppp4r4	0.25	1.4	0.57	1.2	0.00	1.5
A_55_P2110567	NM_001045516	Proca1	0.31	1.2	0.04	1.4	0.00	1.5
A_51_P257065	NM_018786	Prpf40b	0.00	1.3	0.00	1.5	0.00	1.5
A_51_P477682	NM_008939	Prss12	0.19	1.4	0.07	1.5	0.02	1.5
A_51_P194609	NM_178372	Prss34	0.72	1.1	0.32	1.3	0.02	1.5
A_51_P233603	NM_011969	Psma7	0.04	1.2	0.00	1.3	0.00	1.5
A_52_P574190	NM_025894	Psmd12	0.28	1.2	0.07	1.3	0.00	1.5
A_55_P2429225	NM_019976	Psrc1	0.02	1.4	0.05	1.3	0.00	1.5
A_51_P311904	NM_172498	Ptk2b	0.31	1.2	0.06	1.3	0.00	1.5
A_55_P2007871	NM_207232	Ptpdc1	0.07	1.2	0.00	1.3	0.00	1.5
A_55_P2006327	NM_008981	Ptprg	0.98	1.0	0.61	1.2	0.00	1.5
A_51_P371091	NM_178593	Rcsd1	0.77	1.1	0.60	1.1	0.00	1.5
A_66_P138289	NM_134006	Rdh5	0.03	1.3	0.05	1.2	0.00	1.5
A_51_P249594	NM_019570	Rev1	0.02	1.3	0.03	1.3	0.00	1.5
A_52_P69194	NM_177572	Rimkla	0.68	1.1	0.49	1.2	0.00	1.5
A_51_P491987	NM_019955	Ripk3	0.02	1.5	0.15	1.3	0.00	1.5
A_51_P244969	NM_025346	Rmnd5b	0.31	1.2	0.11	1.2	0.00	1.5
A_51_P474454	NM_026001	Rnaseh2b	0.00	1.5	0.01	1.5	0.00	1.5
A_55_P2166262	NM_027859	Rnf215	0.90	-1.1	0.26	1.3	0.02	1.5
A_55_P2083919	NM_175549	Robo2	0.82	1.1	0.06	1.3	0.00	1.5
A_55_P2031471	NM_001034060	Rufy4	0.97	1.0	0.93	1.1	0.04	1.5
A_55_P1998471	NM_009114	S100a9	0.75	1.1	0.16	1.3	0.01	1.5
A_55_P1994807	NM_011314	Saa2	0.95	-1.0	0.93	1.0	0.01	1.5
A_55_P2083023	NM_133678	Sac3d1	0.04	1.3	0.02	1.3	0.00	1.5
A_55_P2010152	NM_001164059	Sell	0.87	1.1	0.21	1.3	0.01	1.5

A_52_P410765	NM_011352	Sema7a	0.34	-1.2	0.57	-1.1	0.00	1.5
A_51_P511949	NM_080793	Setd7	0.44	1.2	0.09	1.3	0.00	1.5
A_55_P2157872	NM_001079695	Sfrs5	0.11	1.4	0.02	1.4	0.00	1.5
A_55_P2043554	NM_009195	Slc12a4	0.13	1.2	0.05	1.2	0.00	1.5
A_55_P2115582	NM_015747	Slc20a1	0.04	1.3	0.01	1.3	0.00	1.5
A_55_P2153743	NM_134420	Slc26a6	0.12	1.2	0.10	1.2	0.00	1.5
A_55_P2048119	NM_146257	Slc29a4	0.81	1.1	0.56	1.1	0.00	1.5
A_52_P578768	NM_178746	Slc38a9	0.91	-1.0	0.08	1.3	0.00	1.5
A_51_P196972	NM_011403	Slc4a1	0.35	1.4	0.43	1.3	0.03	1.5
A_51_P230287	NM_030889	Sorcs2	0.45	1.2	0.08	1.3	0.00	1.5
A_55_P1974028	NM_138673	Stab2	0.35	1.3	0.20	1.3	0.00	1.5
A_52_P681488	NM_026343	Stx17	0.22	1.3	0.31	1.2	0.00	1.5
A_55_P1993109	NM_009315	Taf6	0.37	1.2	0.09	1.2	0.00	1.5
A_55_P2059090	NM_145968	Tagap	0.01	1.3	0.02	1.3	0.00	1.5
A_55_P2095360	NM_011576	Tfpi	0.69	1.1	0.69	1.1	0.00	1.5
A_66_P132295	NM_011576	Tfpi	0.75	1.1	0.39	1.2	0.00	1.5
A_51_P427516	NM_019576	Thsd1	0.37	1.2	0.38	1.2	0.00	1.5
A_55_P2308488	NM_009384	Tiam1	0.34	1.2	0.30	1.2	0.00	1.5
A_55_P2081615	NM_001164081	Timeless	0.10	1.2	0.00	1.5	0.00	1.5
A_51_P514961	NM_178892	Tiparp	0.92	-1.1	0.82	-1.1	0.01	1.5
A_51_P401683	NM_145375	Tm6sf1	0.10	1.4	0.04	1.4	0.01	1.5
A_55_P1955078	NM_145580	Tmem149	0.00	1.5	0.01	1.4	0.00	1.5
A_55_P2058348	NM_025864	Tmem206	0.64	1.1	0.31	1.2	0.00	1.5
A_51_P368496	NM_029537	Tmem98	0.90	1.0	0.64	1.1	0.00	1.5
A_55_P1975645	NM_198967	Tmtc1	0.27	1.2	0.15	1.2	0.00	1.5
A_55_P1962937	NM_031254	Trem2	0.99	1.0	0.94	1.0	0.01	1.5
A_52_P457967	NM_172622	Trerf1	0.81	1.1	0.62	1.1	0.00	1.5
A_55_P1962359	NM_053168	Trim11	0.03	1.3	0.39	1.1	0.00	1.5
A_52_P325477	NM_053169	Trim16	0.23	1.3	0.05	1.4	0.00	1.5
A_55_P2110235	NM_133975	Trip12	0.84	1.1	0.00	1.4	0.00	1.5
A_55_P1966644	NM_013838	Trpc6	0.08	1.3	0.18	1.2	0.00	1.5
A_55_P2160686	NM_009366	Tsc22d1	0.55	1.1	0.07	1.2	0.00	1.5
A_52_P144297	NM_198617	Tspyl3	0.65	1.2	0.20	1.3	0.00	1.5
A_55_P2062836	NM_001013026	Ttf2	0.01	1.6	0.00	1.5	0.00	1.5
A_51_P421140	NM_026473	Tubb6	0.00	1.8	0.02	1.5	0.00	1.5
A_52_P123354	NM_019742	Tusc2	0.94	1.0	0.22	1.2	0.00	1.5
A_52_P613596	NM_020505	Vav3	0.31	1.2	0.49	1.1	0.00	1.5
A_51_P392687	NM_011701	Vim	0.42	1.2	0.33	1.2	0.00	1.5
A_55_P1970120	NM_146168	Vopp1	0.30	1.2	0.01	1.3	0.00	1.5
A_51_P194498	NM_023395	Wfdc1	0.59	1.2	0.04	1.5	0.01	1.5
A_51_P316951	NM_001167860	Wipf3	0.15	1.4	0.89	1.1	0.02	1.5
A_55_P1997275	NM_133216	Xpnpep1	0.40	1.2	0.15	1.3	0.00	1.5
A_66_P137152	NM_025970	Zbtb8os	0.34	1.2	0.01	1.4	0.00	1.5
A_65_P19933	AK137472	Zdhhc23	0.16	1.3	0.56	1.1	0.00	1.5
A_51_P353095	NM_177319	Zfyve27	0.35	1.2	0.04	1.3	0.00	1.5
A_55_P2044252	NM_001164597	Znf512b	0.02	1.2	0.01	1.3	0.00	1.5
A_55_P1991440	NM_011757	Zscan21	0.15	1.2	0.00	1.4	0.00	1.5
A_30_P01020135			0.00	1.8	0.43	1.2	0.07	1.4
A_30_P01019123			0.00	1.8	0.00	2.0	0.04	1.4

A_30_P01025798			0.00	1.9	0.62	1.2	0.07	1.4
A_30_P01025102			0.00	2.0	0.01	1.5	0.03	1.4
A_55_P2145224	AF290571		0.19	1.5	0.00	2.0	0.13	1.4
A_55_P2057283	AY170502		0.14	1.6	0.00	2.0	0.11	1.4
A_55_P2340593	AK007545	1810019D21Rik	0.00	1.5	0.04	1.3	0.00	1.4
A_55_P2126448	NR_027821	1810032O08Rik	0.00	1.7	0.02	1.3	0.00	1.4
A_55_P2372228	DT902180	A430104N18Rik	0.02	1.5	0.30	1.2	0.01	1.4
A_51_P330213	NM_024184	Asf1b	0.00	2.0	0.00	1.7	0.03	1.4
A_52_P338956	NM_001081169	Aspg	0.02	1.9	0.42	1.3	0.14	1.4
A_51_P298802	NM_001002896	Bfsp2	0.00	1.5	0.00	1.6	0.01	1.4
A_55_P2004711	NM_177088	Ccdc45	0.00	1.6	0.00	1.4	0.00	1.4
A_51_P164014	NM_173762	Cenpe	0.00	3.2	0.00	2.2	0.03	1.4
A_51_P367310	NM_028083	Chaf1b	0.02	1.5	0.00	1.6	0.01	1.4
A_55_P2085181	NM_028083	Chaf1b	0.00	1.5	0.00	1.6	0.01	1.4
A_55_P1955656	NM_007796	Ctla2a	0.06	1.3	0.00	1.5	0.00	1.4
A_51_P391955	NM_029723	Dapl1	0.04	2.3	0.22	1.7	0.40	1.4
A_55_P2062598	NM_178609	E2f7	0.00	2.0	0.00	1.8	0.01	1.4
A_51_P407879	NM_145382	Fam193b	0.00	1.6	0.02	1.3	0.00	1.4
A_55_P2110245	NM_001163359	Fignl1	0.00	1.9	0.00	2.1	0.03	1.4
A_55_P2011937	NM_001163359	Fignl1	0.00	2.0	0.00	2.0	0.03	1.4
A_51_P153423	NM_001081416	Fndc1	0.39	-1.3	0.00	-1.7	0.02	1.4
A_51_P462102	NM_133911	Gpr125	0.15	1.3	0.00	1.6	0.01	1.4
A_51_P388819	NM_174998	Hpcal4	0.37	1.3	0.00	1.7	0.02	1.4
A_55_P2125786	NM_028175	Lrrc8e	0.23	1.3	0.01	1.5	0.01	1.4
A_51_P158210	NM_008564	Mcm2	0.02	1.5	0.00	1.9	0.01	1.4
A_51_P324934	AK088142	Mcm3	0.00	1.6	0.00	1.6	0.01	1.4
A_51_P165704	NM_008568	Mcm7	0.00	1.8	0.00	1.7	0.02	1.4
A_55_P2076866	NM_001162977	Megf6	0.02	1.5	0.00	1.6	0.01	1.4
A_51_P247184	NM_008728	Npr3	0.20	1.4	0.00	1.9	0.04	1.4
A_55_P2105002	NM_011045	Pcna	0.00	1.5	0.00	1.6	0.00	1.4
A_55_P2053958	NM_030566	Rabep2	0.00	1.5	0.01	1.4	0.00	1.4
A_51_P148105	NM_011234	Rad51	0.00	1.8	0.00	1.9	0.01	1.4
A_55_P1991688	NM_009013	Rad51ap1	0.03	1.4	0.00	1.6	0.01	1.4
A_55_P2032232	NM_011268	Rgs9	0.00	1.5	0.00	1.5	0.00	1.4
A_55_P2137431	NM_001001882	Rtel1	0.00	1.5	0.01	1.3	0.00	1.4
A_52_P38964	NM_001081962	Sap25	0.01	1.5	0.41	1.2	0.00	1.4
A_51_P112627	NM_009180	St6galnac2	0.13	1.3	0.02	1.5	0.00	1.4
A_51_P295420	NM_145931	Zc3h7a	0.01	1.5	0.00	1.4	0.00	1.4
A_30_P01028297			0.00	1.5	0.11	1.2	0.03	1.3
A_30_P01030198			0.01	1.6	0.22	1.3	0.11	1.3
A_30_P01028362			0.00	1.6	0.24	1.2	0.05	1.3
A_30_P01029720			0.01	1.7	0.70	1.1	0.26	1.3
A_30_P01023251			0.00	1.8	0.40	1.2	0.08	1.3
A_51_P488554	NM_026543	3010026O09Rik	0.02	1.4	0.00	1.5	0.02	1.3
A_55_P2063336	NM_134041	4930427A07Rik	0.00	1.7	0.00	1.7	0.07	1.3
A_55_P2293351	AK082896	C430010C01	0.00	1.7	0.12	1.2	0.02	1.3
A_51_P481920	NM_009828	Ccna2	0.00	3.5	0.00	2.7	0.05	1.3
A_51_P196973	NM_013733	Chaf1a	0.34	1.3	0.01	1.5	0.05	1.3
A_52_P162099	NM_001004140	Ckap2	0.00	3.3	0.00	2.6	0.10	1.3

A_51_P227004	NM_016904	Cks1b	0.00	1.8	0.00	1.5	0.08	1.3
A_55_P2033110	NM_009639	Crisp3	0.01	2.6	0.22	1.7	0.49	1.3
A_51_P252859	NM_010516	Cyr61	0.16	1.4	0.01	1.6	0.14	1.3
A_55_P2135551	NM_025718	Dnase1l2	0.01	2.0	0.52	1.2	0.29	1.3
A_55_P2164534	NM_029766	Dtl	0.00	1.6	0.00	1.7	0.03	1.3
A_51_P239984	NM_012012	Exo1	0.00	1.4	0.00	1.5	0.00	1.3
A_66_P130916	NM_010389	H2-Ob	0.21	1.4	0.04	1.5	0.16	1.3
A_51_P133137	NM_009004	Kif20a	0.00	2.6	0.00	2.2	0.06	1.3
A_55_P2109717	NM_183046	Kif20b	0.00	2.1	0.00	1.9	0.07	1.3
A_55_P2056654	NM_145588	Kif22	0.00	2.3	0.00	2.0	0.02	1.3
A_51_P130079	NM_008511	Lrmp	0.15	1.3	0.01	1.5	0.01	1.3
A_55_P1955015	NM_178440	Myo1g	0.03	1.4	0.01	1.5	0.02	1.3
A_51_P379478	NM_172484	Nckap5	0.03	1.5	0.01	1.5	0.10	1.3
A_51_P223709	NM_080850	Pask	0.02	1.4	0.00	1.5	0.05	1.3
A_51_P204442	NM_028716	Phf19	0.01	1.6	0.04	1.4	0.18	1.3
A_51_P168632	NM_008921	Prim1	0.01	1.6	0.00	1.5	0.07	1.3
A_55_P1969032	NM_011268	Rgs9	0.01	1.5	0.00	1.6	0.01	1.3
A_55_P1983450	NM_011311	S100a4	0.02	1.5	0.00	1.6	0.08	1.3
A_55_P2004801	NM_001040435	Tacc3	0.00	2.1	0.00	1.6	0.06	1.3
A_55_P2152035	NM_001163763	Tcf19	0.00	1.9	0.00	1.8	0.05	1.3
A_51_P302651	NM_176979	Topbp1	0.00	1.5	0.01	1.4	0.01	1.3
A_51_P431996	NM_175494	Zfp367	0.01	1.6	0.00	1.7	0.10	1.3
A_30_P01023567			0.00	1.5	0.34	1.2	0.07	1.2
A_30_P01032180			0.00	1.5	0.34	1.1	0.08	1.2
A_52_P1016836	AV256778		0.00	1.6	0.16	1.3	0.19	1.2
A_55_P1968664			0.00	1.6	0.00	1.4	0.09	1.2
A_55_P1973560			0.00	2.0	0.00	1.5	0.43	1.2
A_30_P01028054			0.92	1.1	0.03	1.5	0.29	1.2
A_30_P01026232			0.84	1.1	0.01	1.5	0.20	1.2
A_51_P295896	NM_028934	4930452B06Rik	0.36	1.2	0.00	1.5	0.05	1.2
A_55_P2435504	NM_176976	5830418K08Rik	0.00	1.5	0.01	1.3	0.01	1.2
A_51_P386304	NM_207678	Ccnl2	0.00	1.5	0.22	1.2	0.09	1.2
A_55_P1996946	NM_023223	Cdc20	0.00	2.1	0.01	1.6	0.44	1.2
A_52_P628067	NM_013538	Cdca3	0.00	1.9	0.01	1.6	0.47	1.2
A_51_P155142	NM_026560	Cdca8	0.00	2.0	0.00	1.6	0.12	1.2
A_51_P133612	NM_026014	Cdt1	0.01	1.6	0.01	1.5	0.23	1.2
A_55_P2106150	NM_021790	Cenpk	0.00	1.6	0.00	1.6	0.18	1.2
A_55_P2077263	NM_021790	Cenpk	0.00	1.6	0.00	1.7	0.09	1.2
A_52_P104824	NM_019670	Diap3	0.02	1.3	0.00	1.5	0.15	1.2
A_66_P137605	NM_173386	E330016A19Rik	0.00	1.7	0.00	1.5	0.15	1.2
A_51_P394802	NM_026640	Fam111a	0.00	1.4	0.00	1.5	0.02	1.2
A_55_P2178378	NR_002840	Gas5	0.02	1.5	0.39	1.2	0.20	1.2
A_55_P2109505	XM_890094	Gm6594	0.02	1.5	0.25	1.2	0.41	1.2
A_55_P2162136	NM_016957	Hmgn2	0.01	1.5	0.04	1.3	0.28	1.2
A_55_P2028496	XM_001472085	Hmgn2l6	0.00	1.6	0.05	1.4	0.18	1.2
A_55_P1978201	NM_016692	Incenp	0.00	1.8	0.00	1.5	0.18	1.2
A_51_P356762	NM_008565	Mcm4	0.01	1.5	0.00	1.5	0.07	1.2
A_51_P190111	NM_008566	Mcm5	0.01	1.5	0.03	1.4	0.31	1.2
A_51_P202074	NM_146171	Ncapd2	0.00	1.8	0.03	1.4	0.32	1.2

A_55_P1997906	NM_172203	Nox1	0.14	1.4	0.00	1.6	0.33	1.2
A_51_P344566	NM_011121	Plk1	0.00	1.5	0.00	1.4	0.06	1.2
A_55_P2007273	NM_011132	Pole	0.02	1.6	0.02	1.5	0.25	1.2
A_55_P2037712	NM_133786	Smc4	0.00	1.6	0.00	1.5	0.08	1.2
A_55_P1995205	NM_011623	Top2a	0.00	1.9	0.00	1.7	0.43	1.2
A_51_P369200	NM_028109	Tpx2	0.00	1.9	0.00	1.8	0.37	1.2
A_30_P01028956			0.00	1.5	0.01	1.4	0.44	1.1
A_30_P01017632			0.00	1.6	0.00	1.5	0.38	1.1
A_30_P01028287			0.00	1.7	0.08	1.3	0.78	1.1
A_30_P01023554			0.00	1.8	0.00	1.5	0.48	1.1
A_55_P2063146			0.00	2.1	0.00	1.7	0.81	1.1
A_55_P2056493			0.02	1.4	0.01	1.5	0.52	1.1
A_52_P354373	XM_001481164	1190002F15Rik	0.00	2.3	0.00	1.8	0.86	1.1
A_55_P2148171	NM_145555	A330049M08Rik	0.00	1.6	0.30	1.2	0.72	1.1
A_55_P1988228	NM_009791	Aspm	0.00	2.1	0.00	1.7	0.67	1.1
A_55_P2186648	NM_007525	Bard1	0.01	1.5	0.04	1.3	0.64	1.1
A_55_P1983768	NM_009689	Birc5	0.00	1.8	0.00	1.7	0.82	1.1
A_51_P457528	NM_007630	Ccnb2	0.00	2.1	0.00	1.7	0.41	1.1
A_55_P2039324	NM_007634	Ccnf	0.00	1.8	0.00	1.4	0.69	1.1
A_51_P125135	NM_026410	Cdca5	0.00	1.9	0.00	1.7	0.63	1.1
A_55_P2061495	NM_016904	Cks1b	0.00	1.6	0.00	1.4	0.30	1.1
A_55_P2115442	NM_053109	Clec2d	0.00	2.0	0.02	1.5	0.73	1.1
A_55_P1976127	NM_007900	Ect2	0.00	1.9	0.01	1.5	0.68	1.1
A_55_P2018904	NM_001034882	Gm5465	0.02	1.5	0.10	1.3	0.66	1.1
A_55_P2103706	XM_485921	Gm5593	0.00	2.0	0.04	1.5	0.70	1.1
A_52_P498208	NM_178183	Hist1h2ak	0.02	1.5	0.03	1.5	0.74	1.1
A_55_P2084631	NM_178184	Hist1h2an	0.01	1.6	0.07	1.3	0.73	1.1
A_55_P1993789	NM_016957	Hmgn2	0.02	1.8	0.27	1.3	0.78	1.1
A_55_P2028054	NM_016692	Incenp	0.00	1.7	0.00	1.4	0.44	1.1
A_52_P588881	NM_001033484	Iqgap3	0.00	2.0	0.00	1.6	0.76	1.1
A_55_P2062543	NM_053173	Kifc1	0.00	1.8	0.00	1.5	0.46	1.1
A_51_P408071	NM_001042421	Kntc1	0.01	1.6	0.00	1.5	0.62	1.1
A_55_P2052834	NM_010734	Lst1	0.16	1.4	0.04	1.5	0.69	1.1
A_55_P2133255	NM_019499	Mad2l1	0.00	1.7	0.01	1.5	0.62	1.1
A_66_P104309	NM_010861	Myl2	0.00	1.7	0.32	1.2	0.61	1.1
A_55_P2154228	NM_178113	Ncapd3	0.00	1.6	0.00	1.4	0.73	1.1
A_52_P529570	NM_198654	Nsl1	0.00	1.4	0.00	1.5	0.42	1.1
A_51_P240453	NM_133851	Nusap1	0.00	2.5	0.00	2.1	0.62	1.1
A_51_P230098	NM_023209	Pbk	0.00	2.5	0.00	2.2	0.57	1.1
A_55_P1988083	NM_145150	Prc1	0.00	2.4	0.00	1.8	0.57	1.1
A_52_P72587	NM_008859	Prkcq	0.00	1.5	0.06	1.3	0.54	1.1
A_51_P234253	NM_145535	Sdcbp2	0.03	1.5	0.58	1.2	0.83	1.1
A_51_P204402	NM_011369	Shcbp1	0.00	2.2	0.00	1.8	0.64	1.1
A_55_P2172274	NM_008017	Smc2	0.00	1.7	0.00	1.5	0.49	1.1
A_55_P2056496	NM_009387	Tk1	0.00	1.5	0.00	1.4	0.56	1.1
A_55_P1976574	NM_016712	Tmod4	0.01	1.6	0.52	1.2	0.46	1.1
A_52_P151320	NM_025566	Tnfaip8l1	0.00	1.7	0.00	1.5	0.37	1.1
A_55_P2030938	NM_025863	Trim59	0.00	2.0	0.00	1.8	0.80	1.1
A_55_P1996941	NM_026785	Ube2c	0.00	1.9	0.01	1.4	0.81	1.1

A_51_P451151	NM_026785	Ube2c	0.00	2.3	0.00	1.8	0.51	1.1
A_30_P01030419			0.00	1.5	0.16	1.2	0.82	1.0
A_30_P01032204			0.16	-1.4	0.03	-1.5	0.96	1.0
A_66_P134542	NM_028390	Anln	0.00	2.1	0.00	1.5	0.98	1.0
A_51_P209327	NM_013912	Apln	0.23	-1.5	0.02	-1.7	0.95	1.0
A_55_P1980636	NM_011497	Aurka	0.00	1.9	0.00	1.5	0.95	1.0
A_55_P2074796	NM_007671	Cdkn2c	0.03	1.5	0.15	1.3	0.86	1.0
A_52_P30989	NM_028222	Cdkn3	0.00	1.9	0.00	1.6	0.90	1.0
A_55_P2158011	NM_026412	D2Ertd750e	0.00	1.8	0.01	1.4	0.91	1.0
A_51_P303749	NM_178683	Depdc1b	0.00	1.6	0.00	1.5	0.89	1.0
A_51_P324287	NM_024245	Kif23	0.00	1.6	0.00	1.3	0.95	1.0
A_51_P373393	NM_033175	Lce3c	0.03	3.2	0.87	1.2	0.97	1.0
A_55_P2013336	NM_010790	Melk	0.00	1.9	0.00	1.5	0.94	1.0
A_52_P190647	NM_016662	Mxd3	0.01	1.6	0.00	1.6	0.93	1.0
A_51_P424810	NM_133762	Ncapg2	0.00	1.6	0.01	1.5	0.99	1.0
A_52_P139650	NM_025581	Ska1	0.00	1.8	0.00	1.7	0.91	1.0
A_51_P513530	NM_017407	Spag5	0.00	1.5	0.08	1.3	0.91	1.0
A_51_P105709	NM_027182	Trip13	0.00	1.5	0.02	1.4	0.83	1.0
A_30_P01018593			0.00	1.6	0.02	1.4	0.93	-1.0
A_30_P01020075			0.00	1.9	0.00	1.5	0.87	-1.0
A_30_P01033296			0.11	-1.5	0.03	-1.6	0.92	-1.0
A_51_P472217	NM_001081085	2010317E24Rik	0.03	1.5	0.45	1.2	0.87	-1.0
A_51_P311038	NM_001024139	Adamts15	0.02	-1.5	0.04	-1.4	0.85	-1.0
A_55_P2065671	NM_172301	Ccnb1	0.01	1.6	0.07	1.4	0.89	-1.0
A_51_P228171	NM_025495	Cenpp	0.02	1.5	0.02	1.4	0.85	-1.0
A_55_P2000833	NM_001013368	E2f8	0.00	1.6	0.01	1.5	0.99	-1.0
A_51_P455897	NM_144526	Fam64a	0.00	3.0	0.00	2.2	0.97	-1.0
A_51_P393958	NM_025995	Fbxo5	0.00	2.0	0.00	1.8	0.98	-1.0
A_55_P2011436	XM_001474074	Gm11223	0.02	1.5	0.01	1.5	0.96	-1.0
A_55_P2112355	XM_980525	Gm7125	0.03	1.6	0.60	1.2	0.91	-1.0
A_51_P151586	NM_010353	Gsg2	0.00	1.8	0.04	1.3	0.85	-1.0
A_55_P2076048	NM_023284	Nuf2	0.00	1.7	0.01	1.5	0.89	-1.0
A_55_P2127702	NM_012025	Racgap1	0.00	1.7	0.02	1.4	0.84	-1.0
A_51_P487999	NM_028232	Sgol1	0.00	2.4	0.00	1.8	0.97	-1.0
A_55_P2043862	NM_019641	Stmn1	0.00	1.5	0.00	1.4	0.97	-1.0
A_55_P2187034			0.01	1.5	0.04	1.4	0.70	-1.1
A_30_P01028673			0.00	1.6	0.02	1.4	0.79	-1.1
A_30_P01032715			0.08	-1.5	0.02	-1.5	0.83	-1.1
A_52_P56397	NM_001081099	2610002D18Rik	0.01	1.5	0.01	1.4	0.82	-1.1
A_51_P430423	NM_007398	Ada	0.01	4.2	0.77	1.3	0.94	-1.1
A_52_P627269	NM_198171	BC015286	0.01	2.0	0.00	2.0	0.81	-1.1
A_55_P2048588	NM_007659	Cdk1	0.00	1.5	0.15	1.2	0.29	-1.1
A_52_P399584	NM_181589	Ckap2l	0.01	1.6	0.06	1.3	0.77	-1.1
A_55_P2131766	NM_023665	D4Wsu53e	0.00	2.6	0.56	1.3	0.93	-1.1
A_55_P1974347	NM_172513	Fam126b	0.02	-1.5	0.25	-1.2	0.73	-1.1
A_55_P2067342	XM_001478579	Gm9782	0.03	3.3	0.93	1.1	0.89	-1.1
A_66_P101930	NM_025501	Lce3b	0.02	3.3	0.93	1.1	0.88	-1.1
A_51_P120717	NM_010721	Lmnb1	0.04	1.6	0.43	1.2	0.77	-1.1
A_51_P466229	NM_026840	Pdgfrl	0.07	-1.4	0.02	-1.5	0.82	-1.1

A_51_P419226	NM_025393	S100a14	0.02	1.9	0.44	1.3	0.87	-1.1
A_55_P2003746	NM_009126	Serpinb3a	0.04	3.7	0.93	1.1	0.95	-1.1
A_55_P2068663	NM_019641	Stmn1	0.04	1.5	0.06	1.4	0.59	-1.1
A_55_P2000454	XM_001478824	Tspan18	0.15	-1.3	0.02	-1.5	0.67	-1.1
A_30_P01020936			0.03	-2.0	0.20	-1.5	0.55	-1.2
A_30_P01022742			0.40	-1.3	0.04	-1.5	0.27	-1.2
A_55_P1978316	NM_001081127	Adamts14	0.25	-1.3	0.01	-1.5	0.16	-1.2
A_66_P121459	NM_007681	Cenpa	0.00	1.5	0.02	1.3	0.20	-1.2
A_55_P1969131	NM_178373	Cidec	0.22	-1.5	0.03	-1.7	0.55	-1.2
A_55_P1994939	NM_008252	Hmgb2	0.01	1.5	0.19	1.2	0.16	-1.2
A_55_P2152472	NM_001039594	Lce3a	0.04	3.8	1.00	1.0	0.84	-1.2
A_55_P1961715	NM_080637	Nme5	0.02	-1.9	0.19	-1.5	0.49	-1.2
A_51_P350453	NM_013743	Pdk4	0.31	-1.4	0.02	-1.7	0.54	-1.2
A_51_P244497	NM_175640	Plin1	0.36	-1.7	0.03	-2.2	0.75	-1.2
A_30_P01020926			0.02	-1.6	0.05	-1.4	0.05	-1.3
A_30_P01019384			0.01	-1.5	0.00	-1.5	0.06	-1.3
A_30_P01027083			0.00	-1.5	0.00	-1.5	0.00	-1.3
A_55_P2022870	XM_888730		0.00	-1.5	0.00	-1.5	0.02	-1.3
A_55_P2130408	XM_001478191		0.23	-1.3	0.01	-1.5	0.02	-1.3
A_30_P01033196			0.01	-1.3	0.00	-1.5	0.00	-1.3
A_55_P2178137	NM_011889	41155.0	0.02	-1.5	0.00	-1.5	0.04	-1.3
A_55_P2061620	NM_023850	Chst1	0.45	-1.3	0.01	-1.7	0.22	-1.3
A_55_P2444965	NM_175518	D730040F13Rik	0.00	-2.1	0.23	-1.4	0.35	-1.3
A_51_P270949	NM_020034	Hist1h1b	0.01	1.7	0.42	1.2	0.16	-1.3
A_55_P2244142	BC030682	Klf13	0.25	-1.3	0.03	-1.5	0.07	-1.3
A_55_P1974984	XM_001474652	LOC100045638	0.19	-1.5	0.03	-1.6	0.25	-1.3
A_55_P2098603	XM_001480211	LOC100048353	0.01	-1.5	0.13	-1.3	0.11	-1.3
A_55_P2105542	NM_001143848	Pde2a	0.23	-1.3	0.02	-1.5	0.06	-1.3
A_51_P224311	NM_022318	Popdc2	0.49	-1.2	0.00	-1.5	0.03	-1.3
A_51_P438619	NM_175407	Sobp	0.09	-1.3	0.01	-1.5	0.01	-1.3
A_52_P237232	NM_009379	Thpo	0.99	1.0	0.03	-4.0	0.75	-1.3
A_30_P01023279			0.04	-1.5	0.00	-1.7	0.01	-1.4
A_30_P01019783			0.85	-1.1	0.04	-1.5	0.07	-1.4
A_30_P01025814			0.05	-1.3	0.00	-1.5	0.00	-1.4
A_51_P136870	AK030022		0.04	-1.3	0.00	-1.5	0.00	-1.4
A_51_P102257	AK053112		0.09	-1.4	0.00	-1.6	0.01	-1.4
A_30_P01032839			0.97	1.1	0.01	-4.6	0.71	-1.4
A_55_P2318934	AK004627	1200007C13Rik	0.32	-1.3	0.02	-1.5	0.04	-1.4
A_55_P2273929	AK076353	4732457N14	0.46	-1.2	0.03	-1.5	0.02	-1.4
A_55_P2125261	NR_028111	4930523C07Rik	0.19	-1.4	0.04	-1.5	0.03	-1.4
A_55_P2262753	AK035477	9530053J19Rik	0.01	-1.5	0.00	-1.4	0.00	-1.4
A_55_P1953400	NM_021477	A2bp1	0.45	-1.2	0.03	-1.5	0.03	-1.4
A_51_P115715	NM_023049	Asb2	0.23	-1.2	0.00	-1.5	0.00	-1.4
A_55_P2042500	NM_145635	BC054059	0.70	-1.3	0.02	-2.2	0.27	-1.4
A_55_P2413722	AK048632	C130090I23Rik	0.00	-1.5	0.01	-1.4	0.00	-1.4
A_51_P509573	NM_013652	Ccl4	0.08	-1.5	0.01	-1.6	0.03	-1.4
A_51_P493016	NM_023821	Cmya5	0.67	-1.3	0.04	-1.9	0.19	-1.4
A_55_P2028576	XM_001478577	Gm7057	0.21	-1.3	0.02	-1.5	0.00	-1.4
A_52_P16419	NM_010271	Gpd1	0.41	-1.3	0.01	-1.5	0.03	-1.4

A_55_P1963533	NM_001003672	Pcdhac2	0.12	-1.5	0.01	-1.7	0.05	-1.4
A_55_P2021011	NM_030207	Sfi1	0.02	-1.6	0.00	-1.6	0.04	-1.4
A_55_P2159234	NM_001033463	Tatdn2	0.06	-1.4	0.01	-1.6	0.03	-1.4
A_55_P2138960	NM_013861	Tpk1	0.04	-1.3	0.00	-1.5	0.00	-1.4
A_55_P2001583	NM_183180	Tspan18	0.46	-1.2	0.00	-1.6	0.01	-1.4
A_51_P426353	NM_009463	Ucp1	0.23	-1.7	0.02	-2.2	0.37	-1.4
A_30_P01019695			0.01	-2.2	0.07	-1.7	0.12	-1.5
A_55_P1956762	AK082974		0.04	-1.5	0.15	-1.3	0.01	-1.5
A_55_P2066453	AK081831		0.07	-1.4	0.02	-1.5	0.00	-1.5
A_30_P01025436			0.53	-1.2	0.06	-1.5	0.02	-1.5
A_30_P01022987			0.46	-1.2	0.45	-1.2	0.01	-1.5
A_55_P2187225	BQ885887		0.54	-1.2	0.09	-1.4	0.01	-1.5
A_51_P321600	AK224942		0.19	-1.4	0.14	-1.4	0.01	-1.5
A_30_P01022868			0.40	-1.3	0.07	-1.5	0.01	-1.5
A_55_P1955733	XM_001477303		0.44	-1.2	0.10	-1.3	0.00	-1.5
A_55_P2013780			0.54	-1.2	0.22	-1.2	0.00	-1.5
A_55_P1998721	AK137722		0.64	-1.1	0.34	-1.2	0.00	-1.5
A_30_P01022343			0.69	-1.1	0.26	-1.2	0.00	-1.5
A_30_P01029072			0.24	-1.2	0.03	-1.3	0.00	-1.5
A_66_P117815			0.88	-1.1	0.23	-1.2	0.00	-1.5
A_30_P01020487			0.05	-1.3	0.02	-1.4	0.00	-1.5
A_30_P01028277			0.61	-1.2	0.14	-1.3	0.00	-1.5
A_55_P2124110	AK004107		0.79	-1.1	0.39	-1.2	0.00	-1.5
A_30_P01021877			0.54	-1.2	0.12	-1.3	0.00	-1.5
A_30_P01018499			0.05	-1.3	0.00	-1.4	0.00	-1.5
A_30_P01021825			0.01	-1.3	0.00	-1.4	0.00	-1.5
A_55_P2069787			0.86	-1.1	0.63	-1.1	0.00	-1.5
A_30_P01032068			0.04	-1.4	0.03	-1.4	0.00	-1.5
A_30_P01019885			0.49	-1.2	0.01	-1.4	0.00	-1.5
A_30_P01029934			0.10	-1.3	0.03	-1.4	0.00	-1.5
A_30_P01028808			0.16	-1.3	0.01	-1.4	0.00	-1.5
A_30_P01025536			0.42	-1.2	0.07	-1.4	0.00	-1.5
A_30_P01030141			0.18	-1.2	0.03	-1.3	0.00	-1.5
A_30_P01032631			0.01	-1.4	0.03	-1.3	0.00	-1.5
A_30_P01024722			0.09	-1.3	0.11	-1.3	0.00	-1.5
A_55_P1991239	AK051413		0.28	-1.3	0.10	-1.4	0.00	-1.5
A_30_P01028474			0.03	-1.3	0.02	-1.3	0.00	-1.5
A_55_P2060793			0.82	-1.1	0.57	-1.1	0.00	-1.5
A_52_P37702	AK011813		0.10	-1.4	0.06	-1.4	0.00	-1.5
A_30_P01018341			0.76	-1.1	0.12	-1.3	0.00	-1.5
A_30_P01028737			0.36	-1.2	0.02	-1.4	0.00	-1.5
A_52_P163515			0.27	-1.2	0.07	-1.3	0.00	-1.5
A_30_P01021078			0.23	-1.3	0.11	-1.3	0.00	-1.5
A_30_P01033630			0.09	-1.3	0.02	-1.3	0.00	-1.5
A_30_P01033625			0.62	-1.2	0.08	-1.4	0.00	-1.5
A_55_P2072315	AK133410		0.72	-1.1	0.17	-1.3	0.00	-1.5
A_55_P2169135			0.58	-1.2	0.30	-1.2	0.00	-1.5
A_30_P01022751			0.61	-1.2	0.18	-1.3	0.00	-1.5
A_55_P1958137	XM_001479647		0.11	-1.3	0.07	-1.3	0.00	-1.5

A_30_P01028020			0.11	-1.3	0.04	-1.4	0.00	-1.5
A_55_P2072940	AK138152		0.22	-1.2	0.00	-1.4	0.00	-1.5
A_30_P01025096			0.56	-1.2	0.09	-1.3	0.00	-1.5
A_55_P2131672	AK163078		0.34	-1.2	0.04	-1.3	0.00	-1.5
A_30_P01017480			0.22	-1.3	0.02	-1.4	0.00	-1.5
A_30_P01025354			0.68	-1.1	0.21	-1.2	0.00	-1.5
A_30_P01028970			0.06	-1.3	0.01	-1.3	0.00	-1.5
A_55_P1982744			0.83	-1.1	0.26	-1.2	0.00	-1.5
A_55_P2083426			0.83	-1.1	0.62	-1.1	0.00	-1.5
A_30_P01027839			0.08	-1.3	0.10	-1.3	0.00	-1.5
A_55_P2090205	U04541		0.19	-1.4	0.41	-1.2	0.00	-1.5
A_55_P2004119			0.80	-1.1	0.55	-1.2	0.00	-1.5
A_30_P01032449			0.11	-1.3	0.07	-1.3	0.00	-1.5
A_55_P2148708			0.16	-1.3	0.04	-1.3	0.00	-1.5
A_55_P2126945	NM_001033168	1700066B19Rik	0.30	-1.2	0.03	-1.4	0.00	-1.5
A_55_P2060445	NM_026511	2810002N01Rik	0.22	-1.4	0.04	-1.5	0.02	-1.5
A_55_P1953157	NM_027279	2810422O20Rik	0.87	-1.1	0.14	-1.4	0.02	-1.5
A_55_P2305339	AK141715	6720427H10Rik	0.86	-1.0	0.06	-1.2	0.00	-1.5
A_55_P2065829	NM_199017	9230110C19Rik	0.15	-1.3	0.02	-1.4	0.00	-1.5
A_55_P1974522	NM_175688	A530099J19Rik	0.01	-1.6	0.03	-1.5	0.01	-1.5
A_55_P2238079	AK031253	A830054O07Rik	0.30	-1.2	0.01	-1.5	0.00	-1.5
A_55_P1996504	NM_011920	Abcg2	0.91	-1.0	0.31	-1.2	0.00	-1.5
A_55_P2039586	NM_001035531	Adrbk2	0.01	-1.3	0.00	-1.6	0.00	-1.5
A_52_P162509	NM_021414	Ahcyl2	0.10	-1.3	0.05	-1.3	0.00	-1.5
A_55_P2375121	AK045926	AI225934	0.55	-1.2	0.29	-1.2	0.00	-1.5
A_55_P2266178	BC028446	AI790276	0.13	-1.5	0.02	-1.7	0.02	-1.5
A_55_P2174743	NM_018747	Akap7	0.26	-1.3	0.06	-1.4	0.00	-1.5
A_55_P2016681	NM_025538	Alkbh7	0.11	-1.3	0.02	-1.3	0.00	-1.5
A_55_P2103501	XM_001474217	Ankrd29	0.16	-1.3	0.02	-1.5	0.00	-1.5
A_55_P2112360	NM_144529	Arhgap17	0.02	-1.2	0.00	-1.4	0.00	-1.5
A_55_P2108784	NM_153800	Arhgap22	0.77	-1.1	0.28	-1.2	0.00	-1.5
A_55_P2035087	NM_177566	Arhgef15	0.01	-1.3	0.00	-1.3	0.00	-1.5
A_52_P549166	NM_173451	Arsj	0.93	-1.0	0.93	-1.0	0.02	-1.5
A_51_P155763	NM_001146089	Ascc3	0.20	-1.3	0.11	-1.3	0.00	-1.5
A_55_P2003823	NM_009714	Asgr1	0.92	-1.1	0.12	-1.4	0.02	-1.5
A_52_P97670	NM_001099628	Atad2b	0.05	-1.4	0.06	-1.3	0.00	-1.5
A_51_P275496	XM_001480912	BC026762	0.70	-1.1	0.31	-1.2	0.00	-1.5
A_52_P413646	NM_007556	Bmp6	0.49	-1.1	0.56	-1.1	0.00	-1.5
A_55_P1985259	NM_001017985	C2cd3	0.96	-1.0	0.48	-1.2	0.00	-1.5
A_52_P416327	NM_178687	Cd226	0.81	-1.1	0.51	-1.2	0.01	-1.5
A_52_P657240	NM_027879	Cdc40	0.64	-1.1	0.22	-1.2	0.00	-1.5
A_55_P2008437	NM_009877	Cdkn2a	0.05	-1.5	0.01	-1.7	0.01	-1.5
A_55_P2073457	NM_173368	Chd6	0.06	-1.3	0.00	-1.5	0.00	-1.5
A_55_P2080956	NM_013490	Chka	0.17	-1.2	0.30	-1.1	0.00	-1.5
A_55_P1999082	NM_025809	Clec14a	0.92	-1.0	0.99	1.0	0.00	-1.5
A_51_P145662	NM_029465	Clec4g	0.31	1.3	0.60	-1.2	0.01	-1.5
A_52_P334670	NM_007715	Clock	0.03	-1.3	0.00	-1.4	0.00	-1.5
A_51_P302167	NM_172496	Cobl	0.59	-1.2	0.08	-1.4	0.00	-1.5
A_51_P304397	NM_027468	Cpm	0.23	-1.2	0.05	-1.3	0.00	-1.5

A_55_P2017914	NM_001113530	Csf1	0.46	-1.2	0.03	-1.4	0.00	-1.5
A_51_P363187	NM_008176	Cxcl1	0.08	-1.6	0.18	-1.4	0.04	-1.5
A_66_P135700	NM_001104531	Cyp2d11	0.72	-1.1	0.19	-1.2	0.00	-1.5
A_55_P2259500	AK083809	D130012P04Rik	0.06	-1.3	0.00	-1.5	0.00	-1.5
A_55_P2307080	AK083890	D130051D11Rik	0.27	-1.2	0.10	-1.3	0.00	-1.5
A_55_P2386256	AK051660	D130062J10Rik	0.16	-1.3	0.02	-1.3	0.00	-1.5
A_55_P2292046	AK084291	D230018H15Rik	0.64	-1.2	0.20	-1.3	0.01	-1.5
A_55_P2233373	AK032962	D930023I05Rik	0.54	-1.2	0.05	-1.4	0.01	-1.5
A_55_P1959973	NM_175539	Dcaf12l2	0.12	-1.3	0.04	-1.4	0.00	-1.5
A_51_P334104	NM_007833	Dcn	0.76	-1.2	0.03	-1.9	0.14	-1.5
A_51_P144349	NM_172442	Dtx4	0.10	-1.4	0.04	-1.4	0.00	-1.5
A_55_P2014978	NM_007889	Dvl3	0.49	-1.2	0.16	-1.3	0.00	-1.5
A_55_P2001233	NM_001162938	E430029J22Rik	0.97	-1.0	0.99	1.0	0.00	-1.5
A_66_P128761	NM_001162938	E430029J22Rik	0.93	1.0	0.90	-1.0	0.00	-1.5
A_51_P388478	NM_010110	Efnb1	0.05	-1.4	0.00	-1.5	0.00	-1.5
A_55_P2279140	AK089751	F830014O18Rik	0.08	-1.5	0.00	-1.6	0.01	-1.5
A_52_P5549	NM_001042501	Fam133b	0.87	-1.1	0.18	-1.3	0.01	-1.5
A_51_P447976	NM_001142952	Fam46c	0.13	-1.4	0.33	-1.2	0.00	-1.5
A_55_P2053933	NM_008259	Foxa1	0.39	-1.2	0.08	-1.3	0.00	-1.5
A_52_P233441	NM_008090	Gata2	0.09	-1.3	0.00	-1.5	0.00	-1.5
A_66_P111011	NM_008091	Gata3	0.98	-1.0	0.75	-1.1	0.01	-1.5
A_51_P411917	NM_010258	Gata6	0.02	-1.3	0.09	-1.2	0.00	-1.5
A_55_P2039196	NM_020014	Gfra4	0.12	-1.2	0.01	-1.3	0.00	-1.5
A_52_P467675	NM_001081971	Gm1337	0.75	-1.1	0.23	-1.2	0.00	-1.5
A_55_P2005420	XM_001478188	Gm16439	0.26	-1.3	0.01	-1.5	0.00	-1.5
A_55_P1999818	XM_001472026	Gm2006	0.66	-1.1	0.20	-1.3	0.00	-1.5
A_55_P2102759	XM_001473665	Gm2459	0.22	-1.3	0.07	-1.3	0.00	-1.5
A_55_P1980621	XM_001473665	Gm2459	0.91	-1.1	0.62	-1.1	0.00	-1.5
A_55_P2133624	XM_001475193	Gm2891	0.74	-1.1	0.25	-1.3	0.00	-1.5
A_55_P2109922	XM_001474867	Gm2921	0.33	-1.2	0.33	-1.2	0.00	-1.5
A_66_P113505	XM_001475977	Gm3006	0.11	-1.2	0.00	-1.4	0.00	-1.5
A_55_P2055523	XM_001475214	Gm3160	0.56	-1.1	0.12	-1.2	0.00	-1.5
A_55_P2042183	XM_001476516	Gm3181	0.39	-1.3	0.03	-1.5	0.00	-1.5
A_55_P1974780	XM_001476301	Gm3306	0.61	-1.2	0.13	-1.4	0.02	-1.5
A_55_P2134712	XM_001478003	Gm3781	0.02	-1.2	0.00	-1.3	0.00	-1.5
A_55_P2019838	XM_001478043	Gm3792	0.54	-1.2	0.16	-1.3	0.00	-1.5
A_55_P1980214	XM_001479026	Gm4099	0.02	-1.3	0.00	-1.3	0.00	-1.5
A_55_P2159850	XM_001479402	Gm4191	0.27	-1.3	0.09	-1.4	0.01	-1.5
A_55_P2063505	XM_001479508	Gm4235	0.43	-1.3	0.06	-1.4	0.01	-1.5
A_55_P2058467	XM_001479915	Gm4415	0.45	-1.2	0.04	-1.3	0.00	-1.5
A_55_P1960738	XM_001472780	Gm4470	0.53	-1.2	0.30	-1.2	0.00	-1.5
A_55_P2086835	XM_899874	Gm5514	0.60	-1.2	0.33	-1.2	0.00	-1.5
A_55_P1969396	XM_619384	Gm5809	0.19	-1.2	0.08	-1.2	0.00	-1.5
A_55_P2055763	NM_001034902	Gm5878	0.41	-1.2	0.12	-1.3	0.00	-1.5
A_55_P2055762	NM_001034902	Gm5878	0.08	-1.3	0.01	-1.4	0.00	-1.5
A_66_P106789	XM_894271	Gm6970	0.04	1.6	0.62	1.2	0.02	-1.5
A_55_P1957871	XM_983688	Gm8090	0.39	-1.2	0.22	-1.2	0.00	-1.5
A_55_P1957870	XM_983688	Gm8090	0.22	-1.2	0.01	-1.4	0.00	-1.5
A_55_P1957865	XM_001472293	Gm8090	0.90	-1.0	0.29	-1.2	0.00	-1.5

A_55_P2184567	XM_989827	Gm9677	0.61	-1.1	0.02	-1.4	0.00	-1.5
A_55_P2004806	NM_027658	Hexim2	0.07	-1.3	0.00	-1.4	0.00	-1.5
A_55_P1972018	NM_178208	Hist1h4c	0.66	-1.1	0.52	-1.1	0.00	-1.5
A_55_P1973349	NM_033596	Hist2h4	0.89	-1.1	0.84	-1.1	0.00	-1.5
A_51_P460643	NM_001079869	Hoxb3	0.16	-1.2	0.02	-1.3	0.00	-1.5
A_55_P1997951	NM_028242	Htatsf1	0.15	-1.2	0.03	-1.2	0.00	-1.5
A_66_P139546	NM_008344	Igfbp6	0.66	-1.2	0.10	-1.4	0.01	-1.5
A_55_P2092492	NM_001161842	Il18r1	0.83	-1.1	0.15	-1.4	0.02	-1.5
A_51_P505617	NM_008365	Il18r1	0.89	1.1	0.04	-1.4	0.00	-1.5
A_55_P2027737	NM_001025602	Il1rl1	0.57	-1.2	0.20	-1.2	0.00	-1.5
A_66_P103271	NM_001164598	Irf2bp2	0.02	-1.5	0.04	-1.4	0.00	-1.5
A_55_P2015670	NM_008397	Itga6	0.99	-1.0	0.97	-1.0	0.04	-1.5
A_55_P2004541	NM_001110323	Klra7	0.99	1.0	0.30	-1.3	0.04	-1.5
A_52_P42245	NM_010737	Klrb1a	0.38	-1.2	0.23	-1.2	0.00	-1.5
A_55_P1954006	NM_008492	Ldhb	0.56	-1.2	0.40	-1.2	0.00	-1.5
A_55_P2054728	NM_178886	Ldlrad3	0.09	-1.3	0.00	-1.4	0.00	-1.5
A_55_P2065537	XM_001478949	LOC100047427	0.37	-1.2	0.05	-1.4	0.00	-1.5
A_66_P118093	XM_001480420	LOC100048617	0.68	1.2	0.55	1.2	0.04	-1.5
A_55_P1988260	XM_001479382	LOC100048847	0.56	-1.2	0.16	-1.3	0.00	-1.5
A_55_P2378827	AK047890	LOC553096	0.03	-1.4	0.03	-1.4	0.00	-1.5
A_55_P1984523	XM_905877	LOC631924	0.04	-1.3	0.00	-1.4	0.00	-1.5
A_55_P2157522	XM_909351	LOC634585	0.13	-1.3	0.04	-1.3	0.00	-1.5
A_51_P116665	XM_912173	LOC636687	0.31	-1.2	0.07	-1.3	0.00	-1.5
A_55_P2150737	XM_975252	LOC674110	0.01	-1.3	0.00	-1.5	0.00	-1.5
A_55_P2036559	XM_990282	LOC676464	0.00	-1.3	0.00	-1.5	0.00	-1.5
A_51_P428134	NM_177152	Lrig3	0.96	-1.0	0.22	-1.2	0.00	-1.5
A_51_P296487	NM_146006	Lss	0.25	-1.3	0.05	-1.4	0.00	-1.5
A_55_P2062642	NM_025952	Magt1	0.20	-1.2	0.10	-1.2	0.00	-1.5
A_55_P2081388	NM_008575	Mdm4	0.60	-1.2	0.22	-1.3	0.00	-1.5
A_51_P386899	NM_145447	Mfsd7c	0.62	-1.2	0.24	-1.3	0.00	-1.5
A_51_P502132	NM_011985	Mmp23	0.31	-1.2	0.15	-1.3	0.00	-1.5
A_52_P123944	NM_029730	Mospd2	0.87	-1.1	0.06	-1.3	0.00	-1.5
A_51_P341746	NM_010823	Mpl	0.86	-1.1	0.29	-1.3	0.02	-1.5
A_51_P392928	NM_138745	Mthfd1	0.64	-1.1	0.32	-1.2	0.00	-1.5
A_55_P2069926	NM_009268	Mucl1	0.56	-1.2	0.15	-1.3	0.01	-1.5
A_55_P1987409	NM_173444	Nbeal1	0.05	-1.4	0.21	-1.3	0.01	-1.5
A_51_P151433	NM_026554	Ncbp2	0.78	-1.1	0.06	-1.3	0.00	-1.5
A_52_P484956	NM_010923	Nnat	0.95	1.0	0.80	-1.1	0.01	-1.5
A_55_P2102002	NM_021315	Noc3l	0.21	-1.2	0.11	-1.2	0.00	-1.5
A_55_P1991199	NM_001109985	Nos1ap	0.04	-1.3	0.01	-1.4	0.00	-1.5
A_66_P134808	NM_027528	Nos1ap	0.10	-1.3	0.00	-1.4	0.00	-1.5
A_55_P2158522	NM_183355	Pbx1	0.57	-1.2	0.15	-1.3	0.00	-1.5
A_55_P1983754	NM_025557	Pcp4l1	0.32	-1.2	0.03	-1.3	0.00	-1.5
A_51_P467448	NM_172453	Pif1	0.07	-1.2	0.00	-1.4	0.00	-1.5
A_55_P2121985	NM_013829	Plcb4	0.93	1.0	0.46	-1.2	0.01	-1.5
A_55_P2000039	NM_011073	Prf1	0.50	-1.2	0.08	-1.3	0.00	-1.5
A_55_P2004752	NM_181852	Prl2c5	0.08	-1.5	0.09	-1.4	0.01	-1.5
A_52_P350664	NM_153781	Pygb	0.29	-1.2	0.04	-1.3	0.00	-1.5
A_51_P381558	NM_133914	Rasa4	0.97	-1.0	0.16	-1.3	0.00	-1.5

A_52_P134023	NM_153100	Rtp3	0.48	-1.2	0.05	-1.4	0.00	-1.5
A_55_P2075070	NM_053190	S1pr5	1.00	1.0	0.46	-1.2	0.01	-1.5
A_55_P1973159	NM_011340	Serpinf1	0.24	-1.3	0.06	-1.4	0.00	-1.5
A_51_P103780	NM_145512	Sft2d2	0.29	-1.2	0.02	-1.4	0.00	-1.5
A_51_P268069	NM_009189	Six1	0.35	-1.4	0.26	-1.4	0.03	-1.5
A_55_P2015074	NM_029415	Slc10a6	0.27	-1.4	0.09	-1.4	0.02	-1.5
A_55_P2099650	NM_053195	Slc24a3	0.03	-1.3	0.01	-1.3	0.00	-1.5
A_52_P98614	NM_009209	Slc6a2	0.17	-1.5	0.68	-1.2	0.04	-1.5
A_55_P2085142	NM_009263	Spp1	0.44	-1.2	0.02	-1.4	0.00	-1.5
A_51_P240864	NM_029012	Sppl3	0.18	-1.2	0.02	-1.3	0.00	-1.5
A_51_P232371	NM_138672	Stab1	0.87	-1.1	0.15	-1.3	0.00	-1.5
A_51_P484158	NM_027399	Steap1	0.34	-1.2	0.19	-1.2	0.00	-1.5
A_52_P606774	NM_021420	Stk4	1.00	-1.0	0.98	-1.0	0.00	-1.5
A_51_P321341	NM_133670	Sult1a1	0.68	-1.2	0.02	-1.5	0.01	-1.5
A_55_P2000978	NM_176931	Syt15	0.57	-1.1	0.05	-1.3	0.00	-1.5
A_55_P2433438	NM_011544	Tcf12	0.89	-1.1	0.53	-1.2	0.01	-1.5
A_55_P2136657	NM_013691	Thbs3	0.89	1.1	0.97	-1.0	0.00	-1.5
A_66_P102879	NM_178060	Thra	0.15	-1.3	0.01	-1.4	0.00	-1.5
A_55_P2041070	NM_001083927	Tle3	0.41	-1.2	0.07	-1.3	0.00	-1.5
A_55_P2178327	NM_009390	Tll1	0.97	1.0	0.12	-1.3	0.00	-1.5
A_55_P2038247	NM_019392	Tyro3	0.54	-1.2	0.14	-1.3	0.00	-1.5
A_51_P183051	NM_133995	Upb1	0.74	-1.1	0.40	-1.2	0.00	-1.5
A_55_P2101666	NM_016872	Vamp5	0.14	-1.2	0.01	-1.3	0.00	-1.5
A_52_P556140	NM_019806	Vapb	0.49	-1.3	0.04	-1.7	0.06	-1.5
A_55_P2013996	XM_001004714		0.00	-1.6	0.02	-1.4	0.00	-1.6
A_30_P01023847			0.03	-1.5	0.00	-1.6	0.00	-1.6
A_30_P01022248			0.02	-1.5	0.00	-1.6	0.00	-1.6
A_30_P01026880			0.00	-1.5	0.03	-1.4	0.00	-1.6
A_30_P01031754			0.41	-1.3	0.04	-1.5	0.00	-1.6
A_30_P01032827			0.15	-1.3	0.03	-1.5	0.00	-1.6
A_30_P01026776			0.15	-1.4	0.00	-1.6	0.00	-1.6
A_30_P01021211			0.72	-1.2	0.14	-1.5	0.02	-1.6
A_30_P01029602			0.49	-1.3	0.07	-1.5	0.01	-1.6
A_30_P01024724			0.65	-1.2	0.37	-1.3	0.01	-1.6
A_30_P01023072			0.05	-1.3	0.02	-1.4	0.00	-1.6
A_55_P2108903	AK038731		0.46	-1.2	0.05	-1.4	0.00	-1.6
A_30_P01022552			0.03	-1.4	0.00	-1.4	0.00	-1.6
A_30_P01021464			0.34	-1.2	0.65	-1.1	0.00	-1.6
A_30_P01023830			0.04	-1.3	0.00	-1.4	0.00	-1.6
A_30_P01026192			0.56	-1.2	0.04	-1.4	0.00	-1.6
A_30_P01022704			0.29	-1.3	0.06	-1.4	0.00	-1.6
A_30_P01031385			0.91	-1.1	0.69	-1.1	0.00	-1.6
A_55_P2363902	AK011494	2610020P09Rik	0.03	-1.4	0.00	-1.5	0.00	-1.6
A_55_P2413727	AK029795	4930565A17	0.14	-1.4	0.17	-1.3	0.00	-1.6
A_55_P2098275	XM_001474884	5730416O20Rik	0.11	-1.4	0.21	-1.3	0.00	-1.6
A_55_P2073935	NM_013790	Abcc5	0.32	-1.2	0.01	-1.5	0.00	-1.6
A_52_P29151	NM_001081433	Ankrd44	0.12	-1.2	0.00	-1.4	0.00	-1.6
A_52_P565575	NM_175535	Arhgap20	0.95	1.0	0.79	-1.1	0.01	-1.6
A_51_P111612	NM_001042592	Arrdc4	0.18	-1.3	0.07	-1.4	0.00	-1.6

A_51_P484842	NM_007493	Asgr2	0.11	-1.2	0.01	-1.3	0.00	-1.6
A_51_P209736	NM_153778	Atoh8	0.22	-1.2	0.00	-1.4	0.00	-1.6
A_52_P168097	NM_009722	Atp2a2	0.21	-1.3	0.02	-1.5	0.00	-1.6
A_52_P1020860	XM_888885	AW112010	0.40	-1.2	0.01	-1.5	0.00	-1.6
A_51_P517430	NM_007639	Cd1d1	0.52	-1.1	0.04	-1.3	0.00	-1.6
A_55_P1971174	NM_007640	Cd1d2	0.85	-1.1	0.14	-1.3	0.00	-1.6
A_51_P312035	NM_172050	Cd300e	0.53	1.2	0.95	1.0	0.01	-1.6
A_55_P2175469	NM_001081345	Chd2	0.58	-1.2	0.31	-1.3	0.00	-1.6
A_52_P329367	NM_001025566	Chka	0.10	-1.3	0.00	-1.5	0.00	-1.6
A_51_P229925	NM_021715	Chst7	0.97	-1.0	0.58	-1.2	0.00	-1.6
A_55_P1962011	NM_019985	Clec1b	0.40	-1.3	0.17	-1.3	0.00	-1.6
A_55_P1962010	NM_019985	Clec1b	0.50	-1.3	0.45	-1.2	0.00	-1.6
A_55_P1962209	NM_030712	Cxcr6	0.36	-1.2	0.07	-1.4	0.00	-1.6
A_55_P2117959	NM_007823	Cyp4b1	0.33	-1.2	0.04	-1.3	0.00	-1.6
A_55_P1993777	NM_001039167	D11Bwg0517e	0.22	-1.3	0.05	-1.4	0.00	-1.6
A_55_P2128148	NM_001167918	D830031N03Rik	0.01	-1.5	0.03	-1.3	0.00	-1.6
A_55_P2333126	AK088506	E330018D03Rik	0.22	-1.2	0.02	-1.4	0.00	-1.6
A_55_P2036627	NM_001162938	E430029J22Rik	0.85	-1.1	0.56	-1.2	0.02	-1.6
A_55_P2017845	NM_153078	Ehbp1	0.19	-1.2	0.00	-1.5	0.00	-1.6
A_66_P109519	NM_007914	Ehf	0.42	-1.3	0.27	-1.4	0.03	-1.6
A_55_P2240823	NM_001128606	Epb4.1	0.82	-1.1	0.35	-1.2	0.00	-1.6
A_55_P1966109	NM_010148	Epn2	0.23	-1.3	0.09	-1.3	0.00	-1.6
A_52_P208613	NM_175027	Fancb	0.92	-1.0	0.27	-1.2	0.00	-1.6
A_51_P375987	NM_021716	Fign	0.08	-1.6	0.19	-1.4	0.01	-1.6
A_55_P2155848	NR_030683	Gm14207	0.44	1.4	0.67	1.2	0.02	-1.6
A_66_P125110	XM_001475977	Gm3006	0.16	-1.2	0.02	-1.3	0.00	-1.6
A_52_P239023	NM_001142957	Gm4455	0.14	-1.3	0.08	-1.3	0.00	-1.6
A_52_P33382	XM_356935	Gm5226	0.62	-1.2	0.49	-1.3	0.02	-1.6
A_52_P352362	NM_001039251	Gm7265	0.03	-1.3	0.01	-1.3	0.00	-1.6
A_55_P1967002	XM_001473953	Gm9441	0.05	-1.5	0.07	-1.4	0.00	-1.6
A_51_P436068	NM_007412	Gpr182	0.95	1.1	0.43	-1.3	0.04	-1.6
A_51_P128463	NM_001099296	Grrp1	0.86	-1.1	0.40	-1.2	0.00	-1.6
A_55_P2091147	NM_178211	Hist1h4k	0.77	-1.1	0.92	-1.0	0.00	-1.6
A_55_P2091145	NM_178211	Hist1h4k	0.63	-1.1	0.58	-1.1	0.00	-1.6
A_51_P436342	NM_008268	Hoxb5	0.32	-1.3	0.11	-1.3	0.00	-1.6
A_55_P2115871	NM_172439	Inpp5j	0.12	-1.5	0.04	-1.6	0.01	-1.6
A_52_P163849	NM_001039511	Ivns1abp	0.88	-1.1	0.28	-1.3	0.00	-1.6
A_55_P2408415	NR_001461	Kcnq1ot1	0.02	-1.4	0.00	-1.5	0.00	-1.6
A_51_P448178	NM_175519	Kctd8	0.84	-1.1	0.32	-1.2	0.00	-1.6
A_55_P1968799	NM_027157	Krtap1-5	0.95	-1.1	0.02	-3.8	0.49	-1.6
A_55_P2151209	NM_130873	Krtap16-4	0.46	-1.2	0.10	-1.3	0.00	-1.6
A_55_P2072041	XM_001472087	LOC100038980	0.56	-1.2	0.08	-1.4	0.00	-1.6
A_55_P2170279	XM_001480005	LOC100048321	0.81	-1.1	0.09	-1.3	0.00	-1.6
A_66_P112862	NR_033146	LOC100316870	0.16	-1.2	0.01	-1.3	0.00	-1.6
A_55_P2108486	XM_903408	LOC630284	0.00	-1.3	0.00	-1.5	0.00	-1.6
A_51_P479818	NM_028894	Lonrf3	0.63	-1.2	0.11	-1.4	0.00	-1.6
A_55_P2181597	NM_008546	Mfap2	0.00	-1.6	0.00	-1.7	0.00	-1.6
A_51_P500882	NM_010746	Ncr1	0.42	-1.2	0.10	-1.4	0.00	-1.6
A_55_P2172470	NM_022029	Nrgn	0.30	-1.3	0.12	-1.3	0.00	-1.6

A_52_P572197	NM_145962	Pank3	0.21	-1.3	0.07	-1.4	0.00	-1.6
A_52_P530291	NM_008842	Pim1	0.17	-1.3	0.02	-1.4	0.00	-1.6
A_51_P495780	NM_020568	Plin4	0.38	-1.5	0.03	-2.0	0.15	-1.6
A_51_P361492	NM_198934	Pou2f1	0.02	-1.4	0.01	-1.5	0.00	-1.6
A_51_P413111	AK147389	Ppm1l	0.56	-1.2	0.15	-1.2	0.00	-1.6
A_51_P340699	NM_026864	Rasl11a	0.02	-1.6	0.14	-1.4	0.00	-1.6
A_52_P541161	NM_022881	Rgs18	0.60	-1.2	0.18	-1.3	0.00	-1.6
A_55_P2142226	NM_001034870	Serpina3h	0.01	-7.8	0.13	-3.4	0.62	-1.6
A_55_P1975185	NM_009270	Sqle	0.21	-1.3	0.03	-1.4	0.00	-1.6
A_51_P240693	NM_027410	Tecpr1	0.01	-1.3	0.00	-1.5	0.00	-1.6
A_55_P2017636	NM_011580	Thbs1	0.81	-1.2	0.43	-1.3	0.04	-1.6
A_55_P2063465	NM_013691	Thbs3	0.92	1.0	0.75	-1.1	0.00	-1.6
A_52_P799815	NM_001025606	Tmem171	0.12	-1.3	0.02	-1.4	0.00	-1.6
A_55_P2034300	NM_001168256	Tmem40	0.79	1.2	0.49	-1.3	0.04	-1.6
A_55_P2145711	NM_172913	Tox3	0.97	-1.0	0.95	-1.0	0.00	-1.6
A_52_P274496	NM_183180	Tspan18	0.02	-1.3	0.01	-1.4	0.00	-1.6
A_55_P2055528	NM_178671	Ubxn10	0.35	-1.4	0.54	-1.2	0.02	-1.6
A_55_P2212027	NM_011682	Utrn	0.73	-1.1	0.56	-1.2	0.00	-1.6
A_66_P100937	XM_001487796	Zfp33b	0.94	-1.0	0.38	-1.2	0.00	-1.6
A_55_P1977003	XM_001475321		0.04	-1.6	0.00	-1.8	0.00	-1.7
A_55_P1967153	AK136565		0.01	-1.6	0.01	-1.7	0.00	-1.7
A_30_P01029711			0.16	-1.3	0.01	-1.5	0.00	-1.7
A_30_P01027919			0.29	-1.2	0.00	-1.5	0.00	-1.7
A_30_P01028904			0.02	-1.3	0.00	-1.5	0.00	-1.7
A_55_P1982075			0.28	-1.3	0.02	-1.6	0.00	-1.7
A_55_P2034625	AK082711		0.18	-1.3	0.00	-1.6	0.00	-1.7
A_30_P01032647			0.70	-1.2	0.18	-1.4	0.01	-1.7
A_30_P01022668			0.31	-1.2	0.02	-1.4	0.00	-1.7
A_30_P01032670			0.13	-1.4	0.10	-1.4	0.00	-1.7
A_55_P2105517	XM_001473956		0.01	-1.4	0.00	-1.4	0.00	-1.7
A_30_P01028021			0.31	-1.3	0.11	-1.3	0.00	-1.7
A_30_P01025092			0.02	-1.4	0.02	-1.4	0.00	-1.7
A_30_P01021970			0.30	-1.3	0.05	-1.4	0.00	-1.7
A_30_P01033587			0.56	-1.3	0.25	-1.4	0.00	-1.7
A_55_P2085835	NM_001033304	5330417C22Rik	0.58	-1.2	0.11	-1.3	0.00	-1.7
A_55_P2059640	NM_029631	Abhd14b	0.08	-1.7	0.18	-1.5	0.03	-1.7
A_55_P2038358	NM_012006	Acot1	0.50	-1.3	0.84	-1.1	0.01	-1.7
A_55_P2193512	BB034038	AI661384	0.65	-1.2	0.08	-1.4	0.00	-1.7
A_55_P1958921	AK162301	Ankrd29	0.18	-1.3	0.54	-1.1	0.00	-1.7
A_55_P2003824	NM_009714	Asgr1	0.93	-1.1	0.22	-1.5	0.02	-1.7
A_66_P102467	AK048565	Casz1	0.19	-1.3	0.00	-1.7	0.00	-1.7
A_55_P2050390	NM_174988	Cdh22	0.49	-1.2	0.10	-1.4	0.00	-1.7
A_51_P339540	NM_009876	Cdkn1c	0.47	-1.2	0.02	-1.5	0.00	-1.7
A_55_P2216976	AK143258	D13Ertd608e	0.11	-1.3	0.01	-1.5	0.00	-1.7
A_55_P2031167	NM_010107	Efna1	0.94	1.0	0.94	-1.0	0.00	-1.7
A_55_P1991911	XM_909743	EG626095	0.52	-1.2	0.17	-1.3	0.00	-1.7
A_52_P106259	NM_207655	Egfr	0.31	-1.2	0.00	-1.3	0.00	-1.7
A_55_P1956863	NM_007912	Egfr	0.07	-1.3	0.00	-1.3	0.00	-1.7
A_51_P286357	NM_139138	Emr4	0.90	1.1	0.99	1.0	0.00	-1.7

A_55_P2088018	NM_175276	Fhod3	0.42	-1.3	0.01	-1.6	0.00	-1.7
A_55_P2144556	NM_178382	Flrt3	0.76	-1.1	0.72	-1.1	0.00	-1.7
A_51_P361220	NM_008055	Fzd4	0.13	-1.3	0.00	-1.5	0.00	-1.7
A_51_P462428	AK019470	Galntl2	0.44	-1.4	0.08	-1.6	0.02	-1.7
A_55_P1957867	XM_001472293	Gm8090	0.33	-1.2	0.02	-1.4	0.00	-1.7
A_55_P2426941	BC040234	Gprin3	0.07	-1.3	0.01	-1.4	0.00	-1.7
A_55_P2158701	NM_175654	Hist1h4d	0.92	-1.0	0.87	-1.1	0.00	-1.7
A_55_P2125013	NM_178210	Hist1h4j	0.46	-1.2	0.62	-1.1	0.00	-1.7
A_55_P1988754	NM_175652	Hist4h4	0.67	-1.1	0.73	-1.1	0.00	-1.7
A_52_P253179	NM_008343	Igfbp3	0.67	-1.2	0.12	-1.4	0.00	-1.7
A_55_P2151638	NM_013793	Klra15	0.58	-1.2	0.07	-1.5	0.00	-1.7
A_55_P1979147	NM_001159904	Klrb1c	0.61	-1.2	0.17	-1.3	0.00	-1.7
A_66_P105422	NM_028894	Lonrf3	0.60	-1.1	0.07	-1.3	0.00	-1.7
A_55_P2062793	NM_008546	Mfap2	0.06	-1.4	0.00	-1.9	0.00	-1.7
A_55_P1980426	NM_010923	Nnat	0.87	1.1	0.79	-1.1	0.00	-1.7
A_55_P1973011	NM_178726	Ppm1l	0.02	-1.4	0.00	-1.5	0.00	-1.7
A_55_P2031989	NM_028724	Rin2	0.92	-1.1	0.34	-1.3	0.00	-1.7
A_55_P2168023	NM_001025379	Sema3g	0.71	1.2	0.74	1.1	0.00	-1.7
A_52_P273169	NM_001082414	Sh3d19	0.14	-1.3	0.05	-1.4	0.00	-1.7
A_55_P2094721	NM_009234	Sox11	0.99	-1.0	0.05	-1.7	0.02	-1.7
A_51_P210143	NM_001005510	Syne2	0.20	-1.3	0.06	-1.4	0.00	-1.7
A_55_P2413458	NM_023755	Tcfcp2l1	0.41	-1.2	0.18	-1.3	0.00	-1.7
A_55_P2062246	NM_001145164	Tgtp2	0.82	-1.2	0.27	-1.5	0.04	-1.7
A_52_P139413	NM_001100462	Tmem221	0.78	-1.2	0.80	-1.1	0.04	-1.7
A_55_P2126363	NM_011718	Wnt10b	0.98	1.0	0.99	1.0	0.00	-1.7
A_55_P2085826			0.13	-1.7	0.19	-1.6	0.02	-1.8
A_55_P2275402	AK020385	9330177L23Rik	0.31	-1.2	0.00	-1.6	0.00	-1.8
A_55_P2373987	AK047818	A730009E18Rik	0.00	-1.5	0.00	-1.6	0.00	-1.8
A_51_P458451	NM_009605	Adipoq	0.34	-2.0	0.01	-3.6	0.24	-1.8
A_55_P2266977	NM_021414	Ahcyl2	0.01	-1.4	0.00	-1.5	0.00	-1.8
A_52_P97572	NM_011784	Aplnr	0.49	1.4	0.68	1.2	0.03	-1.8
A_55_P2402929	AK080903	B430203I24Rik	0.32	-1.3	0.13	-1.3	0.00	-1.8
A_51_P419389	NM_007561	Bmpr2	0.03	-1.4	0.01	-1.4	0.00	-1.8
A_55_P2055985	NM_130904	Cd209d	0.72	-1.1	0.08	-1.3	0.00	-1.8
A_55_P2143837	NM_007735	Col4a4	0.72	-1.2	0.03	-1.6	0.00	-1.8
A_55_P2025675	NM_013811	Dnahc8	0.90	1.1	0.82	1.1	0.00	-1.8
A_55_P2153783	NM_010231	Fmo1	0.33	-1.3	0.26	-1.3	0.00	-1.8
A_52_P493091	NM_008031	Fmr1	0.41	-1.2	0.02	-1.5	0.00	-1.8
A_51_P267544	NM_013522	Frg1	0.36	-1.3	0.02	-1.5	0.00	-1.8
A_55_P2338200	NM_153803	Glb1l2	0.20	-1.3	0.00	-1.7	0.00	-1.8
A_55_P2058601	XM_920293	Gm7097	0.72	-1.2	0.20	-1.3	0.00	-1.8
A_51_P153995	NM_018762	Gp9	0.24	-1.5	0.19	-1.4	0.00	-1.8
A_55_P2119917	NM_011772	Ikzf4	0.03	-1.5	0.00	-1.8	0.00	-1.8
A_55_P1979674	NM_011772	Ikzf4	0.06	-1.4	0.00	-1.7	0.00	-1.8
A_55_P2001494	NM_013598	Kitl	0.23	-1.4	0.01	-1.6	0.00	-1.8
A_52_P52128	NM_001039522	Leo1	0.23	-1.4	0.21	-1.3	0.00	-1.8
A_52_P30451	NM_016854	Ppp1r3c	0.20	-1.4	0.01	-1.7	0.00	-1.8
A_55_P1984806	NM_022984	Retn	0.61	-1.6	0.01	-3.3	0.21	-1.8
A_51_P111164	NM_172612	Rnd1	0.86	-1.1	0.46	-1.2	0.00	-1.8

A_51_P247637	NM_080563	Rnf144a	0.08	-1.5	0.19	-1.3	0.00	-1.8
A_52_P532227	NM_007901	S1pr1	0.01	-1.6	0.00	-1.7	0.00	-1.8
A_51_P380432	NM_198885	Scx	0.76	-1.1	0.57	-1.1	0.00	-1.8
A_51_P493117	NM_025807	Slc16a9	0.18	-1.3	0.07	-1.3	0.00	-1.8
A_51_P279062	NM_027763	Treml1	0.35	-1.4	0.09	-1.5	0.00	-1.8
A_51_P108020	NM_001029929	Zmynd15	0.53	-1.2	0.00	-1.4	0.00	-1.8
A_30_P01028435			0.52	-1.3	0.03	-1.8	0.00	-1.9
A_30_P01021642			0.00	-1.4	0.01	-1.3	0.00	-1.9
A_51_P265571	NM_009627	Adm	0.04	-1.7	0.01	-1.8	0.00	-1.9
A_55_P2153620	NM_001039959	Ahnak	0.85	-1.2	0.31	-1.5	0.02	-1.9
A_51_P351194	NM_028219	Cnfn	0.37	1.6	0.57	-1.3	0.03	-1.9
A_55_P2395911	AK035822	D2Ertd295e	0.00	-1.6	0.00	-1.7	0.00	-1.9
A_55_P1977533	NM_001136062	Eno3	0.93	1.1	0.27	-1.6	0.04	-1.9
A_52_P508991	NM_010231	Fmo1	0.75	-1.2	0.20	-1.4	0.00	-1.9
A_66_P115004	NM_146017	Gabrp	0.74	-1.2	0.08	-1.6	0.00	-1.9
A_52_P669922	NM_032541	Hamp	0.84	-1.2	0.26	-1.6	0.04	-1.9
A_51_P258409	NM_010423	Hey1	0.94	-1.1	0.93	1.1	0.00	-1.9
A_55_P1998416	NM_008330	Ifi47	0.37	-1.3	0.11	-1.4	0.00	-1.9
A_52_P257502	NM_010517	Igfbp4	0.83	-1.1	0.29	-1.2	0.00	-1.9
A_55_P2061645	AK140300	ND6	0.10	-1.3	0.00	-1.5	0.00	-1.9
A_55_P2049186	NM_032398	Plvap	0.91	1.1	0.87	1.1	0.00	-1.9
A_55_P2428514	AK131869	Retn	0.39	-1.7	0.01	-3.0	0.10	-1.9
A_55_P2086885	NM_001033219	Slc45a4	0.05	-1.3	0.00	-1.5	0.00	-1.9
A_55_P1968355	NM_011599	Tle1	0.41	-1.2	0.12	-1.3	0.00	-1.9
A_55_P2025820	XM_001480287		0.15	-1.6	0.09	-1.6	0.00	-2.0
A_55_P2209053	AK013461	2900001G08Rik	0.57	-1.2	0.50	-1.2	0.00	-2.0
A_52_P337126	NM_024285	Bves	0.04	-1.5	0.01	-1.7	0.00	-2.0
A_52_P153291	NM_145700	Ccrl1	0.88	-1.1	0.11	-1.4	0.00	-2.0
A_55_P1964348	NM_023608	Gdpd2	0.92	-1.1	0.07	-2.0	0.02	-2.0
A_55_P2068459	NM_010479	Hspa1a	0.21	-1.6	0.03	-2.0	0.00	-2.0
A_51_P487690	NM_133871	Ifi44	0.15	-1.4	0.00	-1.6	0.00	-2.0
A_55_P2069818	NM_009158	Mapk10	0.61	-1.4	0.04	-2.2	0.03	-2.0
A_55_P2034245	NM_010791	Meox1	0.98	-1.0	0.05	-1.4	0.00	-2.0
A_55_P2165790	NM_178706	Siglech	0.05	-1.5	0.01	-1.6	0.00	-2.0
A_55_P2074453	NM_025807	Slc16a9	0.27	-1.4	0.17	-1.4	0.00	-2.0
A_51_P444137	NM_001143765	Syce1	0.54	-1.3	0.00	-2.0	0.00	-2.0
A_52_P229052	NM_019790	Tmeff2	0.95	-1.1	0.05	-1.8	0.00	-2.0
A_52_P146711	NM_009432	Tshb	0.55	-1.4	0.41	-1.4	0.02	-2.0
A_30_P01021593			0.00	-1.5	0.01	-1.4	0.00	-2.1
A_30_P01022310			0.00	-1.5	0.00	-1.4	0.00	-2.1
A_55_P2025655	CB248850		0.11	-1.8	0.09	-1.8	0.00	-2.1
A_55_P2004532	NM_175696	C530028O21Rik	0.45	-1.5	0.02	-2.1	0.00	-2.1
A_51_P196925	NM_009142	Cx3cl1	0.74	-1.2	0.13	-1.4	0.00	-2.1
A_55_P2007964	NM_009987	Cx3cr1	0.88	-1.1	0.21	-1.3	0.00	-2.1
A_55_P2033780	NM_010174	Fabp3	0.80	-1.2	0.14	-1.8	0.02	-2.1
A_51_P140237	NM_010212	Fhl2	0.52	-1.4	0.01	-2.3	0.00	-2.1
A_52_P514407	NM_013793	Klra15	0.61	-1.2	0.12	-1.5	0.00	-2.1
A_51_P308912	NM_026831	Mybphl	0.55	-1.6	0.02	-2.9	0.06	-2.1
A_52_P381484	NM_133903	Spon2	0.34	-1.4	0.17	-1.5	0.00	-2.1

A_55_P1985950	NM_133213	Xpnpep2	0.99	-1.0	0.27	-1.4	0.00	-2.1
A_51_P431329	NM_007606	Car3	0.74	-1.6	0.01	-5.1	0.21	-2.2
A_51_P148612	NM_009944	Cox7a1	0.76	-1.3	0.05	-2.1	0.01	-2.2
A_51_P336833	NM_024406	Fabp4	0.18	-2.3	0.00	-4.5	0.09	-2.2
A_52_P493620	NM_026218	Fgfr1op2	0.01	-1.7	0.00	-1.9	0.00	-2.2
A_55_P2126557	XM_619973	Gm5858	0.00	-1.6	0.00	-1.6	0.00	-2.2
A_51_P254262	NM_130858	Nxph3	0.02	-1.6	0.00	-1.9	0.00	-2.2
A_55_P2018666	NM_009381	Thrsp	0.32	-1.7	0.00	-2.8	0.01	-2.2
A_55_P2153021	NM_011652	Ttn	0.82	-1.3	0.04	-3.2	0.13	-2.2
A_55_P2179726	NM_027279	2810422O20Rik	0.81	-1.2	0.06	-1.9	0.00	-2.3
A_52_P650387	NM_001045530	Ccnjl	0.00	-1.6	0.00	-1.7	0.00	-2.3
A_66_P105032	NM_001145034	Gm13889	0.17	-1.4	0.00	-1.7	0.00	-2.3
A_55_P2028734	NM_013794	Klra16	0.61	-1.3	0.06	-1.7	0.00	-2.3
A_55_P2054362	XM_001472585	LOC100048875	0.78	-1.1	0.23	-1.4	0.00	-2.3
A_55_P2035320	NM_017373	Nfil3	0.01	-1.8	0.00	-2.2	0.00	-2.3
A_55_P2021585	NM_009362	Tff1	0.03	-3.0	0.09	-2.3	0.04	-2.3
A_51_P156955	NM_013459	Cfd	0.69	-1.7	0.01	-7.1	0.18	-2.4
A_52_P554703	NM_183183	Gprin3	0.01	-1.5	0.00	-1.7	0.00	-2.4
A_51_P179258	NM_001161665	Kif26b	0.02	-1.5	0.00	-1.7	0.00	-2.4
A_55_P2112005	NM_009362	Tff1	0.05	-2.8	0.10	-2.3	0.03	-2.4
A_51_P264495	NM_018870	Pgam2	0.70	-1.5	0.07	-2.6	0.03	-2.5
A_51_P290931	AK019450		0.26	-1.3	0.00	-1.8	0.00	-2.6
A_51_P439085	NM_023516	2310016C08Rik	0.60	-1.3	0.59	-1.2	0.00	-2.6
A_55_P2403769	BB498095	AI481121	0.02	-1.8	0.00	-2.6	0.00	-2.6
A_55_P2050226	AY072938	Ccrl1	0.86	-1.1	0.14	-1.4	0.00	-2.6
A_51_P283473	NM_026271	Fibin	0.32	-1.4	0.00	-2.0	0.00	-2.6
A_55_P2058783	NM_023516	2310016C08Rik	0.51	-1.3	0.51	-1.3	0.00	-2.7
A_52_P631547	NM_001081106	Cytl1	0.46	-1.4	0.00	-2.2	0.00	-2.7
A_51_P248638	NM_021503	Myoz2	0.50	-1.8	0.04	-3.4	0.04	-2.7
A_30_P01024606			0.00	-1.6	0.00	-1.6	0.00	-2.8
A_52_P638459	NM_013653	Ccl5	0.17	-1.9	0.01	-2.4	0.00	-2.8
A_55_P2170349	NM_053152	Klra22	0.30	-1.4	0.03	-1.8	0.00	-2.8
A_55_P2004536	NM_010649	Klra4	0.23	-1.5	0.02	-1.9	0.00	-2.8
A_55_P2129469	XM_918544	LOC641199	0.32	-1.6	0.00	-2.5	0.00	-2.9
A_51_P386983	NM_009406	Tnni3	0.69	-1.7	0.03	-4.1	0.06	-3.0
A_55_P2089710	NM_007904	Ednrb	0.40	-1.4	0.00	-2.4	0.00	-3.1
A_55_P1965030	NM_001003915	Slc5a12	0.01	-2.0	0.00	-2.8	0.00	-3.2
A_55_P1990032	NM_009141	Cxcl5	0.07	-2.3	0.02	-2.5	0.00	-3.3
A_65_P10195	NM_022879	Myl7	0.71	-1.7	0.04	-4.3	0.05	-3.3
A_55_P2000973	NM_181529	Syt15	0.99	-1.0	0.16	-1.4	0.00	-3.3
A_52_P577662	NM_007904	Ednrb	0.25	-1.5	0.00	-2.5	0.00	-3.5
A_55_P2408588	NM_007489	Arntl	0.00	-2.1	0.00	-2.6	0.00	-3.6
A_51_P416858	NM_021285	Myl1	0.98	-1.1	0.01	-5.4	0.01	-3.6
A_51_P356055	NM_175012	Grp	0.54	-1.3	0.00	-2.2	0.00	-3.8
A_52_P257625	NM_023612	Esm1	0.99	1.0	0.93	-1.1	0.00	-4.1
A_51_P175424	NM_011797	Car14	0.00	-1.8	0.00	-2.8	0.00	-4.3
A_51_P501844	NM_175475	Cyp26b1	0.04	-3.1	0.06	-2.7	0.00	-4.3
A_52_P413395	NM_025540	Sln	0.86	-1.4	0.07	-4.8	0.03	-4.7
A_55_P2011341	XM_001474162	LOC100045268	0.37	-1.8	0.01	-3.6	0.00	-5.7

A_55_P2094060	NM_010370	Gzma	0.21	-2.0	0.00	-3.8	0.00	-6.6
A_51_P331328	NM_026730	Gpihbp1	0.82	-1.2	0.26	-1.6	0.00	-10.7

3.3.3 BghiP Lung. Significant probe list. List of all significantly differentially expressed probes in at least 1 treatment group (FDR P ≤ 0.05, fold change ± 1.5) in response to sub-chronic oral exposure to 6.25, 12.5, and 25 mg/kg-bw/day benzo(ghi)perylene in the lung. The list is sorted from highest to lowest fold change in the 25 mg/kg-bw/day treatment group.

			**6.25 mg/kg-bw/day**		**12.5 mg/kg-bw/day**		**25 mg/kg-bw/day**	
Agilent Probe	Accession Number	Gene Symbol	*FDR P value*	*Fold change*	*FDR P value*	*Fold change*	*FDR P value*	*Fold change*
A_55_P2032081	NM_016974	Dbp	0.70	1.4	0.00	2.9	0.00	2.6
A_55_P2032079	NM_016974	Dbp	0.76	1.3	0.03	2.8	0.01	2.5
A_52_P460836	NM_177303	Lrrn4	0.12	1.8	0.00	2.1	0.00	2.4
A_51_P223776	NM_145434	Nr1d1	0.29	1.6	0.00	2.1	0.00	2.2
A_51_P520849	NM_009144	Sfrp2	0.21	1.8	0.02	2.0	0.00	2.1
A_52_P114889	NM_175309	Upk3b	0.11	1.7	0.00	1.8	0.00	2.0
A_55_P1960735	NM_011819	Gdf15	0.96	1.0	0.89	1.1	0.01	1.9
A_55_P1963463	NM_008067	Gabra3	0.00	1.4	0.00	1.5	0.00	1.8
A_30_P01023737			0.33	1.6	0.09	1.7	0.04	1.7
A_55_P2449750	NM_001100116	1700047I17Rik2	0.87	1.1	0.89	1.1	0.01	1.7
A_55_P1986282	NM_001111099	Cdkn1a	0.98	-1.0	0.94	1.0	0.00	1.7
A_51_P300618	NM_001163566	Crb2	0.31	1.5	0.03	1.7	0.03	1.7
A_55_P2054362	XM_001472585	LOC100048875	0.28	1.6	0.14	1.6	0.04	1.7
A_51_P304239	NM_010729	Loxl1	0.17	1.4	0.03	1.5	0.00	1.7
A_55_P2085771	NM_175561	Pcnxl2	0.34	1.4	0.07	1.5	0.01	1.7
A_55_P2109857	NM_009061	Rgs2	0.33	1.3	0.04	1.5	0.00	1.7
A_51_P142421	NM_138683	Rspo1	0.00	1.6	0.03	1.4	0.00	1.7
A_55_P1988689	AK131832		0.24	1.5	0.04	1.6	0.03	1.6
A_51_P363947	NM_007669	Cdkn1a	0.94	-1.1	0.99	1.0	0.04	1.6
A_51_P284426	NM_030137	Cstad	0.40	1.4	0.50	1.2	0.01	1.6
A_55_P2065866	NM_030206	Cygb	0.43	1.3	0.00	1.8	0.00	1.6
A_52_P566681	NM_153581	Gpm6a	0.18	1.5	0.16	1.4	0.00	1.6
A_51_P151902	NM_145584	Spon1	0.42	1.4	0.48	1.3	0.02	1.6
A_52_P62444	NM_013681	Syn2	0.55	1.3	0.67	1.2	0.03	1.6
A_51_P429276	NM_016963	Tmod3	0.35	1.4	0.86	1.1	0.01	1.6
A_52_P173442	NM_177292	Wscd2	0.56	1.2	0.00	1.6	0.00	1.6
A_30_P01027010			0.71	1.1	0.80	1.1	0.01	1.5
A_51_P191893	AK163489		0.34	1.2	0.09	1.3	0.00	1.5
A_30_P01020960			0.87	1.1	0.59	1.1	0.00	1.5
A_55_P2026982	NM_173744	2610019F03Rik	0.34	1.4	0.12	1.4	0.03	1.5
A_55_P2470474	AK087205	9530082P21Rik	0.75	1.1	0.39	1.3	0.04	1.5
A_55_P1954724	NR_002860	A130040M12Rik	0.25	1.3	0.10	1.3	0.01	1.5
A_55_P2169227	NM_177716	AI836003	0.61	1.2	0.60	1.2	0.00	1.5
A_51_P209327	NM_013912	Apln	0.57	1.3	0.41	1.3	0.04	1.5
A_52_P97572	NM_011784	Aplnr	0.39	1.4	0.17	1.4	0.04	1.5
A_55_P1978241	NM_009711	Artn	0.27	1.3	0.00	1.5	0.00	1.5
A_52_P601021	NM_026979	C1qtnf2	0.52	1.3	0.24	1.4	0.03	1.5

A_51_P497985	NM_013484	C2	0.29	1.4	0.00	1.5	0.02	1.5
A_51_P460954	NM_009139	Ccl6	0.55	1.2	0.05	1.5	0.01	1.5
A_55_P2005213	NM_145603	Ces2	0.62	1.2	0.10	1.4	0.01	1.5
A_55_P1959500	NM_172759	Ces5	0.81	1.1	0.04	1.5	0.02	1.5
A_51_P277006	NM_175140	Chst8	0.59	1.2	0.54	1.2	0.03	1.5
A_55_P2062469	NM_007730	Col12a1	0.53	1.2	0.29	1.3	0.00	1.5
A_55_P2118520	NM_007742	Col1a1	0.41	1.3	0.21	1.3	0.00	1.5
A_55_P2001250	NM_153107	Cpz	0.65	1.2	0.15	1.4	0.02	1.5
A_51_P140641	NM_145570	Fam176a	0.29	1.3	0.23	1.3	0.00	1.5
A_55_P2040245	NM_001039485	Fam38b	0.59	1.2	0.20	1.3	0.00	1.5
A_55_P1964664	NM_139149	Fus	0.48	1.3	0.24	1.3	0.01	1.5
A_52_P313217	NM_001081342	Gpr133	0.34	1.4	0.19	1.4	0.03	1.5
A_55_P1992910	NM_013755	Gyg	0.53	1.2	0.32	1.3	0.01	1.5
A_55_P2082929	NM_010389	H2-Ob	0.63	1.2	0.16	1.5	0.04	1.5
A_55_P2096226	NM_010729	Loxl1	0.50	1.2	0.09	1.4	0.00	1.5
A_51_P426270	NM_008597	Mgp	0.77	1.1	0.08	1.4	0.01	1.5
A_55_P2086682	NM_001113209	Nfib	0.28	1.3	0.04	1.3	0.00	1.5
A_66_P106808	NM_018732	Scn3a	0.80	1.1	0.43	1.3	0.04	1.5
A_55_P2103026	NM_028882	Sema3d	0.61	1.2	0.09	1.4	0.01	1.5
A_55_P2091506	NM_001100110	Srp54c	0.95	1.0	0.99	1.0	0.03	1.5
A_51_P450527	NM_011526	Tagln	0.93	1.1	0.59	1.2	0.03	1.5
A_65_P10913	NM_009367	Tgfb2	0.43	1.2	0.07	1.3	0.00	1.5
A_55_P2078633	NM_009780	C4b	0.36	1.5	0.04	1.7	0.22	1.4
A_51_P517695	NM_008530	Ly6f	0.95	-1.0	0.04	1.6	0.24	1.4
A_55_P2117525	NM_001166410	Rbm3	0.93	1.0	0.03	1.5	0.02	1.4
A_55_P2104975	NM_001168294	Serpina3f	0.70	1.2	0.04	1.8	0.22	1.4
A_55_P1962937	NM_031254	Trem2	0.81	1.1	0.03	1.5	0.10	1.4
A_51_P255657	XM_985599	2210011C24Rik	0.49	1.2	0.00	1.5	0.11	1.3
A_51_P278868	NM_010387	H2-DMb1	0.70	1.2	0.02	1.6	0.29	1.3
A_55_P2049752	NM_173749	Pamr1	0.37	1.3	0.04	1.5	0.14	1.3
A_55_P2001628	NR_003634	Rps4y2	0.25	1.4	0.04	1.5	0.23	1.3
A_66_P112024	XM_892675	Gm6816	0.42	1.3	0.00	1.5	0.34	1.2
A_55_P2070869	NM_008491	Lcn2	0.90	-1.1	0.03	1.5	0.41	1.2
A_51_P224164	NM_011867	Slc26a4	0.93	1.1	0.02	2.1	0.92	1.1
A_55_P1963483	NM_030559	Vps16	0.76	1.5	0.00	-5.9	0.95	1.1
A_52_P455404	NM_025922	Itpa	0.84	1.2	0.00	-2.9	1.00	-1.0
A_55_P2105512	NM_024193	Nop56	0.94	1.0	0.02	-1.5	0.66	-1.1
A_51_P514319	NM_172892	Slc13a4	0.74	-1.2	0.02	-1.5	0.40	-1.2
A_30_P01032068			0.11	-1.4	0.00	-1.6	0.08	-1.3
A_51_P308844	NM_153529	Nrn1	0.00	-1.5	0.20	-1.3	0.15	-1.3
A_66_P131433			0.42	-1.3	0.21	-1.4	0.02	-1.5
A_51_P124345	NM_153166	Cpne5	1.00	-1.0	0.56	-1.2	0.04	-1.5
A_66_P115531	NM_145151	Crebzf	0.76	-1.2	0.03	-1.6	0.07	-1.5
A_55_P1988970	NM_021527	Mkks	0.69	-1.1	0.17	-1.3	0.00	-1.5
A_66_P109368	NM_029525	Prex2	0.34	-2.9	0.03	-4.6	0.71	-1.5
A_55_P1969392	NM_026594	Rpl39l	0.08	-1.4	0.02	-1.4	0.01	-1.5
A_52_P670026	NM_021384	Rsad2	0.70	-1.3	0.03	-2.1	0.32	-1.5
A_51_P348652	NM_016962	Spast	0.39	-1.2	0.00	-1.4	0.00	-1.5
A_51_P316801	NM_146039	Wdr60	0.75	-1.1	0.44	-1.2	0.01	-1.5

A_55_P2138104	AK089567		0.41	-1.2	0.18	-1.3	0.00	-1.6
A_55_P2138100			0.48	-1.3	0.26	-1.3	0.00	-1.6
A_55_P2465382	AK172117		0.50	-1.2	0.09	-1.4	0.00	-1.6
A_55_P2035320	NM_017373	Nfil3	0.71	-1.2	0.11	-1.4	0.01	-1.6
A_51_P196972	NM_011403	Slc4a1	0.81	-1.2	0.03	-2.1	0.28	-1.6
A_51_P171616	NM_009518	Wnt10a	0.29	-1.5	0.14	-1.5	0.03	-1.6
A_55_P1968304			0.66	-1.2	0.14	-1.5	0.00	-1.7
A_55_P1966508	NM_010465	Hoxc6	0.22	-1.5	0.18	-1.4	0.00	-1.7
A_55_P2080603	NM_031165	Hspa8	0.64	-1.3	0.27	-1.4	0.03	-1.7
A_66_P124164	AK089567		0.17	-1.4	0.00	-1.5	0.00	-1.8
A_55_P1996456	NM_016799	Srrm1	0.54	-1.4	0.10	-1.6	0.00	-1.9
A_55_P2003513	NM_013559	Hsph1	0.59	-1.5	0.16	-1.8	0.01	-2.2
A_55_P2176963	NM_013559	Hsph1	0.55	-1.5	0.08	-2.1	0.01	-2.3
A_55_P1953377			0.29	-2.1	0.02	-2.6	0.01	-2.7
A_55_P2131766	NM_023665	D4Wsu53e	0.17	-2.0	0.00	-2.5	0.00	-3.4

3.3.4 BkF Lung. Significant probe list. List of all significantly differentially expressed probes in at least 1 treatment group (FDR P ≤ 0.05, fold change ± 1.5) in response to sub-chronic oral exposure to 25, 50, and 100 mg/kg-bw/day benzo(k)fluoranthene in the lung. The list is sorted from highest to lowest fold change in the 100 mg/kg-bw/day treatment group.

			**25 mg/kg-bw/day**		**50 mg/kg-bw/day**		**100 mg/kg-bw/day**	
Agilent Probe	Accession Number	Gene Symbol	*FDR P value*	*Fold change*	*FDR P value*	*Fold change*	*FDR P value*	*Fold change*
A_51_P279693	NM_009992	Cyp1a1	0.00	17.0	0.00	80.1	0.00	117.3
A_51_P255456	NM_009994	Cyp1b1	0.00	10.1	0.00	41.6	0.00	65.6
A_51_P254425	NM_009644	Ahrr	0.00	7.9	0.00	18.3	0.00	33.9
A_55_P2004099	XM_001477458	Gm9933	1.00	2.0	0.00	4.5	0.00	6.3
A_52_P413395	NM_025540	Sln	0.26	6.3	0.03	5.8	0.01	5.8
A_51_P449824	XM_001471750	Exoc3l2	1.00	2.1	0.03	3.4	0.00	5.2
A_66_P113892	NM_001162998	1110017F19Rik	0.08	3.9	0.23	2.2	0.00	5.1
A_66_P115580	AK076360		0.00	2.8	0.00	4.9	0.00	4.9
A_51_P231320	NM_008611	Mmp8	1.00	2.2	0.00	4.6	0.00	4.8
A_66_P118600	NM_008480	Lama1	0.13	3.4	0.00	4.8	0.00	4.1
A_55_P1985890	NM_178892	Tiparp	1.00	1.4	0.04	2.0	0.00	4.1
A_51_P193185	NM_013593	Mb	0.75	3.7	0.11	3.6	0.04	3.8
A_55_P1963483	NM_030559	Vps16	1.00	1.6	0.79	1.7	0.04	3.8
A_51_P110341	NM_170727	Scgb3a1	0.19	3.8	0.30	2.3	0.04	3.4
A_55_P2032081	NM_016974	Dbp	1.00	2.2	0.16	2.3	0.00	3.4
A_51_P202331	NM_011126	Plunc	0.00	13.0	0.02	7.3	0.11	3.3
A_55_P2032079	NM_016974	Dbp	1.00	2.2	0.15	2.4	0.01	3.3
A_51_P354126	NM_011260	Reg3g	0.00	8.6	0.59	2.5	0.27	3.1
A_51_P133684	NM_013808	Csrp3	1.00	3.1	0.04	3.9	0.12	3.1
A_51_P514961	NM_178892	Tiparp	1.00	1.3	0.06	2.0	0.00	3.1
A_51_P424338	NM_008706	Nqo1	0.72	1.6	0.00	2.5	0.00	3.0
A_52_P249733	NM_011540	Tcap	0.95	2.8	0.03	3.4	0.08	2.8
A_52_P675395	NM_007722	Cxcr7	1.00	1.5	0.02	2.5	0.01	2.8
A_51_P253481	NM_021456	Ces1	1.00	1.2	0.03	2.0	0.00	2.8

A_51_P256827	NM_013650	S100a8	1.00	2.1	0.00	5.9	0.06	2.6
A_55_P2000454	XM_001478824	Tspan18	1.00	1.7	0.03	2.4	0.01	2.6
A_55_P2047155	NM_001159569	Meis2	1.00	1.3	0.77	1.3	0.00	2.6
A_51_P187750	NM_153408	Neurl3	1.00	1.9	0.00	2.8	0.02	2.5
A_55_P1978316	NM_001081127	Adamts14	1.00	1.2	0.55	1.5	0.00	2.5
A_55_P1967978	NM_001136073	Nfatc2	1.00	1.2	0.08	2.0	0.00	2.5
A_55_P2144526	NM_001080381	Fam65b	1.00	1.4	0.56	1.5	0.02	2.4
A_51_P153063	NM_029310	Fabp12	1.00	1.7	0.00	2.8	0.02	2.3
A_51_P470079	NM_010555	Il1r2	1.00	1.3	0.00	3.7	0.12	2.2
A_55_P1960238	NM_172659	Slc2a6	1.00	1.3	0.04	2.8	0.10	2.2
A_51_P401501	NM_029921	Tmem213	0.57	1.8	0.00	2.4	0.01	2.2
A_52_P667913	NM_033592	Pcdhga9	1.00	-1.1	0.91	-1.2	0.04	2.2
A_52_P322181	NM_007419	Adrb1	1.00	1.6	0.59	1.4	0.00	2.2
A_55_P2040485	XM_889011	Ms4a4a	1.00	1.5	0.00	3.6	0.20	2.1
A_55_P2038106	NM_009978	Cst8	0.22	1.9	0.00	2.4	0.00	2.1
A_51_P153995	NM_018762	Gp9	1.00	1.5	0.03	2.1	0.04	2.0
A_51_P144264	NM_008452	Klf2	0.98	1.4	0.02	1.9	0.00	2.0
A_51_P419246	NR_028427	5830416P10Rik	1.00	1.2	0.59	1.4	0.04	2.0
A_51_P498631	NM_018769	Dfna5	1.00	1.3	0.14	1.7	0.02	2.0
A_55_P2162935	NM_008744	Ntn1	1.00	1.5	0.36	1.6	0.01	2.0
A_55_P2048493	NR_027955	4931440P22Rik	1.00	1.2	0.17	1.4	0.00	2.0
A_52_P559975	NM_009909	Cxcr2	1.00	1.3	0.04	2.7	0.23	1.9
A_55_P2027083	NM_001039484	Kcnj10	1.00	1.4	0.00	2.0	0.00	1.9
A_51_P382152	NM_011171	Procr	1.00	1.2	0.03	1.9	0.00	1.9
A_51_P299805	NM_027872	Slc46a3	1.00	1.2	0.02	1.5	0.00	1.9
A_51_P256384	NM_009723	Atp2b2	1.00	1.2	0.79	1.2	0.04	1.9
A_51_P392303	NM_010733	Lrrn3	1.00	1.2	0.93	1.1	0.04	1.9
A_55_P1973770	NM_029770	Unc5b	1.00	1.6	0.59	1.4	0.03	1.9
A_51_P147123	NM_008744	Ntn1	1.00	1.2	0.70	1.3	0.02	1.9
A_55_P2128388	NM_009644	Ahrr	1.00	1.2	0.58	1.3	0.01	1.9
A_51_P290576	NM_152804	Plk2	1.00	1.3	0.61	1.3	0.01	1.9
A_55_P2148534	BC096461	Nr1d2	0.83	1.6	0.30	1.4	0.00	1.9
A_55_P2056729	NM_008342	Igfbp2	1.00	1.2	0.38	1.4	0.00	1.9
A_52_P398925	NM_173869	Stfa2l1	1.00	1.5	0.00	4.1	0.48	1.8
A_55_P1962011	NM_019985	Clec1b	1.00	1.0	0.03	1.8	0.00	1.8
A_55_P2101340	NM_019511	Ramp3	0.83	1.6	0.03	1.8	0.02	1.8
A_66_P106113	NM_023275	Rhoj	1.00	1.3	0.59	1.3	0.04	1.8
A_55_P2329923	NM_145358	Camkk2	1.00	1.2	0.66	1.3	0.01	1.8
A_66_P135391	NM_008342	Igfbp2	1.00	1.2	0.55	1.3	0.00	1.8
A_30_P01020131			1.00	1.2	0.03	3.1	0.37	1.7
A_51_P464918	NM_019453	Mefv	1.00	1.3	0.03	2.1	0.10	1.7
A_55_P1959818	NM_001168256	Tmem40	1.00	1.2	0.00	2.1	0.07	1.7
A_51_P428372	NM_023785	Ppbp	1.00	1.1	0.04	1.9	0.10	1.7
A_55_P1966987	NM_010717	Limk1	1.00	1.2	0.02	1.5	0.00	1.7
A_55_P2057050	AK038034		1.00	1.3	0.88	1.1	0.04	1.7
A_51_P108659	NM_011134	Pon1	1.00	1.2	0.85	1.2	0.04	1.7
A_55_P1962404	NM_145079	Ugt1a6a	1.00	1.4	0.33	1.5	0.04	1.7
A_30_P01027194			1.00	1.2	0.78	1.2	0.04	1.7
A_55_P2119985	NM_130862	Baiap2	1.00	1.2	0.48	1.3	0.02	1.7

A_55_P2470474	AK087205	9530082P21Rik	1.00	1.3	0.43	1.4	0.02	1.7
A_55_P1957038	NM_181796	Gstp2	1.00	1.2	0.08	1.5	0.02	1.7
A_30_P01031348			1.00	1.1	0.99	-1.0	0.02	1.7
A_55_P2042156	NM_007377	Aatk	1.00	1.4	0.41	1.3	0.01	1.7
A_51_P254855	NM_011198	Ptgs2	1.00	1.1	0.84	1.2	0.01	1.7
A_55_P2128646	NM_020567	Gmnn	1.00	1.2	0.46	1.3	0.01	1.7
A_51_P291501	NM_001081062	Ccno	1.00	1.2	0.47	1.3	0.00	1.7
A_52_P295104	NM_183259	2210020M01Rik	0.25	1.5	0.96	1.0	0.00	1.7
A_55_P2029558	NM_007420	Adrb2	1.00	1.1	0.06	1.5	0.00	1.7
A_52_P94521	NM_001001881	2510009E07Rik	1.00	1.2	0.06	1.6	0.00	1.7
A_55_P2037343	NM_010391	H2-Q10	1.00	1.4	0.03	2.1	0.16	1.6
A_51_P212782	NM_008361	Il1b	1.00	1.5	0.03	2.0	0.25	1.6
A_55_P2034300	NM_001168256	Tmem40	1.00	1.1	0.02	1.9	0.19	1.6
A_55_P1998471	NM_009114	S100a9	1.00	1.3	0.04	1.7	0.03	1.6
A_55_P2394308	NM_008046	Fst	1.00	1.2	0.02	1.7	0.04	1.6
A_55_P1954835	XM_001475752	LOC100046186	1.00	1.5	0.00	1.7	0.05	1.6
A_55_P1970537	NM_009745	Bcl7b	1.00	-1.0	0.99	-1.0	0.04	1.6
A_51_P234728	AK032901	Lpar6	1.00	1.2	0.11	1.5	0.03	1.6
A_55_P1961014	NM_009150	Selenbp1	1.00	1.1	0.70	1.2	0.03	1.6
A_51_P465128	NM_029935	Chst15	1.00	1.3	0.88	1.1	0.03	1.6
A_55_P2006668	NM_175356	Pi4kb	1.00	1.2	0.98	-1.0	0.03	1.6
A_55_P1957835	NM_198300	Cpeb3	1.00	1.4	0.10	1.4	0.02	1.6
A_55_P2119257	NM_008871	Serpine1	1.00	1.2	0.33	1.3	0.00	1.6
A_55_P2183010	NM_001038887	P2rx7	1.00	1.1	0.10	1.4	0.00	1.6
A_66_P110798	AK145507		1.00	1.3	0.70	1.2	0.00	1.6
A_55_P1981670	NM_033144	41160.0	1.00	1.0	0.36	1.3	0.00	1.6
A_51_P240723	NR_003953	1700022A21Rik	1.00	1.2	0.93	1.1	0.00	1.6
A_51_P516133	NM_015786	Hist1h1c	1.00	1.1	0.04	1.3	0.00	1.6
A_52_P534583	NM_133245	Ahsp	1.00	1.6	0.00	2.6	0.36	1.5
A_51_P196972	NM_011403	Slc4a1	1.00	1.3	0.04	2.5	0.54	1.5
A_55_P1953003	NM_080467	Atp6v0a4	0.13	1.8	0.00	2.1	0.11	1.5
A_55_P2031999	NM_145448	9030617O03Rik	1.00	1.2	0.00	1.7	0.04	1.5
A_51_P149621	NM_024222	Stt3b	1.00	1.2	0.02	1.6	0.05	1.5
A_55_P2037528	NM_144932	Acsf3	0.79	1.5	0.02	1.5	0.05	1.5
A_51_P191893	AK163489		1.00	1.2	0.99	1.0	0.04	1.5
A_55_P1994817	NM_027427	Taf15	1.00	1.0	0.98	1.0	0.04	1.5
A_55_P1952720	NM_183285	Kctd2	1.00	1.3	0.87	1.1	0.04	1.5
A_55_P2007713	NM_053082	Tspan4	1.00	1.3	0.24	1.4	0.04	1.5
A_55_P1967069	NM_175116	Lpar6	1.00	1.2	0.27	1.4	0.03	1.5
A_55_P2183015	NM_011026	P2rx4	1.00	1.2	0.60	1.2	0.03	1.5
A_55_P2030771	NM_019406	Fnbp1	1.00	1.1	0.87	1.1	0.03	1.5
A_30_P01023888			1.00	1.2	0.79	1.2	0.03	1.5
A_51_P456465	NM_021386	Cldn10a	1.00	1.2	0.80	1.2	0.03	1.5
A_30_P01020515			1.00	1.1	0.69	1.2	0.02	1.5
A_30_P01027491			1.00	1.1	0.46	1.3	0.02	1.5
A_55_P1976351	NM_001042672	Prei4	1.00	1.1	0.94	1.0	0.02	1.5
A_66_P134707	NM_001081323	Mphosph9	1.00	1.1	0.95	1.1	0.02	1.5
A_66_P124715	NM_011145	Ppard	1.00	1.2	0.43	1.3	0.02	1.5
A_55_P2074035	NM_025325	Haao	1.00	1.3	0.85	1.1	0.02	1.5

A_55_P2104835	AK162820		1.00	1.2	0.38	1.3	0.02	1.5
A_30_P01027077			1.00	1.2	0.45	1.2	0.01	1.5
A_55_P2116650	NR_002860	A130040M12Rik	1.00	1.0	0.88	1.1	0.01	1.5
A_55_P1993609	NM_028201	2210009G21Rik	1.00	1.2	0.36	1.3	0.00	1.5
A_30_P01030038			1.00	1.0	0.97	1.0	0.00	1.5
A_55_P2121456	NM_177632	Fam43a	1.00	1.2	0.51	1.2	0.00	1.5
A_55_P2126951	NM_001085417	Zfp467	1.00	1.2	0.70	1.2	0.00	1.5
A_52_P425839	NM_181596	Retnlg	1.00	1.2	0.00	3.5	0.67	1.4
A_52_P354744	NM_011401	Slc2a3	1.00	1.1	0.00	1.6	0.00	1.4
A_55_P2165839	NM_207131	Cebpe	1.00	1.3	0.04	1.5	0.06	1.4
A_55_P2008704	NM_181796	Gstp2	1.00	1.3	0.02	1.5	0.06	1.4
A_55_P1981804	NM_010326	Gp1ba	1.00	1.2	0.02	1.6	0.17	1.3
A_51_P374464	NM_013541	Gstp1	1.00	1.2	0.03	1.5	0.08	1.3
A_55_P2031668	NM_013541	Gstp1	1.00	1.3	0.02	1.5	0.11	1.3
A_52_P541161	NM_022881	Rgs18	1.00	1.0	0.02	2.0	0.69	1.2
A_51_P469008	NM_010119	Ehd1	1.00	1.1	0.04	1.5	0.41	1.2
A_55_P2055854	NM_011144	Ppara	1.00	1.1	0.04	1.5	0.33	1.2
A_55_P2019690	NM_001081349	Slc43a1	1.00	1.1	0.00	1.5	0.52	1.2
A_52_P54261	NM_178936	Tmem56	0.19	1.5	0.00	1.5	0.28	1.2
A_51_P113906	NM_025467	Gkn2	0.00	8.8	0.90	1.3	0.97	1.1
A_55_P1996973	NM_029000	Gvin1	1.00	-1.0	0.03	2.1	0.90	1.1
A_30_P01026045			1.00	1.1	0.03	1.5	0.86	1.1
A_55_P2021585	NM_009362	Tff1	0.00	5.8	0.81	1.4	0.99	1.0
A_55_P1973362	NM_010603	Kcnj12	1.00	1.1	0.04	1.5	0.93	-1.0
A_55_P2112005	NM_009362	Tff1	0.00	7.9	0.72	1.6	0.94	-1.1
A_55_P1984745	NM_001085376	Pappa2	1.00	-1.3	0.04	-1.7	0.72	-1.2
A_55_P1989076	NM_001033167	Slc22a23	1.00	-1.1	0.04	-1.5	0.18	-1.3
A_55_P2087414	NM_011348	Sema3e	1.00	-1.0	0.03	-1.5	0.31	-1.3
A_55_P2156425	NM_026815	Upk1a	1.00	-1.2	0.03	-1.5	0.13	-1.3
A_52_P186033	NM_009259	Spn	1.00	-1.2	0.04	-1.6	0.42	-1.3
A_52_P223508	NR_026976	6720401G13Rik	1.00	-1.4	0.03	-1.6	0.32	-1.3
A_51_P319460	NM_011019	Osmr	1.00	-1.2	0.03	-1.6	0.21	-1.3
A_66_P103027	NM_172732	Clec9a	0.32	-1.6	0.03	-1.5	0.06	-1.4
A_55_P2038358	NM_012006	Acot1	1.00	-1.2	0.02	-1.6	0.14	-1.4
A_51_P243514	NM_001163136	Macc1	1.00	-1.1	0.00	-1.6	0.05	-1.4
A_52_P22763	NM_001039934	Mtap2	0.42	-1.4	0.00	-1.7	0.00	-1.4
A_51_P161054	NM_021359	Itgb6	1.00	-1.1	0.04	-1.8	0.46	-1.4
A_51_P509679	XM_001474025		1.00	-1.4	0.04	-1.9	0.42	-1.4
A_55_P1983858	NM_022886	Scel	1.00	-1.3	0.00	-2.0	0.17	-1.4
A_55_P2120551	XM_001474125	9930104M19Rik	1.00	-1.3	0.05	-1.5	0.02	-1.5
A_55_P1960097	NM_013813	Epb4.1l3	1.00	-1.2	0.04	-1.5	0.02	-1.5
A_55_P2175245	NM_030254	Tusc3	1.00	-1.2	0.00	-1.5	0.00	-1.5
A_55_P2170350	NM_053152	Klra22	0.43	-1.4	0.02	-1.6	0.04	-1.5
A_55_P1965101	NM_001081643	Xlr3b	0.59	-1.6	0.02	-1.6	0.05	-1.5
A_55_P2009988	NM_175093	Trib3	1.00	-1.1	0.00	-1.6	0.04	-1.5
A_55_P1978681	NM_146010	Tspan8	1.00	-1.2	0.00	-1.7	0.03	-1.5
A_55_P2125613	NM_001101486	Fam71f2	1.00	-1.2	0.00	-1.9	0.06	-1.5
A_55_P2042973	XM_001473782	1010001N08Rik	1.00	-1.1	0.58	-1.2	0.04	-1.5
A_51_P108020	NM_001029929	Zmynd15	1.00	-1.3	0.11	-1.5	0.04	-1.5

A_55_P2069926	NM_009268	Mucl1	1.00	-1.2	0.11	-1.4	0.04	-1.5
A_51_P257762	NM_020005	Kat2b	1.00	-1.2	0.66	-1.2	0.04	-1.5
A_55_P2095047	NM_145569	Mat2a	1.00	-1.1	0.74	-1.2	0.04	-1.5
A_51_P496054	NM_080855	Zcchc14	1.00	-1.1	0.81	-1.2	0.04	-1.5
A_52_P650180	NM_145469	Nipal2	1.00	-1.2	0.85	-1.1	0.04	-1.5
A_51_P300434	NM_134448	Dst	1.00	-1.2	0.69	-1.2	0.03	-1.5
A_55_P2216976	AK143258	D13Ertd608e	1.00	-1.0	0.07	-1.5	0.03	-1.5
A_55_P2102951			1.00	-1.0	0.56	-1.3	0.02	-1.5
A_55_P2178664			1.00	-1.0	0.71	-1.2	0.02	-1.5
A_30_P01025757			1.00	-1.2	0.49	-1.2	0.01	-1.5
A_51_P114297	NM_010631	Kifc3	1.00	1.0	0.89	-1.1	0.01	-1.5
A_55_P2016114	NM_010177	Fasl	0.91	-1.3	0.03	-1.4	0.00	-1.5
A_30_P01021461			1.00	-1.2	0.49	-1.2	0.00	-1.5
A_30_P01029537			1.00	-1.3	0.58	-1.2	0.00	-1.5
A_55_P1999364	NM_016879	Krt85	0.90	-1.4	0.15	-1.3	0.00	-1.5
A_65_P10913	NM_009367	Tgfb2	1.00	-1.2	0.35	-1.3	0.00	-1.5
A_55_P2189201	AK020017	5830426C09Rik	1.00	-1.2	0.43	-1.2	0.00	-1.5
A_55_P2022332	XM_916590	Klra27	1.00	-1.2	0.10	-1.2	0.00	-1.5
A_55_P2040888	NM_001042620	Dhx15	1.00	-1.1	0.76	-1.2	0.00	-1.5
A_30_P01024768			1.00	-1.3	0.05	-1.5	0.01	-1.6
A_55_P2338200	NM_153803	Glb1l2	0.80	-1.4	0.00	-1.7	0.00	-1.6
A_51_P111612	NM_001042592	Arrdc4	1.00	-1.3	0.02	-1.8	0.03	-1.6
A_55_P1988844	NM_016970	Klrg1	1.00	-1.5	0.23	-1.5	0.04	-1.6
A_55_P2214124	BC019425		1.00	-1.3	0.61	-1.3	0.04	-1.6
A_52_P484956	NM_010923	Nnat	1.00	-1.2	0.06	-1.7	0.04	-1.6
A_30_P01032827			1.00	-1.0	0.88	-1.1	0.04	-1.6
A_52_P78373	NM_011366	Sorbs3	1.00	-1.0	0.96	-1.0	0.04	-1.6
A_30_P01030198			1.00	-1.4	0.11	-1.5	0.04	-1.6
A_55_P2092492	NM_001161842	Il18r1	1.00	-1.1	0.37	-1.4	0.04	-1.6
A_55_P1980426	NM_010923	Nnat	1.00	-1.4	0.12	-1.5	0.03	-1.6
A_51_P344249	NM_009685	Apbb1	1.00	-1.1	0.48	-1.3	0.03	-1.6
A_51_P338397	NM_026145	Kctd10	1.00	-1.2	0.30	-1.4	0.03	-1.6
A_55_P1986551	NM_023061	Mcam	1.00	1.1	0.87	-1.1	0.03	-1.6
A_51_P268094	NM_009255	Serpine2	1.00	-1.2	0.33	-1.3	0.02	-1.6
A_55_P2096226	NM_010729	Loxl1	1.00	1.0	0.48	-1.3	0.02	-1.6
A_65_P05152	NM_201518	Flrt2	1.00	-1.1	0.99	1.0	0.02	-1.6
A_52_P22590	NM_001024720	Hmcn1	1.00	-1.0	0.45	-1.3	0.02	-1.6
A_55_P2071716	NM_153590	Klre1	1.00	-1.1	0.25	-1.3	0.02	-1.6
A_52_P72237	NM_009609	Actg1	1.00	-1.1	0.80	-1.2	0.01	-1.6
A_51_P235878	NM_008193	Guk1	1.00	-1.1	0.43	-1.3	0.01	-1.6
A_51_P153170	NM_029787	Cyb5r3	1.00	-1.1	0.46	-1.3	0.01	-1.6
A_51_P403273	NM_025816	Tax1bp1	1.00	-1.2	0.04	-1.3	0.00	-1.6
A_30_P01028065			1.00	-1.1	0.00	-1.4	0.00	-1.6
A_30_P01022383			1.00	-1.2	0.04	-1.4	0.00	-1.6
A_52_P650387	NM_001045530	Ccnjl	1.00	-1.2	0.00	-1.7	0.00	-1.7
A_51_P505617	NM_008365	Il18r1	1.00	-1.3	0.02	-1.8	0.03	-1.7
A_51_P477121	NM_021451	Pmaip1	0.91	-1.5	0.00	-1.8	0.01	-1.7
A_55_P1961968	NM_009538	Plagl1	1.00	-1.2	0.04	-2.0	0.08	-1.7
A_52_P257625	NM_023612	Esm1	1.00	-1.6	0.00	-2.8	0.11	-1.7

A_51_P286034	NM_134033	Ccdc117	1.00	-1.1	0.89	-1.1	0.04	-1.7
A_66_P136064	XM_001479876	LOC100048139	1.00	-1.4	0.49	-1.4	0.04	-1.7
A_51_P389531	NM_019830	Prmt1	1.00	-1.1	0.88	-1.1	0.04	-1.7
A_55_P2089820	NM_134079	Adk	1.00	-1.4	0.11	-1.6	0.04	-1.7
A_55_P1957424	NM_172294	Sulf1	1.00	-1.1	0.98	1.0	0.04	-1.7
A_52_P481423	NM_030249	Cttnbp2nl	1.00	-1.1	0.92	-1.1	0.04	-1.7
A_51_P319070	NM_026159	Retsat	1.00	-1.1	0.84	-1.2	0.04	-1.7
A_52_P227937	BC028765		1.00	-1.2	0.44	-1.4	0.04	-1.7
A_55_P2123673	NM_001081156	Trnp1	0.49	-1.7	0.20	-1.5	0.03	-1.7
A_51_P332201	NM_009976	Cst3	1.00	-1.2	0.73	-1.2	0.02	-1.7
A_51_P466229	NM_026840	Pdgfrl	1.00	-1.2	0.06	-1.6	0.02	-1.7
A_51_P490023	NM_009450	Tubb2a	1.00	-1.2	0.41	-1.3	0.02	-1.7
A_51_P475342	NM_009601	Chrnb1	1.00	-1.3	0.43	-1.3	0.01	-1.7
A_55_P1977498	NM_008311	Htr2b	1.00	-1.4	0.17	-1.5	0.01	-1.7
A_51_P500882	NM_010746	Ncr1	0.72	-1.5	0.09	-1.5	0.00	-1.7
A_55_P1963463	NM_008067	Gabra3	1.00	-1.0	0.88	-1.1	0.00	-1.7
A_52_P340073	NM_010111	Efnb2	1.00	-1.3	0.05	-1.7	0.00	-1.8
A_51_P333274	NM_013542	Gzmb	0.08	-1.8	0.02	-1.7	0.00	-1.8
A_55_P2129469	XM_918544	LOC641199	0.08	-2.0	0.00	-1.8	0.00	-1.8
A_51_P145662	NM_029465	Clec4g	1.00	-1.6	0.03	-1.9	0.01	-1.8
A_52_P381484	NM_133903	Spon2	1.00	-1.1	0.04	-2.3	0.17	-1.8
A_51_P372456	NM_001081441	Wdr86	1.00	-1.2	0.88	-1.1	0.04	-1.8
A_55_P1957433	NM_201245	Mprip	1.00	-1.2	0.46	-1.4	0.04	-1.8
A_55_P2170105			1.00	-1.1	0.70	-1.3	0.04	-1.8
A_55_P2168023	NM_001025379	Sema3g	1.00	-1.1	0.34	-1.5	0.03	-1.8
A_55_P2056325	NM_013470	Anxa3	1.00	-1.2	0.74	-1.3	0.03	-1.8
A_52_P114905	BC049666		1.00	-1.0	0.93	-1.1	0.03	-1.8
A_55_P1985984	NM_009609	Actg1	1.00	-1.1	0.91	-1.1	0.02	-1.8
A_55_P2266178	BC028446	AI790276	0.97	-1.5	0.07	-1.7	0.01	-1.8
A_51_P283473	NM_026271	Fibin	1.00	-1.5	0.18	-1.5	0.01	-1.8
A_30_P01027974			1.00	-1.3	0.73	-1.3	0.01	-1.8
A_55_P2047809	NM_175692	Snhg11	1.00	-1.3	0.49	-1.3	0.00	-1.8
A_55_P1974522	NM_175688	A530099J19Rik	0.19	-1.7	0.02	-1.6	0.00	-1.9
A_55_P2178137	NM_011889	41155.0	0.13	-1.7	0.02	-1.7	0.00	-1.9
A_55_P2004541	NM_001110323	Klra7	0.08	-1.7	0.00	-1.9	0.00	-1.9
A_55_P2036547	NM_153193	Hsd3b2	1.00	-1.3	0.05	-2.0	0.04	-1.9
A_52_P532456	NM_009538	Plagl1	1.00	-1.2	0.02	-2.1	0.03	-1.9
A_55_P2155848	NR_030683	Gm14207	1.00	-1.3	0.00	-2.1	0.00	-1.9
A_55_P2058783	NM_023516	2310016C08Rik	1.00	-1.1	0.00	-2.7	0.03	-1.9
A_51_P505868	NM_175386	Lhfp	1.00	-1.1	0.61	-1.4	0.04	-1.9
A_51_P444137	NM_001143765	Syce1	1.00	-1.5	0.33	-1.5	0.02	-1.9
A_55_P1965030	NM_001003915	Slc5a12	0.00	-1.8	0.00	-1.9	0.00	-2.0
A_55_P2004527	NM_010650	Klra8	1.00	-1.6	0.03	-1.9	0.00	-2.0
A_55_P2058023	U18569		1.00	-1.4	0.05	-2.0	0.03	-2.0
A_55_P2165790	NM_178706	Siglech	0.83	-1.7	0.00	-2.1	0.00	-2.0
A_55_P2151638	NM_013793	Klra15	0.78	-1.7	0.00	-2.2	0.01	-2.0
A_55_P2063465	NM_013691	Thbs3	1.00	-1.4	0.00	-2.4	0.07	-2.0
A_51_P439085	NM_023516	2310016C08Rik	1.00	-1.1	0.02	-2.6	0.04	-2.0
A_52_P308875	AK049998		1.00	-1.3	0.59	-1.4	0.04	-2.0

A_66_P107790	NM_026064	2900073G15Rik	1.00	-1.2	0.58	-1.4	0.04	-2.0
A_55_P1959763	NM_007826	Dach1	1.00	1.1	0.27	-1.7	0.03	-2.0
A_55_P2063312	NM_001166250	Mgll	1.00	-1.1	0.59	-1.4	0.03	-2.0
A_51_P477682	NM_008939	Prss12	1.00	-1.2	0.55	-1.5	0.02	-2.0
A_55_P2408588	NM_007489	Arntl	1.00	-1.4	0.30	-1.6	0.02	-2.0
A_55_P2136657	NM_013691	Thbs3	1.00	-1.4	0.00	-2.7	0.06	-2.1
A_52_P544043	NM_001163144	Pcsk5	1.00	-1.2	0.13	-1.5	0.00	-2.1
A_65_P07627	NM_007826	Dach1	1.00	-1.1	0.05	-1.7	0.00	-2.2
A_55_P2004526	NM_010650	Klra8	0.52	-1.9	0.00	-2.0	0.00	-2.2
A_55_P2403769	BB498095	AI481121	0.61	-1.7	0.00	-2.1	0.00	-2.2
A_55_P2126363	NM_011718	Wnt10b	1.00	-1.5	0.00	-2.4	0.00	-2.2
A_55_P2075070	NM_053190	S1pr5	0.61	-1.9	0.25	-1.6	0.01	-2.2
A_52_P514407	NM_013793	Klra15	0.26	-1.9	0.00	-2.3	0.02	-2.3
A_55_P2004536	NM_010649	Klra4	0.17	-2.0	0.00	-2.5	0.00	-2.3
A_51_P208931	NM_145974	C330016O10Rik	1.00	-1.0	0.24	-1.8	0.02	-2.3
A_55_P1969477	NM_011076	Abcb1a	1.00	-1.2	0.67	-1.4	0.01	-2.3
A_52_P139413	NM_001100462	Tmem221	1.00	-1.2	0.07	-1.7	0.00	-2.3
A_66_P118093	XM_001480420	LOC100048617	1.00	-1.4	0.00	-2.3	0.00	-2.4
A_51_P346641	BC025879		0.80	-1.7	0.00	-2.3	0.00	-2.4
A_52_P58145	NM_009022	Aldh1a2	1.00	1.1	0.98	1.0	0.03	-2.4
A_51_P267933	NM_175692	Snhg11	1.00	-1.5	0.38	-1.7	0.01	-2.4
A_51_P116651	NM_019759	Dpt	1.00	-1.2	0.90	-1.2	0.01	-2.4
A_55_P2028734	NM_013794	Klra16	0.08	-2.5	0.00	-3.0	0.00	-2.5
A_55_P1993404			1.00	-1.5	0.63	-1.6	0.04	-2.5
A_52_P566681	NM_153581	Gpm6a	1.00	-1.1	0.85	-1.3	0.03	-2.5
A_52_P638459	NM_013653	Ccl5	0.84	-2.0	0.02	-3.0	0.02	-2.7
A_55_P2135967	XM_979793	Gm7816	1.00	-1.7	0.50	-1.8	0.04	-2.7
A_55_P2087984	NM_001164671	Dnaja1	1.00	-1.7	0.46	-1.8	0.02	-2.7
A_55_P2170349	NM_053152	Klra22	0.00	-2.6	0.00	-3.0	0.00	-2.8
A_51_P175424	NM_011797	Car14	0.17	-2.4	0.00	-2.5	0.00	-3.4
A_51_P356055	NM_175012	Grp	0.00	-2.9	0.00	-3.6	0.00	-3.7
A_55_P2094060	NM_010370	Gzma	0.17	-4.4	0.00	-5.1	0.00	-4.2
A_55_P2011341	XM_001474162	LOC100045268	0.08	-4.2	0.00	-5.1	0.01	-4.7
A_55_P2068459	NM_010479	Hspa1a	1.00	-2.3	0.46	-2.2	0.00	-5.9

3.3.5 Chr Lung. Significant probe list. List of all significantly differentially expressed probes in at least 1 treatment group (FDR P ≤ 0.05, fold change ± 1.5) in response to sub-chronic oral exposure to 17.5, 50, and 150 mg/kg-bw/day chrysene in the lung. The list is sorted from highest to lowest fold change in the 150 mg/kg-bw/day treatment group.

			**17.5 mg/kg-bw/day**		**50 mg/kg-bw/day**		**150 mg/kg-bw/day**	
Agilent Probe	Accession Number	Gene Symbol	*FDR P value*	*Fold change*	*FDR P value*	*Fold change*	*FDR P value*	*Fold change*
A_51_P246854	NM_009606	Acta1	0.99	-1.2	0.99	2.5	0.04	15.2
A_55_P2152472	NM_001039594	Lce3a	0.99	-1.2	1.00	1.2	0.03	13.7
A_51_P353232	NM_009394	Tnnc2	0.98	-1.3	1.00	1.1	0.02	11.0
A_55_P2006296	NM_010662	Krt13	0.99	-1.2	1.00	1.1	0.02	10.9
A_51_P461040	NM_028798	Crct1	0.99	-1.2	1.00	1.2	0.03	10.7

A_51_P430423	NM_007398	Ada	0.99	-1.2	1.00	1.0	0.04	9.7
A_55_P2147280	NM_030679	Myh1	0.99	-1.1	1.00	1.3	0.02	9.4
A_55_P2142226	NM_001034870	Serpina3h	0.10	6.8	1.00	1.7	0.00	9.1
A_55_P2067342	XM_001478579	Gm9782	0.99	-1.2	1.00	1.2	0.04	8.8
A_51_P319180	NM_008475	Krt4	0.99	-1.1	1.00	1.2	0.03	8.6
A_51_P373393	NM_033175	Lce3c	0.99	-1.2	1.00	1.2	0.04	8.5
A_55_P2088375	NM_001163664	Tnnt3	0.99	-1.1	1.00	1.2	0.02	8.5
A_51_P499698	NM_026414	Asprv1	0.99	-1.2	1.00	1.2	0.02	8.4
A_66_P101930	NM_025501	Lce3b	0.99	-1.2	1.00	1.3	0.03	8.1
A_51_P495269	NM_008508	Lor	0.99	-1.1	1.00	1.3	0.03	7.9
A_52_P576720	NM_020036	Calm4	0.99	-1.2	1.00	1.1	0.04	7.7
A_51_P100997	NM_201363	Serpinb3c	0.99	-1.2	1.00	1.1	0.03	7.6
A_55_P1957733	NM_026415	2310002J15Rik	0.99	-1.2	1.00	1.1	0.04	7.5
A_52_P656699	NM_013456	Actn3	0.99	-1.1	1.00	1.3	0.03	7.4
A_52_P573336	NM_172205	Sbsn	0.98	1.3	1.00	1.6	0.00	6.9
A_30_P01021588			0.99	-1.2	1.00	1.0	0.04	6.5
A_51_P338072	NM_010855	Myh4	0.98	-1.2	1.00	1.1	0.04	6.4
A_55_P2165299	NM_175418	Mybpc1	1.00	-1.1	1.00	1.2	0.04	6.2
A_51_P379750	NM_021508	Myoz1	0.99	-1.2	1.00	1.2	0.04	6.1
A_51_P414208	NM_028622	Lce1c	0.99	-1.1	1.00	1.2	0.04	5.8
A_55_P1994418	NM_025634	2310042E22Rik	0.99	-1.2	1.00	1.1	0.04	5.5
A_55_P2135095	NM_028622	Lce1c	0.99	-1.2	1.00	1.2	0.04	5.5
A_66_P111426	NM_028625	Lce1a2	0.99	-1.1	1.00	1.3	0.03	5.4
A_51_P345699	NM_011620	Tnnt3	0.99	-1.2	1.00	1.2	0.04	5.2
A_51_P187602	NM_009257	Serpinb5	0.99	-1.1	1.00	1.1	0.03	5.0
A_52_P252180	NM_025984	Lce1a1	0.99	-1.2	1.00	1.1	0.04	4.9
A_52_P391110	NM_009109	Ryr1	1.00	-1.0	1.00	1.3	0.03	4.9
A_55_P2116978	NM_010889	Neb	1.00	-1.0	1.00	1.3	0.04	4.5
A_55_P2165414	NM_009243	Serpina1a	0.17	3.9	0.99	1.6	0.01	4.5
A_30_P01032042			1.00	-1.0	1.00	1.3	0.01	4.5
A_55_P2414524	AK003290	1110002E22Rik	0.99	-1.1	1.00	1.3	0.04	4.4
A_55_P2010301	NM_009245	Serpina1c	0.00	3.4	0.99	1.4	0.00	4.1
A_55_P1972034	XM_001476091	Muc16	0.63	2.3	0.80	3.6	0.03	4.1
A_55_P2046709			0.10	3.4	1.00	1.3	0.02	4.0
A_30_P01020116			1.00	-1.0	1.00	1.2	0.04	3.8
A_55_P1953143	NM_144783	Wt1	0.53	2.3	0.85	2.9	0.02	3.8
A_55_P2113857	NM_009247	Serpina1e	0.00	3.3	0.99	1.4	0.00	3.5
A_51_P210286	NM_007582	Cacng1	0.99	-1.1	1.00	1.2	0.04	3.5
A_52_P592909	NM_026384	Dgat2	0.97	-1.2	0.97	2.0	0.04	3.4
A_52_P460836	NM_177303	Lrrn4	0.54	2.2	0.79	3.2	0.04	3.3
A_51_P520849	NM_009144	Sfrp2	0.70	1.9	0.87	2.7	0.03	3.1
A_51_P390937	XM_984731	LOC675735	0.98	1.2	1.00	1.3	0.02	3.1
A_55_P2010298	NM_009246	Serpina1d	0.22	2.4	1.00	1.2	0.01	2.9
A_55_P1966774	XM_893705	Gm6930	0.29	2.6	1.00	1.0	0.04	2.8
A_55_P2010292	NM_009247	Serpina1e	0.29	2.0	1.00	1.2	0.02	2.7
A_66_P118124	NM_008557	Fxyd3	0.80	1.5	0.95	1.8	0.01	2.7
A_55_P2034067	NM_001085509	Myom3	1.00	-1.0	1.00	1.1	0.04	2.6
A_55_P2010066	NM_007601	Capn3	0.99	-1.1	1.00	1.2	0.03	2.6
A_55_P1993153	NM_024230	Smtnl1	1.00	-1.0	1.00	1.0	0.04	2.5

A_55_P1952915	NM_015825	Sh3bgr	0.99	1.1	1.00	1.1	0.04	2.5
A_55_P2276221	U76382	Gm5222	0.97	1.1	1.00	1.3	0.01	2.5
A_55_P2010312	NM_009243	Serpina1a	0.25	2.1	1.00	1.2	0.04	2.3
A_51_P485421	AK088666		0.99	1.1	1.00	1.1	0.03	2.3
A_55_P2107785	XM_001475876	Gm13105	0.82	1.5	0.88	2.0	0.04	2.2
A_55_P2179341	X63811		0.90	1.3	0.98	1.5	0.03	2.2
A_51_P188271	NM_054042	Cd248	0.95	1.2	0.97	1.5	0.02	2.2
A_55_P1953003	NM_080467	Atp6v0a4	0.97	1.1	0.94	1.6	0.00	2.2
A_51_P368210	NM_145981	Phyhip	0.99	1.1	1.00	-1.0	0.04	2.1
A_55_P2186978	U19317		0.95	1.2	0.97	1.5	0.04	2.1
A_66_P121117	NM_009246	Serpina1d	0.28	1.8	1.00	1.2	0.02	2.1
A_55_P2113703	NM_019866	Spib	0.95	1.2	1.00	1.1	0.02	2.1
A_55_P1957424	NM_172294	Sulf1	0.55	1.5	0.82	1.8	0.02	2.1
A_51_P412914	NM_010112	Efs	0.88	1.3	0.92	1.5	0.00	2.1
A_51_P110672	NM_009074	Mst1r	0.68	1.4	0.94	1.5	0.03	2.0
A_66_P101477	NM_031368	Bglap-rs1	1.00	1.0	1.00	1.1	0.03	2.0
A_51_P405397	NM_007899	Ecm1	0.95	1.2	1.00	1.2	0.03	2.0
A_52_P537545	NM_021491	Smpd3	0.95	1.2	0.99	1.3	0.04	1.9
A_51_P239766	NM_019676	Plcd1	0.95	1.2	0.99	1.3	0.03	1.9
A_55_P2130501	XM_001472813	LOC100044727	0.99	1.0	1.00	1.1	0.02	1.9
A_55_P2117119	NM_028889	Efhd1	0.28	1.7	0.97	1.4	0.02	1.9
A_51_P408649	NM_145463	Shisa2	0.91	1.2	0.88	1.4	0.00	1.9
A_55_P2002903	NM_022315	Smoc2	0.82	1.3	0.99	1.3	0.04	1.8
A_55_P2107155	NM_029182	Rasd2	0.91	1.2	0.99	1.3	0.04	1.8
A_51_P407323	NM_007976	F5	0.99	1.1	0.98	1.4	0.04	1.8
A_55_P2043430	AF144972		0.97	1.1	0.99	1.3	0.04	1.8
A_51_P483576	AF138742		0.98	1.1	1.00	1.1	0.03	1.8
A_55_P2262593	AK142388	4930429F24Rik	0.94	1.2	0.99	1.3	0.03	1.8
A_52_P313217	NM_001081342	Gpr133	0.95	1.1	0.97	1.3	0.04	1.7
A_51_P513776	XM_918237	LOC640979	0.97	1.1	1.00	-1.1	0.04	1.7
A_51_P507801	NM_028784	F13a1	1.00	-1.0	0.95	1.4	0.04	1.7
A_55_P2122830	NM_009549	Zfp185	0.89	1.2	0.94	1.4	0.03	1.7
A_55_P2087429	XM_001477552	LOC100047162	1.00	1.0	1.00	1.1	0.03	1.7
A_52_P531175	NM_013902	Fkbp3	0.97	1.1	0.99	1.2	0.03	1.7
A_51_P104768	XM_001476673	Igg2a	0.99	1.0	0.99	1.2	0.03	1.7
A_55_P2163363	XM_001480288	Clec2f	0.97	1.1	0.99	1.2	0.02	1.7
A_52_P267824	NM_197999	2210023G05Rik	1.00	1.0	1.00	1.1	0.01	1.7
A_55_P2104761			0.97	-1.1	1.00	-1.0	0.01	1.7
A_51_P426276	AF267660	Pdk2	0.90	1.2	1.00	1.2	0.04	1.6
A_55_P2102335	NM_033327	Zfp423	1.00	-1.0	1.00	1.2	0.04	1.6
A_55_P2162204	NM_146188	Kctd15	0.96	1.1	0.99	1.2	0.04	1.6
A_66_P113868	NM_007665	Cdh3	0.89	1.2	0.97	1.3	0.04	1.6
A_66_P105175	NM_009738	Bche	0.93	1.2	0.99	1.3	0.04	1.6
A_55_P1963463	NM_008067	Gabra3	0.89	1.2	0.87	1.5	0.03	1.6
A_55_P1966690	NM_021282	Cyp2e1	0.99	1.0	0.94	1.4	0.03	1.6
A_52_P314129	NM_008862	Pkia	0.94	1.1	0.99	1.2	0.02	1.6
A_51_P407657	U37911		0.92	1.1	1.00	1.1	0.02	1.6
A_55_P2112185	NM_173390	Nhsl1	0.95	1.1	0.89	1.4	0.04	1.5
A_55_P1959500	NM_172759	Ces5	0.99	1.0	1.00	1.0	0.04	1.5

A_55_P2124228	NM_028283	Uaca	0.94	1.1	1.00	1.1	0.04	1.5
A_30_P01017728			1.00	1.0	1.00	1.1	0.04	1.5
A_55_P2155620	NM_025282	Mef2c	0.96	1.1	0.99	1.2	0.02	1.5
A_55_P1968841			0.87	-1.2	1.00	-1.0	0.02	1.5
A_55_P2031979	NM_010656	Sspn	0.93	1.1	0.92	1.3	0.01	1.5
A_55_P1961099	NM_175029	Atg4c	0.98	1.0	0.77	1.4	0.00	1.5
A_55_P1989524	NM_001081416	Fndc1	0.78	1.1	1.00	1.1	0.00	1.5
A_55_P2367415	AK035805	A630026N12Rik	0.00	1.5	0.89	1.2	0.20	1.2
A_30_P01018165			0.00	-1.6	0.86	-1.4	0.12	-1.3
A_51_P123625	NM_008392	Irg1	0.66	-1.3	0.88	-1.4	0.04	-1.5
A_55_P2158962	NM_178118	Dixdc1	0.92	-1.1	0.99	-1.1	0.04	-1.5
A_66_P134690	NM_146043	Spin1	0.99	-1.0	0.99	-1.2	0.04	-1.5
A_55_P2161219	NM_001164805	Thsd7a	0.87	-1.2	0.94	-1.3	0.04	-1.5
A_55_P2125006	NM_026181	Gpatch1	0.83	-1.2	0.99	-1.2	0.04	-1.5
A_51_P416509	NM_030609	Hist1h1a	0.18	-1.5	0.68	-1.6	0.04	-1.5
A_55_P2039038	NM_001081750	Zfp664	0.94	-1.1	0.99	-1.2	0.04	-1.5
A_30_P01031631			0.85	-1.2	0.99	-1.2	0.04	-1.5
A_51_P388478	NM_010110	Efnb1	0.78	-1.2	0.92	-1.3	0.04	-1.5
A_55_P1963980	NM_026825	Lrrc16a	0.77	-1.2	0.92	-1.3	0.04	-1.5
A_55_P2085335	NM_019394	Mia1	0.82	-1.2	0.96	-1.3	0.04	-1.5
A_51_P464822	NM_015787	Hist1h1e	0.56	-1.3	0.86	-1.5	0.04	-1.5
A_30_P01021019			0.99	-1.0	0.99	-1.1	0.04	-1.5
A_30_P01024724			0.86	-1.2	0.68	-1.6	0.03	-1.5
A_51_P485458	NM_001005506	Txlna	0.96	-1.1	0.98	-1.2	0.03	-1.5
A_51_P144500	NM_016667	Sntb1	0.98	-1.1	0.99	-1.2	0.03	-1.5
A_51_P477364	NM_007483	Rhob	0.93	-1.1	0.89	-1.4	0.03	-1.5
A_30_P01028666			0.94	-1.1	0.95	-1.3	0.02	-1.5
A_55_P2063251	NM_028207	Dusp3	0.86	-1.2	1.00	-1.1	0.02	-1.5
A_55_P2171897	NM_008714	Notch1	0.78	-1.2	0.99	-1.1	0.02	-1.5
A_30_P01020476			0.53	-1.3	0.89	-1.4	0.02	-1.5
A_55_P2077866	NM_010918	Nktr	0.62	-1.3	0.95	-1.3	0.02	-1.5
A_52_P393306	NM_009222	Snap23	0.87	-1.2	0.97	-1.2	0.02	-1.5
A_51_P295192	NM_010907	Nfkbia	0.73	-1.2	0.94	-1.3	0.02	-1.5
A_55_P2076418	NM_001110327	Dmtf1	0.58	-1.3	0.92	-1.3	0.02	-1.5
A_55_P2037618	NM_001163502	C130039O16Rik	0.95	-1.1	0.97	-1.2	0.01	-1.5
A_30_P01020298			0.25	-1.3	0.79	-1.3	0.01	-1.5
A_51_P303424	NM_021334	Itgax	1.00	1.0	0.89	-1.3	0.01	-1.5
A_55_P2107207	AK009736		0.87	-1.1	0.80	-1.3	0.01	-1.5
A_30_P01022751			0.36	-1.3	0.79	-1.4	0.01	-1.5
A_55_P2072315	AK133410		0.43	-1.3	0.75	-1.5	0.01	-1.5
A_55_P2010321	AK146791		0.42	-1.3	0.99	-1.2	0.01	-1.5
A_55_P1955733	XM_001477303		0.42	-1.2	0.68	-1.4	0.01	-1.5
A_51_P161354	NM_144907	Sesn2	0.99	-1.0	0.99	-1.2	0.01	-1.5
A_30_P01020570			0.76	-1.2	0.90	-1.3	0.01	-1.5
A_30_P01028277			0.22	-1.3	0.77	-1.4	0.00	-1.5
A_55_P2134690	NM_001081112	Ankrd26	0.60	-1.2	0.90	-1.3	0.00	-1.5
A_30_P01022987			0.59	-1.2	0.68	-1.5	0.00	-1.5
A_55_P1987409	NM_173444	Nbeal1	0.92	-1.1	0.99	-1.2	0.00	-1.5
A_52_P180373	NM_010821	Mpeg1	0.95	-1.1	0.87	-1.3	0.00	-1.5

A_55_P2126627	NM_001081409	Phf20l1	0.89	-1.1	0.93	-1.3	0.00	-1.5
A_30_P01017999			0.28	-1.3	0.79	-1.3	0.00	-1.5
A_66_P118165	XM_001471574	LOC100038935	0.80	-1.1	0.68	-1.4	0.00	-1.5
A_55_P2158522	NM_183355	Pbx1	0.30	-1.3	0.81	-1.3	0.00	-1.5
A_55_P2193064	AK142114	6720473M08Rik	0.92	-1.1	0.89	-1.2	0.00	-1.5
A_30_P01028970			0.29	-1.2	0.77	-1.3	0.00	-1.5
A_55_P2021923	AB241120		0.48	-1.2	0.86	-1.3	0.00	-1.5
A_30_P01031585			0.85	-1.2	0.98	-1.2	0.00	-1.5
A_55_P2048660	XM_908118	LOC633654	0.22	-1.3	0.68	-1.4	0.00	-1.5
A_52_P311031	AK016943		0.58	-1.3	0.87	-1.4	0.00	-1.5
A_55_P1988260	XM_001479382	LOC100048847	0.29	-1.3	0.79	-1.3	0.00	-1.5
A_55_P2377156	BG076663	AA408251	0.69	-1.2	0.99	-1.1	0.00	-1.5
A_55_P2162472			0.25	-1.3	0.68	-1.4	0.00	-1.5
A_51_P444447	NM_007679	Cebpd	0.68	-1.3	0.90	-1.4	0.04	-1.6
A_55_P2081388	NM_008575	Mdm4	0.72	-1.3	0.83	-1.5	0.04	-1.6
A_55_P1960735	NM_011819	Gdf15	0.86	-1.2	0.90	-1.4	0.04	-1.6
A_30_P01019885			0.91	-1.2	0.92	-1.4	0.04	-1.6
A_55_P1953489	NM_001033550	Lrrc8b	0.86	-1.2	0.98	-1.3	0.04	-1.6
A_52_P351816	NM_010578	Itgb1	0.94	-1.1	0.98	-1.3	0.04	-1.6
A_55_P2018176	AK039146	AI504432	0.99	-1.0	0.99	-1.2	0.04	-1.6
A_55_P2143837	NM_007735	Col4a4	0.96	-1.1	1.00	-1.1	0.03	-1.6
A_55_P2105321	NM_023190	Acin1	0.72	-1.3	0.99	-1.2	0.03	-1.6
A_55_P2240823	NM_001128606	Epb4.1	0.82	-1.2	0.96	-1.3	0.03	-1.6
A_55_P2104917	NM_016902	Nphp1	0.56	-1.3	0.98	-1.2	0.02	-1.6
A_55_P2124553	NM_031998	Tsga14	0.70	-1.2	0.95	-1.3	0.02	-1.6
A_55_P2127174	NM_170759	Zfp628	0.89	-1.2	0.98	-1.2	0.02	-1.6
A_51_P267544	NM_013522	Frg1	0.80	-1.2	0.92	-1.4	0.01	-1.6
A_30_P01026230			0.57	-1.3	0.87	-1.4	0.01	-1.6
A_55_P2175752	NM_001081975	Mfap1b	0.12	-1.5	0.49	-1.6	0.01	-1.6
A_55_P2014978	NM_007889	Dvl3	0.22	-1.4	0.79	-1.4	0.01	-1.6
A_30_P01018146			0.38	-1.3	0.79	-1.4	0.00	-1.6
A_55_P1999818	XM_001472026	Gm2006	0.38	-1.3	0.79	-1.4	0.00	-1.6
A_30_P01032942			0.36	-1.3	0.82	-1.4	0.00	-1.6
A_30_P01025536			0.14	-1.4	0.77	-1.4	0.00	-1.6
A_52_P194316	NM_001128094	Atp13a3	0.79	-1.2	0.91	-1.3	0.00	-1.6
A_55_P2132800	AK041361		0.58	-1.2	0.79	-1.3	0.00	-1.6
A_55_P2133624	XM_001475193	Gm2891	0.27	-1.3	0.37	-1.5	0.00	-1.6
A_66_P127262	XM_985872	Gm8799	0.22	-1.3	0.68	-1.4	0.00	-1.6
A_30_P01022343			0.26	-1.3	0.77	-1.4	0.00	-1.6
A_55_P2018307	XM_001005025	LOC677576	0.31	-1.3	0.77	-1.4	0.00	-1.6
A_55_P2151209	NM_130873	Krtap16-4	0.22	-1.4	0.77	-1.4	0.00	-1.6
A_51_P459661	NM_021460	Lipa	0.95	-1.1	0.82	-1.4	0.00	-1.6
A_55_P2097518	NM_001080943	Zdhhc22	0.00	-2.6	0.68	-2.3	0.09	-1.7
A_52_P45738	NM_029979	Trim35	0.79	-1.3	0.99	-1.2	0.04	-1.7
A_55_P2126662	NM_007616	Cav1	0.96	-1.1	0.98	-1.3	0.01	-1.7
A_55_P1985259	NM_001017985	C2cd3	0.60	-1.3	0.93	-1.4	0.00	-1.7
A_30_P01032670			0.29	-1.3	0.55	-1.6	0.00	-1.7
A_30_P01024297			0.53	-1.3	0.79	-1.4	0.00	-1.7
A_55_P2304507	NM_172204	Noxa1	0.84	-1.3	0.79	-1.9	0.03	-1.8

A_55_P1974407	NM_011074	Cdk14	0.92	-1.2	0.99	-1.2	0.01	-1.8
A_55_P2073935	NM_013790	Abcc5	0.97	-1.1	0.97	-1.3	0.00	-1.8
A_55_P2085142	NM_009263	Spp1	0.57	-1.3	0.00	-1.8	0.00	-1.8
A_52_P667913	NM_033592	Pcdhga9	0.66	-1.4	0.99	-1.4	0.04	-1.9
A_30_P01032068			0.41	-1.5	0.79	-1.7	0.01	-1.9
A_51_P279693	NM_009992	Cyp1a1	0.00	-2.2	0.71	-1.9	0.01	-2.0
A_51_P484842	NM_007493	Asgr2	0.68	-1.4	0.90	-1.6	0.02	-2.0
A_55_P2056344	NM_001142539	Gm9992	0.00	-4.1	0.68	-3.1	0.10	-2.1
A_55_P2001494	NM_013598	Kitl	0.95	-1.2	0.99	-1.4	0.03	-2.2
A_55_P1990032	NM_009141	Cxcl5	0.49	-2.8	0.55	-6.6	0.02	-5.8

3.3.6 DBahA Lung. Significant probe list. List of all significantly differentially expressed probes in at least 1 treatment group (FDR P ≤ 0.05, fold change ± 1.5) in response to sub-chronic oral exposure to 6.25, 12.5, and 25 mg/kg-bw/day dibenz(ah)anthracene in the lung. The list is sorted from highest to lowest fold change in the 25 mg/kg-bw/day treatment group.

			**6.25 mg/kg-bw/day**		**12.5 mg/kg-bw/day**		**25 mg/kg-bw/day**	
Agilent Probe	Accession Number	Gene Symbol	*FDR P value*	*Fold change*	*FDR P value*	*Fold change*	*FDR P value*	*Fold change*
A_55_P1960735	NM_011819	Gdf15	0.00	6.1	0.00	9.1	0.00	11.6
A_51_P363947	NM_007669	Cdkn1a	0.00	4.4	0.00	6.3	0.00	7.1
A_55_P1986282	NM_001111099	Cdkn1a	0.00	4.0	0.00	4.7	0.00	6.7
A_51_P414396	NM_153127	Mmrn2	0.00	3.2	0.00	4.3	0.00	5.7
A_55_P1959500	NM_172759	Ces5	0.00	4.0	0.00	5.1	0.00	5.3
A_55_P2142226	NM_001034870	Serpina3h	0.86	1.2	0.00	4.5	0.00	5.3
A_55_P2032079	NM_016974	Dbp	0.00	3.3	0.00	5.1	0.00	5.1
A_55_P2005213	NM_145603	Ces2	0.00	2.7	0.00	3.8	0.00	5.0
A_55_P2032081	NM_016974	Dbp	0.00	3.0	0.00	4.6	0.00	4.8
A_55_P1972948	NM_176954	Brunol5	0.00	1.9	0.00	3.5	0.00	4.3
A_51_P329928	NM_013750	Phlda3	0.00	2.7	0.00	3.5	0.00	4.1
A_51_P487073	NM_138684	Wfdc12	0.03	1.8	0.00	3.2	0.00	4.1
A_52_P612803	NM_009831	Ccng1	0.00	2.6	0.00	3.7	0.00	3.8
A_55_P2107528			0.00	2.0	0.00	2.9	0.00	3.7
A_55_P2165414	NM_009243	Serpina1a	0.19	1.7	0.00	3.1	0.00	3.4
A_51_P204740	NM_133654	Cd34	0.06	1.6	0.00	2.8	0.00	3.4
A_52_P679105	NM_029614	Prss23	0.00	2.0	0.00	2.8	0.00	3.1
A_55_P1954835	XM_001475752	LOC100046186	0.00	1.8	0.00	2.2	0.00	3.1
A_52_P539310	NM_001160326	Serp2	0.00	1.7	0.00	2.4	0.00	3.1
A_55_P1965154	NM_025565	Spc25	0.00	2.7	0.00	2.9	0.00	3.0
A_55_P2095271	NM_153805	Pkn3	0.00	2.0	0.00	2.1	0.00	3.0
A_55_P2101340	NM_019511	Ramp3	0.00	1.7	0.00	1.9	0.00	2.9
A_55_P2010301	NM_009245	Serpina1c	0.25	1.6	0.01	2.7	0.00	2.9
A_30_P01019901			0.12	1.6	0.04	1.9	0.00	2.9
A_55_P1983773	NM_001012273	Birc5	0.00	2.6	0.00	3.0	0.00	2.8
A_30_P01018535			0.19	1.4	0.03	1.9	0.00	2.8
A_55_P2141860	NM_026531	Aen	0.00	2.0	0.00	2.5	0.00	2.7
A_55_P2145804	NM_026531	Aen	0.00	1.9	0.00	2.6	0.00	2.7
A_55_P2046709			0.25	1.5	0.00	2.6	0.00	2.7

A_30_P01032234			0.16	1.4	0.00	2.4	0.00	2.7
A_55_P2119257	NM_008871	Serpine1	0.07	1.5	0.00	2.3	0.00	2.7
A_55_P2173982	NM_009104	Rrm2	0.00	2.1	0.00	2.5	0.00	2.6
A_51_P326191	NM_009251	Serpina3g	0.00	2.1	0.00	3.4	0.00	2.6
A_55_P2031999	NM_145448	9030617O03Rik	0.00	1.8	0.00	2.2	0.00	2.6
A_55_P2092286	XM_001481225	LOC100048780	0.00	1.8	0.00	2.1	0.00	2.6
A_55_P2007713	NM_053082	Tspan4	0.00	1.7	0.00	2.0	0.00	2.6
A_55_P2113857	NM_009247	Serpina1e	0.16	1.6	0.01	2.4	0.00	2.6
A_55_P2015292	NM_008521	Ltc4s	0.33	1.3	0.01	2.2	0.00	2.6
A_52_P675395	NM_007722	Cxcr7	0.07	1.5	0.00	1.9	0.00	2.6
A_55_P1967291	NM_144818	Ncaph	0.00	2.3	0.00	2.4	0.00	2.5
A_51_P175580	NM_021897	Trp53inp1	0.00	2.0	0.00	2.5	0.00	2.5
A_30_P01027010			0.00	2.0	0.00	2.2	0.00	2.5
A_30_P01026536			0.00	2.0	0.00	2.4	0.00	2.5
A_55_P2137406	NM_007527	Bax	0.00	2.0	0.00	2.2	0.00	2.5
A_55_P2011146	NM_178608	Reep1	0.00	1.9	0.00	2.1	0.00	2.5
A_51_P433870	NM_009506	Vegfc	0.00	1.9	0.00	2.0	0.00	2.5
A_51_P323620	NM_144543	Thyn1	0.00	1.8	0.00	2.2	0.00	2.5
A_52_P311853	NM_030143	Ddit4l	0.01	1.7	0.00	2.2	0.00	2.5
A_55_P2039320	NM_178679	Zfp365	0.02	1.5	0.00	2.3	0.00	2.5
A_55_P1966774	XM_893705	Gm6930	0.67	1.2	0.00	2.8	0.00	2.5
A_30_P01026923			0.21	1.6	0.05	1.9	0.00	2.5
A_52_P249733	NM_011540	Tcap	0.89	1.1	0.50	1.5	0.00	2.5
A_51_P424959	NM_007528	Bcl6b	0.00	1.8	0.00	2.1	0.00	2.4
A_55_P2073377	NM_001081117	Mki67	0.00	2.5	0.00	2.6	0.00	2.3
A_51_P253803	NM_001081117	Mki67	0.00	2.5	0.00	2.4	0.00	2.3
A_51_P480328	NM_133222	Eltd1	0.00	2.0	0.00	2.0	0.00	2.3
A_55_P2104975	NM_001168294	Serpina3f	0.00	2.0	0.00	2.1	0.00	2.3
A_55_P2148534	BC096461	Nr1d2	0.00	1.9	0.00	2.4	0.00	2.3
A_52_P29953	NM_175398	6530418L21Rik	0.00	1.7	0.00	2.0	0.00	2.3
A_52_P686785	NM_053247	Lyve1	0.34	1.5	0.01	2.5	0.00	2.3
A_52_P536494	NM_008709	Mycn	0.00	1.4	0.00	1.7	0.00	2.3
A_51_P481920	NM_009828	Ccna2	0.00	2.2	0.00	2.3	0.00	2.2
A_52_P303891	NM_011584	Nr1d2	0.00	1.8	0.00	2.1	0.00	2.2
A_51_P350403	NM_009148	Exoc4	0.00	1.8	0.00	2.0	0.00	2.2
A_55_P2169227	NM_177716	AI836003	0.00	1.6	0.00	1.8	0.00	2.2
A_51_P415220	NM_009517	Zmat3	0.00	1.6	0.00	2.2	0.00	2.2
A_66_P111562	NM_007631	Ccnd1	0.01	1.5	0.00	2.0	0.00	2.2
A_51_P269203	NM_022017	Trpv4	0.32	1.4	0.02	2.0	0.00	2.2
A_52_P624434	NM_175526	Clec1a	0.07	1.6	0.01	1.9	0.00	2.2
A_51_P351896	NM_133187	Fam198b	0.18	1.5	0.04	1.8	0.00	2.2
A_30_P01028922			0.07	1.4	0.00	1.7	0.00	2.2
A_66_P118600	NM_008480	Lama1	0.00	2.2	0.01	1.9	0.00	2.1
A_51_P164014	NM_173762	Cenpe	0.00	1.9	0.00	2.1	0.00	2.1
A_30_P01020960			0.00	1.9	0.00	2.1	0.00	2.1
A_52_P151320	NM_025566	Tnfaip8l1	0.00	1.8	0.00	2.0	0.00	2.1
A_55_P2052062	NM_010818	Cd200	0.01	1.7	0.06	1.6	0.00	2.1
A_51_P369252	NM_001080995	4632434I11Rik	0.00	1.7	0.00	1.9	0.00	2.1
A_55_P2002578	NM_010145	Ephx1	0.00	1.7	0.00	2.0	0.00	2.1

A_55_P2018666	NM_009381	Thrsp	0.89	1.1	0.01	2.0	0.00	2.1
A_51_P514449	NM_030676	Nr5a2	0.19	1.4	0.00	1.9	0.00	2.1
A_55_P2009225	NM_133654	Cd34	0.17	1.4	0.00	1.9	0.00	2.1
A_55_P2039250	NM_026037	Mboat2	0.09	1.4	0.00	1.9	0.00	2.1
A_55_P2009752	NM_172563	Hlf	0.22	1.4	0.01	1.8	0.00	2.1
A_30_P01026613			0.30	1.3	0.02	1.7	0.00	2.1
A_52_P669005	NM_023624	Lrat	0.65	1.3	0.95	1.1	0.01	2.1
A_51_P111962	NM_001141922	mCG_21548	0.69	1.2	0.46	1.3	0.00	2.1
A_30_P01026797			0.22	1.3	0.18	1.4	0.00	2.1
A_55_P1988048	NM_001163522	Emcn	0.11	1.5	0.22	1.4	0.00	2.1
A_30_P01030802			0.09	1.3	0.03	1.4	0.00	2.1
A_55_P2103837	NM_008620	Gbp4	0.00	2.1	0.00	1.9	0.00	2.0
A_52_P162099	NM_001004140	Ckap2	0.00	2.1	0.00	1.9	0.00	2.0
A_52_P232637	NM_007857	Dhh	0.00	1.9	0.03	1.5	0.00	2.0
A_51_P270949	NM_020034	Hist1h1b	0.00	1.8	0.01	1.6	0.00	2.0
A_52_P136782	NM_009063	Rgs5	0.00	1.7	0.00	1.9	0.00	2.0
A_30_P01025790			0.00	1.7	0.00	2.1	0.00	2.0
A_52_P350554	NM_008420	Kcnb1	0.03	1.6	0.00	2.0	0.00	2.0
A_52_P418489	NM_023476	Tinagl1	0.02	1.6	0.01	1.7	0.00	2.0
A_51_P487813	NM_016753	Lxn	0.01	1.6	0.00	1.8	0.00	2.0
A_55_P1976584	NM_001038710	Tmod2	0.01	1.6	0.01	1.7	0.00	2.0
A_55_P1986833	NM_054040	Tulp4	0.00	1.6	0.00	1.9	0.00	2.0
A_55_P2269819	NM_183187	Fam107a	0.01	1.5	0.00	1.8	0.00	2.0
A_55_P2035286	NM_010931	Uhrf1	0.00	1.5	0.00	1.9	0.00	2.0
A_55_P2078365	NM_173402	Rgs12	0.03	1.4	0.00	1.7	0.00	2.0
A_55_P2006008	NM_025429	Serpinb1a	0.28	1.3	0.04	1.6	0.00	2.0
A_55_P2017977	NM_001033266	Gm525	0.06	1.4	0.20	1.3	0.00	2.0
A_51_P455897	NM_144526	Fam64a	0.00	2.0	0.00	1.9	0.00	1.9
A_52_P627068	NM_170593	Disp2	0.05	1.6	0.01	2.0	0.00	1.9
A_30_P01025511			0.01	1.6	0.01	1.8	0.00	1.9
A_55_P2094925	NM_011157	Srgn	0.01	1.6	0.04	1.4	0.00	1.9
A_66_P106789	XM_894271	Gm6970	0.01	1.6	0.04	1.5	0.00	1.9
A_55_P2084631	NM_178184	Hist1h2an	0.00	1.6	0.00	1.6	0.00	1.9
A_51_P113178	NM_175398	6530418L21Rik	0.00	1.6	0.00	1.9	0.00	1.9
A_55_P2099961	NM_178186	Hist1h2ag	0.00	1.6	0.00	1.7	0.00	1.9
A_55_P2140941	NM_023476	Tinagl1	0.00	1.6	0.13	1.4	0.00	1.9
A_51_P161354	NM_144907	Sesn2	0.00	1.6	0.00	1.9	0.00	1.9
A_55_P2187076	NM_011430	Sncg	0.00	1.6	0.00	1.9	0.00	1.9
A_51_P401683	NM_145375	Tm6sf1	0.00	1.6	0.00	1.7	0.00	1.9
A_66_P138053	AK044848		0.03	1.5	0.03	1.5	0.00	1.9
A_55_P1979893	NM_017376	Tef	0.03	1.5	0.00	1.7	0.00	1.9
A_66_P120125	NM_053078	D0H4S114	0.03	1.5	0.00	1.8	0.00	1.9
A_30_P01030486			0.03	1.5	0.09	1.4	0.00	1.9
A_51_P329332	NM_054087	Slc19a2	0.01	1.5	0.00	1.8	0.00	1.9
A_51_P246903	NM_026467	Rps27l	0.01	1.5	0.00	1.7	0.00	1.9
A_52_P498208	NM_178183	Hist1h2ak	0.00	1.5	0.01	1.6	0.00	1.9
A_30_P01029956			0.00	1.5	0.00	1.7	0.00	1.9
A_51_P372550	NM_026770	Cgref1	0.95	1.0	0.05	1.7	0.00	1.9
A_52_P198898	NM_177271	Samd5	0.22	1.4	0.04	1.7	0.00	1.9

A_66_P107038	NM_028243	Prcp	0.55	1.2	0.00	1.7	0.00	1.9
A_55_P2071858	NM_008598	Mgmt	0.49	1.2	0.02	1.6	0.00	1.9
A_55_P2034027	NM_008713	Nos3	0.09	1.3	0.00	1.6	0.00	1.9
A_51_P270184	NM_025811	Nhlrc2	0.08	1.4	0.02	1.5	0.00	1.9
A_30_P01018099			0.14	1.3	0.01	1.5	0.00	1.9
A_52_P87839	NM_013657	Sema3c	0.99	1.0	0.11	1.4	0.00	1.9
A_51_P461429	NM_007825	Cyp7b1	0.74	1.1	0.21	1.4	0.00	1.9
A_52_P111031	NM_001013753	Pcdh17	0.28	1.4	0.19	1.6	0.00	1.9
A_51_P360492	NM_008567	Mcm6	0.18	1.5	0.12	1.6	0.00	1.9
A_51_P230098	NM_023209	Pbk	0.00	1.9	0.00	1.7	0.00	1.8
A_55_P1996946	NM_023223	Cdc20	0.00	1.8	0.00	1.8	0.00	1.8
A_51_P133137	NM_009004	Kif20a	0.00	1.7	0.00	1.8	0.00	1.8
A_51_P103594	NM_029894	Fam162b	0.01	1.6	0.05	1.5	0.00	1.8
A_55_P2004801	NM_001040435	Tacc3	0.00	1.6	0.00	1.7	0.00	1.8
A_52_P55772	NM_009325	Tbxa2r	0.04	1.5	0.06	1.5	0.00	1.8
A_55_P2109505	XM_890094	Gm6594	0.02	1.5	0.01	1.6	0.00	1.8
A_55_P2036007	NM_198409	Rai2	0.02	1.5	0.05	1.5	0.00	1.8
A_55_P2037812	NM_023245	Palmd	0.01	1.5	0.01	1.5	0.00	1.8
A_55_P2102335	NM_033327	Zfp423	0.01	1.5	0.00	1.6	0.00	1.8
A_52_P209484	NM_025915	Tmem88	0.01	1.5	0.01	1.6	0.00	1.8
A_51_P276479	NM_178098	4930486L24Rik	0.01	1.5	0.00	1.7	0.00	1.8
A_51_P179697	NM_026884	Fam57b	0.01	1.5	0.00	1.6	0.00	1.8
A_55_P1960049	NM_027419	2810408A11Rik	0.01	1.5	0.01	1.6	0.00	1.8
A_55_P2470474	AK087205	9530082P21Rik	0.00	1.5	0.00	1.6	0.00	1.8
A_55_P2002577	NM_010145	Ephx1	0.00	1.5	0.00	1.6	0.00	1.8
A_55_P2110245	NM_001163359	Fignl1	0.00	1.5	0.00	1.7	0.00	1.8
A_51_P110471	NM_026993	Ddah1	0.06	1.6	0.01	1.7	0.00	1.8
A_55_P2008936	NM_001102414	Slc2a9	0.18	1.3	0.00	1.7	0.00	1.8
A_55_P2114779	NM_015764	Greb1	0.35	1.3	0.05	1.6	0.00	1.8
A_51_P139651	NM_008713	Nos3	0.05	1.4	0.01	1.6	0.00	1.8
A_55_P1992084	NM_008981	Ptprg	0.02	1.3	0.00	1.6	0.00	1.8
A_66_P122086	NM_001039720	9030619P08Rik	0.01	1.4	0.00	1.6	0.00	1.8
A_55_P2175284	NM_007915	Ei24	0.01	1.4	0.00	1.6	0.00	1.8
A_55_P1953728	NM_016701	Nes	0.13	1.3	0.01	1.5	0.00	1.8
A_55_P2129316			0.00	1.4	0.00	1.5	0.00	1.8
A_52_P90363	NM_029803	Ifi27l2a	0.99	1.0	0.20	1.7	0.04	1.8
A_55_P1983769	NM_001012273	Birc5	0.37	1.4	0.53	1.3	0.01	1.8
A_55_P2143025	NM_013657	Sema3c	0.99	-1.0	0.28	1.4	0.00	1.8
A_55_P2103452	NM_010934	Npy1r	0.30	1.3	0.21	1.4	0.00	1.8
A_30_P01029287			0.37	1.2	0.21	1.4	0.00	1.8
A_51_P390804	NM_016873	Wisp2	0.29	1.3	0.52	1.3	0.00	1.8
A_30_P01031906			0.21	1.3	0.41	1.3	0.00	1.8
A_66_P136186	NM_009516	Wee1	0.13	1.4	0.06	1.5	0.00	1.8
A_51_P367780	NM_029981	Adamtsl2	0.21	1.4	0.11	1.5	0.00	1.8
A_30_P01021307			0.15	1.4	0.50	1.2	0.00	1.8
A_55_P2060922	NM_153131	Unc5a	0.02	2.7	0.29	1.9	0.21	1.7
A_55_P2056654	NM_145588	Kif22	0.00	1.9	0.00	1.9	0.00	1.7
A_55_P1988083	NM_145150	Prc1	0.00	1.9	0.00	1.7	0.00	1.7
A_51_P240453	NM_133851	Nusap1	0.00	1.9	0.00	1.8	0.00	1.7

A_55_P1990067	XM_001478394	Gm9525	0.00	1.7	0.00	1.8	0.00	1.7
A_55_P2063146			0.00	1.7	0.00	1.7	0.00	1.7
A_51_P369200	NM_028109	Tpx2	0.00	1.7	0.00	1.6	0.00	1.7
A_51_P204402	NM_011369	Shcbp1	0.00	1.7	0.00	1.7	0.00	1.7
A_30_P01024344			0.04	1.6	0.17	1.4	0.00	1.7
A_51_P169693	NM_198095	Bst2	0.01	1.6	0.02	1.7	0.00	1.7
A_55_P2003813	NM_153522	Scn3b	0.01	1.6	0.00	1.8	0.00	1.7
A_66_P134542	NM_028390	Anln	0.00	1.6	0.00	1.8	0.00	1.7
A_30_P01020075			0.00	1.6	0.00	1.7	0.00	1.7
A_55_P2288232	AK018137	6330407I18Rik	0.00	1.6	0.00	1.7	0.00	1.7
A_66_P101835	NM_007646	Cd38	0.00	1.6	0.01	1.5	0.00	1.7
A_55_P2048588	NM_007659	Cdk1	0.00	1.6	0.00	1.7	0.00	1.7
A_55_P2087087	NM_001032378	Pecam1	0.02	1.5	0.01	1.6	0.00	1.7
A_30_P01025143			0.01	1.5	0.00	1.6	0.00	1.7
A_55_P2095311	XM_001475753	Ly6g	0.00	1.5	0.06	1.4	0.00	1.7
A_52_P220810	NM_144551	Trib2	0.00	1.5	0.00	1.6	0.00	1.7
A_55_P2006327	NM_008981	Ptprg	0.00	1.5	0.00	1.6	0.00	1.7
A_55_P1973906	NM_021897	Trp53inp1	0.00	1.5	0.00	1.7	0.00	1.7
A_55_P2018847	NM_001164735	Crlf2	0.00	1.5	0.01	1.5	0.00	1.7
A_55_P2062543	NM_053173	Kifc1	0.00	1.5	0.00	1.5	0.00	1.7
A_55_P2107155	NM_029182	Rasd2	0.14	1.5	0.00	2.0	0.01	1.7
A_55_P2010298	NM_009246	Serpina1d	0.53	1.2	0.01	1.9	0.01	1.7
A_55_P2161450	NM_173024	Serpina3b	0.54	1.2	0.00	1.8	0.00	1.7
A_51_P231320	NM_008611	Mmp8	0.09	1.3	0.00	1.8	0.00	1.7
A_55_P2024704	NM_013494	Cpe	0.87	1.1	0.05	1.7	0.01	1.7
A_51_P336721	NM_025372	Tipin	0.16	1.4	0.01	1.7	0.00	1.7
A_55_P2076866	NM_001162977	Megf6	0.00	1.4	0.00	1.7	0.00	1.7
A_55_P1998001	NM_001025384	DXBay18	0.09	1.4	0.00	1.7	0.00	1.7
A_52_P89567	NM_007483	Rhob	0.04	1.4	0.01	1.6	0.00	1.7
A_55_P1987914	NM_008696	Map4k4	0.00	1.4	0.00	1.6	0.00	1.7
A_51_P367310	NM_028083	Chaf1b	0.06	1.4	0.00	1.6	0.00	1.7
A_66_P119376	NM_177715	Kctd12	0.00	1.4	0.00	1.6	0.00	1.7
A_55_P1975415	NM_146067	Cpped1	0.01	1.3	0.00	1.6	0.00	1.7
A_51_P290576	NM_152804	Plk2	0.01	1.4	0.00	1.6	0.00	1.7
A_55_P2084656	NM_175659	Hist1h2ah	0.07	1.4	0.05	1.5	0.00	1.7
A_55_P1954221	NM_010128	Emp1	0.31	1.3	0.03	1.5	0.00	1.7
A_55_P2084652	NM_178183	Hist1h2ak	0.04	1.4	0.01	1.5	0.00	1.7
A_55_P2002122	NM_173402	Rgs12	0.05	1.4	0.01	1.5	0.00	1.7
A_55_P2405784	AK141429	BC023202	0.04	1.4	0.01	1.5	0.00	1.7
A_55_P1975475	NM_010786	Mdm2	0.07	1.3	0.01	1.5	0.00	1.7
A_55_P2054854	NM_026639	Art4	0.06	1.4	0.01	1.5	0.00	1.7
A_55_P2408355	AK037728	BB166591	0.28	1.3	0.01	1.5	0.00	1.7
A_55_P2116465	NM_008981	Ptprg	0.04	1.4	0.00	1.5	0.00	1.7
A_51_P413785	NM_147778	Commd3	0.00	1.4	0.00	1.5	0.00	1.7
A_55_P1961499	NM_010741	Ly6c1	0.02	1.3	0.00	1.5	0.00	1.7
A_66_P128384	NM_008228	Hdac1	0.60	1.3	1.00	-1.0	0.04	1.7
A_55_P2025038	NM_013494	Cpe	0.73	1.2	0.17	1.5	0.02	1.7
A_52_P469789	NM_007588	Calcr	0.45	1.3	0.12	1.6	0.01	1.7
A_30_P01026973			0.35	1.2	0.13	1.4	0.00	1.7

A_51_P426270	NM_008597	Mgp	0.59	1.2	0.23	1.4	0.00	1.7
A_30_P01023651			0.10	1.3	0.06	1.3	0.00	1.7
A_51_P259975	NM_023113	Aspa	0.91	1.1	0.70	1.1	0.00	1.7
A_55_P1993789	NM_016957	Hmgn2	0.06	1.5	0.38	1.3	0.00	1.7
A_51_P159453	NM_009252	Serpina3n	0.16	1.4	0.15	1.4	0.00	1.7
A_51_P468464	NM_201352	Gdpd5	0.12	1.4	0.21	1.4	0.00	1.7
A_52_P587738	NM_008773	P2ry2	0.42	1.3	0.07	1.6	0.00	1.7
A_55_P1985591	NM_024170	Cxx1a	0.07	1.4	0.06	1.5	0.00	1.7
A_65_P07627	NM_007826	Dach1	0.07	1.6	0.30	1.4	0.00	1.7
A_52_P264790	NM_027924	Pdgfd	0.05	1.4	0.12	1.4	0.00	1.7
A_55_P2137421	NM_022017	Trpv4	0.23	1.3	0.06	1.5	0.00	1.7
A_51_P124254	NM_009931	Col4a1	0.10	1.5	0.07	1.6	0.00	1.7
A_55_P2083988	NM_008505	Lmo2	0.48	1.2	0.46	1.2	0.00	1.7
A_51_P220343	NM_018865	Wisp1	0.53	1.2	0.07	1.5	0.00	1.7
A_55_P1974028	NM_138673	Stab2	0.04	1.4	0.10	1.4	0.00	1.7
A_55_P2064771	NM_010741	Ly6c1	0.07	1.4	0.06	1.4	0.00	1.7
A_51_P354706	NM_010094	Lefty1	0.07	1.4	0.02	1.4	0.00	1.7
A_51_P383194	NM_008804	Pde9a	0.00	1.4	0.00	1.4	0.00	1.7
A_55_P2355330	NM_145613	C1qtnf5	0.00	1.4	0.41	1.2	0.00	1.7
A_55_P1953402	NM_029993	Mlana	0.62	1.2	0.47	1.2	0.00	1.7
A_55_P2057936	NM_001135115	Gm12250	0.01	2.2	0.14	1.8	0.16	1.6
A_52_P627269	NM_198171	BC015286	0.00	2.0	0.00	1.7	0.00	1.6
A_52_P327664	NM_153564	Gbp5	0.01	1.8	0.03	1.7	0.03	1.6
A_51_P451151	NM_026785	Ube2c	0.00	1.8	0.00	1.7	0.00	1.6
A_55_P2168628	NM_133678	Sac3d1	0.00	1.6	0.00	1.6	0.00	1.6
A_51_P487999	NM_028232	Sgol1	0.00	1.6	0.02	1.5	0.00	1.6
A_52_P281145	NM_172872	Kank4	0.00	1.6	0.13	1.4	0.00	1.6
A_55_P2124712	NM_145603	Ces2	0.00	1.6	0.00	1.6	0.00	1.6
A_55_P1976204	U09507		0.00	1.6	0.00	1.6	0.00	1.6
A_51_P457528	NM_007630	Ccnb2	0.00	1.6	0.00	1.6	0.00	1.6
A_51_P155142	NM_026560	Cdca8	0.00	1.6	0.01	1.6	0.00	1.6
A_55_P1960216	NM_009842	Cd151	0.02	1.5	0.00	1.8	0.00	1.6
A_30_P01026167			0.01	1.5	0.00	1.6	0.00	1.6
A_55_P2108708	NM_020574	Kcne3	0.01	1.5	0.06	1.5	0.00	1.6
A_30_P01026752			0.00	1.5	0.00	1.7	0.00	1.6
A_55_P2096867	NM_008083	Gap43	0.00	1.5	0.01	1.5	0.00	1.6
A_30_P01019123			0.00	1.5	0.00	1.6	0.00	1.6
A_55_P1994939	NM_008252	Hmgb2	0.00	1.5	0.00	1.6	0.00	1.6
A_51_P227004	NM_016904	Cks1b	0.00	1.5	0.00	1.6	0.00	1.6
A_51_P382152	NM_011171	Procr	0.66	1.2	0.03	1.9	0.05	1.6
A_55_P2065671	NM_172301	Ccnb1	0.01	1.4	0.00	1.7	0.00	1.6
A_55_P2000533	NM_012048	Polk	0.00	1.4	0.00	1.7	0.00	1.6
A_52_P322181	NM_007419	Adrb1	0.01	1.4	0.00	1.7	0.00	1.6
A_55_P2018017	NM_009425	Tnfsf10	0.06	1.4	0.00	1.7	0.00	1.6
A_55_P2151006	NM_001135172	C1qtnf7	0.06	1.5	0.05	1.6	0.01	1.6
A_55_P2040245	NM_001039485	Fam38b	0.16	1.4	0.05	1.6	0.01	1.6
A_51_P307168	NM_026993	Ddah1	0.08	1.5	0.04	1.6	0.01	1.6
A_55_P1994194	NM_001159628	Heph	0.27	1.3	0.04	1.6	0.00	1.6
A_52_P501816	NM_020049	Slc6a14	0.11	1.3	0.01	1.6	0.00	1.6

A_52_P577729	NM_175388	Rnf169	0.00	1.4	0.00	1.6	0.00	1.6
A_51_P502082	NM_009103	Rrm1	0.09	1.3	0.00	1.6	0.00	1.6
A_55_P1965298	NM_001099302	Gm5640	0.31	1.2	0.00	1.6	0.00	1.6
A_51_P301636	NM_001109685	9030409G11Rik	0.05	1.4	0.00	1.6	0.00	1.6
A_51_P367866	NM_007913	Egr1	0.24	1.3	0.04	1.5	0.00	1.6
A_30_P01030372			0.02	1.4	0.04	1.5	0.00	1.6
A_55_P1984690	NM_011217	Ptprr	0.08	1.4	0.03	1.5	0.00	1.6
A_55_P2103706	XM_485921	Gm5593	0.04	1.4	0.03	1.5	0.00	1.6
A_51_P148105	NM_011234	Rad51	0.05	1.4	0.03	1.5	0.00	1.6
A_55_P1980262	NM_001018063	Cxx1b	0.11	1.3	0.02	1.5	0.00	1.6
A_55_P2336558	NM_001163640	Chn2	0.25	1.2	0.01	1.5	0.00	1.6
A_51_P140641	NM_145570	Fam176a	0.29	1.2	0.01	1.5	0.00	1.6
A_55_P2031979	NM_010656	Sspn	0.20	1.2	0.01	1.5	0.00	1.6
A_55_P2177154	NM_178200	Hist1h2bm	0.08	1.3	0.01	1.5	0.00	1.6
A_55_P2320313	BC058714	AI852064	0.01	1.4	0.01	1.5	0.00	1.6
A_55_P2133255	NM_019499	Mad2l1	0.00	1.4	0.00	1.5	0.00	1.6
A_30_P01019169			0.00	1.4	0.00	1.5	0.00	1.6
A_51_P393426	NM_011173	Pros1	0.00	1.4	0.00	1.5	0.00	1.6
A_55_P2090359	NM_148937	Plcd4	0.28	1.4	0.14	1.6	0.04	1.6
A_55_P2035424	NM_008278	Hpgd	0.26	1.4	0.77	1.2	0.04	1.6
A_55_P1969477	NM_011076	Abcb1a	0.45	1.3	0.62	1.2	0.03	1.6
A_52_P5891	NM_029658	Fam101b	0.11	1.4	0.10	1.5	0.02	1.6
A_55_P2176792	NM_017400	Sh3gl3	0.65	1.2	0.22	1.4	0.02	1.6
A_51_P392687	NM_011701	Vim	0.40	1.3	0.12	1.6	0.02	1.6
A_55_P2349148	AK017909	5830408B19Rik	0.37	1.3	0.20	1.4	0.01	1.6
A_55_P2151011	BC090967	C1qtnf7	0.37	1.3	0.33	1.4	0.01	1.6
A_51_P342877	NM_011322	Scn1b	0.25	1.3	0.08	1.5	0.01	1.6
A_30_P01032002			0.06	1.5	0.20	1.4	0.01	1.6
A_52_P211956	NM_025658	Ms4a4d	0.13	1.5	0.86	1.1	0.01	1.6
A_66_P117730	NM_013500	Hapln1	0.58	1.2	0.21	1.4	0.01	1.6
A_55_P2082688	NM_021515	Ak1	0.95	-1.0	0.25	1.3	0.01	1.6
A_30_P01026626			0.20	1.3	0.49	1.2	0.00	1.6
A_55_P1967538	NM_015755	Hunk	0.03	1.4	0.17	1.3	0.00	1.6
A_55_P2154027	NM_008968	Ptgis	0.09	1.3	0.09	1.4	0.00	1.6
A_55_P2393021	AK053260	A430106G13Rik	0.04	1.4	0.08	1.4	0.00	1.6
A_51_P185906	NM_025659	Abi3	0.01	1.4	0.01	1.4	0.00	1.6
A_52_P227267	NM_178405	Atp1a2	0.02	1.4	0.09	1.4	0.00	1.6
A_51_P233160	NM_027309	Lysmd2	0.01	1.3	0.00	1.4	0.00	1.6
A_66_P104815	NM_007899	Ecm1	0.31	1.3	0.63	1.2	0.00	1.6
A_55_P2142232	XM_893705	Gm6930	0.70	1.1	0.20	1.3	0.00	1.6
A_30_P01028961			0.02	1.3	0.01	1.4	0.00	1.6
A_51_P218975	NM_173417	Kcns3	0.05	1.4	0.44	1.2	0.00	1.6
A_55_P1995173	NM_013614	Odc1	0.82	1.1	0.07	1.4	0.00	1.6
A_51_P355753	NM_010430	Hic1	0.25	1.3	0.20	1.3	0.00	1.6
A_51_P170807	AB021861	Map3k6	0.33	1.3	0.30	1.3	0.00	1.6
A_55_P2133266	NM_028375	Cxx1c	0.13	1.3	0.09	1.4	0.00	1.6
A_55_P2170813	NM_001164053	Pkig	0.89	1.1	0.17	1.3	0.00	1.6
A_30_P01017458			0.19	1.3	0.36	1.2	0.00	1.6
A_51_P474454	NM_026001	Rnaseh2b	0.03	1.4	0.04	1.4	0.00	1.6

A_51_P516133	NM_015786	Hist1h1c	0.68	1.1	0.14	1.4	0.00	1.6
A_55_P2112355	XM_980525	Gm7125	0.06	1.4	0.27	1.3	0.00	1.6
A_52_P222350	NM_134080	Flnb	0.09	1.4	0.10	1.4	0.00	1.6
A_55_P1972040	NM_015760	Nox4	0.12	1.4	0.08	1.4	0.00	1.6
A_55_P2055819	NM_001037722	Adam15	0.57	1.2	0.35	1.3	0.00	1.6
A_51_P170641	NM_030246	Dcaf4	0.08	1.3	0.02	1.4	0.00	1.6
A_55_P2028496	XM_001472085	Hmgn2l6	0.01	1.4	0.04	1.4	0.00	1.6
A_51_P284426	NM_030137	Cstad	0.71	1.1	0.24	1.3	0.00	1.6
A_52_P106709	NM_009027	Rasgrf2	0.06	1.3	0.04	1.4	0.00	1.6
A_52_P148678	AK169695		0.02	1.3	0.01	1.4	0.00	1.6
A_55_P1970105	NM_013589	Ltbp2	0.27	1.3	0.19	1.4	0.00	1.6
A_52_P193925	NM_028072	Sulf2	0.84	1.1	0.06	1.4	0.00	1.6
A_51_P109258	NM_138686	Cys1	0.30	1.3	0.10	1.5	0.00	1.6
A_51_P330213	NM_024184	Asf1b	0.00	1.4	0.01	1.4	0.00	1.6
A_55_P2181288	NM_025800	Ppp1r2	0.33	1.2	0.13	1.4	0.00	1.6
A_30_P01025359			0.00	2.4	0.27	1.7	0.28	1.5
A_51_P463846	NM_145545	Gbp6	0.00	1.8	0.04	1.6	0.01	1.5
A_55_P1990633	NM_001146275	Iigp1	0.01	1.7	0.05	1.6	0.05	1.5
A_52_P354373	XM_001481164	1190002F15Rik	0.00	1.7	0.00	1.6	0.00	1.5
A_55_P1988228	NM_009791	Aspm	0.00	1.6	0.03	1.5	0.01	1.5
A_55_P2127702	NM_012025	Racgap1	0.00	1.6	0.00	1.6	0.00	1.5
A_55_P2062598	NM_178609	E2f7	0.00	1.6	0.00	1.6	0.00	1.5
A_55_P1996941	NM_026785	Ube2c	0.00	1.6	0.00	1.6	0.00	1.5
A_51_P516870	NM_008409	Itm2a	0.03	1.5	0.13	1.4	0.00	1.5
A_55_P1978465	NM_023124	H2-Q8	0.03	1.5	0.15	1.4	0.02	1.5
A_51_P416822	NM_020259	Hhip	0.02	1.5	0.01	1.6	0.00	1.5
A_55_P1954277	NM_080595	Emid1	0.02	1.5	0.04	1.5	0.01	1.5
A_51_P100327	NM_013683	Tap1	0.02	1.5	0.21	1.3	0.00	1.5
A_52_P30989	NM_028222	Cdkn3	0.01	1.5	0.02	1.4	0.00	1.5
A_55_P1953919	NM_181407	Me3	0.01	1.5	0.00	1.7	0.00	1.5
A_51_P252859	NM_010516	Cyr61	0.01	1.5	0.13	1.4	0.01	1.5
A_55_P2109717	NM_183046	Kif20b	0.01	1.5	0.03	1.5	0.00	1.5
A_51_P212420	NM_010681	Lama4	0.01	1.5	0.00	1.6	0.00	1.5
A_51_P202074	NM_146171	Ncapd2	0.00	1.5	0.00	1.6	0.00	1.5
A_52_P139650	NM_025581	Ska1	0.00	1.5	0.01	1.5	0.00	1.5
A_55_P1964078	XM_001479553	Gm4248	0.00	1.5	0.00	1.6	0.00	1.5
A_30_P01018593			0.00	1.5	0.01	1.4	0.00	1.5
A_51_P394558	NM_001162943	Dchs1	0.00	1.5	0.01	1.5	0.00	1.5
A_51_P386344	NM_172742	Mtmr10	0.00	1.5	0.02	1.4	0.00	1.5
A_51_P151902	NM_145584	Spon1	0.00	1.5	0.04	1.4	0.00	1.5
A_51_P300572	NM_009579	Slc30a1	0.00	1.5	0.00	1.5	0.00	1.5
A_51_P125135	NM_026410	Cdca5	0.00	1.5	0.01	1.5	0.00	1.5
A_55_P1980636	NM_011497	Aurka	0.00	1.5	0.00	1.5	0.00	1.5
A_55_P2024155	NM_001033324	Zbtb16	0.73	1.2	0.00	2.6	0.15	1.5
A_55_P2142222	NM_001034870	Serpina3h	0.94	-1.0	0.03	1.7	0.02	1.5
A_55_P2061495	NM_016904	Cks1b	0.01	1.4	0.00	1.7	0.00	1.5
A_51_P255456	NM_009994	Cyp1b1	0.33	1.2	0.02	1.6	0.01	1.5
A_30_P01023370			0.01	1.3	0.00	1.6	0.00	1.5
A_30_P01020899			0.13	1.3	0.00	1.6	0.00	1.5

A_55_P2012960	NM_024198	Gpx7	0.10	1.3	0.00	1.6	0.00	1.5
A_55_P1953920	NM_181407	Me3	0.02	1.4	0.00	1.6	0.00	1.5
A_55_P1968664			0.00	1.4	0.00	1.6	0.00	1.5
A_55_P2130178	NM_010233	Fn1	0.30	1.3	0.05	1.5	0.01	1.5
A_51_P451338	NM_010588	Jag2	0.20	1.3	0.04	1.5	0.01	1.5
A_55_P2027836	NM_020275	Tnfrsf10b	0.32	1.2	0.02	1.5	0.00	1.5
A_66_P100853	XM_978341	RP23-480B19.10	0.13	1.3	0.02	1.5	0.00	1.5
A_66_P115580	AK076360		0.15	1.3	0.02	1.5	0.01	1.5
A_51_P133612	NM_026014	Cdt1	0.01	1.4	0.02	1.5	0.00	1.5
A_55_P2011490	XM_001473460	Gm16510	0.02	1.4	0.01	1.5	0.00	1.5
A_55_P1980119	NM_024472	Gltpd1	0.05	1.4	0.01	1.5	0.00	1.5
A_55_P2160686	NM_009366	Tsc22d1	0.03	1.4	0.01	1.5	0.00	1.5
A_52_P151393	NM_198860	AI646023	0.01	1.4	0.01	1.5	0.00	1.5
A_55_P2136121	NM_145448	9030617O03Rik	0.00	1.4	0.01	1.5	0.00	1.5
A_51_P433026	NM_028922	Ppapdc2	0.11	1.3	0.01	1.5	0.00	1.5
A_55_P2023235	NM_007999	Fen1	0.13	1.2	0.00	1.5	0.00	1.5
A_51_P158210	NM_008564	Mcm2	0.00	1.4	0.00	1.5	0.00	1.5
A_55_P1953087	NM_008563	Mcm3	0.01	1.3	0.00	1.5	0.00	1.5
A_66_P125389	NM_001101475	F830016B08Rik	0.21	1.2	0.00	1.5	0.00	1.5
A_52_P335064	NM_181390	Mustn1	0.00	1.4	0.00	1.5	0.00	1.5
A_55_P2047621	NM_030021	D730039F16Rik	0.05	1.3	0.00	1.5	0.00	1.5
A_52_P529570	NM_198654	Nsl1	0.00	1.4	0.00	1.5	0.00	1.5
A_55_P2021285	NM_146067	Cpped1	0.01	1.3	0.00	1.5	0.00	1.5
A_30_P01023554			0.00	1.4	0.00	1.5	0.00	1.5
A_55_P1963483	NM_030559	Vps16	0.97	1.1	0.00	-3.6	0.42	1.5
A_55_P1963960	XM_918548	LOC641201	0.33	1.3	0.13	1.5	0.04	1.5
A_55_P1998943	NM_145211	Oas1a	0.32	1.3	0.32	1.4	0.04	1.5
A_55_P2048478	NM_172907	Olfml1	0.23	1.4	0.15	1.5	0.04	1.5
A_55_P2236291	NM_008903	Ppap2a	0.25	1.4	0.50	1.3	0.03	1.5
A_30_P01021453			0.84	1.1	0.27	1.4	0.03	1.5
A_66_P126640	NM_007592	Car8	0.43	1.2	0.12	1.4	0.03	1.5
A_55_P1958887	NM_001025384	DXBay18	0.33	1.3	0.26	1.4	0.03	1.5
A_55_P1998299	NM_001005608	Itgb4	0.79	1.1	0.56	1.2	0.03	1.5
A_51_P158678	NM_021427	Fam181b	0.59	1.2	0.23	1.4	0.03	1.5
A_51_P282760	NM_011066	Per2	0.25	1.3	0.10	1.5	0.03	1.5
A_51_P156438	NM_027460	Slc25a33	0.97	-1.0	0.51	1.2	0.03	1.5
A_55_P2088601	NM_010180	Fbln1	0.74	1.1	0.14	1.5	0.03	1.5
A_55_P1982818	NM_013614	Odc1	0.75	1.1	0.37	1.3	0.03	1.5
A_51_P491350	NM_009932	Col4a2	0.21	1.3	0.30	1.3	0.02	1.5
A_51_P493037	NM_018831	Dclre1a	0.53	1.2	0.79	1.1	0.02	1.5
A_51_P249118	NM_008830	Abcb4	0.27	1.3	0.39	1.3	0.02	1.5
A_30_P01018726			0.27	1.3	0.35	1.3	0.02	1.5
A_55_P2010312	NM_009243	Serpina1a	0.73	1.1	0.17	1.4	0.02	1.5
A_51_P195153	NM_013882	Gtse1	0.33	1.3	0.11	1.5	0.02	1.5
A_55_P2106039	NM_016693	Map3k6	0.29	1.3	0.60	1.2	0.02	1.5
A_51_P157042	NM_010217	Ctgf	0.19	1.4	0.16	1.5	0.02	1.5
A_55_P2026223	NM_011373	St6galnac4	0.28	1.3	0.26	1.3	0.02	1.5
A_51_P226269	NM_025427	1190002H23Rik	0.37	1.3	0.30	1.3	0.02	1.5

A_51_P514319	NM_172892	Slc13a4	0.07	1.4	0.25	1.3	0.01	1.5
A_51_P117226	AK046533	Zdhhc2	0.36	1.3	0.28	1.3	0.01	1.5
A_52_P645862	NM_177322	Agtr1a	0.13	1.4	0.35	1.3	0.01	1.5
A_51_P236267	NM_009183	St8sia4	0.83	1.1	0.38	1.3	0.01	1.5
A_55_P1971604	NM_001109758	Bcan	0.25	1.3	0.21	1.3	0.01	1.5
A_66_P118772	NM_001034863	Tmem136	0.51	1.2	0.31	1.3	0.01	1.5
A_55_P2130448	BY662175		0.94	1.0	0.37	1.3	0.01	1.5
A_55_P2093286	NM_007470	Apod	0.92	1.1	0.47	1.2	0.01	1.5
A_51_P272553	NM_011498	Bhlhe40	0.58	1.2	0.07	1.5	0.01	1.5
A_55_P2079009	NM_175316	Slco2b1	0.44	1.2	0.07	1.4	0.01	1.5
A_55_P2024555	NM_008903	Ppap2a	0.23	1.3	0.35	1.3	0.01	1.5
A_52_P325477	NM_053169	Trim16	0.08	1.4	0.21	1.3	0.01	1.5
A_55_P2010292	NM_009247	Serpina1e	0.84	1.1	0.31	1.3	0.01	1.5
A_55_P2083481	NM_001130412	Lpin1	0.34	1.2	0.18	1.3	0.01	1.5
A_52_P273821	NM_026179	Abhd5	0.60	1.1	0.20	1.3	0.01	1.5
A_52_P293120	NM_001081135	Prrg3	0.78	1.1	0.15	1.4	0.01	1.5
A_51_P256246	NM_025359	Tspan13	0.11	1.3	0.08	1.3	0.00	1.5
A_55_P1967539	NM_015755	Hunk	0.03	1.4	0.12	1.3	0.00	1.5
A_55_P2095360	NM_011576	Tfpi	0.07	1.3	0.07	1.3	0.00	1.5
A_55_P2037817	AK136939		0.16	1.3	0.02	1.4	0.00	1.5
A_55_P2129685	NM_001164053	Pkig	0.25	1.2	0.16	1.3	0.00	1.5
A_30_P01032951			0.00	1.4	0.02	1.3	0.00	1.5
A_66_P129188	NM_175662	Hist2h2ac	0.06	1.4	0.15	1.3	0.00	1.5
A_51_P179919	NM_172759	Ces5	0.19	1.2	0.07	1.3	0.00	1.5
A_65_P12104	NM_198967	Tmtc1	0.00	1.4	0.02	1.3	0.00	1.5
A_30_P01019396			0.08	1.2	0.02	1.3	0.00	1.5
A_52_P665675	NM_013454	Abca1	0.21	1.3	0.25	1.3	0.00	1.5
A_52_P130727	NM_177630	Ldoc1l	0.17	1.3	0.04	1.4	0.00	1.5
A_55_P2163928	NM_028375	Cxx1c	0.11	1.3	0.58	1.1	0.00	1.5
A_66_P112886	NM_011908	Ubl3	0.19	1.3	0.26	1.3	0.00	1.5
A_52_P380263	NM_013723	Podxl	0.10	1.4	0.39	1.3	0.00	1.5
A_55_P2084666	NM_175661	Hist1h2af	0.11	1.4	0.19	1.3	0.00	1.5
A_55_P2054628	NM_020252	Nrxn1	0.59	1.1	0.12	1.4	0.00	1.5
A_30_P01033082			0.09	1.3	0.05	1.4	0.00	1.5
A_30_P01025357			0.01	1.4	0.00	1.4	0.00	1.5
A_55_P2085295	NM_198411	Inf2	0.19	1.2	0.03	1.4	0.00	1.5
A_51_P163444	NM_025821	Carhsp1	0.00	1.4	0.00	1.4	0.00	1.5
A_30_P01025342			0.19	1.2	0.15	1.3	0.00	1.5
A_51_P432511	NM_145743	Lace1	0.05	1.3	0.05	1.3	0.00	1.5
A_51_P352968	NM_008538	Marcks	0.08	1.3	0.04	1.4	0.00	1.5
A_55_P2123502	NM_023844	Jam2	0.01	1.3	0.00	1.4	0.00	1.5
A_55_P1998797			0.65	1.1	0.26	1.3	0.00	1.5
A_55_P2048119	NM_146257	Slc29a4	0.70	1.1	0.25	1.2	0.00	1.5
A_51_P126626	NM_145459	Zfp503	0.02	1.3	0.01	1.3	0.00	1.5
A_51_P245368	NM_011075	Abcb1b	0.51	1.2	0.55	1.2	0.00	1.5
A_55_P1960496	NM_025800	Ppp1r2	0.20	1.3	0.09	1.4	0.00	1.5
A_51_P336599	NM_020574	Kcne3	0.29	1.2	0.07	1.4	0.00	1.5
A_30_P01029534			0.19	1.3	0.04	1.4	0.00	1.5
A_51_P265495	NM_010738	Ly6a	0.02	1.3	0.08	1.3	0.00	1.5

A_55_P1994042	NM_001139519	Zbp1	0.22	1.2	0.04	1.4	0.00	1.5
A_55_P2064328	NM_008481	Lama2	0.17	1.3	0.04	1.4	0.00	1.5
A_55_P2042016	XM_001479435	LOC100048058	0.45	1.2	0.42	1.2	0.00	1.5
A_55_P2158011	NM_026412	D2Ertd750e	0.08	1.3	0.15	1.3	0.00	1.5
A_55_P2083023	NM_133678	Sac3d1	0.01	1.4	0.04	1.4	0.00	1.5
A_51_P341918	NM_009366	Tsc22d1	0.12	1.3	0.05	1.4	0.00	1.5
A_52_P489778	NM_178688	Ablim1	0.02	1.4	0.01	1.4	0.00	1.5
A_55_P2115189	XM_001477969	Gm3796	0.09	1.3	0.21	1.3	0.00	1.5
A_30_P01027325			0.53	1.1	0.32	1.2	0.00	1.5
A_51_P248122	NM_133234	Bbc3	0.00	1.4	0.00	1.4	0.00	1.5
A_55_P2056496	NM_009387	Tk1	0.00	1.4	0.00	1.4	0.00	1.5
A_55_P1976744	XM_001478262	Gm4324	0.14	1.3	0.18	1.3	0.00	1.5
A_55_P1957245	XM_884146	Vmn2r-ps134	0.94	1.0	0.63	1.1	0.00	1.5
A_51_P374726	NM_008987	Ptx3	0.76	1.1	0.12	1.3	0.00	1.5
A_30_P01026572			0.01	1.4	0.01	1.4	0.00	1.5
A_52_P617327	NM_019466	Rcan1	0.13	1.3	0.05	1.4	0.00	1.5
A_55_P1975645	NM_198967	Tmtc1	0.00	1.3	0.01	1.4	0.00	1.5
A_55_P2183010	NM_001038887	P2rx7	0.01	1.3	0.00	1.4	0.00	1.5
A_55_P2096827	NM_181728	Art3	0.04	1.4	0.04	1.4	0.00	1.5
A_55_P2162136	NM_016957	Hmgn2	0.00	1.4	0.00	1.4	0.00	1.5
A_55_P2007273	NM_011132	Pole	0.06	1.4	0.02	1.4	0.00	1.5
A_51_P254425	NM_009644	Ahrr	0.01	1.3	0.02	1.4	0.00	1.5
A_52_P266132	NM_008013	Fgl2	0.15	1.3	0.10	1.4	0.00	1.5
A_52_P216672	NM_008940	Klk8	0.15	1.3	0.43	1.2	0.00	1.5
A_30_P01022991			0.02	1.3	0.00	1.4	0.00	1.5
A_66_P132295	NM_011576	Tfpi	0.22	1.3	0.42	1.2	0.00	1.5
A_51_P504354	NM_025980	Nrarp	0.05	1.3	0.01	1.4	0.00	1.5
A_55_P1964183	NM_001077694	Dysf	0.16	1.3	0.02	1.4	0.00	1.5
A_55_P2029558	NM_007420	Adrb2	0.10	1.3	0.07	1.4	0.00	1.5
A_55_P1981949	NM_175174	Klhl5	0.02	1.3	0.01	1.4	0.00	1.5
A_52_P108447	NM_031184	Glis2	0.06	1.3	0.45	1.2	0.00	1.5
A_30_P01018336			0.14	1.2	0.01	1.3	0.00	1.5
A_51_P268094	NM_009255	Serpine2	0.04	1.3	0.03	1.4	0.00	1.5
A_52_P217710	NM_008056	Fzd6	0.23	1.2	0.00	1.4	0.00	1.5
A_55_P2106150	NM_021790	Cenpk	0.14	1.2	0.04	1.3	0.00	1.5
A_55_P1967736	NM_027280	Nkd1	0.04	1.4	0.21	1.3	0.00	1.5
A_55_P2000833	NM_001013368	E2f8	0.01	1.4	0.04	1.4	0.00	1.5
A_55_P2142172	NM_001011761	Olfr1229	0.00	1.4	0.01	1.4	0.00	1.5
A_55_P1992421	NM_008253	Hmgb3	0.59	1.1	0.07	1.4	0.00	1.5
A_52_P393314	NM_011027	P2rx7	0.00	1.4	0.00	1.4	0.00	1.5
A_55_P2175451	XM_001474886	Gm9791	0.02	1.3	0.00	1.3	0.00	1.5
A_55_P2157033	NM_019517	Bace2	0.32	1.2	0.04	1.4	0.00	1.5
A_30_P01021819			0.42	1.2	0.01	1.4	0.00	1.5
A_55_P2119985	NM_130862	Baiap2	0.62	1.1	0.25	1.3	0.00	1.5
A_51_P224564	NM_176833	Ppm1f	0.13	1.3	0.11	1.3	0.00	1.5
A_55_P2023762	NM_007936	Epha4	0.08	1.3	0.05	1.3	0.00	1.5
A_30_P01018981			0.00	1.4	0.06	1.3	0.00	1.5
A_55_P2026214	XM_620527	Gm5899	0.01	1.4	0.01	1.4	0.00	1.5
A_52_P267824	NM_197999	2210023G05Rik	0.14	1.3	0.42	1.2	0.00	1.5

A_55_P2171086	XM_001479969	LOC100048394	0.53	1.2	0.17	1.4	0.00	1.5
A_52_P420466	NM_175660	Hist1h2ab	0.01	1.4	0.03	1.4	0.00	1.5
A_51_P449824	XM_001471750	Exoc3l2	0.41	1.2	0.12	1.3	0.00	1.5
A_55_P2085181	NM_028083	Chaf1b	0.25	1.2	0.11	1.3	0.00	1.5
A_55_P2112445	NM_011536	Tbx4	0.12	1.3	0.21	1.3	0.00	1.5
A_51_P282508	NM_007484	Rhoc	0.64	1.1	0.39	1.2	0.00	1.5
A_55_P1959869	NM_025800	Ppp1r2	0.73	1.1	0.09	1.3	0.00	1.5
A_52_P198435	NM_207246	Rasgrp3	0.06	1.4	0.17	1.3	0.00	1.5
A_55_P2244677	AK032738	6720422M22Rik	0.78	1.1	0.27	1.2	0.00	1.5
A_30_P01031572			0.05	1.4	0.09	1.4	0.00	1.5
A_55_P2099742	NM_011888	Ccl19	0.41	1.2	0.10	1.4	0.00	1.5
A_55_P1982817	XR_002167	Gm9115	0.82	1.1	0.26	1.3	0.00	1.5
A_30_P01025572			0.03	1.4	0.07	1.4	0.00	1.5
A_55_P2012734	NM_178699	B930041F14Rik	0.61	1.1	0.32	1.2	0.00	1.5
A_55_P1973560			0.03	1.4	0.02	1.4	0.00	1.5
A_55_P2054027	NM_019950	Chst5	0.12	1.3	0.06	1.3	0.00	1.5
A_55_P2080163	NM_025821	Carhsp1	0.01	1.3	0.01	1.3	0.00	1.5
A_51_P371091	NM_178593	Rcsd1	0.70	1.1	0.61	1.1	0.00	1.5
A_51_P363801	NM_023217	Pgpep1	0.00	1.4	0.01	1.4	0.00	1.5
A_30_P01030475			0.00	2.1	0.21	1.6	0.22	1.4
A_30_P01023291			0.04	1.8	0.25	1.5	0.26	1.4
A_51_P112355	NM_018738	Igtp	0.04	1.6	0.45	1.3	0.18	1.4
A_55_P2133195	NM_001033767	Gm4951	0.02	1.6	0.07	1.6	0.06	1.4
A_51_P393958	NM_025995	Fbxo5	0.00	1.6	0.01	1.5	0.00	1.4
A_52_P522372	NM_175503	Aard	0.00	1.6	0.14	1.4	0.02	1.4
A_52_P588881	NM_001033484	Iqgap3	0.04	1.5	0.13	1.4	0.04	1.4
A_55_P2192662	NM_001122899	Lepr	0.04	1.5	0.28	1.3	0.08	1.4
A_52_P601021	NM_026979	C1qtnf2	0.04	1.5	0.78	1.1	0.07	1.4
A_55_P2051334	NR_004446	Gm7035	0.03	1.5	0.27	1.3	0.05	1.4
A_51_P427516	NM_019576	Thsd1	0.02	1.5	0.15	1.4	0.03	1.4
A_55_P2070079	NM_010708	Lgals9	0.01	1.5	0.15	1.4	0.02	1.4
A_51_P264695	NM_016669	Crym	0.01	1.5	0.48	1.2	0.03	1.4
A_55_P2164469	NM_001163318	Gm11744	0.01	1.5	0.19	1.4	0.04	1.4
A_55_P2088401	NM_010399	H2-T9	0.01	1.5	0.12	1.3	0.01	1.4
A_30_P01025883			0.00	1.5	0.06	1.3	0.01	1.4
A_55_P2039324	NM_007634	Ccnf	0.00	1.5	0.00	1.6	0.00	1.4
A_55_P2021114	NM_010500	Ier5	0.00	1.5	0.00	1.5	0.00	1.4
A_55_P2294184	NM_031997	Tmem2	0.00	1.5	0.00	1.5	0.01	1.4
A_55_P2068663	NM_019641	Stmn1	0.00	1.5	0.08	1.3	0.00	1.4
A_55_P2187034			0.00	1.5	0.03	1.3	0.00	1.4
A_55_P1971599	NM_007529	Bcan	0.15	1.4	0.01	1.7	0.07	1.4
A_51_P361830	NM_144945	Lgi2	0.47	1.2	0.03	1.6	0.05	1.4
A_55_P1990066			0.04	1.4	0.02	1.6	0.03	1.4
A_55_P2111380	NM_008729	Ctnnd2	0.02	1.3	0.00	1.6	0.00	1.4
A_55_P2095508	XM_001478695	LOC100047693	0.14	1.3	0.05	1.5	0.04	1.4
A_51_P322972	NM_145419	Hkdc1	0.11	1.4	0.04	1.5	0.05	1.4
A_55_P2083919	NM_175549	Robo2	0.09	1.3	0.03	1.5	0.01	1.4
A_55_P2056729	NM_008342	Igfbp2	0.03	1.4	0.01	1.5	0.00	1.4
A_51_P477121	NM_021451	Pmaip1	0.00	1.4	0.00	1.5	0.00	1.4

A_51_P344566	NM_011121	Plk1	0.01	1.3	0.00	1.5	0.00	1.4
A_51_P258493	NM_011067	Per3	0.04	1.3	0.00	1.5	0.00	1.4
A_51_P366672	NM_153170	Slc36a2	0.00	1.4	0.00	1.5	0.00	1.4
A_51_P214985	NM_145492	Zfp521	0.01	1.3	0.00	1.5	0.00	1.4
A_30_P01033274			0.20	1.2	0.00	1.5	0.00	1.4
A_55_P2169659	AK083237		0.01	1.5	0.41	1.2	0.03	1.3
A_51_P346641	BC025879		0.22	1.2	0.00	1.7	0.08	1.3
A_55_P1992834	NM_007706	Socs2	0.25	1.2	0.01	1.5	0.04	1.3
A_55_P2055324	NM_016907	Spint1	0.30	1.2	0.01	1.5	0.03	1.3
A_52_P337259	NM_013905	Heyl	0.15	1.3	0.00	1.5	0.13	1.3
A_51_P184223	NM_053132	Pcdhb7	0.88	1.0	0.00	1.5	0.01	1.3
A_30_P01020841			0.03	1.8	0.38	1.4	0.55	1.2
A_55_P2027999	NM_001146100	Hk1	0.74	1.2	0.03	2.2	0.69	1.2
A_52_P425092			0.44	1.2	0.04	1.5	0.34	1.2
A_51_P207591	NM_013473	Anxa8	0.02	-1.5	0.86	-1.1	0.84	1.1
A_55_P2137828	NM_022420	Gprc5b	0.29	1.6	0.00	2.6	0.96	1.0
A_55_P2160416	NM_028765	Acoxl	0.47	-1.5	0.01	-2.9	0.96	-1.0
A_55_P2214124	BC019425		0.02	1.7	0.32	1.4	0.89	-1.1
A_55_P2180744	NM_153508	Clstn3	0.00	-1.5	0.10	-1.4	0.04	-1.3
A_55_P1957865	XM_001472293	Gm8090	0.00	-1.5	0.07	-1.4	0.07	-1.3
A_55_P2023537	XM_001476520	LOC100046290	0.00	-1.6	0.05	-1.5	0.09	-1.3
A_55_P2141479	NR_004414	Rnu2	0.01	-1.7	0.25	-1.4	0.19	-1.3
A_51_P110341	NM_170727	Scgb3a1	0.02	-2.7	0.37	-1.8	0.70	-1.3
A_66_P115531	NM_145151	Crebzf	0.98	-1.0	0.04	-1.5	0.04	-1.3
A_55_P1999992	NM_172693	Galnt12	0.05	-1.3	0.00	-1.5	0.01	-1.3
A_55_P2144597	NM_001123370	9030025P20Rik	0.24	-1.2	0.00	-1.6	0.02	-1.3
A_51_P501844	NM_175475	Cyp26b1	0.48	-1.6	0.04	-2.9	0.71	-1.3
A_51_P268069	NM_009189	Six1	0.04	-1.6	0.54	-1.2	0.08	-1.4
A_55_P2007088	NM_023270	Rnf128	0.04	-1.6	0.34	-1.3	0.13	-1.4
A_55_P2181191	NM_007569	Btg1	0.03	-1.7	0.18	-1.6	0.18	-1.4
A_30_P01018771			0.93	1.0	0.05	-1.5	0.01	-1.4
A_30_P01023251			0.99	-1.0	0.05	-1.5	0.03	-1.4
A_55_P2279807	AK160312	6720427I07Rik	0.92	1.1	0.05	-1.5	0.03	-1.4
A_30_P01022612			0.63	-1.1	0.04	-1.5	0.03	-1.4
A_55_P2426941	BC040234	Gprin3	0.21	-1.2	0.01	-1.5	0.01	-1.4
A_51_P517430	NM_007639	Cd1d1	0.01	-1.3	0.00	-1.5	0.00	-1.4
A_52_P708792	XM_489019	Gm5547	0.00	-1.4	0.00	-1.5	0.00	-1.4
A_55_P2156425	NM_026815	Upk1a	0.20	-1.3	0.02	-1.6	0.03	-1.4
A_51_P273609	NM_146125	Itpka	0.05	-1.5	0.05	-1.6	0.03	-1.5
A_51_P227445	AK008147		0.04	-1.5	0.36	-1.3	0.02	-1.5
A_30_P01024606			0.04	-1.5	0.06	-1.5	0.01	-1.5
A_55_P1966383	XM_001475163	Gm2936	0.02	-1.5	0.18	-1.3	0.00	-1.5
A_55_P2088720	XM_001477698	Gm3651	0.01	-1.5	0.46	-1.2	0.00	-1.5
A_55_P2122130	AK030294		0.01	-1.5	0.09	-1.4	0.00	-1.5
A_55_P2017362	NM_207203	BC068157	0.01	-1.5	0.08	-1.4	0.00	-1.5
A_66_P101360	NM_172916	Hydin	0.01	-1.5	0.05	-1.4	0.00	-1.5
A_55_P1966749	NM_028775	Cyp2s1	0.04	-1.6	0.19	-1.5	0.02	-1.5
A_66_P137660	NM_177377	Fam166b	0.01	-1.7	0.06	-1.6	0.01	-1.5
A_52_P281702	NM_010518	Igfbp5	0.00	-1.8	0.18	-1.3	0.00	-1.5

A_52_P514407	NM_013793	Klra15	0.16	-1.4	0.04	-1.5	0.01	-1.5
A_55_P1985623	NM_029600	Abcc3	0.16	-1.3	0.04	-1.5	0.00	-1.5
A_66_P139546	NM_008344	Igfbp6	0.13	-1.3	0.03	-1.5	0.00	-1.5
A_30_P01017637			0.17	-1.3	0.02	-1.5	0.00	-1.5
A_30_P01029746			0.87	-1.1	0.01	-1.5	0.00	-1.5
A_51_P139069	NM_172372	Wdr45	0.19	-1.2	0.00	-1.5	0.00	-1.5
A_55_P1992045	NM_054043	Msi2	0.00	-1.4	0.00	-1.5	0.00	-1.5
A_30_P01031307			0.44	-1.1	0.00	-1.5	0.00	-1.5
A_30_P01020135			0.93	-1.0	0.03	-1.6	0.01	-1.5
A_52_P650387	NM_001045530	Ccnjl	0.24	-1.3	0.02	-1.6	0.00	-1.5
A_55_P2293351	AK082896	C430010C01	0.36	-1.2	0.01	-1.6	0.00	-1.5
A_30_P01022310			0.11	-1.4	0.00	-1.6	0.00	-1.5
A_55_P2372228	DT902180	A430104N18Rik	0.80	-1.1	0.00	-1.7	0.00	-1.5
A_55_P2028365	NM_001170788	Lrrc36	0.34	-1.3	0.07	-1.5	0.04	-1.5
A_55_P2292046	AK084291	D230018H15Rik	0.95	1.0	0.52	-1.3	0.04	-1.5
A_55_P1965101	NM_001081643	Xlr3b	0.54	-1.2	0.46	-1.3	0.04	-1.5
A_55_P2190152	AK054449	4921509J17Rik	0.25	-1.4	0.43	-1.3	0.04	-1.5
A_55_P2032916	NM_139142	Slc6a20a	0.96	-1.0	0.65	-1.2	0.04	-1.5
A_55_P2017636	NM_011580	Thbs1	0.70	-1.2	0.91	-1.1	0.04	-1.5
A_52_P244193	NM_009846	Cd24a	0.19	-1.4	0.12	-1.5	0.04	-1.5
A_55_P2105808	NM_146186	Wdr62	0.91	-1.1	0.09	-1.5	0.04	-1.5
A_52_P222230	XM_001476722		0.89	-1.1	0.60	-1.2	0.03	-1.5
A_51_P150710	NM_152839	Igj	0.80	-1.1	0.85	-1.1	0.03	-1.5
A_55_P2005743	NM_026257	Ubxn11	0.06	-1.5	0.12	-1.5	0.03	-1.5
A_51_P419226	NM_025393	S100a14	0.23	-1.4	0.60	-1.2	0.03	-1.5
A_52_P461343	NM_023478	Upk3a	0.26	-1.4	0.82	-1.1	0.03	-1.5
A_55_P2089710	NM_007904	Ednrb	0.62	1.2	0.55	-1.2	0.03	-1.5
A_51_P509643	NM_001042451	Snca	0.97	1.0	0.99	1.0	0.03	-1.5
A_51_P131164	NM_027728	Enkur	0.10	-1.5	0.16	-1.5	0.03	-1.5
A_55_P2153783	NM_010231	Fmo1	0.64	1.2	0.23	-1.4	0.03	-1.5
A_55_P1965030	NM_001003915	Slc5a12	0.74	-1.1	0.18	-1.4	0.03	-1.5
A_55_P2078138	NM_172709	Otop1	0.77	-1.1	0.68	-1.2	0.02	-1.5
A_30_P01032357			0.77	-1.1	0.65	-1.2	0.02	-1.5
A_51_P196925	NM_009142	Cx3cl1	0.39	-1.2	0.24	-1.3	0.01	-1.5
A_55_P2009861	XM_001472138	Gm2015	0.02	-1.4	0.17	-1.3	0.01	-1.5
A_51_P116601	NM_172447	A330021E22Rik	0.11	-1.4	0.09	-1.4	0.01	-1.5
A_55_P2043337	NM_009937	Colq	0.93	-1.0	0.95	1.0	0.01	-1.5
A_51_P371174	NM_013863	Bag3	0.31	-1.3	0.23	-1.4	0.01	-1.5
A_55_P2022569	NM_008778	Pak3	0.13	-1.4	0.17	-1.4	0.01	-1.5
A_55_P1992617	NM_019985	Clec1b	0.67	1.1	0.97	-1.0	0.01	-1.5
A_55_P1980883	NM_001146351	Ephb6	0.53	-1.2	0.19	-1.4	0.01	-1.5
A_55_P2166985	NM_207677	Dedd2	0.33	-1.3	0.17	-1.4	0.01	-1.5
A_55_P1979674	NM_011772	Ikzf4	0.39	-1.2	0.42	-1.2	0.01	-1.5
A_55_P2076805	AK129022		0.24	-1.3	0.31	-1.3	0.01	-1.5
A_55_P2266977	NM_021414	Ahcyl2	0.45	-1.2	0.10	-1.3	0.01	-1.5
A_30_P01022284			0.93	-1.0	0.16	-1.3	0.00	-1.5
A_30_P01030436			0.14	-1.2	0.08	-1.3	0.00	-1.5
A_55_P2096422	NM_008381	Inhbb	0.05	-1.3	0.12	-1.3	0.00	-1.5
A_51_P111612	NM_001042592	Arrdc4	0.15	-1.3	0.05	-1.4	0.00	-1.5

A_66_P128079	AK166854	Krt8	0.26	-1.3	0.36	-1.2	0.00	-1.5
A_55_P2063465	NM_013691	Thbs3	0.38	-1.2	0.18	-1.3	0.00	-1.5
A_55_P2169839	AK154941		0.43	-1.2	0.05	-1.4	0.00	-1.5
A_30_P01031627			0.42	-1.2	0.16	-1.3	0.00	-1.5
A_55_P2408415	NR_001461	Kcnq1ot1	0.07	-1.4	0.26	-1.3	0.00	-1.5
A_52_P541270	NM_177687	Crebl2	0.07	-1.3	0.04	-1.3	0.00	-1.5
A_51_P446477	NM_171826	Cldnd1	0.11	-1.3	0.12	-1.4	0.00	-1.5
A_55_P2212027	NM_011682	Utrn	0.14	-1.3	0.26	-1.3	0.00	-1.5
A_66_P108019	NM_144835	Heatr1	0.15	-1.2	0.01	-1.4	0.00	-1.5
A_52_P554703	NM_183183	Gprin3	0.72	-1.1	0.22	-1.3	0.00	-1.5
A_55_P2159259	NM_013584	Lifr	0.24	-1.2	0.03	-1.4	0.00	-1.5
A_66_P128434	NM_001122733	Kit	0.99	1.0	0.65	-1.2	0.00	-1.5
A_55_P2136657	NM_013691	Thbs3	0.26	-1.2	0.21	-1.3	0.00	-1.5
A_55_P2413458	NM_023755	Tcfcp2l1	0.47	-1.2	0.60	-1.2	0.00	-1.5
A_55_P2286493	AK016664	4933405E24Rik	0.18	-1.3	0.20	-1.3	0.00	-1.5
A_66_P105801	NM_010513	Igf1r	0.08	-1.3	0.03	-1.3	0.00	-1.5
A_52_P274496	NM_183180	Tspan18	0.51	-1.1	0.03	-1.4	0.00	-1.5
A_55_P2017789	NM_001008700	Il4ra	0.93	-1.0	0.37	-1.2	0.00	-1.5
A_51_P475995	NM_178060	Thra	0.76	-1.1	0.28	-1.2	0.00	-1.5
A_51_P142923	NM_013490	Chka	0.30	-1.2	0.01	-1.3	0.00	-1.5
A_55_P2165869	NM_009883	Cebpb	0.01	-1.4	0.16	-1.3	0.00	-1.5
A_30_P01019232			0.85	-1.1	0.08	-1.4	0.00	-1.5
A_55_P2035315	NM_145839	Rasgef1b	0.71	-1.1	0.23	-1.3	0.00	-1.5
A_55_P2140212	XM_001474216	Gm2627	0.02	-1.5	0.30	-1.3	0.01	-1.6
A_30_P01032068			0.01	-1.5	0.00	-1.6	0.00	-1.6
A_55_P2029746	XM_001472371	1200016E24Rik	0.00	-1.6	0.32	-1.3	0.00	-1.6
A_55_P2363030	BC022771	Gm9853	0.02	-1.7	0.30	-1.4	0.01	-1.6
A_55_P2003824	NM_009714	Asgr1	0.00	-1.7	0.01	-1.7	0.03	-1.6
A_55_P2031167	NM_010107	Efna1	0.04	-1.4	0.01	-1.5	0.00	-1.6
A_55_P2069425	NM_133923	Ttll3	0.30	-1.2	0.01	-1.5	0.00	-1.6
A_30_P01025798			1.00	1.0	0.04	-1.6	0.00	-1.6
A_30_P01029720			0.98	-1.0	0.02	-1.6	0.01	-1.6
A_51_P234692	NR_003513	Neat1	0.91	-1.1	0.01	-1.6	0.00	-1.6
A_55_P2125613	NM_001101486	Fam71f2	0.15	-1.2	0.00	-1.6	0.00	-1.6
A_55_P2053258	NM_027354	Wdr51a	0.64	-1.2	0.27	-1.5	0.04	-1.6
A_51_P356055	NM_175012	Grp	0.83	-1.1	0.67	-1.2	0.04	-1.6
A_55_P2106911	NM_021279	Wnt1	0.15	-1.5	0.34	-1.4	0.03	-1.6
A_55_P2015832	NM_001005422	Gm1574	0.27	-1.4	0.55	-1.3	0.02	-1.6
A_55_P2068247	XM_001473755	Gm2488	0.24	-1.4	0.53	-1.3	0.02	-1.6
A_55_P2170349	NM_053152	Klra22	0.25	-1.4	0.15	-1.5	0.02	-1.6
A_55_P2021089	XM_001472541	Ighg	0.31	-1.3	0.27	-1.4	0.02	-1.6
A_51_P422369	NM_001013022	Odf3b	0.18	-1.4	0.15	-1.5	0.01	-1.6
A_66_P136389	NM_007560	Bmpr1b	0.09	-1.5	0.33	-1.4	0.01	-1.6
A_66_P108380	NM_172447	A330021E22Rik	0.24	-1.3	0.28	-1.4	0.01	-1.6
A_55_P2273929	AK076353	4732457N14	0.48	-1.2	0.13	-1.4	0.01	-1.6
A_55_P2035038	XM_001473590	Gm2437	0.27	-1.3	0.62	-1.2	0.01	-1.6
A_55_P2028734	NM_013794	Klra16	0.29	-1.3	0.08	-1.5	0.01	-1.6
A_55_P2004536	NM_010649	Klra4	0.19	-1.4	0.17	-1.4	0.00	-1.6
A_55_P2209053	AK013461	2900001G08Rik	0.17	-1.3	0.09	-1.4	0.00	-1.6

A_30_P01027510			0.59	-1.2	0.11	-1.4	0.00	-1.6
A_55_P2125557	NM_010574	Irx2	0.27	-1.2	0.07	-1.3	0.00	-1.6
A_55_P1977875	NM_176849	Arglu1	0.07	-1.4	0.32	-1.3	0.00	-1.6
A_55_P2169669	NM_001113530	Csf1	0.04	-1.3	0.08	-1.3	0.00	-1.6
A_55_P2017982	NM_001170669	Pde8b	0.60	-1.1	0.03	-1.4	0.00	-1.6
A_52_P799815	NM_001025606	Tmem171	0.07	-1.4	0.12	-1.4	0.00	-1.6
A_52_P312102	AK129018	Sema3g	0.22	-1.3	0.07	-1.4	0.00	-1.6
A_51_P279062	NM_027763	Treml1	0.61	1.1	0.39	-1.2	0.00	-1.6
A_55_P2175469	NM_001081345	Chd2	0.09	-1.4	0.15	-1.4	0.00	-1.6
A_55_P2020612	NM_025404	Arl4d	0.19	-1.3	0.15	-1.4	0.00	-1.6
A_51_P114616	NM_016767	Batf	0.44	-1.2	0.26	-1.3	0.00	-1.6
A_52_P233441	NM_008090	Gata2	0.21	-1.3	0.04	-1.4	0.00	-1.6
A_55_P2126557	XM_619973	Gm5858	0.32	-1.2	0.09	-1.4	0.00	-1.6
A_51_P227392	NM_133955	Rhou	0.02	-1.6	0.14	-1.5	0.00	-1.7
A_55_P2116650	NR_002860	A130040M12Rik	0.02	-1.6	0.60	-1.2	0.00	-1.7
A_51_P156955	NM_013459	Cfd	0.00	-4.8	0.58	-1.7	0.36	-1.7
A_30_P01021604			0.24	-1.2	0.01	-1.5	0.00	-1.7
A_55_P2168023	NM_001025379	Sema3g	0.62	-1.2	0.05	-1.6	0.00	-1.7
A_55_P2049771	XM_001480935	Gm4621	0.17	-1.4	0.03	-1.6	0.00	-1.7
A_30_P01029918			0.27	-1.3	0.03	-1.6	0.00	-1.7
A_30_P01032544			0.37	-1.3	0.02	-1.6	0.00	-1.7
A_55_P2347976	NR_029382	Mirhg1	0.47	-1.2	0.00	-1.6	0.00	-1.7
A_51_P283473	NM_026271	Fibin	0.70	-1.2	0.04	-1.7	0.00	-1.7
A_51_P125205	NM_007472	Aqp1	0.26	-1.3	0.00	-1.8	0.00	-1.7
A_30_P01021631			0.82	-1.1	0.00	-1.9	0.00	-1.7
A_55_P2370384	NR_028123	1600029I14Rik	0.60	-1.3	0.64	-1.3	0.04	-1.7
A_55_P2025655	CB248850		0.22	-1.5	0.11	-1.8	0.03	-1.7
A_55_P2002517	NM_001077361	Fhl1	1.00	-1.0	0.91	-1.1	0.03	-1.7
A_55_P1985950	NM_133213	Xpnpep2	0.88	1.1	0.12	-1.6	0.01	-1.7
A_51_P509679	XM_001474025		1.00	1.0	0.81	-1.1	0.01	-1.7
A_51_P343429	NM_026331	Slc25a37	0.69	-1.2	0.40	-1.3	0.01	-1.7
A_55_P2167803	NM_001014423	Abi3bp	0.50	-1.2	0.18	-1.4	0.00	-1.7
A_55_P2340593	AK007545	1810019D21Rik	0.50	-1.2	0.06	-1.5	0.00	-1.7
A_51_P172502	NM_001012477	Cxcl12	0.61	-1.2	0.10	-1.5	0.00	-1.7
A_51_P199352	XM_483917	2310015B20Rik	0.56	-1.2	0.21	-1.4	0.00	-1.7
A_51_P428372	NM_023785	Ppbp	0.41	1.2	0.47	-1.3	0.00	-1.7
A_66_P131433			0.19	-1.4	0.09	-1.5	0.00	-1.7
A_51_P305843	NM_025844	Chordc1	0.34	-1.3	0.12	-1.5	0.00	-1.7
A_55_P2035320	NM_017373	Nfil3	0.11	-1.4	0.09	-1.5	0.00	-1.7
A_55_P2403769	BB498095	AI481121	0.34	-1.2	0.00	-1.6	0.00	-1.8
A_52_P161495	NM_009744	Bcl6	0.12	-1.3	0.00	-1.6	0.00	-1.8
A_30_P01031764			0.20	-1.3	0.00	-1.6	0.00	-1.8
A_51_P439085	NM_023516	2310016C08Rik	0.26	-1.3	0.02	-1.7	0.00	-1.8
A_51_P155323	NM_010406	Hc	0.49	-1.2	0.01	-1.7	0.00	-1.8
A_55_P2058783	NM_023516	2310016C08Rik	0.27	-1.3	0.01	-1.7	0.00	-1.8
A_51_P247637	NM_080563	Rnf144a	0.45	-1.2	0.00	-1.8	0.00	-1.8
A_52_P33382	XM_356935	Gm5226	0.85	1.1	0.62	-1.3	0.03	-1.8
A_66_P138584	NM_029797	Mnd1	0.92	-1.1	0.73	-1.2	0.01	-1.8
A_51_P196972	NM_011403	Slc4a1	0.99	1.0	0.86	-1.1	0.01	-1.8

A_55_P2184023	XM_355197	Dnahc7a	0.09	-1.6	0.23	-1.5	0.00	-1.8
A_52_P577662	NM_007904	Ednrb	0.77	1.1	0.15	-1.6	0.00	-1.8
A_51_P265571	NM_009627	Adm	0.44	-1.3	0.07	-1.6	0.00	-1.8
A_51_P447976	NM_001142952	Fam46c	0.28	-1.3	0.14	-1.5	0.00	-1.8
A_51_P431329	NM_007606	Car3	0.00	-2.7	0.66	-1.4	0.07	-1.9
A_51_P279693	NM_009992	Cyp1a1	0.00	-4.3	0.00	-3.7	0.02	-1.9
A_55_P1963533	NM_001003672	Pcdhac2	0.64	-1.1	0.03	-1.5	0.00	-1.9
A_55_P2056344	NM_001142539	Gm9992	0.89	-1.1	0.10	-1.9	0.03	-1.9
A_55_P2131766	NM_023665	D4Wsu53e	0.47	-1.4	0.13	-1.7	0.01	-1.9
A_55_P2087985			0.55	-1.2	0.12	-1.6	0.00	-1.9
A_55_P2087984	NM_001164671	Dnaja1	0.72	-1.2	0.25	-1.5	0.00	-1.9
A_55_P2050226	AY072938	Ccrl1	0.79	-1.1	0.08	-1.4	0.00	-1.9
A_51_P505617	NM_008365	Il18r1	0.49	-1.2	0.15	-1.4	0.00	-1.9
A_52_P229052	NM_019790	Tmeff2	0.74	-1.2	0.23	-1.5	0.00	-1.9
A_55_P2038358	NM_012006	Acot1	0.14	-1.4	0.15	-1.5	0.00	-1.9
A_52_P340073	NM_010111	Efnb2	0.08	-1.4	0.00	-1.6	0.00	-2.0
A_55_P1990032	NM_009141	Cxcl5	0.71	-1.3	0.18	-1.9	0.03	-2.0
A_55_P2301312	NM_146254	Wdr78	0.10	-1.8	0.36	-1.6	0.02	-2.0
A_55_P2135967	XM_979793	Gm7816	0.66	-1.2	0.14	-1.6	0.00	-2.0
A_55_P1993404			0.65	-1.2	0.13	-1.6	0.00	-2.0
A_51_P179258	NM_001161665	Kif26b	0.22	-1.4	0.02	-1.8	0.00	-2.1
A_51_P153486	NM_018808	Dnajb1	0.20	-1.4	0.01	-1.9	0.00	-2.1
A_55_P2176963	NM_013559	Hsph1	0.69	-1.3	0.60	-1.4	0.02	-2.1
A_55_P2003513	NM_013559	Hsph1	0.58	-1.4	0.61	-1.4	0.00	-2.2
A_55_P2097518	NM_001080943	Zdhhc22	0.70	-1.2	0.06	-2.0	0.00	-2.5
A_51_P331328	NM_026730	Gpihbp1	0.32	-1.3	0.00	-1.8	0.00	-2.6
A_55_P2408588	NM_007489	Arntl	0.11	-1.6	0.00	-2.5	0.00	-2.8
A_52_P257625	NM_023612	Esm1	0.00	-1.7	0.01	-1.9	0.00	-3.0
A_55_P2000973	NM_181529	Syt15	0.32	-1.3	0.00	-2.0	0.00	-3.1
A_52_P381484	NM_133903	Spon2	0.08	-1.7	0.01	-2.0	0.00	-3.3
A_55_P2068459	NM_010479	Hspa1a	0.88	-1.1	0.15	-1.9	0.00	-3.3

3.3.7 IP Lung. Significant probe list. List of all significantly differentially expressed probes in at least 1 treatment group (FDR P ≤ 0.05, fold change ± 1.5) in response to sub-chronic oral exposure to 12.5, 25, and 50 mg/kg-bw/day indeno(123,cd)pyrene in the lung. The list is sorted from highest to lowest fold change in the 50 mg/kg-bw/day treatment group.

			**12.5 mg/kg-bw/day**		**25 mg/kg-bw/day**		**50 mg/kg-bw/day**	
Agilent Probe	Accession Number	Gene Symbol	*FDR P value*	*Fold change*	*FDR P value*	*Fold change*	*FDR P value*	*Fold change*
A_51_P426353	NM_009463	Ucp1	0.00	-7.6	0.00	-7.1	0.00	-7.7
A_51_P336833	NM_024406	Fabp4	0.00	-4.4	0.00	-5.8	0.00	-4.8
A_51_P199168	NM_007702	Cidea	0.00	-2.6	0.00	-2.7	0.00	-3.0
A_66_P121110	NM_011044	Pck1	0.00	-2.6	0.00	-3.2	0.00	-3.0
A_55_P2018666	NM_009381	Thrsp	0.02	-2.5	0.00	-4.6	0.00	-3.8
A_52_P423814	NM_007751	Cox8b	0.04	-2.3	0.01	-2.5	0.00	-2.7
A_30_P01033043			0.00	-2.2	0.02	-1.7	0.00	-1.9
A_51_P244497	NM_175640	Plin1	0.04	-2.2	0.00	-2.9	0.00	-2.7

A_52_P592909	NM_026384	Dgat2	0.00	-2.1	0.00	-2.1	0.00	-2.2
A_55_P2056344	NM_001142539	Gm9992	0.00	-2.1	0.00	-1.9	0.00	-2.5
A_55_P2068459	NM_010479	Hspa1a	0.02	-2.1	0.00	-3.5	0.00	-4.6
A_30_P01018650			0.00	-2.0	0.04	-1.6	0.01	-1.6
A_51_P350453	NM_013743	Pdk4	0.00	-2.0	0.01	-1.9	0.00	-2.4
A_55_P2107785	XM_001475876	Gm13105	0.05	-2.0	0.00	-3.1	0.00	-2.4
A_30_P01019643			0.00	-1.9	0.01	-1.6	0.00	-1.7
A_55_P2403769	BB498095	AI481121	0.00	-1.8	0.01	-1.6	0.00	-2.2
A_55_P2124461	XM_001472850	Gm2251	0.02	-1.8	0.00	-2.5	0.00	-2.1
A_51_P346964	NM_029844	Mrap	0.04	-1.8	0.00	-1.9	0.00	-1.8
A_30_P01029645			0.00	-1.7	0.05	-1.4	0.02	-1.5
A_55_P1958840	XM_001474656	Gm5811	0.00	-1.7	0.01	-1.8	0.00	-1.7
A_55_P1965030	NM_001003915	Slc5a12	0.00	-1.7	0.01	-1.6	0.00	-2.0
A_55_P2140212	XM_001474216	Gm2627	0.02	-1.7	0.00	-2.1	0.01	-1.7
A_30_P01026062			0.00	-1.6	0.05	-1.3	0.01	-1.4
A_51_P283473	NM_026271	Fibin	0.00	-1.6	0.10	-1.4	0.00	-1.9
A_30_P01019556			0.00	-1.6	0.04	-1.4	0.01	-1.5
A_52_P670812	NR_028575	Snord123	0.04	-1.6	0.19	-1.3	0.06	-1.4
A_51_P463440	NM_130450	Elovl6	0.04	-1.6	0.00	-1.8	0.00	-1.6
A_30_P01023130			0.04	-1.6	0.04	-1.4	0.06	-1.4
A_55_P2105808	NM_146186	Wdr62	0.00	-1.5	0.00	-1.5	0.00	-1.6
A_30_P01029468			0.00	-1.5	0.09	-1.3	0.01	-1.4
A_55_P2178137	NM_011889	41155.0	0.00	-1.5	0.00	-1.6	0.00	-1.9
A_55_P2020612	NM_025404	Arl4d	0.00	-1.5	0.00	-1.5	0.00	-1.8
A_55_P2027812	NM_001166369	2900062L11Rik	0.02	-1.5	0.05	-1.4	0.00	-1.6
A_30_P01017626			0.00	1.5	0.00	1.6	0.00	1.5
A_51_P185660	NM_011338	Ccl9	0.02	1.5	0.71	1.1	0.02	1.4
A_66_P115580	AK076360		0.00	1.7	0.00	1.8	0.00	2.5
A_51_P447189	AK041326	1700030C10Rik	0.04	1.8	0.13	1.5	0.02	1.6
A_55_P1966774	XM_893705	Gm6930	0.02	2.5	0.92	-1.1	0.02	2.3
A_51_P326191	NM_009251	Serpina3g	0.00	2.7	0.53	1.3	0.00	2.9
A_55_P2060922	NM_153131	Unc5a	0.00	3.1	0.06	2.1	0.00	3.3
A_55_P2142226	NM_001034870	Serpina3h	0.00	5.0	0.78	-1.3	0.00	5.0
A_51_P156955	NM_013459	Cfd	0.71	-1.6	0.00	-6.4	0.05	-2.8
A_52_P413395	NM_025540	Sln	0.20	-3.7	0.00	-6.3	0.03	-3.8
A_51_P431329	NM_007606	Car3	0.22	-2.4	0.00	-5.9	0.00	-3.7
A_55_P2173952	NM_001164171	Myh6	0.31	-3.2	0.01	-5.5	0.13	-2.9
A_51_P193185	NM_013593	Mb	0.42	-2.2	0.01	-3.9	0.05	-2.8
A_55_P2107045	NM_010858	Myl4	0.28	-2.9	0.02	-3.8	0.06	-2.9
A_51_P202331	NM_011126	Plunc	0.51	-2.2	0.04	-3.7	0.00	-5.2
A_65_P10195	NM_022879	Myl7	0.28	-2.7	0.02	-3.7	0.06	-2.8
A_51_P458451	NM_009605	Adipoq	0.26	-2.2	0.00	-3.3	0.02	-2.6
A_51_P416858	NM_021285	Myl1	0.11	-2.9	0.02	-3.1	0.09	-2.2
A_55_P1984806	NM_022984	Retn	0.49	-1.7	0.00	-3.1	0.03	-2.2
A_51_P165293	NM_198415	Ckmt2	0.22	-2.5	0.02	-3.0	0.25	-1.8
A_55_P1953143	NM_144783	Wt1	0.31	-1.6	0.00	-3.0	0.01	-2.2
A_51_P133684	NM_013808	Csrp3	0.32	-2.3	0.04	-2.9	0.10	-2.3
A_51_P386983	NM_009406	Tnni3	0.32	-2.2	0.03	-2.9	0.14	-2.1
A_55_P2071952	NM_178909	Wdr92	0.06	-1.9	0.00	-2.9	0.00	-2.4

A_30_P01023737			0.18	-1.8	0.00	-2.9	0.00	-2.4
A_51_P338262	NM_011619	Tnnt2	0.28	-2.3	0.04	-2.8	0.07	-2.4
A_55_P2428514	AK131869	Retn	0.50	-1.6	0.00	-2.7	0.02	-2.2
A_55_P1994229	NM_010473	Hrc	0.43	-1.9	0.03	-2.6	0.10	-2.0
A_51_P264495	NM_018870	Pgam2	0.13	-2.4	0.02	-2.5	0.14	-1.8
A_52_P58145	NM_009022	Aldh1a2	0.17	-1.7	0.00	-2.5	0.00	-2.3
A_55_P2042500	NM_145635	BC054059	0.06	-2.0	0.00	-2.4	0.00	-2.2
A_52_P460836	NM_177303	Lrrn4	0.20	-1.7	0.00	-2.4	0.03	-1.7
A_55_P1990032	NM_009141	Cxcl5	0.89	1.2	0.00	-2.4	0.02	-2.1
A_51_P146560	NM_018857	Msln	0.61	-1.4	0.00	-2.3	0.03	-1.8
A_55_P1972034	XM_001476091	Muc16	0.54	-1.5	0.00	-2.3	0.00	-2.2
A_51_P175424	NM_011797	Car14	0.32	-1.4	0.00	-2.2	0.00	-2.7
A_55_P2176963	NM_013559	Hsph1	0.50	-1.4	0.00	-2.2	0.00	-2.7
A_55_P2088720	XM_001477698	Gm3651	0.06	-1.6	0.00	-2.2	0.00	-1.7
A_55_P2003513	NM_013559	Hsph1	0.54	-1.4	0.00	-2.2	0.00	-2.6
A_51_P271984	NM_144936	Tmem45b	0.11	-1.8	0.00	-2.2	0.00	-2.4
A_51_P148612	NM_009944	Cox7a1	0.17	-1.9	0.02	-2.1	0.01	-2.2
A_55_P2159485	XM_001476778	Gm3459	0.75	-1.3	0.01	-2.1	0.21	-1.5
A_55_P2011341	XM_001474162	LOC100045268	0.21	-1.6	0.01	-2.1	0.01	-1.9
A_55_P2011862	NM_008653	Mybpc3	0.35	-1.7	0.02	-2.0	0.02	-2.0
A_51_P255699	NM_010809	Mmp3	0.32	-1.5	0.00	-2.0	0.05	-1.6
A_55_P2087984	NM_001164671	Dnaja1	0.34	-1.4	0.00	-2.0	0.00	-2.3
A_55_P2036240	XM_001478041	Gm3804	0.36	-1.5	0.00	-2.0	0.01	-1.7
A_55_P2090505	XM_001480011	Gm4382	0.20	-1.5	0.00	-2.0	0.00	-1.8
A_51_P495780	NM_020568	Plin4	0.44	-1.5	0.04	-1.9	0.06	-1.7
A_51_P217498	NM_009204	Slc2a4	0.26	-1.7	0.03	-1.9	0.08	-1.7
A_51_P497985	NM_013484	C2	0.74	-1.2	0.02	-1.9	0.08	-1.5
A_55_P2094060	NM_010370	Gzma	0.29	-1.6	0.01	-1.9	0.02	-1.7
A_55_P2169586	NM_026439	Ccdc80	0.35	-1.4	0.01	-1.9	0.04	-1.5
A_51_P492456	NM_008215	Has1	0.35	-1.4	0.00	-1.9	0.01	-1.8
A_52_P281702	NM_010518	Igfbp5	0.32	-1.4	0.00	-1.9	0.01	-1.6
A_55_P1978866	XM_001472829	LOC100039402	0.07	-1.6	0.00	-1.9	0.02	-1.6
A_55_P2135967	XM_979793	Gm7816	0.35	-1.4	0.00	-1.9	0.00	-2.3
A_55_P2048767	NM_146015	Efemp1	0.82	-1.2	0.00	-1.9	0.04	-1.6
A_52_P114889	NM_175309	Upk3b	0.56	-1.3	0.00	-1.9	0.02	-1.7
A_55_P1954092	XM_001473985	Gm2563	0.07	-1.6	0.00	-1.9	0.02	-1.5
A_55_P2029746	XM_001472371	1200016E24Rik	0.15	-1.6	0.00	-1.9	0.05	-1.5
A_55_P2097518	NM_001080943	Zdhhc22	0.14	-1.7	0.02	-1.8	0.00	-2.6
A_55_P1957213	XM_001479389	Gm4148	0.26	-1.5	0.01	-1.8	0.06	-1.5
A_55_P1962918	NM_001033450	Mnda	0.17	-1.4	0.00	-1.8	0.01	-1.5
A_55_P2035320	NM_017373	Nfil3	0.26	-1.3	0.00	-1.8	0.00	-1.9
A_55_P1953377			0.73	-1.2	0.00	-1.8	0.00	-1.8
A_55_P2007496	XM_001472254	Gm2045	0.86	-1.1	0.02	-1.7	0.22	-1.4
A_51_P101985	NM_001166662	Ccdc85a	0.50	-1.4	0.02	-1.7	0.00	-2.0
A_55_P1964348	NM_023608	Gdpd2	1.00	-1.0	0.01	-1.7	0.06	-1.5
A_55_P1954724	NR_002860	A130040M12Rik	0.29	-1.4	0.01	-1.7	0.12	-1.4
A_55_P1974984	XM_001474652	LOC100045638	0.23	-1.5	0.01	-1.7	0.06	-1.5
A_52_P482897	NM_009704	Areg	0.41	-1.4	0.01	-1.7	0.05	-1.5
A_55_P1960148	XM_001472091		0.39	-1.4	0.01	-1.7	0.00	-2.2

A_55_P1960936	XM_001473982	Gm2562	0.30	-1.4	0.01	-1.7	0.14	-1.4
A_52_P676819	NM_007562	Bnc1	0.27	-1.4	0.00	-1.7	0.02	-1.5
A_55_P2173837	NM_001164734	Mpp6	0.32	-1.3	0.00	-1.7	0.01	-1.4
A_52_P16419	NM_010271	Gpd1	0.11	-1.5	0.00	-1.7	0.00	-1.6
A_55_P1973347	NR_002860	A130040M12Rik	0.13	-1.5	0.00	-1.7	0.00	-1.6
A_51_P269203	NM_022017	Trpv4	0.99	-1.0	0.05	-1.6	0.04	-1.6
A_51_P464703	NM_021443	Ccl8	0.57	-1.3	0.05	-1.6	0.03	-1.6
A_51_P407984	NM_030022	Grifin	0.62	1.3	0.04	-1.6	0.84	-1.1
A_55_P2087985			0.77	-1.2	0.04	-1.6	0.00	-1.7
A_55_P1993404			0.62	-1.3	0.04	-1.6	0.00	-1.9
A_55_P2016105	NM_133753	Errfi1	0.09	-1.5	0.02	-1.6	0.00	-1.7
A_55_P2028734	NM_013794	Klra16	0.19	-1.5	0.02	-1.6	0.00	-1.7
A_55_P2057946	NM_010480	Hsp90aa1	0.73	-1.2	0.02	-1.6	0.01	-1.6
A_51_P509679	XM_001474025		0.41	-1.4	0.01	-1.6	0.00	-1.9
A_51_P305843	NM_025844	Chordc1	0.35	-1.3	0.01	-1.6	0.00	-1.8
A_55_P2170349	NM_053152	Klra22	0.26	-1.4	0.00	-1.6	0.01	-1.6
A_55_P2105843	XM_001472740	Gm2371	0.20	-1.5	0.00	-1.6	0.04	-1.5
A_30_P01025818			0.41	-1.3	0.00	-1.6	0.02	-1.4
A_55_P2034655	AK007918		0.05	-1.4	0.00	-1.6	0.01	-1.4
A_52_P514407	NM_013793	Klra15	0.08	-1.4	0.00	-1.6	0.01	-1.5
A_55_P2182716	NM_145635	BC054059	0.26	-1.3	0.00	-1.6	0.01	-1.5
A_52_P250555	NM_019682	Dynll1	0.63	-1.2	0.00	-1.6	0.00	-1.6
A_51_P311038	NM_001024139	Adamts15	0.00	-1.3	0.00	-1.6	0.00	-1.2
A_51_P150710	NM_152839	Igj	0.29	-1.3	0.00	-1.6	0.00	-1.5
A_51_P173678	NM_029415	Slc10a6	0.47	-1.3	0.05	-1.5	0.00	-2.1
A_55_P2009861	XM_001472138	Gm2015	0.35	-1.4	0.04	-1.5	0.14	-1.3
A_52_P537545	NM_021491	Smpd3	0.51	-1.3	0.03	-1.5	0.10	-1.4
A_55_P2165790	NM_178706	Siglech	0.51	-1.3	0.03	-1.5	0.00	-1.7
A_30_P01029006			0.63	-1.2	0.03	-1.5	0.18	-1.3
A_55_P2025820	XM_001480287		0.33	-1.3	0.02	-1.5	0.03	-1.4
A_30_P01020751			0.25	-1.4	0.02	-1.5	0.10	-1.3
A_55_P2214124	BC019425		0.33	-1.3	0.02	-1.5	0.00	-1.6
A_66_P105175	NM_009738	Bche	0.08	-1.4	0.02	-1.5	0.34	-1.2
A_52_P573336	NM_172205	Sbsn	0.52	-1.2	0.02	-1.5	0.11	-1.3
A_55_P2057528	NM_025404	Arl4d	0.13	-1.4	0.01	-1.5	0.00	-1.6
A_55_P2154252	NM_013529	Gfpt2	0.47	-1.3	0.01	-1.5	0.07	-1.4
A_51_P142744	NM_172294	Sulf1	0.32	-1.3	0.01	-1.5	0.00	-1.5
A_55_P2058157	NM_134034	Smek2	0.73	-1.2	0.01	-1.5	0.02	-1.4
A_55_P2044282	NM_001164734	Mpp6	0.61	-1.2	0.01	-1.5	0.18	-1.3
A_55_P2185890	XM_001479650	LOC100048018	0.16	-1.3	0.01	-1.5	0.00	-1.6
A_55_P2048378	NM_001008427	Gm5595	0.06	-1.5	0.01	-1.5	0.03	-1.4
A_55_P2139430	XM_001479756	LOC100048207	0.88	-1.1	0.01	-1.5	0.77	-1.1
A_51_P487175	NM_212441	Acsm3	0.43	-1.3	0.01	-1.5	0.01	-1.5
A_55_P1955587	NM_010477	Hspd1	0.60	-1.2	0.01	-1.5	0.00	-1.7
A_51_P386880	NM_016737	Stip1	0.70	-1.2	0.01	-1.5	0.00	-1.6
A_55_P2004536	NM_010649	Klra4	0.50	-1.3	0.01	-1.5	0.03	-1.4
A_51_P514405	NM_019741	Slc2a5	0.00	-1.4	0.01	-1.5	0.01	-1.4
A_66_P103027	NM_172732	Clec9a	0.00	-1.4	0.00	-1.5	0.00	-1.4
A_66_P103271	NM_001164598	Irf2bp2	0.39	-1.2	0.00	-1.5	0.00	-1.5

A_30_P01032068			0.38	-1.3	0.00	-1.5	0.02	-1.4
A_55_P1983418	NM_007446	Amy1	0.06	-1.5	0.00	-1.5	0.02	-1.4
A_55_P2279140	AK089751	F830014O18Rik	0.51	-1.2	0.00	-1.5	0.01	-1.4
A_52_P624434	NM_175526	Clec1a	0.90	1.1	0.04	1.5	0.22	1.3
A_55_P1955015	NM_178440	Myo1g	0.28	1.4	0.04	1.5	0.04	1.4
A_55_P2032232	NM_011268	Rgs9	0.85	1.1	0.03	1.5	0.01	1.5
A_51_P157042	NM_010217	Ctgf	0.91	1.1	0.03	1.5	0.08	1.3
A_51_P408649	NM_145463	Shisa2	0.82	1.1	0.02	1.5	0.14	1.3
A_51_P181286	NM_001033122	Cd69	0.54	1.2	0.02	1.5	0.00	1.6
A_52_P216613	NM_182806	Gpr18	0.16	1.4	0.01	1.5	0.11	1.3
A_30_P01025417			0.45	1.2	0.00	1.5	0.00	1.5
A_51_P307168	NM_026993	Ddah1	0.92	1.0	0.00	1.5	0.00	1.7
A_55_P1986282	NM_001111099	Cdkn1a	0.57	1.2	0.00	1.5	0.00	1.8
A_51_P134812	NM_026929	Chac1	0.63	1.1	0.00	1.5	0.01	1.3
A_55_P2137406	NM_007527	Bax	0.00	1.3	0.00	1.5	0.00	1.7
A_30_P01027827			0.53	1.2	0.00	1.5	0.00	1.6
A_55_P2027022	NM_007548	Prdm1	0.69	1.1	0.00	1.5	0.01	1.4
A_51_P202623	NM_028832	Mterfd3	0.07	1.3	0.00	1.5	0.00	1.5
A_55_P2026295	NM_153399	Syne1	0.88	1.1	0.00	1.5	0.11	1.2
A_52_P263658	NM_008236	Hes2	0.70	1.1	0.00	1.5	0.04	1.3
A_51_P194498	NM_023395	Wfdc1	0.15	1.4	0.00	1.5	0.01	1.5
A_55_P1969032	NM_011268	Rgs9	0.79	1.1	0.00	1.5	0.02	1.4
A_51_P290576	NM_152804	Plk2	0.06	1.3	0.00	1.5	0.00	1.6
A_55_P2005213	NM_145603	Ces2	0.28	1.2	0.00	1.5	0.00	1.5
A_52_P73620	NM_198028	Serpinb10	0.71	1.2	0.03	1.6	0.01	1.6
A_51_P449325	NM_008206	H2-Oa	0.16	1.5	0.01	1.6	0.00	1.8
A_66_P122158	NR_003518	Pisd-ps3	0.16	1.3	0.00	1.6	0.00	1.6
A_55_P1960735	NM_011819	Gdf15	0.42	1.2	0.00	1.6	0.00	1.8
A_30_P01023499			0.42	1.2	0.00	1.6	0.00	1.5
A_51_P110471	NM_026993	Ddah1	0.57	1.2	0.00	1.6	0.00	1.6
A_51_P247184	NM_008728	Npr3	0.28	1.6	0.05	1.7	0.03	1.7
A_30_P01032234			0.68	1.3	0.02	1.7	0.01	1.7
A_52_P70796	NM_007551	Cxcr5	0.42	1.4	0.02	1.7	0.01	1.8
A_55_P2117345	NM_013517	Fcer2a	0.41	1.4	0.02	1.7	0.00	1.9
A_52_P390944	NM_016803	Chst3	0.52	1.2	0.00	1.7	0.00	1.6
A_55_P1959500	NM_172759	Ces5	0.81	1.1	0.00	1.7	0.00	1.8
A_52_P627269	NM_198171	BC015286	0.14	1.3	0.00	1.7	0.00	1.6
A_66_P135391	NM_008342	Igfbp2	0.57	1.3	0.01	1.8	0.05	1.5
A_51_P444137	NM_001143765	Syce1	0.38	1.5	0.01	1.8	0.30	1.3
A_51_P154485	NM_145141	Fcrla	0.51	1.4	0.01	1.8	0.00	1.9
A_52_P257625	NM_023612	Esm1	0.47	1.2	0.00	1.8	0.00	1.5
A_52_P303891	NM_011584	Nr1d2	0.47	1.4	0.01	1.9	0.00	2.1
A_55_P2056729	NM_008342	Igfbp2	0.52	1.3	0.00	1.9	0.02	1.6
A_55_P2019577	NR_027818	1500011B03Rik	0.92	1.1	0.00	2.0	0.01	1.7
A_66_P118600	NM_008480	Lama1	0.14	1.9	0.01	2.2	0.00	3.4
A_51_P254425	NM_009644	Ahrr	0.14	1.7	0.00	2.5	0.00	5.3
A_51_P223776	NM_145434	Nr1d1	0.37	1.6	0.00	2.6	0.00	2.7
A_51_P255456	NM_009994	Cyp1b1	0.15	2.1	0.00	2.8	0.00	12.5
A_55_P2032081	NM_016974	Dbp	0.61	1.6	0.00	3.5	0.00	4.5

A_55_P2032079	NM_016974	Dbp	0.45	1.7	0.00	3.8	0.00	5.2
A_51_P279693	NM_009992	Cyp1a1	0.91	1.2	0.00	5.5	0.00	17.8
A_55_P1995698	NM_145585	Thumpd1	0.87	1.3	0.00	6.2	0.00	4.3
A_55_P2165414	NM_009243	Serpina1a	0.10	2.3	0.99	-1.0	0.00	2.6
A_55_P2148534	BC096461	Nr1d2	0.56	1.4	0.06	1.8	0.00	2.4
A_55_P2104975	NM_001168294	Serpina3f	0.50	1.5	0.12	1.7	0.00	2.2
A_55_P1965154	NM_025565	Spc25	0.95	1.0	0.42	1.2	0.00	2.2
A_55_P2010301	NM_009245	Serpina1c	0.27	1.8	0.98	-1.0	0.02	2.1
A_55_P2205650	AK162965	2610507I01Rik	0.86	1.2	0.24	1.7	0.04	2.0
A_55_P2046709			0.34	1.7	0.90	-1.1	0.02	2.0
A_55_P2113857	NM_009247	Serpina1e	0.30	1.7	0.86	-1.1	0.02	2.0
A_52_P483336	NM_007641	Ms4a1	0.41	1.6	0.08	1.8	0.01	2.0
A_51_P455897	NM_144526	Fam64a	0.76	1.1	0.31	1.2	0.00	2.0
A_55_P1983773	NM_001012273	Birc5	0.96	1.0	0.41	1.2	0.00	2.0
A_51_P481920	NM_009828	Ccna2	0.95	1.0	0.37	1.2	0.00	2.0
A_51_P231320	NM_008611	Mmp8	0.34	1.3	0.04	1.4	0.00	2.0
A_55_P2082929	NM_010389	H2-Ob	0.54	1.4	0.08	1.6	0.00	2.0
A_51_P483576	AF138742		0.65	1.4	0.16	1.6	0.03	1.9
A_51_P342652	NM_008339	Cd79b	0.49	1.5	0.10	1.7	0.02	1.9
A_55_P2145224	AF290571		0.60	1.4	0.16	1.6	0.01	1.9
A_55_P2057283	AY170502		0.61	1.4	0.19	1.6	0.01	1.9
A_55_P2089233	NM_011136	Pou2af1	0.38	1.5	0.10	1.6	0.00	1.9
A_51_P253803	NM_001081117	Mki67	0.98	-1.0	0.73	1.1	0.00	1.9
A_55_P1988202	NM_001045481	Ifi203	0.53	1.3	0.06	1.4	0.00	1.9
A_55_P2010298	NM_009246	Serpina1d	0.40	1.5	0.93	-1.1	0.04	1.8
A_51_P485421	AK088666		0.35	1.5	0.10	1.6	0.02	1.8
A_55_P2142430	NM_001033350	Bank1	0.20	1.5	0.11	1.5	0.01	1.8
A_55_P2073377	NM_001081117	Mki67	0.96	1.0	0.88	1.0	0.00	1.8
A_51_P187750	NM_153408	Neurl3	0.88	1.1	0.11	1.3	0.00	1.8
A_51_P133137	NM_009004	Kif20a	0.61	1.2	0.22	1.2	0.00	1.8
A_55_P2117614	NM_028075	Tnfrsf13c	0.49	1.3	0.14	1.4	0.00	1.8
A_51_P378298	NM_026976	Faim3	0.82	1.2	0.07	1.6	0.04	1.7
A_55_P1964648	NM_001037719	Btla	0.77	1.2	0.11	1.6	0.03	1.7
A_55_P1974080	NM_001135127	LOC100189605	0.98	-1.0	0.94	1.0	0.03	1.7
A_55_P1970090	NM_010350	Grin2c	0.26	1.6	0.07	1.6	0.03	1.7
A_55_P2066230	NM_010407	Hck	0.41	1.4	0.22	1.4	0.02	1.7
A_66_P130916	NM_010389	H2-Ob	0.48	1.4	0.09	1.5	0.01	1.7
A_52_P675395	NM_007722	Cxcr7	0.60	1.3	0.43	1.2	0.01	1.7
A_51_P164014	NM_173762	Cenpe	0.97	-1.0	0.92	1.0	0.00	1.7
A_55_P2103706	XM_485921	Gm5593	0.80	1.1	0.35	1.2	0.00	1.7
A_55_P2025675	NM_013811	Dnahc8	0.34	1.3	0.06	1.4	0.00	1.7
A_55_P1996946	NM_023223	Cdc20	0.83	1.1	0.62	1.1	0.00	1.7
A_55_P1961270	NM_001110320	Cd72	0.13	1.5	0.10	1.4	0.00	1.7
A_55_P2173982	NM_009104	Rrm2	0.86	-1.1	0.89	1.0	0.00	1.7
A_52_P162099	NM_001004140	Ckap2	0.97	1.0	0.92	1.0	0.00	1.7
A_51_P212782	NM_008361	Il1b	0.28	1.4	0.47	1.2	0.00	1.7
A_55_P2040485	XM_889011	Ms4a4a	0.51	1.2	0.15	1.3	0.00	1.7
A_55_P2030938	NM_025863	Trim59	0.49	1.3	0.48	1.2	0.00	1.7
A_51_P302358	NM_008518	Ltb	0.49	1.4	0.26	1.4	0.04	1.6

A_55_P2079079	NM_009844	Cd19	0.29	1.5	0.06	1.6	0.04	1.6
A_51_P256827	NM_013650	S100a8	0.33	1.5	0.99	-1.0	0.04	1.6
A_51_P170959	AK005011	Proz	0.61	1.3	0.14	1.5	0.04	1.6
A_66_P106388	NM_029499	Ms4a4c	0.65	1.3	0.30	1.4	0.02	1.6
A_51_P279437	NM_029662	Mfsd2a	0.84	-1.1	0.87	-1.1	0.02	1.6
A_55_P2090025	NM_008590	Mest	0.80	1.2	0.09	1.5	0.02	1.6
A_55_P2002757	NM_008528	Blnk	0.33	1.4	0.19	1.4	0.02	1.6
A_51_P509263	NM_010455	Hoxa7	0.43	1.4	0.15	1.4	0.01	1.6
A_51_P305052	NM_172900	Siglecg	0.29	1.4	0.07	1.4	0.01	1.6
A_55_P2052062	NM_010818	Cd200	0.98	1.0	0.71	1.1	0.01	1.6
A_55_P1958275	NM_016707	Bcl11a	0.34	1.4	0.07	1.5	0.01	1.6
A_52_P588881	NM_001033484	Iqgap3	0.82	-1.1	0.79	1.1	0.00	1.6
A_51_P405397	NM_007899	Ecm1	0.78	1.1	0.00	1.4	0.00	1.6
A_55_P2470474	AK087205	9530082P21Rik	0.29	1.2	0.00	1.4	0.00	1.6
A_51_P464918	NM_019453	Mefv	0.28	1.3	0.21	1.2	0.00	1.6
A_51_P130079	NM_008511	Lrmp	0.25	1.4	0.09	1.4	0.00	1.6
A_30_P01027010			0.35	1.2	0.00	1.4	0.00	1.6
A_52_P625277	NM_183220	Accs	0.32	1.2	0.03	1.3	0.00	1.6
A_51_P498631	NM_018769	Dfna5	0.22	1.3	0.17	1.3	0.00	1.6
A_51_P336721	NM_025372	Tipin	0.42	1.2	0.13	1.2	0.00	1.6
A_55_P1988228	NM_009791	Aspm	0.95	1.0	0.64	1.1	0.00	1.6
A_55_P2169227	NM_177716	AI836003	0.63	1.2	0.06	1.4	0.00	1.6
A_55_P2157872	NM_001079695	Sfrs5	0.76	1.1	0.25	1.3	0.00	1.6
A_55_P2095271	NM_153805	Pkn3	0.29	1.2	0.06	1.3	0.00	1.6
A_55_P2010152	NM_001164059	Sell	0.71	1.2	0.62	1.2	0.04	1.5
A_55_P2035286	NM_010931	Uhrf1	0.74	-1.2	0.82	-1.1	0.04	1.5
A_55_P1976278	NM_008855	Prkcb	0.47	1.4	0.40	1.3	0.04	1.5
A_51_P247359	NM_016933	Ptprcap	0.43	1.3	0.19	1.4	0.04	1.5
A_55_P2088145			0.50	1.3	0.31	1.3	0.04	1.5
A_55_P2033600	NM_019992	Stap1	0.57	1.3	0.48	1.2	0.03	1.5
A_51_P272106	NM_007705	Cirbp	0.67	1.2	0.15	1.4	0.03	1.5
A_55_P1964664	NM_139149	Fus	0.68	1.2	0.45	1.2	0.02	1.5
A_55_P1958887	NM_001025384	DXBay18	0.70	1.2	0.27	1.3	0.02	1.5
A_66_P119034	NM_013737	Pla2g7	0.43	1.3	0.60	1.2	0.02	1.5
A_51_P230098	NM_023209	Pbk	0.85	-1.1	0.92	1.0	0.01	1.5
A_51_P149714	NM_026835	Ms4a6d	0.44	1.2	0.29	1.2	0.01	1.5
A_55_P1973770	NM_029770	Unc5b	0.91	1.1	0.10	1.3	0.01	1.5
A_55_P1960238	NM_172659	Slc2a6	0.58	1.2	0.51	1.2	0.01	1.5
A_51_P246677	NM_020002	Rec8	0.96	1.0	0.45	1.2	0.01	1.5
A_51_P267314	NM_178650	Tbc1d10c	0.29	1.4	0.14	1.4	0.01	1.5
A_51_P472217	NM_001081085	2010317E24Rik	0.82	1.1	0.90	-1.1	0.01	1.5
A_55_P2180415	NM_001110320	Cd72	0.26	1.4	0.27	1.3	0.01	1.5
A_66_P120987	NM_007655	Cd79a	0.33	1.3	0.34	1.2	0.01	1.5
A_55_P2062543	NM_053173	Kifc1	0.90	1.1	0.76	1.1	0.00	1.5
A_51_P249313	NM_028705	Herc3	0.57	1.2	0.23	1.2	0.00	1.5
A_55_P2004801	NM_001040435	Tacc3	0.98	1.0	0.65	1.1	0.00	1.5
A_55_P1973560			0.92	1.0	0.63	1.1	0.00	1.5
A_55_P1988048	NM_001163522	Emcn	0.61	1.2	0.02	1.4	0.00	1.5
A_55_P2037454	NM_023794	Etv5	0.26	1.2	0.01	1.4	0.00	1.5

A_55_P2040805	NM_130903	Cd209c	0.00	1.4	0.02	1.3	0.00	1.5
A_51_P240453	NM_133851	Nusap1	0.91	1.1	0.71	1.1	0.00	1.5
A_52_P325527	NM_010103	Edil3	0.98	-1.0	0.04	1.4	0.00	1.5
A_51_P209327	NM_013912	Apln	0.99	1.0	0.09	1.3	0.00	1.5
A_30_P01023554			0.74	1.1	0.37	1.2	0.00	1.5
A_51_P204402	NM_011369	Shcbp1	0.94	-1.0	0.79	1.1	0.00	1.5
A_51_P487999	NM_028232	Sgol1	0.96	1.0	0.84	1.1	0.00	1.5
A_55_P1955998	NM_001004142	Nlrp1a	0.13	1.4	0.05	1.4	0.00	1.5
A_52_P51429	NM_153551	Dennd1c	0.36	1.3	0.13	1.3	0.00	1.5
A_55_P2304507	NM_172204	Noxa1	0.31	1.3	0.81	1.1	0.00	1.5
A_51_P449824	XM_001471750	Exoc3l2	0.84	1.1	0.35	1.2	0.00	1.5
A_51_P329928	NM_013750	Phlda3	0.77	1.1	0.00	1.4	0.00	1.5
A_55_P2018847	NM_001164735	Crlf2	0.00	1.3	0.00	1.4	0.00	1.5
A_55_P2017759	NM_001033711	Evi2a	0.41	1.3	0.37	1.2	0.00	1.5
A_55_P2127702	NM_012025	Racgap1	1.00	1.0	0.49	1.1	0.00	1.5
A_52_P527800	NM_145158	Emilin2	0.19	1.3	0.29	1.2	0.00	1.5
A_51_P488554	NM_026543	3010026O09Rik	0.22	1.2	0.00	1.4	0.00	1.5
A_30_P01023785			0.57	1.2	0.00	1.4	0.00	1.5
A_55_P2087528	NM_172681	D930015E06Rik	0.00	1.4	0.03	1.3	0.00	1.5
A_55_P1967978	NM_001136073	Nfatc2	0.25	1.3	0.48	1.2	0.00	1.5
A_52_P469789	NM_007588	Calcr	0.90	1.1	0.44	1.2	0.00	1.5
A_51_P451151	NM_026785	Ube2c	0.84	1.1	0.32	1.2	0.00	1.5
A_51_P455807	NM_133838	Ehd4	0.66	1.2	0.04	1.4	0.00	1.5
A_55_P1985850	NM_001044384	Timp1	0.32	-1.4	0.06	-1.5	0.04	-1.5
A_55_P2062070	NM_001081064	Pdzd2	0.61	-1.2	0.77	-1.1	0.03	-1.5
A_51_P321341	NM_133670	Sult1a1	0.53	-1.3	0.19	-1.3	0.03	-1.5
A_51_P284823	NM_009086	Polr1b	0.86	-1.1	0.34	-1.3	0.03	-1.5
A_51_P245414	NM_010639	Klk1	0.82	-1.1	0.54	-1.2	0.02	-1.5
A_51_P234253	NM_145535	Sdcbp2	0.69	-1.2	0.37	-1.2	0.02	-1.5
A_52_P444946	NM_001033212	Rprml	0.97	-1.0	0.96	1.0	0.02	-1.5
A_51_P158678	NM_021427	Fam181b	0.43	-1.3	0.17	-1.3	0.01	-1.5
A_55_P2157023	NM_011441	Sox17	0.79	-1.1	0.49	-1.2	0.01	-1.5
A_30_P01032480			0.79	-1.1	1.00	-1.0	0.01	-1.5
A_51_P484842	NM_007493	Asgr2	0.83	-1.1	0.30	-1.2	0.01	-1.5
A_30_P01027526			0.67	-1.1	0.14	-1.2	0.00	-1.5
A_51_P112762	NM_017391	Slc5a3	0.65	-1.1	0.01	-1.3	0.00	-1.5
A_55_P2072453	NM_001081361	Mosc1	0.00	-1.4	0.01	-1.4	0.00	-1.5
A_55_P2334484	BE650233	D9Ertd115e	0.88	-1.1	0.11	-1.3	0.00	-1.5
A_66_P105422	NM_028894	Lonrf3	0.65	-1.2	0.12	-1.3	0.00	-1.5
A_55_P2181597	NM_008546	Mfap2	0.68	-1.1	0.10	-1.2	0.00	-1.5
A_51_P224311	NM_022318	Popdc2	0.63	-1.2	0.63	-1.1	0.00	-1.5
A_51_P208931	NM_145974	C330016O10Rik	0.52	-1.2	0.17	-1.2	0.00	-1.5
A_52_P161495	NM_009744	Bcl6	0.61	-1.2	0.19	-1.2	0.00	-1.5
A_55_P2126557	XM_619973	Gm5858	0.95	-1.0	0.78	-1.1	0.00	-1.5
A_55_P2045055	NM_010796	Clec10a	0.61	-1.2	0.02	-1.4	0.00	-1.5
A_55_P2052623	NM_145583	Pgap2	0.57	-1.2	0.22	-1.3	0.00	-1.5
A_52_P33382	XM_356935	Gm5226	0.56	-1.3	0.34	-1.3	0.04	-1.6
A_52_P566681	NM_153581	Gpm6a	0.58	-1.3	0.30	-1.3	0.03	-1.6
A_55_P2161410	NM_009253	Serpina3m	0.89	1.1	0.19	-1.4	0.02	-1.6

A_55_P2035946	NM_001002927	Penk	0.15	-1.6	0.06	-1.5	0.02	-1.6
A_55_P2058201	NM_001009952	Abpd	0.40	-1.4	0.16	-1.4	0.01	-1.6
A_51_P129803	NM_009786	Cacybp	0.65	-1.2	0.06	-1.4	0.01	-1.6
A_52_P650387	NM_001045530	Ccnjl	0.33	-1.3	0.10	-1.4	0.01	-1.6
A_55_P2080603	NM_031165	Hspa8	0.74	-1.2	0.13	-1.4	0.01	-1.6
A_55_P2069721	NM_031165	Hspa8	0.78	-1.2	0.19	-1.4	0.01	-1.6
A_55_P1985764	XM_001479044	LOC100047875	0.74	-1.2	0.05	-1.4	0.01	-1.6
A_55_P2078138	NM_172709	Otop1	0.50	-1.3	0.34	-1.3	0.01	-1.6
A_30_P01018165			0.14	-1.3	0.00	-1.4	0.00	-1.6
A_55_P2062793	NM_008546	Mfap2	0.30	-1.2	0.00	-1.4	0.00	-1.6
A_30_P01026318			0.91	-1.1	0.53	-1.2	0.00	-1.6
A_51_P116651	NM_019759	Dpt	0.69	-1.3	0.44	-1.3	0.04	-1.7
A_30_P01029503			0.20	-1.7	0.39	-1.4	0.03	-1.7
A_51_P444447	NM_007679	Cebpd	0.36	-1.5	0.10	-1.5	0.02	-1.7
A_55_P2408588	NM_007489	Arntl	0.88	-1.1	0.17	-1.4	0.01	-1.7
A_52_P671812	XM_918536	LOC641192	0.58	-1.2	0.12	-1.4	0.00	-1.7
A_30_P01023143			0.24	-1.3	0.05	-1.3	0.00	-1.7
A_55_P1974522	NM_175688	A530099J19Rik	0.12	-1.3	0.01	-1.4	0.00	-1.7
A_55_P1968304			0.59	-1.2	0.06	-1.4	0.00	-1.7
A_55_P1980883	NM_001146351	Ephb6	0.20	-1.3	0.01	-1.4	0.00	-1.7
A_55_P2444965	NM_175518	D730040F13Rik	0.75	-1.2	0.70	-1.1	0.00	-1.7
A_52_P12877	NM_031165	Hspa8	0.77	-1.2	0.27	-1.4	0.00	-1.8
A_55_P2292046	AK084291	D230018H15Rik	0.61	-1.2	0.06	-1.4	0.00	-1.8
A_55_P1989061	NM_001077364	Tsc22d3	0.33	-1.6	0.07	-1.7	0.01	-1.9
A_51_P127681	NM_013885	Clic4	0.97	-1.1	0.19	-1.7	0.03	-2.0
A_55_P2131766	NM_023665	D4Wsu53e	0.85	-1.2	0.12	-1.8	0.02	-2.0
A_51_P356055	NM_175012	Grp	0.80	-1.2	0.20	-1.4	0.00	-2.1
A_51_P195374	NM_025833	Baiap2l1	0.95	-1.1	0.97	-1.0	0.02	-2.2
A_51_P338443	NM_020581	Angptl4	0.41	-1.7	0.65	-1.3	0.01	-2.2
