# Supplementary material for: Comparative transcriptomic analyses to scrutinize the assumption that genotoxic PAHs exert effects via a common mode of action
Source: Arch Toxicol. 2015 Sep 16;90(10):2461–80. doi: 10.1007/s00204-015-1595-5 (PMC5043007; doi:10.1007/s00204-015-1595-5)
Supplement: Supplementary file 4 — Supplementary material 4 (PDF 254 kb) [file 204_2015_1595_MOESM4_ESM.pdf]

Article Title: Comparative Transcriptomic Analyses to Scrutinise the Assumption That Genotoxic Priority PAHs Exert Effects via a Common Mode of Action
Journal: Archives in Toxicology
Authors: Labib S, Williams A, Guo CH, Leingartner K, Arlt VM, Schmeiser HH, Yauk CL, White PA, Halappanavar S*
Corresponding Author: *Sabina Halappanavar, Health Canada, Sabina.halappanavar@hc-sc.gc.ca

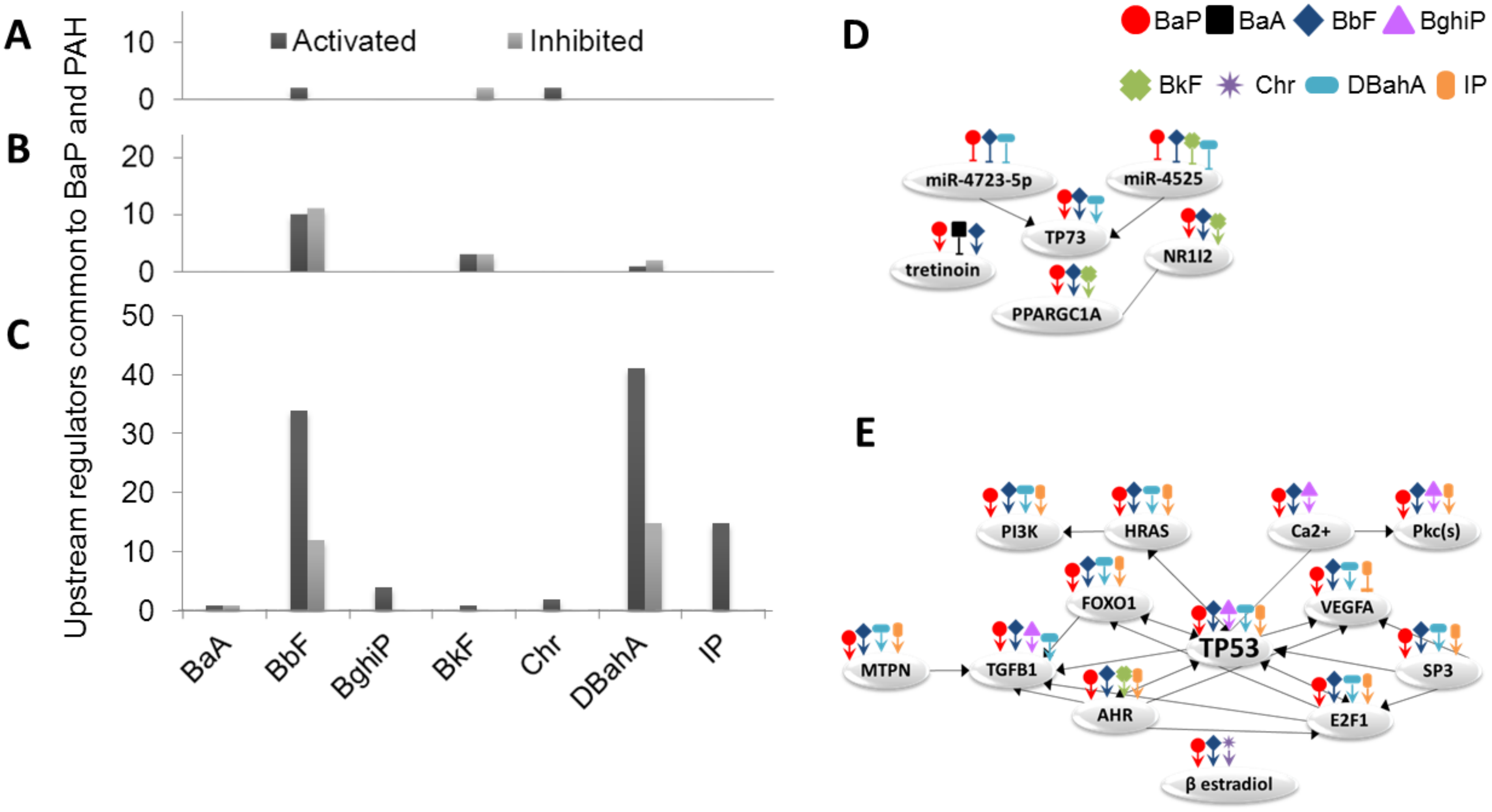

Supplementary File 6 – IPA upstream regulator analysis. Graphs show number of upstream regulatory molecules common to each PAH and BaP in the (A) forestomach, (B) liver , and (C) lung. (D-E) Network of upstream regulators common to the PAHs and BaP in the (D) liver and (E) lung.
