# Supplementary material for: Comparative transcriptomic analyses to scrutinize the assumption that genotoxic PAHs exert effects via a common mode of action
Source: Arch Toxicol. 2015 Sep 16;90(10):2461–80. doi: 10.1007/s00204-015-1595-5 (PMC5043007; doi:10.1007/s00204-015-1595-5)
Supplement: Supplementary file 5 — Supplementary material 5 (PDF 274 kb) [file 204_2015_1595_MOESM5_ESM.pdf]

Article Title: Comparative Transcriptomic Analyses to Scrutinise the Assumption That Genotoxic Priority PAHs Exert Effects via a Common Mode of Action
Journal: Archives in Toxicology
Authors: Labib S, Williams A, Guo CH, Leingartner K, Arlt VM, Schmeiser HH, Yauk CL, White PA, Halappanavar S*
Corresponding Author: *Sabina Halappanavar, Health Canada, Sabina.halappanavar@hc-sc.gc.ca

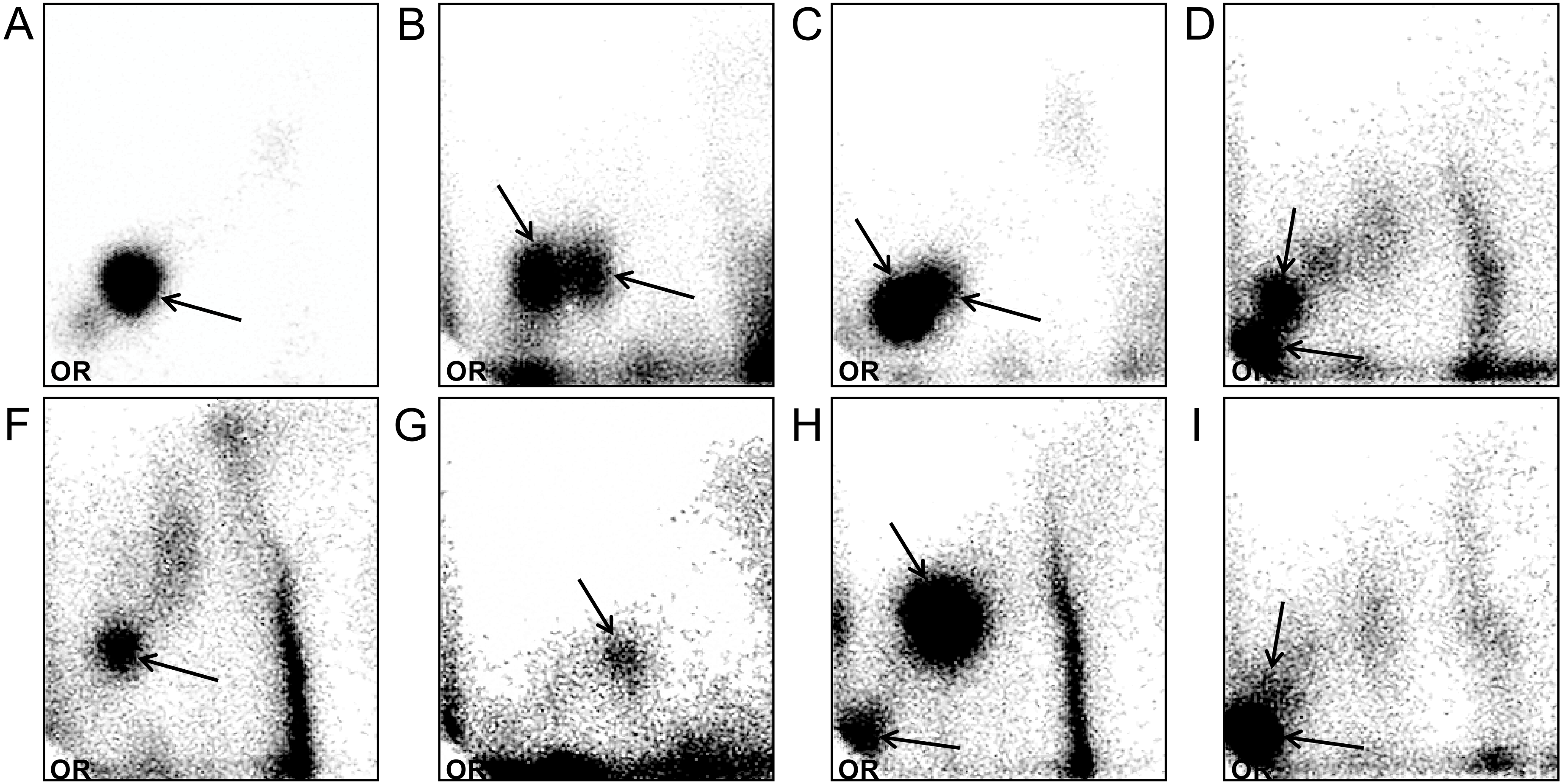

Supplementary File 7 - Representative autoradiographic profiles of DNA adducts, measured by ^{32}P-post-labeling, in lungs from Muta^{TM}Mouse subchronically exposed to BaP (A), BaA (B), BbF (C), BghiP (D), BkF (F), Chr (G), DBahA (H) and IP (I). Solvent conditions for the separation of PAH-derived DNA adducts using thin-layer chromatography were as follows: D1, 1.0 M sodium phosphate, pH 6.0; D3, 3.5 M lithium-formate, 8.5 M urea, pH 3.5; D4, 0.8 M lithium chloride, 0.5 M Tris, 8.5 M urea. The origins (OR), at the bottom left corner, were cut off before imaging. Arrow(s) indicate major adduct spot(s) used to calculate DNA adduct level(s).
